# Comparison of alternative approaches for analysing multi-level RNA-seq data

**DOI:** 10.1371/journal.pone.0182694

**Published:** 2017-08-08

**Authors:** Irina Mohorianu, Amanda Bretman, Damian T. Smith, Emily K. Fowler, Tamas Dalmay, Tracey Chapman

**Affiliations:** 1 School of Biological Sciences, University of East Anglia, Norwich Research Park, Norwich, United Kingdom; 2 School of Computing Sciences, University of East Anglia, Norwich Research Park, Norwich, United Kingdom; 3 School of Biology, University of Leeds, Leeds, LS2 9JT, United Kingdom; Niels Bohr Institute, DENMARK

## Abstract

RNA sequencing (RNA-seq) is widely used for RNA quantification in the environmental, biological and medical sciences. It enables the description of genome-wide patterns of expression and the identification of regulatory interactions and networks. The aim of RNA-seq data analyses is to achieve rigorous quantification of genes/transcripts to allow a reliable prediction of differential expression (DE), despite variation in levels of noise and inherent biases in sequencing data. This can be especially challenging for datasets in which gene expression differences are subtle, as in the behavioural transcriptomics test dataset from *D*. *melanogaster* that we used here. We investigated the power of existing approaches for quality checking mRNA-seq data and explored additional, quantitative quality checks. To accommodate nested, multi-level experimental designs, we incorporated sample layout into our analyses. We employed a subsampling without replacement-based normalization and an identification of DE that accounted for the hierarchy and amplitude of effect sizes within samples, then evaluated the resulting differential expression call in comparison to existing approaches. In a final step to test for broader applicability, we applied our approaches to a published set of *H*. *sapiens* mRNA-seq samples, The dataset-tailored methods improved sample comparability and delivered a robust prediction of subtle gene expression changes. The proposed approaches have the potential to improve key steps in the analysis of RNA-seq data by incorporating the structure and characteristics of biological experiments.

## Introduction

RNA sequencing (RNA-seq) has revolutionized the field of transcriptomics [1, 2], giving powerful insight into the identity and abundance of RNAs in cells, tissues and whole organisms [3]. In contrast to the fixed, predefined set of probes used for microarray experiments, RNA-seq generates a diverse set of reads and facilitates analyses of expression level variation for known and unknown RNA transcripts and variants. It also offers the possibility to study additional facets of the transcriptome [4], such as those arising from (re)annotations of reference genomes [5], or to identify alternative splicing events [6] and variation in abundance across transcripts [7, 8]. Several bioinformatics methods have been developed for the analysis of the rapidly rising number of RNA-seq datasets (reviewed in [4, 7, 9]). However, to accommodate the use of RNA-seq in complex experimental designs, there is scope for further advances in: (i) minimising the effect of normalisation on the pattern of DE, hence facilitating the robust detection of subtle signatures of gene expression, the concordance of which is often very low between different bioinformatics methods [10, 11]; (ii) the incorporation of hierarchical (e.g. nested) experimental designs [12–14], e.g. as often used in evolutionary experiments [15].

### Quality checks (QC)

A key step in the analysis of RNA-seq data consists of sample quality checks and the identification, characterization and potential exclusion of sample outliers, e.g. those samples that are compromised due to technical issues [16]. Tools such as FastQC [17], SeqMonk [17] or TagCleaner [18] evaluate the sequencing and per-base quality. Additional QC may include an analysis of the per-base nucleotide composition and an evaluation of overall GC content [19–21]. QC procedures focused on sequencing bias include the characterization of k-mer distributions [22] as well as detection of other adapter ligation effects [23, 24].

Quantitative analysis of sequencing output currently considers measures such as yield, coverage, 3’/5’ bias, number of detectable transcripts, strand specificity and read distribution across the genome [16]. Additional steps, at the transcript level, include the classification of reads into annotation classes, which can highlight the presence of potential contaminant ncRNAs such as tRNAs and rRNAs [19, 21]. Such analyses can reveal over-represented classes of sequences, which can then be removed to minimise distortion in the subsequent normalization and reduce changes in the ranking of abundances.

A QC criterion often used is the Pearson Correlation Coefficient (PCC) between the expression levels of genes across replicates [25, 26]. Values of *r*^2^ ∈ [0.92,0.98] are considered acceptable. If the PCC falls below 0.9 the suggestion is to exclude the problematic replicates/samples [4]. However, this criterion can lack discriminatory power, because, due the high number of data points (expressed genes, e.g. vector containing >15K genes for *D*. *melanogaster*), the resulting correlations will often be very high between *all* samples.

We suggest that there are some potentially useful, and as yet under-utilized, additional steps for quantitative evaluation of RNA-seq samples. These include analyses of per-sample or per-gene complexities, defined as the ratio of non–redundant (NR, unique) to redundant (R, total) reads [27], similarity comparisons [28, 29] and enhanced PCC-based analyses. Complexity is an informative measure of the number of unique reads, average abundance of reads per transcript and average coverage (a complexity of ~0 would indicate a sample in which all reads were the same and 1 a sample where every read was different). Sample complexity is influenced by sequencing depth. Samples with high sequencing depth have a lower overall complexity and vice versa. However, samples with comparable sequencing depths, but very different numbers of unique reads, can suggest the presence transcripts/replicates that are not comparable. Other quantitative checks include the Jaccard similarity index [27, 28], which evaluates the proportion of the top most abundant genes that are shared across samples. Because this index is calculated on highly-expressed genes it is not biased by low level, noise-derived, variability in expression.

Additional insight into the reproducibility of gene quantification can be achieved by using the ‘point-to-point’ Pearson correlation coefficient (p2pPCC). A standard PCC uses one expression value for each gene (the sum of abundances of all incident reads). In contrast, the p2pPCC is calculated using the distribution of expression across the whole transcript. For example, consider a gene with 2 exons (A, B). In sample 1, A has abundance of 5 and B of 0. In sample 2, A has 0 and B has 5. The standard PCC = 1 and the expression in the two samples is correlated. However, the p2pPCC = 0 indicating that, in reality, the distribution of expression is not the same. Hence, the p2pPCC can provide an accurate evaluation of replicate-to-replicate variability. However, its accuracy falls if alternative splicing events are common or variable in the samples being compared. However, alternative splicing appears more prevalent between different tissues and developmental stages than between environmental conditions or manipulations [30] such as those compared in this study.

### Normalizations

The next key stage in the analysis of RNA-seq data is the normalization of gene expression levels [4, 11, 31], [7, 32]. Normalization is designed to transform the distributions of abundances for each sample, without distortion, into distributions that can be compared. An effective normalization increases the chances of an accurate call of DE. It accounts for differences in sequencing depths and in biases arising from the library preparation or its sequencing [33–35]. Nevertheless, despite extensive attention from the community [36], there is as yet no clear consensus on whether any single normalization method is optimal [7, 11]. Nor is there any general appreciation of the potential magnitude of the consequences of ineffectively normalizing data. The extent of this problem depends on the amplitude and distribution of DE, with small gene expression differences being more sensitive than larger ones to the method of normalization. Therefore, particularly for analyses of subtle gene expression differences, as in the *D*. *melanogaster* test data used here, it can be important to assess how well the data are normalized by different methods [37]. However, such tests are not a routine part of bioinformatics analyses [9].

In this study, in addition to the extra QC described above, we used a normalization based on subsampling without replacement [38]. This was subsampling of the original set of reads, to a smaller, predetermined and fixed total. Each read that was selected and assigned to the normalized sample was not returned to the original pool (‘without replacement’), hence could not be selected again. We also conducted additional checks and downstream enhancements in the calculation of DE [39]. We chose this normalization method because subsampling normalizations are distribution-free and circumvent the need of scaling factors, which lack consistency across all abundances and can lead to downstream biases [38, 40]. A subsampling *with* replacement normalisation (in which reads that are selected and assigned to the normalized sample are returned to the original pool and hence can be selected again) has previously been proposed [25, 40, 41]. The main difference between the two approaches is that ‘with replacement’, the probabilities for selecting reads remain constant throughout subsampling, whereas for ‘without replacement’ they do not and are updated as the subsampling progresses. Subsampling with replacement [40] was introduced for its potential suitability for samples with low sequencing depth that could be subsampled to higher total read number. However, this may over-amplify high abundance and exclude low abundance reads, with attendant downstream knock-on effects. To our knowledge, no direct comparison of the two subsampling approaches to RNA-seq, has yet been conducted.

### Identification of differential expression

The goal of transcriptomics analyses is the accurate and unbiased identification of expressed genes and genes showing DE between treatments. The majority of existing methods exhibit a good level of overlap in terms of highly differentially expressed genes [11, 42]. However, the agreement is far less when DE is subtle. Comparative analyses of existing normalization procedures on real and simulated data sets show that only ~50% of significantly differentially expressed genes are identified by all methods [10, 35].

ANOVA-based methods are a powerful and extensively applied approach for the analysis of microarray data [43]. However, such methods are based on *a priori*, to some extent arbitrary, significance thresholds. In addition, the type of experiment can greatly influence the expected number of genes showing DE. For example, if the frequency distribution of DE is narrow, *P*-values will indicate as statistically significant genes that typically show only very small fold change differences. Such differences are also unlikely to be validated by low throughput methods [9]. Therefore, the set of DE genes identified by a fixed *P*-value may not necessarily reflect biologically important facets of the data.

Newer methods such as DESeq2 [12] and edgeR [44] are based on the negative binomial distribution model for expression levels, using the variance and mean linked by local regression to detect DE genes (DESeq2) and empirical Bayes methods for moderating the degree of over dispersion across transcripts (edgeR). However, additional options for analysis could be useful to accommodate hierarchical experimental designs [45], e.g. those in which the different levels of an experiment are nested within each other.

In the *D*. *melanogaster* study that provided the test data examined here, we applied both known and novel qualitative and quantitative quality checks for the analysis of a hierarchical RNA-seq dataset of the behavioural responses to conspecific rivals in *D*. *melanogaster* [39]. We accounted for the different hierarchical levels of the experimental design (i.e. body part, then presence or absence of rivals) by including the magnitude of gene expression differences at each level into our analysis, prior to the DE call. We structured the analysis for the mRNA-seq data (S1 Fig) and conducted QC tests, a subsampling without replacement-based normalization and finally a hierarchical approach for the identification of transcripts showing DE. The analysis also featured the use of an adjustable, empirically-determined offset to filter out low abundance genes and a DE call based on maximal confidence intervals. We then compared the adapted normalization method to existing approaches in the analysis of an additional, publicly available, *H*. *sapiens* mRNA-seq dataset [46]. Overall, the adapted methods complemented existing approaches and performed well in the analysis of complex, challenging datasets.

## Results and discussion

We first used the *D*. *melanogaster* dataset from [39] in which we analysed the subtle effects on gene expression of exposure of males to conspecific mating rivals. The steps of this analysis (S1 Fig) followed the approaches described in [4]. We then compared the output of our pipeline with that of existing methods on the same input data. The final step was to assess the broader applicability of our adapted normalization to a publicly available *H*. *sapiens* mRNA-seq dataset [46].

### Quality checking

#### Stage 1: QC of sequencing quality

The *D*. *melanogaster* test data comprised of 3 replicate mRNA-seq samples of 2 rival treatments (rivals versus no rivals), 2 body parts (Head+Thorax (HT) and Abdomen (A)) and one exposure treatment (2h after the exposure to conspecific rivals). The first stage of the QC (S1 Fig) focussed on existing approaches and comprised of the analysis of: (i) FastQ quality scores [17], (ii) sequencing depth, (iii) nucleotide (nt) composition / GC content [19, 21], (iv) strand bias and (v) proportions of genome and annotation classes—matching reads e.g. mRNAs, t/rRNAs, miRNAs, UTRs, introns, intergenic regions [4]. The FastQ QC indicated good quality reads for all 50nt, though we observed high variability in sequencing depths. Variation in nucleotide content was observed across the first 12nt [24], but conformed after that to the nucleotide composition of the *D*. *melanogaster* transcriptome. Strand bias was comparable across samples and the proportion of genome-mapping reads was high. Based on these stage 1 quality checks, all samples were retained for further analyses and entered stage 2 QC. The detailed results, supporting the conclusion that the samples were consistent based on these criteria, are presented in S1 and S2 Tables.

#### Stage 2: Quantitative QC of replicate and sample comparability

We computed the Jaccard similarity [27, 28] at the gene level, to compare the similarity in expression of the top 1000 most abundant genes present in each sample (S3 Table). Samples drawn from the same body parts shared > 90% similarity, and between body parts (HT versus A) the similarity dropped to ~50%. Similarity between the experimental (± rivals) treatments was sometimes higher than between replicates, which highlighted the need for an alternative approach to normalize gene expression levels.

**Fig 1:**
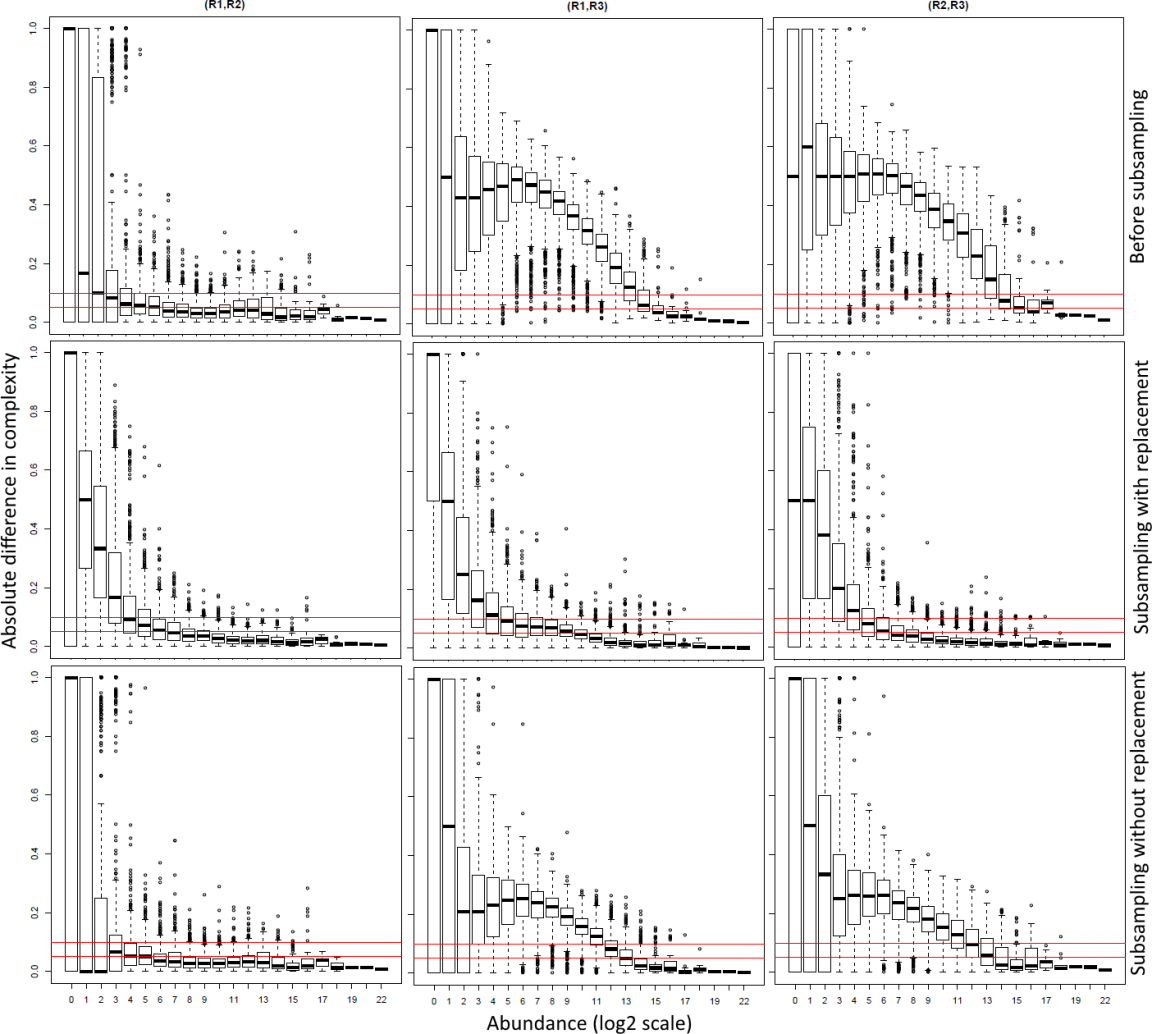
Differences in gene level complexity between replicates in the *D*. *melanogaster* mRNA-seq data. X-axis shows binned transcript abundances (log_2_ scale) and the Y-axis the absolute difference in complexity (non-redundant/redundant, NR/R ratio) between biological replicates: left column, replicate 1 vs 2 (R1,R2); middle, replicate 1 vs 3 (R1,R3); right, replicate 2 vs 3 (R2,R3). The differences in complexities were calculated on the raw data (top row), the data after subsampling normalization with replacement (middle row) and without replacement (bottom row). To ease visual comparison, the red horizontal lines indicate 0.05 and 0.1 complexity difference. In the raw data, complexity differences were high for the R1,R3 and R2,R3 plots. Both subsampling approaches (with or without replacement) reduced complexity differences across all transcript abundances. The subsampling without replacement suggested that the third replicate (R3) was an outlier (‘hump’ in complexity remaining in R1,R3 and R2,R3 comparisons), whereas the subsampling with replacement did not. The example data shown are for the three biological replicates of the 02+H (2 hours, rivals present, head thorax) samples of the *D*. *melanogaster* mRNA-seq data.

As noted in the introduction, samples with comparable sequencing depths, but very different number of unique reads, may represent non-comparable replicates. To understand how this may influence the accuracy of DE, we calculated the variation in complexity, between replicates at gene level (Fig 1). Highly comparable replicates should exhibit minimal differences in complexity across all levels of abundance (i.e. form a flat horizontal line of low complexity differences). However, in the non-normalized data (Fig 1A) there were sizeable differences in complexity for most genes, especially in the comparisons between replicates 1 vs 3 and 2 vs 3. The differences in the samples appeared to be due to the presence of highly variable numbers of spurious reads, especially for low abundance genes, a conclusion also supported by the point-to-point correlation (see below). To test whether the normalization would flatten out and reduce this technical variation we tested the effect of subsampling with (Fig 1B) and without (Fig 1C) replacement. This resulted in an overall reduction in complexity differences, and hence an increase in similarity between replicates. However, the two methods showed different results for replicate 3 (R3). Subsampling without replacement appeared to most reduce complexity differences (Fig 1C) and indicated that all replicates were acceptable. However, our analyses indicated that the calling of R3 as an outlier by the subsampling without replacement method (Fig 1C) was actually more accurate. This is described in more detail under normalization, below.

Correlations were first calculated between gene expression vectors in each sample to assess their comparability (using Pearson (PCC), Spearman (SCC) and Kendall (KCC) correlation coefficients) as in [25]. Using the raw expression levels, correlations were computed between each sample and every other. Correlations between HT and A samples (in the range of 0.75–0.8) were lower than correlations between same body part samples. This was expected on the basis of HT- and A-specific genes whose expression is restricted to each body part. When only A or HT samples were considered, all correlation coefficients were above 0.95 (S2 Fig), even though, based on other quantitative QC measures, some samples represented potential outliers (see below). Hence, these standard correlation metrics may not be sufficiently sensitive to fully evaluate sample quality.

Next, we tested the correlation in gene expression versus gene abundances between the different replicates. This analysis revealed low correlation (PCC) and high variation between replicates at low gene abundance (S3 Fig). This was evident in the subsequent analysis as greater DE at low abundance. The p2pPCC was then used to determine a noise threshold, or “offset”, below which replicates were not correlated and hence data should not be considered. Noise between replicates occurs for low abundance genes because these have only a few reads that align to random locations on the gene and these locations are likely to differ between replicates, resulting in low p2pPCC. As abundance increases, the alignments ‘pile up’ and become more similar across the whole gene in each replicate leading to an increase in the p2pPCC. Hence the value of the p2pPCC can be used to determine an offset, which we defined here as the gene abundance for which the median p2pPCC > 0.7 between replicates. A similar approach, based on the entropy of strand bias, was implemented for sRNA sequencing in [28].

Overall the additional quantitative QC metrics we applied, which focused on the comparability at gene level by analyses of complexity and similarity, had good discriminatory power and represented a potentially valuable addition to overall QC.

### Normalization

To attenuate the effect of the variable sequencing depth between replicates and samples, we implemented a subsampling (without replacement) normalization on read expression levels (adapted from [40]), which we enriched with additional checks on the per gene consistency of the subsample plus its similarity to the original sample. Existing subsampling approaches have employed subsampling with replacement, applied on the aligned reads [25], on gene abundances [41], or subsampling without replacement applied on all reads [38].

First we tested the homogeneity of each sample [38]. To check for the presence of high abundance reads which, due to their higher probability of being selected, could distort the normalized distributions, we conducted a subsampling exercise, from 95% down to 45% (in steps of 5%) of the original redundant reads. We undertook an additional step to assess the consistency of each subsample by checking if the proportion of redundant genome matching reads had been affected by the subsampling (S4 Table and S4 Fig). We found that even when the data were subsampled to 45% of the original sequencing depth, the proportion of redundant genome matching reads remained unchanged. However, the complexity of the sample increased and became comparable to other samples with similar number of reads.

Next we evaluated the extent to which the data could be subsampled without affecting its structure. We calculated the point-to-point PCC on expression levels of the original versus subsampled data from 95% to 40% of the original R set (S4 Fig). This showed that the correlations of abundantly expressed genes remained high over all subsamples, but that the correlation of low abundance transcripts decreased as the proportion of data subsampled dropped (note though that the variability between the original versus subsamples was lower than the variability between the biological replicates). We concluded that the subsampling was effective as it maintained high p2pPCC and strong concordance between the expression levels of the raw versus normalized data (S4 Fig).

The number of genes ‘lost’ due to the exclusion of some low abundance reads was <2%. Once we had determined that all samples passed the consistency check, we subsampled every sample to a fixed total of 50M reads and checked whether subsamples were representative of the original data using bootstrapping (S1 Methods). Following this step, one subsample was selected at random for each sample and used in the subsequent downstream analysis. This subsampling was efficient at correcting wide variation in read number, complexity differences and minimising the impact of normalization on the original data structure (Fig 1).

The analysis of the distributions of complexity differences between replicates, coupled with the Jaccard similarity analyses applied on the normalized data, was used to identify outlier replicates, which were excluded from subsequent analyses. We classified as outliers, samples for which the between-replicate similarity was higher than between-sample similarity, as shown by the Jaccard, complexity and p2pPCC analyses. In the 02+H example data (Fig 1), the subsampling without replacement correctly highlighted replicate 3 as an outlier, while subsampling with replacement did not. Following this post-normalization QC, we retained two biological replicates for each treatment for downstream analysis data. In general, we advocate the use of as many biological replicates as possible. However, as in [39], the analysis of subtle gene expression even with a limited number of replicates is possible and can be validated. To exclude the little variation that remained in the samples, using the summarised expression levels, we applied a further quantile normalization to our data for subsequent downstream analyses. This correction did not change the distributions of gene abundances.

### Subsampling with replacement vs subsampling without replacement

**Fig 2:**
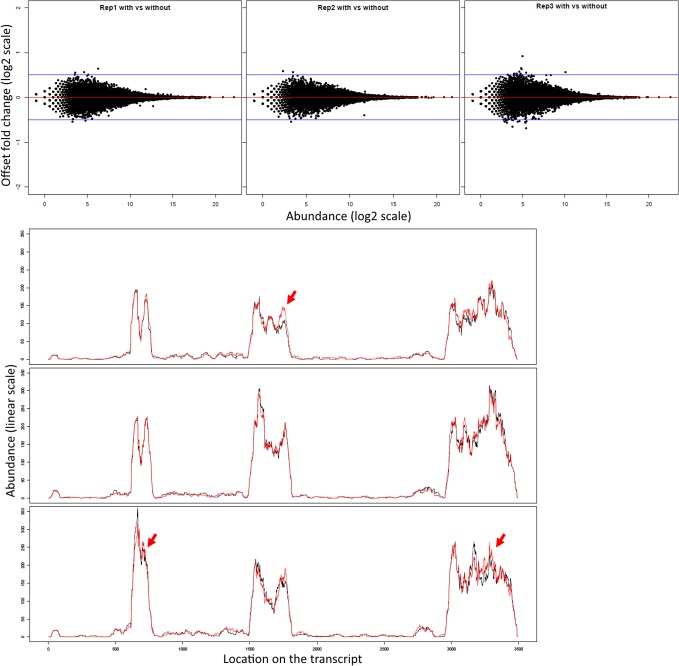
Comparison of results obtained using the subsampling with or without replacement normalization. In the top panel we present the MA plots on the gene expression levels, normalized using either the with- or without- replacement approaches, for the three replicates of the 02+H sample (02h, rivals, HT body part). Although the variability between the two approaches was contained within the +/- 0.5 log_2_(OFC) boundary, we observed a higher variability in expression for the low abundance genes. The bottom panel shows the presence plots for the gene FBgn0033865 for each of the three replicates (on individual panels) obtained using either the subsampling without replacement (black solid line) or subsampling with replacement (red solid line). The arrows indicate the regions where the two approaches provided different answers. The arrow indicating the first exon of the gene highlighted the difference observed for the third replicate (02+H, rep3).

We compared the effect of the subsampling normalization with and without replacement. The MA plots were used to compare the with- versus without- subsampling, on the same replicates, to the same total (Fig 2A). This showed that the two approaches agreed for higher abundances (>2^10^) but exhibited some variability for the lower abundance range. The presence plots, on the three 02+H sample replicates for an example gene (FBgn0033865) exhibited 0.4 difference in complexity between the two approaches (Fig 2B). This highlighted that the omission of the low abundance reads changed the expression profiles across transcripts (differences indicated by the red arrows). In addition, the change in expression profile for the third replicate, visible on the first exon, supported the earlier indication that this replicate was an outlier.

The direct comparison between the with- and without- replacement approaches illustrated that the resulting expression levels differed. Specifically, the subsampling without replacement correctly identified an outlier replicate, whereas the with-replacement subsampling masked this result.

### Calling of DE using a hierarchical approach

To incorporate the different levels (body part, presence or absence of rivals) of the experimental design into the DE call [45] we used a hierarchical approach for the prediction of DE transcripts (S2 Methods). The order of levels in the hierarchy was determined based on the magnitude of DE for each level in the experiment (S5 Fig). For the *D*. *melanogaster* dataset the highest level (i.e. that showed most DE) was body part (HT vs A), the second was ± rivals treatment. The distribution of DE between treatments and between replicates overlapped (S5A and S5B Fig), which indicated that the treatment DE was subtle.

**Fig 3:**
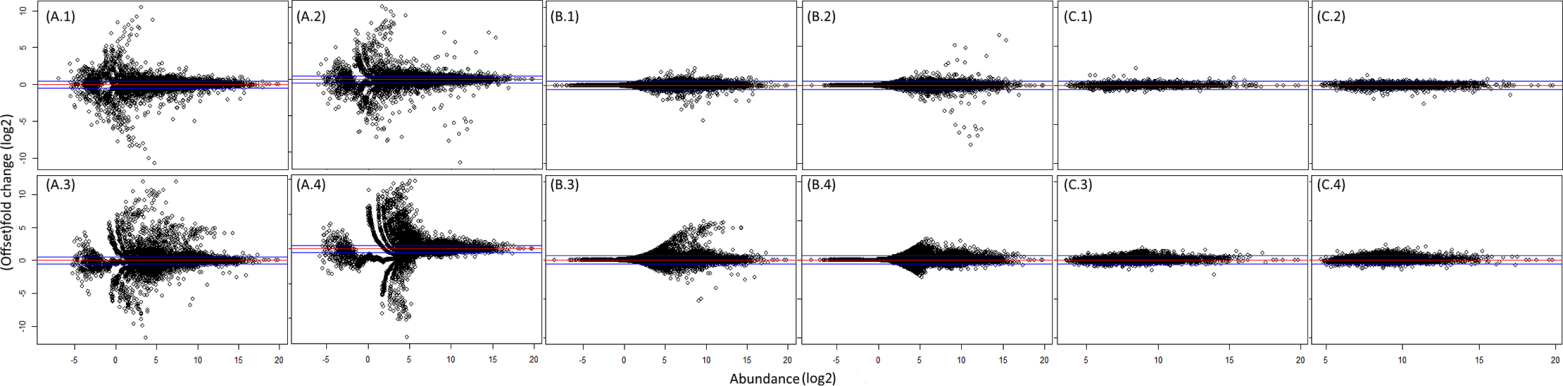
Distribution of DE as calculated by using fold change (FC) versus offset fold change (OFC) and the effect of incorporating hierarchical DE (HDE). Shown are MA plots (x-axis showing gene abundance (log_2_), y-axis indicating FC/OFC for replicate-to-replicate comparisons for the 2h samples. Panels A1, B1, C1 show 02-A comparisons, panels A2, B2, C2 02+A samples, A3, B3, C3 for 02-H and A4, B4, C4 for 02+HT samples (sample codes: 02 = 2h of exposure, A = abdomen, HT = head-thorax, + = with rivals,— = without rivals). Panel A shows the distribution of DE calculated using FC, highlighting how the low abundance genes distorted the distribution of DE. Panel B shows the DE distribution using OFC (offset = 20). Here the low abundance genes were no longer underlined as DE. Panel C shows the DE distribution following hierarchical DE analysis using OFC for A- and HT-specific genes emphasizing the effect of excluding potentially leaky genes. The red horizontal lines denote 0 log_2_ FC/OFC and the blue lines ± 0.5 log_2_ FC/OFC.

We observed direct evidence of the biasing effect of low abundance FC (Fig 3A). For example, using standard FC, numerous low abundance DE genes in the HT were in fact a signature from the A body part (e.g. sperm and semen genes are specific to the A body part, but detected as differentially expressed in the HT at lower abundance than in the A samples; Fig 3A and 3B). The RNA-seq technique is highly sensitive and detected these transcripts due to leak through, contamination with small amounts of non-target tissues during dissections, or movement of mRNAs. Without applying a correction, the list of DE genes is likely to contain numerous spurious (often low-abundance) entries. A practical solution was to use offset fold change (OFC), with an offset determined empirically from the data (as described above) instead of FC (Fig 3A vs 3B) and to apply the hierarchical DE (Fig 3C; S2 Methods). The introduction of the offset reduced the number of DE transcripts; however some variation in the medium-expression range was retained. The genes with higher variability between replicates, found in this region were mainly leaky genes (identified based on the location information from FlyAtlas [47]). A comparison of the MA plots for all genes (Fig 3B) versus the A- and the HT-specific genes highlighted the effect of the hierarchical differential expression (Fig 3C) i.e. a reduction in the replicate-to-replicate variability which ensured a more accurate DE call, especially when the treatment DE is subtle (S5 Fig).

### Comparisons with existing approaches

The final stage of analysis of the *D*. *melanogaster* data was to evaluate the output gained from our pipeline, with that obtained from the analysis of the same, original input data (consisting of all 3 replicates for each condition) using DESeq2 [12] and edgeR [44, 48]. The input data for edgeR and DEseq2 comprised the H and A samples and the results for the analysis of DE in the H samples is presented. It should be noted that we compared the results from edgeR or DEseq2 (all replicates, as the QC of neither method required any exclusions) with that from our pipeline, in which the exclusion of an outlier replicate was recommended (and performed).

### Effect of the normalization

**Fig 4:**
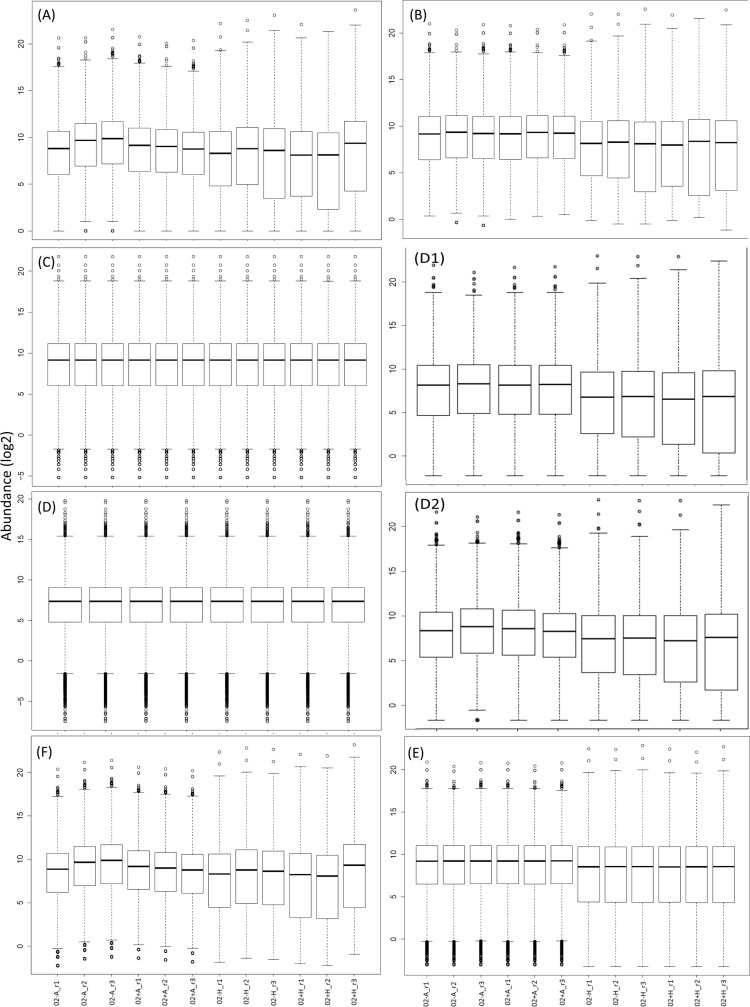
Comparison of expression distributions resulting from different normalization methods. Shown are standard boxplots of normalized gene expressions. On the x-axis are the different samples (e.g. 02A-1 = 2h time point abdomen body part, no rivals, replicate 1) and the on the y-axis the log_2_ gene expression. Panel A shows raw expression levels, B RPM normalization to a fixed total of 50M reads, C quantile normalization, D subsampling without replacement + quantile correction, E the DESeq2 normalization and F the edgeR normalization. D1 and D2 show normalization by subsampling (to 50M reads) with and without replacement normalization, respectively.

**Fig 5:**
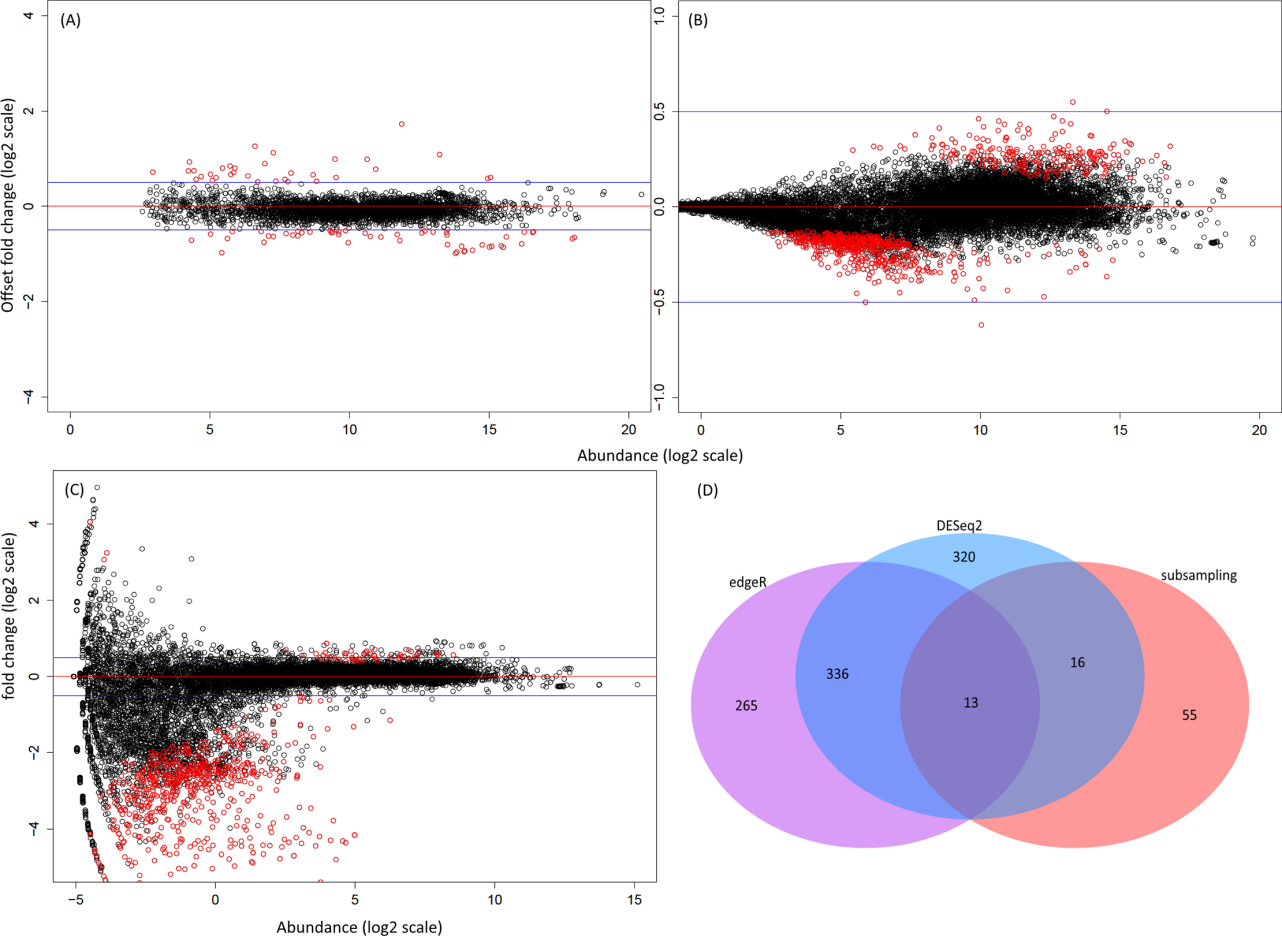
Comparison of distribution of DE obtained using different approaches. MA plots, with x-axis showing log_2_ average abundances against OFC with an offset of 20 (panel A) and FC (panels B and C). The example shown is the 02H ± rivals DE comparison. The red line indicates 0 log_2_ FC/OFC and the blue lines ±0.5 log_2_ FC/OFC. Red data points represent the genes ‘called’ differentially expressed by each of the methods. Panel A shows the results for subsampling normalization with DE calculated using the hierarchical approach, Panel B for DEseq2 and Panel C for edgeR. Panel D shows a Venn diagram identifying the number of differentially expressed genes identified by two or more methods versus uniquely by each.

An *a priori* (and necessary, but not sufficient) condition for reliable DE call is good comparability between the distributions of expression levels. We compared the distributions of expression levels (log_2_ scale) of the raw data versus RPM, quantile, subsampling with and without replacement, DEseq2 and edgeR normalizations (Fig 4). The boxplot of the raw abundances (Fig 4A) illustrated the variation among the replicates and samples and the need for normalization. The RPM normalization (Fig 4B) rendered the A and HT distributions comparable to some extent. However, the variability between samples was still present (especially for the HT samples). The quantile normalization (Fig 4C) resulted in comparable distributions, as did both the subsampling with and without replacement (Fig 4D1 and 4D2). DEseq2 performed well (Fig 4E)–although residual differences in the distributions of the A vs HT samples remained. EdgeR (Fig 4F) did not effectively equalize the distributions of abundances. We conclude that the subsampling, quantile and DESeq2 normalizations (Fig 4C, 4D1, 4D2 and 4E) were most effective at producing comparable distributions of normalized expression levels for this dataset. However, we note that inspection of the distributions of abundances (Fig 4) did not fully reveal which methods are most effective at reducing gene level replicate to replicate variation. This is instead best illustrated by MA plots (Fig 5 and S6 Fig), which indicated that it was the DESeq2 and the subsampling approaches that appeared to perform well in terms of minimizing both overall and per gene variation.

### Differences in the DE call between methods

To evaluate the effect of the normalization and hierarchical DE call we compared analyses of the *D*. *melanogaster* 2h HT and A samples ±rivals with the output of DEseq2 and edgeR (Fig 5). The subsampling without replacement normalization and hierarchical DE call (Fig 5A) showed a relatively low number of up/down-regulated genes with relevant biological functions. The equivalent analysis for DEseq2 (Fig 5B) called many more genes as DE that fell in the region of +/- 0.5 log_2_ FC (i.e. below the validatable threshold [49]). The analysis using edgeR (Fig 5C) showed a high frequency of low abundance DE and of leaky genes, which is likely to either represent noise or biological signal of an insufficient magnitude to be captured effectively in the low throughput validation. The degree of overlap between the three methods (Fig 5D) revealed a small number of core genes present in the intersection. edgeR and DEseq2 called many more genes as DE and the number of genes uniquely identified by edgeR and DEseq2 was also larger than the number identified in common between the two. These results show that the analysis pipeline chosen may have a strong effect on the biological interpretation of the DE analysis.

For the ±rivals comparison for the HT samples, out of the 575 genes that were specific to edgeR, 14 were HT genes (all with max abundance > 50) and 561 were A genes (327 with max abundance > 50 and 234 < 50; S7 Fig). Out of the 578 genes specific to DESeq2, 101 were HT genes (100 with abundance > 50) and 477 were A genes (271 with max abundance > 50 and 206 < 50; S7 Fig). The predominance of A genes in the DE call supported the use of the hierarchical DE approach. The presence of low abundance genes supported the use of an offset for the calculation of DE. Of some concern was that for genes with a reasonable abundance (> 50) the expression intervals for the ± rivals differences called by DEseq2 and edgeR were close/overlapping, which could make independent validation using low throughput methods challenging. Samples of the high number of DE genes called by both or either of DEseq2 and edgeR could be difficult to validate independently.

Reasons for the differences in DE call between different analysis methods could result from many sources. These potentially include poor comparability of normalized gene expression between replicates and an inadequate incorporation of differences in the magnitude of DE across the different experimental levels. In DEseq2, replicate-to-replicate variability is averaged and it is DE over and above this variation that is called. The accuracy of the calling procedure is increased if replicates have a low coefficient of variation (CV = standard deviation/mean). However, in the *D*. *melanogaster* data, the CV was often > 0.25 and for some was > 0.5 (S8 Fig). In the example shown (S8A–S8D Fig), there was clearly higher dispersion (CV) at low transcript abundance and consistently high CV across higher abundances. This variation was reduced here by the subsampling normalization and the use of the offset (S8E–S8H Fig, in which dispersion showed minimal variation across transcript abundance and the CV was consistently low (generally < 0.1)). DESeq2 [12] notes this effect of high replicate variation and proposes a shrinkage estimation for dispersions based on empirical Bayes and FC, to improve stability and interpretability of estimates. These changes do appear to minimize to some extent the overall issue of high variability in transcript abundance.

### Comparison of low throughput validated genes with DESeq2 and edgeR outputs

We next investigated whether the set of DE genes identified using the hierarchical approach from our *D*. *melanogaster* dataset, and validated by qRT-PCR [39], were present in the output of DEseq2 and edgeR. Reassuringly, based on DESeq2 our qRT-PCR reference genes were not called DE. Two other genes of interest from the A samples that were validated as DE, had a *P*-value < 0.05 by DESeq2 (although adjusted *P*-value > 0.05). One gene of interest validated from the HT had a *P*-value < 0.05 (but again not according to the adjusted *P*-value). For DESeq2 the log_2_(FC) values were small (0.15 and 0.16, respectively). Hence these genes were not likely to have been selected for further investigation. Based on the edgeR output, our reference genes were also determined as not significantly DE. For the validated A genes of interest (GOI), only one was called marginally DE by edgeR (p = 0.08, FBgn0259998, but with small log_2_(FC) = 0.24). For the HT, one GOI, with log_2_(FC) = 0.58, was called as significantly DE (the same gene as identified by DESeq2). Another GOI, FBgn0044812, was identified with log_2_(FC) = 0.82 yet with a p-value of 0.49 from edgeR and therefore would not have been selected (S5 Table).

Comparing the validated gene set with the output of edgeR and DESeq2, we conclude that some GOIs failed to be identified and therefore the corresponding biological functions (immunity, odorant perception) might have been overlooked.

### Which normalization to choose?

An RNA-seq sample is a snapshot of RNA fragments present at a given time, randomly selected according to the RNA abundances, to fill the sequencing space. Due to the stochastic nature of the sequencing process, even technical replicates, at different sequencing depths, do not exhibit a constant scaling factor for all abundances. Also, RNA-seq outputs have varying fits to standard distributions, making it difficult to define “the best” choice. Although the subsampling without replacement normalization was efficient in minimizing the effects of the variable sequencing depth, while preserving a high similarity with the original samples (Fig 3), we suggest that it is advisable to test different normalizations on mRNA-seq and choose the most appropriate method for the given dataset, on a case-by-case basis [28]

### Case study—Analysis of human mRNA-seq datasets using subsampling (without replacement) normalization

RNA-seq is expected to have good external validity and produce comparable results when the same RNA is used, across different laboratories. A recent study of mRNA-seq conducted on the same human samples (expression level variation in lymphblastoid cell lines) involved the use of the same samples sequenced in two different locations: Yale versus Argonne [46]. Some variation between the results from the different laboratories was observed and these data were further analysed to test whether edgeR could reduce the variability between replicates [48]. Here we tested whether our subsampling normalization could result in any additional variance reduction. We randomly selected 5 sets of samples (144, 153, 201, 209 and 210) with two replicates each, one from the Yale laboratory source and one from the Argonne source. For these runs, the length of the reads was 36nt for Yale and 46nt for the Argonne-derived data. Since the length of the sequencing read influences the number of unique fragments and the mapping to the reference transcriptome (and, as a result, the gene expression) we trimmed all reads to comparable lengths (35nt) and mapped the reads to the reference human genome using full length, no mismatch or gap criteria and using PatMaN [50]. The subsampling, without replacement, was conducted on 7M reads (the number of reads for the smallest sample was 7.1M, and for the largest sample was 8.7M). This normalization was followed by a correction using a quantile normalization applied on the matrix of gene expressions.

We created comparable plots to [48] for the data subjected to subsampling normalization (S9 Fig). MA plots for the two replicates of each sample showed high reproducibility between runs. The distribution of the coefficient of variation (CV) versus the abundance for the 5 selected sets of samples with two replicates each (one for Yale and one for Argonne) (S9 Fig) showed that the CV for all 5 pairs of samples was consistently (< 0.1) lower than for the analysis of [48]) indicating a very high similarity between the runs. The MA plots on the same sets of two samples showed a high reproducibility between replicates (no genes showing |log_2_(OFC)| > 1). The genes showing DE were mainly localized in the 2^4^ (16)– 2^6^ (64) range, which is borderline for validation/noise. Together, these analyses showed that: (i) the CV obtained when the subsampling (without replacement) normalization was employed was lower than the CV reported in [48], suggesting that our normalization was tighter, (ii) there was very little DE between replicates, indicating good reproducibility between the sequencing runs.

Overall, we conclude that the subsampling, without replacement approach minimized the technical differences between the two runs in the different laboratories and this approach rendered the samples comparable, potentially improving the biological inference.

## Conclusion

The main findings from this study highlight that both qualitative and quantitative QC can be informative and that subsampling (without replacement) -based normalization and hierarchical structuring of the DE call, is efficient in managing variation in read number and differences in sample complexities. In comparison to existing approaches, our adapted methods performed well and identified valid candidates that were confirmed using low throughput approaches [39]. We also successfully applied the subsampling (without replacement) normalization to existing mRNA-seq datasets, and in doing so reduced inter-laboratory variation [46]. The adapted approaches proved to be efficient in comparison with existing methods at minimizing potentially confounding sources of variation. Evaluation of accurate gene expression levels is essential for all mRNA profiles but is also key to successful correlation analysis between mRNAs and sRNAs [51, 52]. Overall, the study shows the value of tailoring bioinformatics analyses and checking multiple approaches to leverage maximum power and accuracy from the analysis of a RNA-seq dataset.

## Materials and methods

### Quality check (QC)

For the mRNA-seq samples, the QC consisted of two stages. Stage 1 comprised of previously described methods [4] including: (i) the analysis of FastQ quality scores [17], (ii) the total number of reads (sequencing depth) and the read duplication rate, defined as complexity [27], (iii) nucleotide composition relative to the genome and transcriptome of *D*. *melanogaster*, used to highlight biases such as PCR and ligation bias [53], (iv) strand bias quantified on CDS incident reads as |*P* − 0.5| + |*N* − 0.5|, where P and N were the proportion of positive and negative strand read matches, respectively [27] and (v) proportions of reads matching the different genome annotation classes (e.g. mRNAs, t/rRNAs, miRNAs, UTRs, introns, intergenic regions [4], with matching done using PatMaN, [50] on full length reads with no mismatches or gaps allowed). Stage 2 comprised of quantitative approaches, some applied/designed on mRNA-seq data for the first time, which provided an increased insight into sample comparability and enabled us to evaluate the effectiveness of the normalization. The expression level of a gene/ transcript was calculated as the algebraic sum of the raw/normalized abundances of the incident reads [31]. We examined: (i) sample similarity calculated using the Jaccard similarity index [29] on the top 1000 most abundant genes, and intersection analyses). These measures were calculated as the ratio between number of genes found in common to the number of unique genes present in either sample, (ii) complexities (calculated at gene level and presented as Bland-Altman (MA) plots) and (iii) point-to-point PCC between gene expression profiles in different replicates/ samples. The latter were computed on the vector of expression defined for each gene. For all positions *i* on a gene we computed y[i] which is the sum of abundances of fragments incident with position *i*. The point-to-point PCC was computed as the standard PCC on the corresponding vectors from the two samples which were compared.

### Normalization

We adapted a normalization procedure based on subsampling (without replacement) [40]. The consistency of subsamples was validated using bootstrapping. The subsampling without replacement was done on the redundant set of reads (before genome matching, with the ncRNAs incident reads removed). The proportion of genome matching reads and the variation in gene complexities (coupled with the p2pPCC between the subsamples and the original sample) were used as criteria for consistency of the subsamples. Each sample was first subjected to incremental subsampling in order to investigate the effect on the data structure (complexities, both for non-matching and genome-matching reads) of sampling 95% through to 45% of the data, with successive decreasing steps of 5%. A sample was deemed satisfactory if the proportion of redundant genome matching reads remained constant and the average point-to-point PCC were above 95% as the number of redundant reads was decreased from 95% to 45%. This step represented an empirical determination of the level of subsampling that could be done whilst preserving the original data structure. The second step of the normalization was the subsampling to a fixed total (the minimum sequencing depth of the accepted samples). Samples with low sequencing depths, which would lead to a heavy subsampling for the samples with high read numbers (less than 55%, empirically determined), were treated on a case-by-case basis. A quantile normalization [54] may be employed after this step, as in our analysis of the *D*. *melanogaster* data, to render the distributions fully comparable. For the analysis of the *D melanogaster* and *H*. *sapiens* datasets the quantile correction was employed. If the distributions of abundances, after the subsampling, are already perfectly aligned, then the quantile correction would not be necessary. The pseudocode is presented in S1 Methods.

Existing procedures which were used for the comparison of the new normalization methods were: scaling normalization [31], for which the scaling total was the mean of the sequencing depths of the compared samples, quantile normalization [54] and the normalization approaches from edgeR [48] and DESeq2 [12]. All were employed using the recommended standard parameters.

### Differential expression call

Existing methods for the DE call are often based on comparing the variability between replicates with the difference between the treatments. However, coefficient of variance (or CV) based on a small number of points may often not reflect the true variance of the given gene/transcript [55–57]. Moreover, when small numbers of measurements are available, a more conservative approach, which we used here, is to approximate that replicate measurements will fall within the limits of the maximal confidence interval (CI) [58]. These intervals are defined on the minimum and maximum normalized expression levels for the replicated measurements. The amplitude of the DE can then be calculated at varying levels of stringency, depending upon the distance threshold selected between the proximal ends of the maximal CIs [28, 59]. All genes called DE using these stringent rules would also called DE under statistical tests. Here, we used a threshold on the amplitude of the DE of 1.5 fold change, in line with empirically determined high [39] and low throughput method detection thresholds [49]. This prevented the selection of genes whose expression ranges did not differ markedly and also ensured a higher chance of low throughput validation.

DE was calculated using a hierarchical approach and by applying an offset fold change (OFC) method (with offset = 20, empirically determined, using the point-to-point PCC, for all replicates within all samples). There were 3 steps to the hierarchical analysis used for the analysis of the *D*. *melanogaster* transcriptome data. (i) Identification of levels for the hierarchical differential expression and the constituent internal classes. For the *D*. *melanogaster* data one ‘level’ was body part (with HT and A as internal classes) and the other was treatment (with presence or absence of rivals as classes). (ii) The ordering of the hierarchical levels based on the amplitude of differential expression. This was quantified by the width/ spread of the distribution of DE in terms of mean/ median, IQR and min/max values. The amplitude of DE in descending order provided the correct ordering of the levels for the hierarchical DE. (iii) The DE analysis on the proximal ends of the CIs, using OFC [27]. The pseudocode is presented in S2 Methods.

The two-step DE procedure (using OFC) consisted of (i) calculation of the list of genes showing DE between body parts, followed by (ii) calculation of the DE between genes in the ± rivals treatment comparisons. Step (i) was conducted on the summed expression levels in the ± rivals pairs (i.e. the ± HT samples combined, and the ± A samples combined, for all time points). The genes were then separated into genes expressed only in HT, only in A, and in both the HT and A. Step (ii) of the DE was then applied on the resulting 3 categories (HT; A; HT+A) using the ± rival condition. The DE call was conducted on the +/- rivals condition, using maximal CIs created on the normalized replicates. As outlined above, we called DE genes with more than 1.5 fold between the proximal ends of the maximal CIs. The DE call as determined by edgeR and DESeq2 were calculated using the default functions and parameters.

### Data access

*mRNA samples*: (a) *D melanogaster*: males of *D melanogaster* exposed to conspecific rivals (or not) for 3 time periods (GSE55930). (b) *H sapiens*: For the mRNA Human samples, we chose 5 samples from the Pickrell et al. 2010 [46] study (GSE19480) in order to compare gene expression variation in RNA sequencing between the Argonne and the Yale laboratory sequencing runs. The selected samples were: GSM485369 (NA19144_yale), GSM485380 (NA19144_argonne); GSM485368 (NA19153_yale), GSM485383 (NA19153_argonne); GSM485367 (NA19201_yale), GSM485381 (NA19201_argonne); GSM485365 (NA19209_yale), GSM485388 (NA19209_argonne); GSM485364 (NA19210_yale), GSM485382 (NA19210_argonne). These samples were derived from lymphoblastoid cell lines (LCLs) derived from unrelated individuals from Nigeria (extensively genotyped by the International HapMap Project). The sequencing was done on Illumina GAII, with sequencing reads of 36nt, for the Yale sequencing samples and 46nt for the Argonne sequencing.

## Supporting information

S1 Methods Subsampling normalization–pseudocode. A description with details for (1) Incremental subsampling and bootstrapping check for consistency of a sample, and (2) Subsampling to a fixed total.
(PDF)

S2 Methods Two step (hierarchical) differential expression (HDE)—Pseudocode. A description with technical details for the two step (hierarchical) DE, including the identification of levels in the hierarchy.
(PDF)

S1 Table Overview of the *D. melanogaster* samples used for the comparative analysis of different approaches for the identification of differentially expressed genes. From the study described in Mohorianu et al. 2017, RNA 23:1048–1059 [39], we selected four samples (each with 3 biological replicates, 1–3) comprising of 2 head-thorax (H) samples and 2 abdomen (A) samples of flies exposed to rivals (+) or kept alone (-) for 02h. For each sequencing library, before and after the matching to known annotations, we present the redundant/total number of reads (R), the non-redundant/unique number of reads (NR) and the overall complexity (C), defined as ratio of NR to R reads. The sequencing reads were matched to the genome, annotated exons, introns, 5’ UTRs, 3’ UTRs, ncRNAs and intergenic regions. For all annotation alignments we also present the proportions of R and NR reads. (%R and %NR, respectively). For these samples, we observed a high variability in sequencing depth, from 38M reads (sample 02+A3) to 129.7M reads (02+H3), and, as a consequence, a high variability in the resulting complexities, yet little variation in the %R for each annotation, suggesting a high quality of the sequencing output.
(XLSX)

S2 Table Example of intersection analysis for the 02-A, 02+A, 02-H and 02+H samples in the *D. melanogaster* dataset. Replicate 1 samples 02-A, 02+A, 02-H and 02+H (sample codes: 02h of exposure, ± rivals, HT or A tissue) were used to illustrate the proportion of reads mapping simultaneously to pairwise groups of CDSs, exons, 5’ and 3’ UTRs, introns and intergenic regions; on the diagonal, the proportion is 1.00 since each category is compared to itself; the matrix is not symmetric because each proportion is calculated relative to the number of reads present in the category indicated by the column. We observe that a high proportion of reads is incident with protein coding genes, with few reads showing multiple matching to protein coding-genes and introns or intergenic regions; overall, these results suggest a high quality of the data. In the main study we computed the expression levels using gene mapping reads.
(XLSX)

S3 Table Jaccard similarity indices computed on the top 1000 most abundant genes in each sample (out of a total of 15 513 genes expressed in at least one sample). Shown is a 12 by 12 matrix of all the original samples compared with each other. Samples are labelled by time point (2h), by ± rivals treatment, by body part (A or HT) and then by replicate number. Each sample tested against itself along the diagonal is therefore 100% similar and shares the top 1000 most abundant genes in common. A to A comparisons are shaded in purple, HT to HT comparisons in peach. Samples drawn from the same body parts shared > 90% similarity, and between body parts the similarity dropped to ~50%. Similarity between the ± rivals treatments tended to be higher than between replicates. Two illustrative examples are highlighted, in which ± rivals indices (in red bold) were generally higher than replicate to replicate similarity (blue bold). This highlighted the need for adapted normalization methods.
(XLSX)

S4 Table Example of incremental check for consistency done using subsampling without replacement for sample 02-H2 in the *D. melanogaster* dataset. For sample 02-H2 (sample code: 02h, no exposure to rivals, HT body part, replicate 2) we present the incremental subsampling, without replacement, from 99% to 40% of the data. To judge whether a sample is consistent, and to determine the consistency threshold, we used the proportion of redundant reads matching to the reference genome (*D*. *melanogaster*, v 6.11). As a consequence of the incremental subsampling, the complexity, defined as the ratio of non-redundant to redundant reads, increased, and became comparable to the complexity of the other replicates, when subsampled at the same sequencing depth. A replicate was accepted if it exhibited a similar complexity (and distribution of per-gene complexities) with the other replicates for the same type of sample.
(XLSX)

S5 Table **Results from (A) DEseq2 and (B) edgeR analyses of the *Drosophila melanogaster* qRT-PCR 'validated' gene set from Mohorianu et al. 2017 (RNA 23:1048–1059).** For the validations we used 3 reference genes and validated 15 A genes and 6 HT genes based on the DE selection using subsampling normalization and hierarchical DE. We investigated whether these genes were called DE by either (A) DESeq2 or (B) edgeR. In S5A Table we present the results for DESeq2, in S5B Table the results for edgeR. For each of the three categories of genes (reference genes, A genes and HT genes) we show the average of normalized abundances (baseMean for DESeq2 and logCPM for edgeR), the fold change between treatments (log_2_ FoldChange for DESeq2 and log_2_FC for edgeR) and the DE *P*-value and adjusted *P*-value (used for the DE call).
(XLSX)

S1 Fig Analysis framework for the *D. melanogaster* mRNA-seq data. Required inputs (sequencing data in FASTQ format, the corresponding reference genome and transcriptome in FASTA/GFF) and the six main steps of the analysis are shown in a workflow diagram, following Conesa et al. 2016 (Genome Biology, 17:13). The steps, for which additional details are included, are: Quality check (QC), alignment, normalization of gene abundances, identification of DE, functional enrichment and finally low-throughput validation.
(PDF)

S2 Fig **Correlation analyses (Pearson (PCC), Spearman (SCC) and Kendall correlation coefficients (KCC))** between the gene expression levels for the *D*. *melanogaster* data for (A) all samples, (B) HT samples, (C) A samples. A1, B1, C1 show the PCC; A2, B2, C2 show the SCC; A3, B3 and C3 show the KCC. Each panel shows the distributions of correlation coefficients for all pairwise comparisons. For example, in panel A.1, sample 1 on the x-axis shows the distribution of the n = 35 correlation coefficients calculated between the gene expressions in sample 1 compared with gene expressions in all other 35 samples, using the PCC. The results are presented as a standard boxplots i.e. the box indicates the inter-quartile range, the middle line is the median and the whiskers extend to 5% and 95%; the outliers are represented with circles. All three approaches supported the same conclusion i.e. the A and H gene expression levels in the A and H samples, respectively, correlated very well (minimum correlation between any two samples was >0.97, B and C panels B), whereas, if we compared between A and H samples (A panels), the minimum correlation dropped to 0.5.
(PDF)

S3 Fig Distribution of point-to-point Pearson correlation coefficient (PCC) (y-axis) between gene expression profiles against gene expression levels (x-axis, log_2_ scale) for pairwise comparisons for the *D. melanogaster* data for the 3 replicates of the 02-H sample as an example (2h, HT body part, no rivals). Panel A shows replicate 1 vs 2, B replicate 1 vs 3, and C replicate 2 vs 3. Shown are the raw data, prior to normalization. For all replicate comparisons, more variability was consistently observed at lower abundances and derived mostly from the small number or scattered incident reads. For the higher abundance genes, the p2pPCC was tight, indicating a high reproducibility of the expression profile and a low incidence of alternative splicing events. This analysis formed the basis of the offset identification i.e. the offset was selected to be the value for which, for all replicates, for all samples, the median of the p2pPCC was above 0.5.
(PDF)

S4 Fig Point-to-point Pearson correlation coefficient (PCC) between the raw and subsampled data for the 02+H3 sample of the *D. melanogaster* data (sample code: 02h, + rivals. HT body part replicate 3). To indicate the consistency during the subsampling, without replacement, the plots show the point-to-point PCC between the original and incrementally subsampled (from 40% to 95%) data (Panels A to L; A: 40%, B: 45%, C: 50%, D: 55%, E: 60%, F: 65%, G: 70%, H: 75% I: 80%, J: 85%, K: 90%, L: 95%). On the x-axis is the gene abundance (log_2_) and on the y-axis the distribution of point-to-point PCCs calculated for each expressed gene.
(PDF)

S5 Fig Identification of the hierarchy levels for the hierarchical differential expression (HDE) analysis based on the distribution of DE for the different classes of samples, i.e. replicates, body parts and ± rivals treatments (for the *D. melanogaster* data). Frequency density plots were used to show the distribution of DE between samples (offset fold change, log_2_ scale). Panel A shows the replicate-replicate DE (blue) and the with/without rivals DE (red) for the abdomen (A) samples. Panel B shows the corresponding data for the HT (H) body part. Panel C shows the distribution of DE for the with/without rivals treatments (blue for HT and green for A samples) and the DE between H and A (orange). The DE distribution for the treatment (+/- rivals, red) was overlapping with the DE distribution between the replicates (blue), indicating a subtle DE signature. However, the DE distribution between body parts (orange) showed a good separation between HT- and AB- specific genes. These DE distributions supported the choice of a hierarchical design for the identification of DE genes between the treatments.
(PDF)

S6 Fig Replicate-to-replicate MA plot on the 02-A samples for checking the efficiency of the A RPM, B quantile, C DESeq2 and D edgeR normalization methods. (Sample code: 02h, no rivals, A body part). On the x-axis we represent the average abundance between replicates (log_2_ scale), on the y-axis the FC (log_2_ scale). Although the quantile and the DESeq2 produced very similar distributions of abundances between replicates/samples, the former did not produce a tight MA plot when variability in expression at gene level was assessed. For the comparable results for the subsampling (without replacement) approach, see main Fig 3C.
(PDF)

S7 Fig Distribution of abundances for the *D. melanogaster* data (for the ± rivals treatment DE) for the full set of genes identified as DE exclusively by each method. EdgeR only genes shown in S7A Fig, DEseq2 only in S7B Fig and subsampling normalization (without replacement) only in S7C Fig Genes are denoted by their FBgn identifiers. For each gene identified as DE exclusively by each method, the normalized abundance is given for each of the 2h HT (H) and A ± rivals samples. The leaky genes visible in the DE calls of edgeR and DESeq2 are not highlighted as DE using our adapted DE approach (i.e. hierarchical design and use of offset).
(PDF)

S8 Fig Comparison of the coefficients of variation across abundance (*D. melanogaster* data). On the x-axis is the abundance in log_2_ scale, on the y-axis the coefficient of variation (CV)—the ratio between the standard deviation and the mean. For clarity, the distributions are represented as standard boxplots. The upper panels (A,B,C,D) show the CV for the original data for A samples without rivals (A), A samples with rivals (B), HT samples without rivals (C) and HT samples with rivals (D), respectively. The lower panels (E,F,G,H) give the CV for the same samples, after the subsampling normalization (without replacement). The red horizontal lines indicate 0.5 and 0,25 CV. It is clear that the subsampling normalization reduced the variance between the replicates to < 0.25 CV across most abundances (panels E-H), whereas the CV was much higher across all abundances for the raw data (panels A-D).
(PDF)

S9 Fig Analysis of the effect of the subsampling normalization on technical (laboratory-laboratory) variation in mRNA-seq for human mRNA-seq data (Pickrell et al. 2010, Nature, 464:768–772). In the upper plots we show the coefficient of variation (CV), y-axis vs the average abundance, x-axis, obtained after the subsampling normalization (without replacement) for 5 sequencing pairs (each pair consisted of a Yale laboratory run compared to an Argonne run: A1,A2 = Sample 144; B1,B2 = Sample 153; C1,C2 = Sample 201; D1,D2 = Sample 209; E1,E2 = Sample 210). The F1 and F2 plots show the CV for the combined pairs for the Yale and Argonne replicates, respectively. For individual comparisons we achieved lower CVs in comparison to Zhou et al. 2014 (Nucleic Acids Res, 42:e91) analysis of these sample data. For the sets of lab replicates, Yale and Argonne, respectively, the results using subsampling without replacement are in line with the edgeR results (in red we represent the CV of these data obtained using edgeR, in blue the CV using DESeq2). Based on these distributions we concluded that the samples from the different laboratories could be rendered comparable using our subsampling approach (because it removed technical differences between the two different laboratory runs). In the lower panels, we present the MA plots, after the subsampling normalization, for the same pairs of samples. The tightness of these plots (all falling within ±0.5 OFC) supported the conclusion that the subsampling made these samples derived from sequencing in different laboratories highly comparable.
(PDF)
