# Supplementary material for: Comparison of alternative approaches for analysing multi-level RNA-seq data
Source: PLoS One. 2017 Aug 8;12(8):e0182694. doi: 10.1371/journal.pone.0182694 (PMC5549751; doi:10.1371/journal.pone.0182694)
Supplement: S1 Methods — A description with details for (1) Incremental subsampling and bootstrapping check for consistency of a sample, and (2) Subsampling to a fixed total. (PDF) [file pone.0182694.s001.pdf]

Comparison of alternative approaches for analysing multi-level RNA-seq data

Irina Mohorianu[1,2], Amanda Bretman[1,3], Damian T. Smith[1], Emily K. Fowler[1],

Tamas Dalmay[1], Tracey Chapman[1]*

[1]School of Biological Sciences, University of East Anglia, Norwich Research Park, Norwich, NR4 7TJ, United Kingdom.

[2]School of Computing Sciences, University of East Anglia, Norwich Research Park, Norwich, NR4 7TJ, United Kingdom.

[3]School of Biology, University of Leeds, Leeds, LS2 9JT, United Kingdom.

* Corresponding author

E-mail: tracey.chapman@uea.ac.uk (TC)

S1 Methods – Subsampling (without replacement) normalization – pseudocode

The full details for (1) **Incremental subsampling without replacement and boostrapping, for checking the consistency of a sample, and (2) Subsampling to a fixed total** are presented as pseudocode below:

STEP 1. Incremental subsampling and bootstrapping, for checking the consistency of a sample

```
foreach proportion p from 100% to 45%, step -5%
{
	Sampling - the procedure was repeated 50 times
	foreach repeat r from 1 to 50
	{
		sample without replacement p% from the data
		compute gene expression levels
		(the 0 mis-match, full length approach is assumed)

		the distribution of gene expression levels is compared with the
		original distribution (differential expression between gene
		expressions). The confidence intervals are built on the DE
		distribution
		compare the proportion of genome matching reads (Redundant form)

	}

	Bootstrapping
	foreach gene g
	{
		compute the coefficient of variation
		(which is negatively correlated with the abundance)
		compute the bootstrap estimates (mean + CI)

		test if the original mean is centred on the resulting distribution
		i.e. is the distribution representative for the original information
	}

	Check
	foreach gene g
	{
		compute the point to point Pearson correlation
	}
	foreach window of abundances (W=100)
	{
		compute the window Spearman correlation
	}
}
```

To evaluate the sources of changes in expression across transcripts, under different sampling proportions, the point to point Pearson correlation was computed on the vector of expression defined on each nucleotide for each gene. For all positions *i* on a gene we computed y[i] which is the sum of abundances of fragments incident with position *i*. The point to point Pearson was

computed as the standard Pearson correlation on the vectors from the original file and on the bootstrapped file.

The window Spearman correlation was calculated on windows of abundance (for simplicity, the window of abundance was set to W=100). The accuracy of the subsampling normalization was assessed by examining whether the ranking of DE was preserved following resampling (using the correlation analyses). This was important as the bootstrapping procedure should not itself introduce new differentially expressed (DE) genes (false positives) or omit any existing DE genes (false negatives).

STEP 2. Subsampling to a fixed total

```
Pseudocode

If the sample was consistent (as defined above), sampling was conducted without
replacement at a fixed total (for this project, the fixed total was set at 50M
reads per sample).
```

The resulting distributions of gene expression levels following the sampling to a fixed total of reads are shown in Figure S5B.

To further address the variability in the data, the annotation analysis was conducted, to determine the nature of the remaining outliers. We observed a high proportion of rRNA matching reads, with high variability. These reads were removed and a quantile normalization (Bolstad et al. 2003) was then employed to render the distributions fully comparable.

Reference

Bolstad BM, Irizarry RA, Astrand M, Speed TP. 2003. A comparison of normalization methods for high density oligonucleotide array data based on variance and bias. *Bioinformatics* 19:185-193.
