# Supplementary material for: Comparison of alternative approaches for analysing multi-level RNA-seq data
Source: PLoS One. 2017 Aug 8;12(8):e0182694. doi: 10.1371/journal.pone.0182694 (PMC5549751; doi:10.1371/journal.pone.0182694)
Supplement: S2 Methods — A description with technical details for the two step (hierarchical) DE, including the identification of levels in the hierarchy. (PDF) [file pone.0182694.s002.pdf]

Comparison of alternative approaches for analysing multi-level RNA-seq data

Irina Mohorianu[1,2], Amanda Bretman[1,3], Damian T. Smith[1], Emily K. Fowler[1],

Tamas Dalmay[1], Tracey Chapman[1]*

[1]School of Biological Sciences, University of East Anglia, Norwich Research Park, Norwich, NR4 7TJ, United Kingdom.

[2]School of Computing Sciences, University of East Anglia, Norwich Research Park, Norwich, NR4 7TJ, United Kingdom.

[3]School of Biology, University of Leeds, Leeds, LS2 9JT, United Kingdom.

* Corresponding author

E-mail: tracey.chapman@uea.ac.uk (TC)

S2 Methods - Two step (hierarchical) differential expression (HDE)

The `pseudocode` and technical details for the two step (hierarchical) DE are outlined below:

Step1. Identification of the conditions which may result in levels in a hierarchy (in this example, body part – HT and A and treatment – presence/ absence of rivals)

Step2. Identification of amplitude of differential expression for the levels in the data (description of the DE distribution, in terms of mean/ median, IQR, min, max)

```
foreach level
{
       foreach component of the level
       {
              sum the expression levels that are categorized in the selected level
       }
       the differential expression is computed between the components of the
       selected level e.g. between H and A samples or between + and – rivals
       samples, the distribution of DE is fully characterized (mean/ median, IQR,
       min, max)
}
The order of the levels in the hierarchy is the decreasing order of DE amplitude
```

The results shown above indicate more variation between body parts that between treatments, therefore the HT/A level was higher in the analysis hierarchy than was the +/- rivals level.

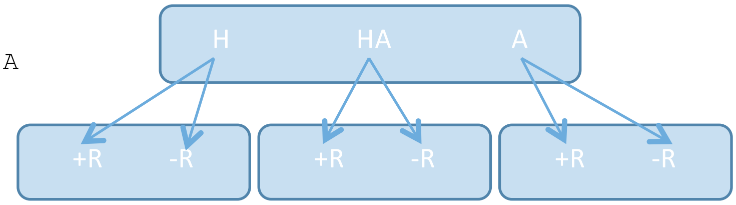

Fig. 1. Levels of the hierarchical differential expression. The first level is the H/A the second level is +/- rivals. The threshold for the H/A level was 2 OFC. The HA genes were the genes that had comparable expression levels in both H and A samples. The threshold for +/- rivals was set at 1.5 OFC.

Step3. The differential expression analysis (using OFC) was conducted according to the levels in the hierarchy i.e. first we separated the genes into H only, A only and HA genes, next for the body part specific genes we investigate the DE between the +/- rivals treatment.

The differential expression between treatments was conducted pairwise on each replicate.
