# Supplementary material for: Comparison of alternative approaches for analysing multi-level RNA-seq data
Source: PLoS One. 2017 Aug 8;12(8):e0182694. doi: 10.1371/journal.pone.0182694 (PMC5549751; doi:10.1371/journal.pone.0182694)
Supplement: S1 Fig — Required inputs (sequencing data in FASTQ format, the corresponding reference genome and transcriptome in FASTA/GFF) and the six main steps of the analysis are shown in a workflow diagram, following Conesa et al. 2016 (Genome Biology, 17:13). The steps, for which additional details are included, are: Quality check (QC), alignment, normalization of gene abundances, identification of DE, functional enrichment and finally low-throughput validation. (PDF) [file pone.0182694.s008.pdf]

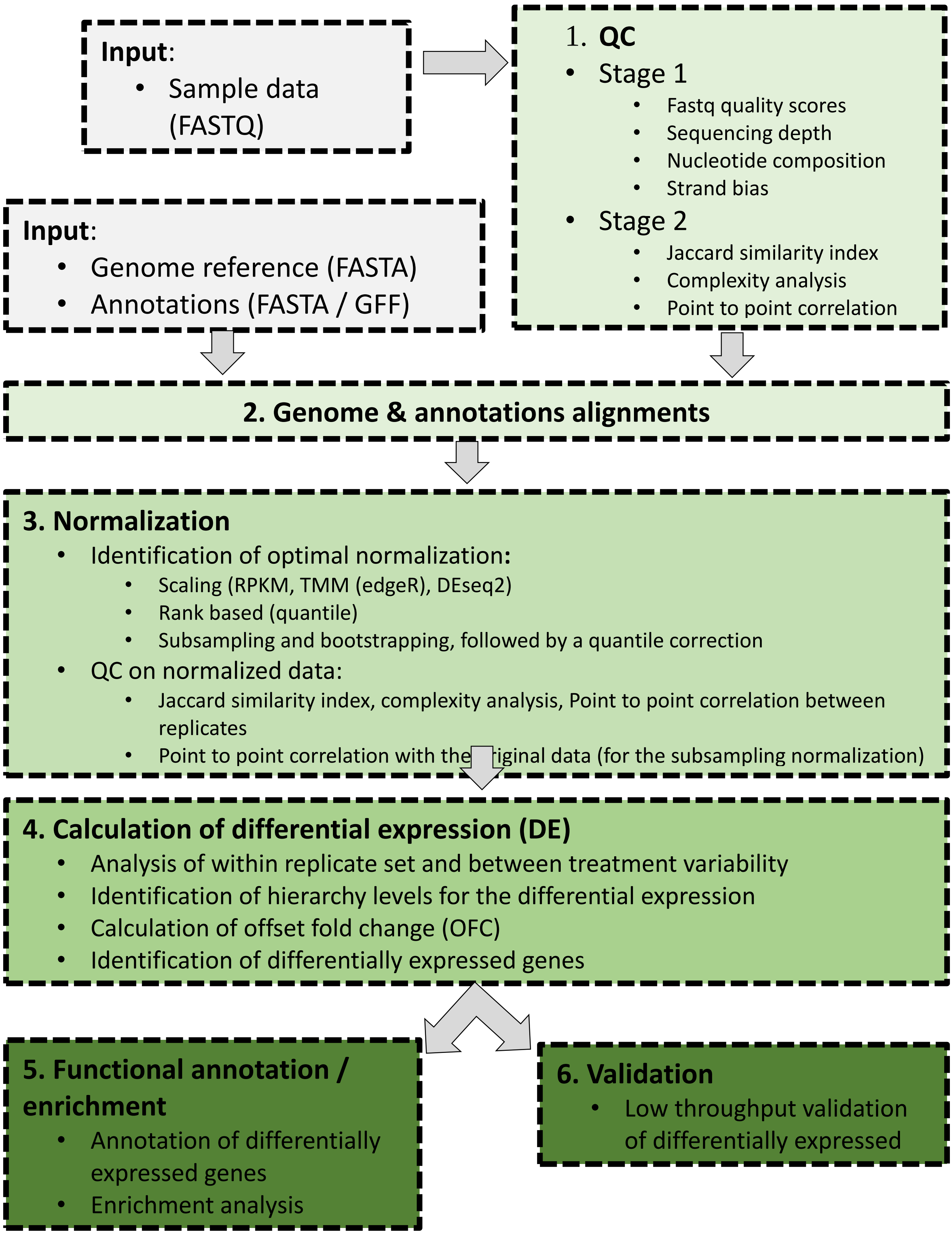

S1 Fig. Analysis framework for the *D. melanogaster* mRNA-seq data.
Required inputs (sequencing data in FASTQ format, and the corresponding reference genome and transcriptome in FASTA/GFF) and the six main steps of the analysis are shown in a workflow diagram, following Conesa et al. 2016 (Genome Biology, 17:13). The steps, for which additional details are included, are: Quality check (QC), alignment, normalization of gene abundances, identification of DE, functional enrichment and finally low-throughput validation
