# Supplementary material for: Comparison of alternative approaches for analysing multi-level RNA-seq data
Source: PLoS One. 2017 Aug 8;12(8):e0182694. doi: 10.1371/journal.pone.0182694 (PMC5549751; doi:10.1371/journal.pone.0182694)
Supplement: S2 Fig — Correlation analyses (Pearson (PCC), Spearman (SCC) and Kendall correlation coefficients (KCC)) between the gene expression levels for the D. melanogaster data for (A) all samples, (B) HT samples, (C) A samples. A1, B1, C1 show the PCC; A2, B2, C2 show the SCC; A3, B3 and C3 show the KCC. Each panel shows the distributions of correlation coefficients for all pairwise comparisons. For example, in panel A.1, sample 1 on the x-axis shows the distribution of the n = 35 correlation coefficients calculated between the gene expressions in sample 1 compared with gene expressions in all other 35 samples, using the PCC. The results are presented as a standard boxplots i.e. the box indicates the inter-quartile range, the middle line is the median and the whiskers extend to 5% and 95%; the outliers are represented with circles. All three approaches supported the same conclusion i.e. the A and H gene expression levels in the A and H samples, respectively, correlated very well (minimum correlation between any two samples was >0.97, B and C panels B), whereas, if we compared between A and H samples (A panels), the minimum correlation dropped to 0.5. (PDF) [file pone.0182694.s009.pdf]

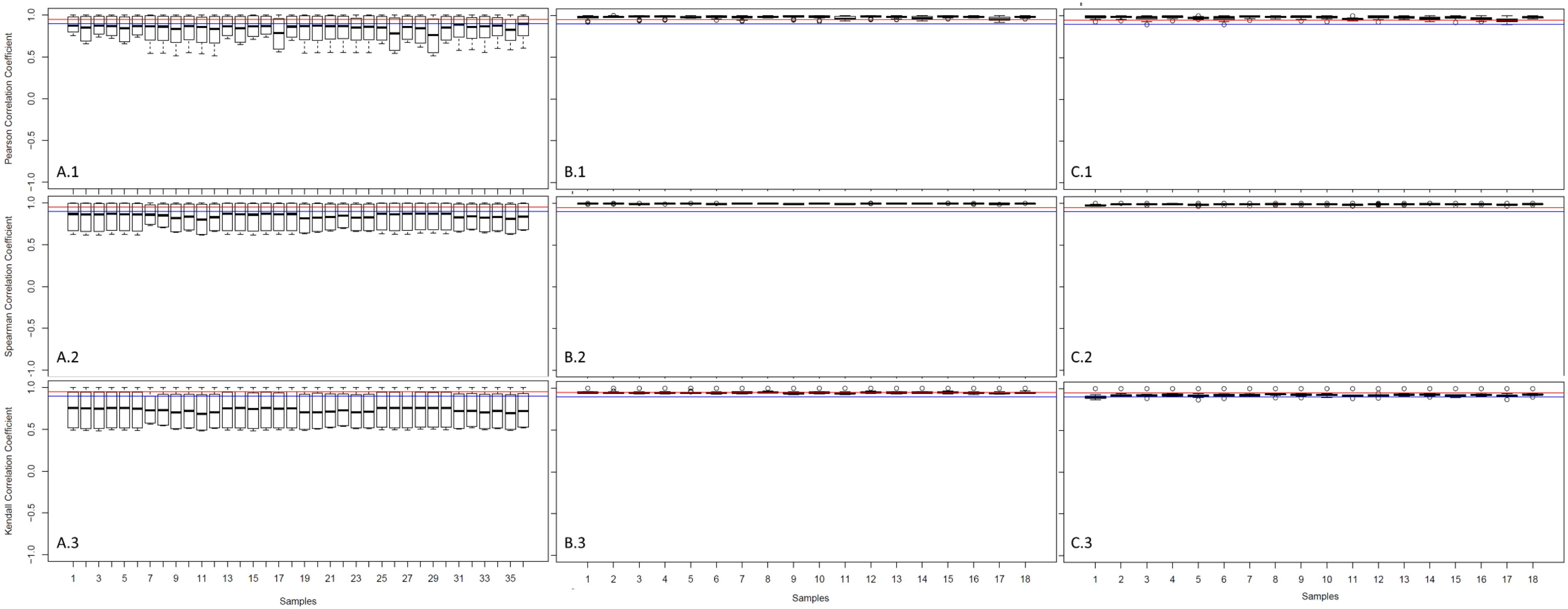

S2 Fig. **Correlation analyses (Pearson (PCC), Spearman (SCC) and Kendall correlation coefficients (KCC))** between the gene expression levels for the *D. melanogaster* data for (A) all samples, (B) HT samples, (C) A samples. A1, B1, C1 show the PCC; A2, B2, C2 show the SCC; A3, B3 and C3 show the KCC. Each panel shows the distributions of correlation coefficients for all pairwise comparisons. For example, in panel A.1, sample 1 on the x-axis shows the distribution of the n=35 correlation coefficients calculated between the gene expressions in sample 1 compared with gene expressions in all other 35 samples, using the PCC. The results are presented as a standard boxplots i.e. the box indicates the inter-quartile range, the middle line is the median and the whiskers extend to 5% and 95%; the outliers are represented with circles. All three approaches supported the same conclusion i.e. the A and HT gene expression levels in the A and HT samples, respectively, correlated very well (minimum correlation between any two samples was >0.97, B and C panels), whereas, if we compared between A and H samples (A panels), the minimum correlation dropped to 0.5.
