# Supplementary material for: Comparison of alternative approaches for analysing multi-level RNA-seq data
Source: PLoS One. 2017 Aug 8;12(8):e0182694. doi: 10.1371/journal.pone.0182694 (PMC5549751; doi:10.1371/journal.pone.0182694)
Supplement: S3 Fig — Panel A shows replicate 1 vs 2, B replicate 1 vs 3, and C replicate 2 vs 3. Shown are the raw data, prior to normalization. For all replicate comparisons, more variability was consistently observed at lower abundances and derived mostly from the small number or scattered incident reads. For the higher abundance genes, the p2pPCC was tight, indicating a high reproducibility of the expression profile and a low incidence of alternative splicing events. This analysis formed the basis of the offset identification i.e. the offset was selected to be the value for which, for all replicates, for all samples, the median of the p2pPCC was above 0.5. (PDF) [file pone.0182694.s010.pdf]

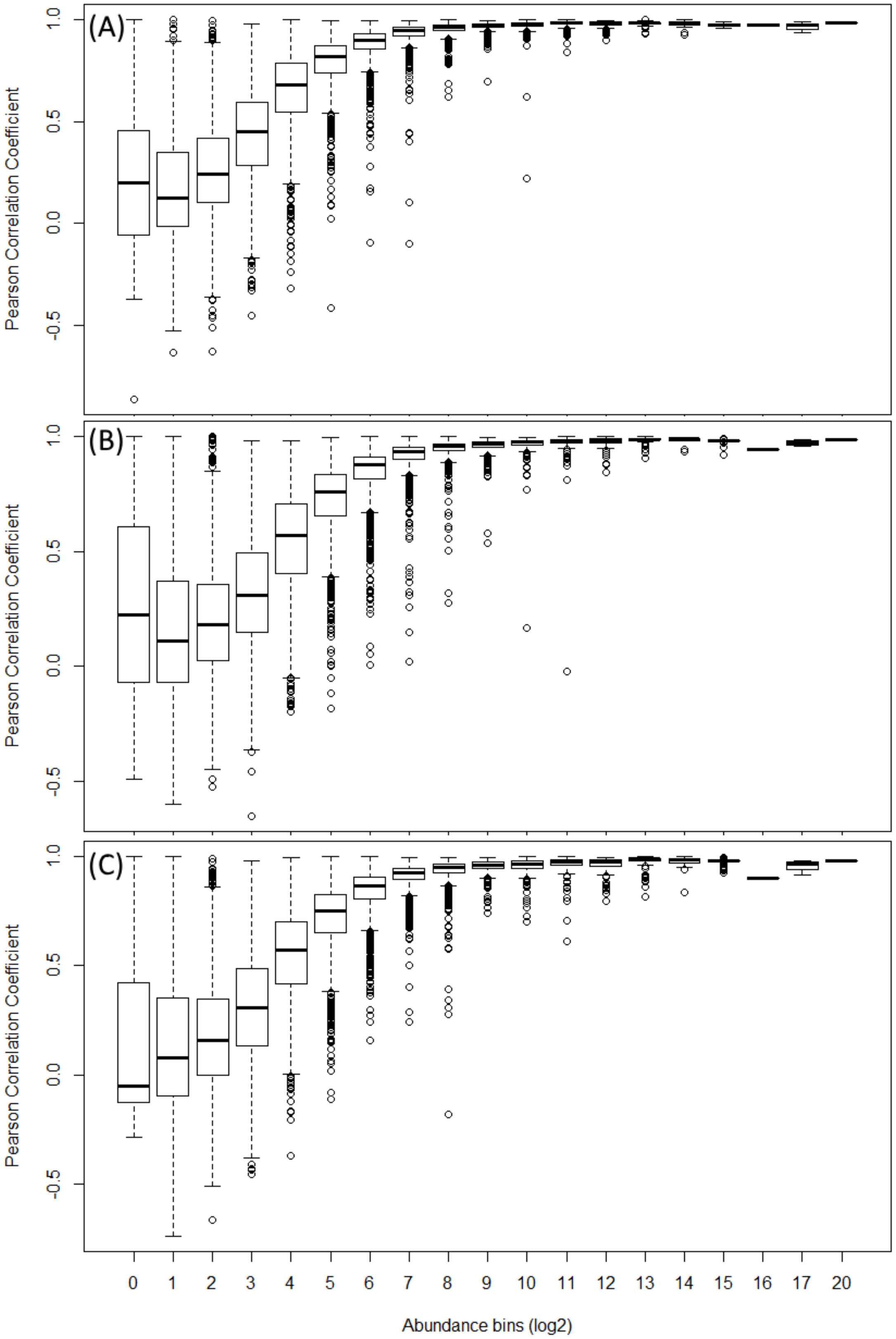

S3 Fig. Distribution of point-to-point Pearson Correlation Coefficient (PCC) (y-axis) between gene expression profiles against gene expression levels (x-axis, $\log_2$ scale) for pairwise comparisons for the *D. melanogaster* data for the 3 replicates of the 02-H sample as an example (2h, HT body part, no rivals). Panel A shows replicate 1 vs 2, B replicate 1 vs 3, and C replicate 2 vs 3. Shown are the raw data, prior to normalization. For all replicate comparisons, more variability was consistently observed at lower abundances and derived mostly from the small number or scattered incident reads. For the higher abundance genes, the p2pPCC was tight, indicating a high reproducibility of the expression profile and a low incidence of alternative splicing events. This analysis formed the basis of the offset identification i.e. the offset was selected to be the value for which, for all replicates, for all samples, the median of the p2pPCC was above 0.5.
