# Supplementary material for: Comparison of alternative approaches for analysing multi-level RNA-seq data
Source: PLoS One. 2017 Aug 8;12(8):e0182694. doi: 10.1371/journal.pone.0182694 (PMC5549751; doi:10.1371/journal.pone.0182694)
Supplement: S4 Fig — To indicate the consistency during the subsampling, without replacement, the plots show the point-to-point PCC between the original and incrementally subsampled (from 40% to 95%) data (Panels A to L; A: 40%, B: 45%, C: 50%, D: 55%, E: 60%, F: 65%, G: 70%, H: 75% I: 80%, J: 85%, K: 90%, L: 95%). On the x-axis is the gene abundance (log2) and on the y-axis the distribution of point-to-point PCCs calculated for each expressed gene. (PDF) [file pone.0182694.s011.pdf]

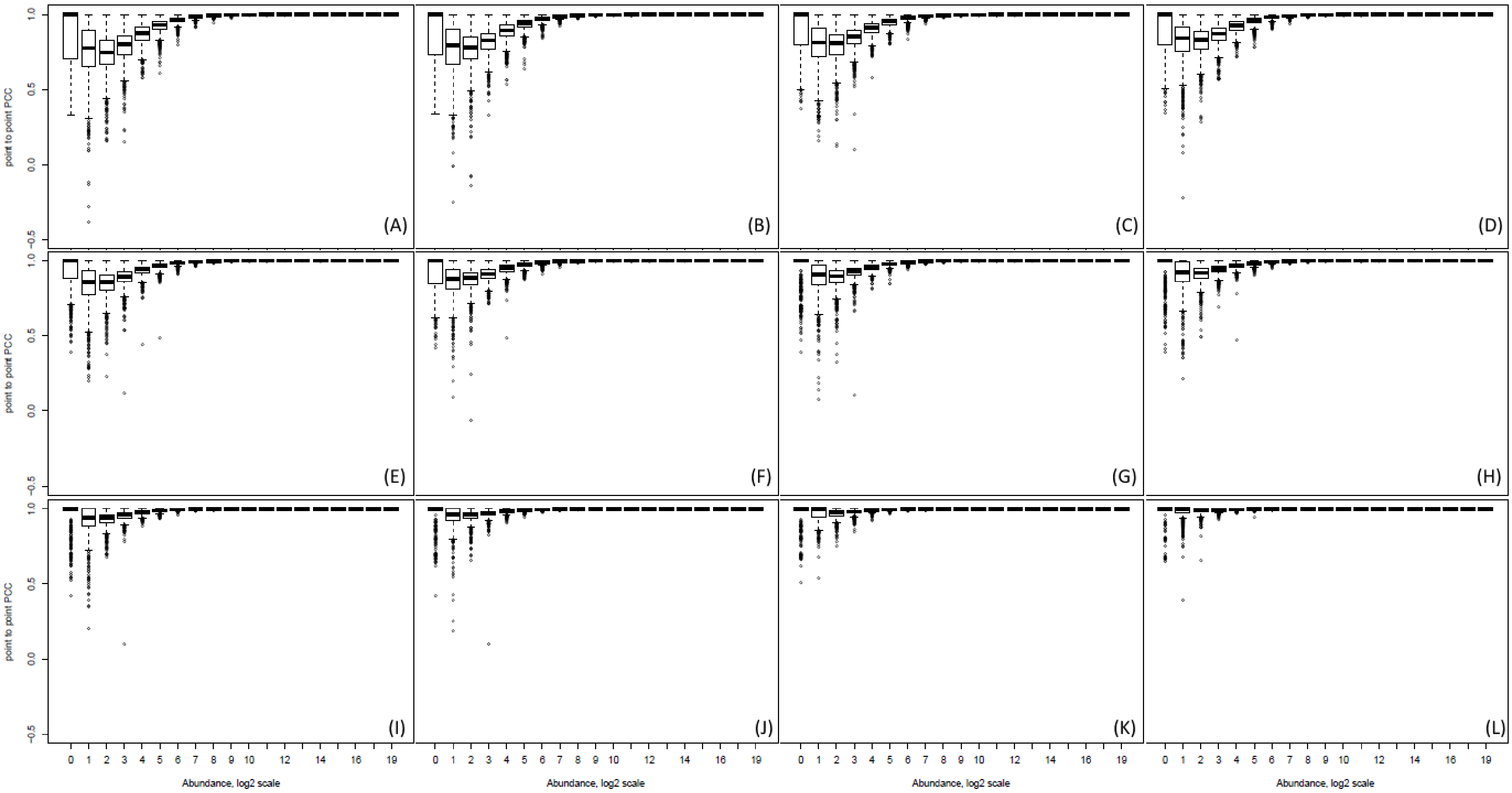

S4 Fig. Point-to-point Pearson Correlation Coefficient (PCC) between the raw and subsampled data for the 02+H3 sample of the *D. melanogaster* data (sample code: 02h, + rivals. HT body part replicate 3). To indicate the consistency during the subsampling, without replacement, the plots show the point-to-point PCC between the original and incrementally subsampled (from 40% to 95%) data (Panels A to L; A: 40%, B: 45%, C: 50%, D: 55%, E: 60%, F: 65%, G: 70%, H: 75% I: 80%, J: 85%, K: 90%, L: 95%). On the x-axis is the gene abundance (log_2) and on the y-axis the distribution of point-to-point PCCs calculated for each expressed gene.
