# Supplementary material for: Comparison of alternative approaches for analysing multi-level RNA-seq data
Source: PLoS One. 2017 Aug 8;12(8):e0182694. doi: 10.1371/journal.pone.0182694 (PMC5549751; doi:10.1371/journal.pone.0182694)
Supplement: S5 Fig — Frequency density plots were used to show the distribution of DE between samples (offset fold change, log2 scale). Panel A shows the replicate-replicate DE (blue) and the with/without rivals DE (red) for the abdomen (A) samples. Panel B shows the corresponding data for the HT (H) body part. Panel C shows the distribution of DE for the with/without rivals treatments (blue for HT and green for A samples) and the DE between H and A (orange). The DE distribution for the treatment (+/- rivals, red) was overlapping with the DE distribution between the replicates (blue), indicating a subtle DE signature. However, the DE distribution between body parts (orange) showed a good separation between HT- and AB- specific genes. These DE distributions supported the choice of a hierarchical design for the identification of DE genes between the treatments. (PDF) [file pone.0182694.s012.pdf]

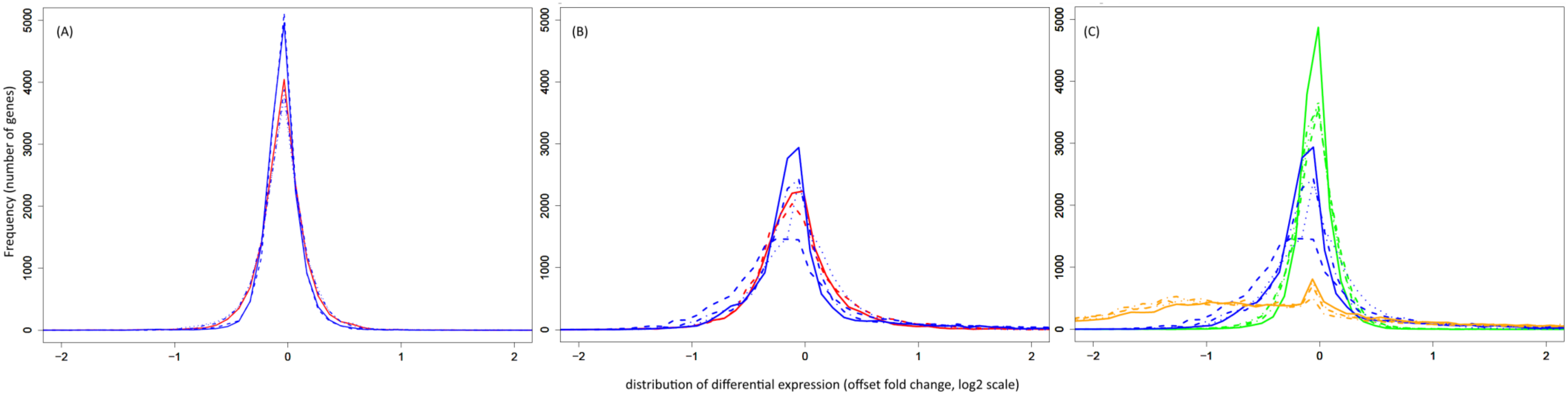

S5 Fig. **Identification of the hierarchy levels for the hierarchical differential expression (HDE) analysis based on the distribution of DE for the different classes of samples**, i.e. replicates, body parts and ± rivals treatments (for the *D. melanogaster* data). Frequency density plots were used to show the distribution of DE between samples (offset fold change, log_2 scale). Panel A shows the replicate-replicate DE (blue) and the with/without rivals DE (red) for the abdomen (A) samples. Panel B shows the corresponding data for the HT (H) body part. Panel C shows the distribution of DE for the with/without rivals treatments (blue for HT and green for A samples) and the DE between H and A (orange). The DE distribution for the treatment (+/- rivals, red) was overlapping with the DE distribution between the replicates (blue), indicating a subtle DE signature. However, the DE distribution between body parts (orange) showed a good separation between HT- and AB- specific genes. These DE distributions supported the choice of a hierarchical design for the identification of DE genes between the treatments.
