# Supplementary material for: Comparison of alternative approaches for analysing multi-level RNA-seq data
Source: PLoS One. 2017 Aug 8;12(8):e0182694. doi: 10.1371/journal.pone.0182694 (PMC5549751; doi:10.1371/journal.pone.0182694)
Supplement: S6 Fig — (Sample code: 02h, no rivals, A body part). On the x-axis we represent the average abundance between replicates (log2 scale), on the y-axis the FC (log2 scale). Although the quantile and the DESeq2 produced very similar distributions of abundances between replicates/samples, the former did not produce a tight MA plot when variability in expression at gene level was assessed. For the comparable results for the subsampling (without replacement) approach, see main Fig 3C. (PDF) [file pone.0182694.s013.pdf]

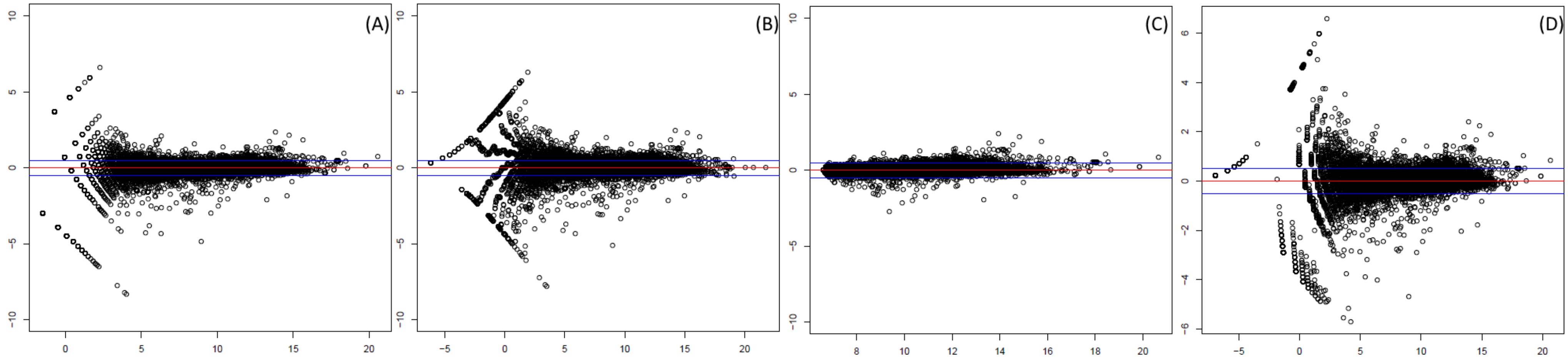

S6 Fig. **Replicate-to-replicate MA plot on the 02-A samples for checking the efficiency of the A RPM, B quantile, C DESeq2 and D edgeR normalization methods**. (Sample code: 02h, no rivals, A body part). On the x-axis we represent the average abundance between replicates (log_2 scale), on the y-axis the FC (log_2 scale). Although the quantile and the DESeq2 produced very similar distributions of abundances between replicates/samples, the former did not produce a tight MA plot when variability in expression at gene level was assessed. For the comparable results for the subsampling (without replacement) approach, see main Fig 3C.
