# Supplementary material for: Comparison of alternative approaches for analysing multi-level RNA-seq data
Source: PLoS One. 2017 Aug 8;12(8):e0182694. doi: 10.1371/journal.pone.0182694 (PMC5549751; doi:10.1371/journal.pone.0182694)
Supplement: S7 Fig — EdgeR only genes shown in S7A Fig, DEseq2 only in S7B Fig and subsampling normalization (without replacement) only in S7C Fig Genes are denoted by their FBgn identifiers. For each gene identified as DE exclusively by each method, the normalized abundance is given for each of the 2h HT (H) and A ± rivals samples. The leaky genes visible in the DE calls of edgeR and DESeq2 are not highlighted as DE using our adapted DE approach (i.e. hierarchical design and use of offset). (PDF) [file pone.0182694.s014.pdf]

S7 Fig. **Distribution of abundances for the *D. melanogaster* data (for the ± rivals treatment DE) for the full set of genes identified as DE exclusively by each method**. EdgeR only genes shown in S7A Fig, DEseq2 only in S7B Fig and subsampling normalization (without replacement) only in S7C Fig. Genes are denoted by their FBgn identifiers. For each gene identified as DE exclusively by each method, the normalized abundance is given for each of the 2h HT (H) and A ± rivals samples. The leaky genes visible in the DE calls of edgeR and DESeq2 are not highlighted as DE using our adapted DE approach (i.e. hierarchical design and use of offset).

Fig S7A

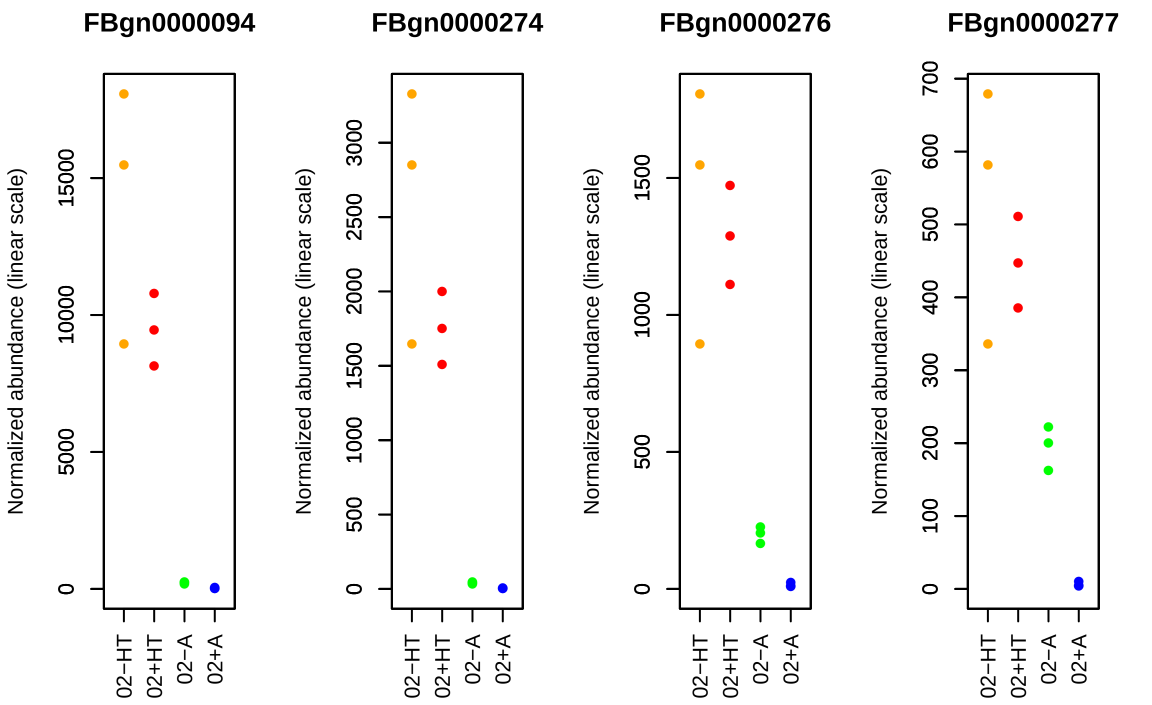
FBgn0000094
FBgn0000274
FBgn0000276
FBgn0000277
Normalized abundance (linear scale)
0
5000
10000
15000
500
1000
1500
2000
2500
3000
100
200
300
400
500
600
700
02−HT
02+HT
02−A
02+A

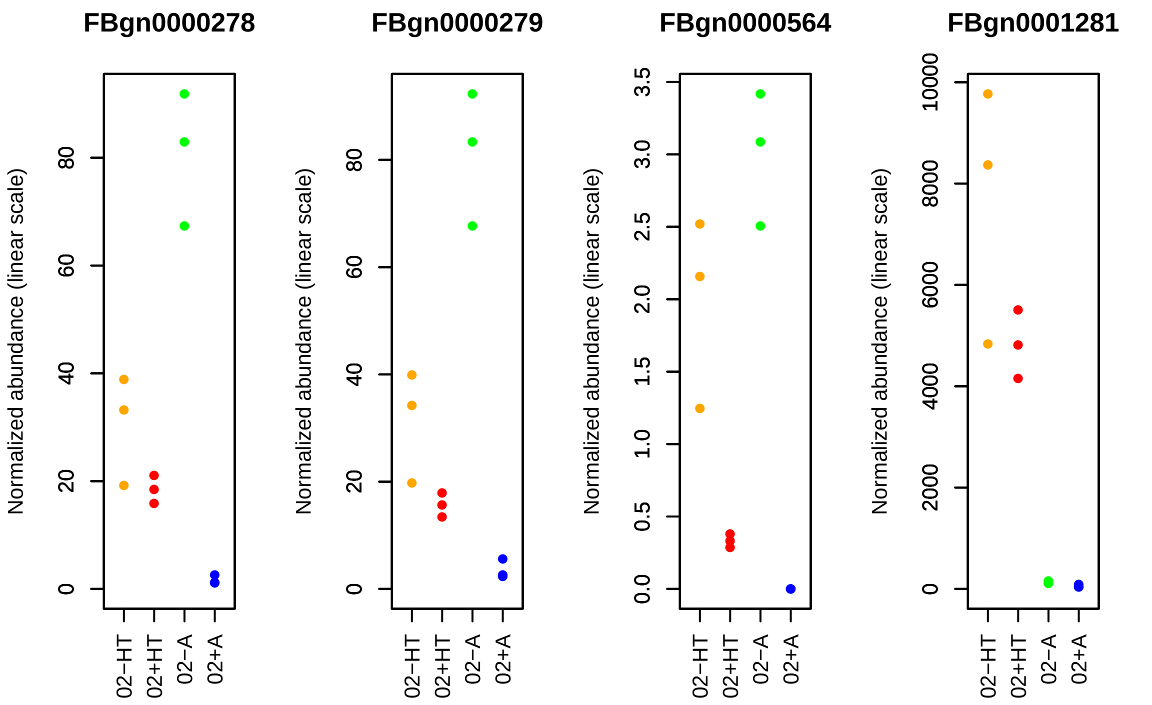

FBgn0000278
FBgn0000279
FBgn0000564
FBgn0001281
Normalized abundance (linear scale)
Normalized abundance (linear scale)
Normalized abundance (linear scale)
Normalized abundance (linear scale)
0
20
40
60
80
0
20
40
60
80
0.0
0.5
1.0
1.5
2.0
2.5
3.0
3.5
0
2000
4000
6000
8000
10000
02−HT
02+HT
02−A
02+A
02−HT
02+HT
02−A
02+A
02−HT
02+HT
02−A
02+A
02−HT
02+HT
02−A
02+A

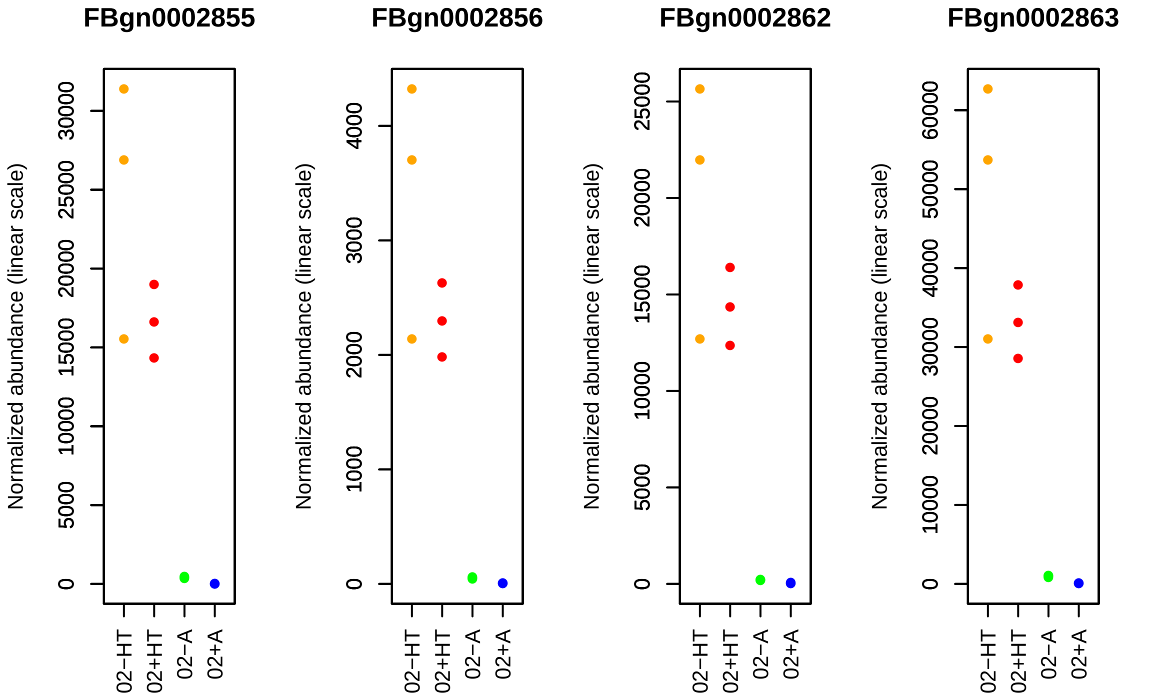

FBgn0002855
FBgn0002856
FBgn0002862
FBgn0002863
Normalized abundance (linear scale)
02−HT
02+HT
02−A
02+A

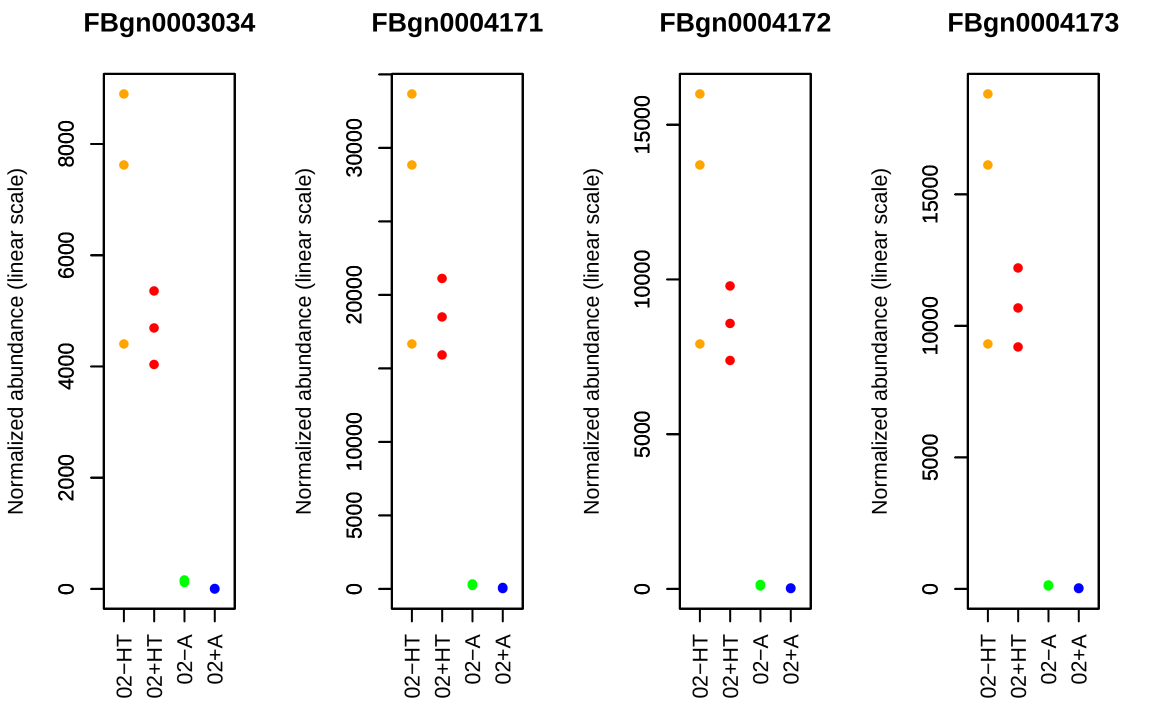

FBgn0003034
FBgn0004171
FBgn0004172
FBgn0004173
Normalized abundance (linear scale)
0
2000
4000
6000
8000
5000
10000
20000
30000
15000
02−HT
02+HT
02−A
02+A

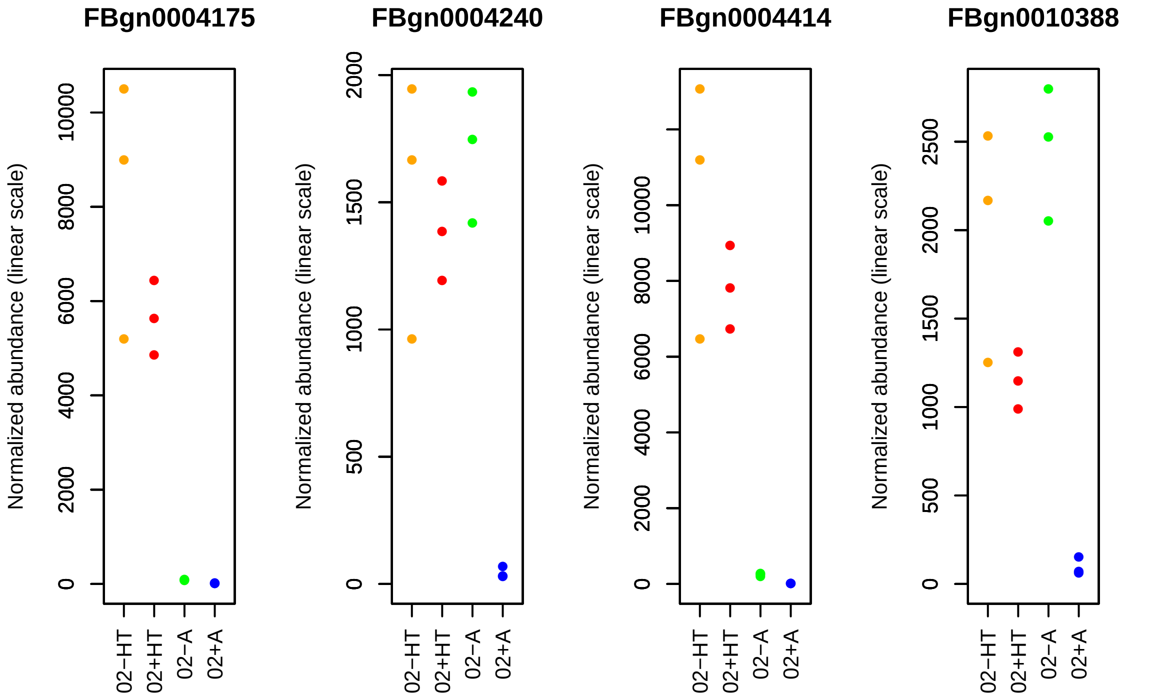

FBgn0004175
FBgn0004240
FBgn0004414
FBgn0010388
Normalized abundance (linear scale)
0
2000
4000
6000
8000
10000
500
1000
1500
2000
2500
02−HT
02+HT
02−A
02+A

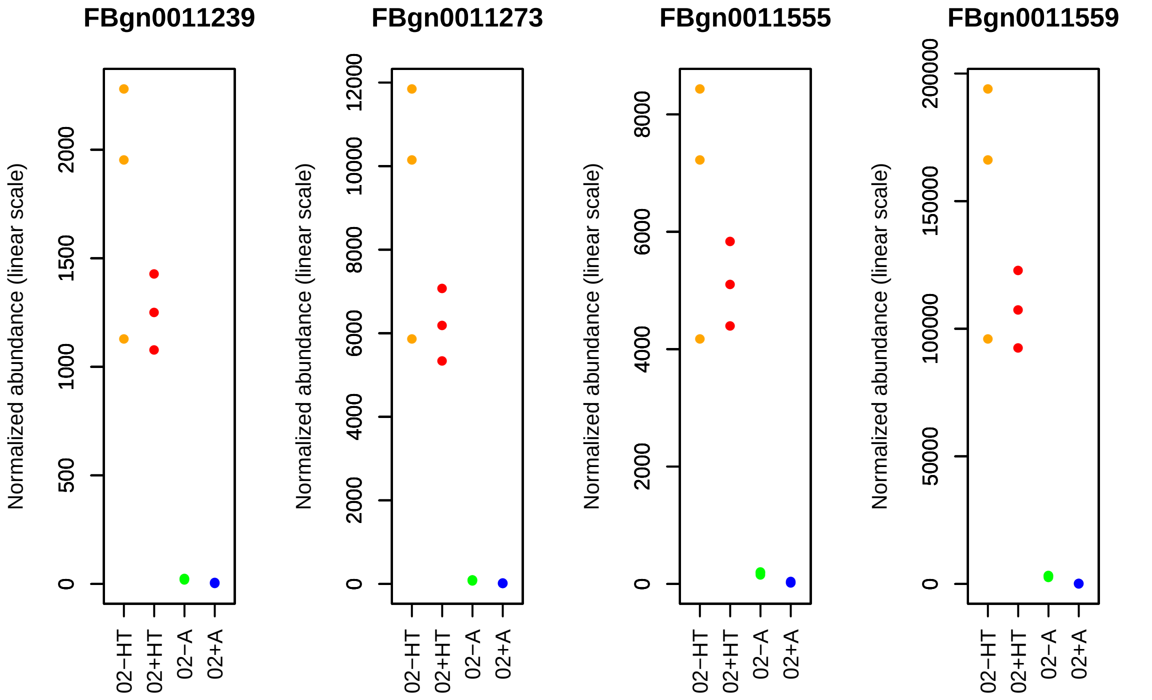

FBgn0011239
FBgn0011273
FBgn0011555
FBgn0011559
Normalized abundance (linear scale)
0
500
1000
1500
2000
2000
4000
6000
8000
10000
12000
50000
100000
150000
200000
02−HT
02+HT
02−A
02+A

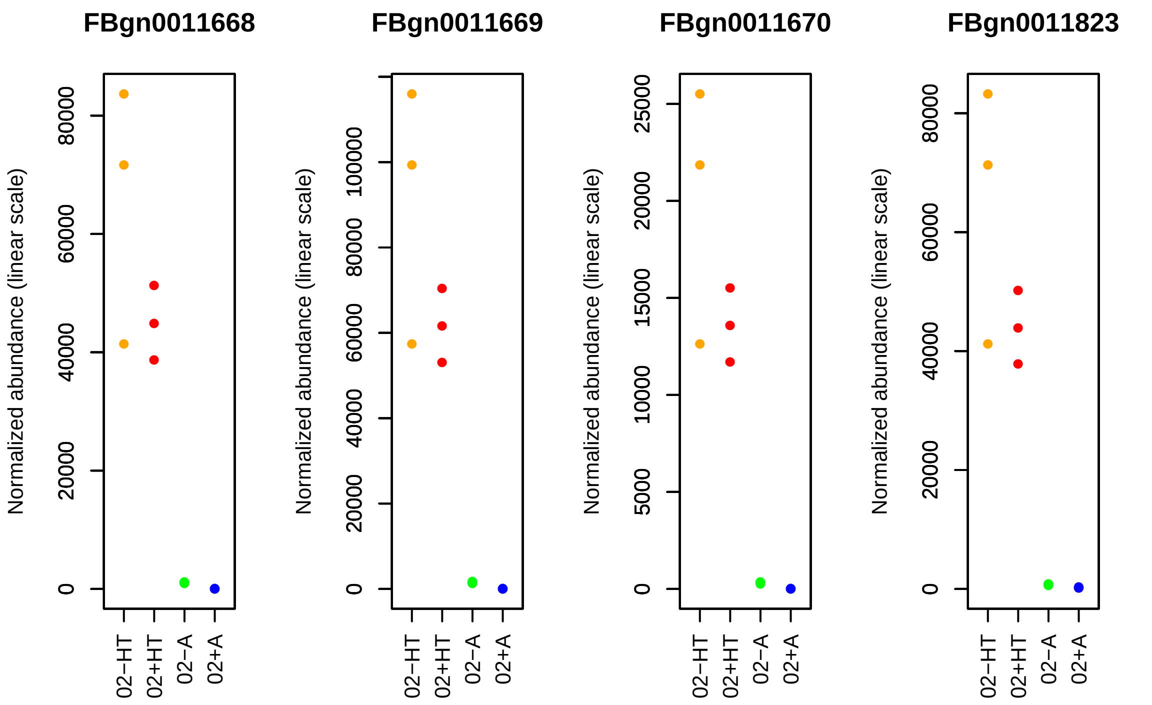

FBgn0011668
FBgn0011669
FBgn0011670
FBgn0011823
Normalized abundance (linear scale)
0
20000
40000
60000
80000
100000
5000
10000
15000
20000
25000
02−HT
02+HT
02−A
02+A

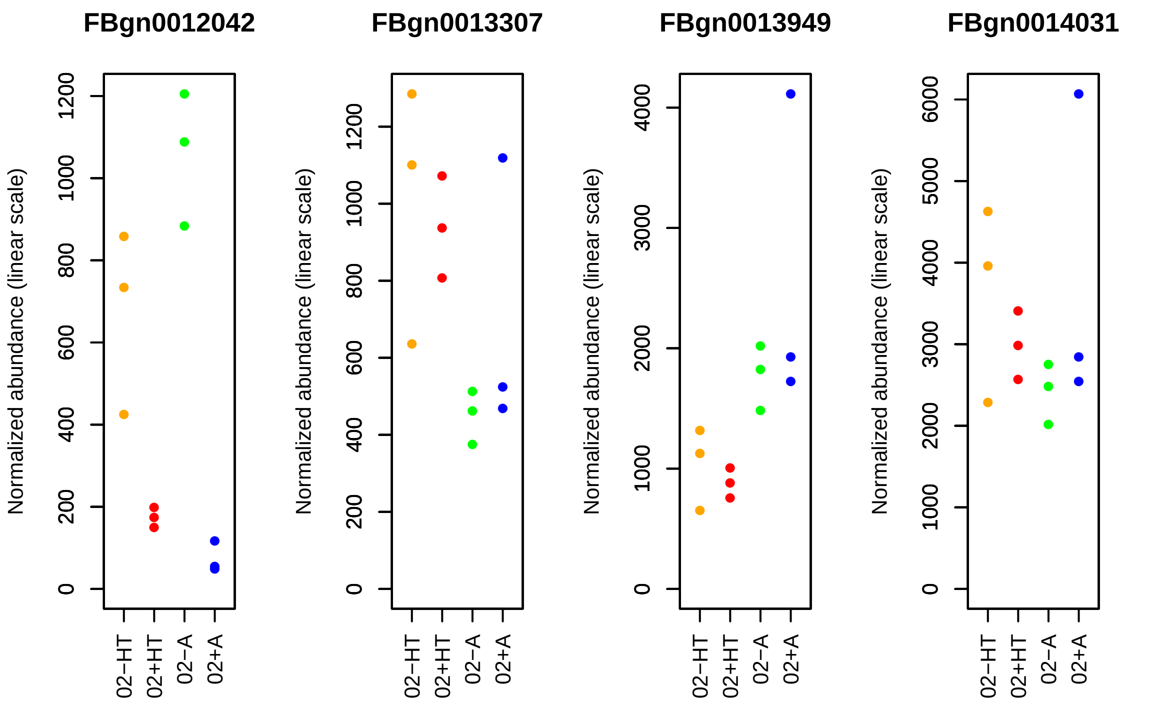
FBgn0012042
FBgn0013307
FBgn0013949
FBgn0014031
Normalized abundance (linear scale)
0
200
400
600
800
1000
1200
1000
2000
3000
4000
5000
6000
02−HT
02+HT
02−A
02+A

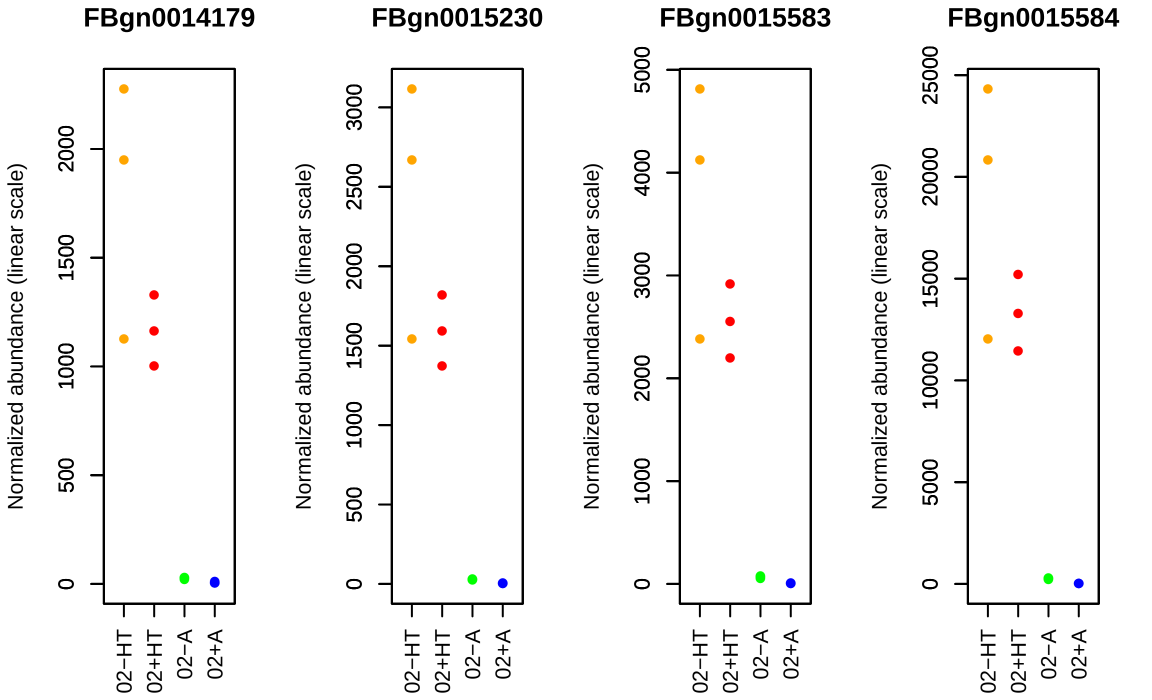
FBgn0014179
FBgn0015230
FBgn0015583
FBgn0015584
Normalized abundance (linear scale)
0
500
1000
1500
2000
2500
3000
4000
5000
10000
15000
20000
25000
02−HT
02+HT
02−A
02+A

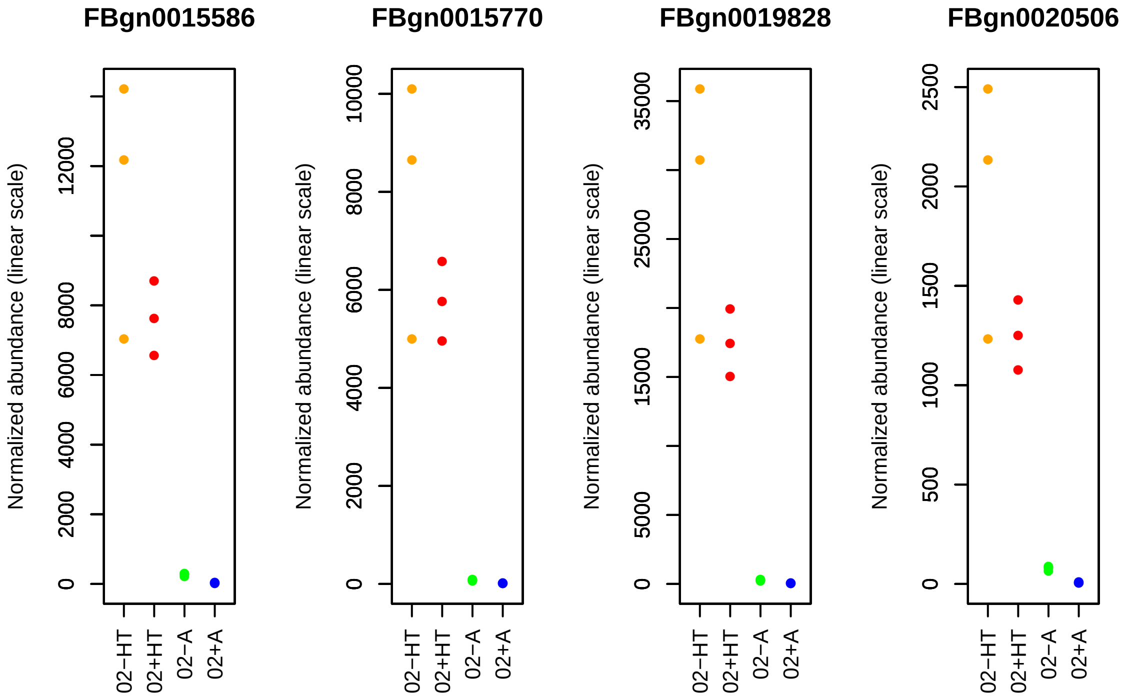
FBgn0015586
FBgn0015770
FBgn0019828
FBgn0020506
Normalized abundance (linear scale)
0
2000
4000
6000
8000
12000
10000
25000
35000
500
1000
1500
2000
2500
5000
15000
02−HT
02+HT
02−A
02+A

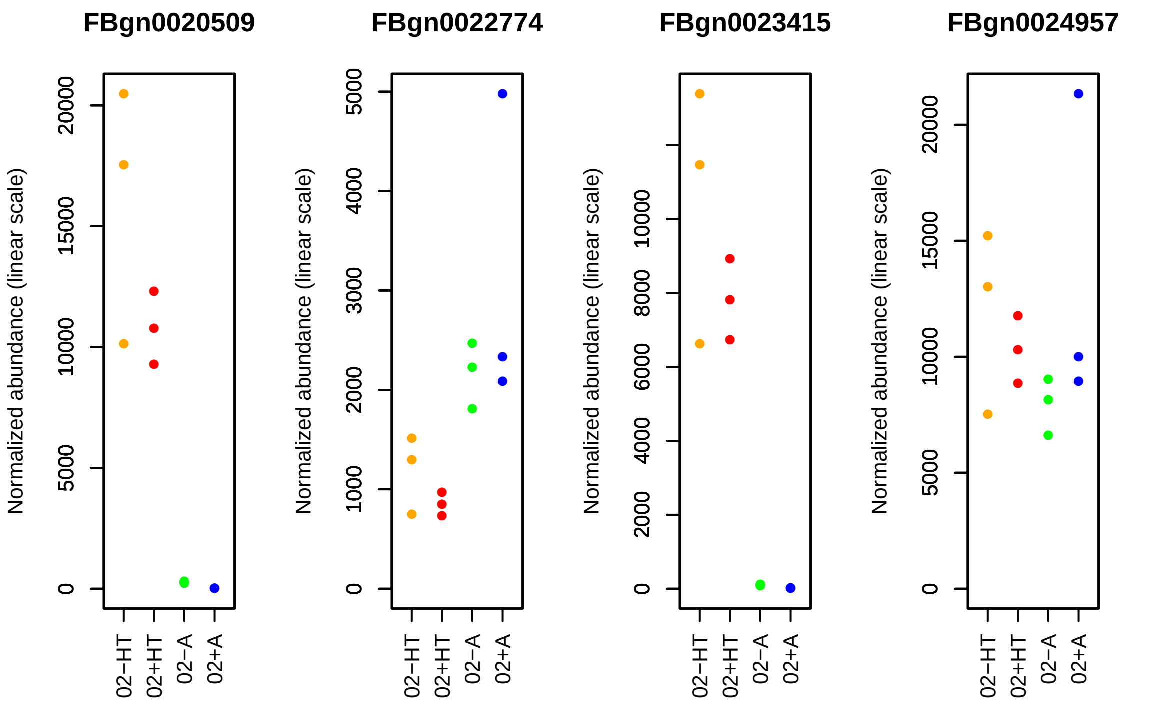

FBgn0020509
FBgn0022774
FBgn0023415
FBgn0024957
Normalized abundance (linear scale)
0
5000
10000
15000
20000
0
1000
2000
3000
4000
5000
0
2000
4000
6000
8000
10000
02−HT
02+HT
02−A
02+A

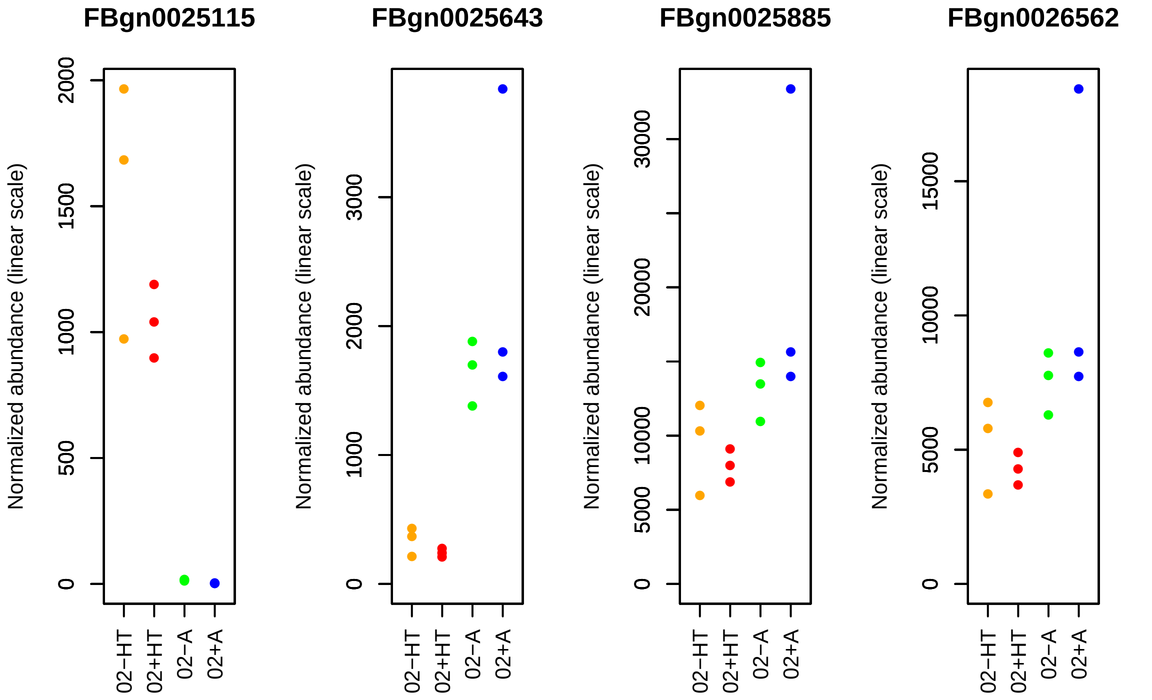

FBgn0025115
FBgn0025643
FBgn0025885
FBgn0026562
Normalized abundance (linear scale)
0
500
1000
1500
2000
3000
5000
10000
15000
20000
30000
02−HT
02+HT
02−A
02+A

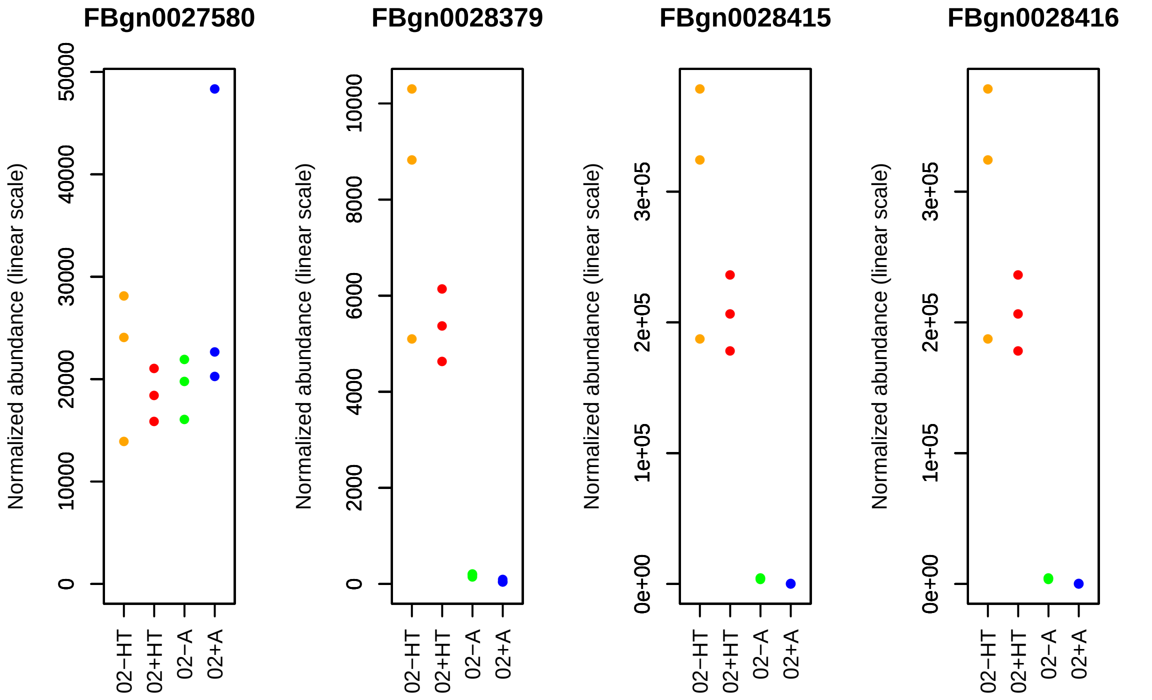
FBgn0027580
FBgn0028379
FBgn0028415
FBgn0028416
Normalized abundance (linear scale)
0
10000
20000
30000
40000
50000
2000
4000
6000
8000
10000
0e+00
1e+05
2e+05
3e+05
02−HT
02+HT
02−A
02+A

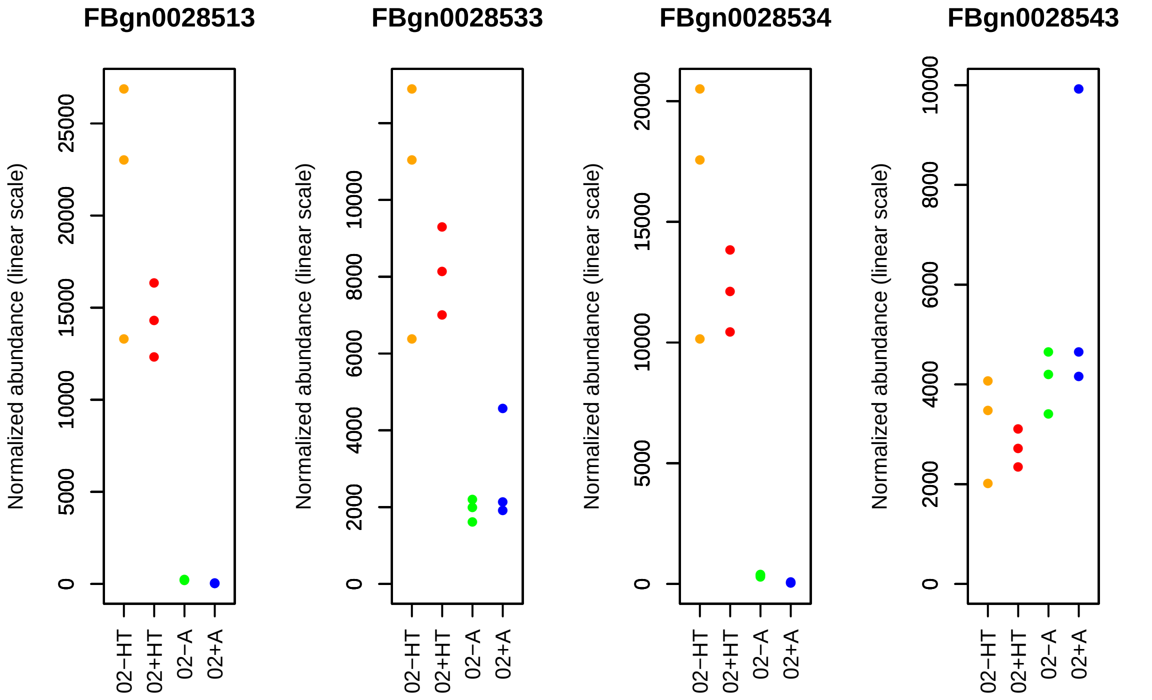
FBgn0028513
FBgn0028533
FBgn0028534
FBgn0028543
Normalized abundance (linear scale)
0
5000
10000
15000
20000
25000
2000
4000
6000
8000
10000
02−HT
02+HT
02−A
02+A

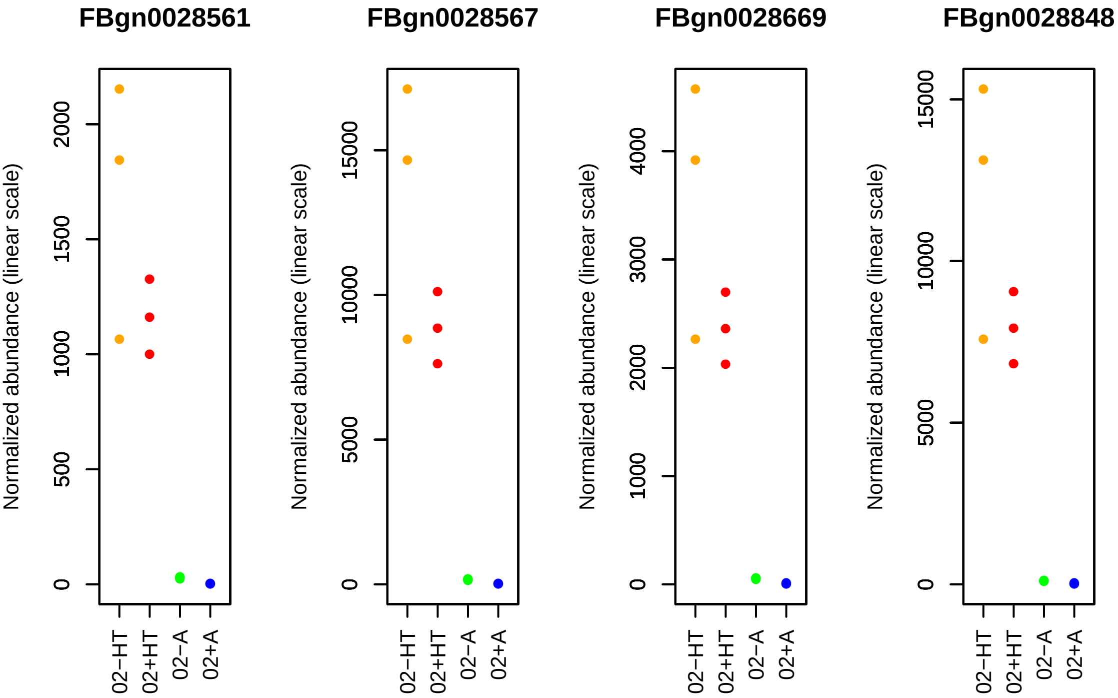

FBgn0028561
FBgn0028567
FBgn0028669
FBgn0028848
Normalized abundance (linear scale)
0
500
1000
1500
2000
5000
10000
15000
3000
4000
02−HT
02+HT
02−A
02+A

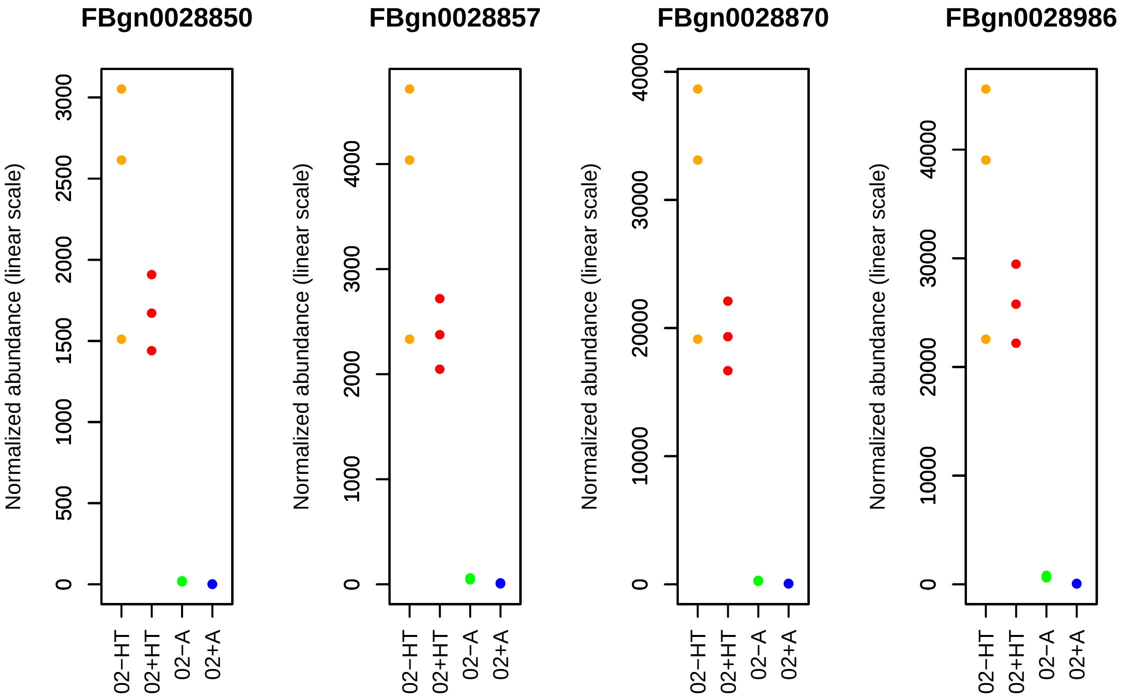

FBgn0028850
FBgn0028857
FBgn0028870
FBgn0028986
Normalized abundance (linear scale)
0
500
1000
1500
2000
2500
3000
4000
10000
20000
30000
40000
02−HT
02+HT
02−A
02+A

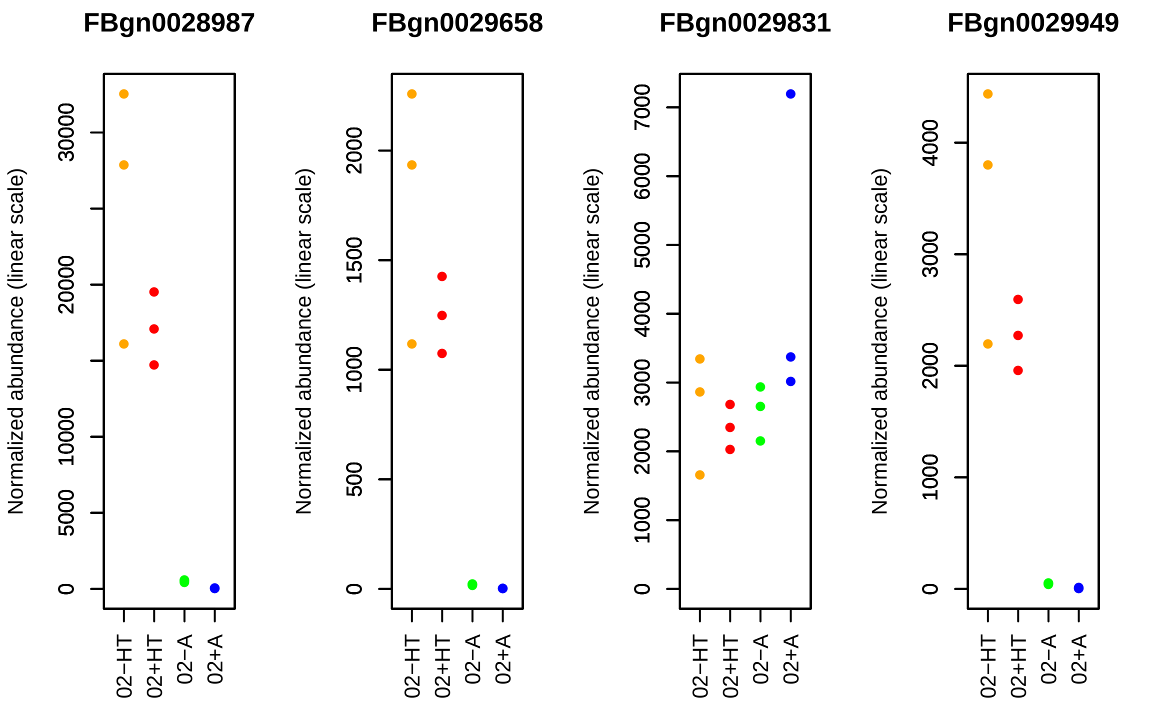
FBgn0028987
FBgn0029658
FBgn0029831
FBgn0029949
Normalized abundance (linear scale)
0
5000
10000
20000
30000
500
1000
1500
2000
1000
2000
3000
4000
5000
6000
7000
02−HT
02+HT
02−A
02+A

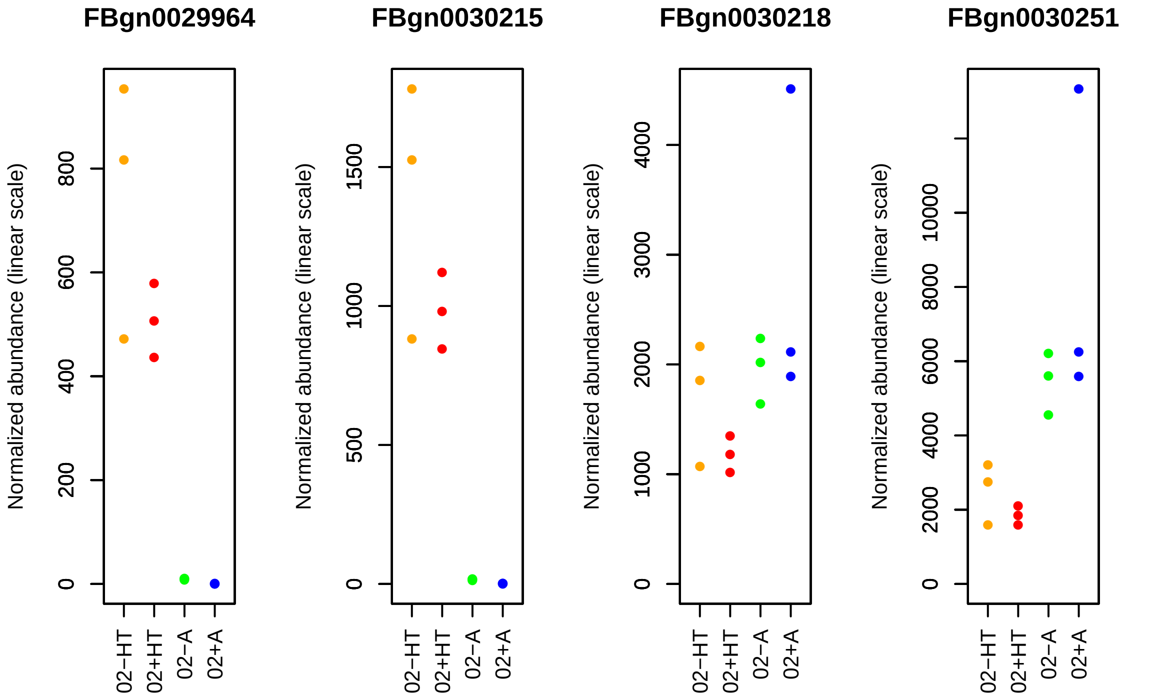

FBgn0029964
FBgn0030215
FBgn0030218
FBgn0030251
Normalized abundance (linear scale)
0
200
400
600
800
500
1000
1500
2000
3000
4000
2000
4000
6000
8000
10000
02−HT
02+HT
02−A
02+A

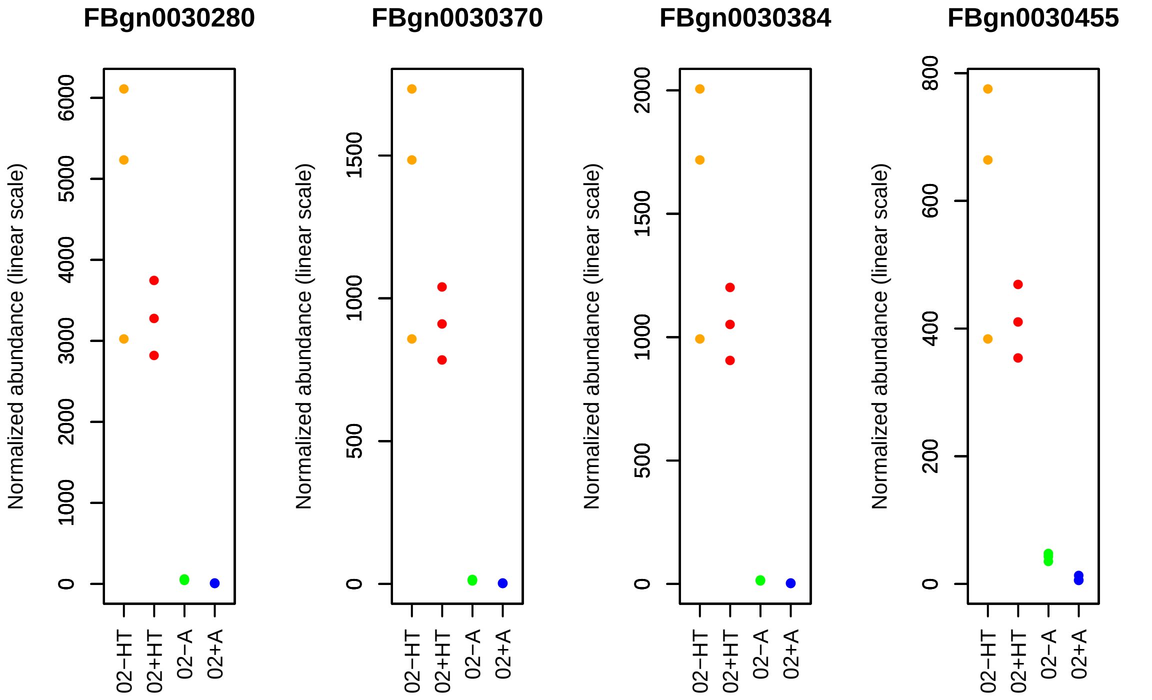

FBgn0030280
FBgn0030370
FBgn0030384
FBgn0030455
Normalized abundance (linear scale)
0
1000
2000
3000
4000
5000
6000
500
1500
200
400
600
800
02−HT
02+HT
02−A
02+A

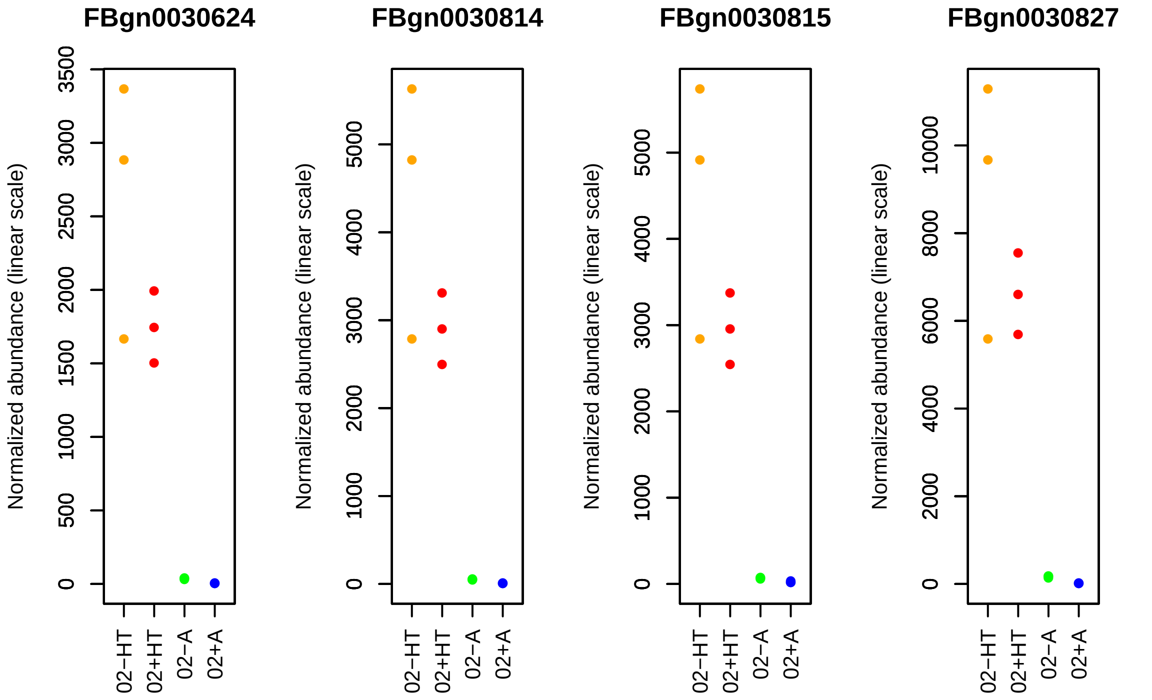
FBgn0030624
FBgn0030814
FBgn0030815
FBgn0030827
Normalized abundance (linear scale)
0
500
1000
1500
2000
2500
3000
3500
1000
2000
3000
4000
5000
4000
6000
8000
10000
02−HT
02+HT
02−A
02+A

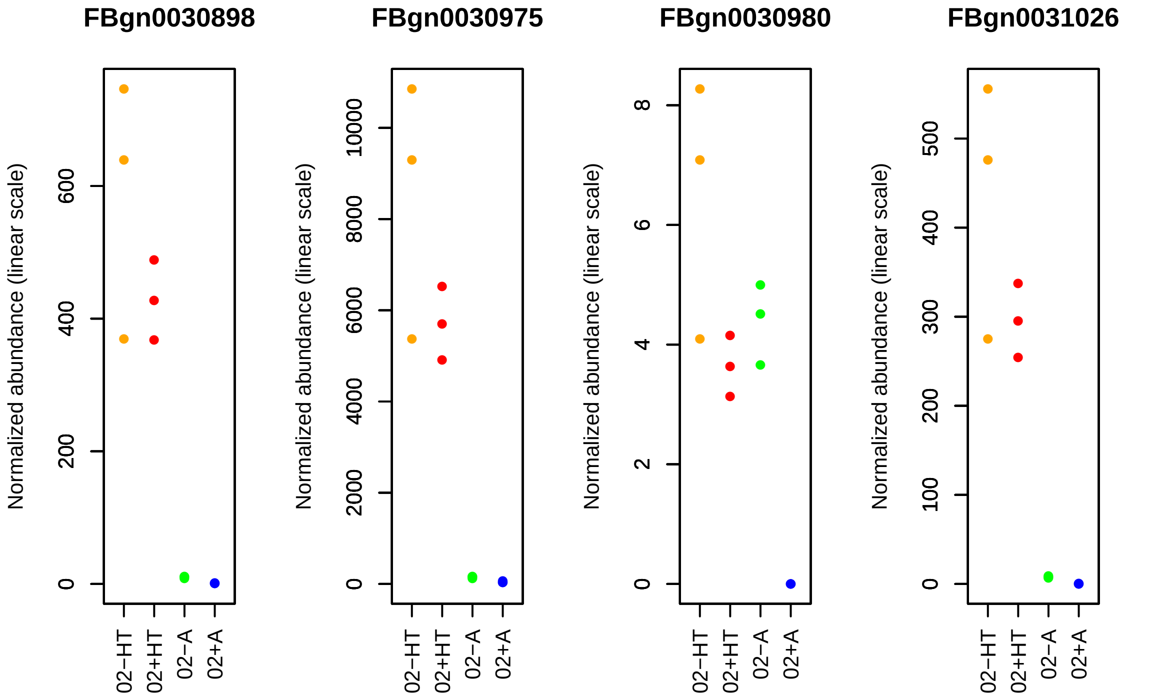

FBgn0030898
FBgn0030975
FBgn0030980
FBgn0031026
Normalized abundance (linear scale)
0
200
400
600
2000
4000
6000
8000
10000
2
4
6
8
100
300
500
02−HT
02+HT
02−A
02+A

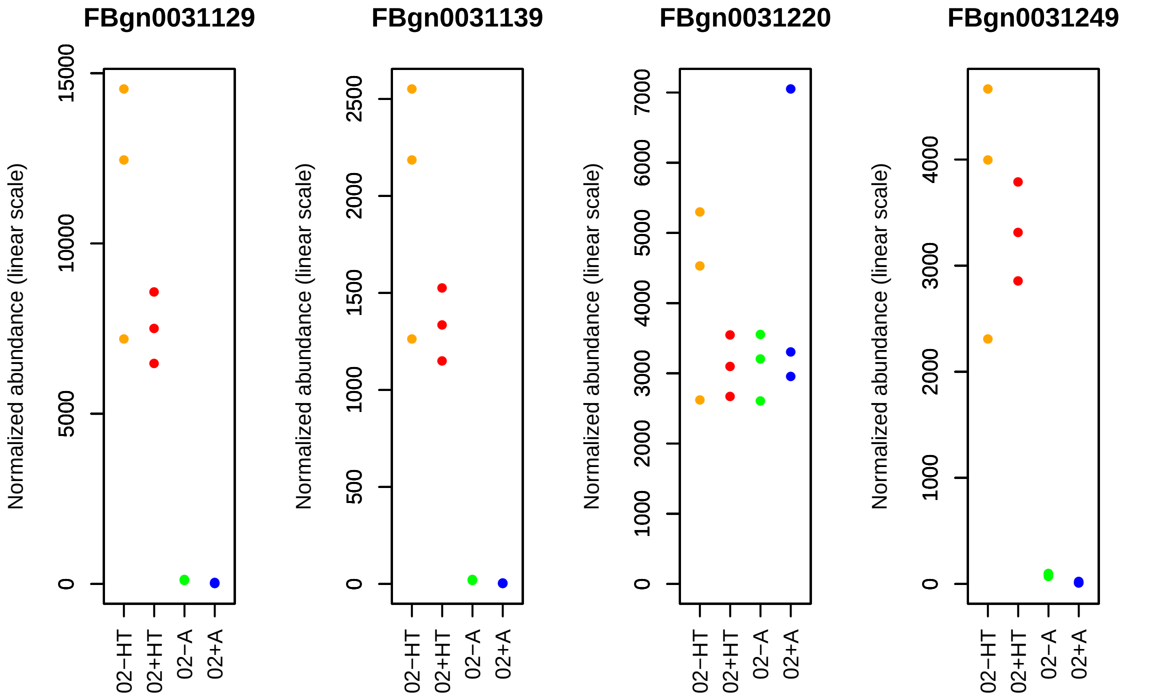

FBgn0031129
FBgn0031139
FBgn0031220
FBgn0031249
Normalized abundance (linear scale)
0
5000
10000
15000
500
1000
1500
2000
2500
2000
3000
4000
5000
6000
7000
02−HT
02+HT
02−A
02+A

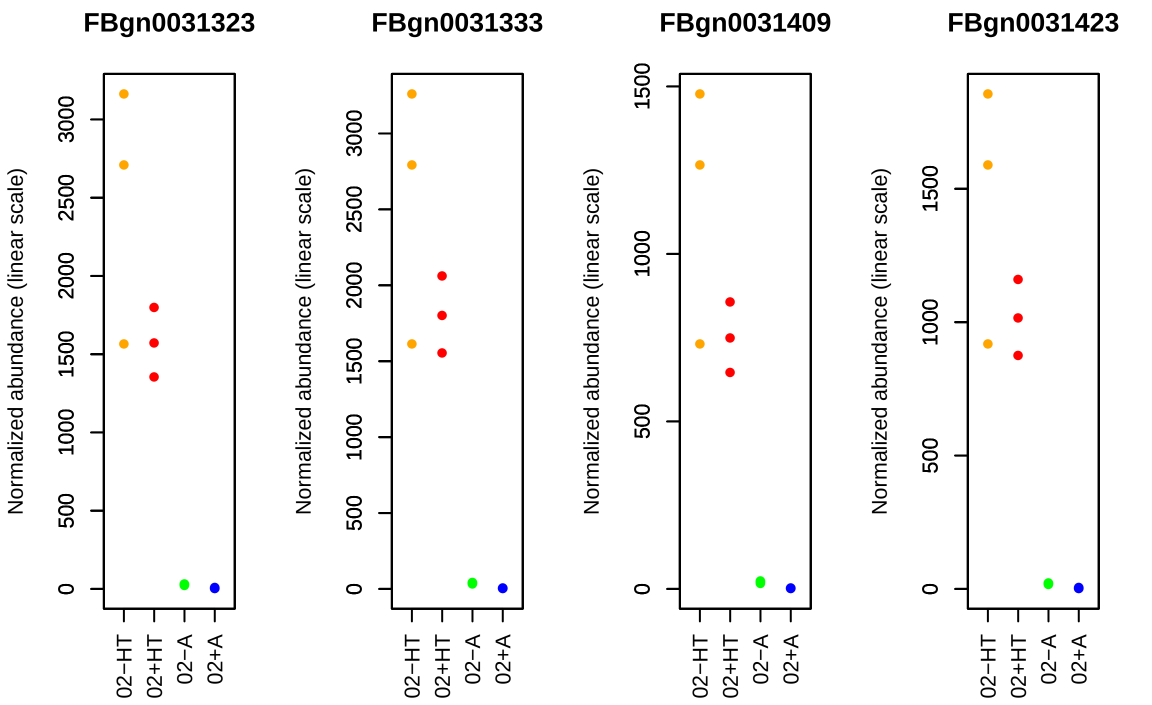

FBgn0031323
FBgn0031333
FBgn0031409
FBgn0031423
Normalized abundance (linear scale)
0
500
1000
1500
2000
2500
3000
02−HT
02+HT
02−A
02+A

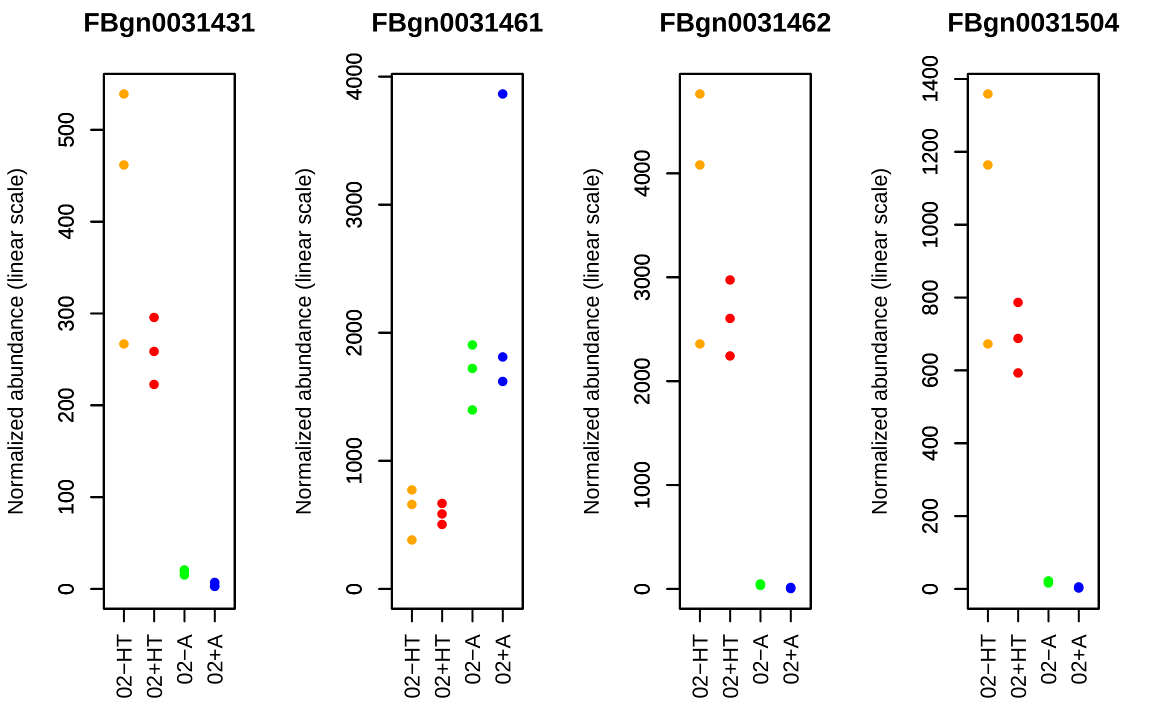

FBgn0031431
FBgn0031461
FBgn0031462
FBgn0031504
Normalized abundance (linear scale)
0
100
200
300
400
500
1000
2000
3000
4000
200
400
600
800
1000
1200
1400
02−HT
02+HT
02−A
02+A

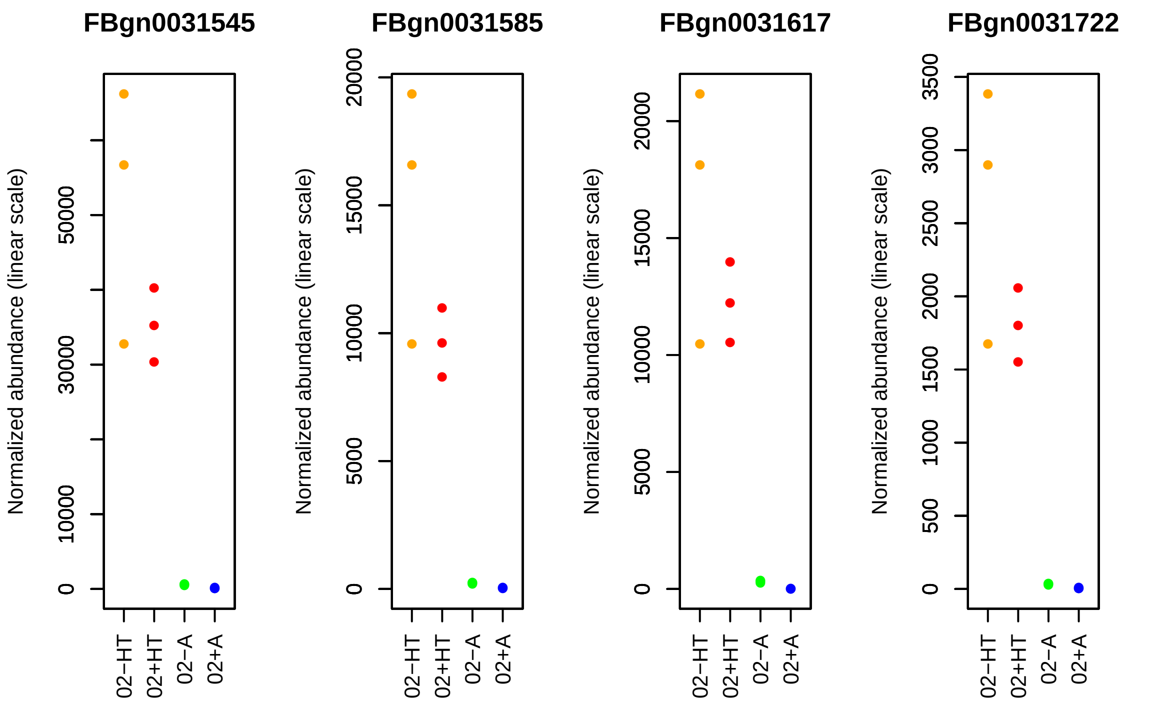

FBgn0031545
FBgn0031585
FBgn0031617
FBgn0031722
Normalized abundance (linear scale)
0
10000
30000
50000
5000
15000
20000
500
1000
1500
2000
2500
3000
3500
02−HT
02+HT
02−A
02+A

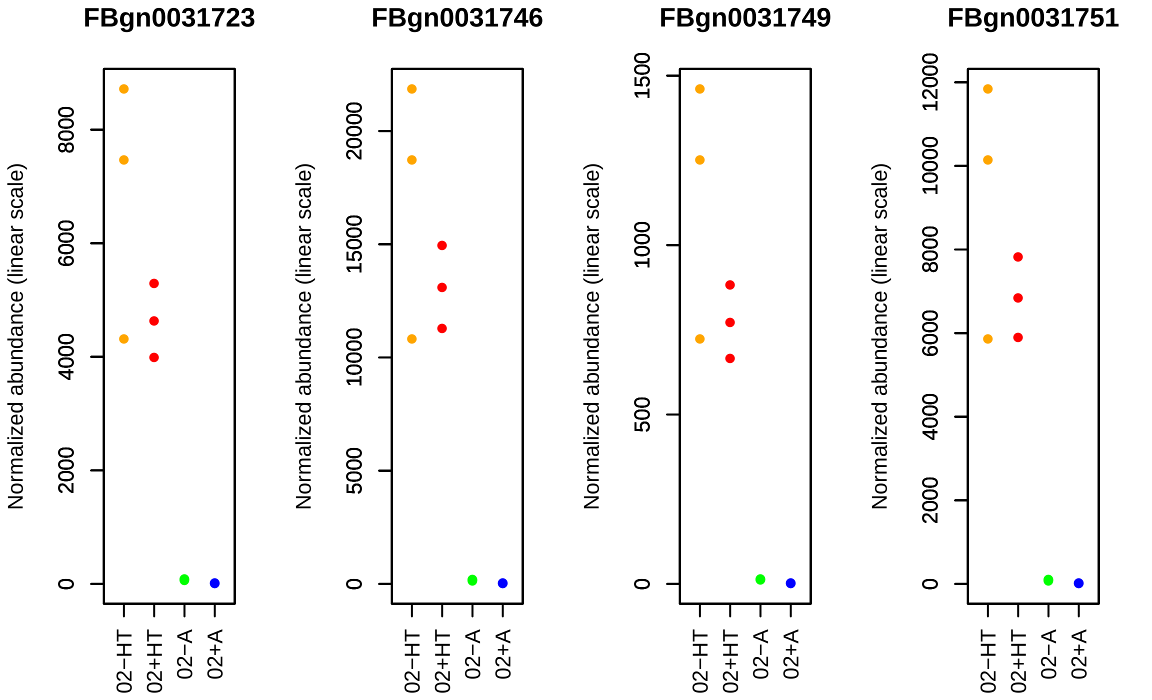
FBgn0031723
FBgn0031746
FBgn0031749
FBgn0031751
Normalized abundance (linear scale)
0
2000
4000
6000
8000
5000
10000
15000
20000
500
1000
1500
12000
02−HT
02+HT
02−A
02+A

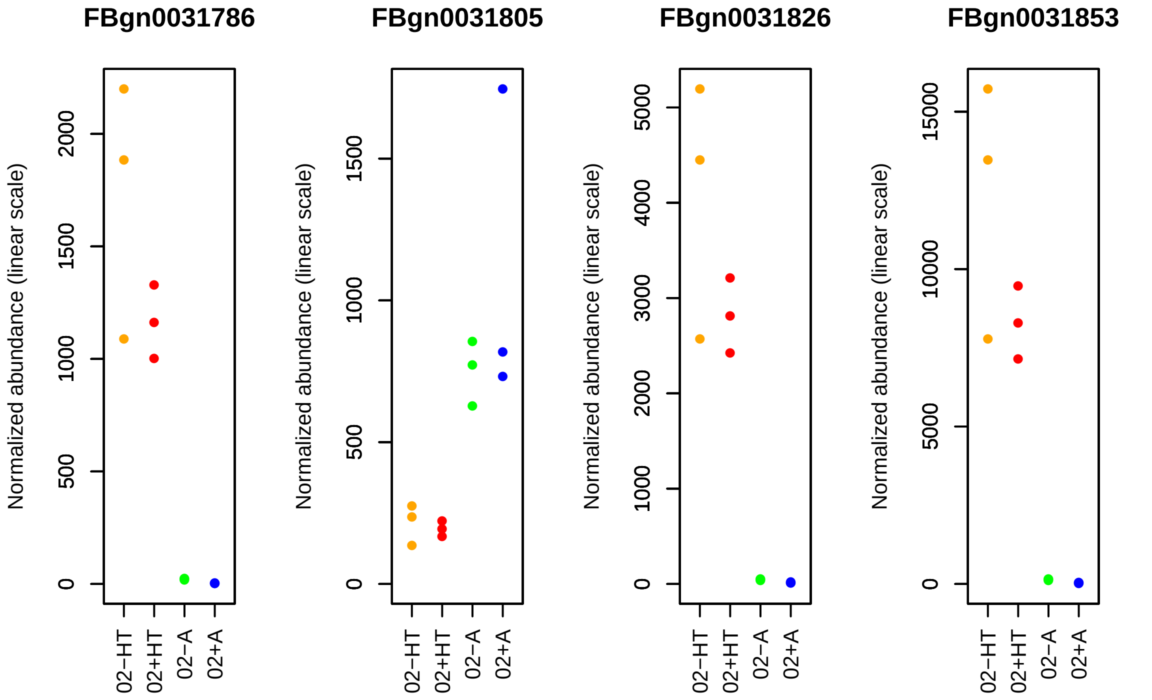

FBgn0031786
FBgn0031805
FBgn0031826
FBgn0031853
Normalized abundance (linear scale)
0
500
1000
1500
2000
5000
10000
15000
3000
4000
02−HT
02+HT
02−A
02+A

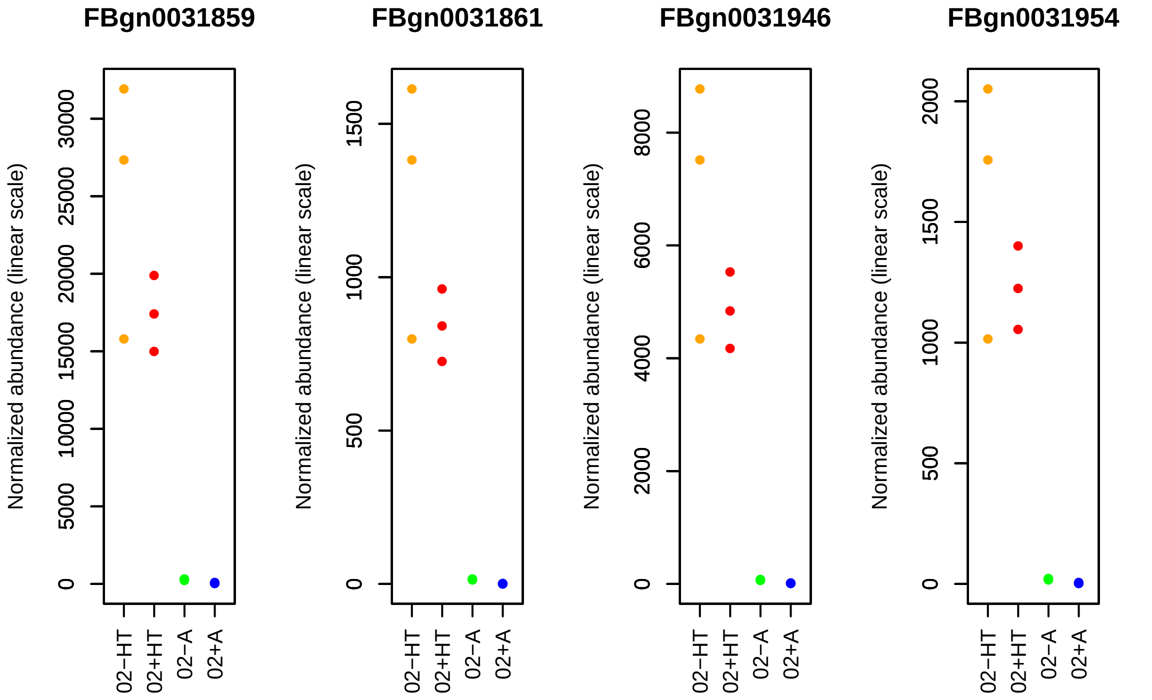

FBgn0031859
FBgn0031861
FBgn0031946
FBgn0031954
Normalized abundance (linear scale)
0
5000
10000
15000
20000
25000
30000
500
1000
1500
2000
4000
6000
8000
02−HT
02+HT
02−A
02+A

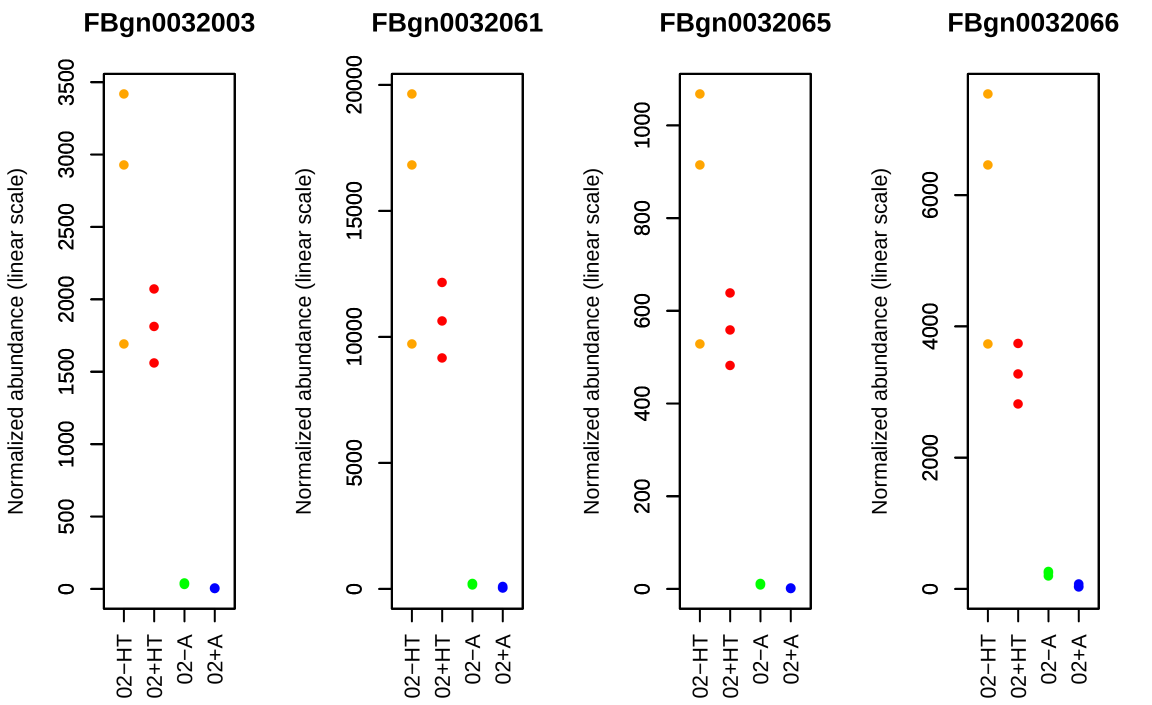

FBgn0032003
FBgn0032061
FBgn0032065
FBgn0032066
Normalized abundance (linear scale)
0
500
1000
1500
2000
2500
3000
3500
5000
10000
15000
20000
200
400
600
800
1000
2000
4000
6000
02−HT
02+HT
02−A
02+A

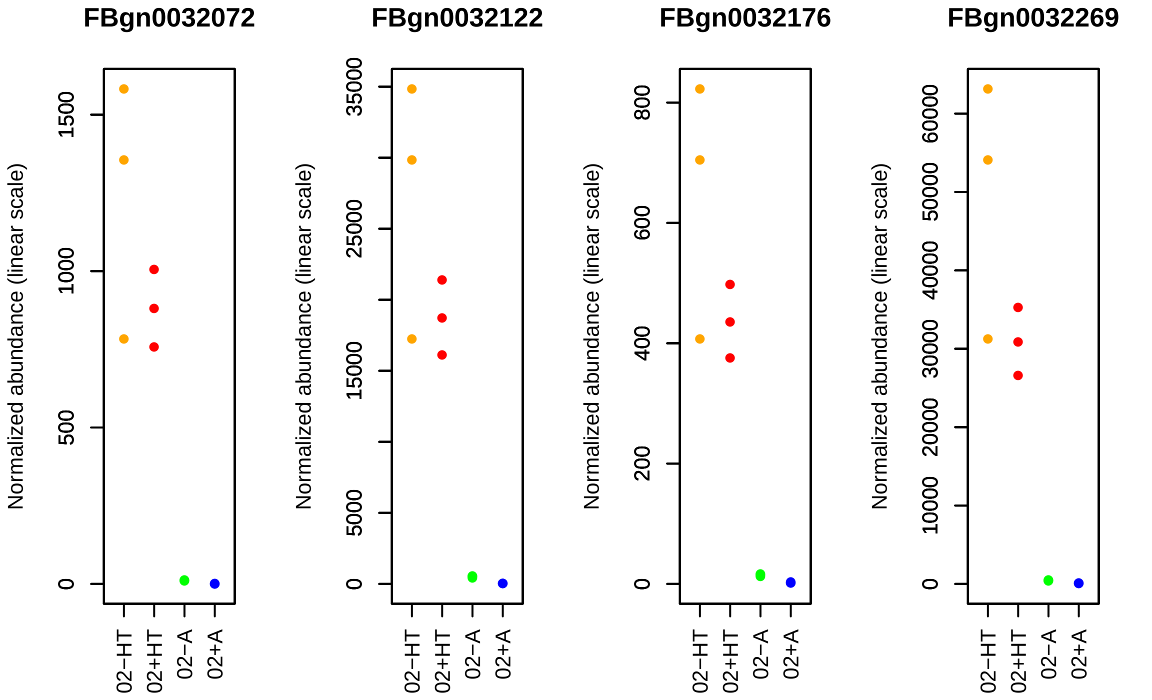
FBgn0032072
FBgn0032122
FBgn0032176
FBgn0032269
Normalized abundance (linear scale)
0
500
1000
1500
5000
15000
25000
35000
200
400
600
800
10000
20000
30000
40000
50000
60000
02−HT
02+HT
02−A
02+A

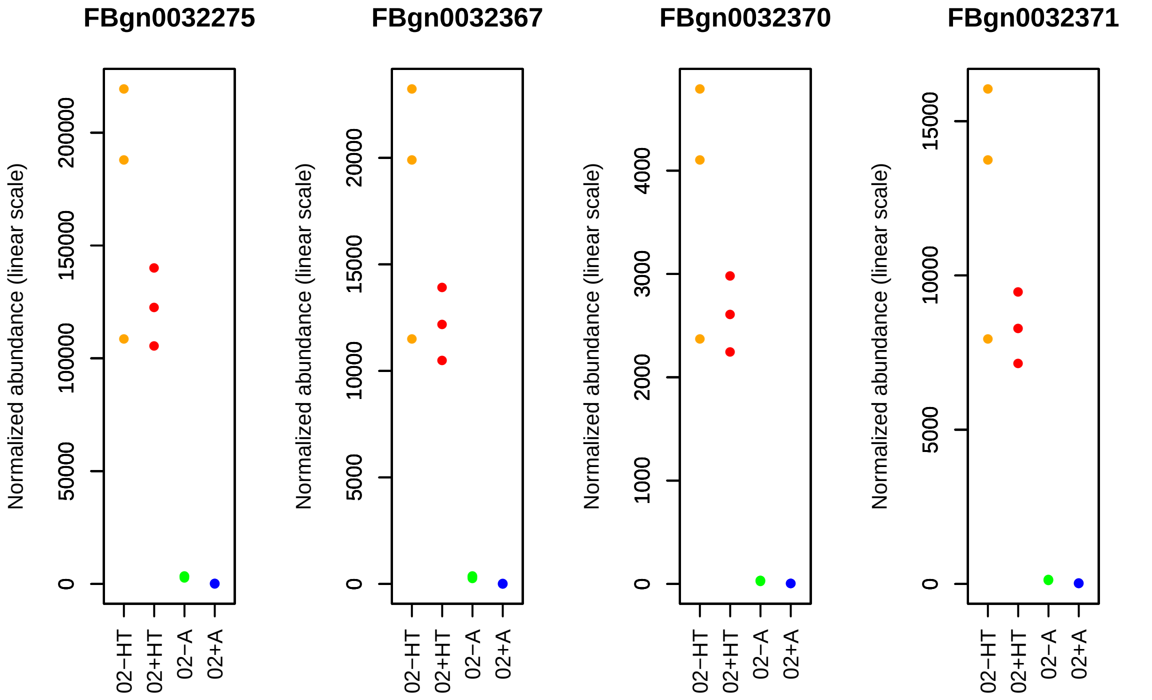

FBgn0032275
FBgn0032367
FBgn0032370
FBgn0032371
Normalized abundance (linear scale)
0
50000
100000
150000
200000
5000
10000
15000
20000
1000
2000
3000
4000
02−HT
02+HT
02−A
02+A

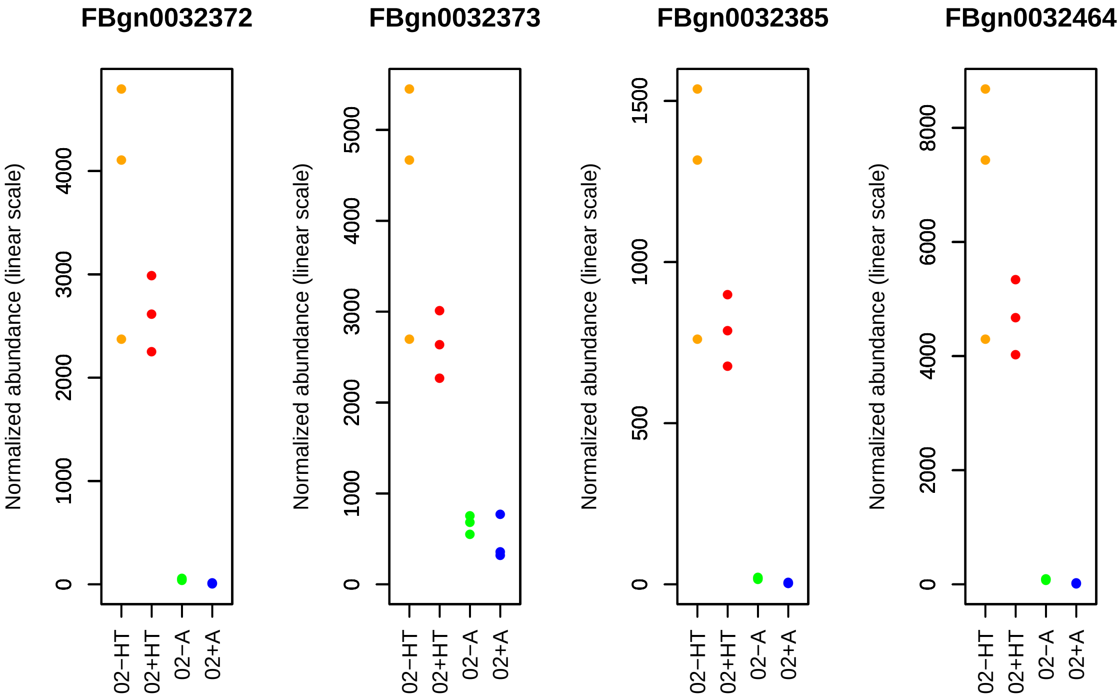
FBgn0032372
FBgn0032373
FBgn0032385
FBgn0032464
Normalized abundance (linear scale)
0
1000
2000
3000
4000
5000
500
1500
6000
8000
02−HT
02+HT
02−A
02+A

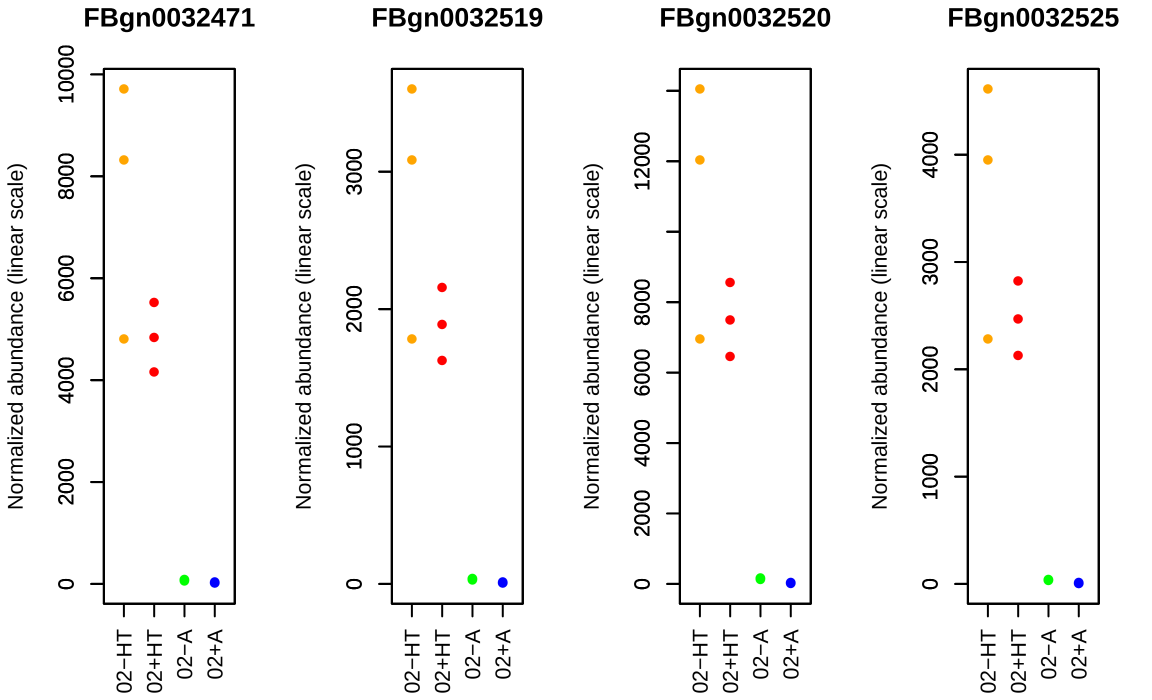
FBgn0032471
FBgn0032519
FBgn0032520
FBgn0032525
Normalized abundance (linear scale)
0
2000
4000
6000
8000
10000
1000
3000
12000
02−HT
02+HT
02−A
02+A

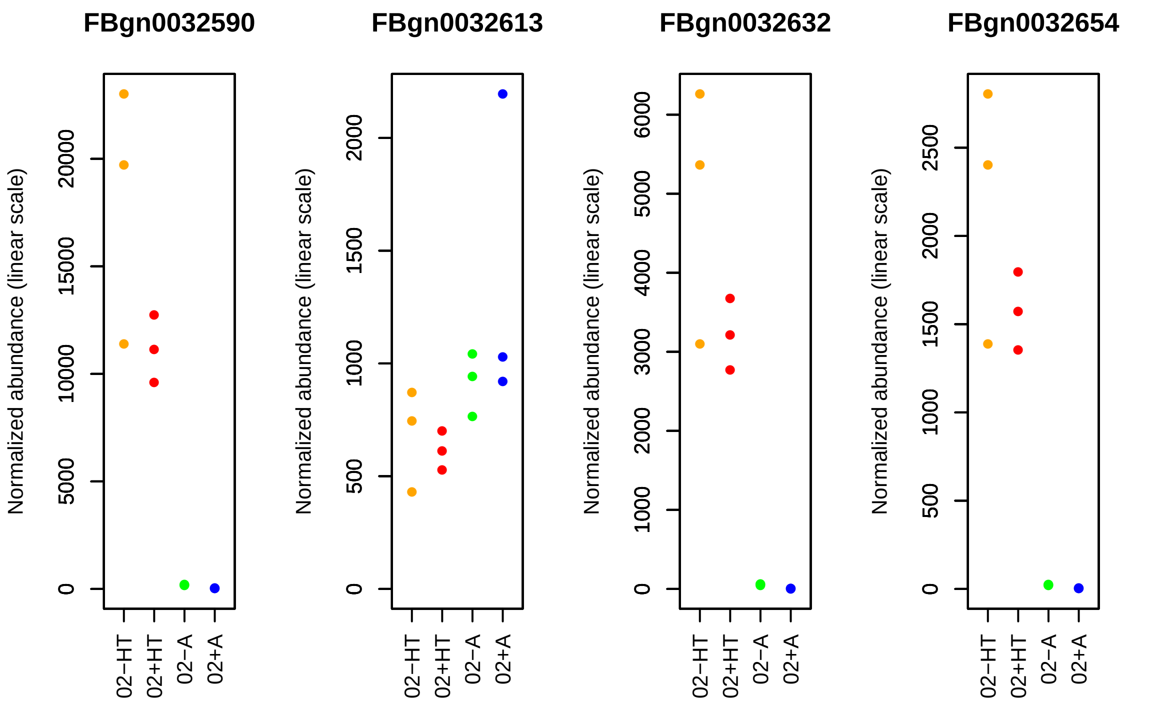
FBgn0032590
FBgn0032613
FBgn0032632
FBgn0032654
Normalized abundance (linear scale)
0
5000
10000
15000
20000
500
1000
1500
2000
2000
3000
4000
5000
6000
2500
02−HT
02+HT
02−A
02+A

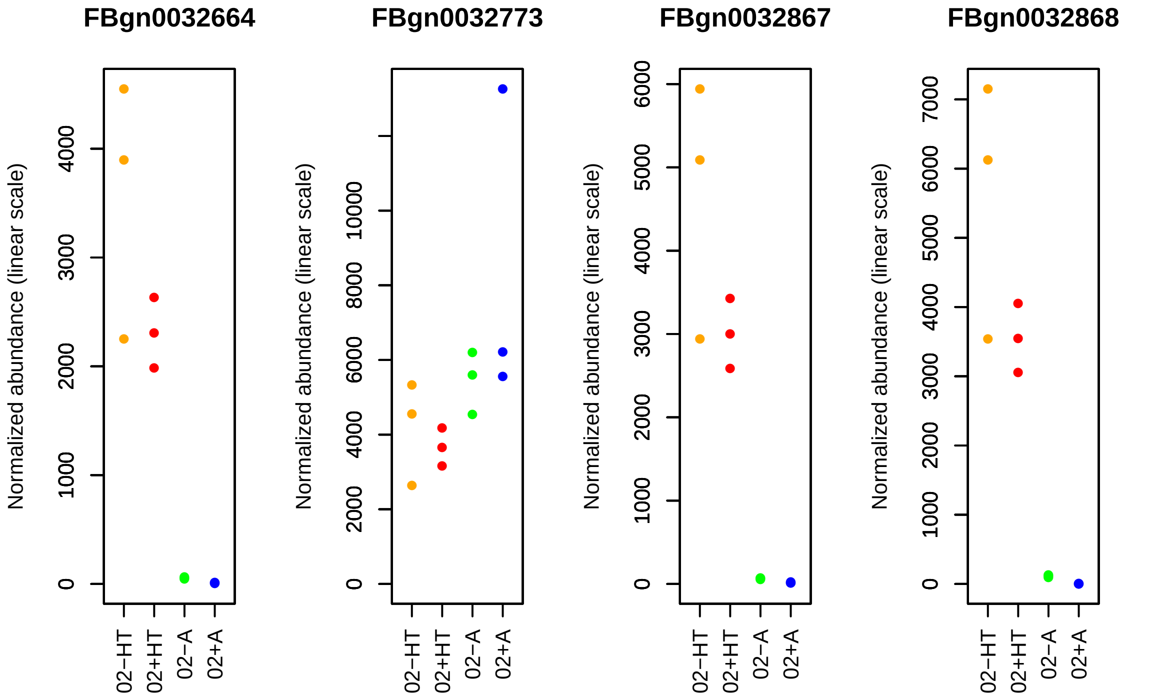

FBgn0032664
FBgn0032773
FBgn0032867
FBgn0032868
Normalized abundance (linear scale)
0
1000
2000
3000
4000
5000
6000
7000
8000
10000
02−HT
02+HT
02−A
02+A

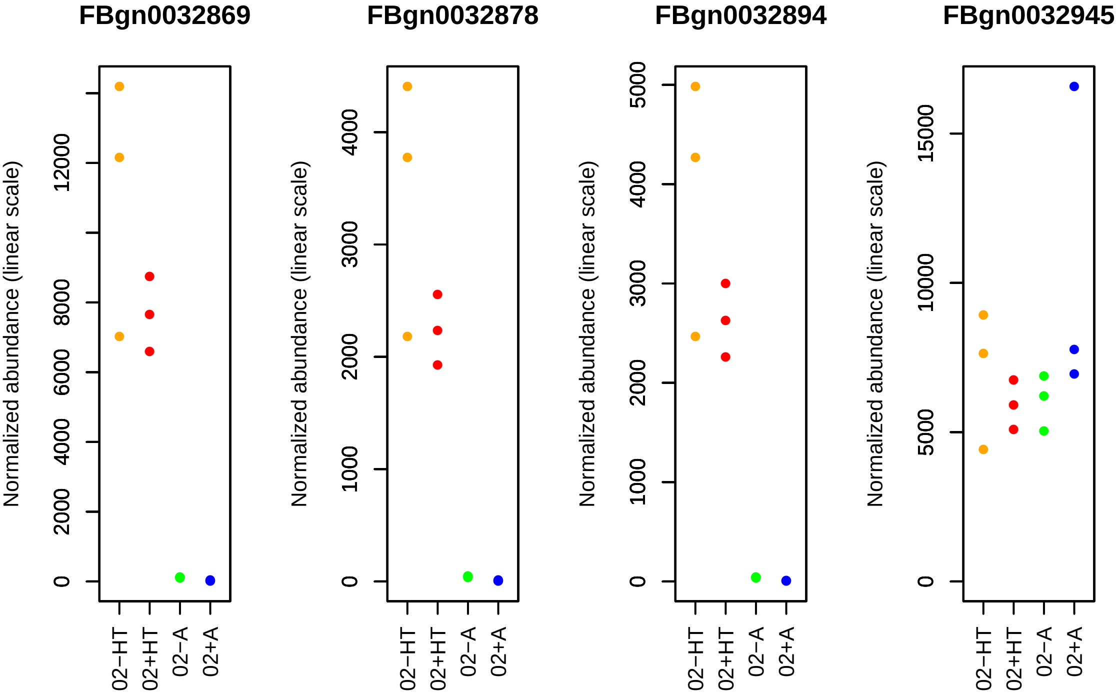

FBgn0032869
FBgn0032878
FBgn0032894
FBgn0032945
Normalized abundance (linear scale)
0
2000
4000
6000
8000
12000
1000
3000
5000
10000
15000
02−HT
02+HT
02−A
02+A

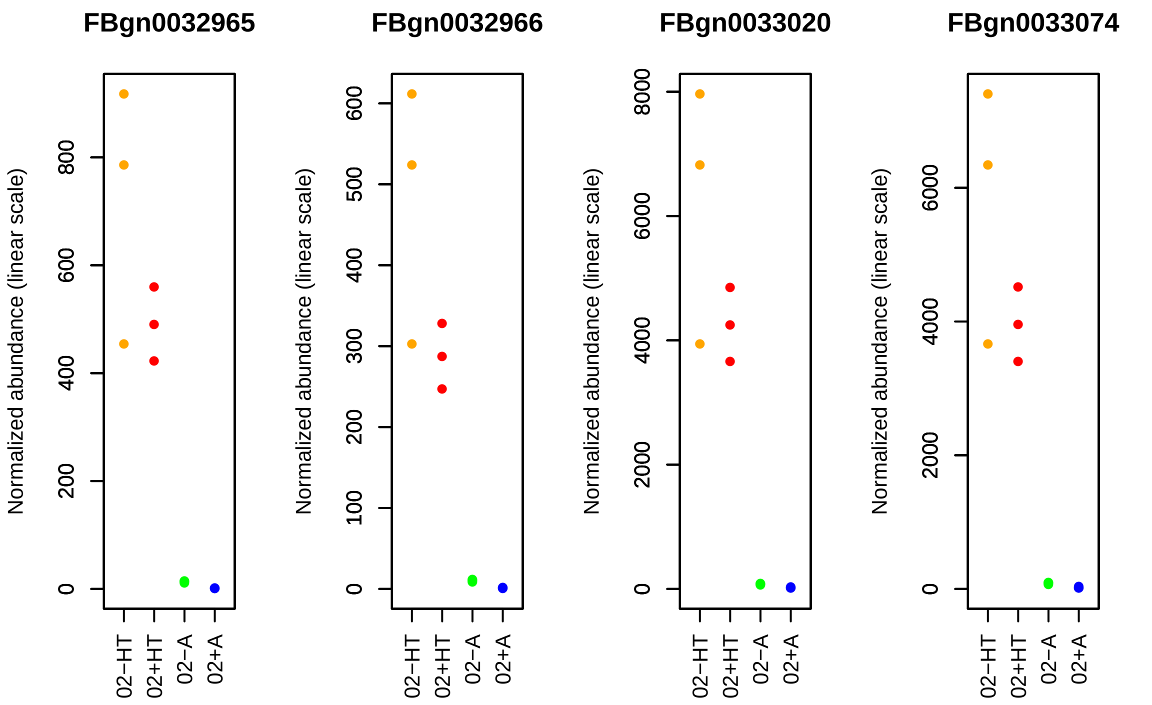
FBgn0032965
FBgn0032966
FBgn0033020
FBgn0033074
Normalized abundance (linear scale)
02−HT
02+HT
02−A
02+A
0
200
400
600
800
100
300
500
2000
4000
6000
8000

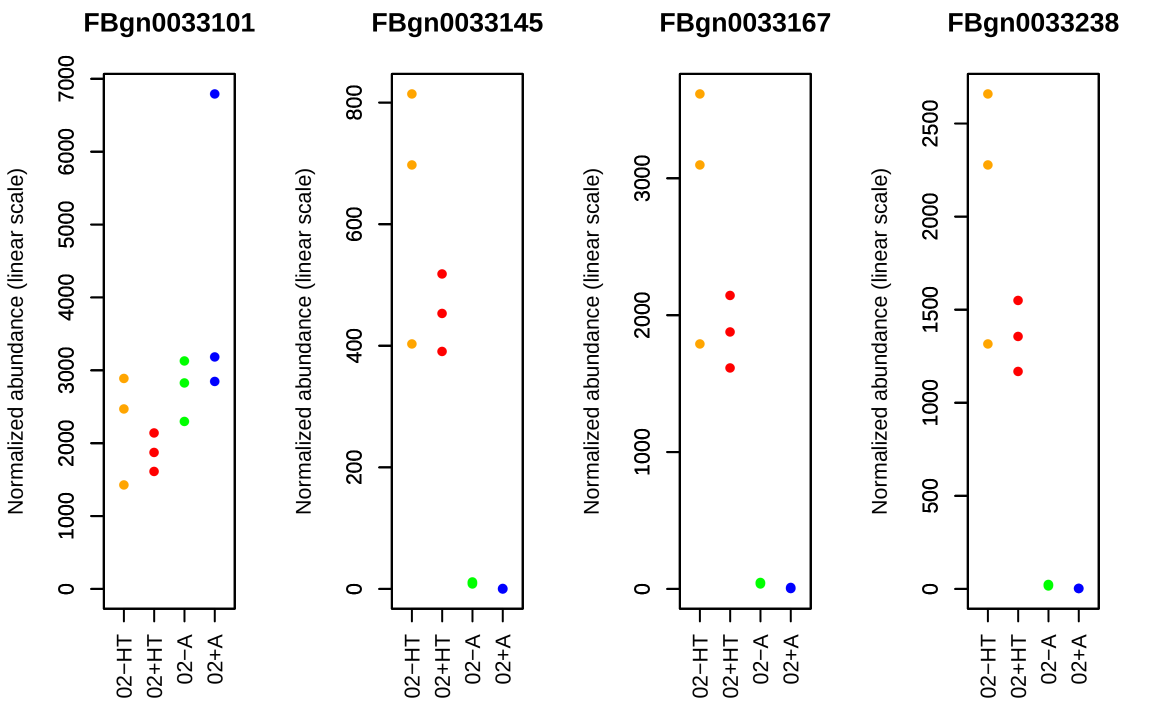
FBgn0033101
FBgn0033145
FBgn0033167
FBgn0033238
Normalized abundance (linear scale)
0
1000
2000
3000
4000
5000
6000
7000
200
400
600
800
500
1500
2500
02−HT
02+HT
02−A
02+A

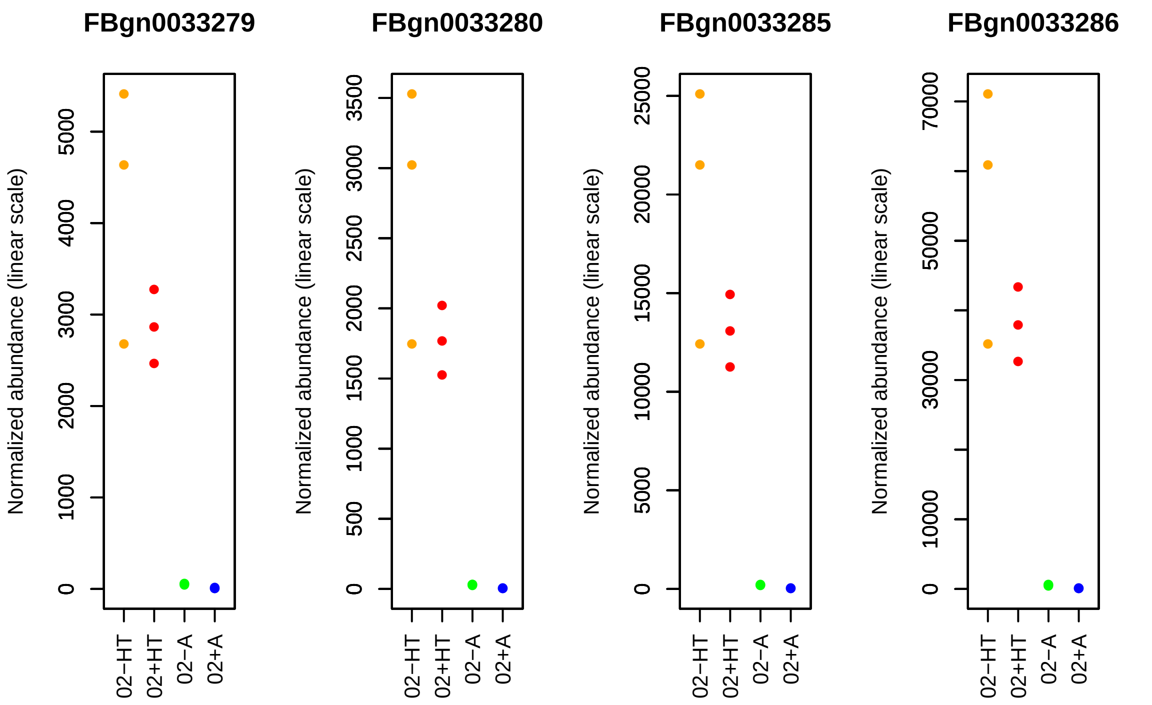

FBgn0033279
FBgn0033280
FBgn0033285
FBgn0033286
Normalized abundance (linear scale)
0
1000
2000
3000
4000
5000
500
1500
2500
3500
5000
10000
15000
20000
25000
30000
50000
70000
02−HT
02+HT
02−A
02+A

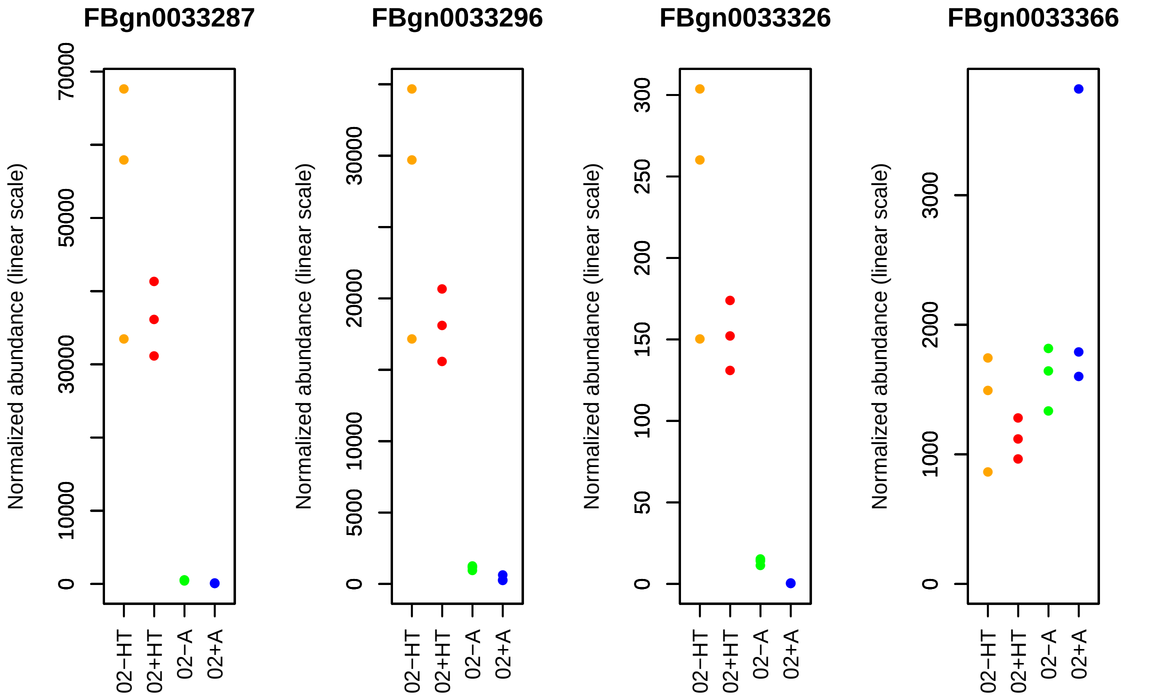

FBgn0033287
FBgn0033296
FBgn0033326
FBgn0033366
Normalized abundance (linear scale)
0
10000
30000
50000
70000
5000
20000
30000
50
100
150
200
250
300
1000
2000
3000
02−HT
02+HT
02−A
02+A

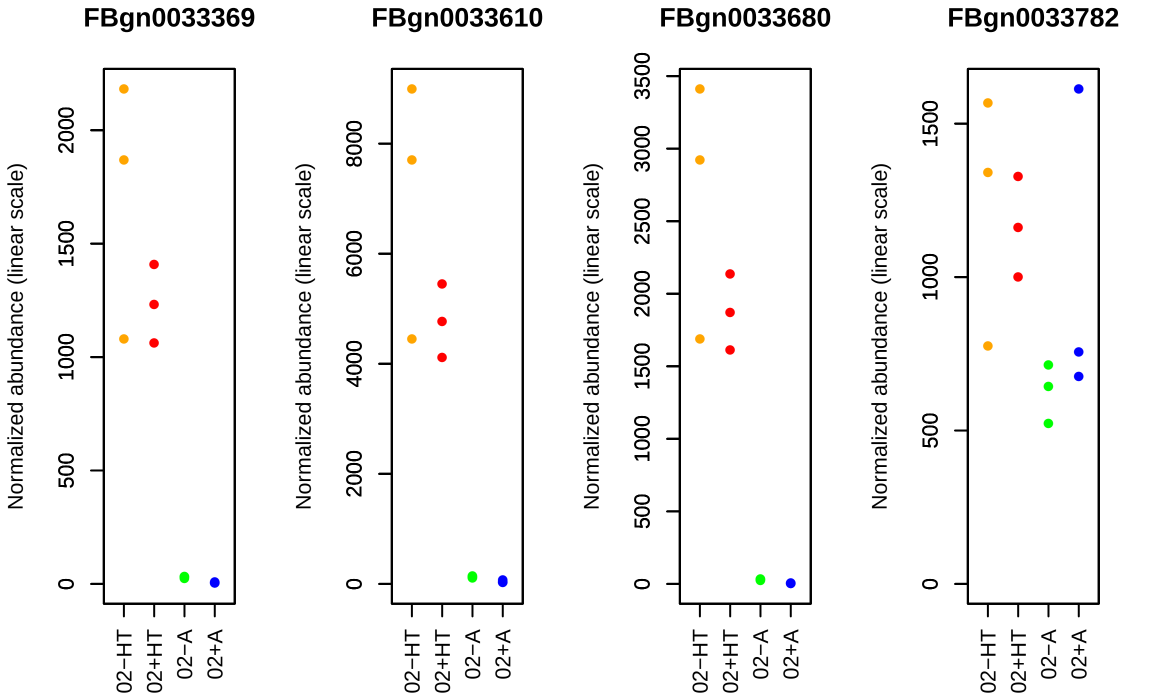

FBgn0033369
FBgn0033610
FBgn0033680
FBgn0033782
Normalized abundance (linear scale)
0
500
1000
1500
2000
2000
4000
6000
8000
2500
3000
3500
02−HT
02+HT
02−A
02+A

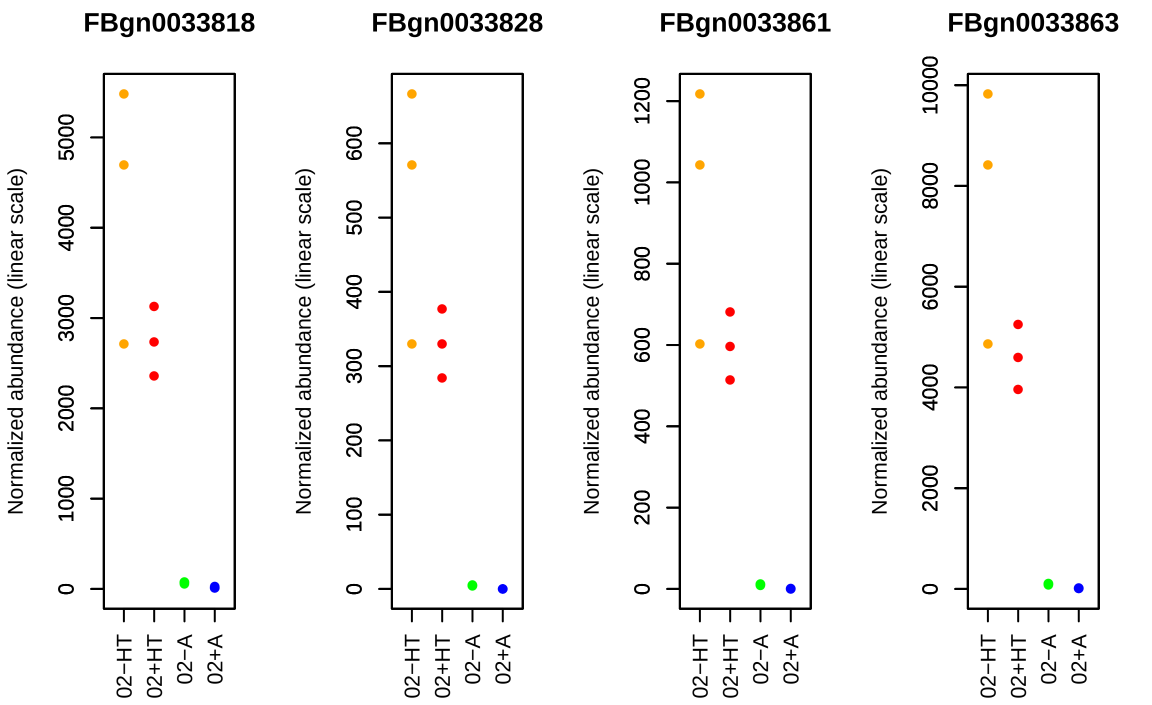

FBgn0033818
FBgn0033828
FBgn0033861
FBgn0033863
Normalized abundance (linear scale)
0
1000
2000
3000
4000
5000
100
200
300
400
500
600
800
1000
1200
6000
8000
10000
02−HT
02+HT
02−A
02+A

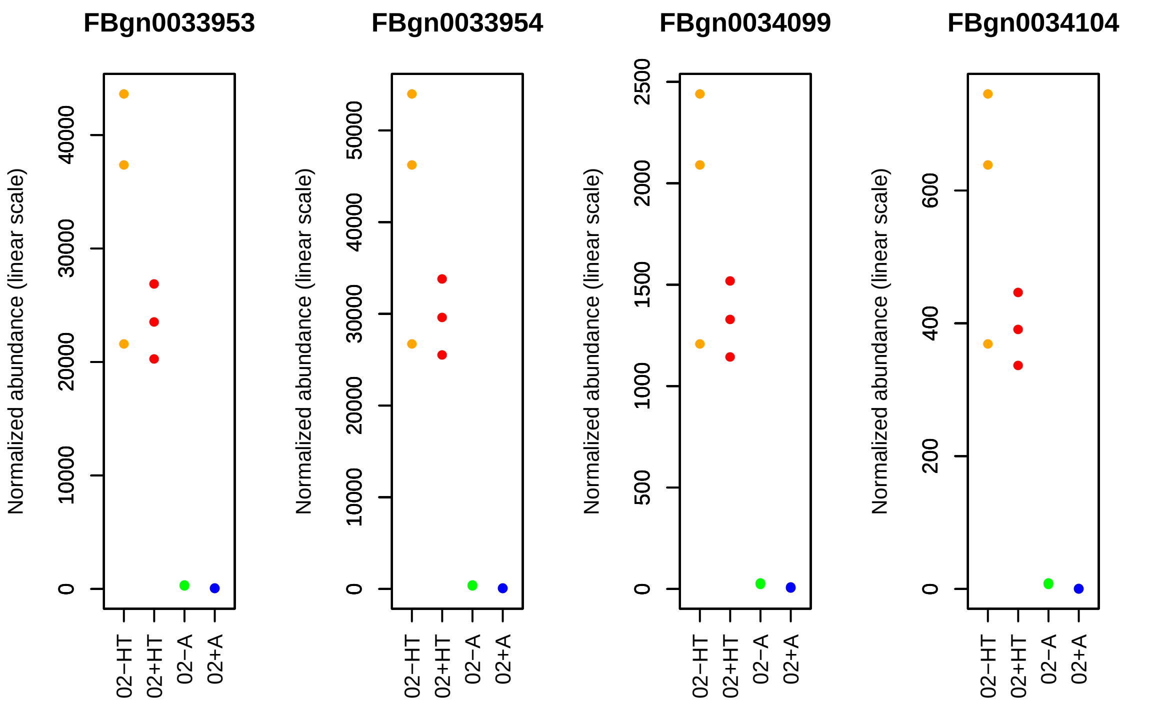

FBgn0033953
FBgn0033954
FBgn0034099
FBgn0034104
Normalized abundance (linear scale)
0
10000
20000
30000
40000
50000
500
1000
1500
2000
2500
200
400
600
02−HT
02+HT
02−A
02+A

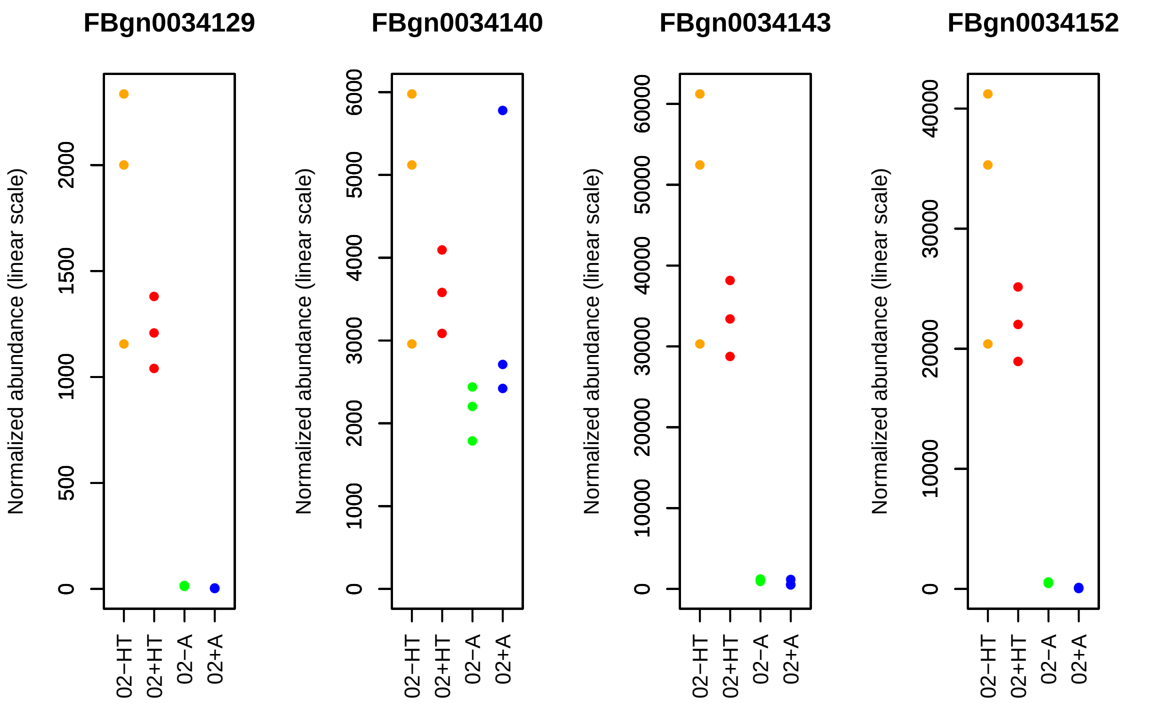

FBgn0034129
FBgn0034140
FBgn0034143
FBgn0034152
Normalized abundance (linear scale)
0
500
1000
1500
2000
0
1000
2000
3000
4000
5000
6000
0
10000
20000
30000
40000
50000
60000
0
10000
20000
30000
40000
02−HT
02+HT
02−A
02+A

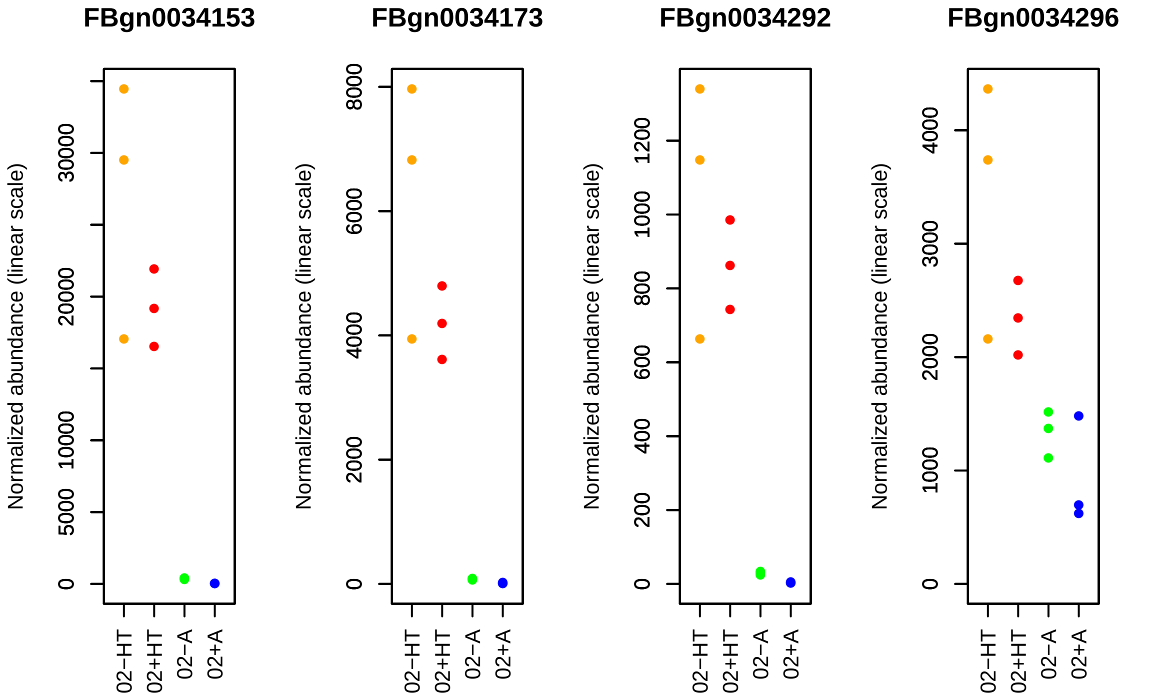
FBgn0034153
FBgn0034173
FBgn0034292
FBgn0034296
Normalized abundance (linear scale)
0
5000
10000
20000
30000
2000
4000
6000
8000
200
400
600
800
1000
1200
1000
2000
3000
4000
02−HT
02+HT
02−A
02+A

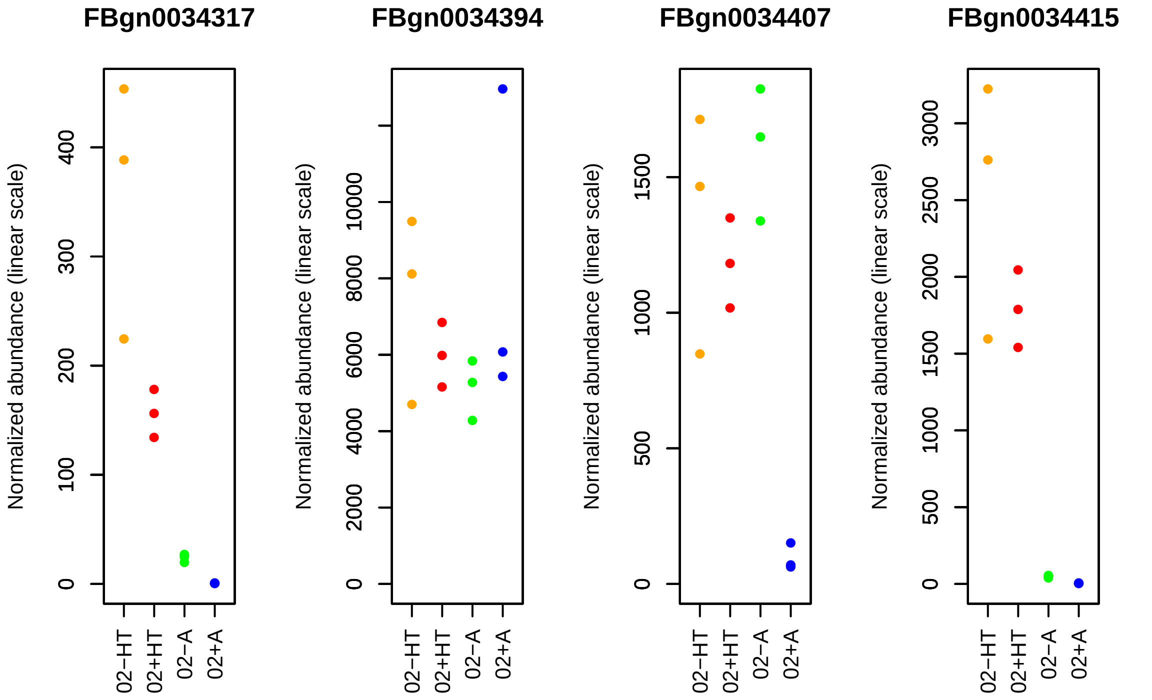

FBgn0034317
FBgn0034394
FBgn0034407
FBgn0034415
Normalized abundance (linear scale)
0
100
200
300
400
2000
4000
6000
8000
10000
500
1000
1500
2500
3000
02−HT
02+HT
02−A
02+A

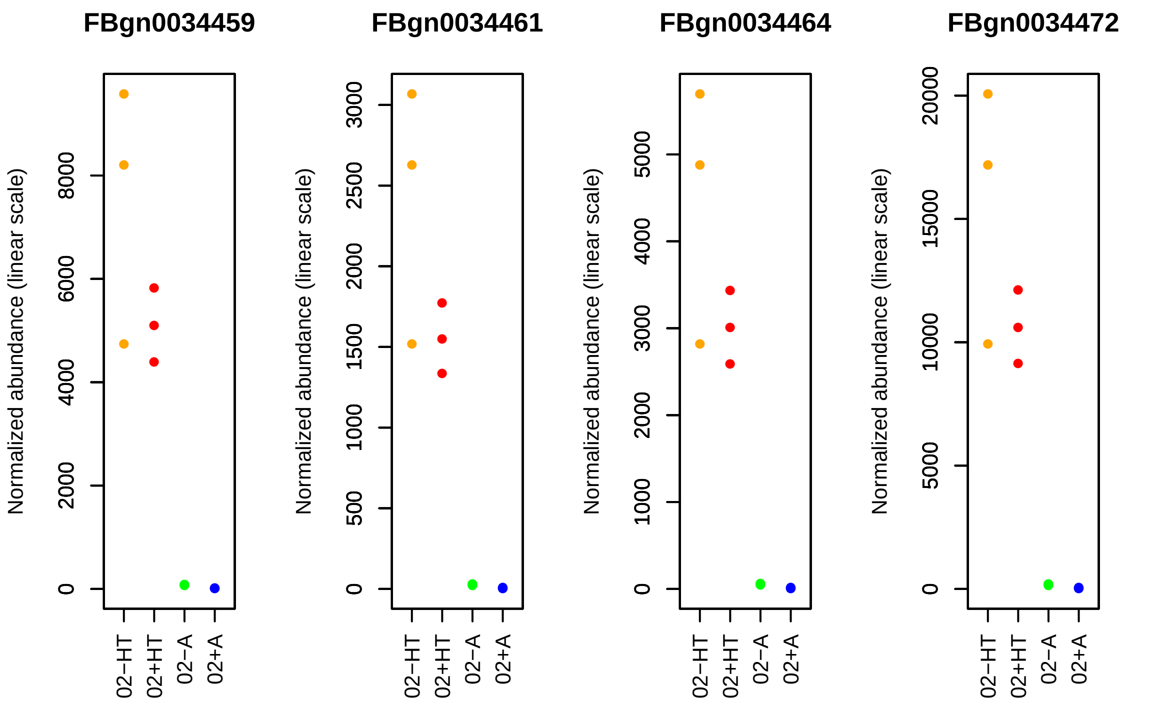
FBgn0034459
FBgn0034461
FBgn0034464
FBgn0034472
Normalized abundance (linear scale)
02−HT
02+HT
02−A
02+A

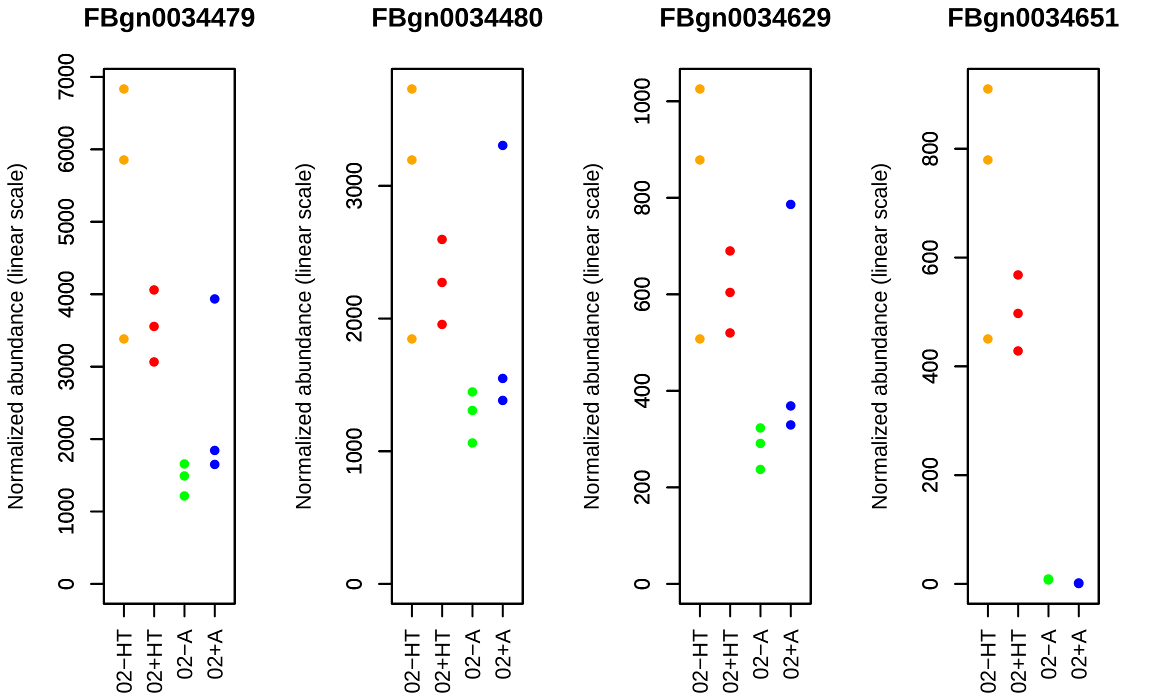

FBgn0034479
FBgn0034480
FBgn0034629
FBgn0034651
Normalized abundance (linear scale)
0
1000
2000
3000
4000
5000
6000
7000
800
600
400
200
02−HT
02+HT
02−A
02+A

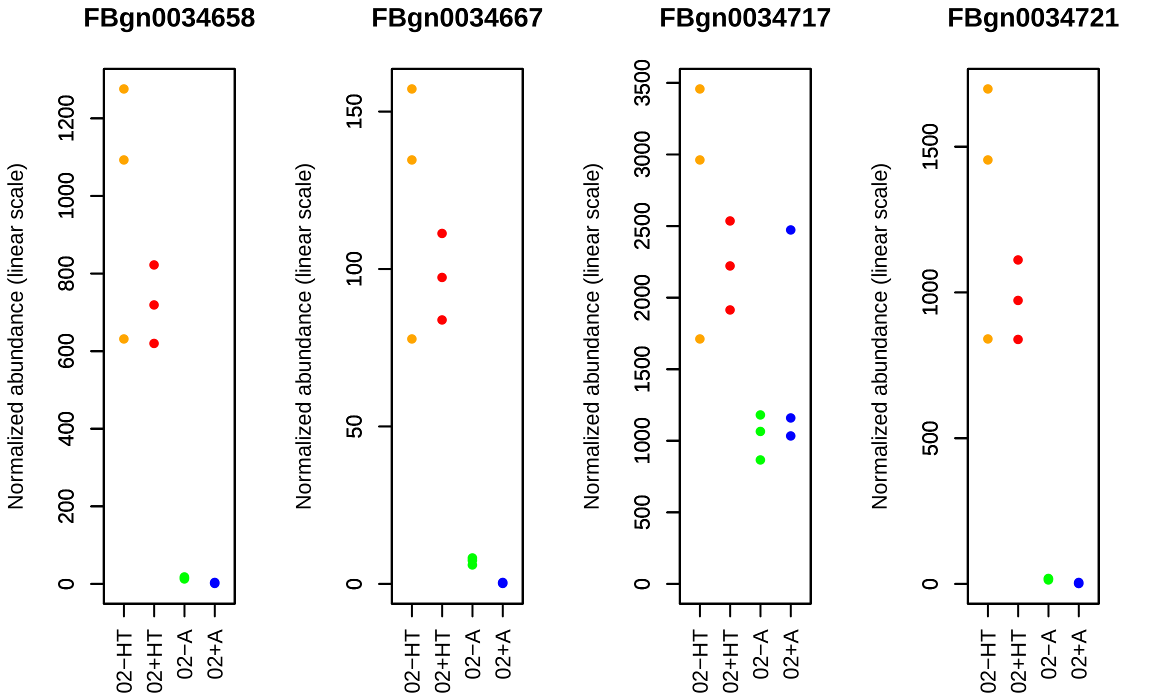
FBgn0034658
FBgn0034667
FBgn0034717
FBgn0034721
Normalized abundance (linear scale)
0
200
400
600
800
1000
1200
50
100
150
500
1500
2000
2500
3000
3500
02−HT
02+HT
02−A
02+A

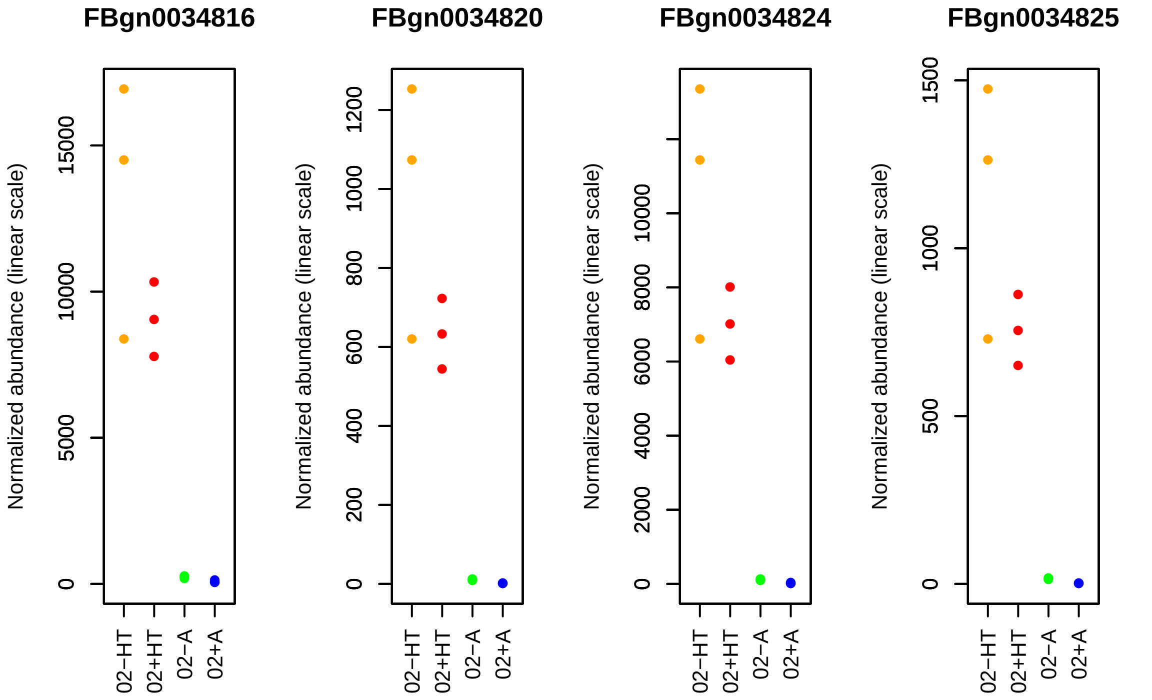
FBgn0034816
FBgn0034820
FBgn0034824
FBgn0034825
Normalized abundance (linear scale)
0
5000
10000
15000
0
200
400
600
800
1000
1200
0
2000
4000
6000
8000
10000
0
500
1000
1500
02−HT
02+HT
02−A
02+A

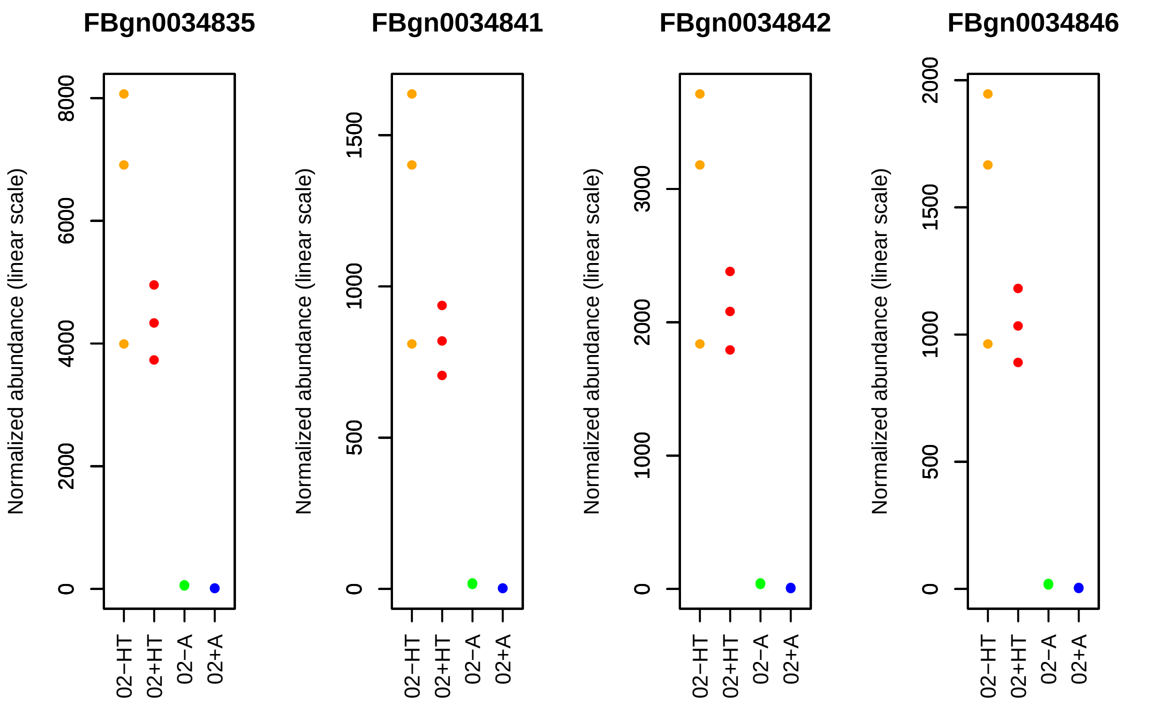

FBgn0034835
FBgn0034841
FBgn0034842
FBgn0034846
Normalized abundance (linear scale)
0
2000
4000
6000
8000
500
1000
1500
3000
2000
02−HT
02+HT
02−A
02+A

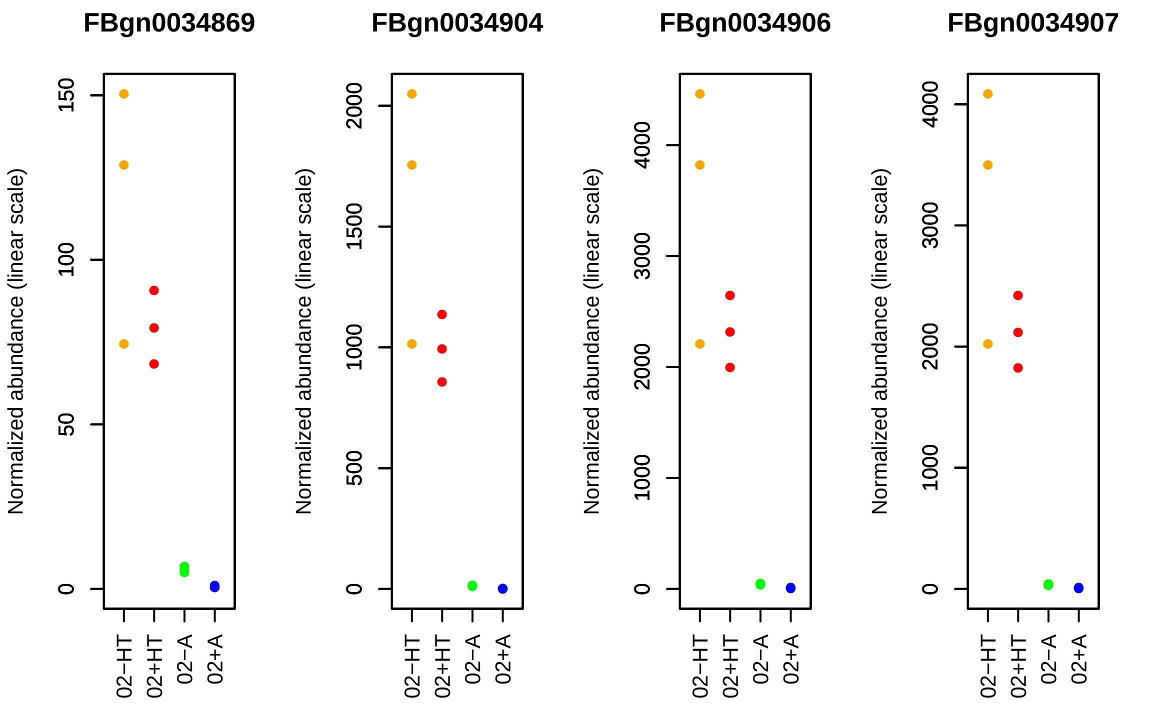

FBgn0034869
FBgn0034904
FBgn0034906
FBgn0034907
Normalized abundance (linear scale)
0
50
100
150
500
1000
1500
2000
3000
4000
02−HT
02+HT
02−A
02+A

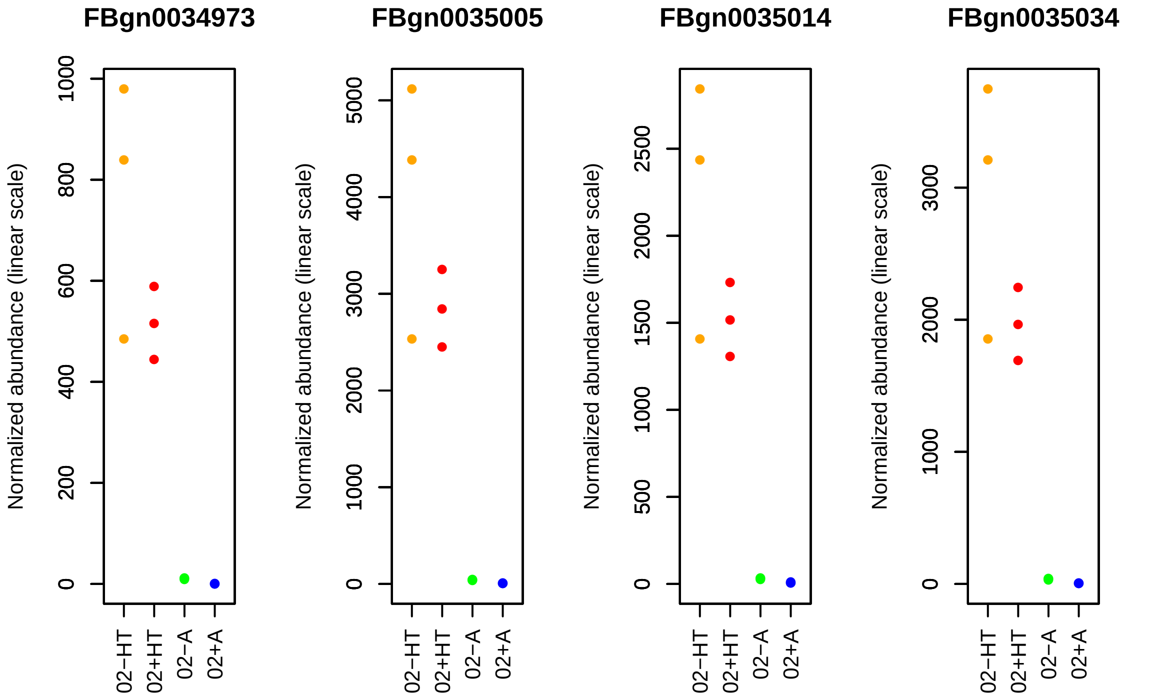
FBgn0034973
FBgn0035005
FBgn0035014
FBgn0035034
Normalized abundance (linear scale)
0
200
400
600
800
1000
0
1000
2000
3000
4000
5000
0
500
1000
1500
2000
2500
0
1000
2000
3000
02−HT
02+HT
02−A
02+A

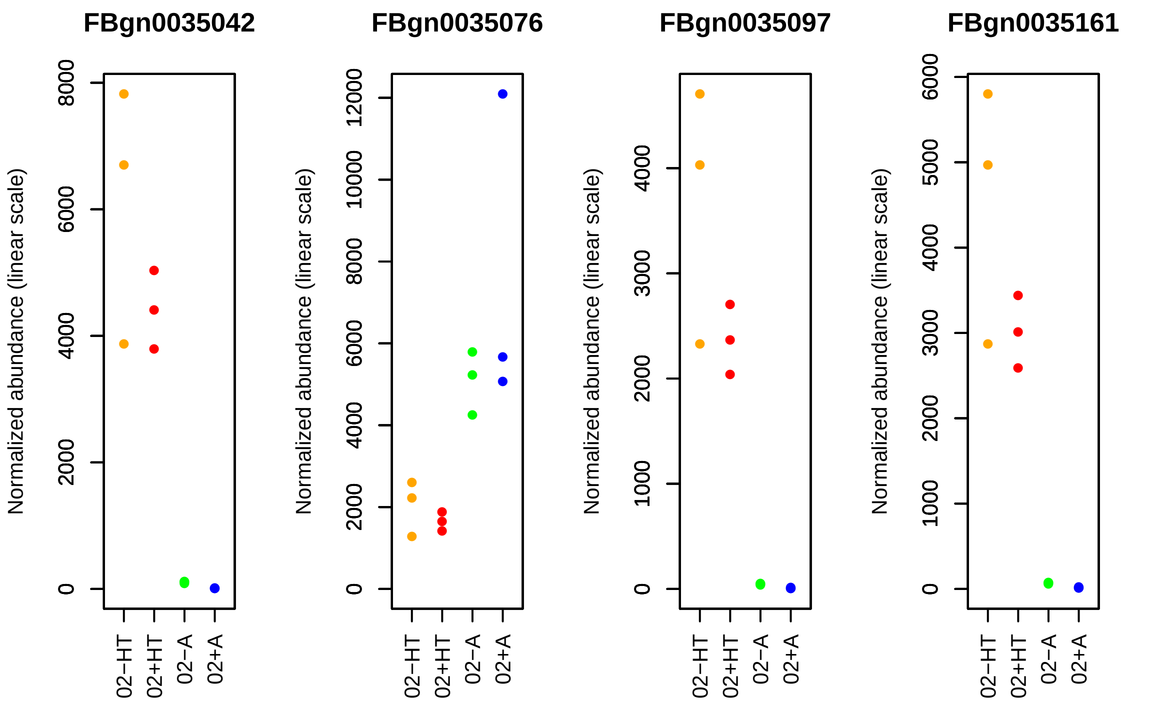

FBgn0035042
FBgn0035076
FBgn0035097
FBgn0035161
Normalized abundance (linear scale)
02−HT
02+HT
02−A
02+A

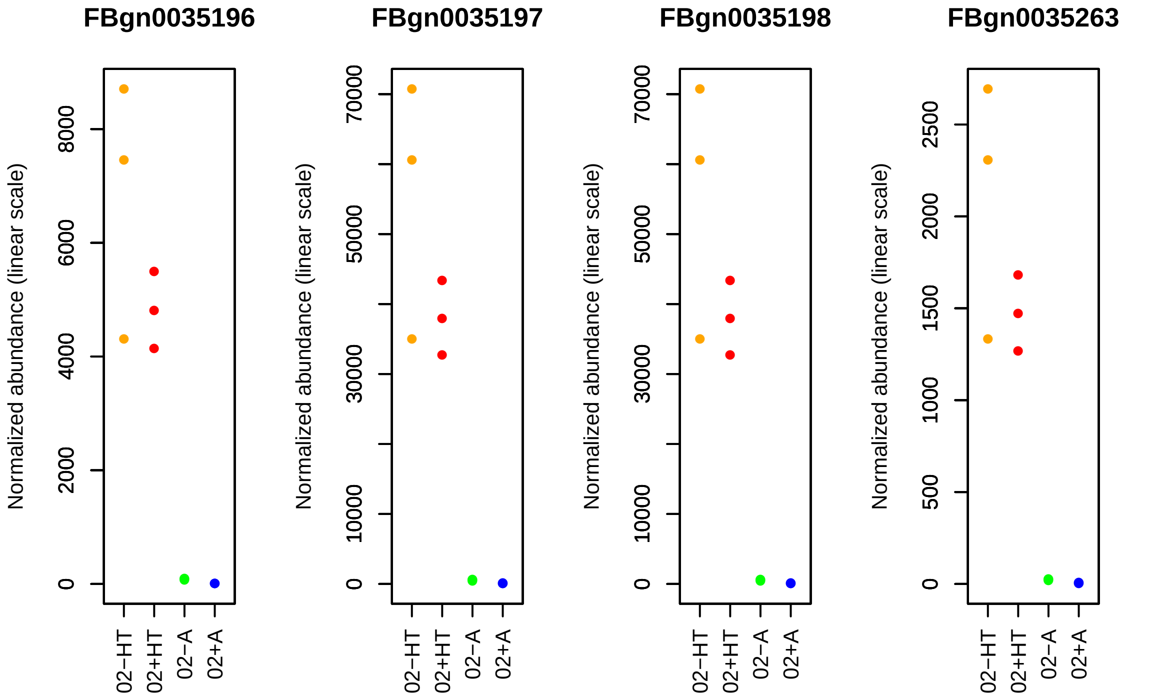

FBgn0035196
FBgn0035197
FBgn0035198
FBgn0035263
Normalized abundance (linear scale)
0
2000
4000
6000
8000
10000
30000
50000
70000
500
1000
1500
2000
2500
02−HT
02+HT
02−A
02+A

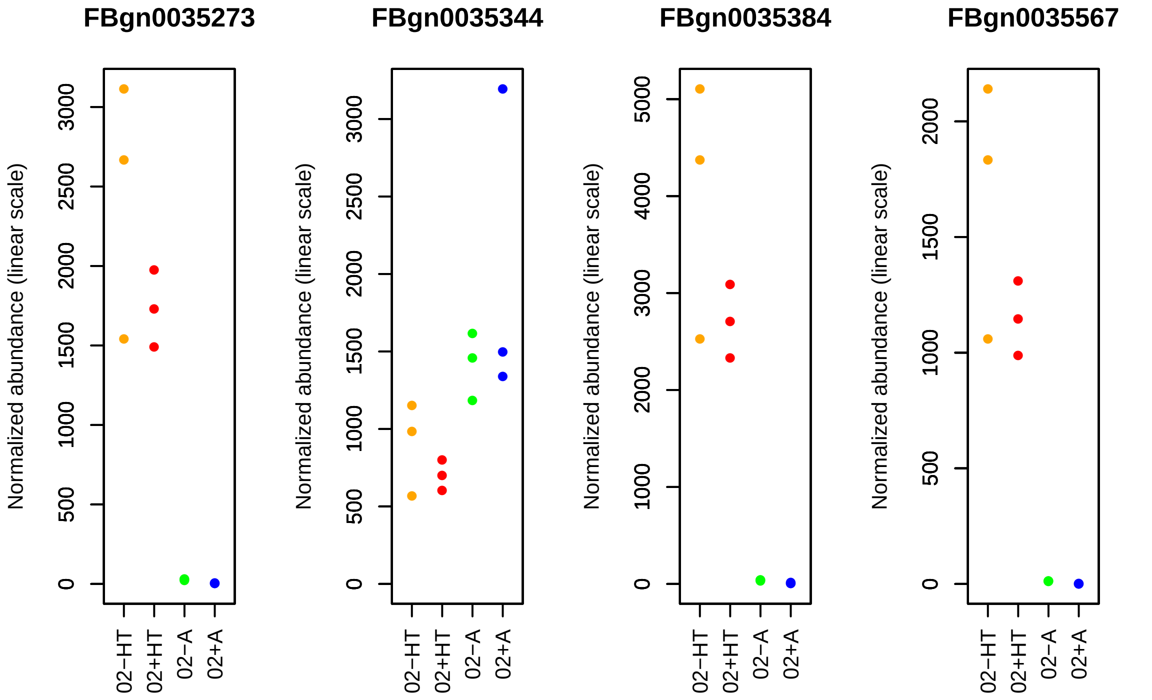

FBgn0035273
FBgn0035344
FBgn0035384
FBgn0035567
Normalized abundance (linear scale)
0
500
1000
1500
2000
2500
3000
4000
5000
02−HT
02+HT
02−A
02+A

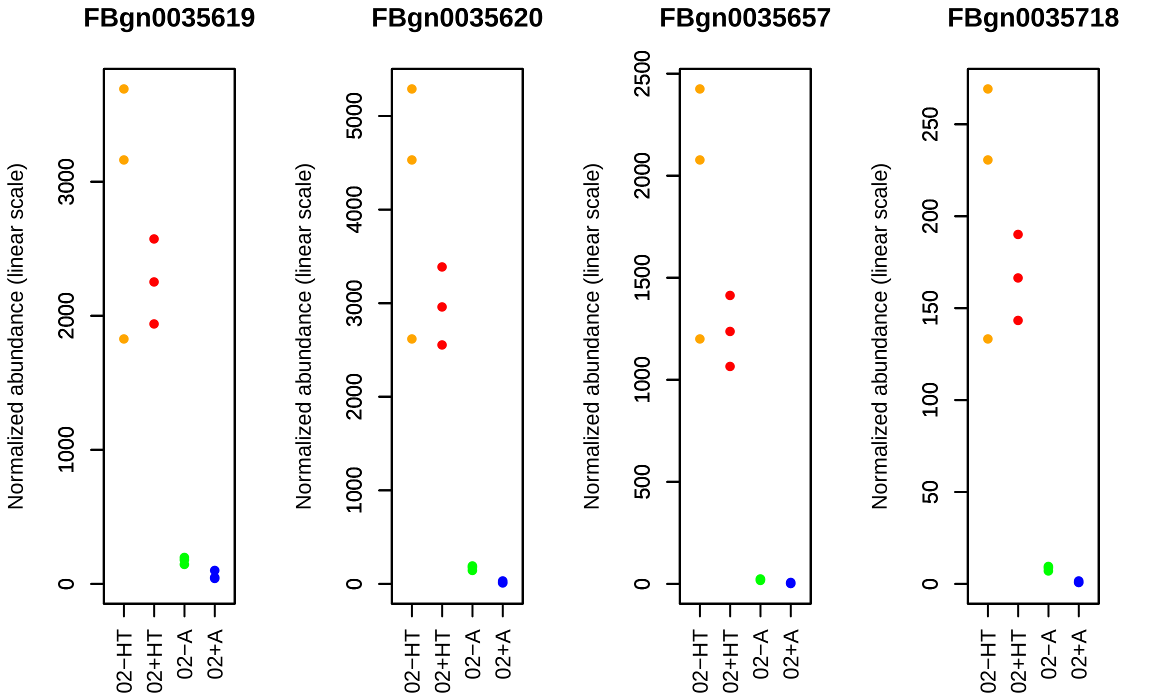
FBgn0035619
FBgn0035620
FBgn0035657
FBgn0035718
Normalized abundance (linear scale)
0
1000
2000
3000
4000
5000
500
1500
2500
50
100
150
200
250
02−HT
02+HT
02−A
02+A

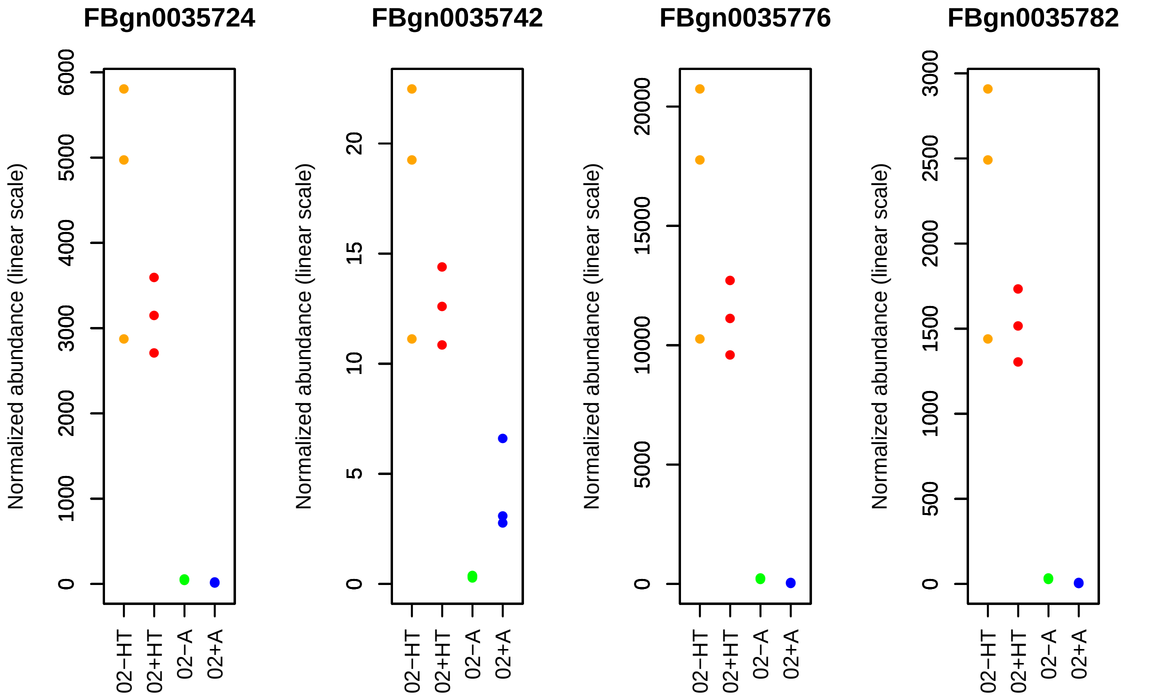

FBgn0035724
FBgn0035742
FBgn0035776
FBgn0035782
Normalized abundance (linear scale)
0
1000
2000
3000
4000
5000
6000
0
5
10
15
20
0
5000
10000
15000
20000
0
500
1000
1500
2000
2500
3000
02−HT
02+HT
02−A
02+A

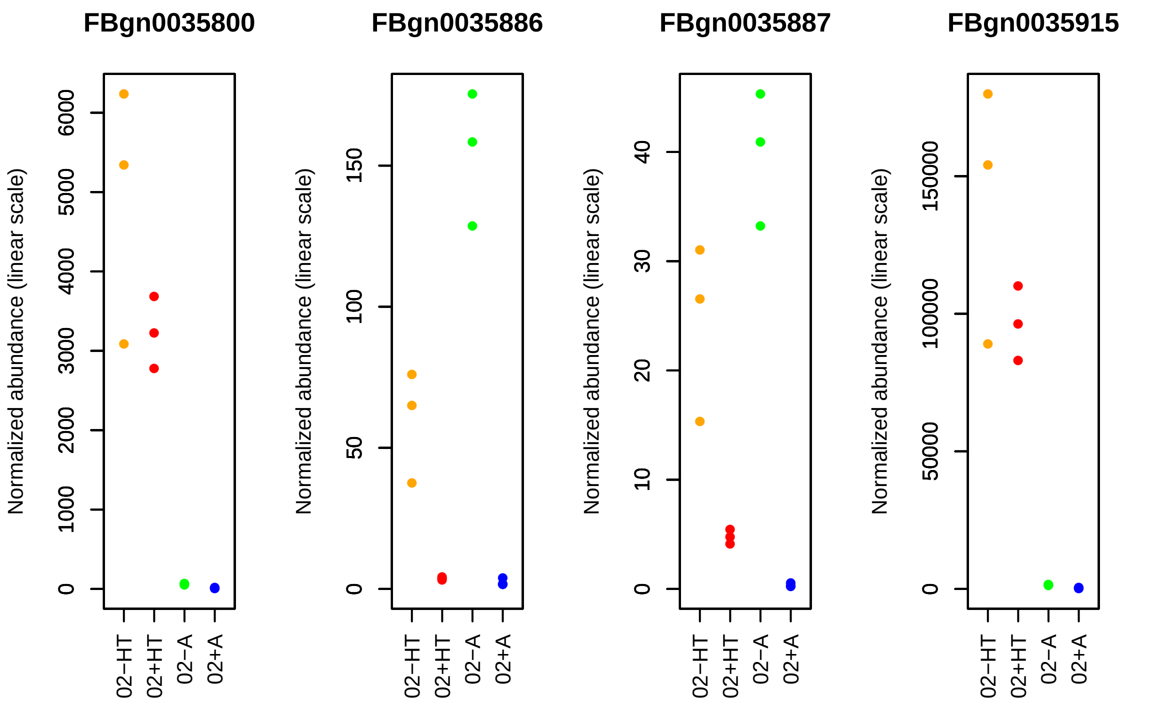

FBgn0035800
FBgn0035886
FBgn0035887
FBgn0035915
Normalized abundance (linear scale)
0
1000
2000
3000
4000
5000
6000
50
100
150
10
20
30
40
50000
100000
150000
02−HT
02+HT
02−A
02+A

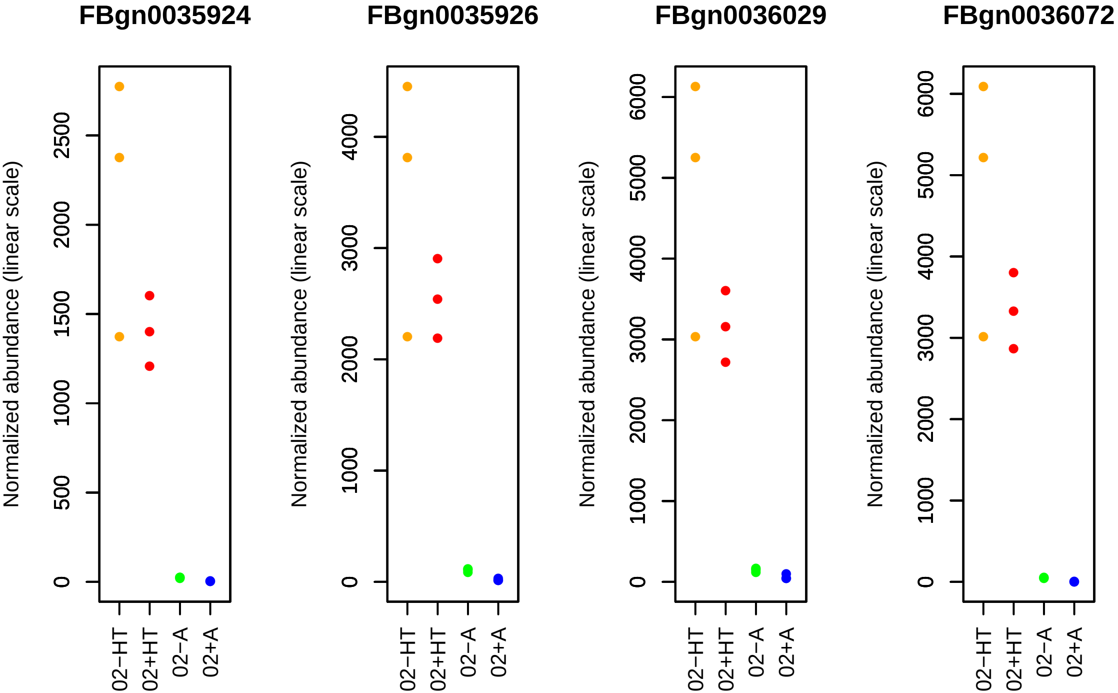
FBgn0035924
FBgn0035926
FBgn0036029
FBgn0036072
Normalized abundance (linear scale)
0
500
1000
1500
2000
2500
2000
3000
4000
5000
6000
02−HT
02+HT
02−A
02+A

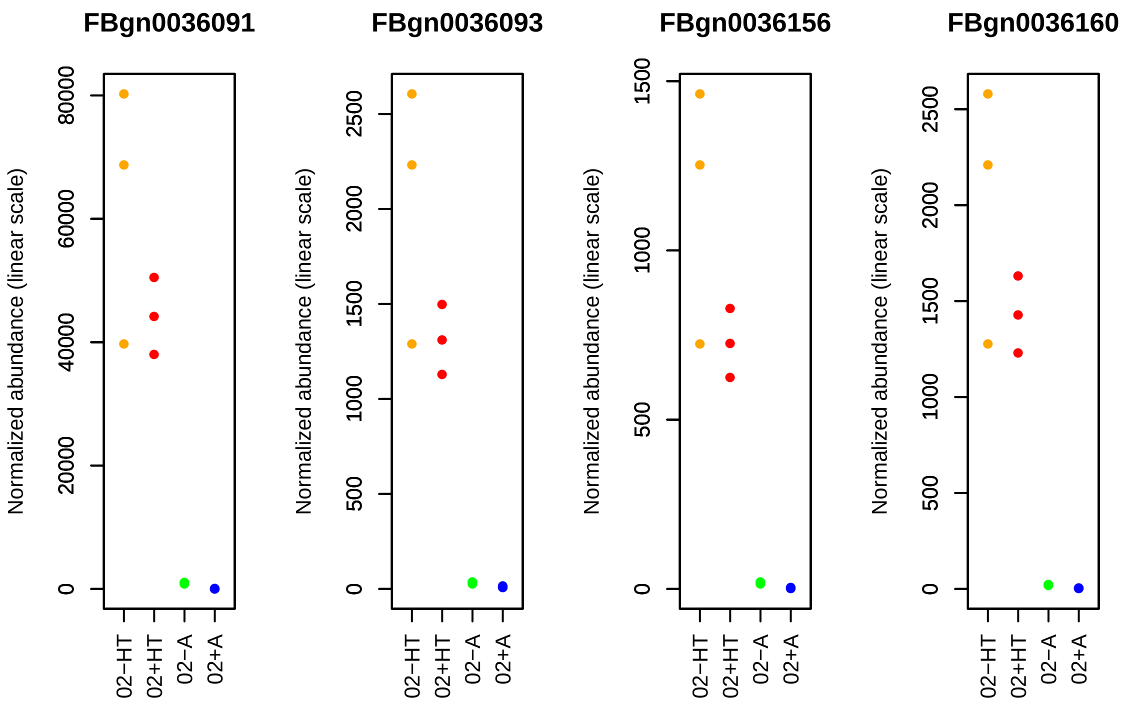

FBgn0036091
FBgn0036093
FBgn0036156
FBgn0036160
Normalized abundance (linear scale)
0
20000
40000
60000
80000
500
1000
1500
2000
2500
02−HT
02+HT
02−A
02+A

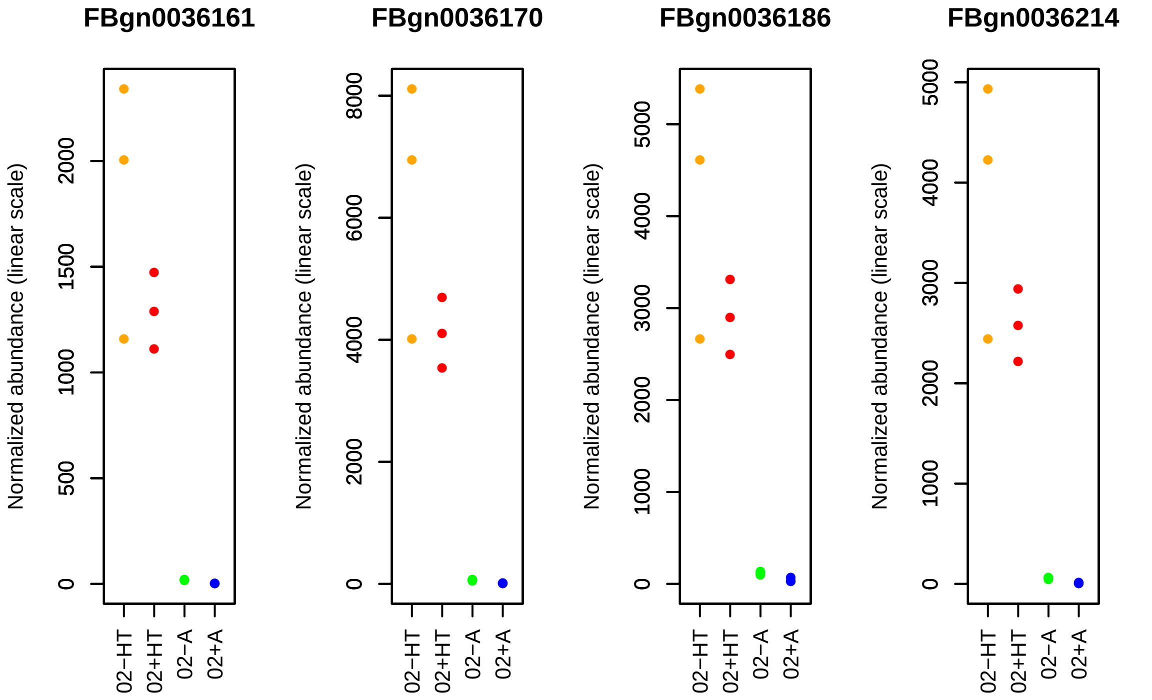

FBgn0036161
FBgn0036170
FBgn0036186
FBgn0036214
Normalized abundance (linear scale)
0
500
1000
1500
2000
2000
4000
6000
8000
3000
5000
02−HT
02+HT
02−A
02+A

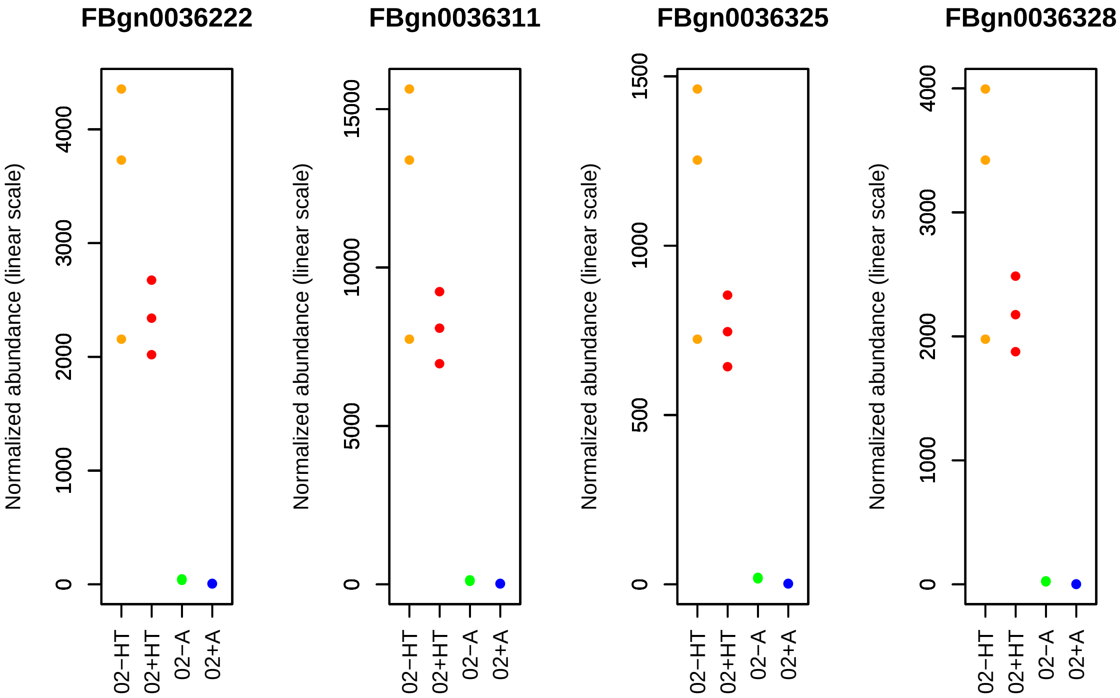
FBgn0036222
FBgn0036311
FBgn0036325
FBgn0036328
Normalized abundance (linear scale)
0
1000
2000
3000
4000
5000
10000
15000
500
1500
02−HT
02+HT
02−A
02+A

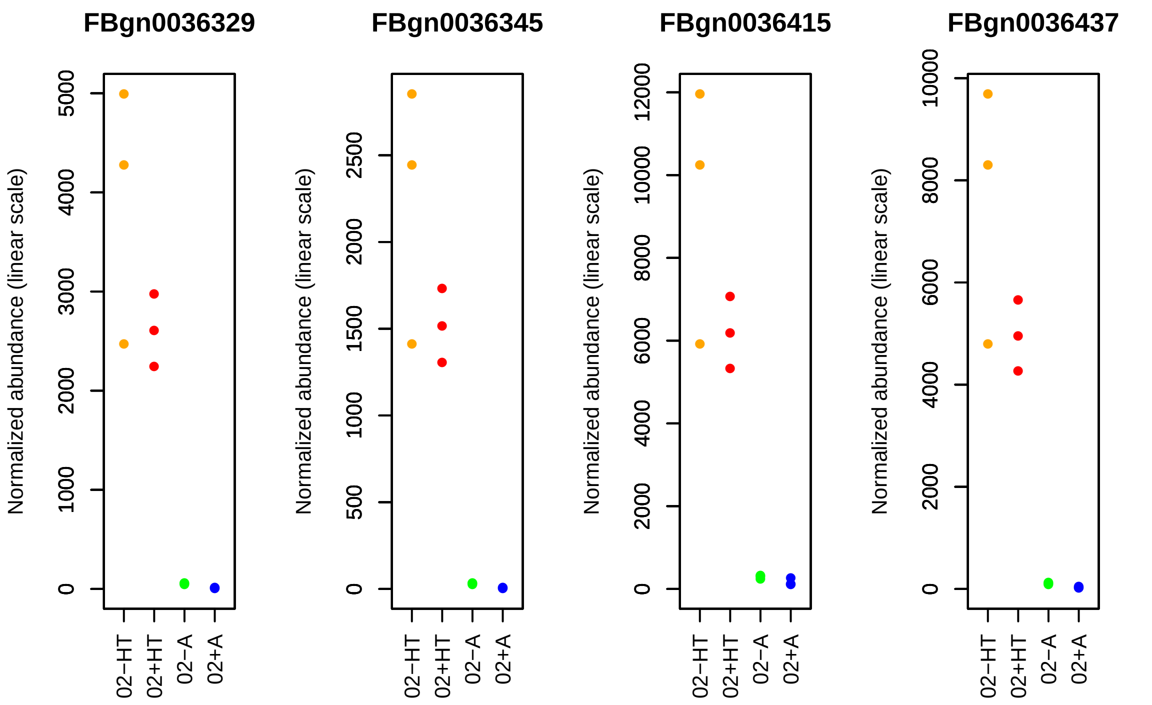

FBgn0036329
FBgn0036345
FBgn0036415
FBgn0036437
Normalized abundance (linear scale)
0
1000
2000
3000
4000
5000
500
1500
2500
6000
8000
10000
12000
02−HT
02+HT
02−A
02+A

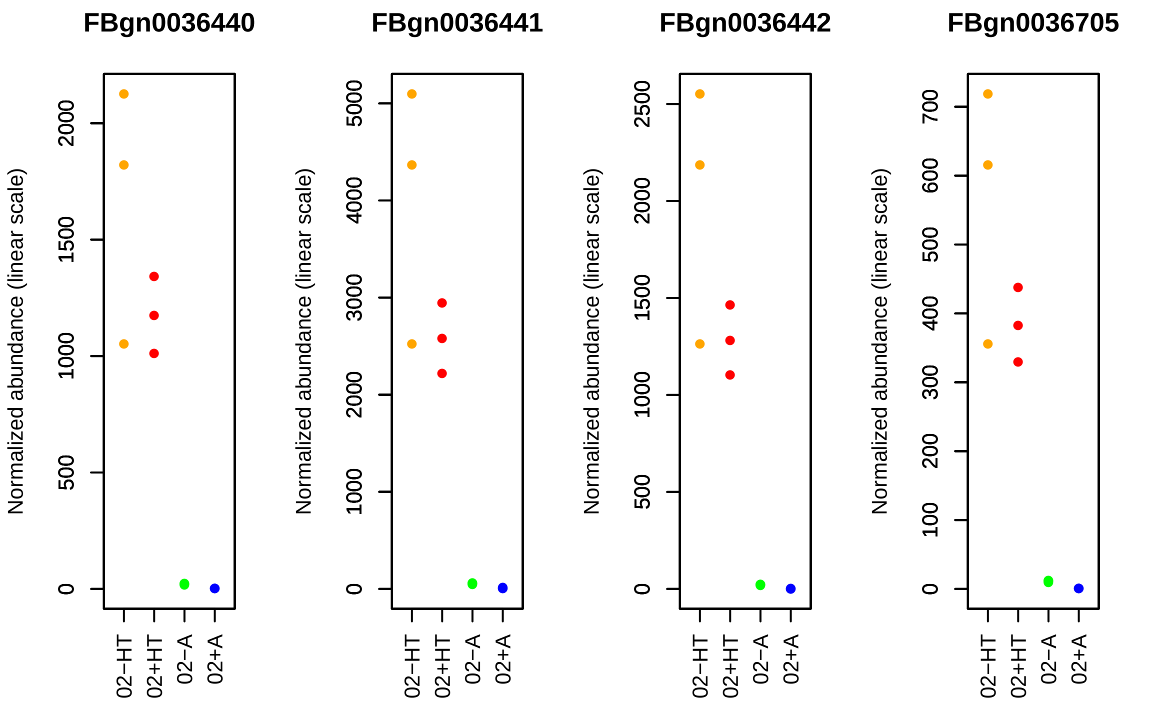

FBgn0036440
FBgn0036441
FBgn0036442
FBgn0036705
Normalized abundance (linear scale)
0
500
1000
1500
2000
2500
3000
4000
5000
100
200
300
400
500
600
700
02−HT
02+HT
02−A
02+A

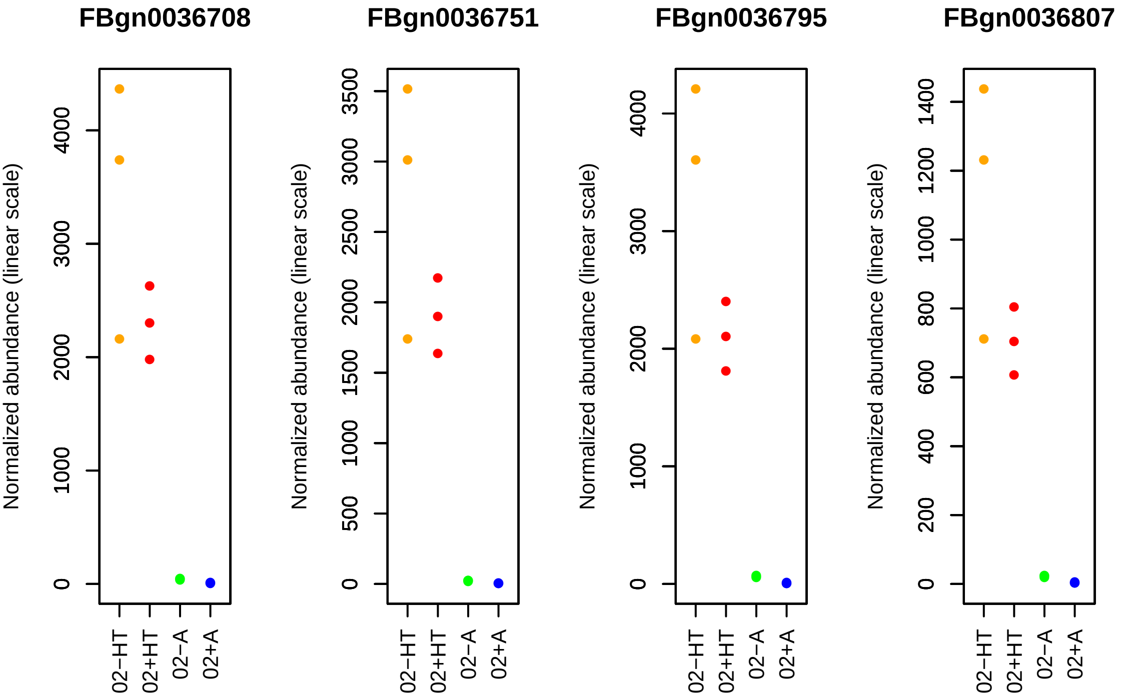
FBgn0036708
FBgn0036751
FBgn0036795
FBgn0036807
Normalized abundance (linear scale)
0
1000
2000
3000
4000
500
1500
2500
3500
200
400
600
800
1200
1400
02−HT
02+HT
02−A
02+A

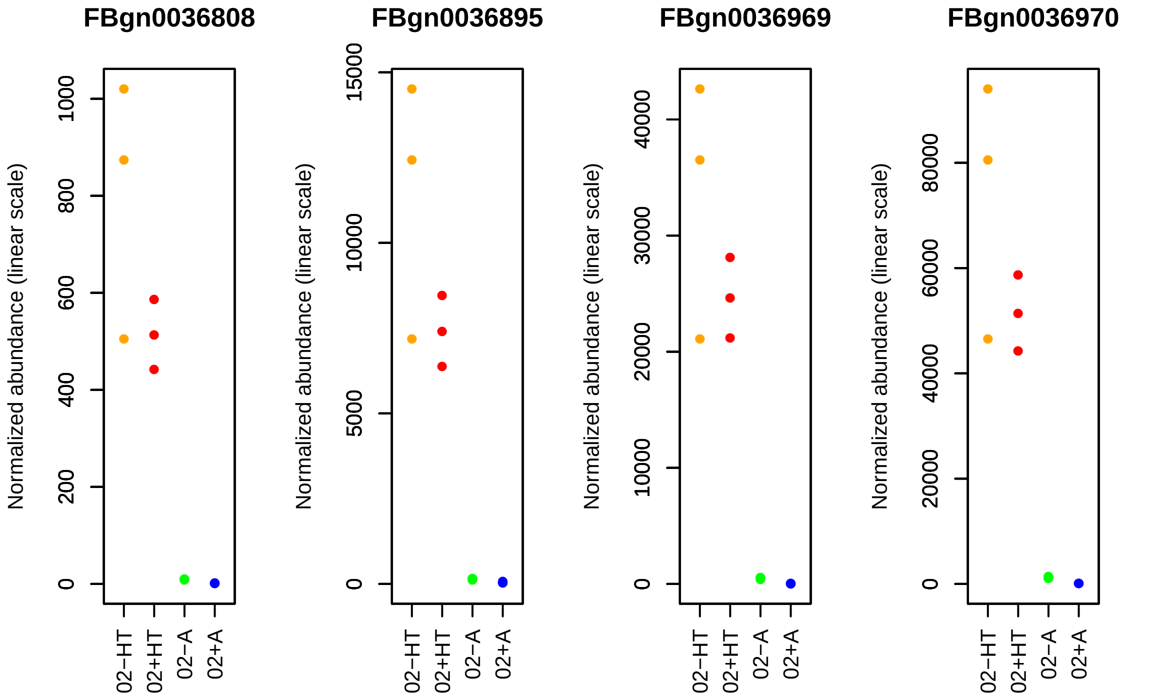

FBgn0036808
FBgn0036895
FBgn0036969
FBgn0036970
Normalized abundance (linear scale)
0
200
400
600
800
1000
5000
10000
15000
20000
30000
40000
60000
80000
02−HT
02+HT
02−A
02+A

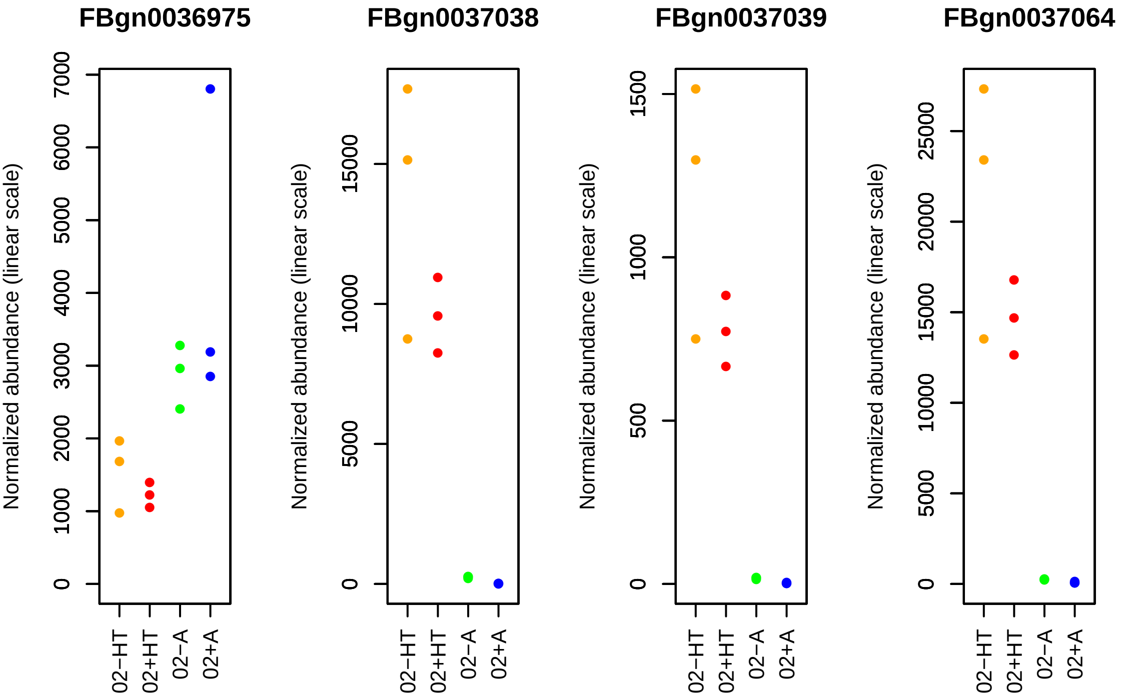
FBgn0036975
FBgn0037038
FBgn0037039
FBgn0037064
Normalized abundance (linear scale)
0
1000
2000
3000
4000
5000
6000
7000
5000
10000
15000
500
1500
20000
25000
02−HT
02+HT
02−A
02+A

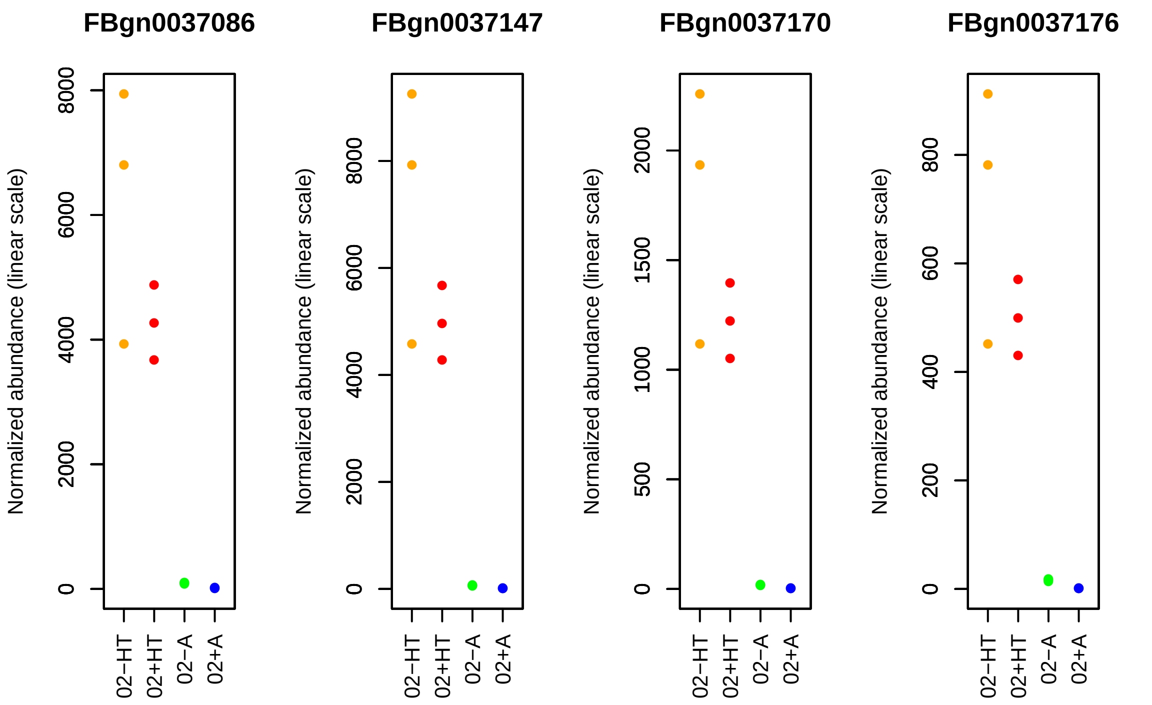

FBgn0037086
FBgn0037147
FBgn0037170
FBgn0037176
Normalized abundance (linear scale)
0
2000
4000
6000
8000
500
1000
1500
200
400
600
800
02−HT
02+HT
02−A
02+A

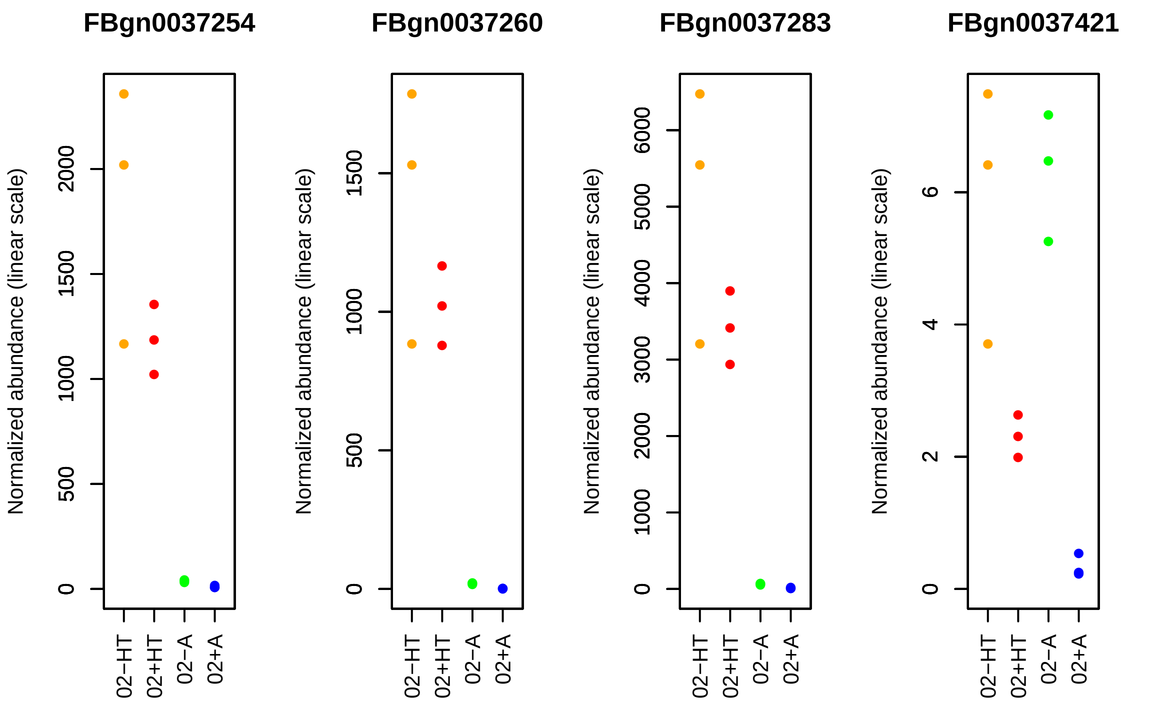

FBgn0037254
FBgn0037260
FBgn0037283
FBgn0037421
Normalized abundance (linear scale)
0
500
1000
1500
2000
4000
5000
6000
2
4
6
02−HT
02+HT
02−A
02+A

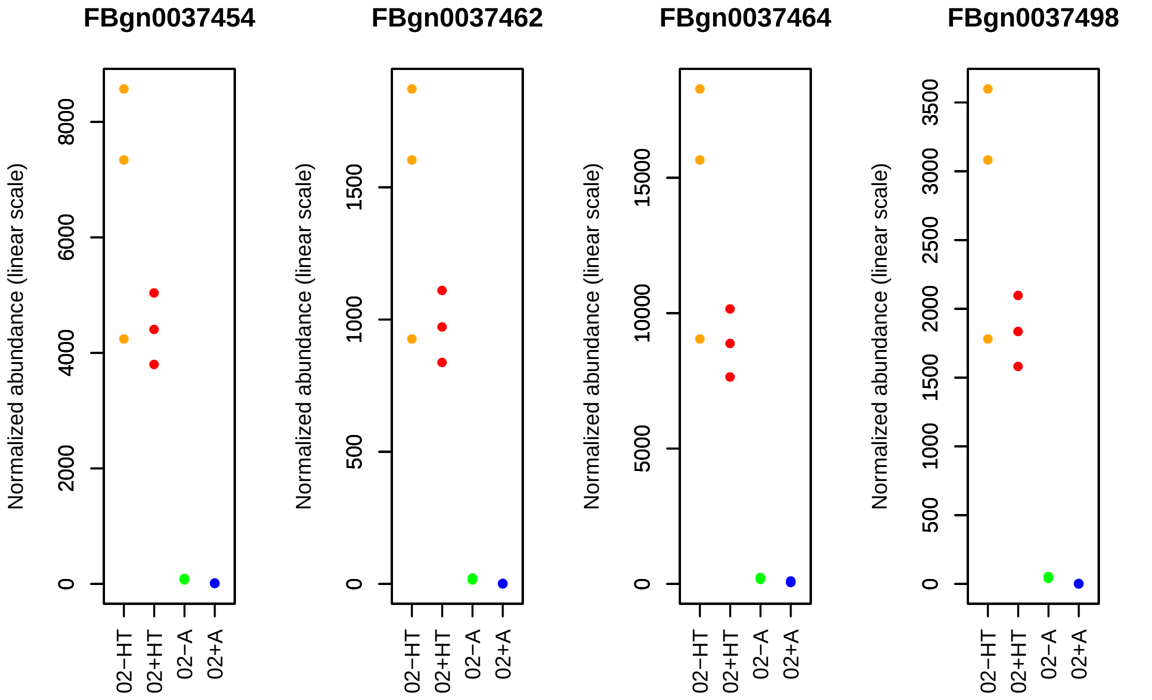

FBgn0037454
FBgn0037462
FBgn0037464
FBgn0037498
Normalized abundance (linear scale)
0
2000
4000
6000
8000
500
1000
1500
5000
10000
15000
2500
3000
3500
02−HT
02+HT
02−A
02+A

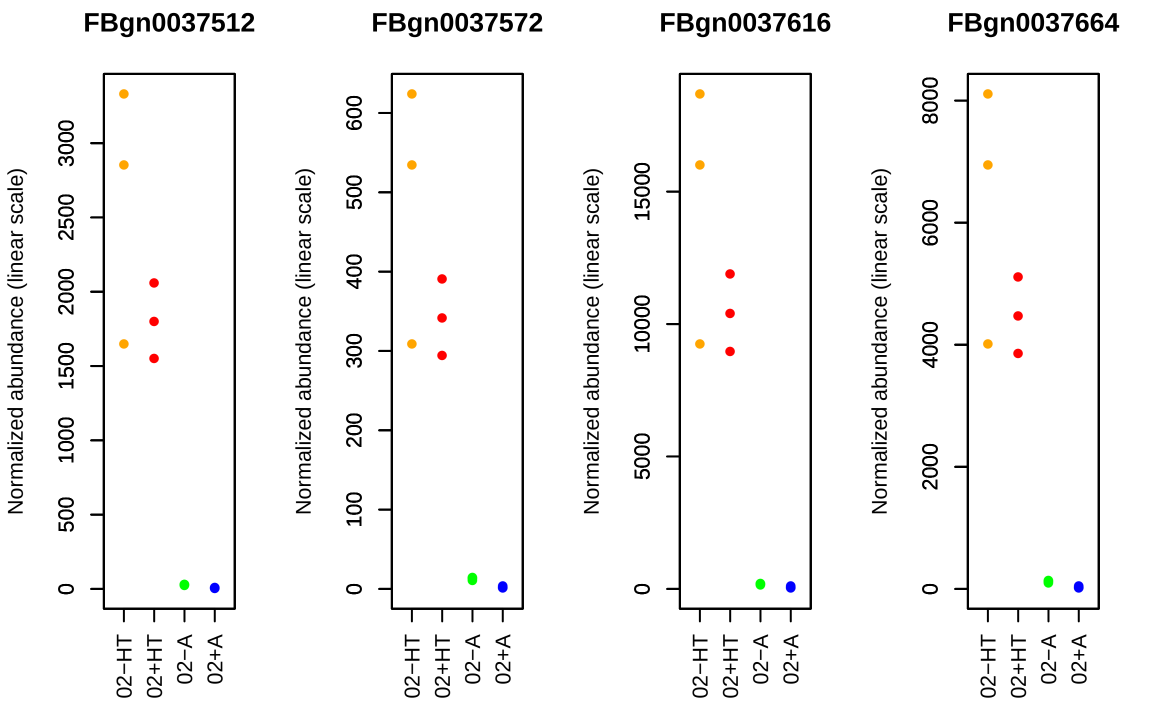

FBgn0037512
FBgn0037572
FBgn0037616
FBgn0037664
Normalized abundance (linear scale)
0
500
1000
1500
2000
2500
3000
100
200
300
400
500
600
5000
10000
15000
2000
4000
6000
8000
02−HT
02+HT
02−A
02+A

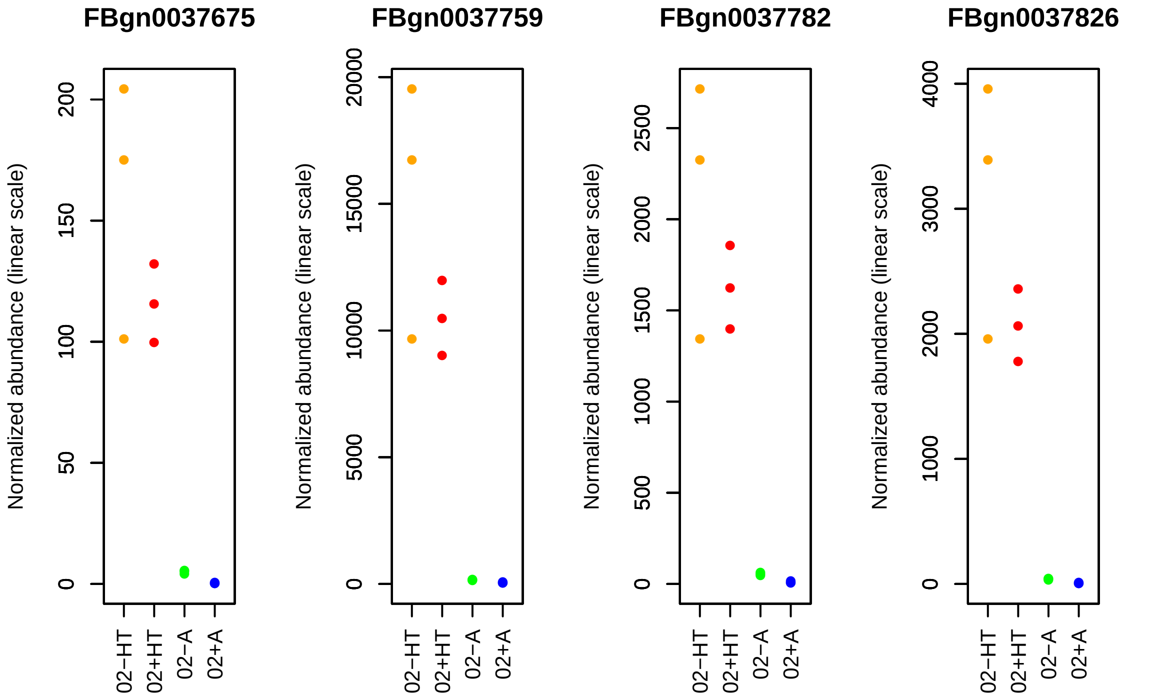

FBgn0037675
FBgn0037759
FBgn0037782
FBgn0037826
Normalized abundance (linear scale)
0
50
100
150
200
5000
10000
15000
20000
500
1000
1500
2000
2500
1000
2000
3000
4000
02−HT
02+HT
02−A
02+A

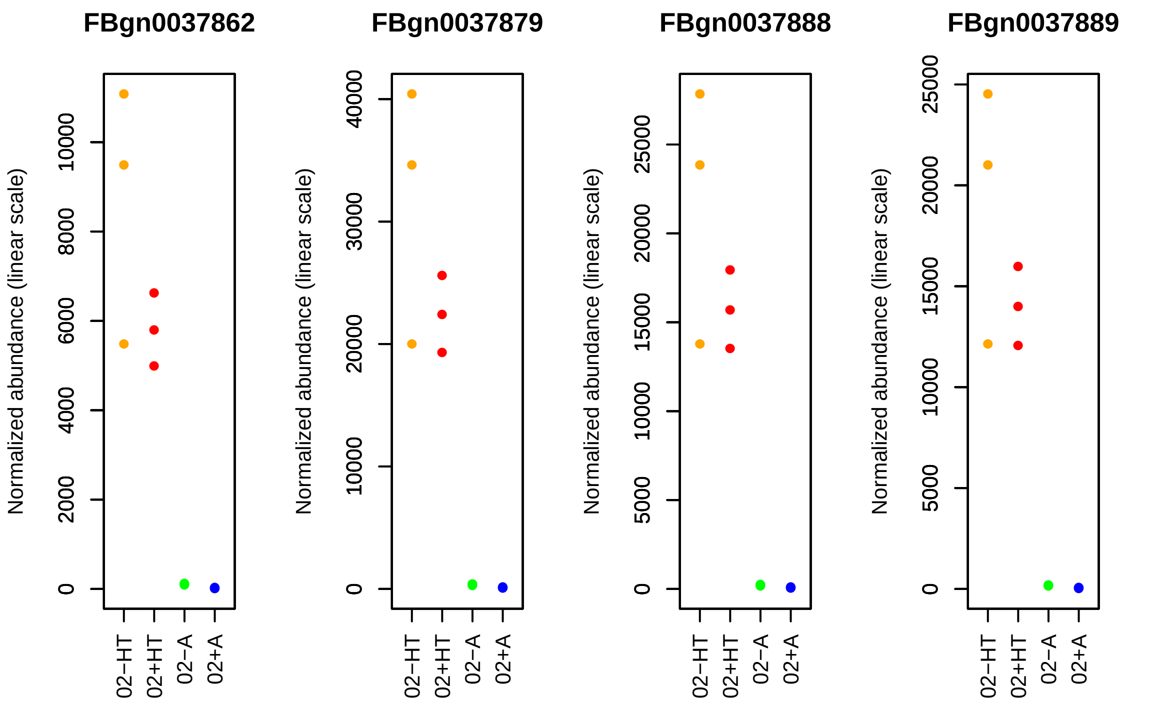
FBgn0037862
FBgn0037879
FBgn0037888
FBgn0037889
Normalized abundance (linear scale)
0
2000
4000
6000
8000
10000
20000
30000
40000
5000
15000
25000
02−HT
02+HT
02−A
02+A

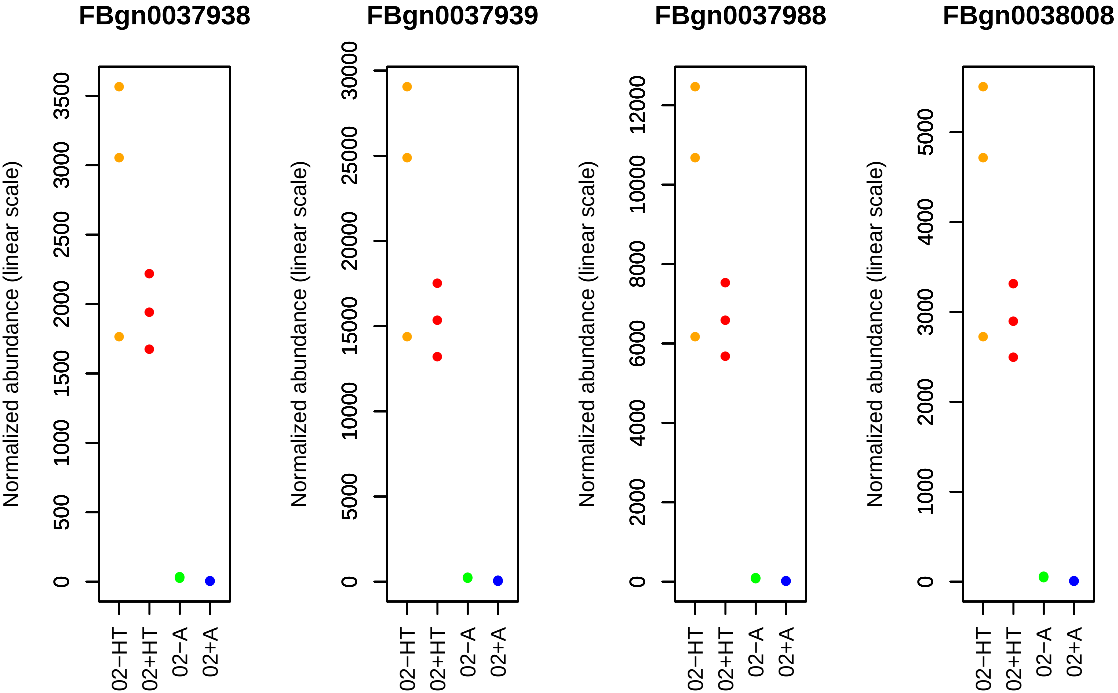

FBgn0037938
FBgn0037939
FBgn0037988
FBgn0038008
Normalized abundance (linear scale)
0
500
1000
1500
2000
2500
3000
3500
0
5000
10000
15000
20000
25000
30000
0
2000
4000
6000
8000
10000
12000
0
1000
2000
3000
4000
5000
02−HT
02+HT
02−A
02+A

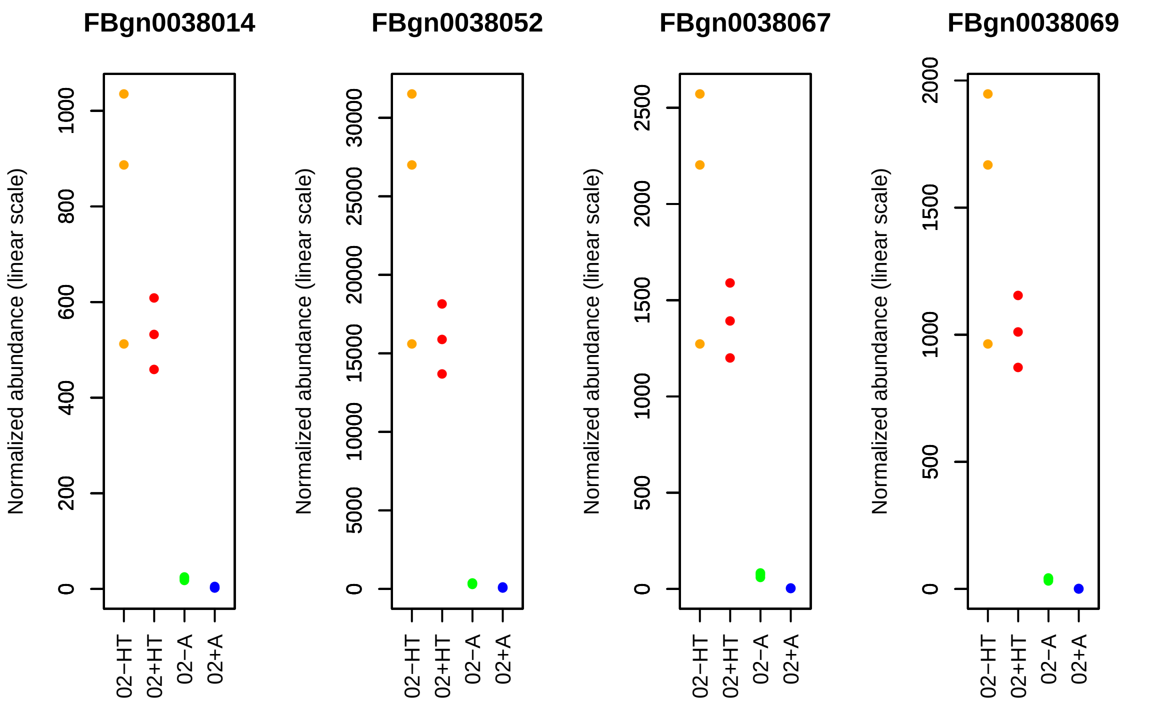

FBgn0038014
FBgn0038052
FBgn0038067
FBgn0038069
Normalized abundance (linear scale)
0
200
400
600
800
1000
5000
10000
15000
20000
25000
30000
500
1000
1500
2000
2500
02−HT
02+HT
02−A
02+A

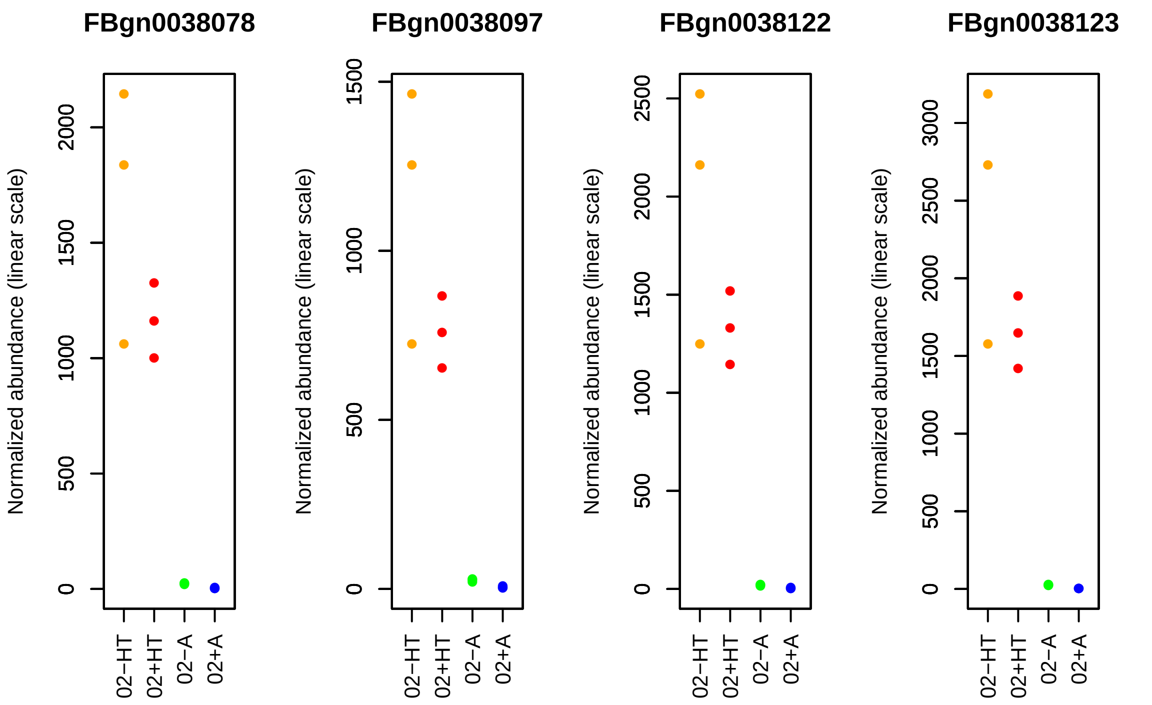
FBgn0038078
FBgn0038097
FBgn0038122
FBgn0038123
Normalized abundance (linear scale)
0
500
1000
1500
2000
2500
3000
02−HT
02+HT
02−A
02+A

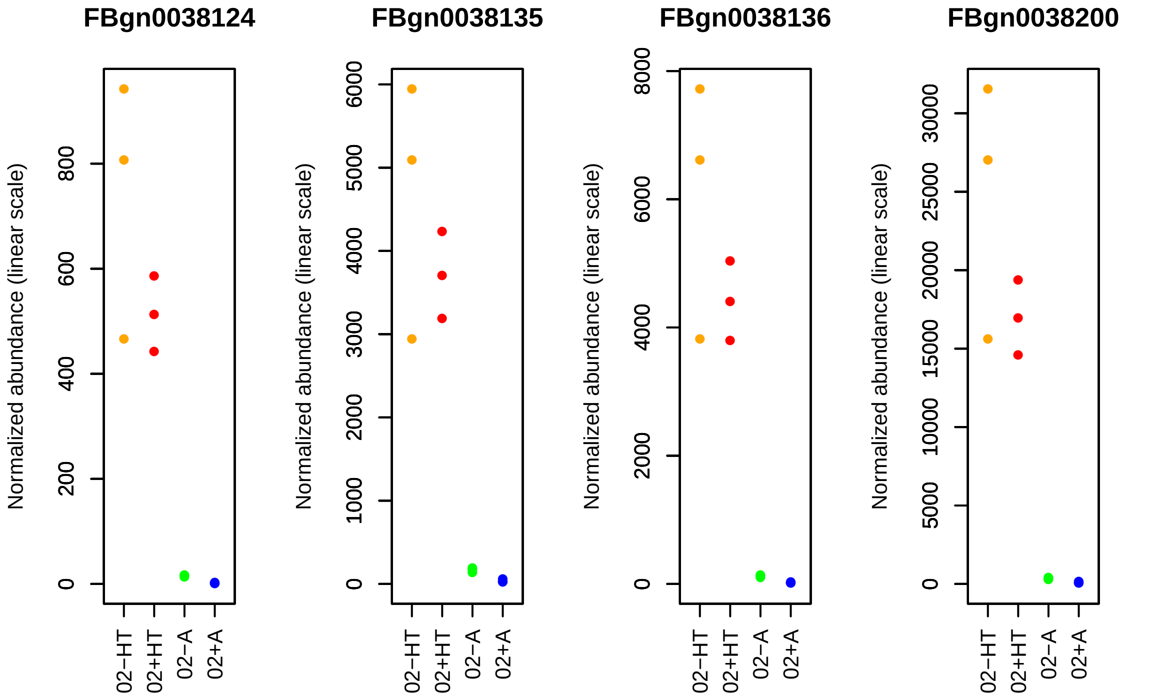
FBgn0038124
FBgn0038135
FBgn0038136
FBgn0038200
Normalized abundance (linear scale)
0
200
400
600
800
1000
2000
3000
4000
5000
6000
8000
10000
15000
20000
25000
30000
02−HT
02+HT
02−A
02+A

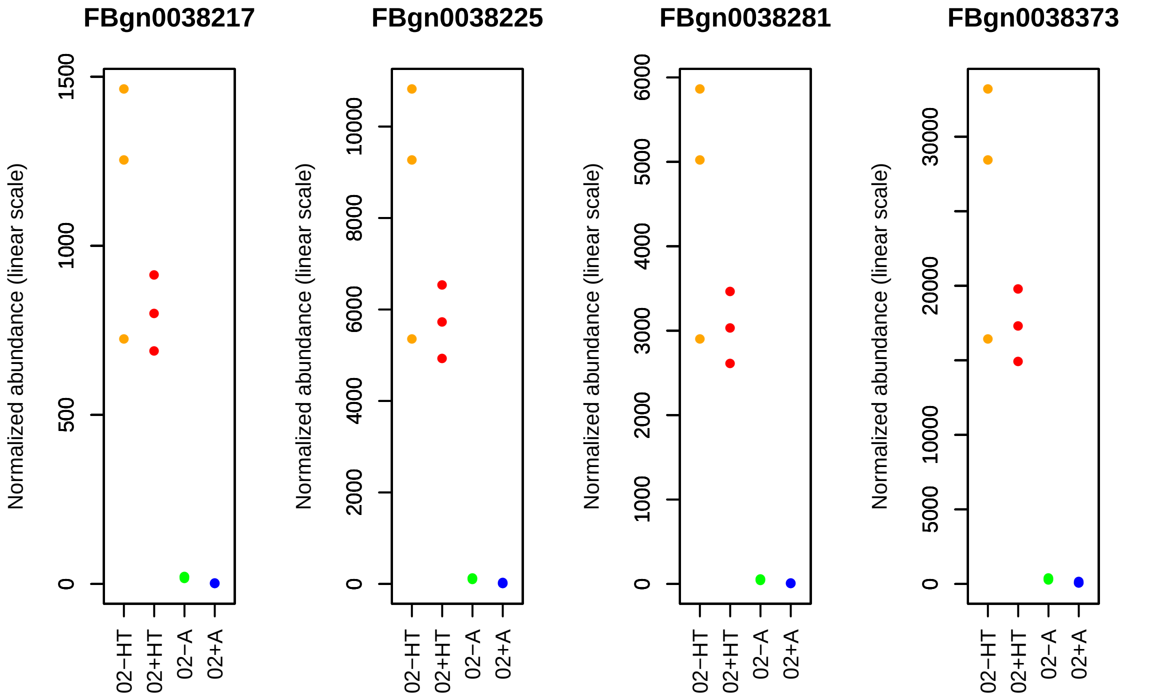

FBgn0038217
FBgn0038225
FBgn0038281
FBgn0038373
Normalized abundance (linear scale)
0
500
1000
1500
2000
4000
6000
8000
10000
3000
5000
6000
10000
20000
30000
02−HT
02+HT
02−A
02+A

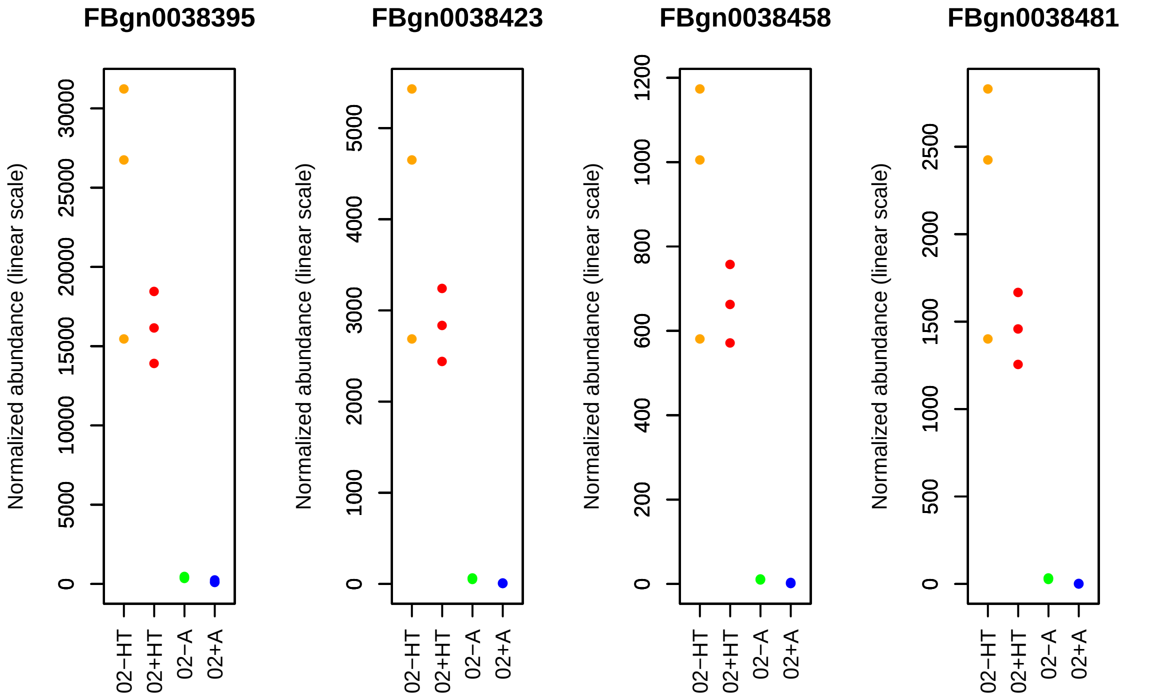

FBgn0038395
FBgn0038423
FBgn0038458
FBgn0038481
Normalized abundance (linear scale)
0
5000
10000
15000
20000
25000
30000
1000
2000
3000
4000
5000
200
400
600
800
1000
1200
500
1500
2500
02−HT
02+HT
02−A
02+A

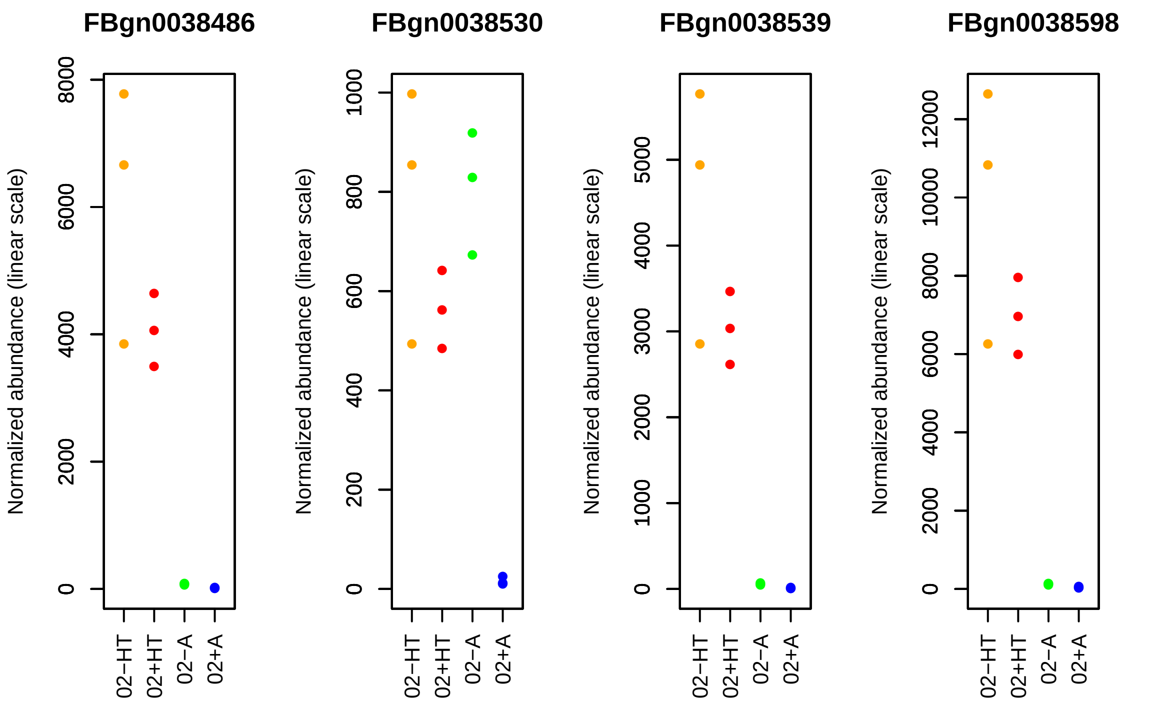

FBgn0038486
FBgn0038530
FBgn0038539
FBgn0038598
Normalized abundance (linear scale)
0
2000
4000
6000
8000
0
200
400
600
800
1000
0
1000
2000
3000
4000
5000
0
2000
4000
6000
8000
10000
12000
02−HT
02+HT
02−A
02+A

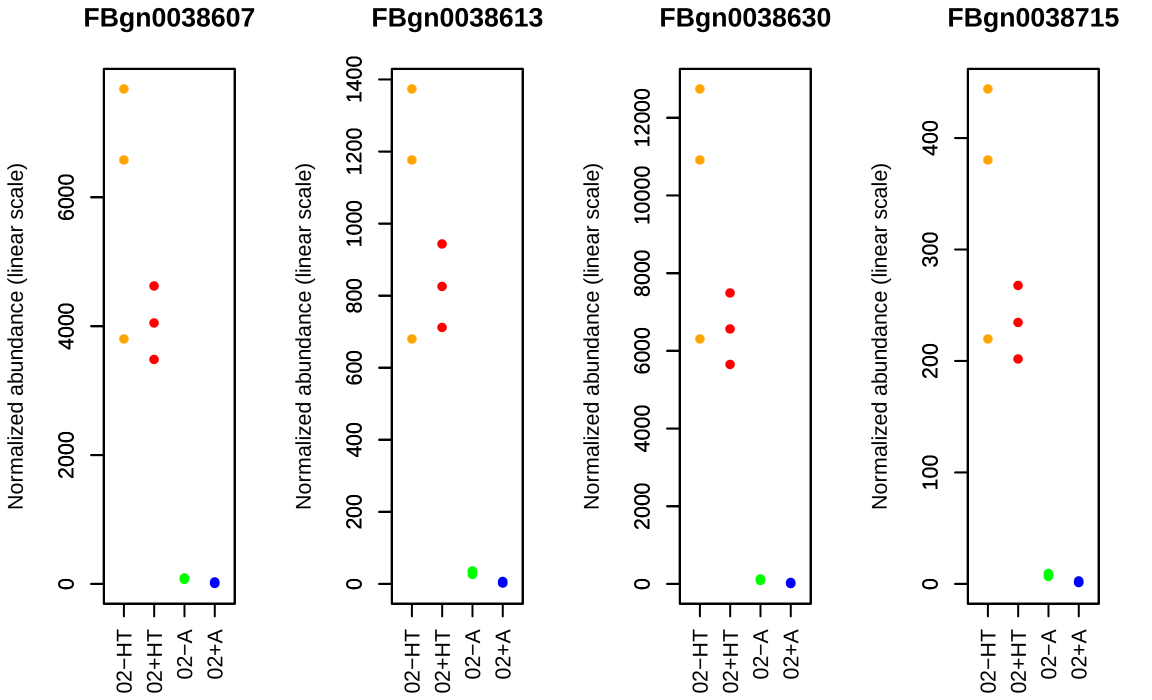
FBgn0038607
FBgn0038613
FBgn0038630
FBgn0038715
Normalized abundance (linear scale)
02−HT
02+HT
02−A
02+A
0
2000
4000
6000
200
400
600
800
1000
1200
1400
8000
10000
12000
100
300

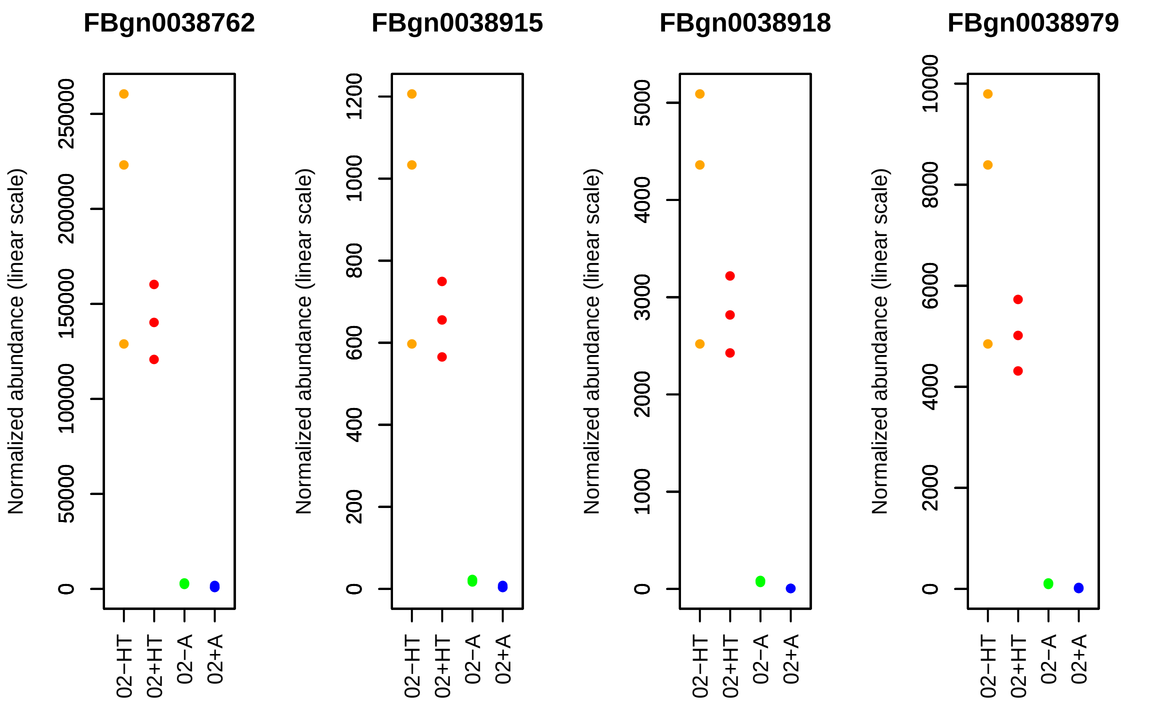

FBgn0038762
FBgn0038915
FBgn0038918
FBgn0038979
Normalized abundance (linear scale)
0
50000
100000
150000
200000
250000
200
400
600
800
1000
1200
1000
2000
3000
4000
5000
2000
4000
6000
8000
10000
02−HT
02+HT
02−A
02+A

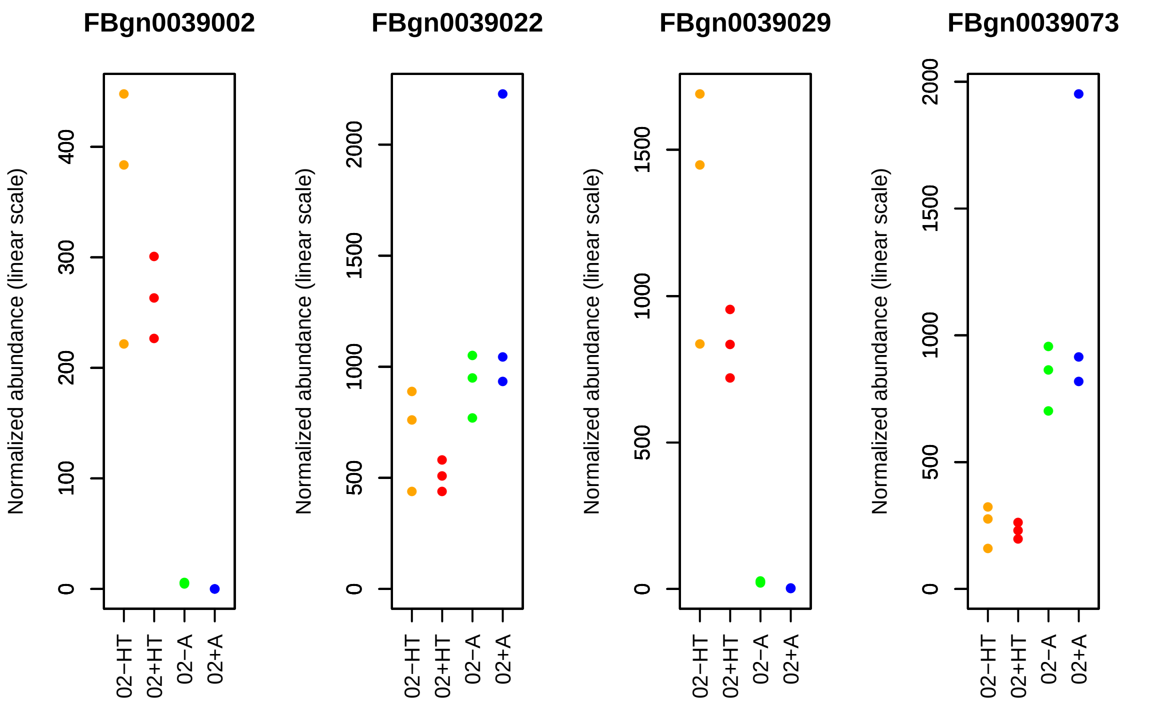

FBgn0039002
FBgn0039022
FBgn0039029
FBgn0039073
Normalized abundance (linear scale)
0
100
200
300
400
500
1000
1500
2000
02−HT
02+HT
02−A
02+A

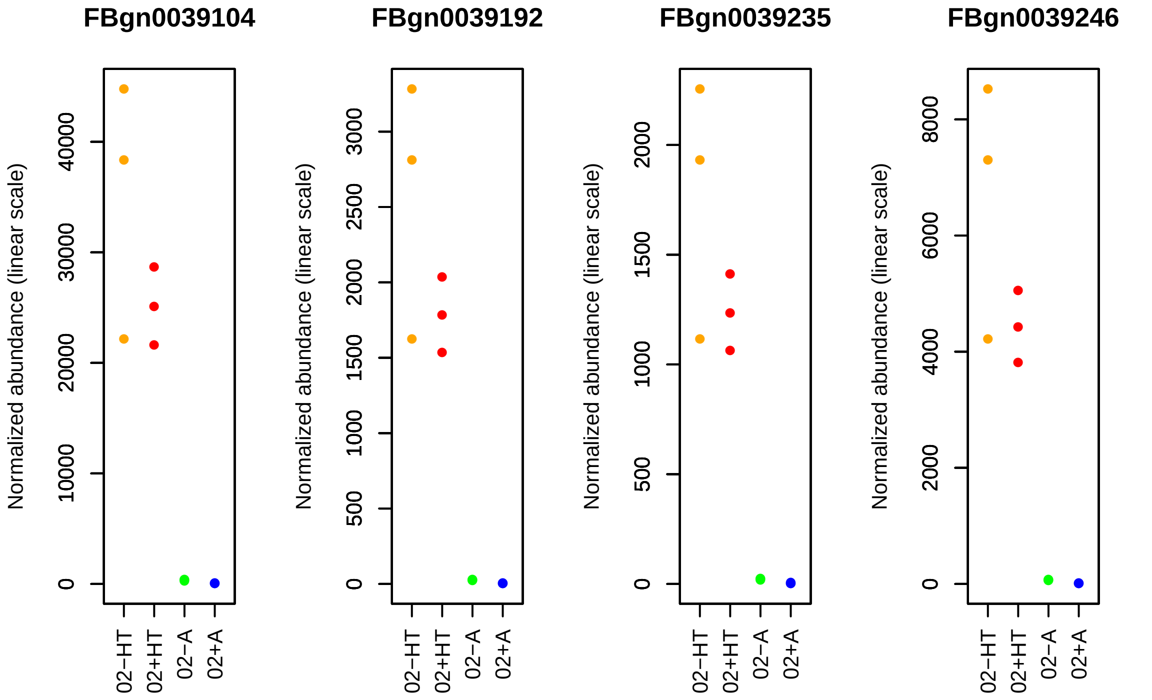

FBgn0039104
FBgn0039192
FBgn0039235
FBgn0039246
Normalized abundance (linear scale)
0
10000
20000
30000
40000
500
1000
1500
2000
2500
3000
4000
6000
8000
02−HT
02+HT
02−A
02+A

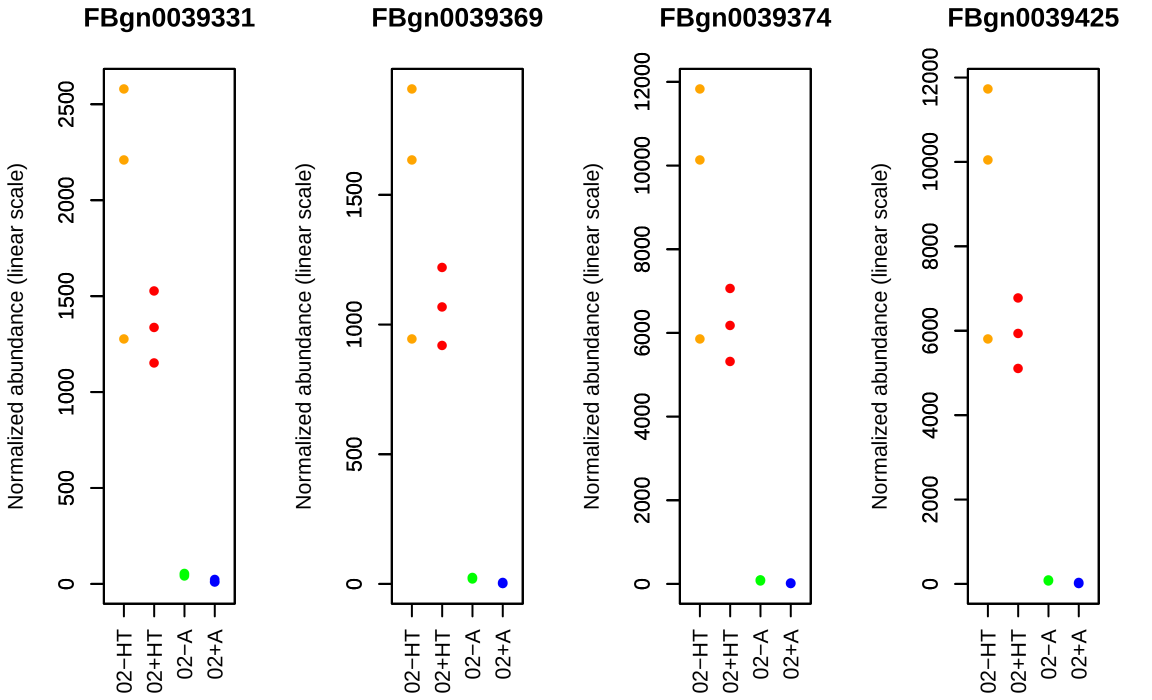
FBgn0039331
FBgn0039369
FBgn0039374
FBgn0039425
Normalized abundance (linear scale)
0
500
1000
1500
2000
2500
0
500
1000
1500
0
2000
4000
6000
8000
10000
12000
02−HT
02+HT
02−A
02+A

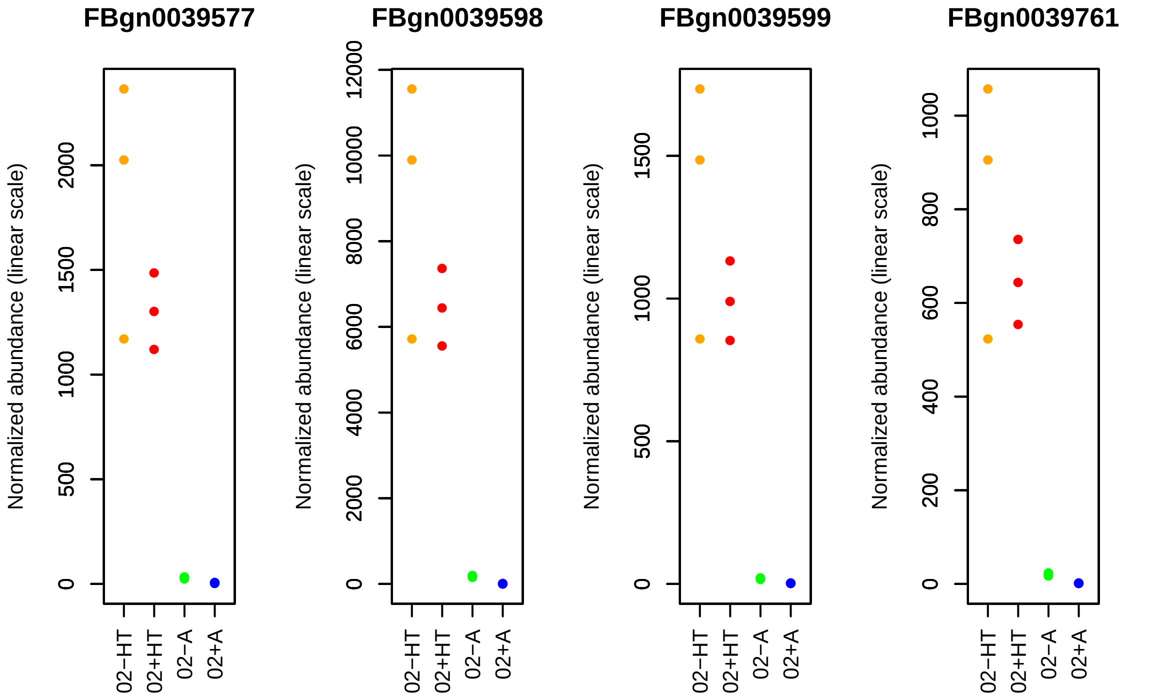
FBgn0039577
FBgn0039598
FBgn0039599
FBgn0039761
Normalized abundance (linear scale)
0
500
1000
1500
2000
2000
4000
6000
8000
10000
12000
200
400
600
800
02−HT
02+HT
02−A
02+A

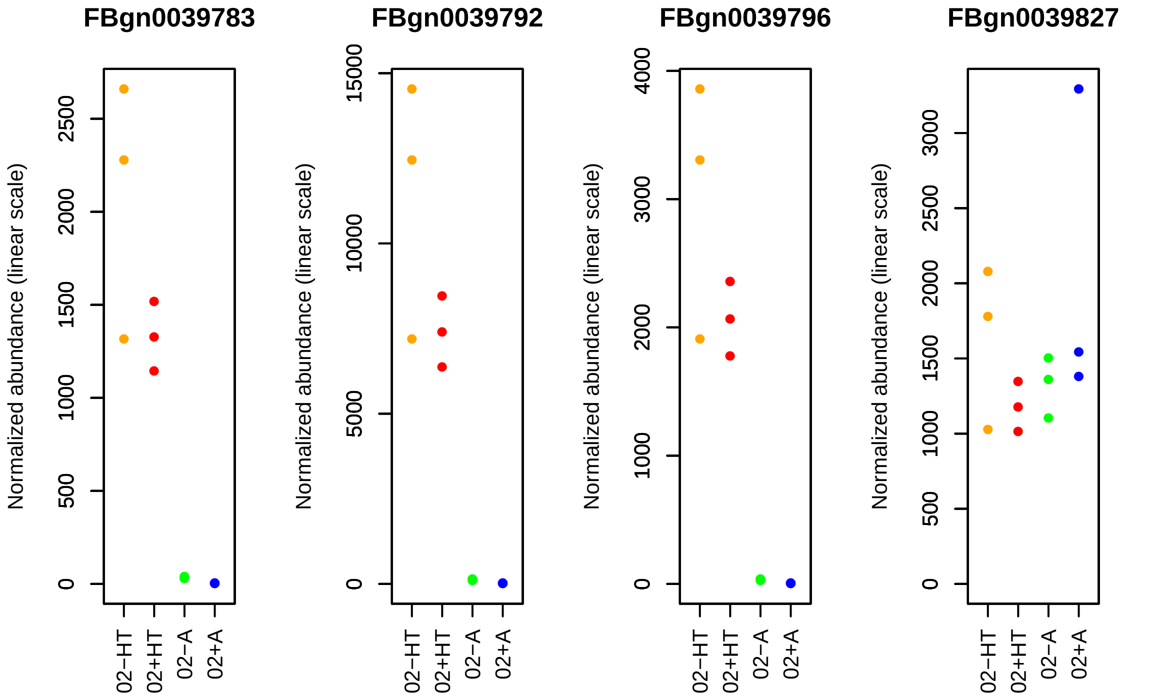

FBgn0039783
FBgn0039792
FBgn0039796
FBgn0039827
Normalized abundance (linear scale)
0
500
1000
1500
2000
2500
3000
5000
10000
15000
4000
02−HT
02+HT
02−A
02+A

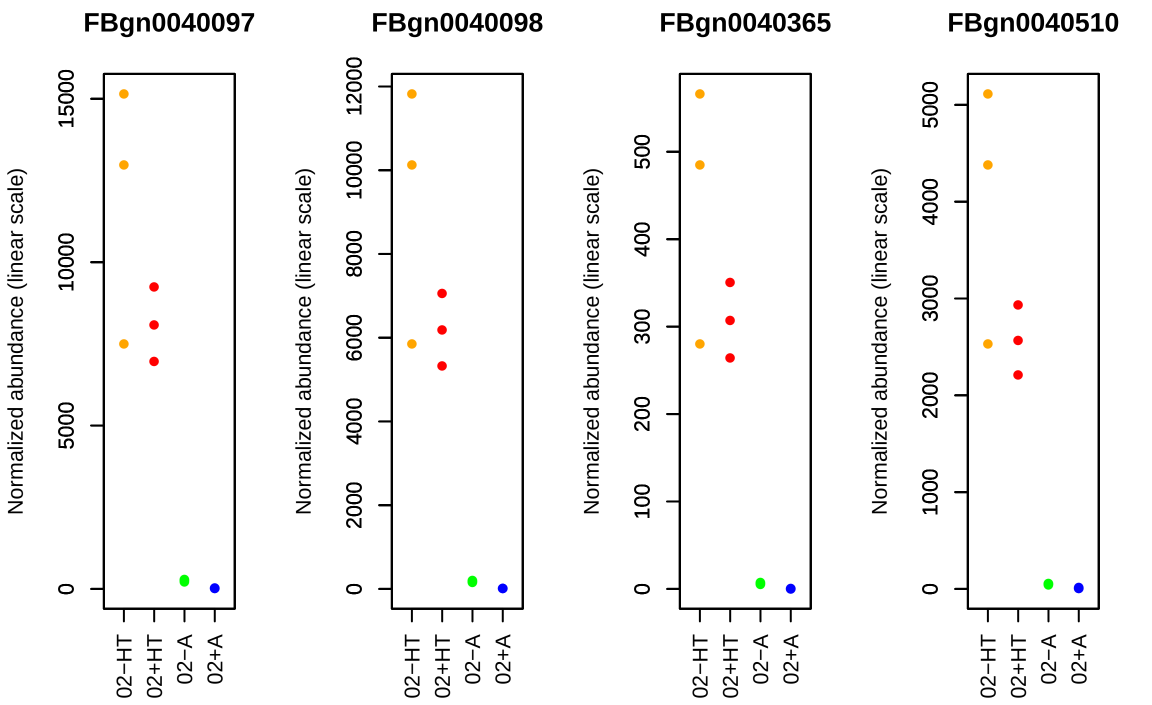

FBgn0040097
FBgn0040098
FBgn0040365
FBgn0040510
Normalized abundance (linear scale)
0
5000
10000
15000
0
2000
4000
6000
8000
10000
12000
0
100
200
300
400
500
0
1000
2000
3000
4000
5000
02−HT
02+HT
02−A
02+A

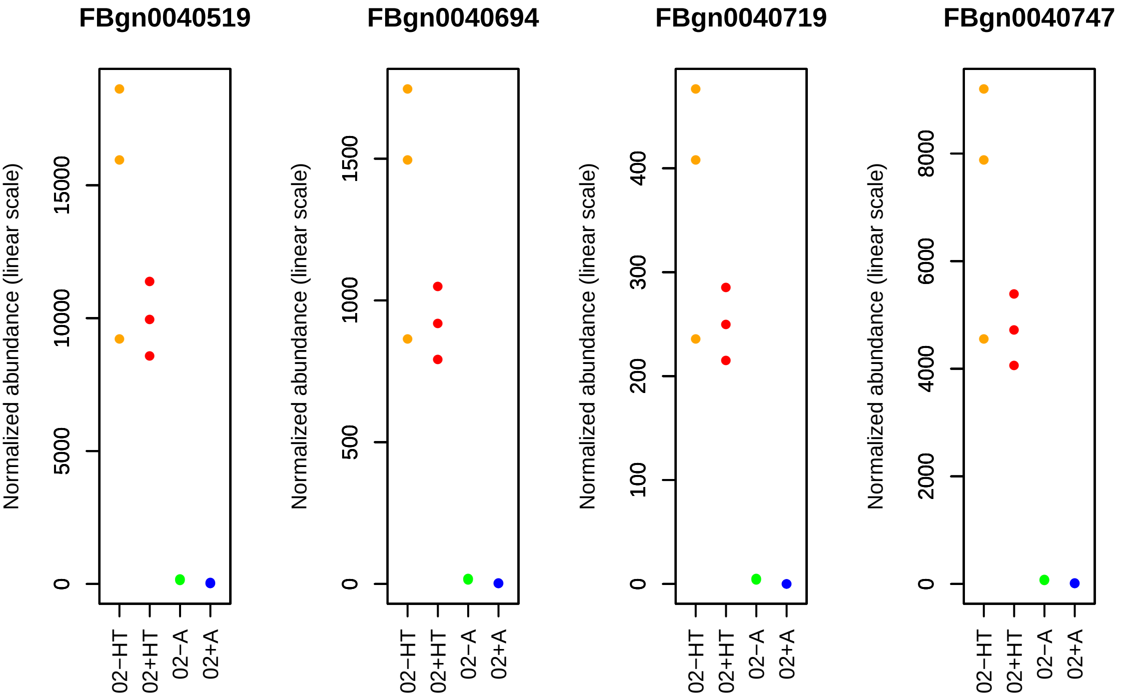
FBgn0040519
FBgn0040694
FBgn0040719
FBgn0040747
Normalized abundance (linear scale)
0
5000
10000
15000
500
1000
1500
100
200
300
400
2000
4000
6000
8000
02−HT
02+HT
02−A
02+A

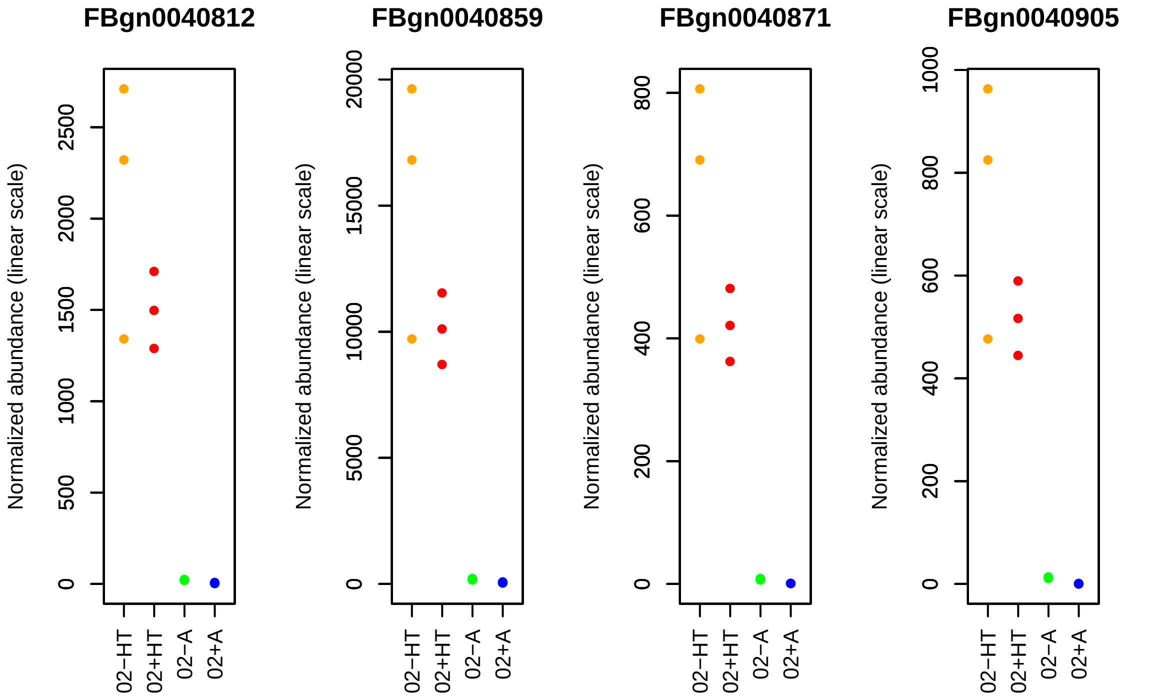
FBgn0040812
FBgn0040859
FBgn0040871
FBgn0040905
Normalized abundance (linear scale)
0
500
1000
1500
2000
2500
5000
10000
15000
20000
200
400
600
800
1000
02−HT
02+HT
02−A
02+A

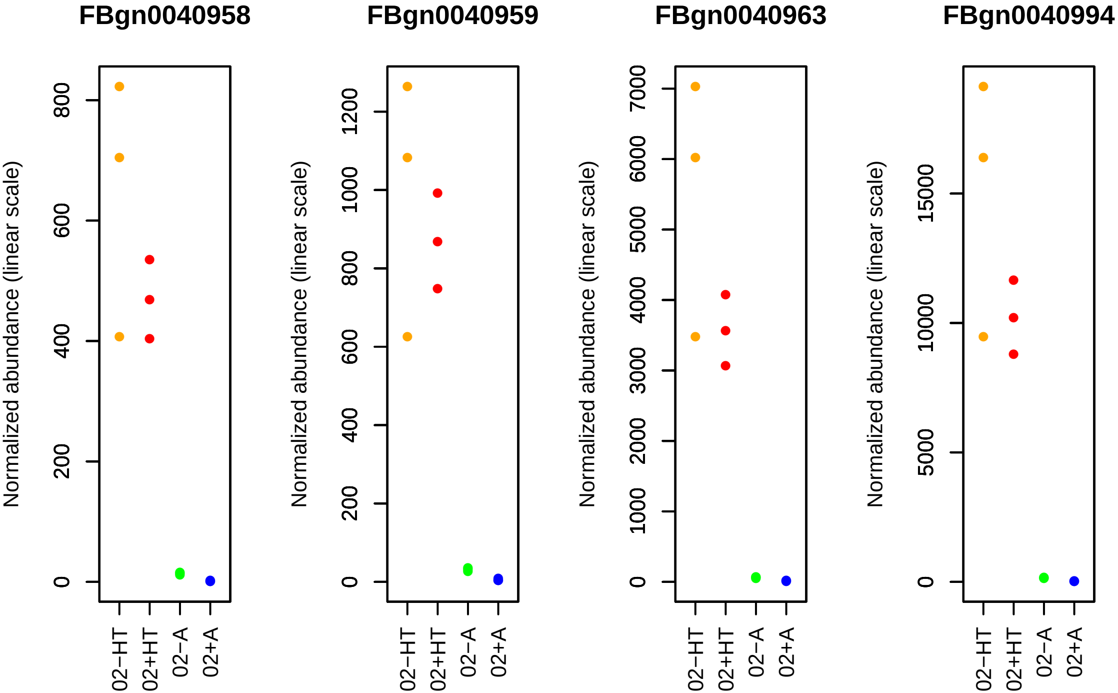
FBgn0040958
FBgn0040959
FBgn0040963
FBgn0040994
Normalized abundance (linear scale)
0
200
400
600
800
1000
1200
2000
3000
4000
5000
6000
7000
5000
10000
15000
02−HT
02+HT
02−A
02+A

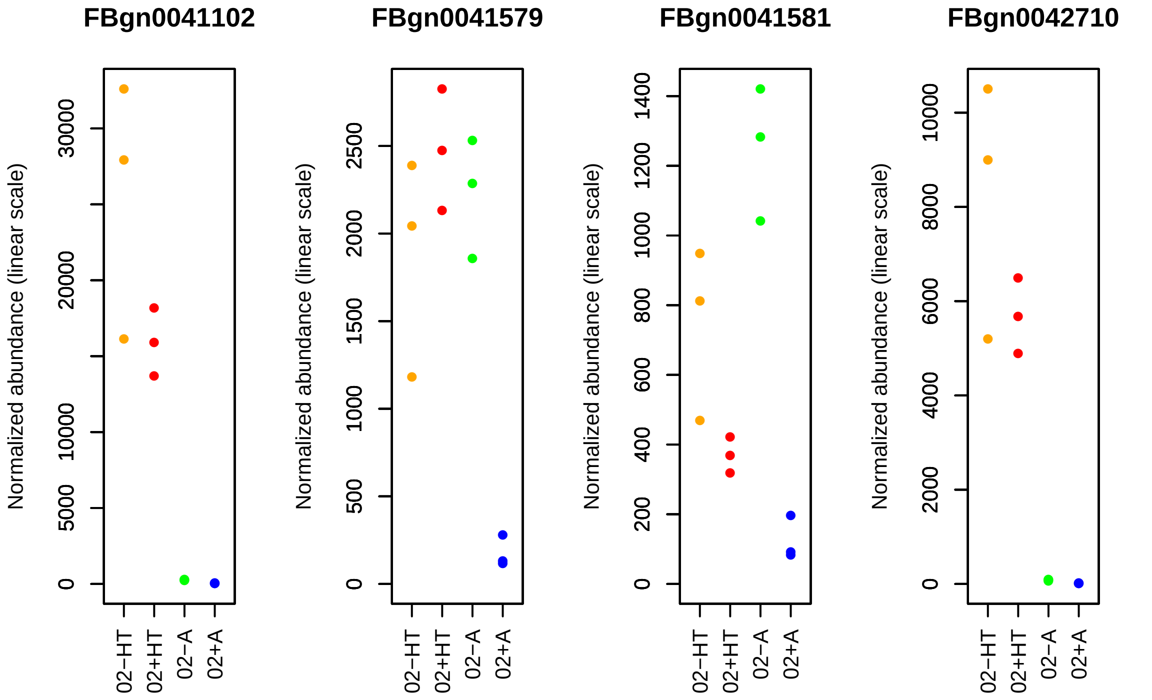

FBgn0041102
FBgn0041579
FBgn0041581
FBgn0042710
Normalized abundance (linear scale)
0
5000
10000
20000
30000
500
1000
1500
2000
2500
200
400
600
800
1000
1200
1400
2000
4000
6000
8000
10000
02−HT
02+HT
02−A
02+A

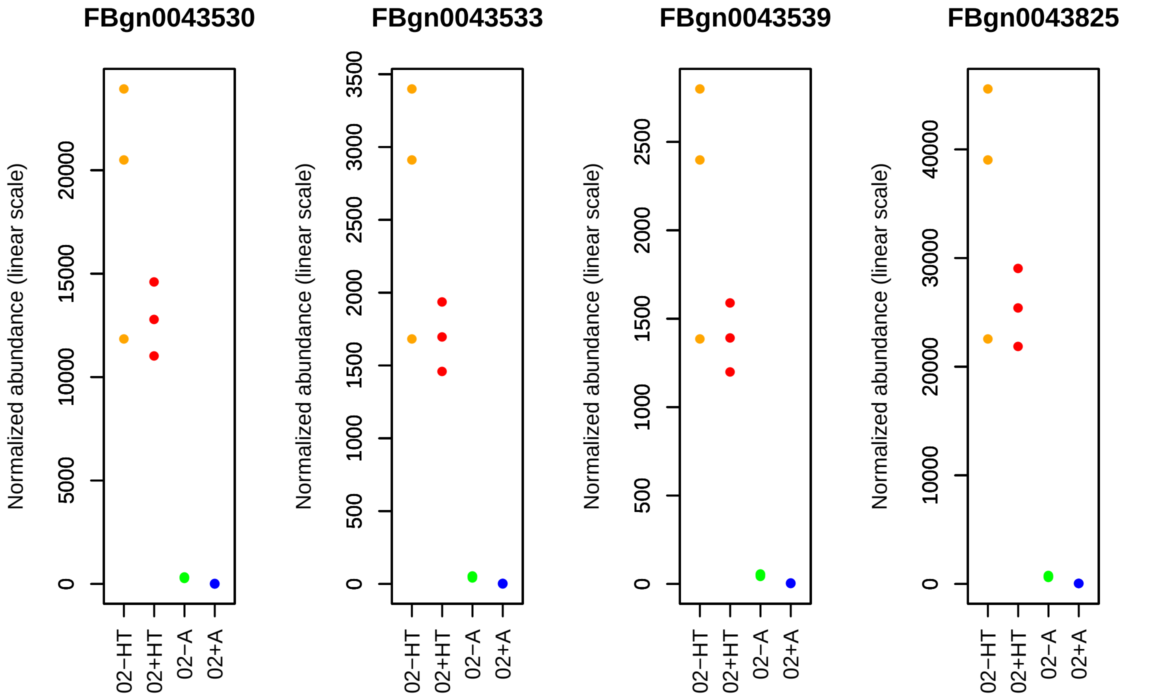

FBgn0043530
FBgn0043533
FBgn0043539
FBgn0043825
Normalized abundance (linear scale)
0
5000
10000
15000
20000
500
1000
1500
2000
2500
3000
3500
30000
40000
02−HT
02+HT
02−A
02+A

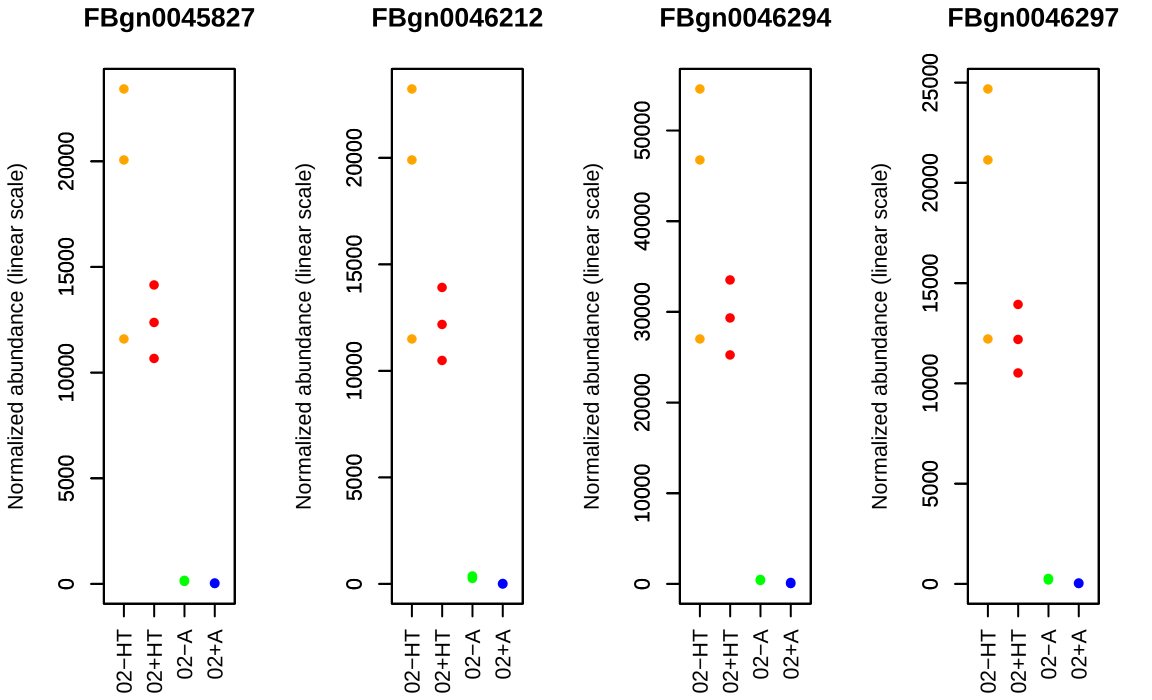

FBgn0045827
FBgn0046212
FBgn0046294
FBgn0046297
Normalized abundance (linear scale)
0
5000
10000
15000
20000
25000
30000
40000
50000
02−HT
02+HT
02−A
02+A

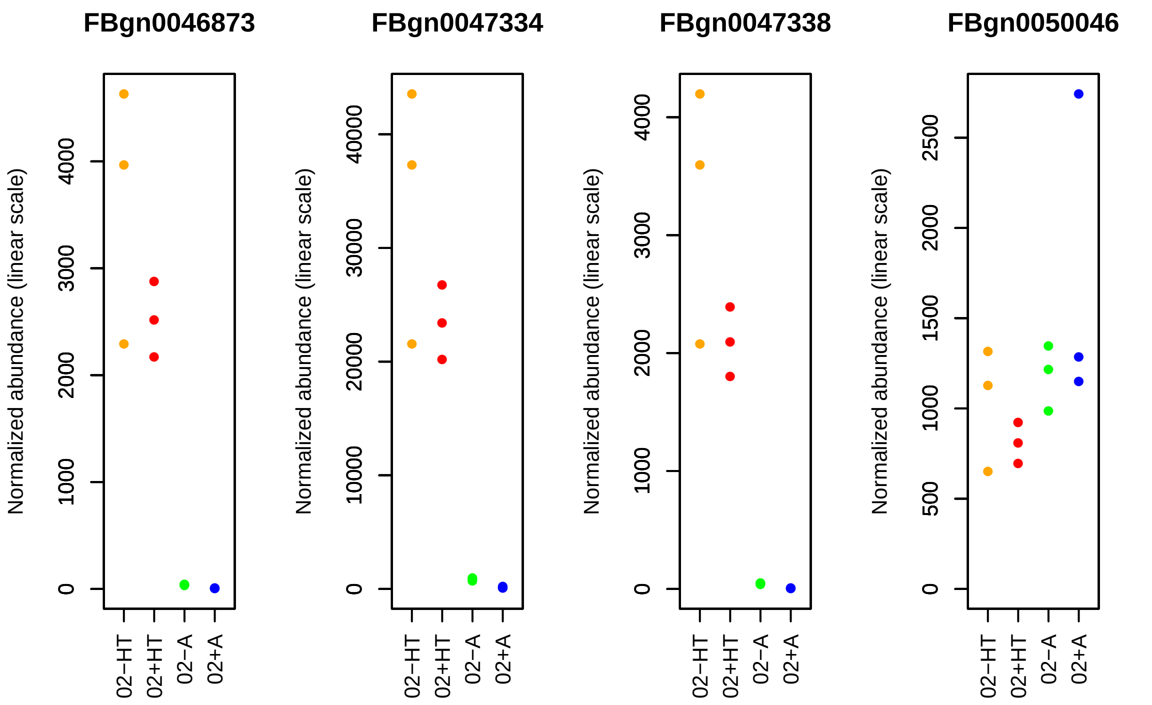

FBgn0046873
FBgn0047334
FBgn0047338
FBgn0050046
Normalized abundance (linear scale)
0
1000
2000
3000
4000
10000
20000
30000
40000
500
1500
2500
02−HT
02+HT
02−A
02+A

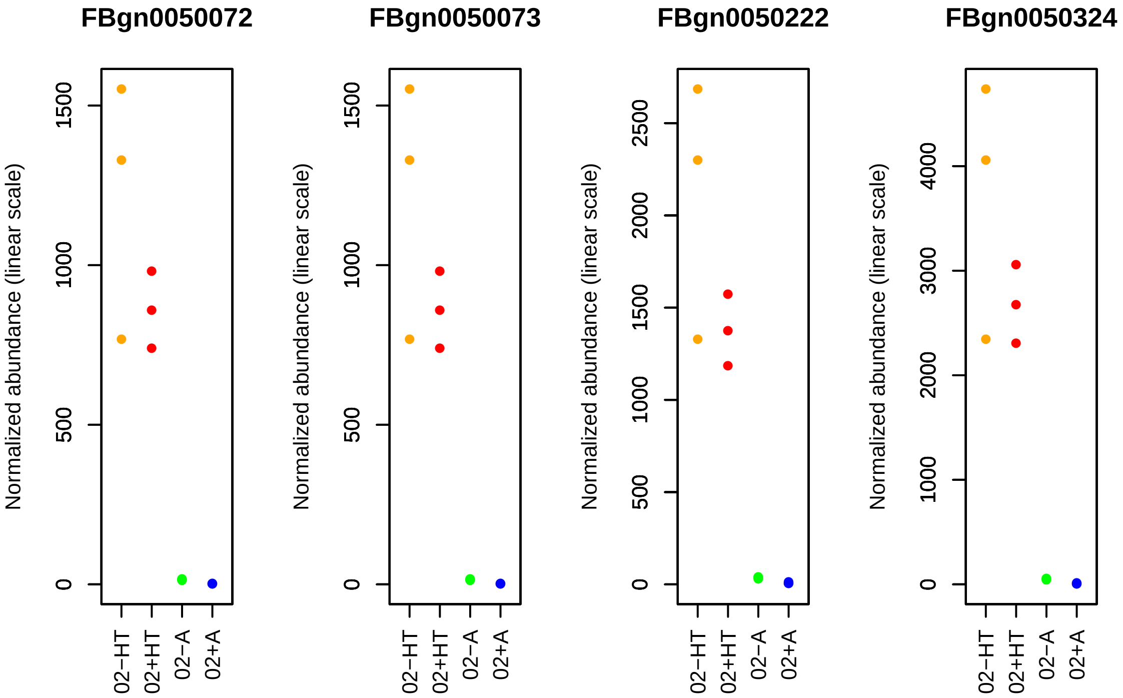
FBgn0050072
FBgn0050073
FBgn0050222
FBgn0050324
Normalized abundance (linear scale)
0
500
1000
1500
2000
2500
3000
4000
02−HT
02+HT
02−A
02+A

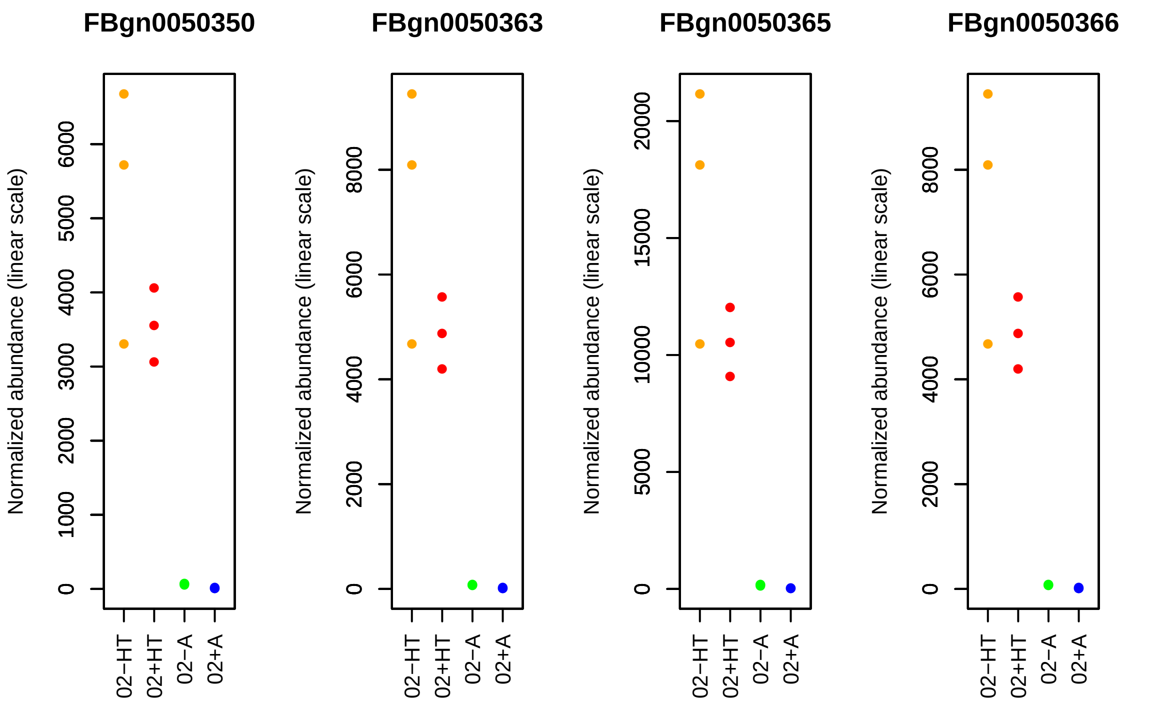
FBgn0050350
FBgn0050363
FBgn0050365
FBgn0050366
Normalized abundance (linear scale)
0
1000
2000
3000
4000
5000
6000
8000
10000
15000
20000
02−HT
02+HT
02−A
02+A

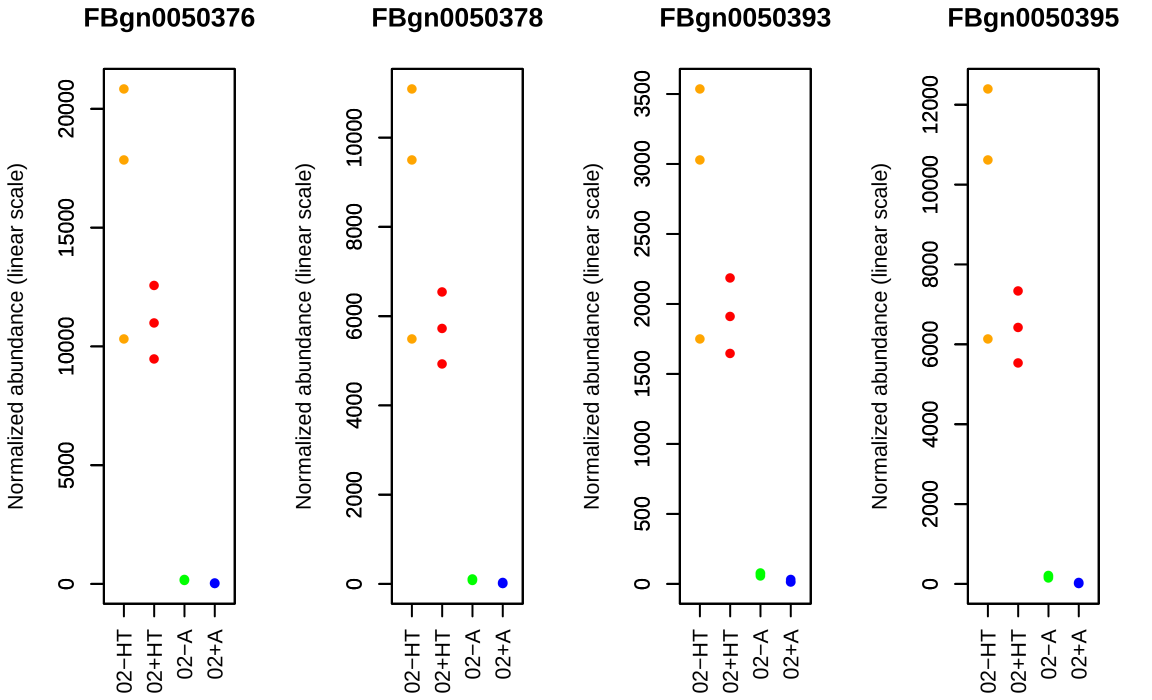

FBgn0050376
FBgn0050378
FBgn0050393
FBgn0050395
Normalized abundance (linear scale)
0
5000
10000
15000
20000
2000
4000
6000
8000
10000
500
1000
1500
2000
2500
3000
3500
12000
02−HT
02+HT
02−A
02+A

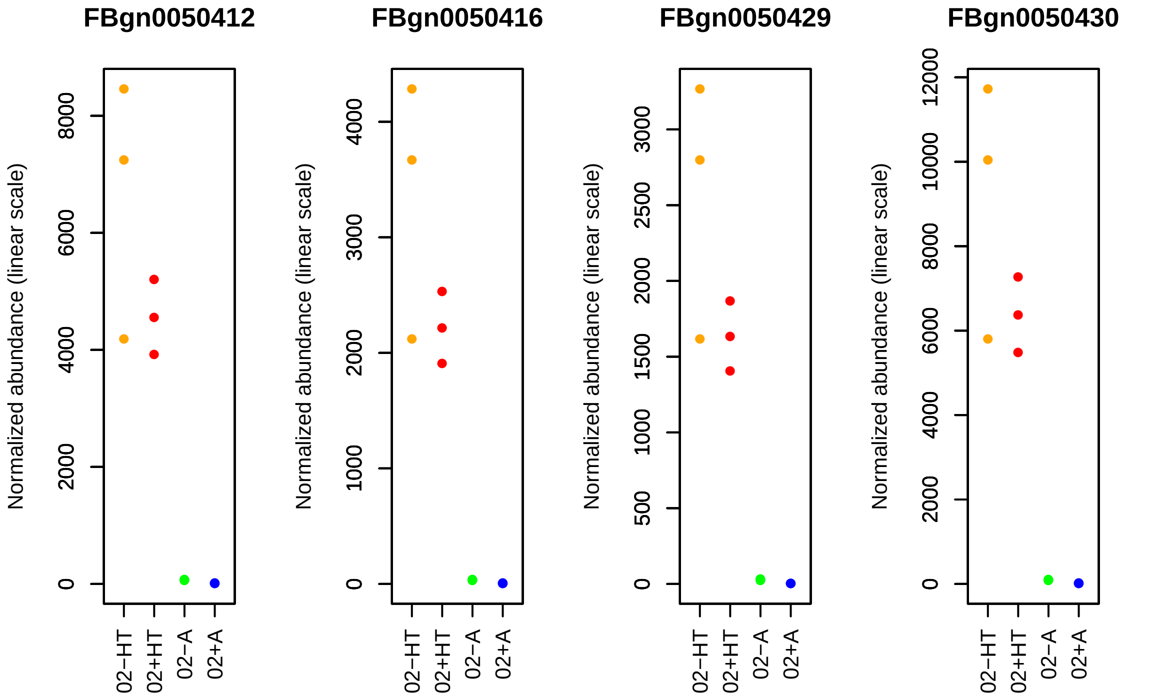

FBgn0050412
FBgn0050416
FBgn0050429
FBgn0050430
Normalized abundance (linear scale)
0
2000
4000
6000
8000
0
1000
2000
3000
4000
0
500
1000
1500
2000
2500
3000
0
2000
4000
6000
8000
10000
12000
02−HT
02+HT
02−A
02+A

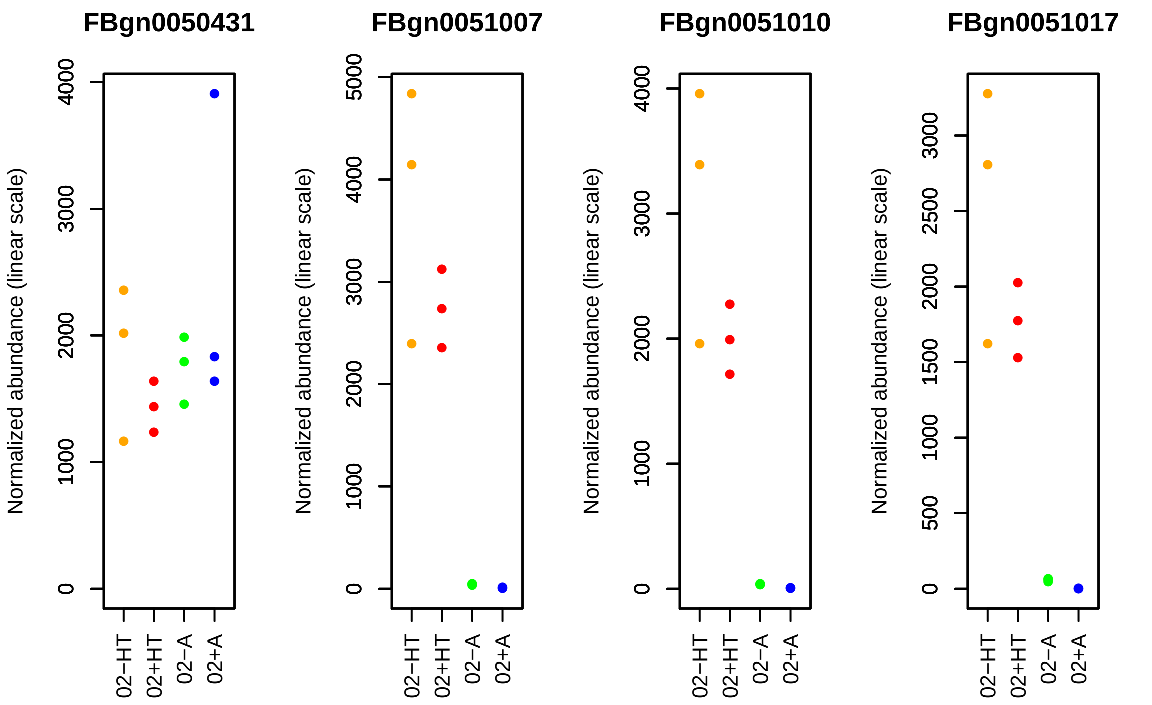
FBgn0050431
FBgn0051007
FBgn0051010
FBgn0051017
Normalized abundance (linear scale)
0
1000
2000
3000
4000
5000
500
1500
2500
02−HT
02+HT
02−A
02+A

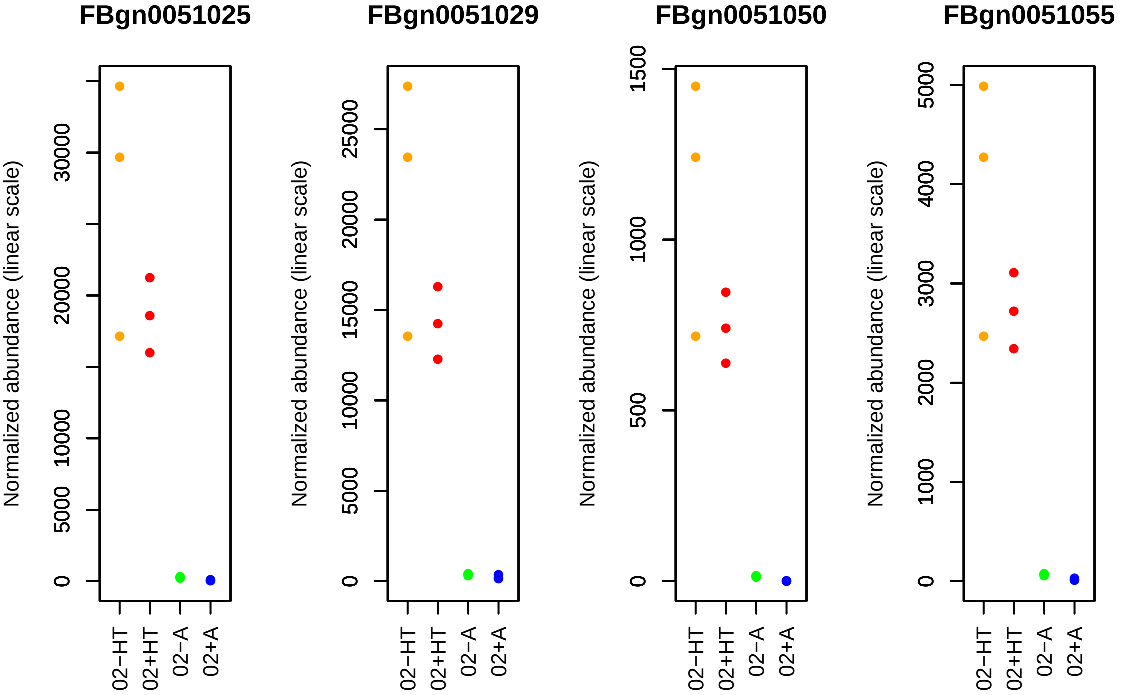
FBgn0051025
FBgn0051029
FBgn0051050
FBgn0051055
Normalized abundance (linear scale)
0
5000
10000
20000
30000
15000
25000
500
1000
1500
1000
2000
3000
4000
5000
02−HT
02+HT
02−A
02+A

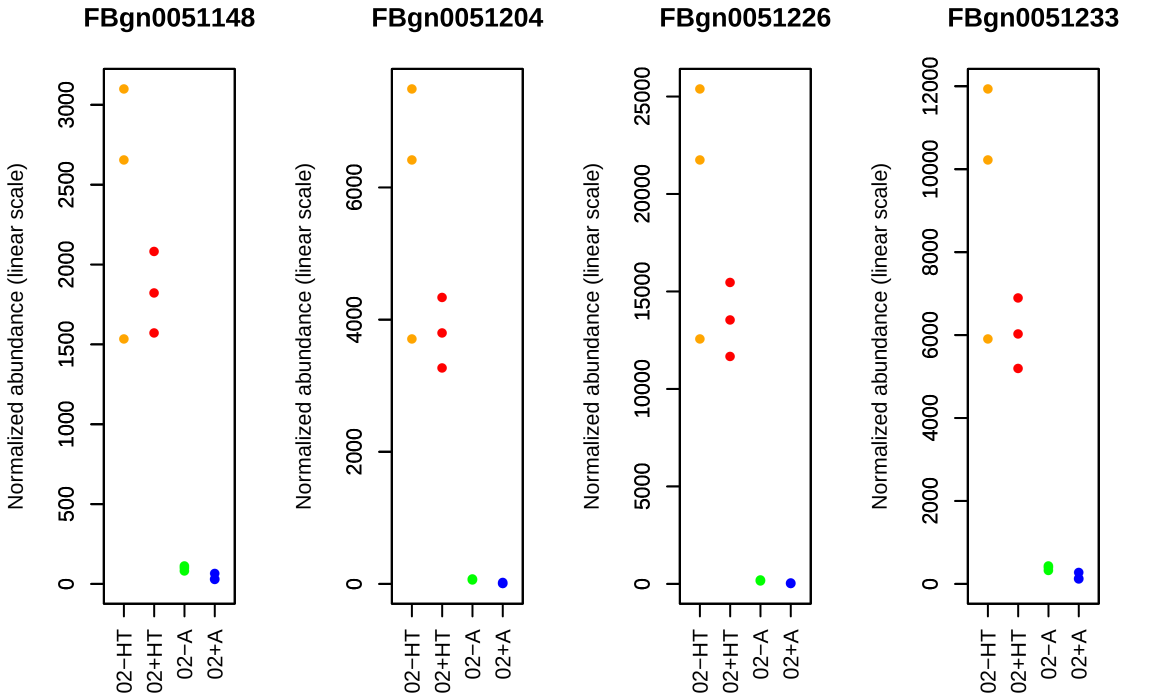

FBgn0051148
FBgn0051204
FBgn0051226
FBgn0051233
Normalized abundance (linear scale)
0
500
1000
1500
2000
2500
3000
2000
4000
6000
5000
10000
15000
20000
25000
8000
12000
02−HT
02+HT
02−A
02+A

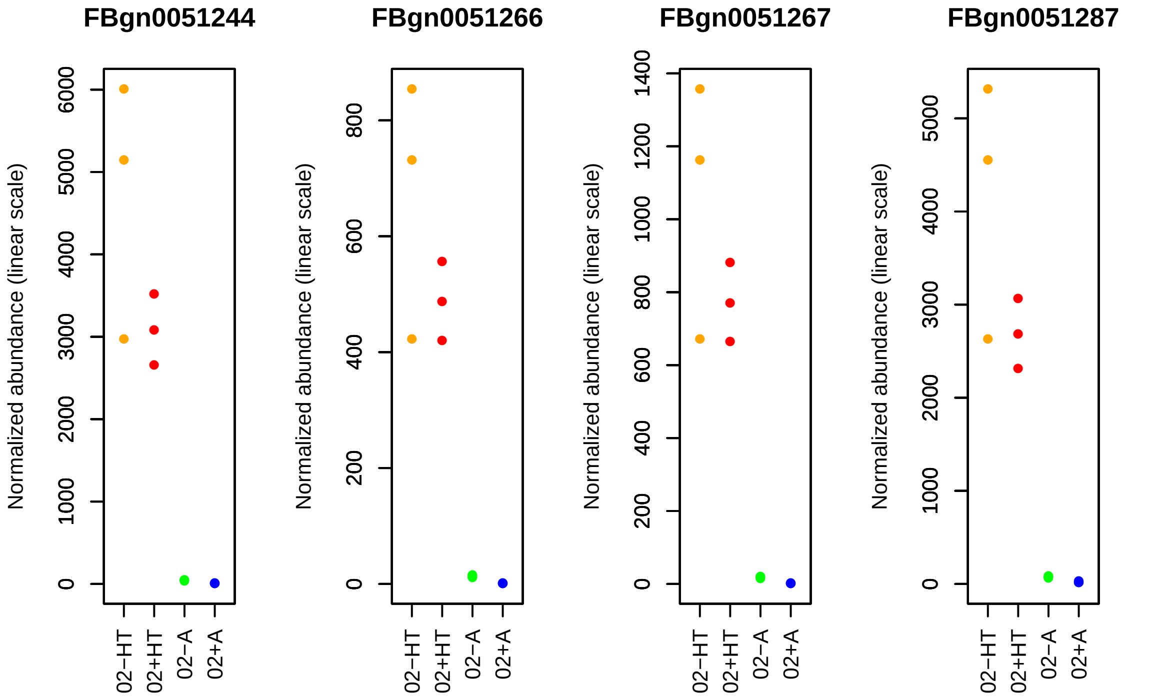

FBgn0051244
FBgn0051266
FBgn0051267
FBgn0051287
Normalized abundance (linear scale)
0
1000
2000
3000
4000
5000
6000
200
400
600
800
1200
1400
02−HT
02+HT
02−A
02+A

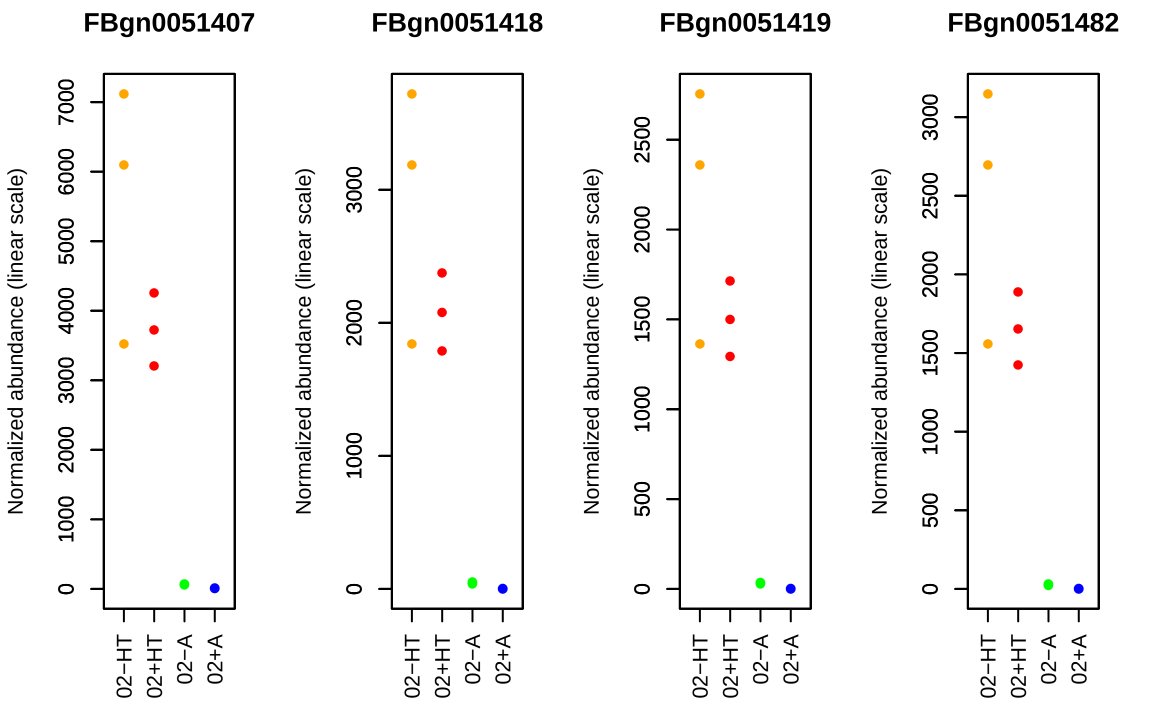
FBgn0051407
FBgn0051418
FBgn0051419
FBgn0051482
Normalized abundance (linear scale)
0
1000
2000
3000
4000
5000
6000
7000
500
1500
2500
02−HT
02+HT
02−A
02+A

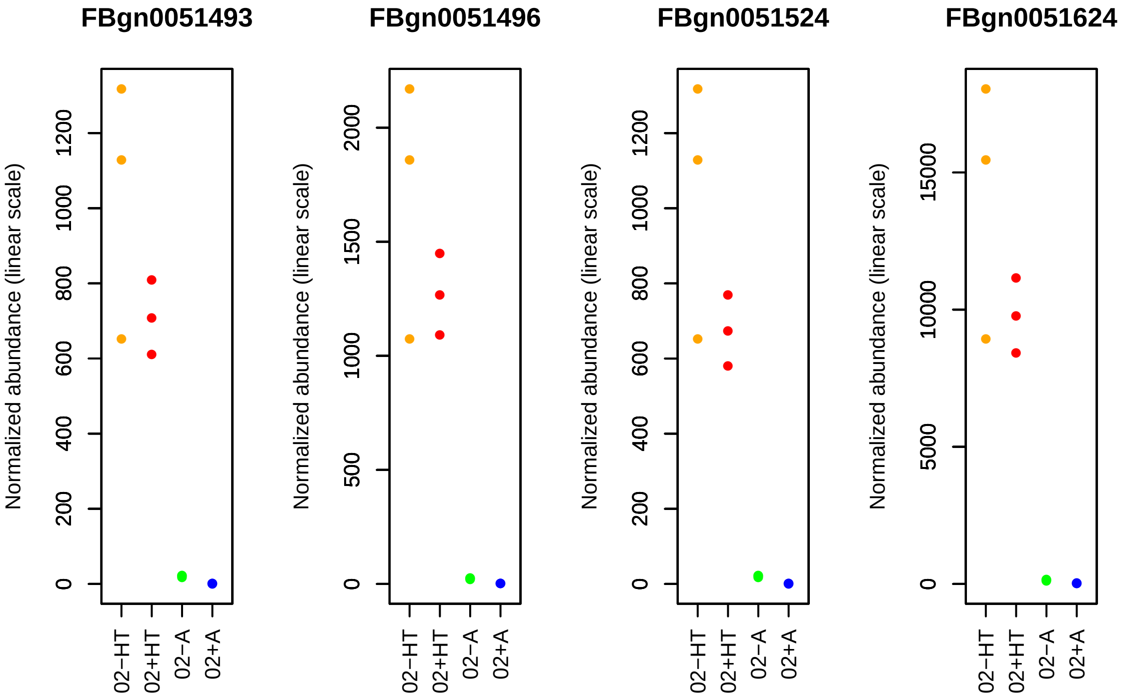
FBgn0051493
FBgn0051496
FBgn0051524
FBgn0051624
Normalized abundance (linear scale)
0
200
400
600
800
1000
1200
500
1000
1500
2000
5000
10000
15000
02−HT
02+HT
02−A
02+A

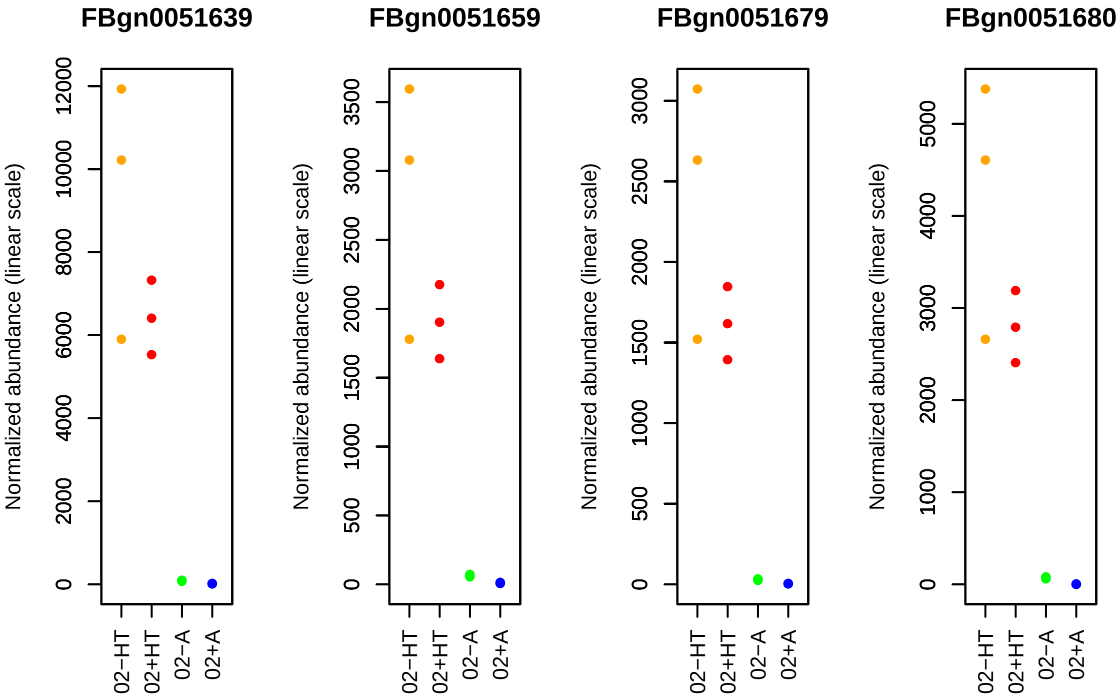
FBgn0051639
FBgn0051659
FBgn0051679
FBgn0051680
Normalized abundance (linear scale)
02−HT
02+HT
02−A
02+A

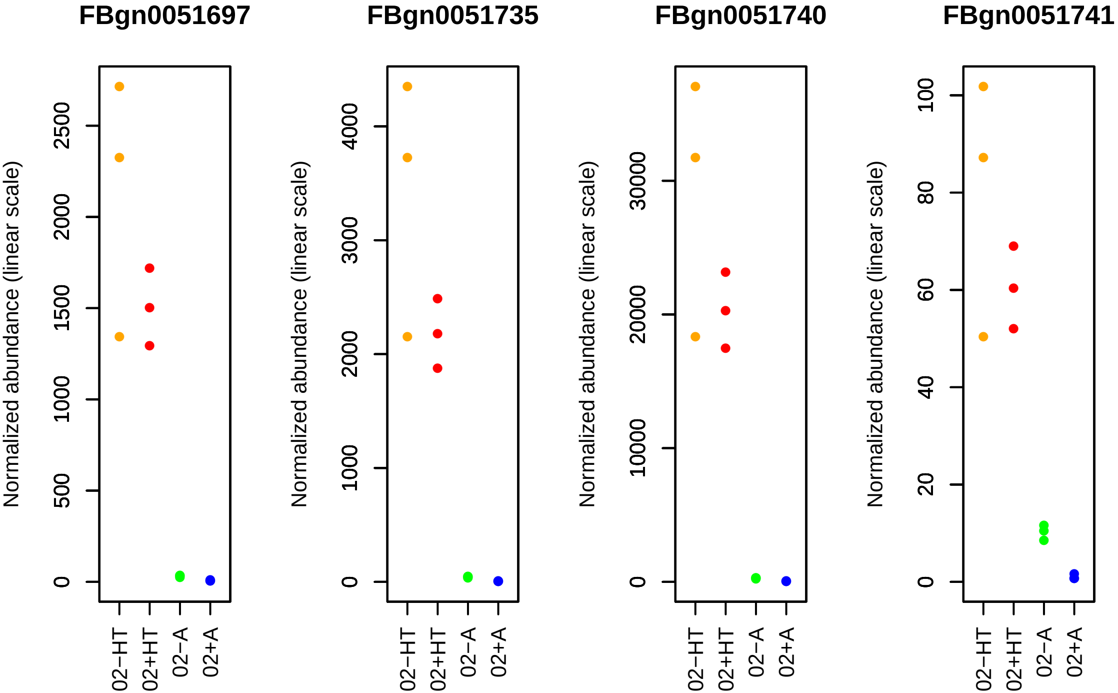
FBgn0051697
FBgn0051735
FBgn0051740
FBgn0051741
Normalized abundance (linear scale)
0
500
1000
1500
2000
2500
1000
2000
3000
4000
10000
20000
30000
20
40
60
80
100
02−HT
02+HT
02−A
02+A

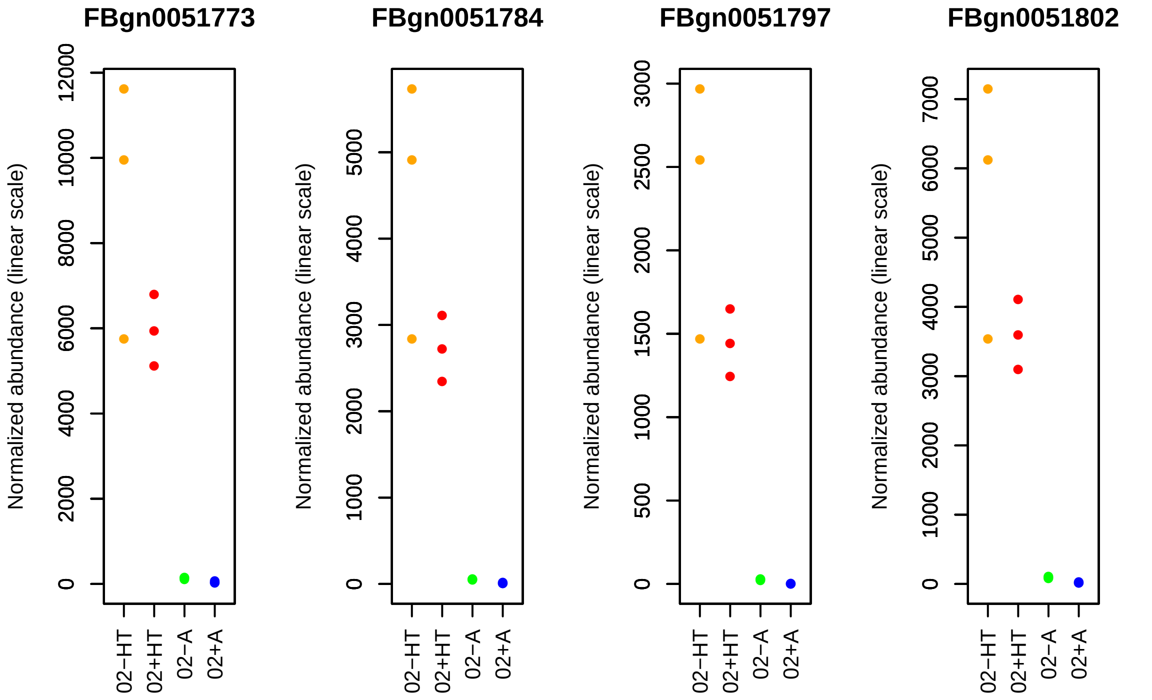
FBgn0051773
FBgn0051784
FBgn0051797
FBgn0051802
Normalized abundance (linear scale)
02−HT
02+HT
02−A
02+A

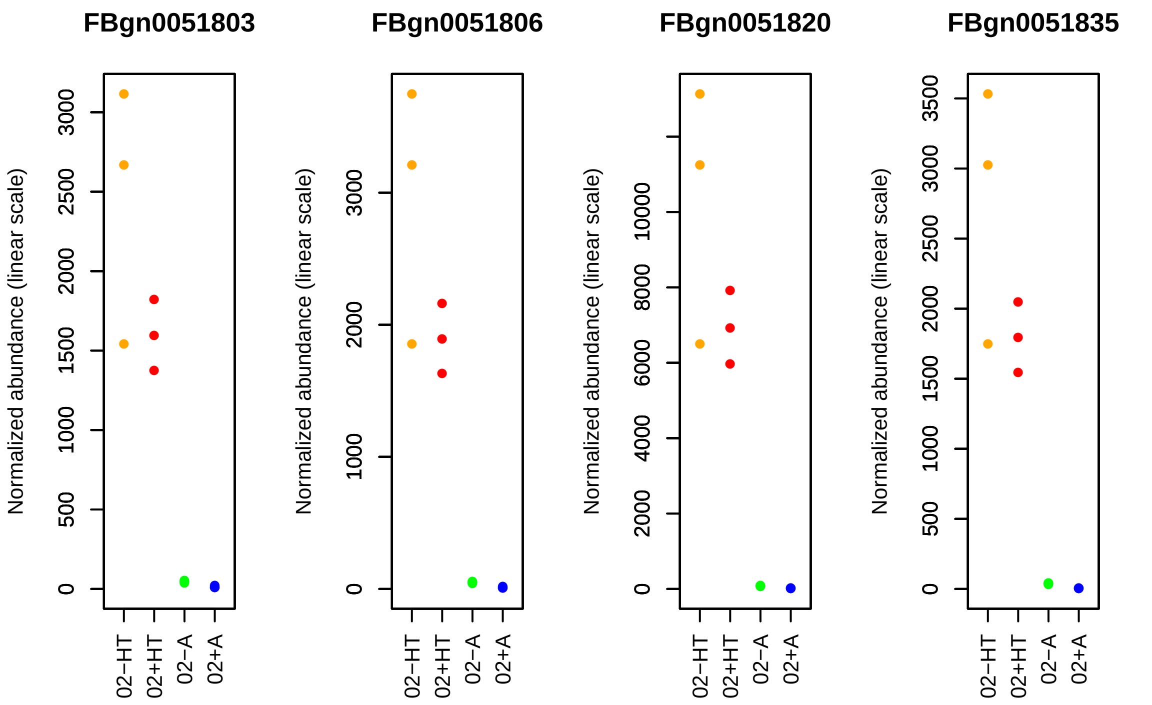

FBgn0051803
FBgn0051806
FBgn0051820
FBgn0051835
Normalized abundance (linear scale)
0
500
1000
1500
2000
2500
3000
3500
4000
6000
8000
10000
02−HT
02+HT
02−A
02+A

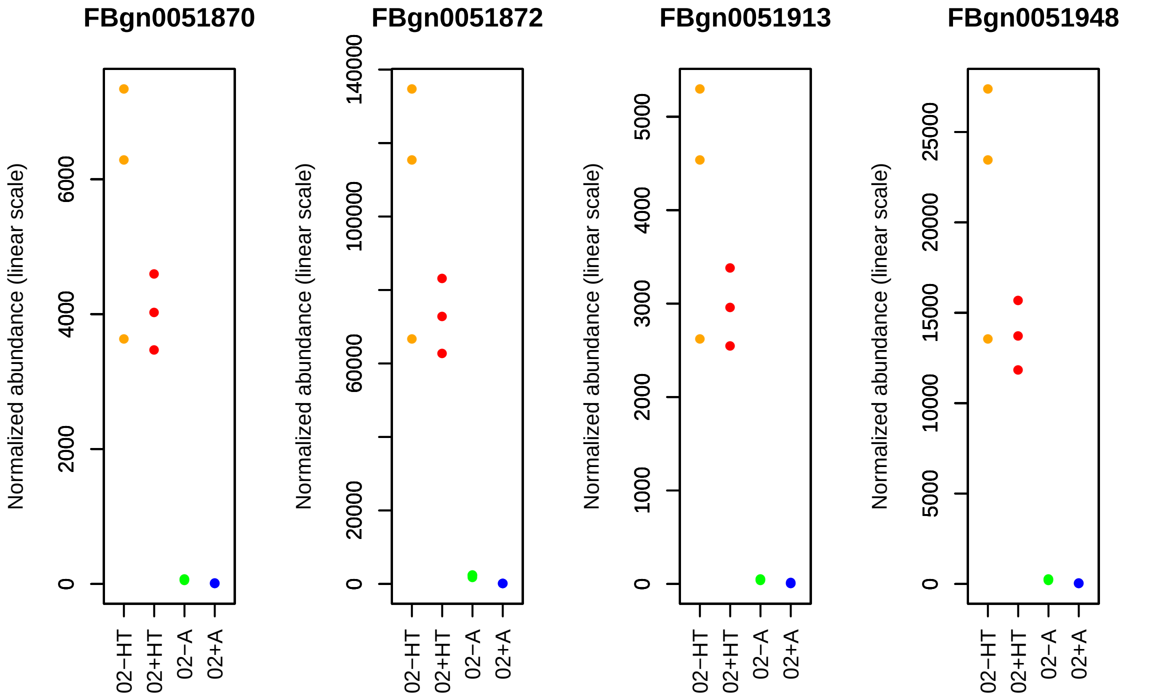

FBgn0051870
FBgn0051872
FBgn0051913
FBgn0051948
Normalized abundance (linear scale)
0
2000
4000
6000
20000
60000
100000
140000
1000
3000
5000
5000
10000
15000
20000
25000
02−HT
02+HT
02−A
02+A

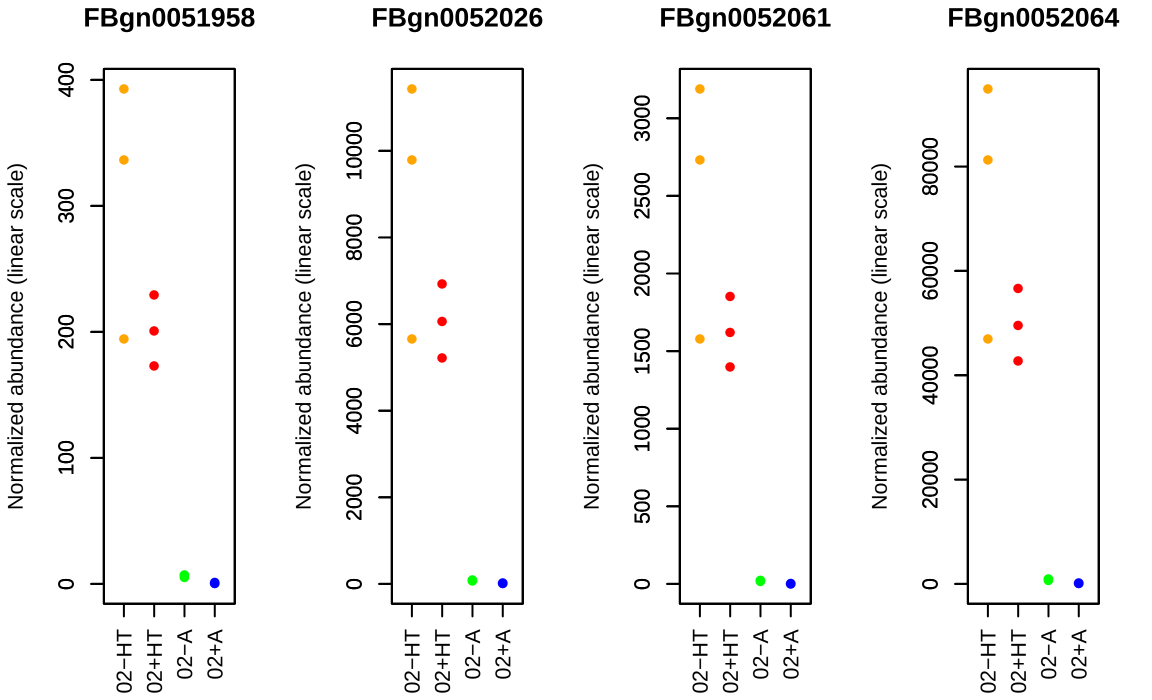

FBgn0051958
FBgn0052026
FBgn0052061
FBgn0052064
Normalized abundance (linear scale)
0
100
200
300
400
2000
4000
6000
8000
10000
500
1000
1500
2000
2500
3000
20000
40000
60000
80000
02−HT
02+HT
02−A
02+A

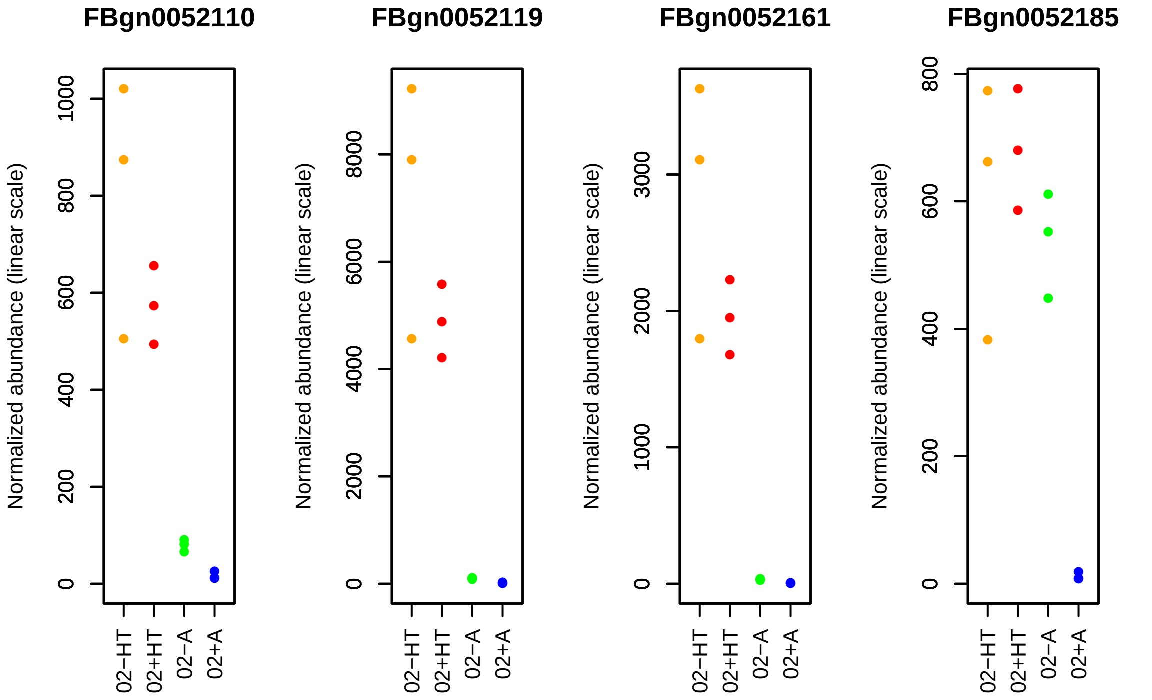

FBgn0052110
FBgn0052119
FBgn0052161
FBgn0052185
Normalized abundance (linear scale)
0
200
400
600
800
1000
2000
4000
6000
8000
3000
02−HT
02+HT
02−A
02+A

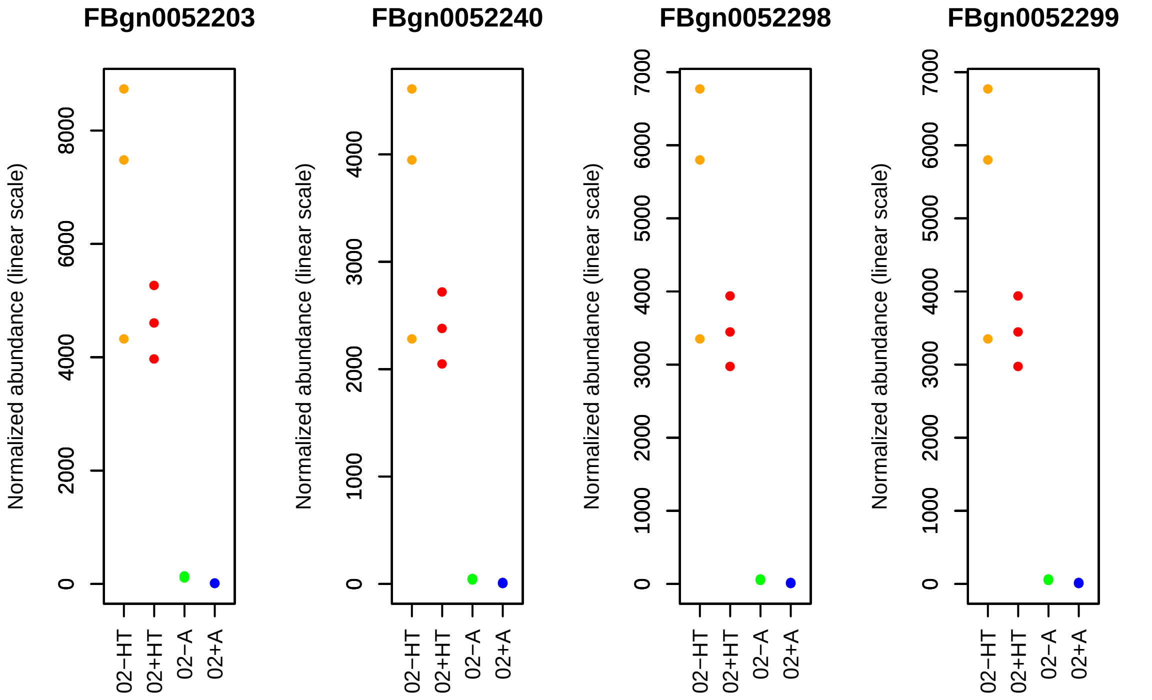

FBgn0052203
FBgn0052240
FBgn0052298
FBgn0052299
Normalized abundance (linear scale)
0
2000
4000
6000
8000
1000
3000
5000
7000
02−HT
02+HT
02−A
02+A

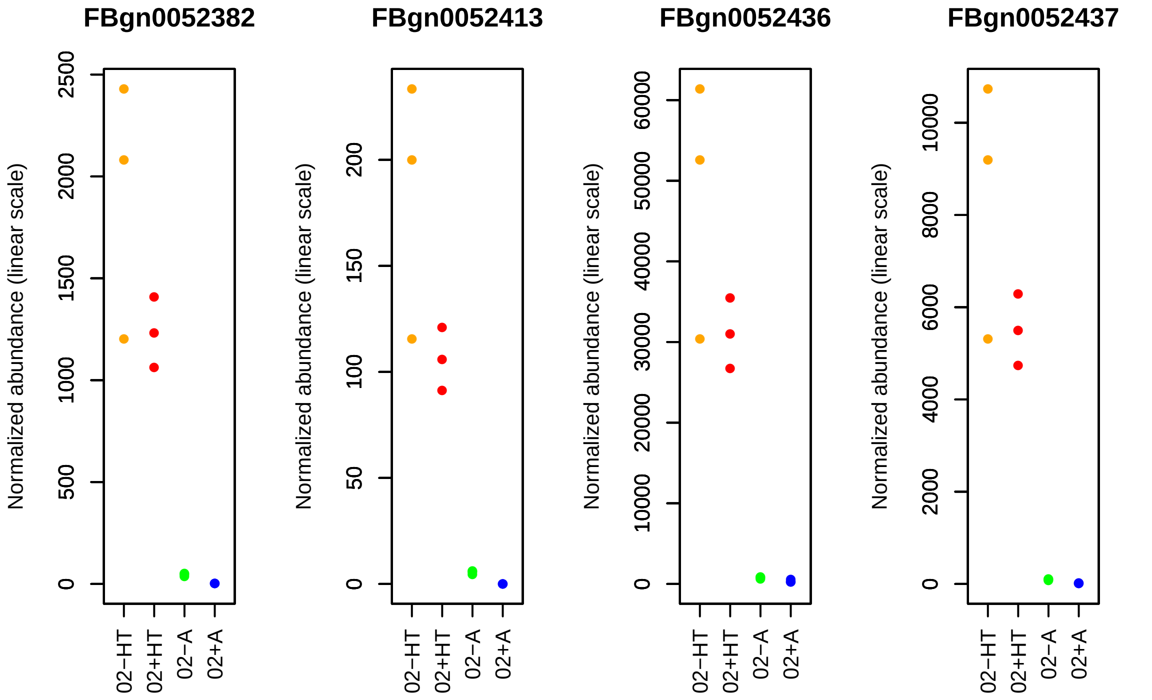

FBgn0052382
FBgn0052413
FBgn0052436
FBgn0052437
Normalized abundance (linear scale)
0
500
1000
1500
2000
2500
50
100
150
200
10000
20000
30000
40000
50000
60000
2000
4000
6000
8000
10000
02−HT
02+HT
02−A
02+A

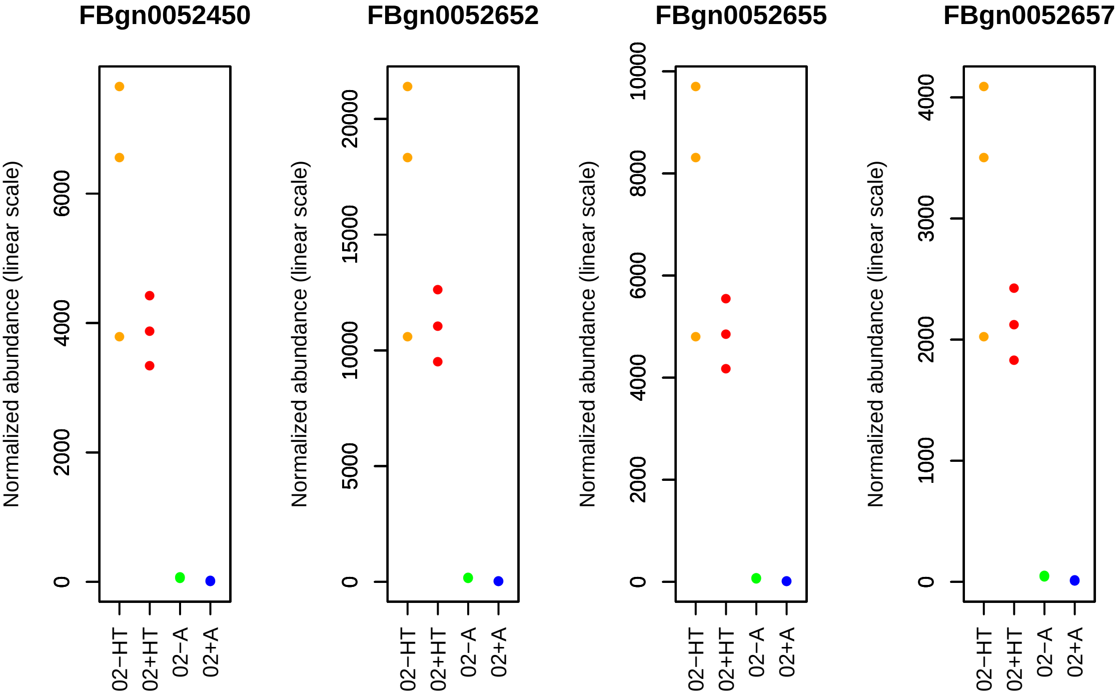
FBgn0052450
FBgn0052652
FBgn0052655
FBgn0052657
Normalized abundance (linear scale)
0
2000
4000
6000
5000
10000
15000
20000
8000
10000
1000
3000
02−HT
02+HT
02−A
02+A

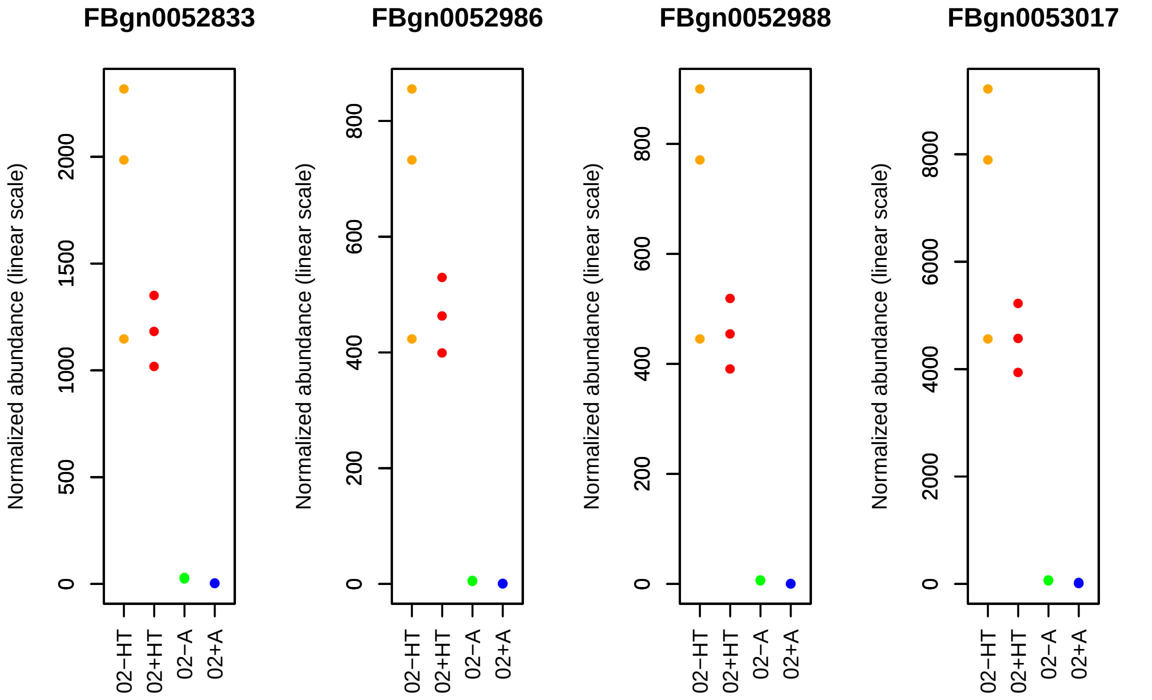
FBgn0052833
FBgn0052986
FBgn0052988
FBgn0053017
Normalized abundance (linear scale)
0
500
1000
1500
2000
200
400
600
800
2000
4000
6000
8000
02−HT
02+HT
02−A
02+A

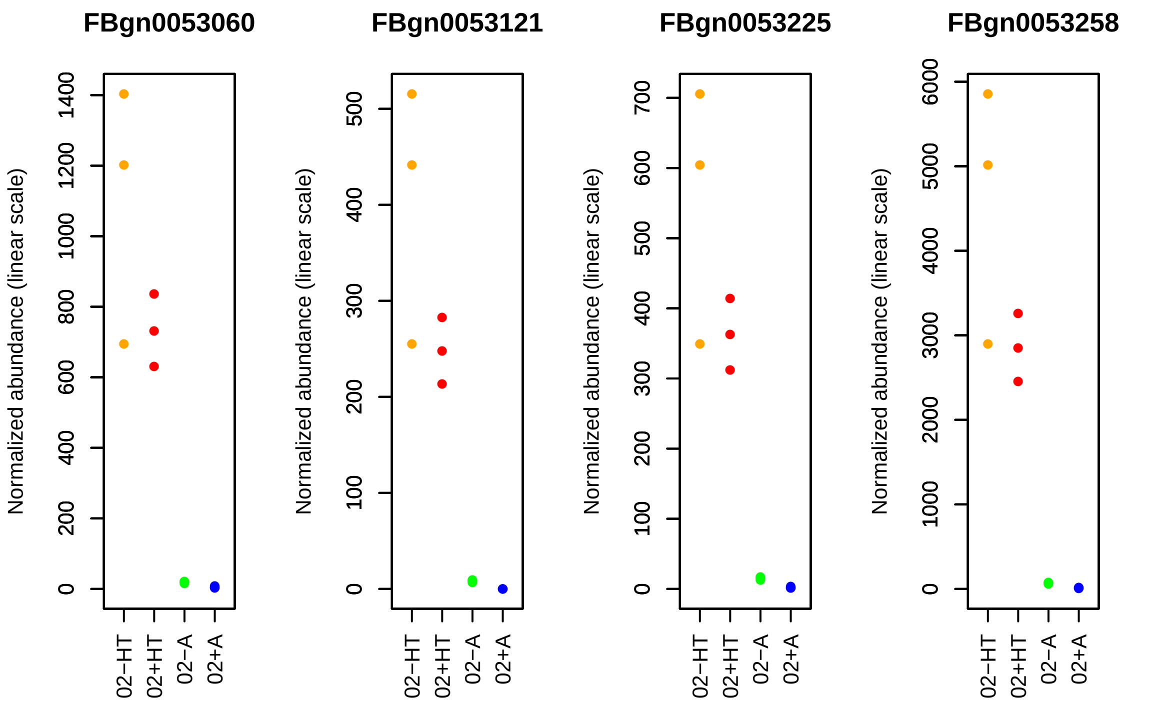

FBgn0053060
FBgn0053121
FBgn0053225
FBgn0053258
Normalized abundance (linear scale)
0
200
400
600
800
1000
1200
1400
100
300
500
700
2000
3000
4000
5000
6000
02−HT
02+HT
02−A
02+A

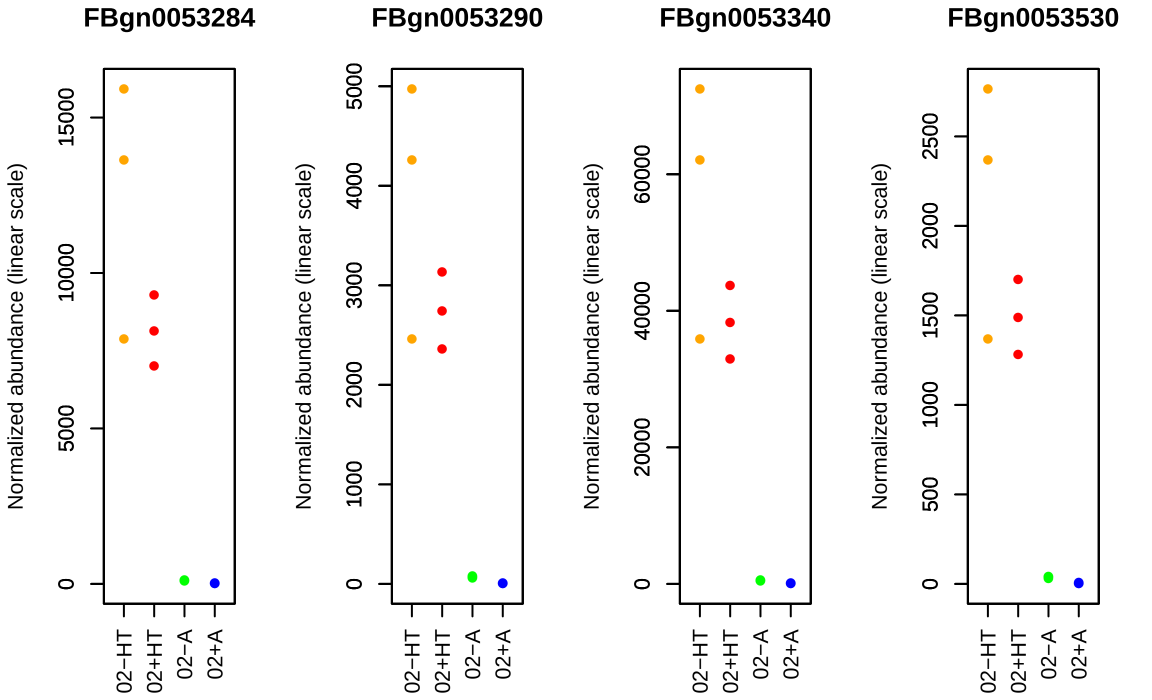
FBgn0053284
FBgn0053290
FBgn0053340
FBgn0053530
Normalized abundance (linear scale)
0
5000
10000
15000
1000
2000
3000
4000
5000
20000
40000
60000
500
1500
2500
02−HT
02+HT
02−A
02+A

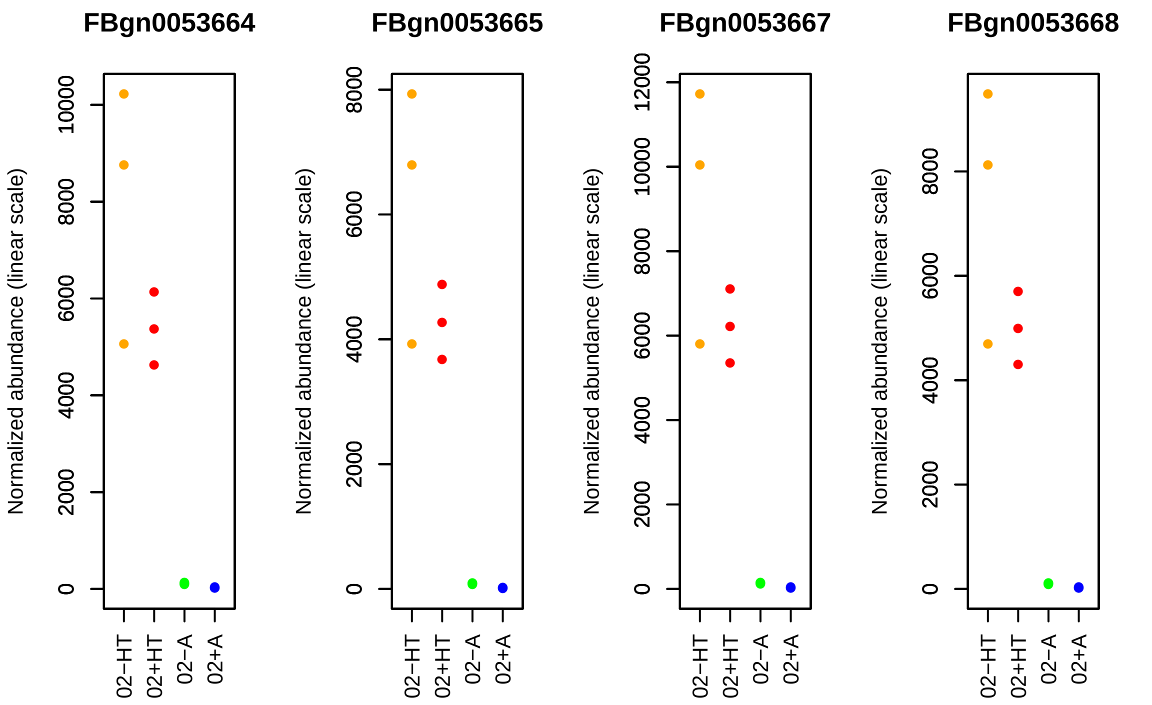
FBgn0053664
FBgn0053665
FBgn0053667
FBgn0053668
Normalized abundance (linear scale)
0
2000
4000
6000
8000
10000
12000
02−HT
02+HT
02−A
02+A

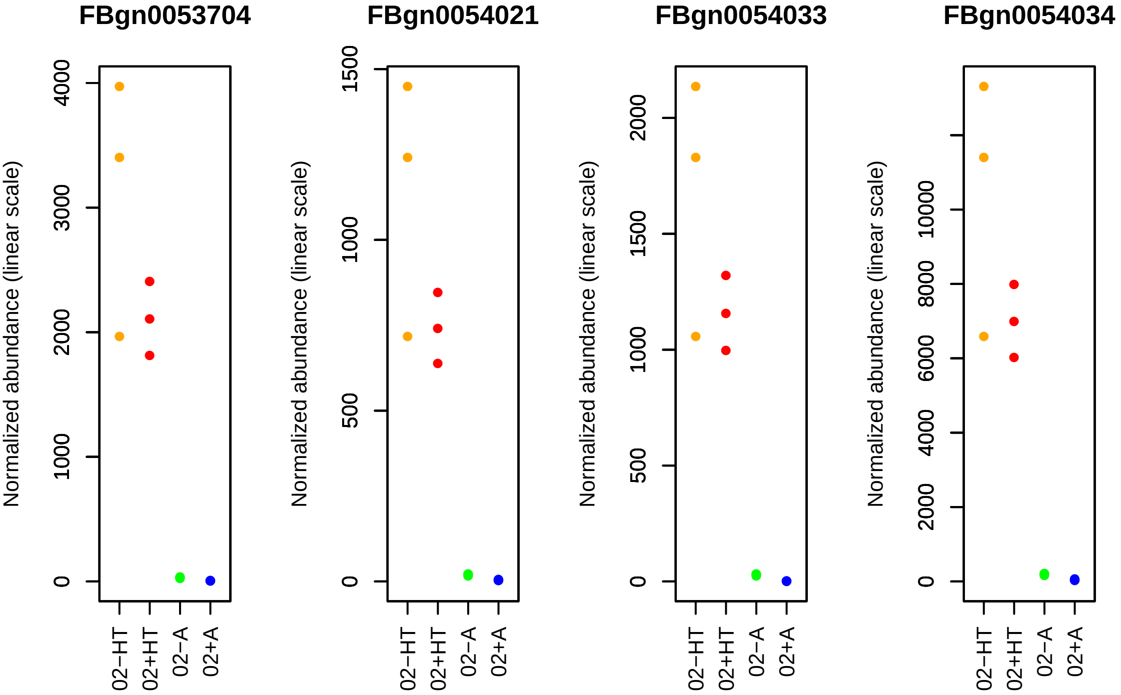
FBgn0053704
FBgn0054021
FBgn0054033
FBgn0054034
Normalized abundance (linear scale)
0
1000
2000
3000
4000
500
1500
6000
8000
10000
02−HT
02+HT
02−A
02+A

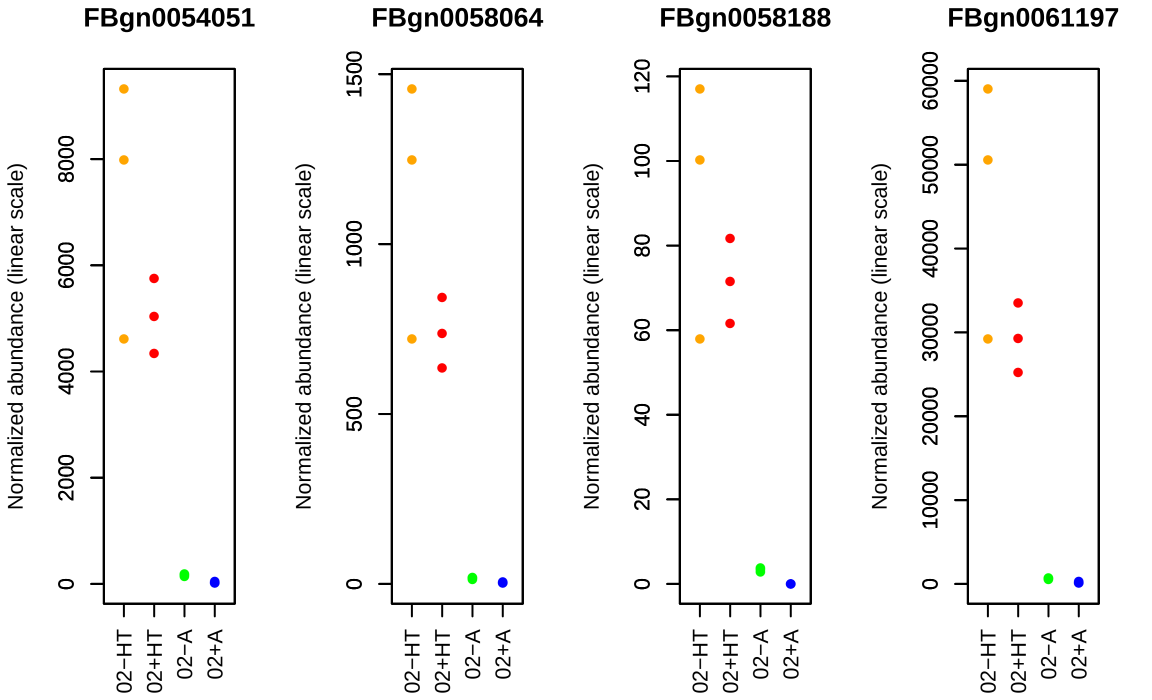

FBgn0054051
FBgn0058064
FBgn0058188
FBgn0061197
Normalized abundance (linear scale)
0
2000
4000
6000
8000
500
1000
1500
20
40
60
80
100
120
10000
20000
30000
40000
50000
60000
02−HT
02+HT
02−A
02+A

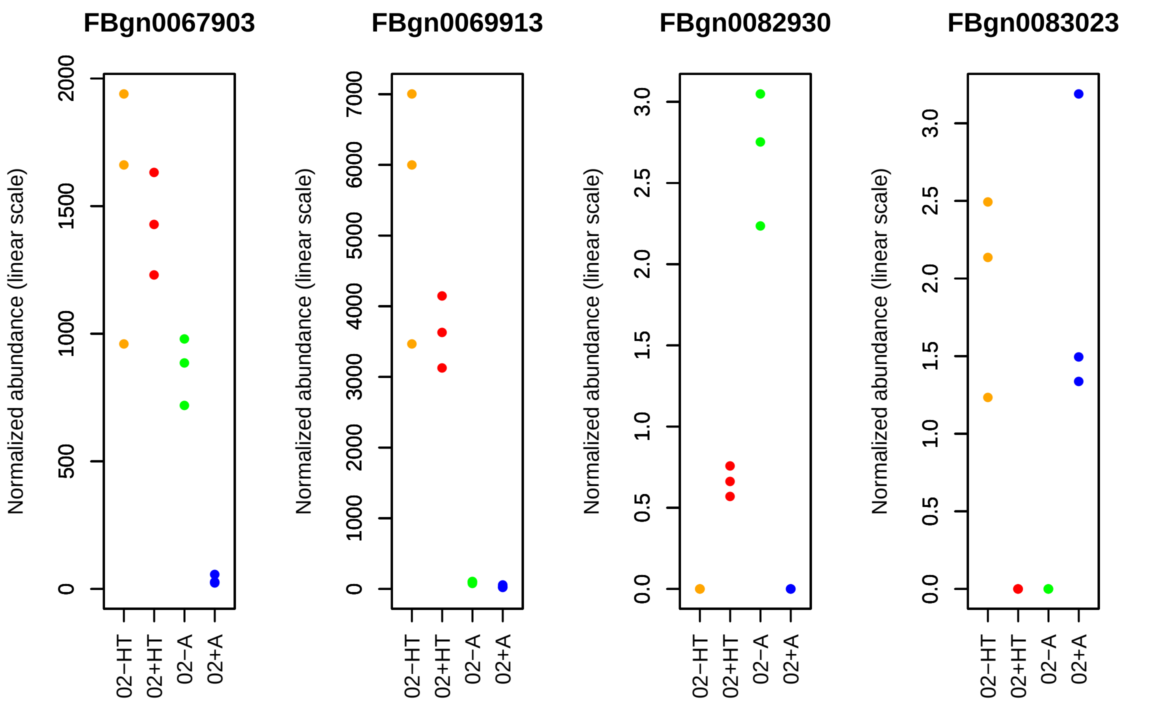

FBgn0067903
FBgn0069913
FBgn0082930
FBgn0083023
Normalized abundance (linear scale)
02−HT
02+HT
02−A
02+A

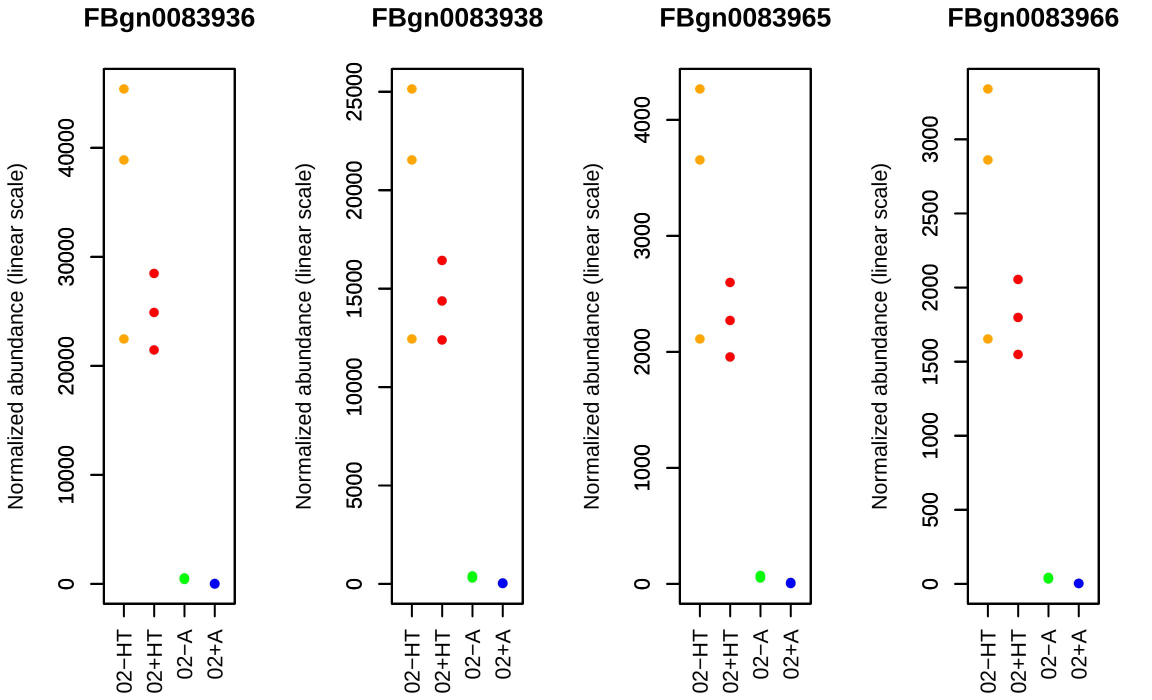
FBgn0083936
FBgn0083938
FBgn0083965
FBgn0083966
Normalized abundance (linear scale)
0
10000
20000
30000
40000
5000
15000
25000
1000
2000
3000
4000
500
1500
2500
02−HT
02+HT
02−A
02+A

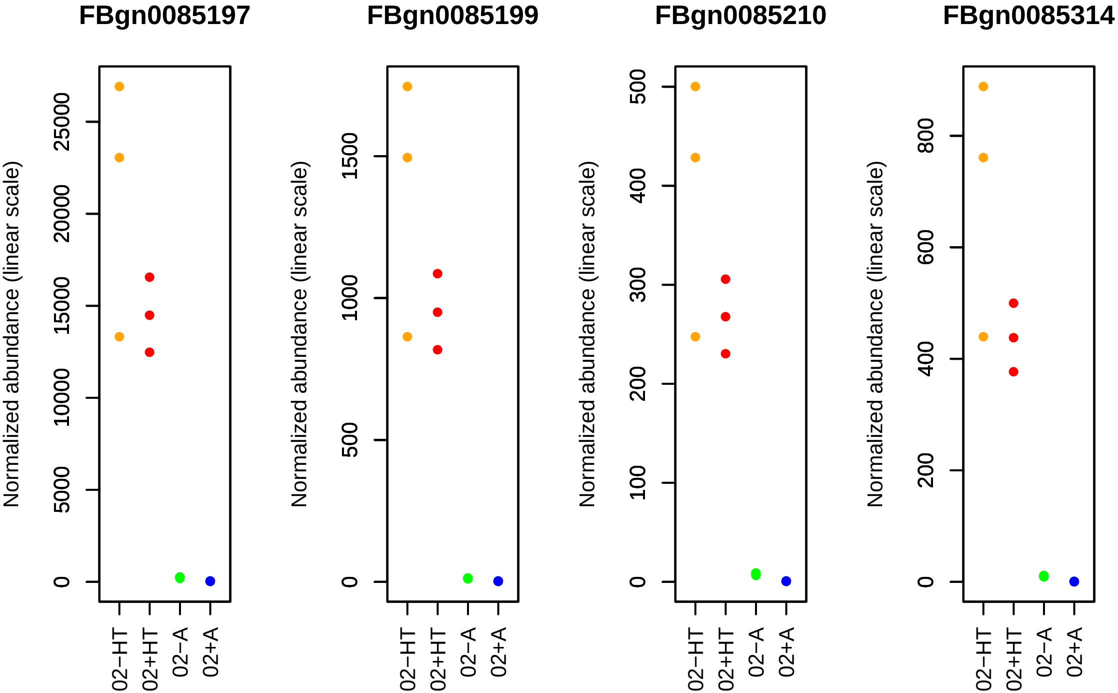
FBgn0085197
FBgn0085199
FBgn0085210
FBgn0085314
Normalized abundance (linear scale)
0
5000
10000
15000
20000
25000
500
1000
1500
100
200
300
400
600
800
02−HT
02+HT
02−A
02+A

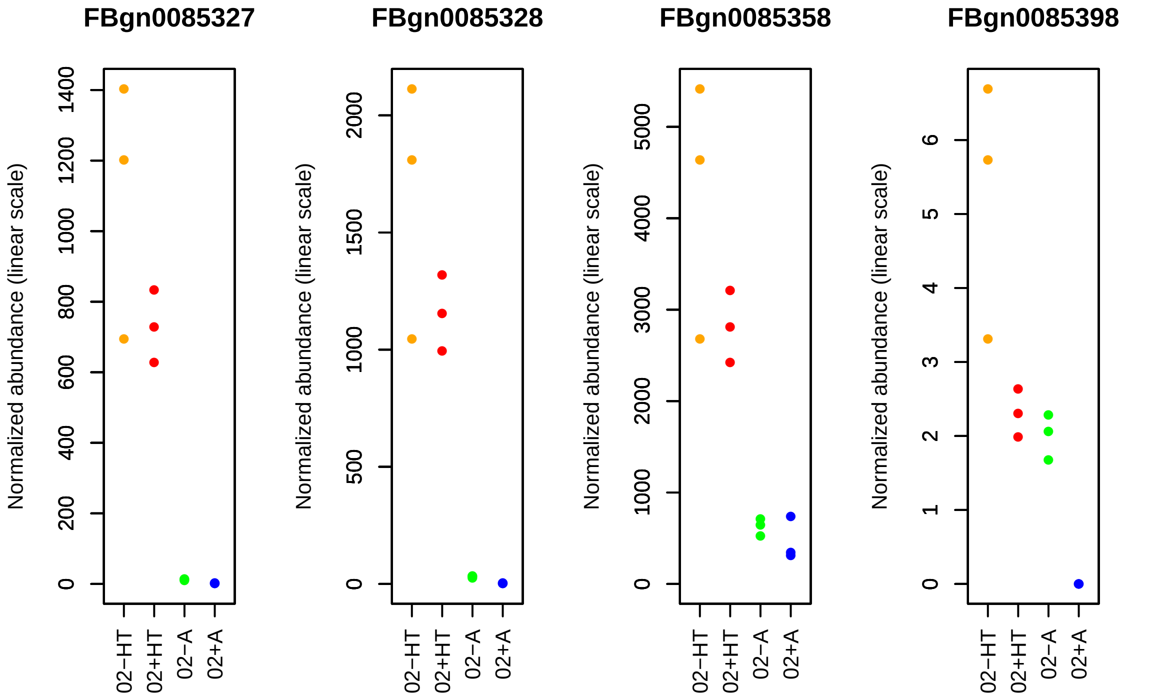

FBgn0085327
FBgn0085328
FBgn0085358
FBgn0085398
Normalized abundance (linear scale)
02−HT
02+HT
02−A
02+A

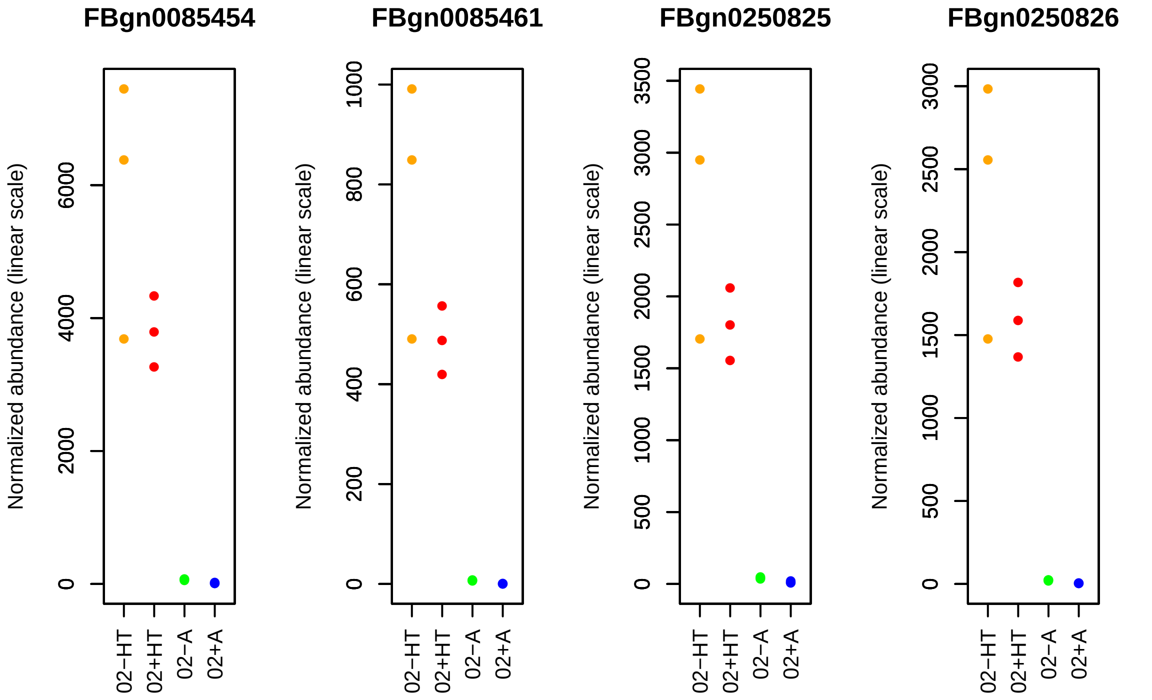

FBgn0085454
FBgn0085461
FBgn0250825
FBgn0250826
Normalized abundance (linear scale)
02−HT
02+HT
02−A
02+A

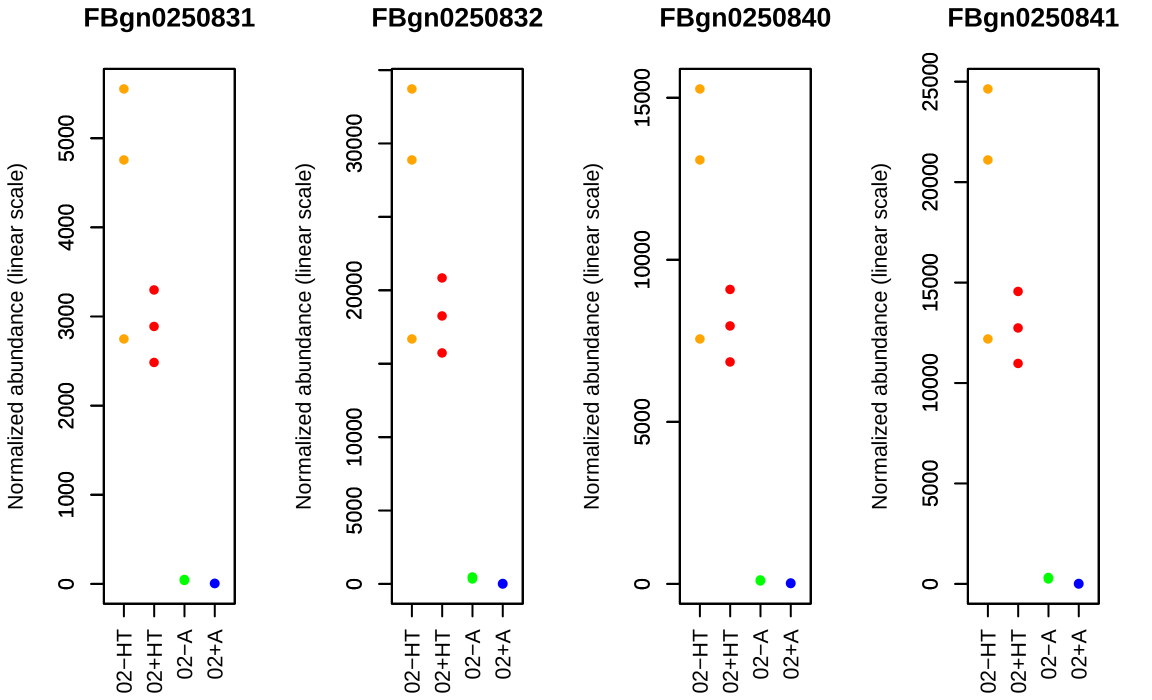

FBgn0250831
FBgn0250832
FBgn0250840
FBgn0250841
Normalized abundance (linear scale)
0
1000
2000
3000
4000
5000
5000
10000
15000
20000
25000
30000
02−HT
02+HT
02−A
02+A

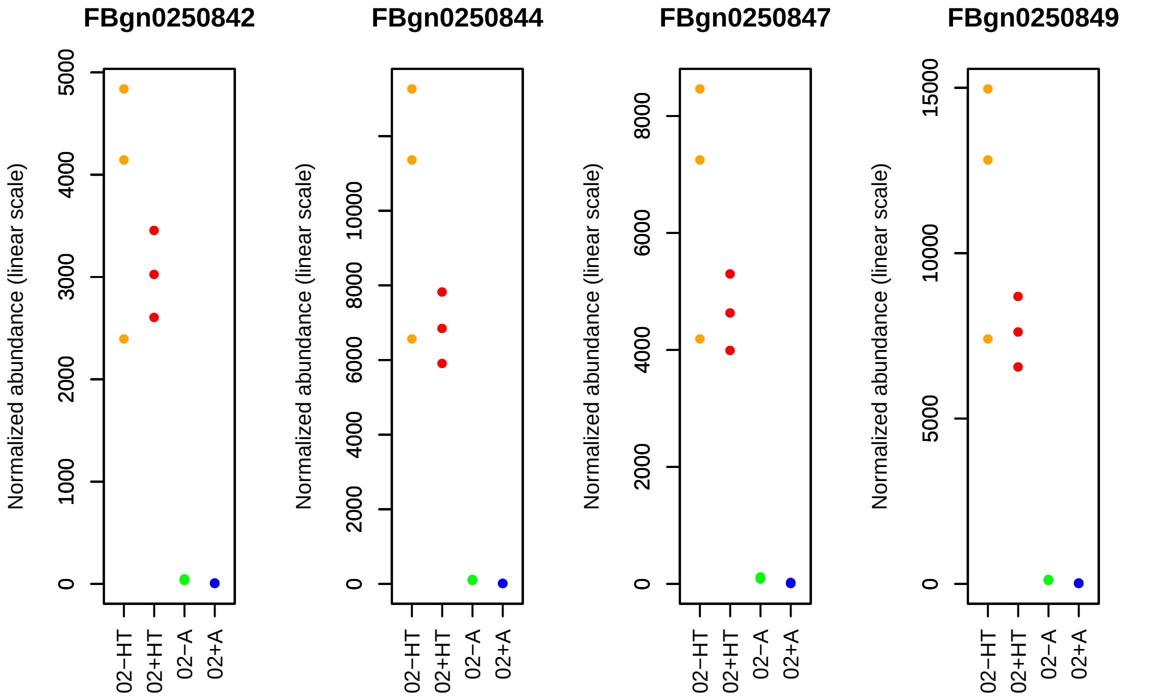
FBgn0250842
FBgn0250844
FBgn0250847
FBgn0250849
Normalized abundance (linear scale)
0
1000
2000
3000
4000
5000
6000
8000
10000
15000
02−HT
02+HT
02−A
02+A

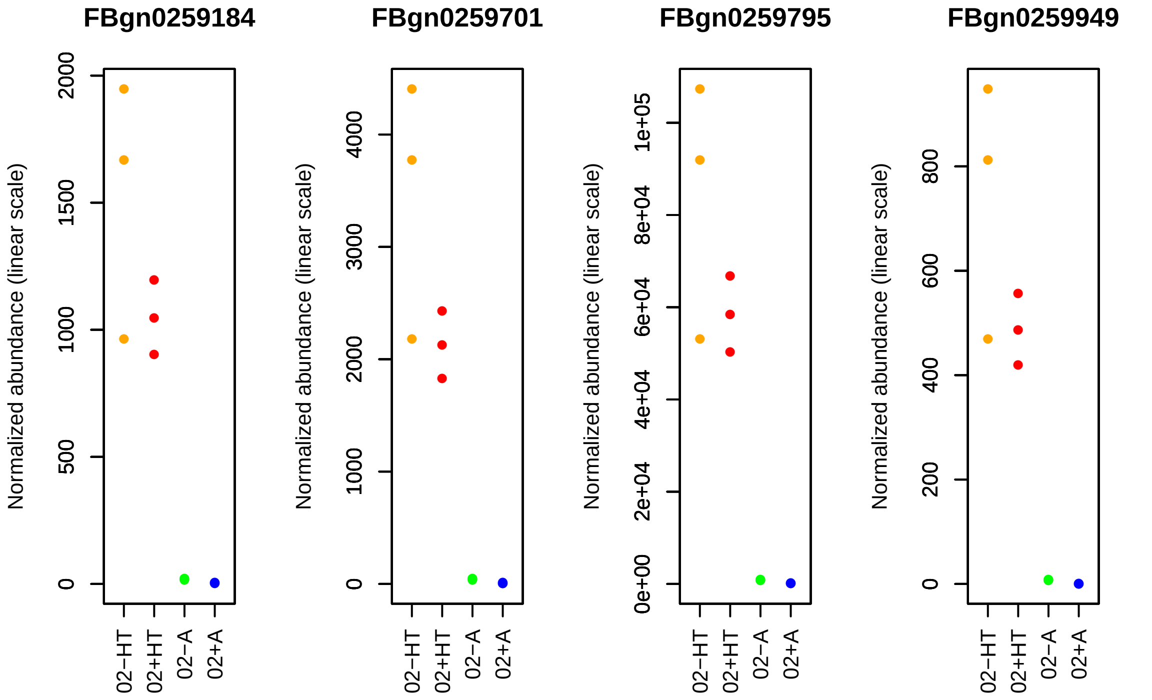
FBgn0259184
FBgn0259701
FBgn0259795
FBgn0259949
Normalized abundance (linear scale)
0
500
1000
1500
2000
1000
2000
3000
4000
0e+00
2e+04
4e+04
6e+04
8e+04
1e+05
200
400
600
800
02−HT
02+HT
02−A
02+A

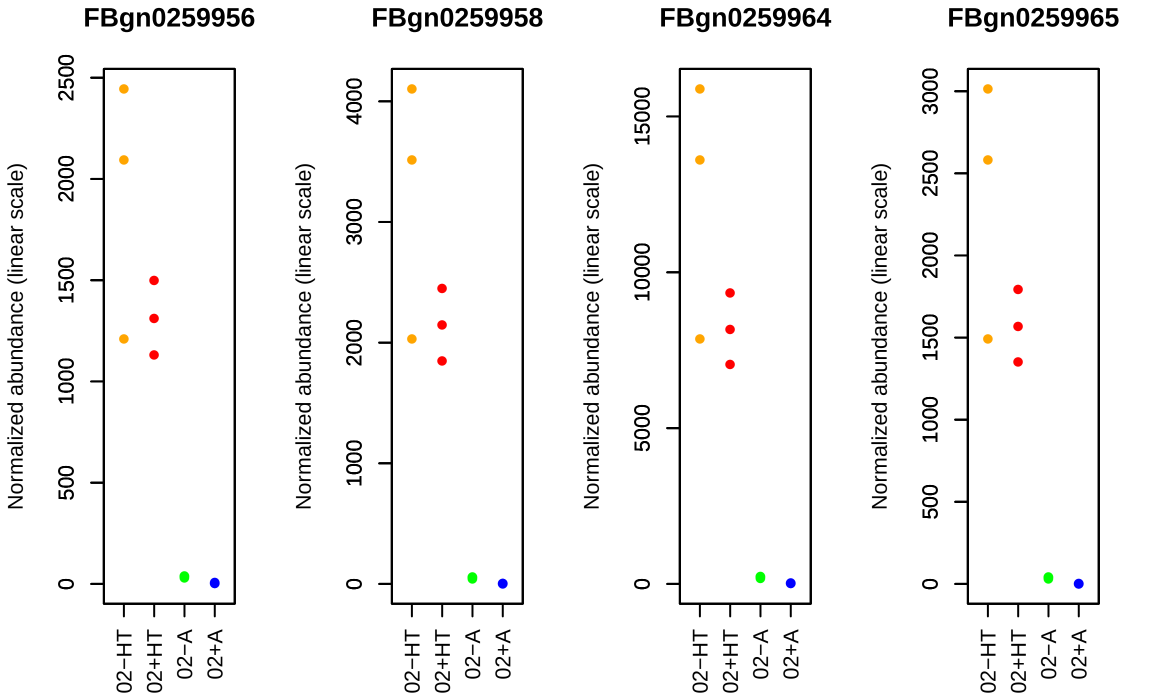

FBgn0259956
FBgn0259958
FBgn0259964
FBgn0259965
Normalized abundance (linear scale)
0
500
1000
1500
2000
2500
3000
4000
5000
10000
15000
02−HT
02+HT
02−A
02+A

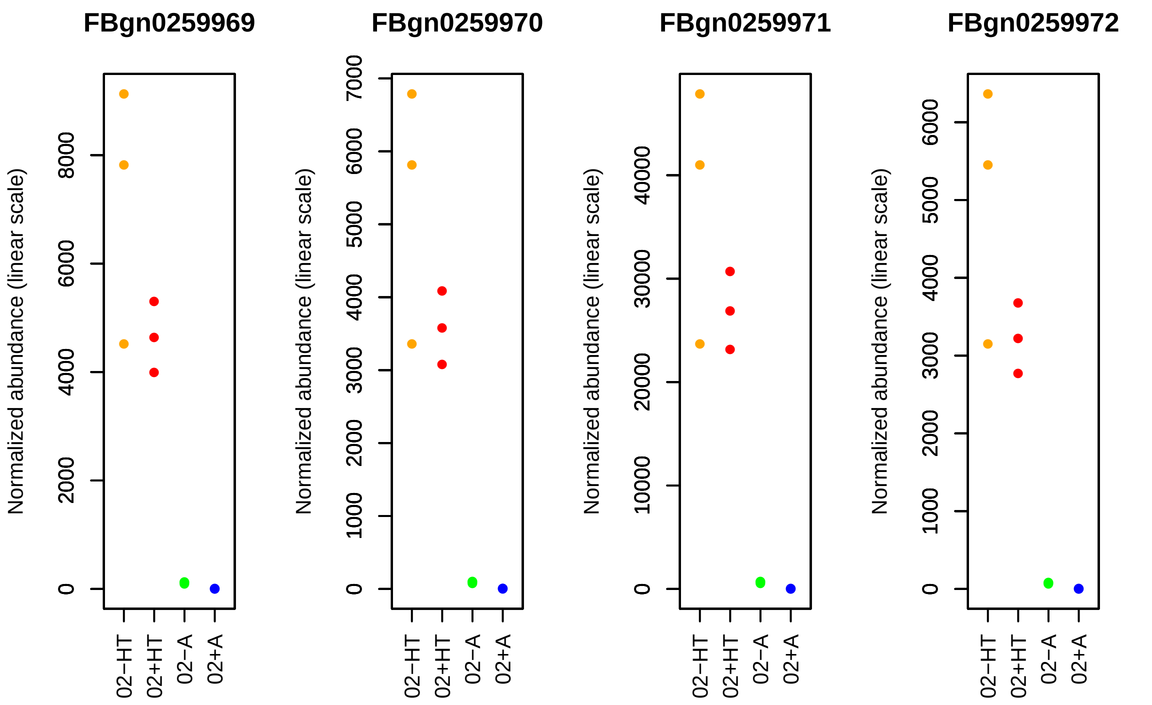

FBgn0259969
FBgn0259970
FBgn0259971
FBgn0259972
Normalized abundance (linear scale)
0
2000
4000
6000
8000
1000
3000
5000
7000
10000
20000
30000
40000
02−HT
02+HT
02−A
02+A

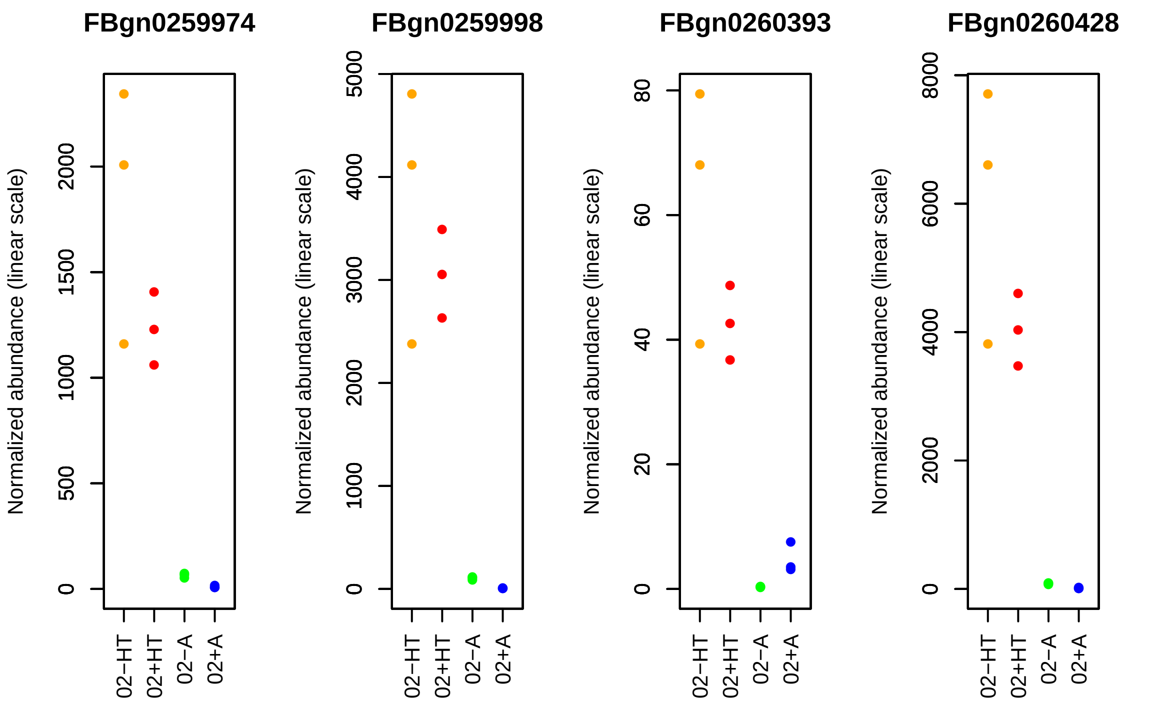

FBgn0259974
FBgn0259998
FBgn0260393
FBgn0260428
Normalized abundance (linear scale)
0
500
1000
1500
2000
1000
2000
3000
4000
5000
20
40
60
80
6000
8000
02−HT
02+HT
02−A
02+A

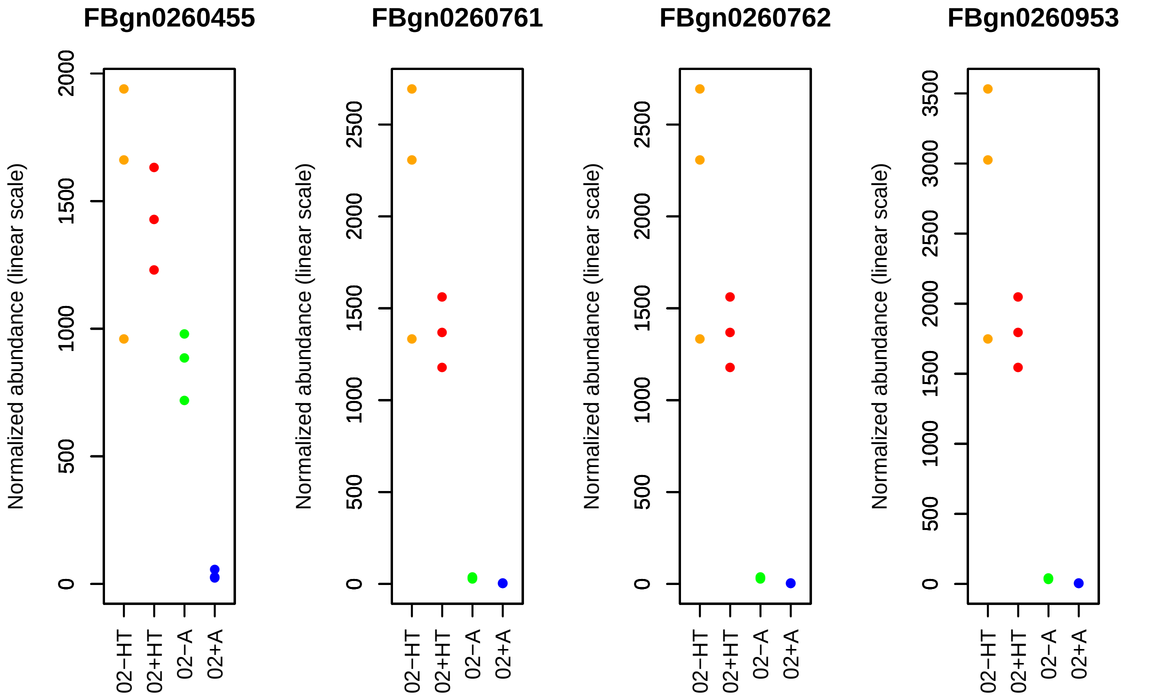

FBgn0260455
FBgn0260761
FBgn0260762
FBgn0260953
Normalized abundance (linear scale)
0
500
1000
1500
2000
2500
3000
3500
02−HT
02+HT
02−A
02+A

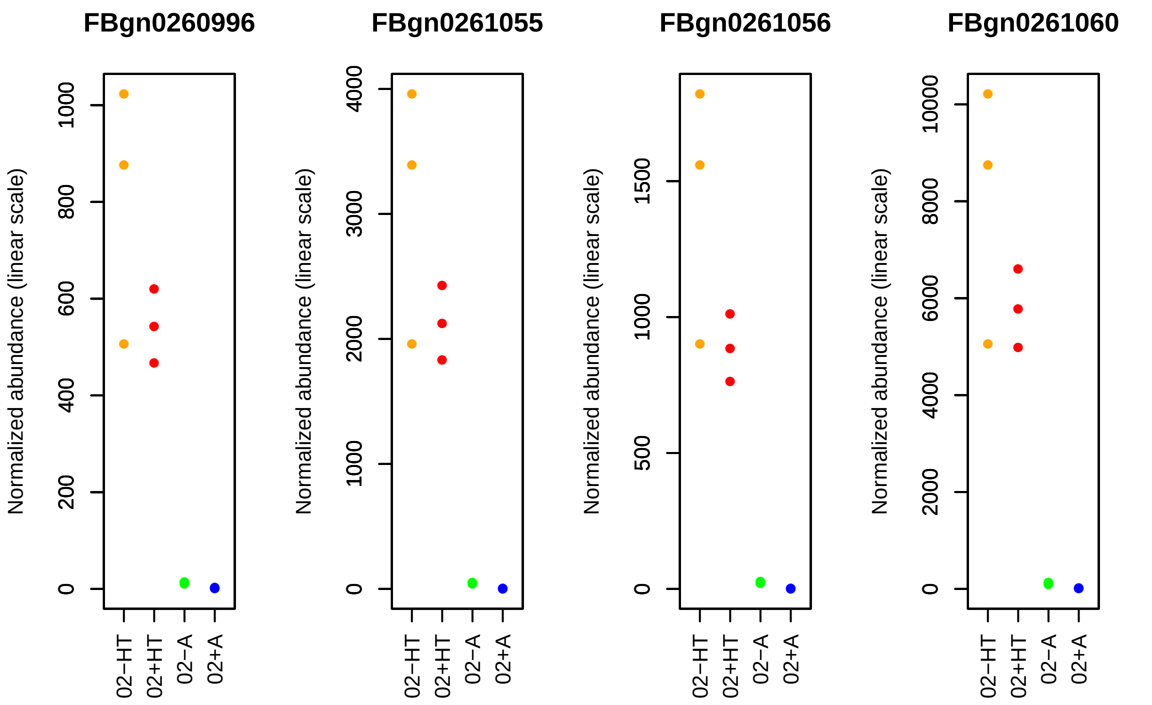
FBgn0260996
FBgn0261055
FBgn0261056
FBgn0261060
Normalized abundance (linear scale)
0
200
400
600
800
1000
1000
2000
3000
4000
500
1500
2000
4000
6000
8000
10000
02−HT
02+HT
02−A
02+A

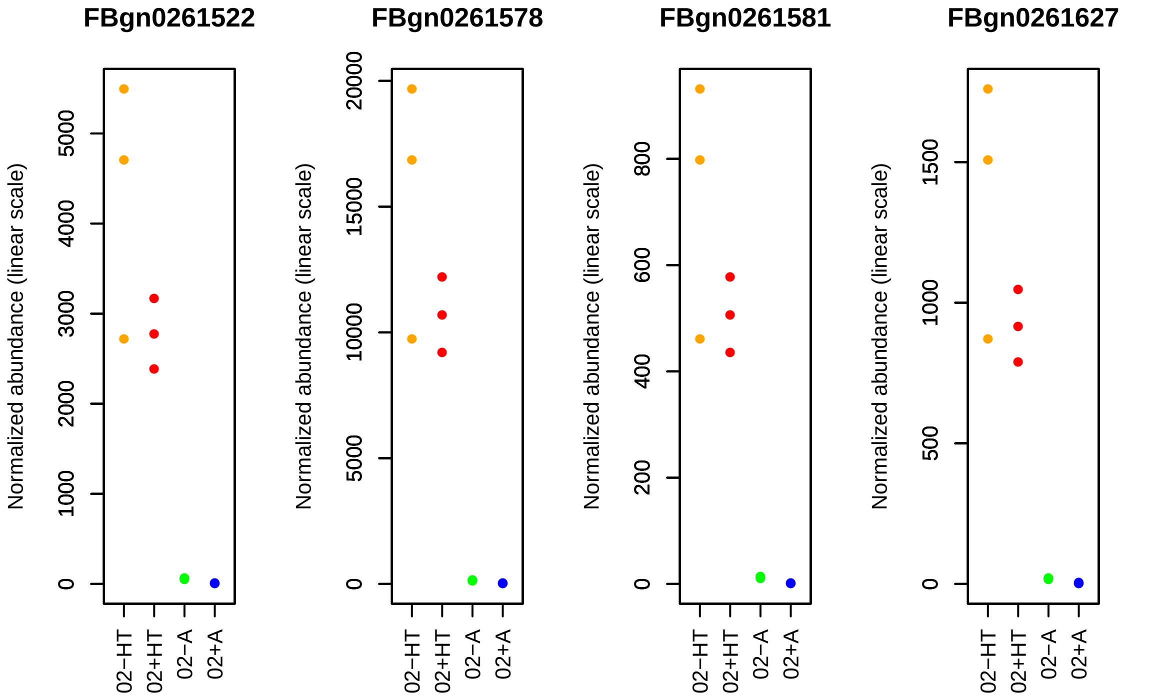

FBgn0261522
FBgn0261578
FBgn0261581
FBgn0261627
Normalized abundance (linear scale)
0
1000
2000
3000
4000
5000
5000
10000
15000
20000
200
400
600
800
500
1000
1500
02−HT
02+HT
02−A
02+A

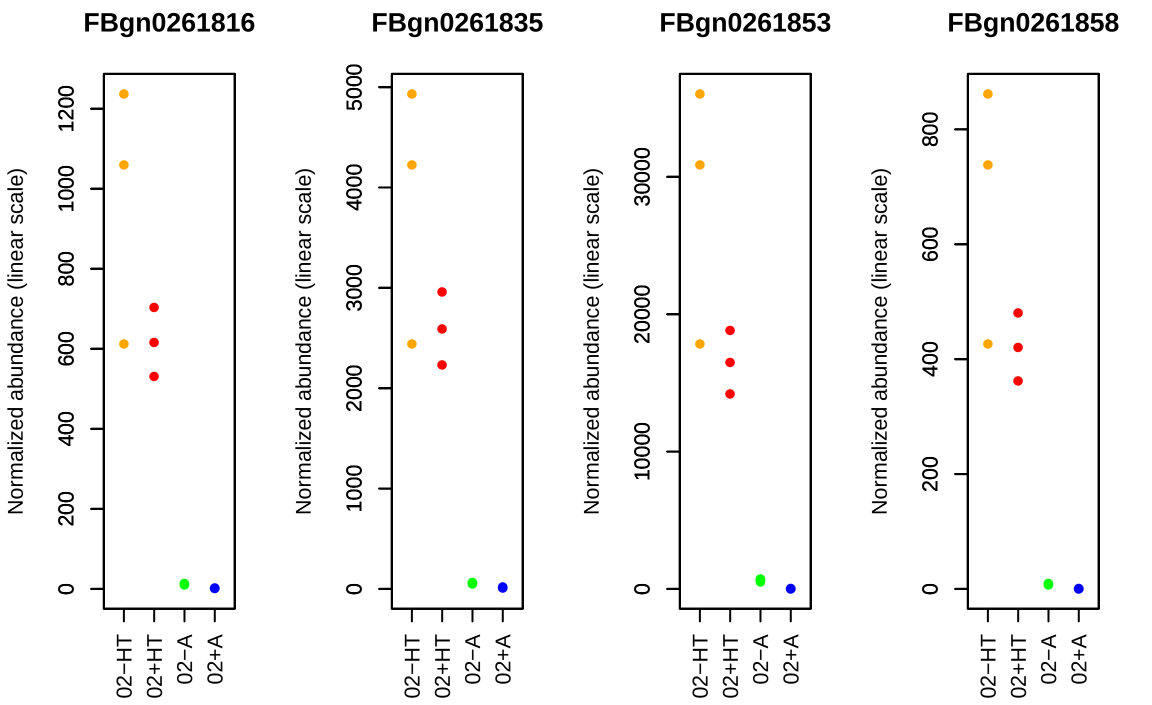

FBgn0261816
FBgn0261835
FBgn0261853
FBgn0261858
Normalized abundance (linear scale)
0
200
400
600
800
1000
1200
1000
2000
3000
4000
5000
10000
20000
30000
02−HT
02+HT
02−A
02+A

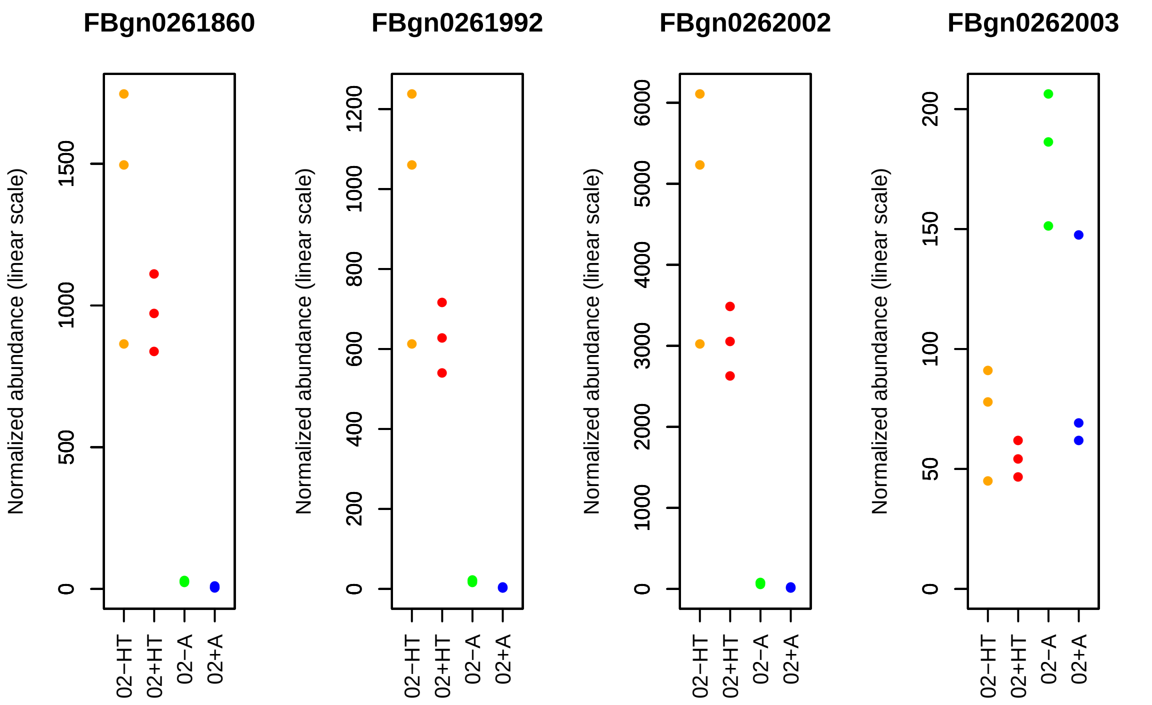
FBgn0261860
FBgn0261992
FBgn0262002
FBgn0262003
Normalized abundance (linear scale)
0
500
1000
1500
200
400
600
800
1200
2000
3000
4000
5000
6000
50
100
150
02−HT
02+HT
02−A
02+A

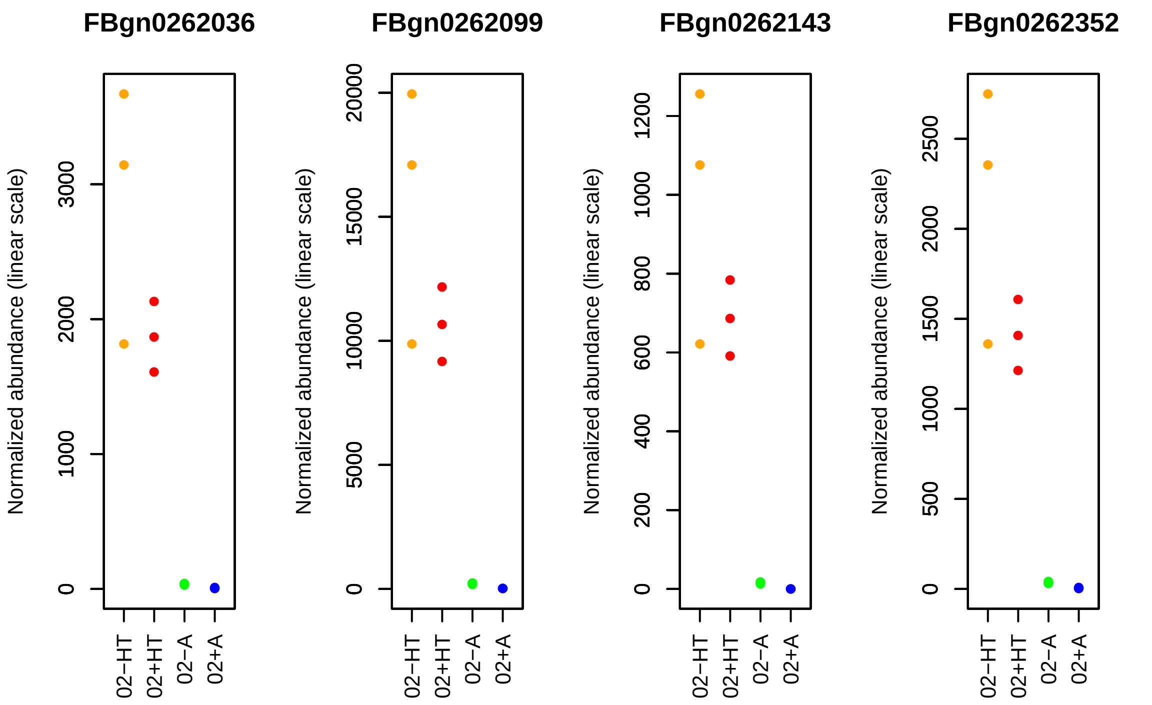
FBgn0262036
FBgn0262099
FBgn0262143
FBgn0262352
Normalized abundance (linear scale)
0
1000
2000
3000
5000
10000
15000
20000
200
400
600
800
1000
1200
500
1500
2000
2500
02−HT
02+HT
02−A
02+A

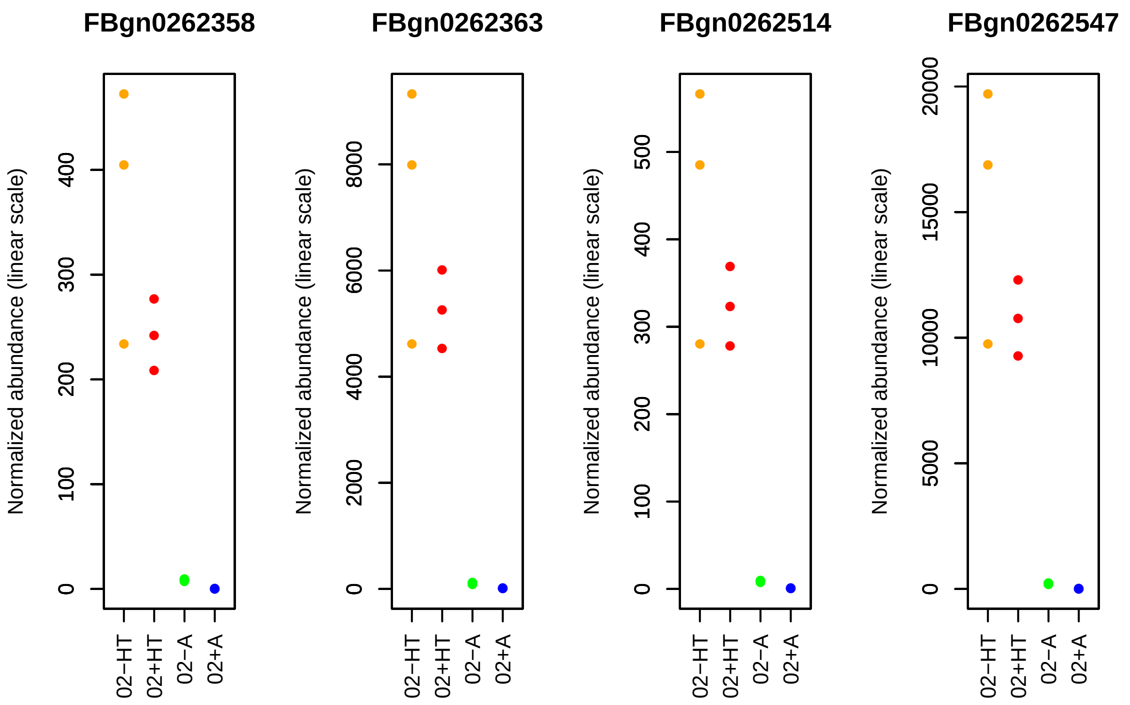

FBgn0262358
FBgn0262363
FBgn0262514
FBgn0262547
Normalized abundance (linear scale)
0
100
200
300
400
0
2000
4000
6000
8000
500
5000
10000
15000
20000
02−HT
02+HT
02−A
02+A

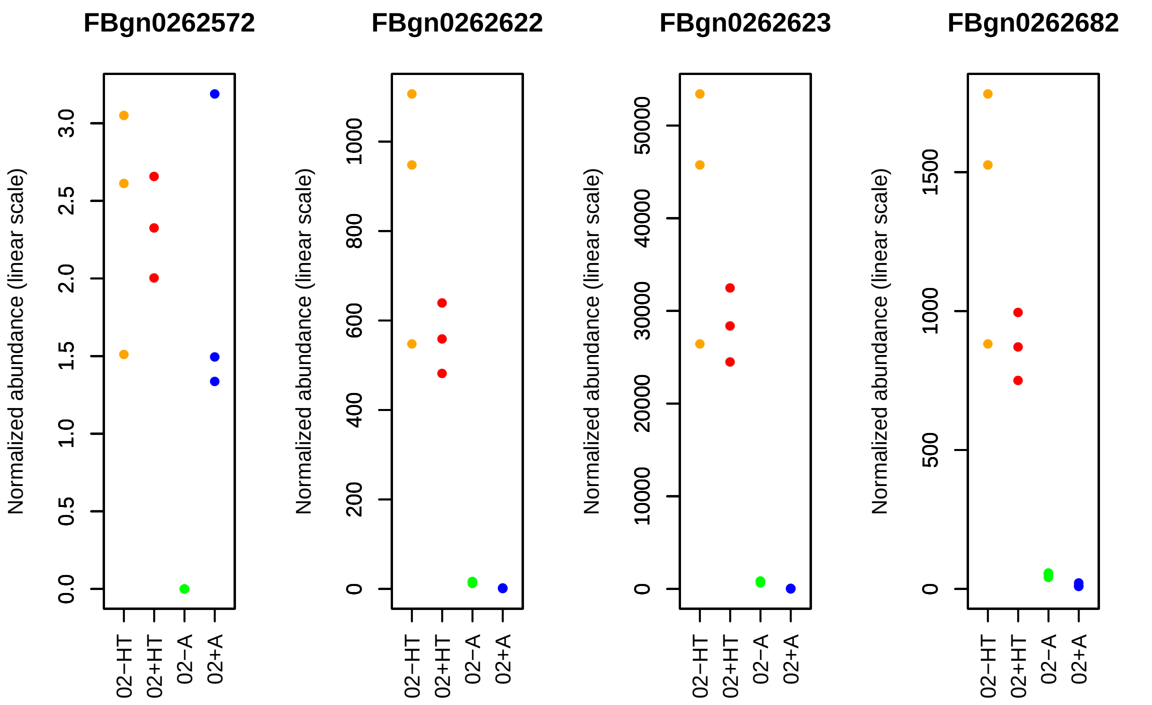
FBgn0262572
FBgn0262622
FBgn0262623
FBgn0262682
Normalized abundance (linear scale)
0.0
0.5
1.0
1.5
2.0
2.5
3.0
0
200
400
600
800
1000
0
10000
20000
30000
40000
50000
0
500
1000
1500
02−HT
02+HT
02−A
02+A

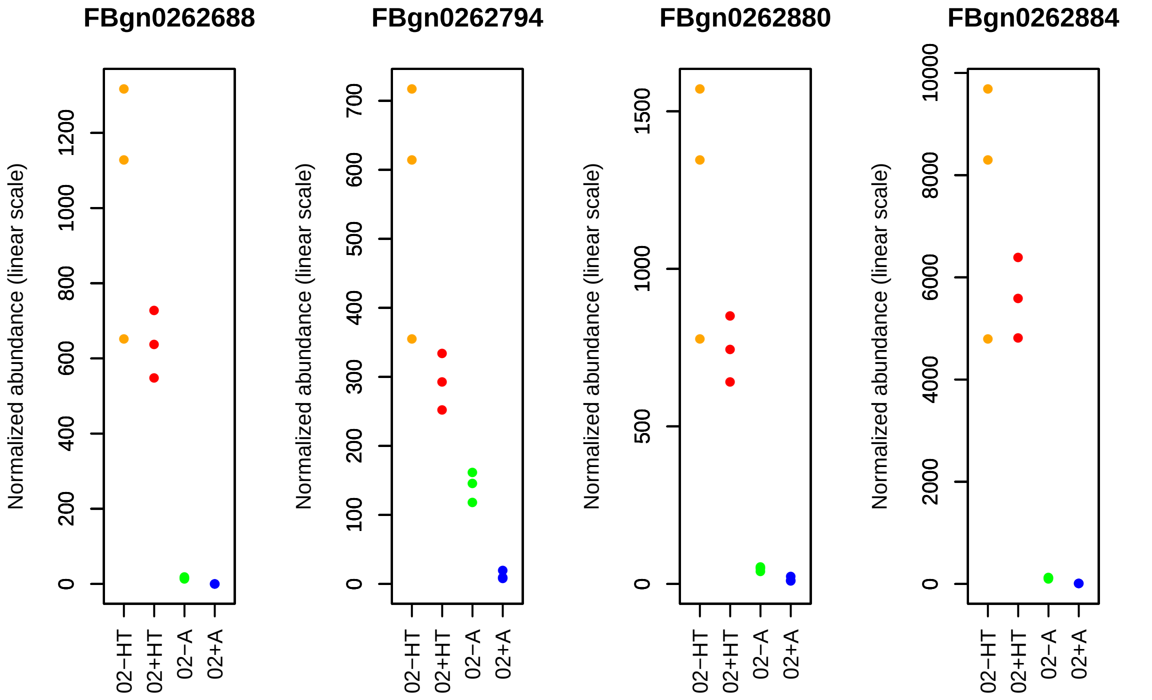

FBgn0262688
FBgn0262794
FBgn0262880
FBgn0262884
Normalized abundance (linear scale)
0
200
400
600
800
1000
1200
100
300
500
700
1500
2000
4000
6000
8000
10000
02−HT
02+HT
02−A
02+A

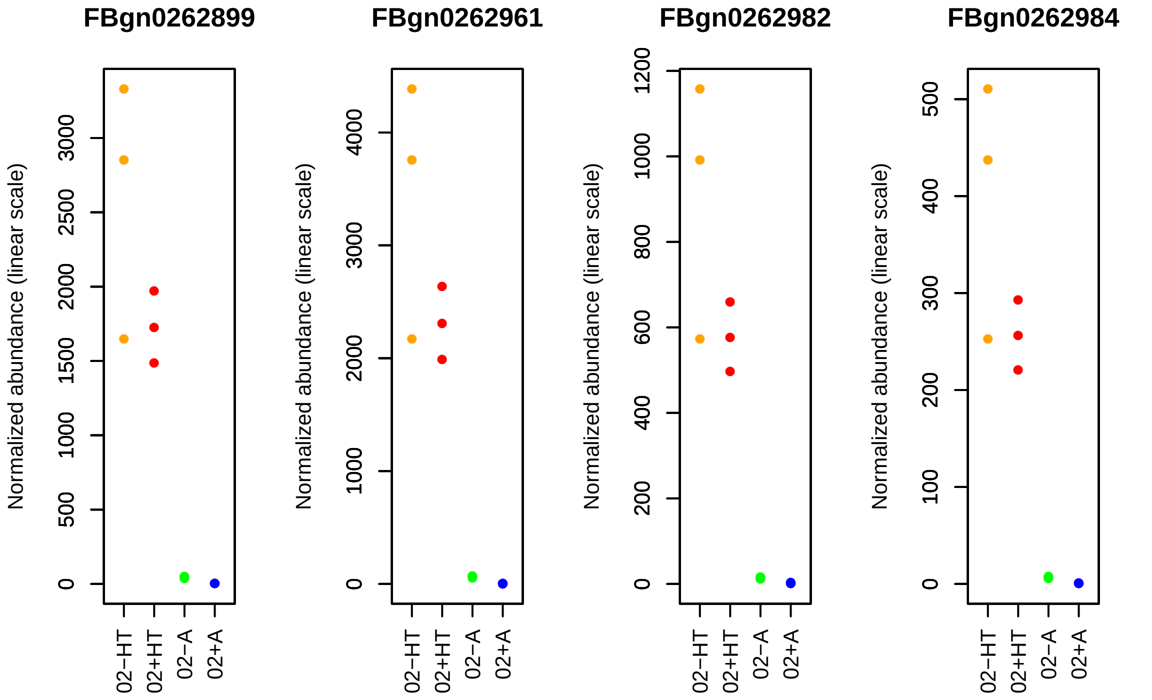
FBgn0262899
FBgn0262961
FBgn0262982
FBgn0262984
Normalized abundance (linear scale)
02−HT
02+HT
02−A
02+A

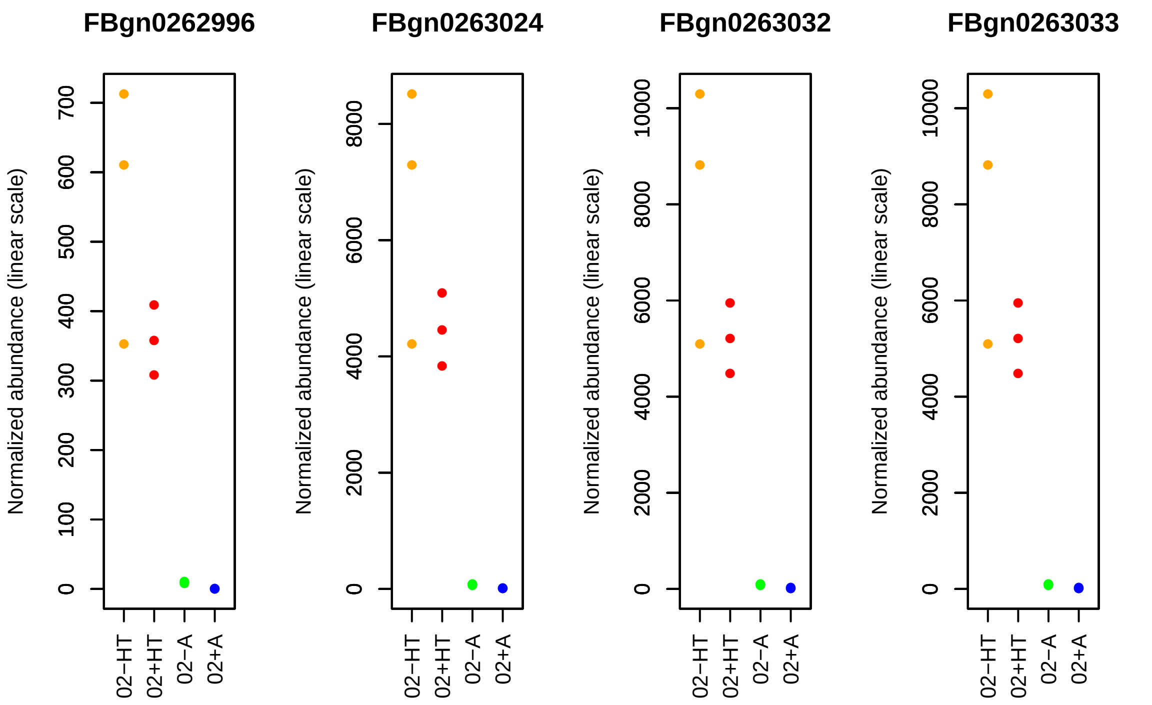
FBgn0262996
FBgn0263024
FBgn0263032
FBgn0263033
Normalized abundance (linear scale)
0
100
200
300
400
500
600
700
2000
4000
6000
8000
10000
02−HT
02+HT
02−A
02+A

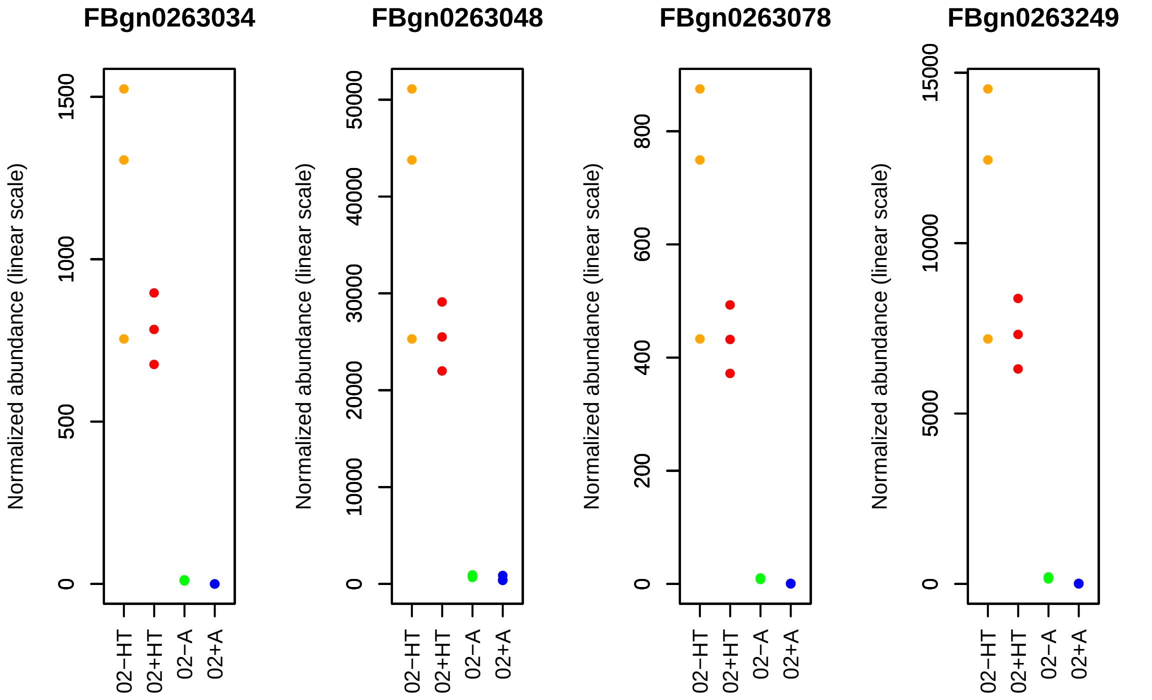
FBgn0263034
FBgn0263048
FBgn0263078
FBgn0263249
Normalized abundance (linear scale)
0
500
1000
1500
10000
20000
30000
40000
50000
200
400
600
800
5000
15000
02−HT
02+HT
02−A
02+A

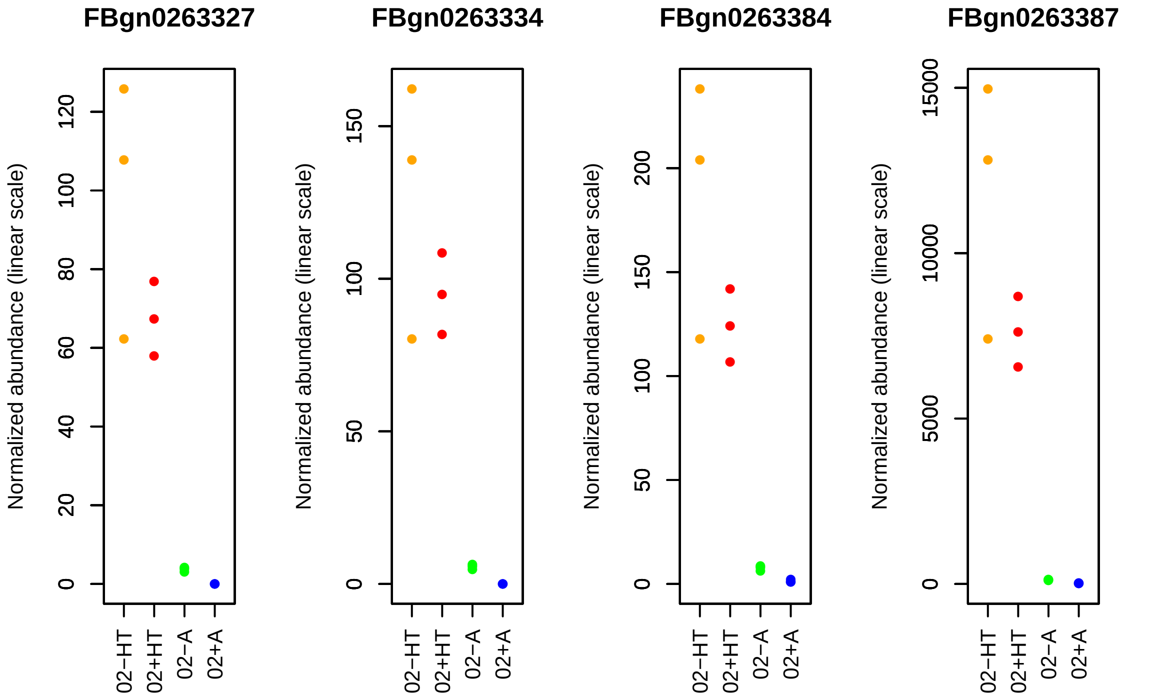
FBgn0263327
FBgn0263334
FBgn0263384
FBgn0263387
Normalized abundance (linear scale)
0
20
40
60
80
100
120
150
50
200
5000
10000
15000
02−HT
02+HT
02−A
02+A

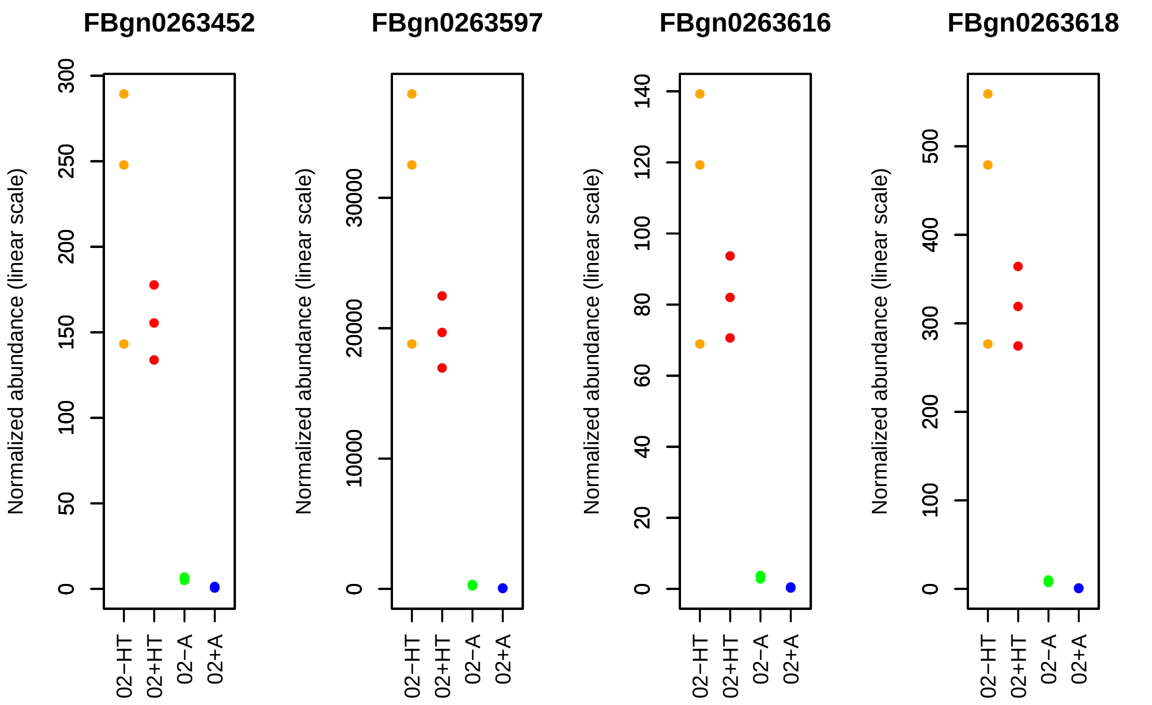

FBgn0263452
FBgn0263597
FBgn0263616
FBgn0263618
Normalized abundance (linear scale)
0
50
100
150
200
250
300
10000
20000
30000
20
40
60
80
100
120
140
200
400
500
02−HT
02+HT
02−A
02+A

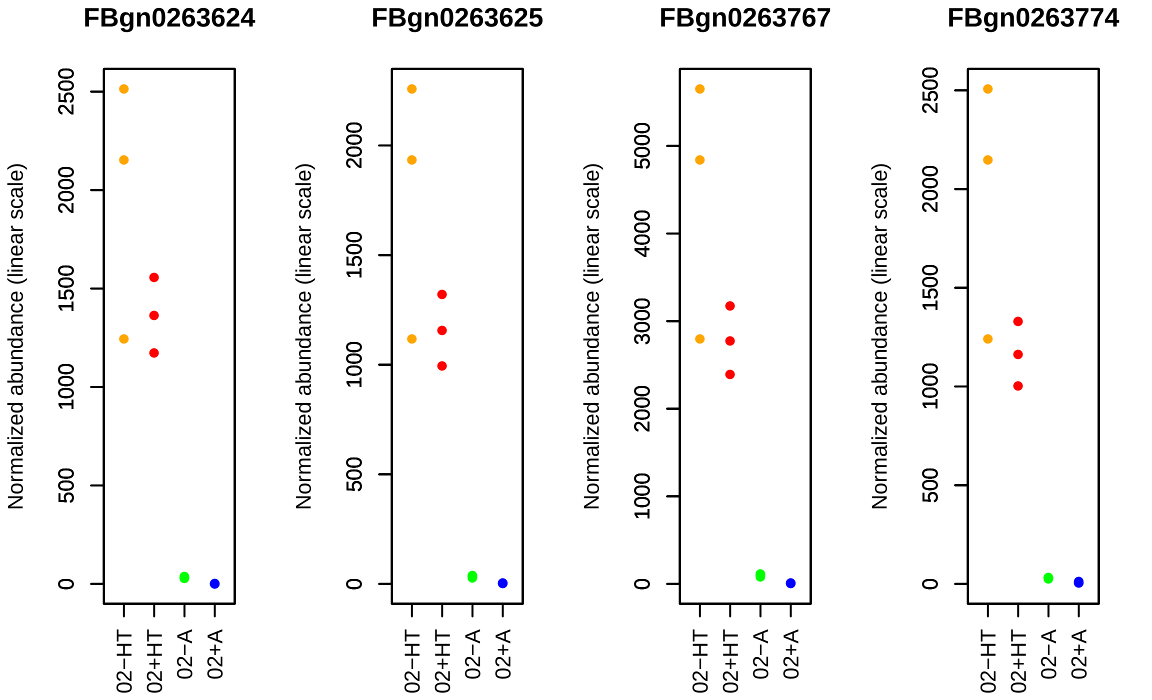
FBgn0263624
FBgn0263625
FBgn0263767
FBgn0263774
Normalized abundance (linear scale)
0
500
1000
1500
2000
2500
3000
4000
5000
02−HT
02+HT
02−A
02+A

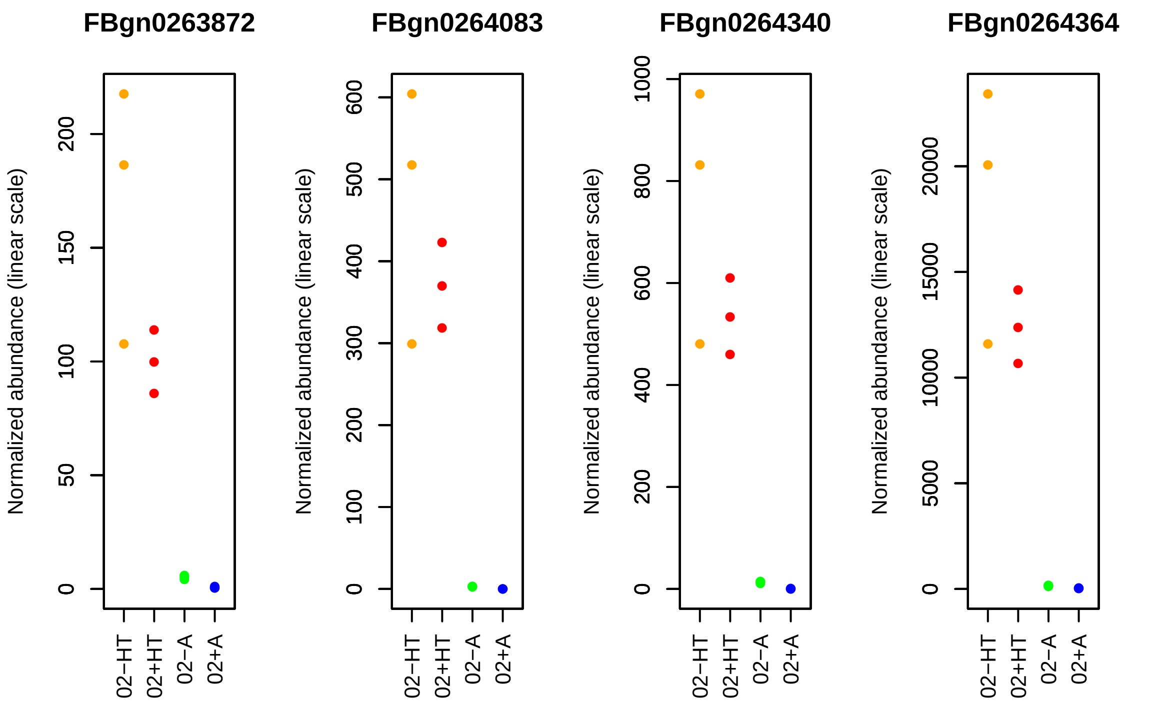

FBgn0263872
FBgn0264083
FBgn0264340
FBgn0264364
Normalized abundance (linear scale)
0
50
100
150
200
100
200
300
400
500
600
400
600
800
1000
5000
10000
15000
20000
02−HT
02+HT
02−A
02+A

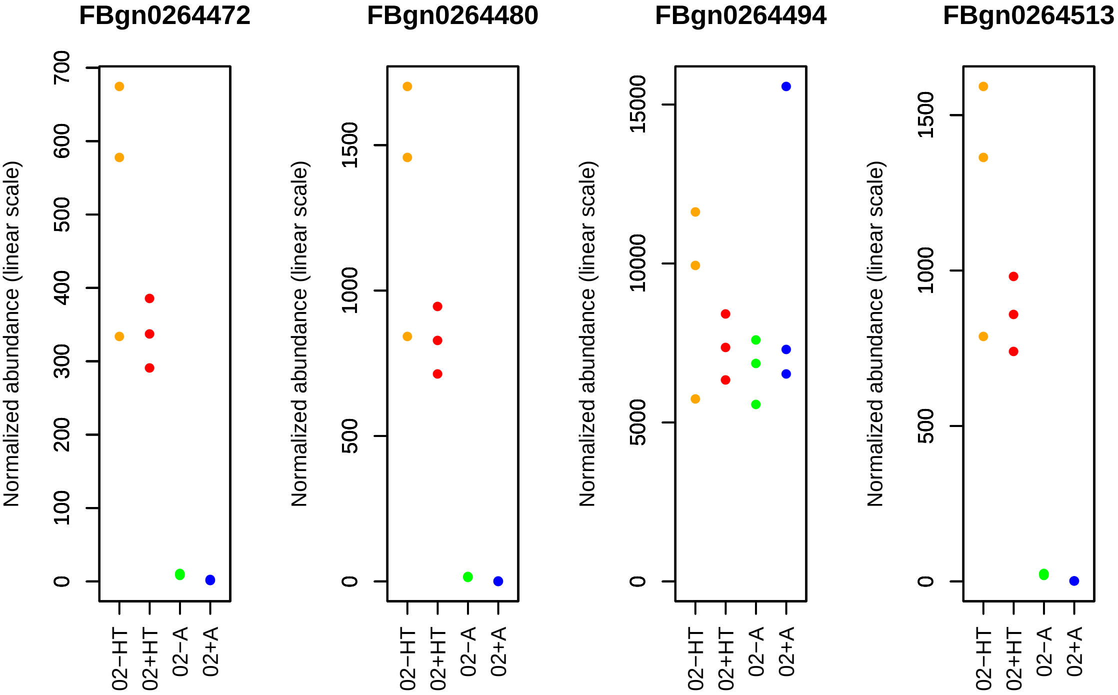
FBgn0264472
FBgn0264480
FBgn0264494
FBgn0264513
Normalized abundance (linear scale)
0
100
200
300
400
500
600
700
500
1000
1500
5000
10000
15000
02−HT
02+HT
02−A
02+A

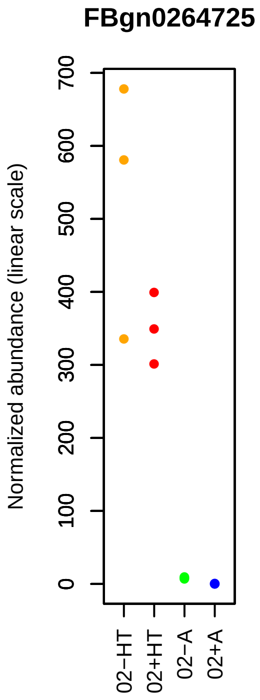
FBgn0264725
Normalized abundance (linear scale)
0
100
200
300
400
500
600
700
02−HT
02+HT
02−A
02+A

Fig S7B

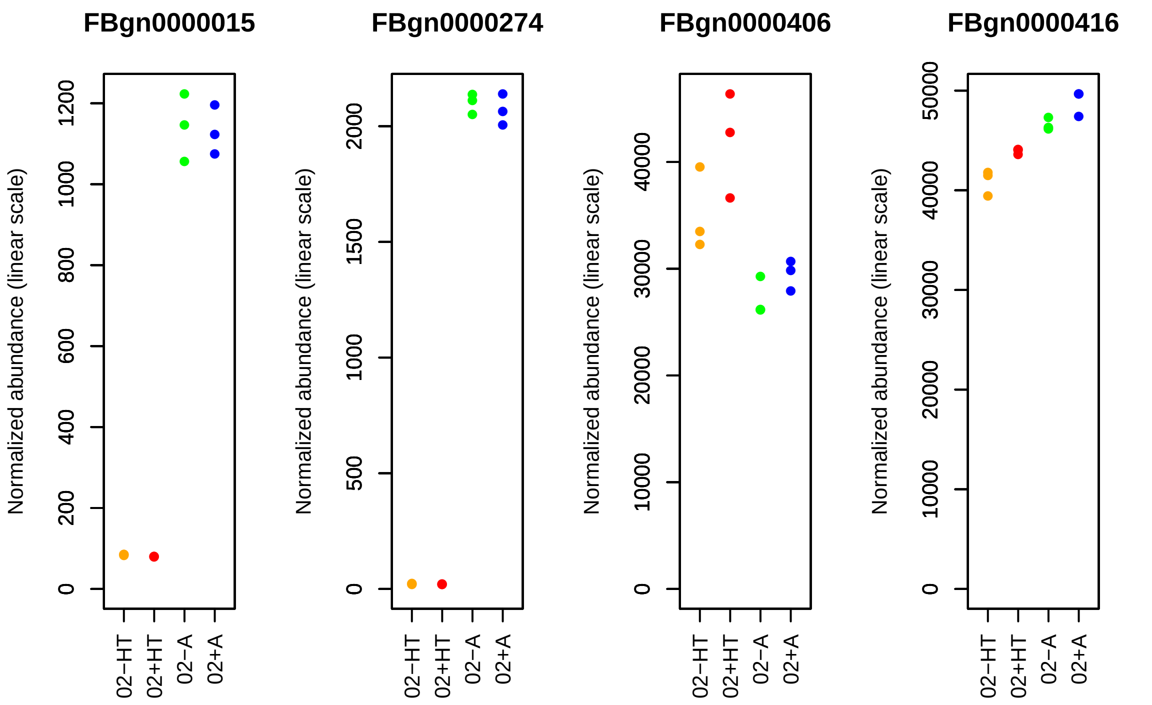
FBgn0000015
FBgn0000274
FBgn0000406
FBgn0000416
Normalized abundance (linear scale)
0
200
400
600
800
1000
1200
500
1000
1500
2000
10000
20000
30000
40000
50000
02−HT
02+HT
02−A
02+A

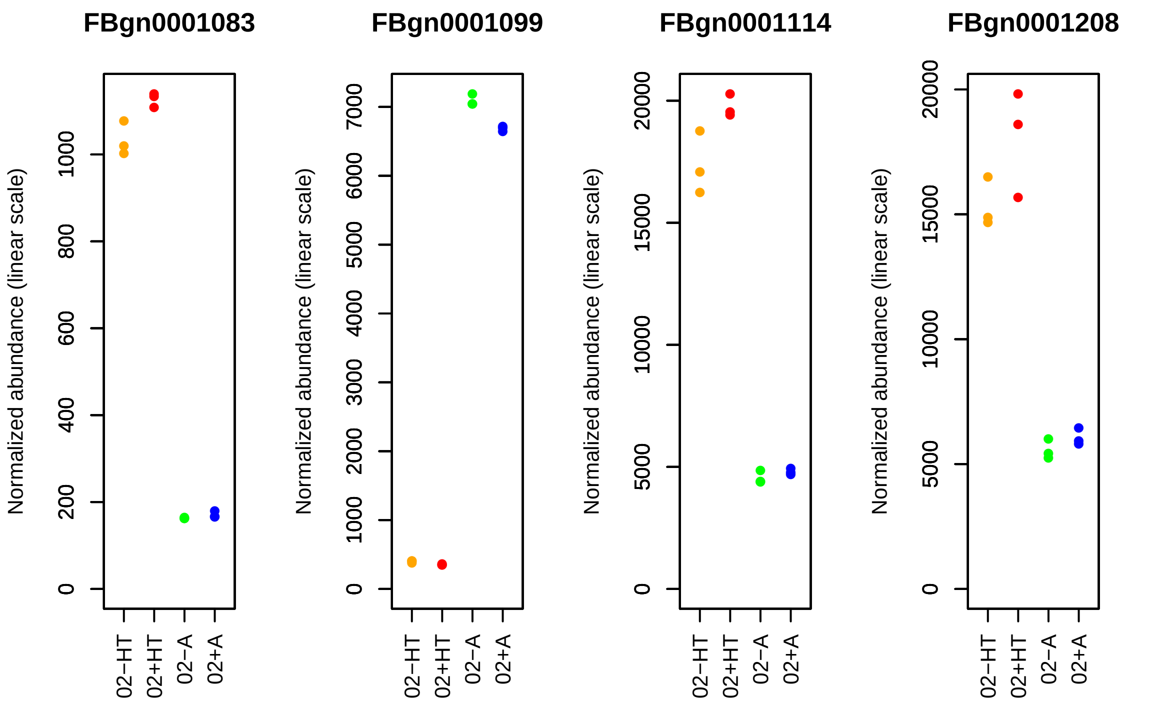

FBgn0001083
FBgn0001099
FBgn0001114
FBgn0001208
Normalized abundance (linear scale)
0
200
400
600
800
1000
1000
2000
3000
4000
5000
6000
7000
5000
10000
15000
20000
02−HT
02+HT
02−A
02+A

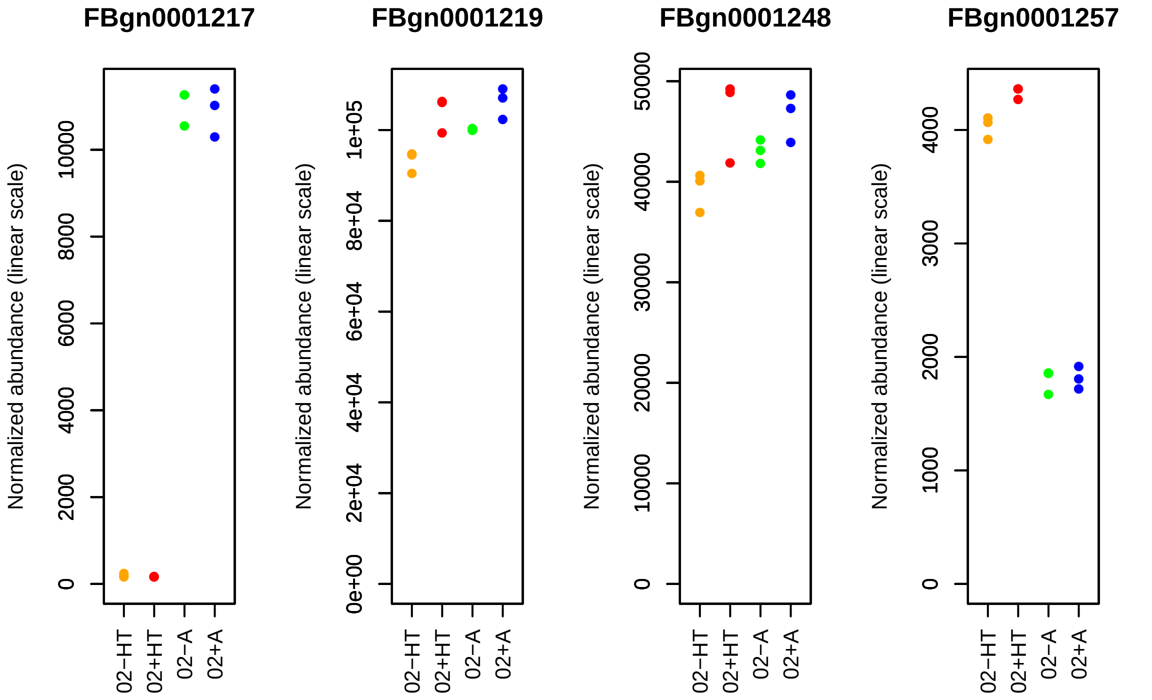

FBgn0001217
FBgn0001219
FBgn0001248
FBgn0001257
Normalized abundance (linear scale)
0
2000
4000
6000
8000
10000
0e+00
2e+04
4e+04
6e+04
8e+04
1e+05
10000
20000
30000
40000
50000
1000
2000
3000
4000
02−HT
02+HT
02−A
02+A

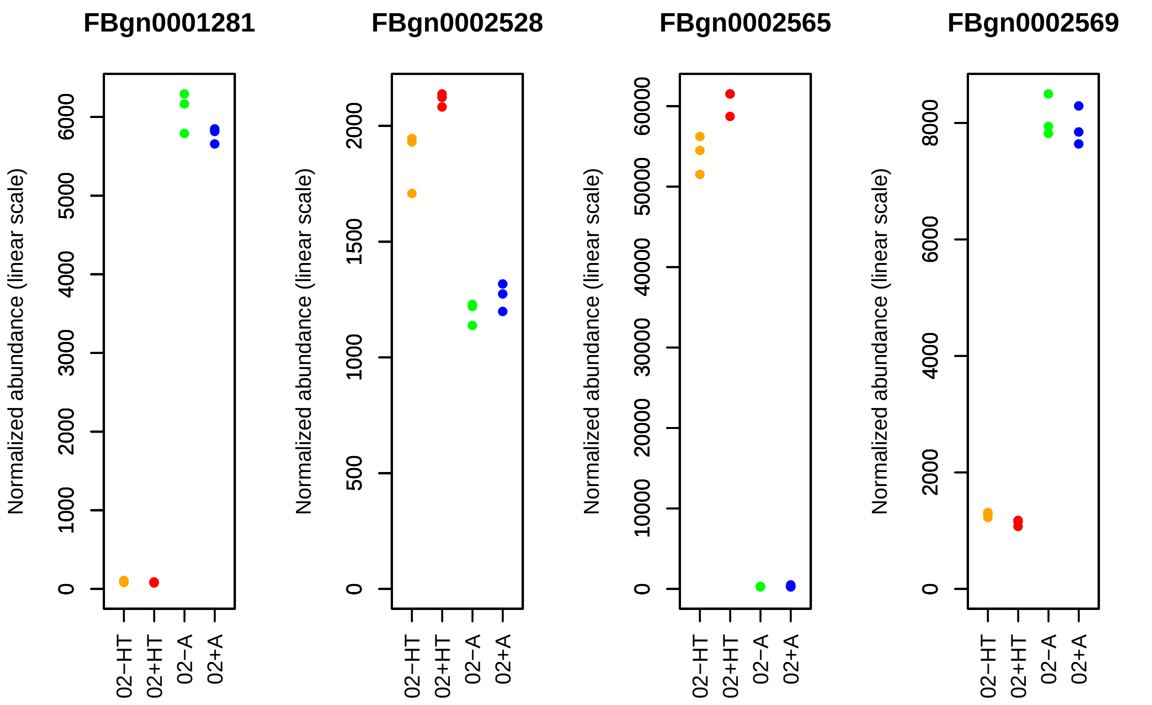
FBgn0001281
FBgn0002528
FBgn0002565
FBgn0002569
Normalized abundance (linear scale)
0
1000
2000
3000
4000
5000
6000
500
1500
10000
20000
30000
40000
50000
60000
8000
02−HT
02+HT
02−A
02+A

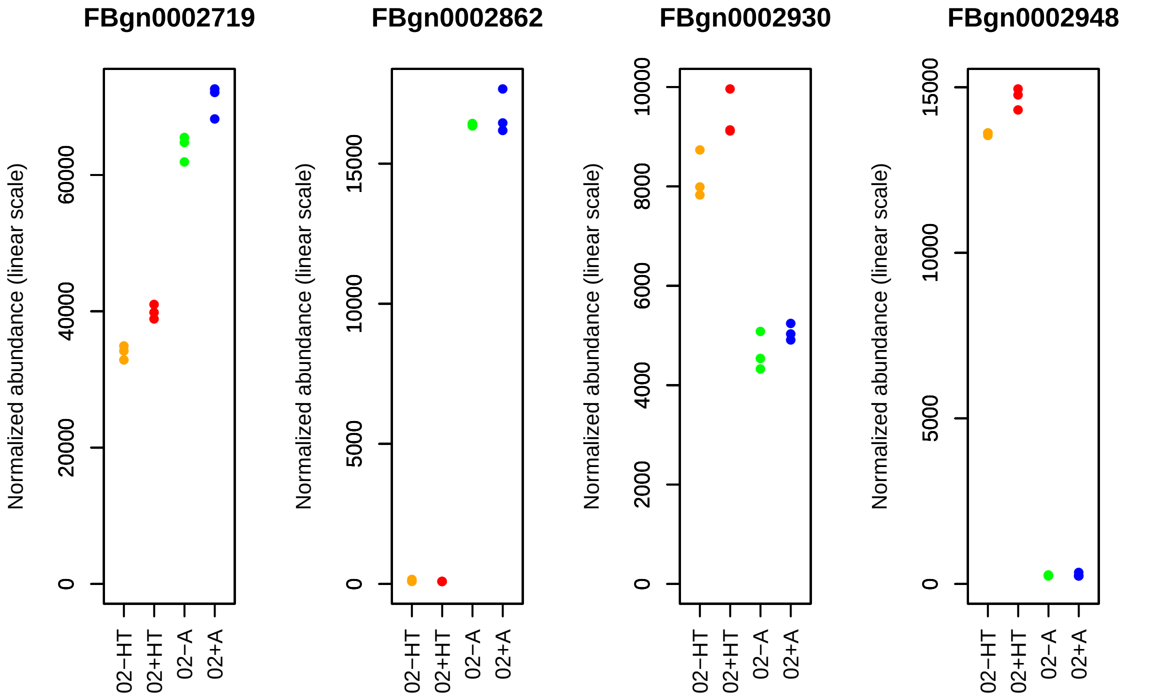
FBgn0002719
FBgn0002862
FBgn0002930
FBgn0002948
Normalized abundance (linear scale)
0
20000
40000
60000
5000
10000
15000
2000
4000
6000
8000
10000
02−HT
02+HT
02−A
02+A

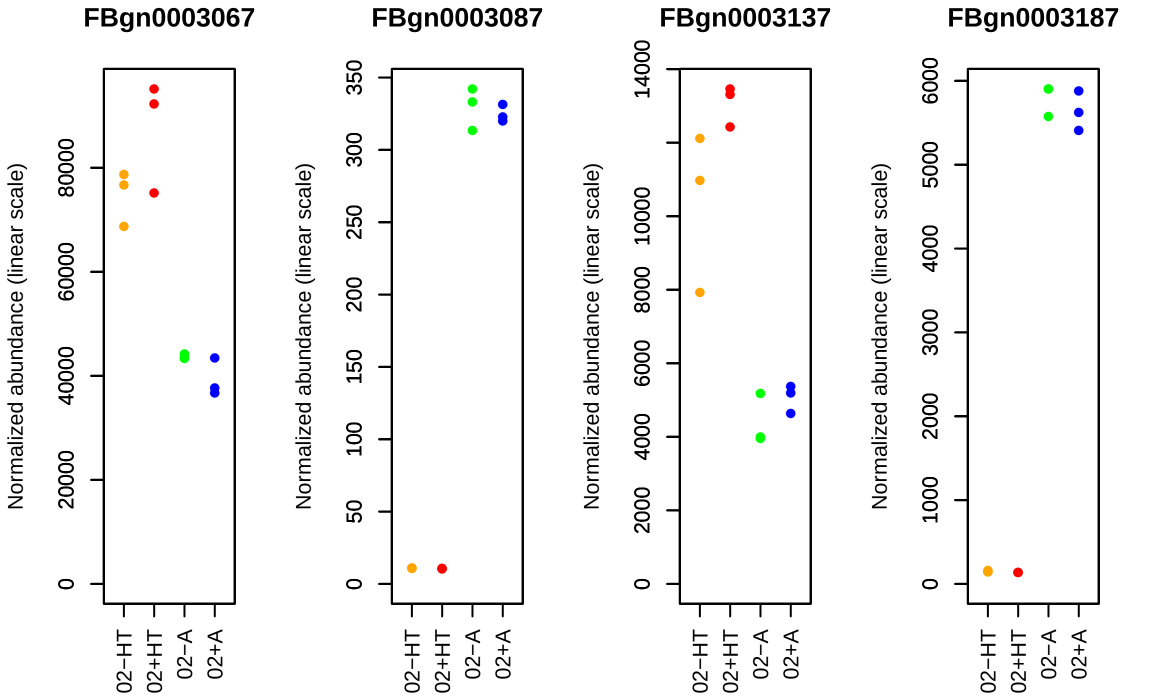
FBgn0003067
FBgn0003087
FBgn0003137
FBgn0003187
Normalized abundance (linear scale)
0
20000
40000
60000
80000
50
100
150
200
250
300
350
2000
4000
6000
8000
10000
14000
1000
2000
3000
4000
5000
6000
02−HT
02+HT
02−A
02+A

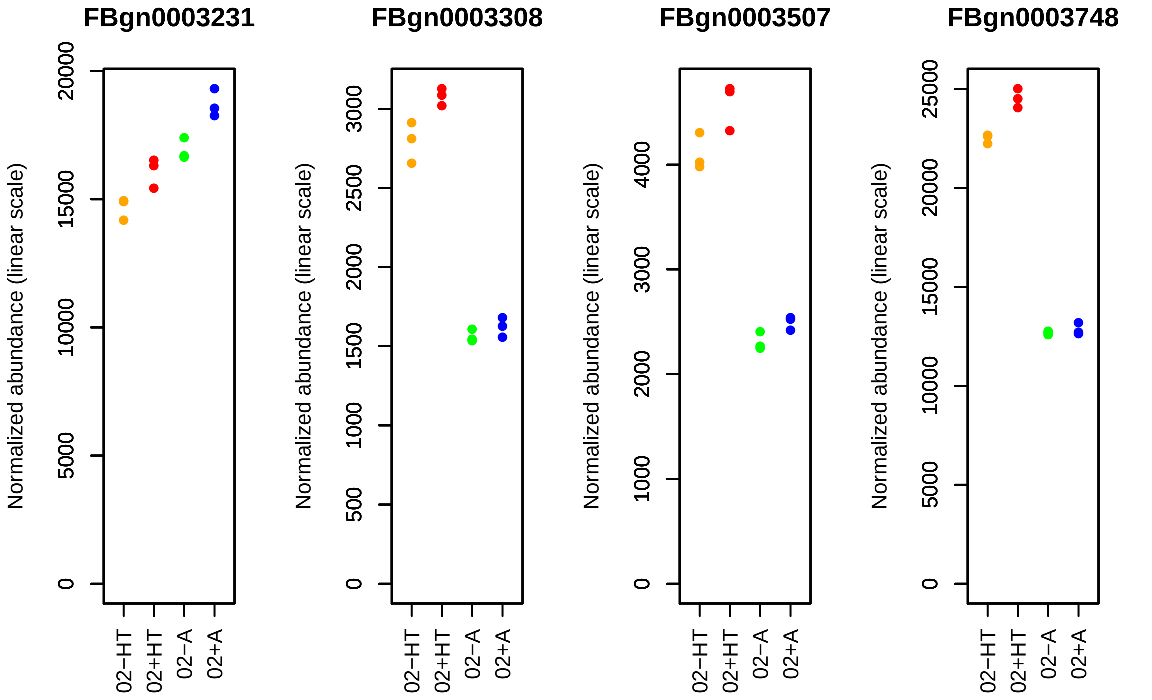

FBgn0003231
FBgn0003308
FBgn0003507
FBgn0003748
Normalized abundance (linear scale)
0
5000
10000
15000
20000
500
1000
1500
2000
2500
3000
4000
25000
02−HT
02+HT
02−A
02+A

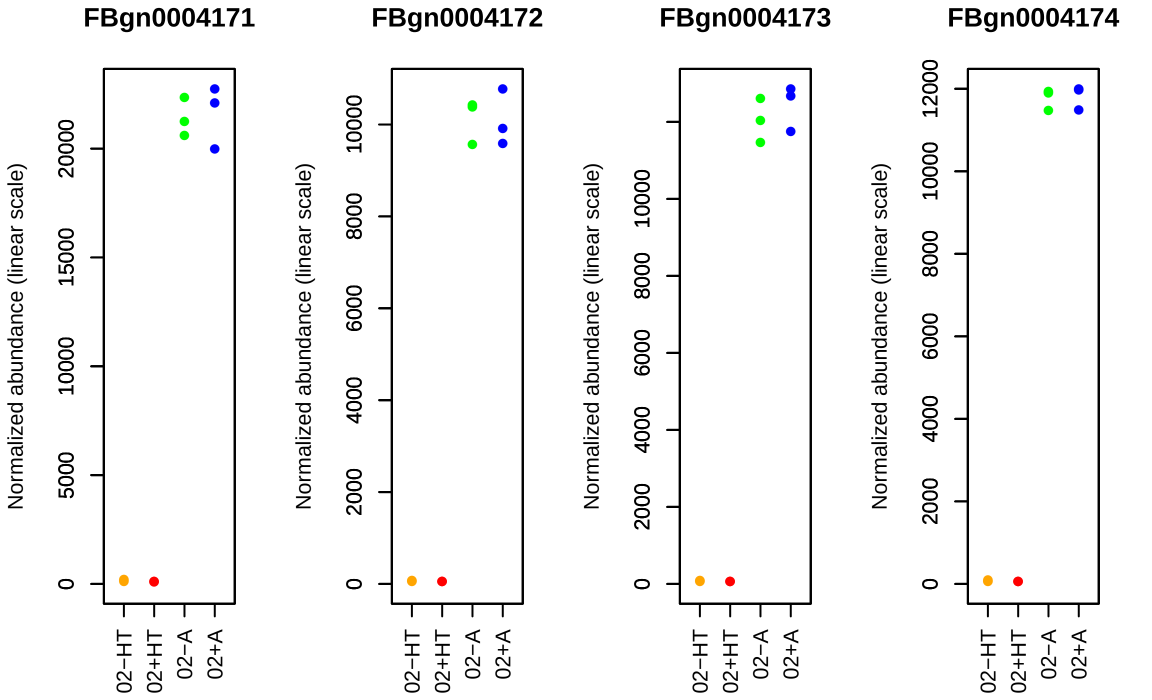
FBgn0004171
FBgn0004172
FBgn0004173
FBgn0004174
Normalized abundance (linear scale)
0
5000
10000
15000
20000
2000
4000
6000
8000
10000
12000
02−HT
02+HT
02−A
02+A

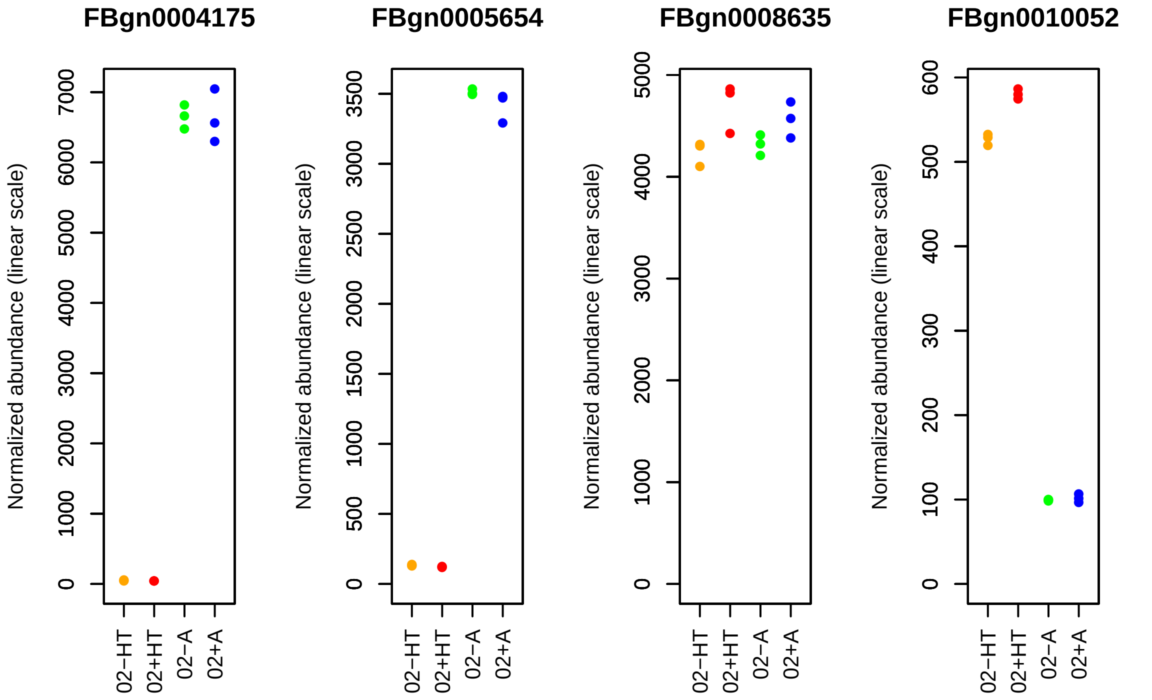

FBgn0004175
FBgn0005654
FBgn0008635
FBgn0010052
Normalized abundance (linear scale)
0
1000
2000
3000
4000
5000
6000
7000
500
1500
2500
3500
100
200
300
400
600
02−HT
02+HT
02−A
02+A

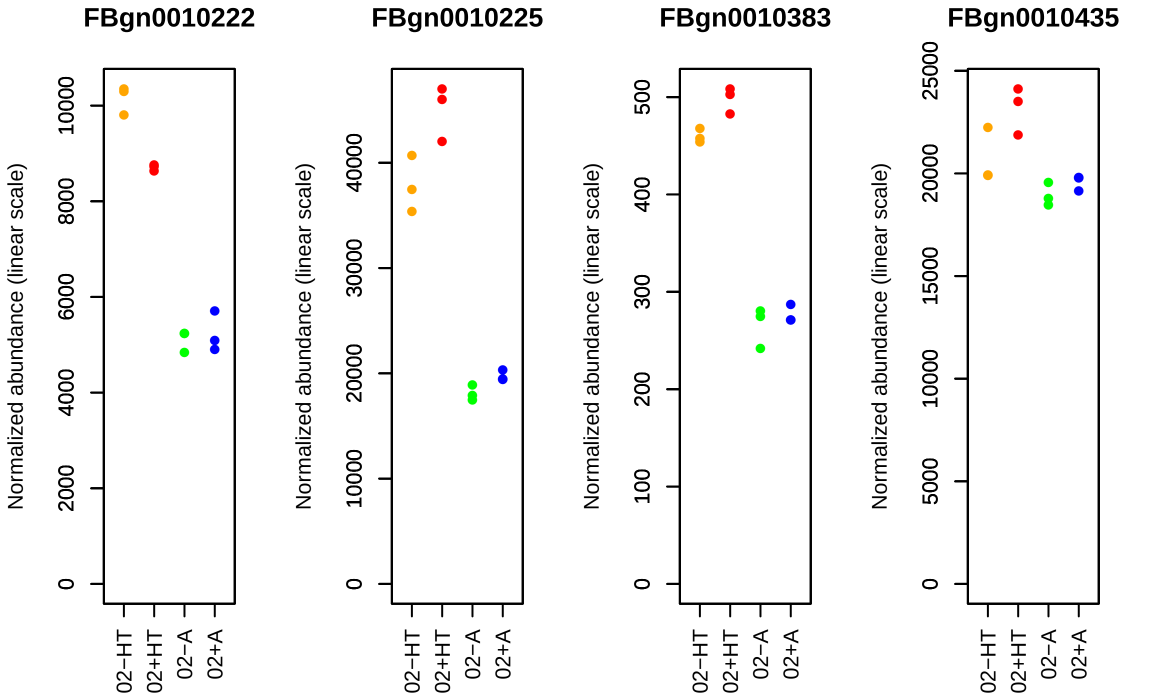

FBgn0010222
FBgn0010225
FBgn0010383
FBgn0010435
Normalized abundance (linear scale)
0
2000
4000
6000
8000
10000
10000
20000
30000
40000
100
200
300
400
500
5000
15000
20000
25000
02−HT
02+HT
02−A
02+A

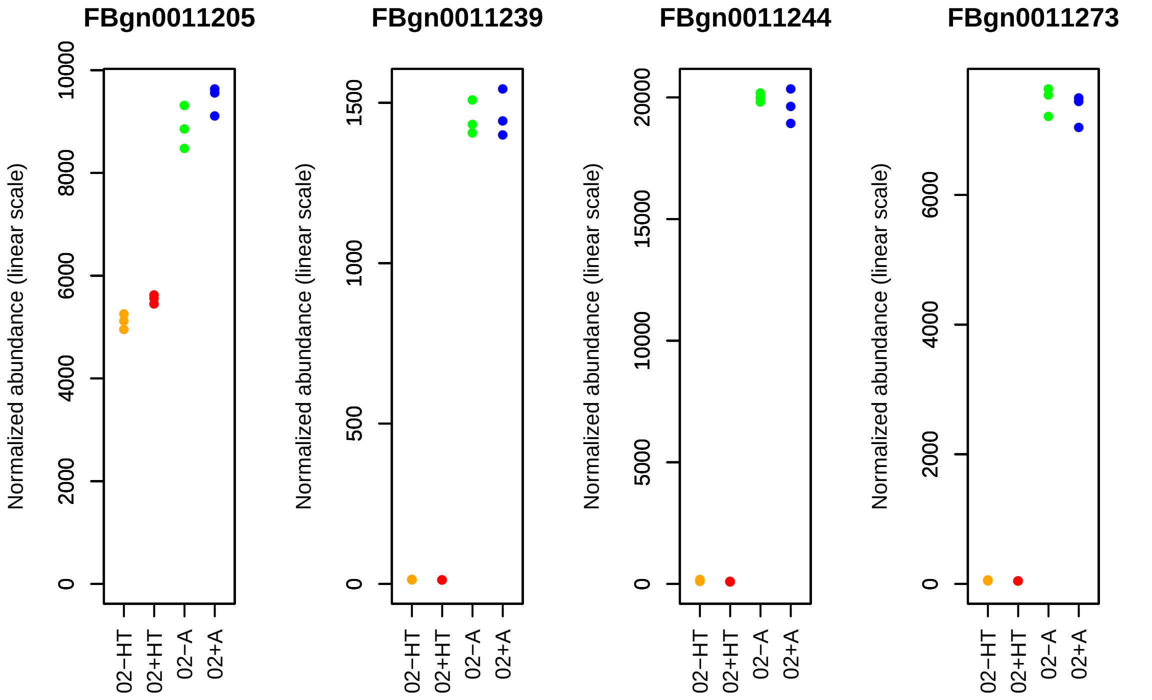
FBgn0011205
FBgn0011239
FBgn0011244
FBgn0011273
Normalized abundance (linear scale)
0
2000
4000
6000
8000
10000
500
1000
1500
5000
15000
20000
02−HT
02+HT
02−A
02+A

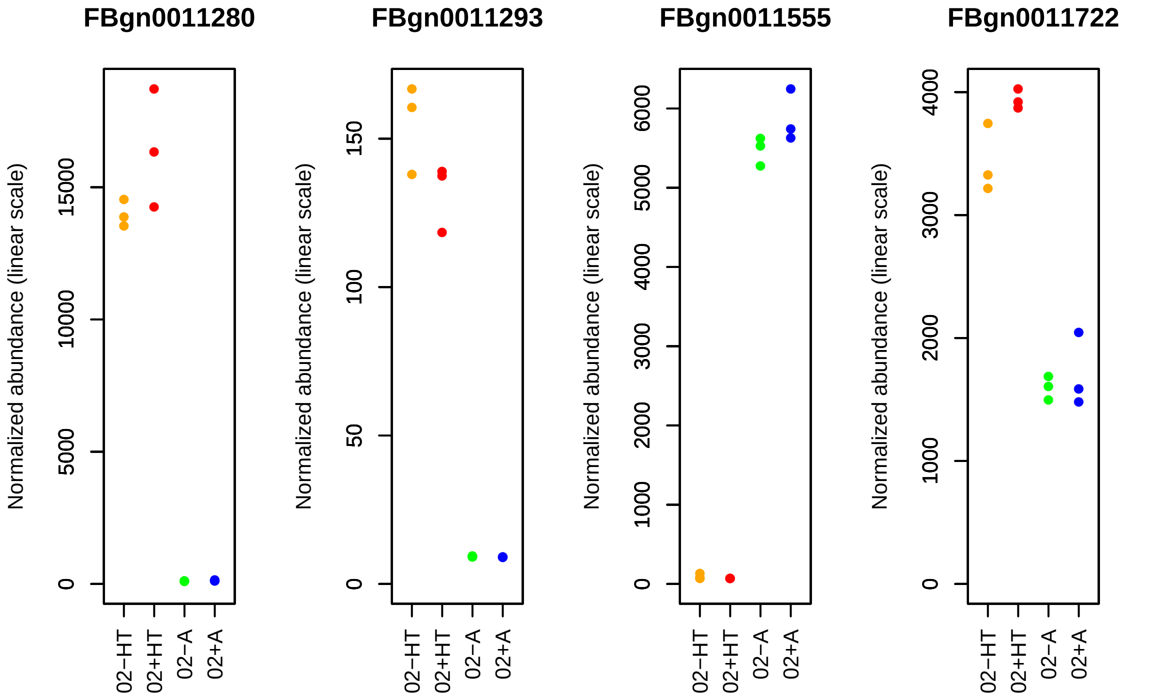

FBgn0011280
FBgn0011293
FBgn0011555
FBgn0011722
Normalized abundance (linear scale)
0
5000
10000
15000
50
100
150
1000
2000
3000
4000
5000
6000
02−HT
02+HT
02−A
02+A

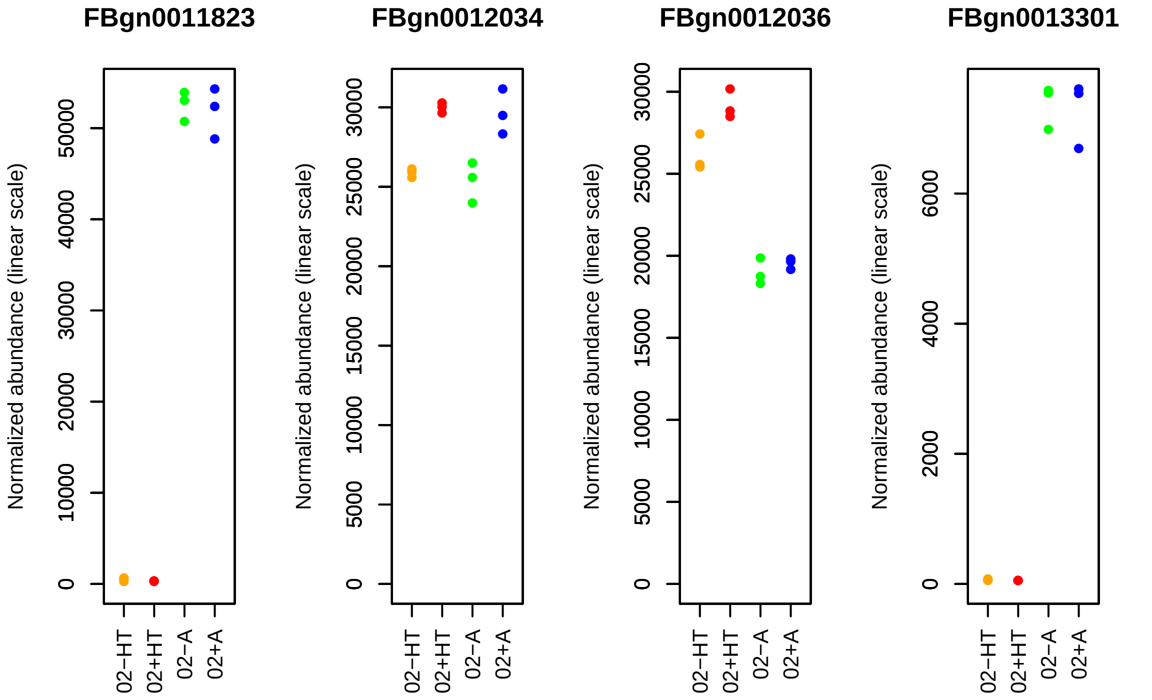
FBgn0011823
FBgn0012034
FBgn0012036
FBgn0013301
Normalized abundance (linear scale)
0
10000
20000
30000
40000
50000
5000
15000
25000
2000
4000
6000
02−HT
02+HT
02−A
02+A

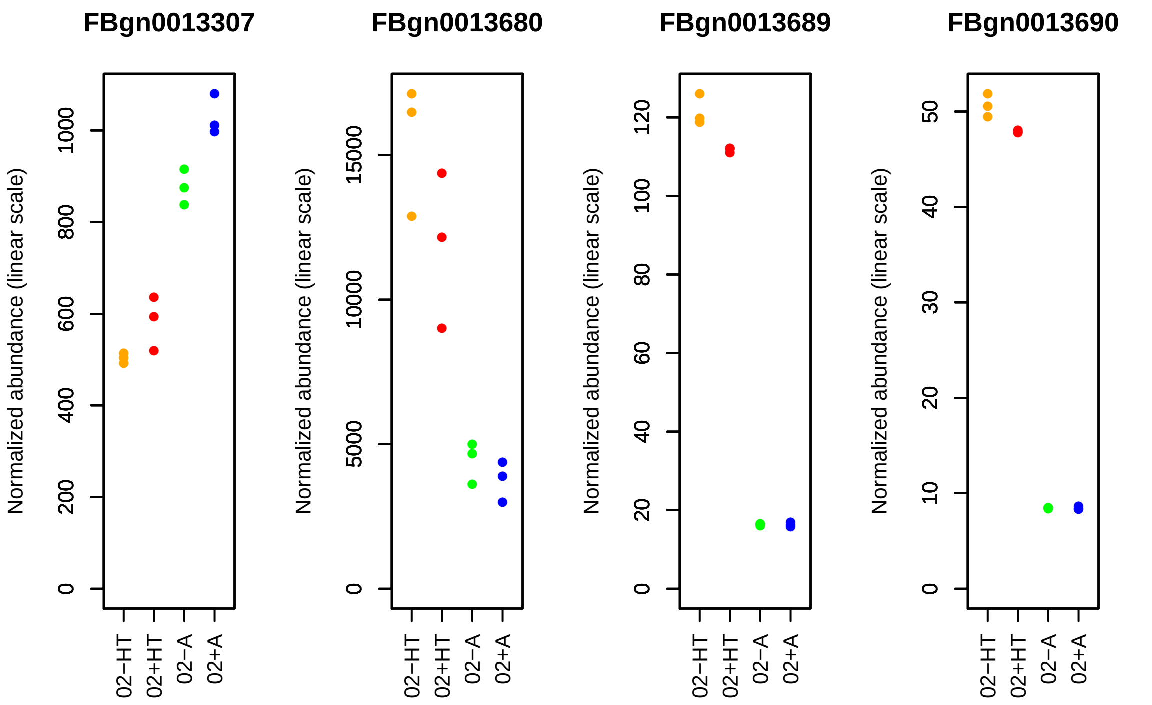

FBgn0013307
FBgn0013680
FBgn0013689
FBgn0013690
Normalized abundance (linear scale)
0
200
400
600
800
1000
5000
10000
15000
20
40
60
80
100
120
10
30
50
02−HT
02+HT
02−A
02+A

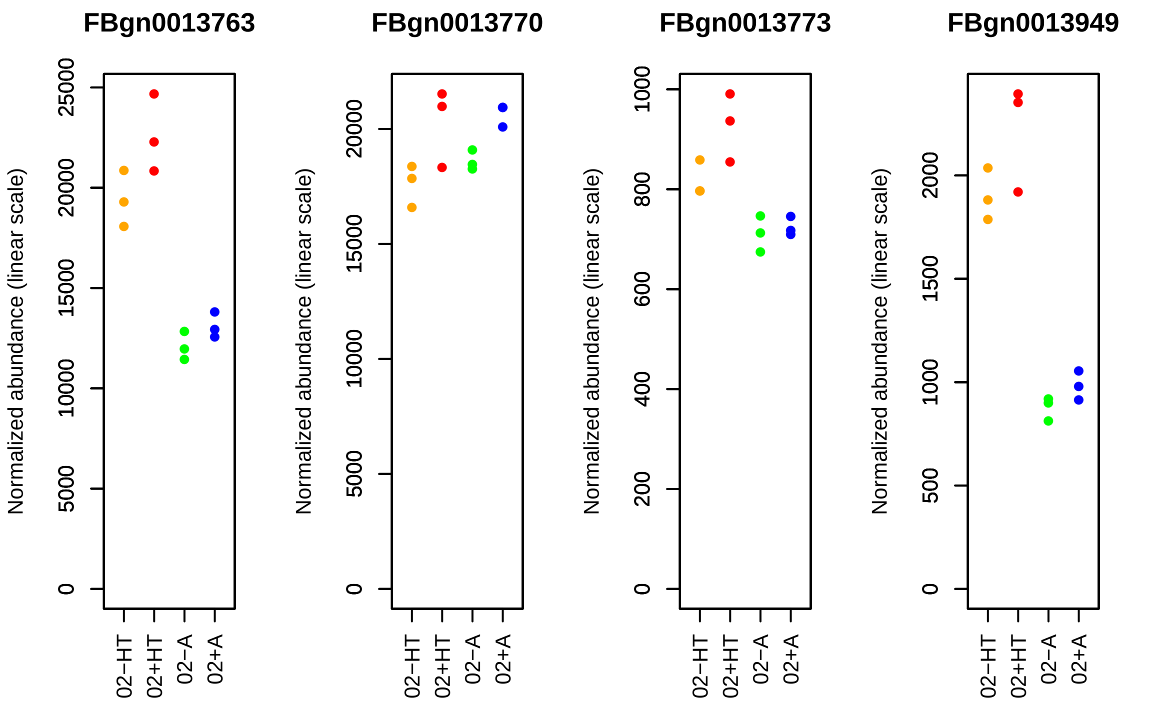

FBgn0013763
FBgn0013770
FBgn0013773
FBgn0013949
Normalized abundance (linear scale)
0
5000
10000
15000
20000
25000
200
400
600
800
1000
500
1000
1500
2000
02−HT
02+HT
02−A
02+A

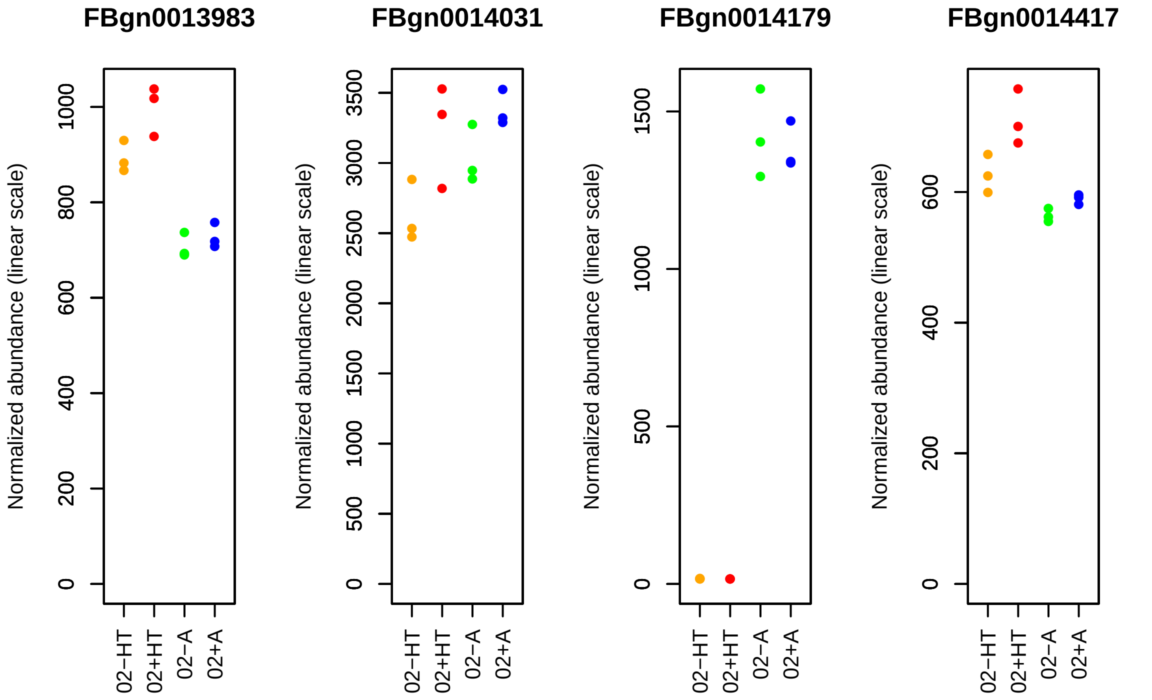
FBgn0013983
FBgn0014031
FBgn0014179
FBgn0014417
Normalized abundance (linear scale)
0
200
400
600
800
1000
500
1000
1500
2000
2500
3000
3500
02−HT
02+HT
02−A
02+A

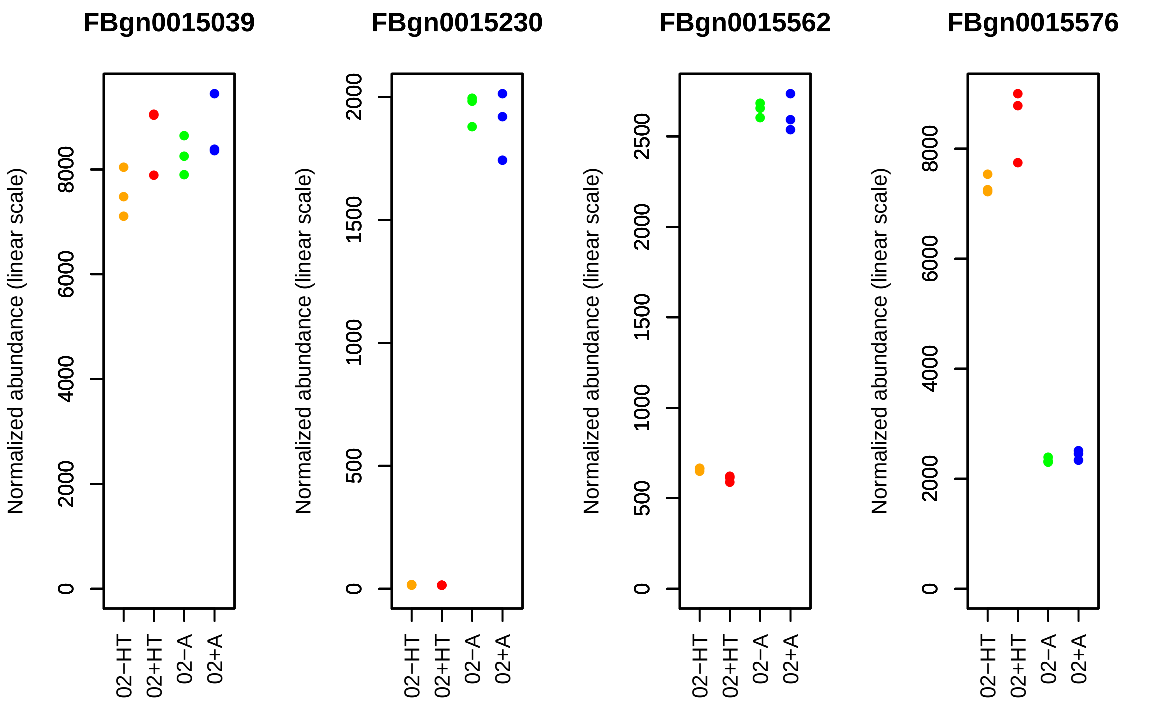
FBgn0015039
FBgn0015230
FBgn0015562
FBgn0015576
Normalized abundance (linear scale)
0
2000
4000
6000
8000
0
500
1000
1500
2000
2500
02−HT
02+HT
02−A
02+A

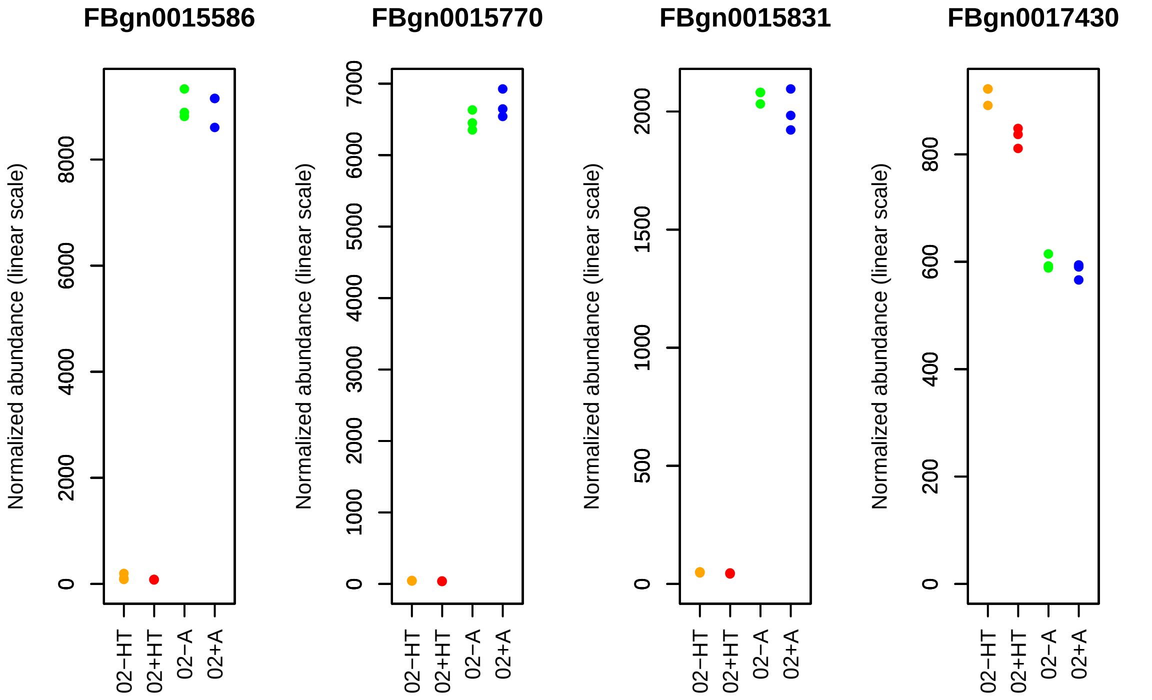

FBgn0015586
FBgn0015770
FBgn0015831
FBgn0017430
Normalized abundance (linear scale)
0
2000
4000
6000
8000
1000
3000
5000
7000
500
1500
200
400
600
800
02−HT
02+HT
02−A
02+A

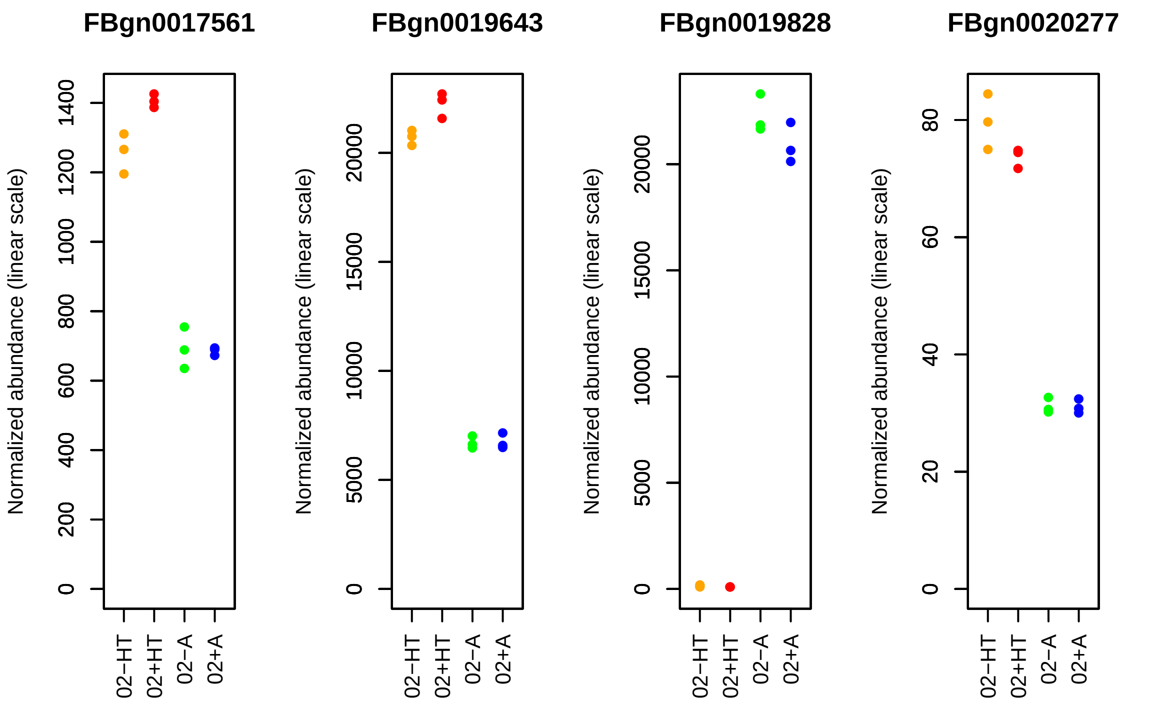

FBgn0017561
FBgn0019643
FBgn0019828
FBgn0020277
Normalized abundance (linear scale)
0
200
400
600
800
1000
1200
1400
5000
10000
15000
20000
20
40
60
80
02−HT
02+HT
02−A
02+A

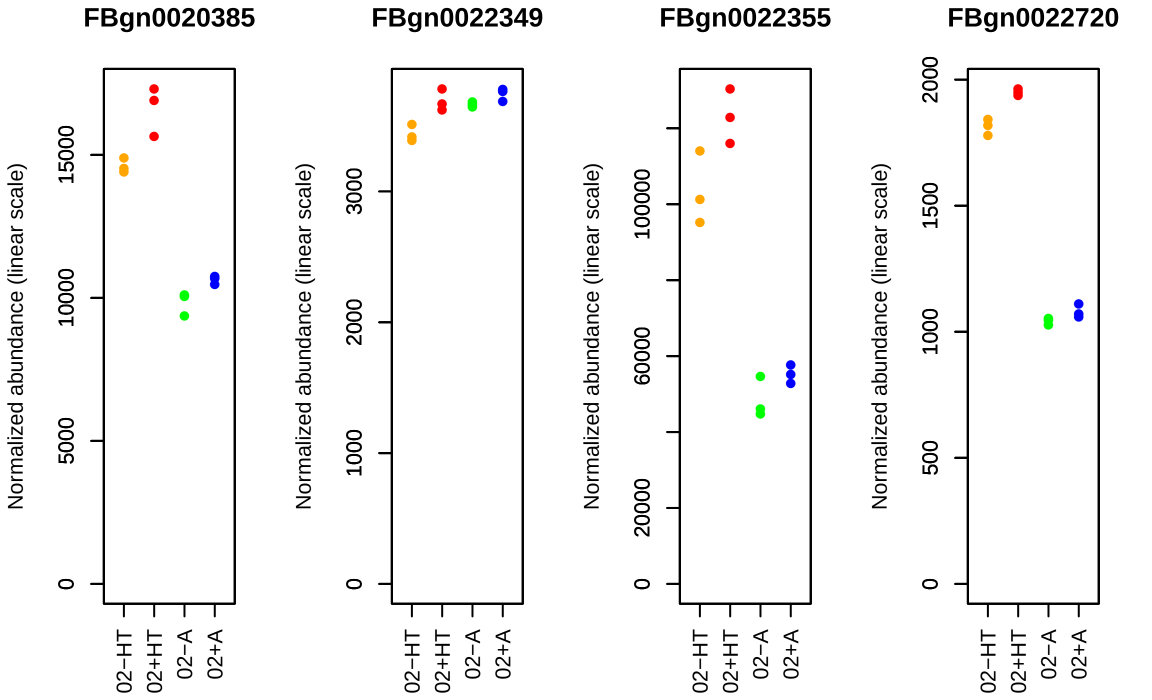
FBgn0020385
FBgn0022349
FBgn0022355
FBgn0022720
Normalized abundance (linear scale)
0
5000
10000
15000
1000
2000
3000
20000
60000
100000
500
1500
2000
02−HT
02+HT
02−A
02+A

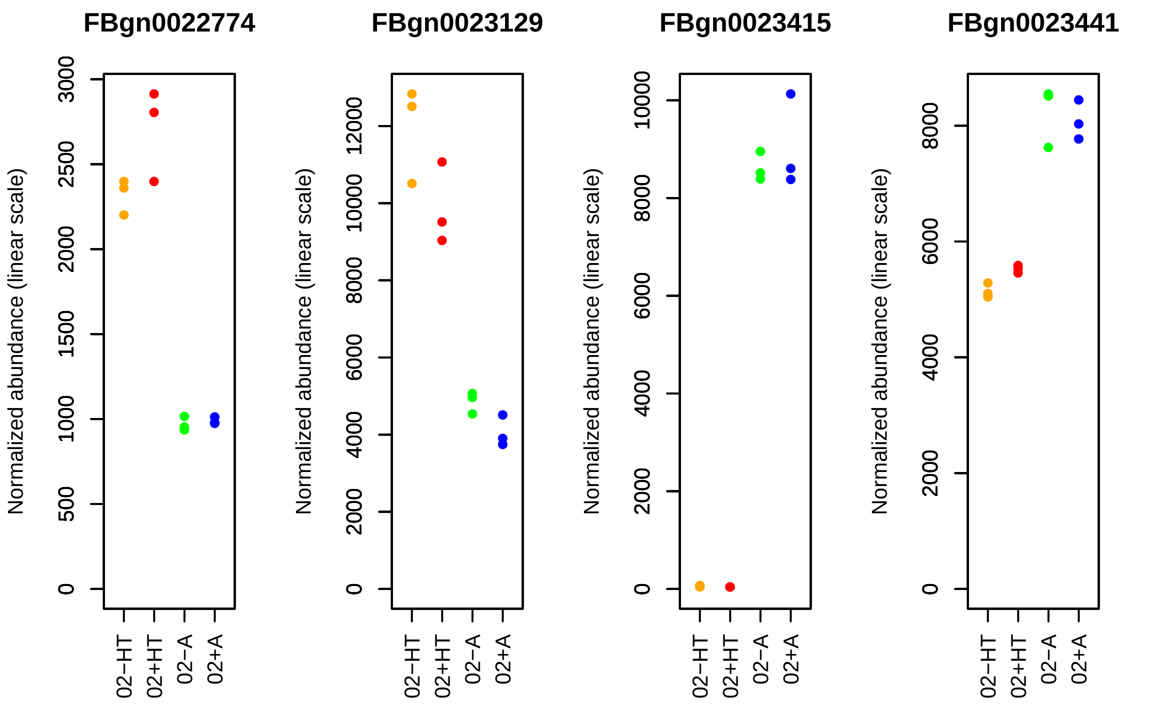
FBgn0022774
FBgn0023129
FBgn0023415
FBgn0023441
Normalized abundance (linear scale)
0
500
1000
1500
2000
2500
3000
2000
4000
6000
8000
10000
12000
02−HT
02+HT
02−A
02+A

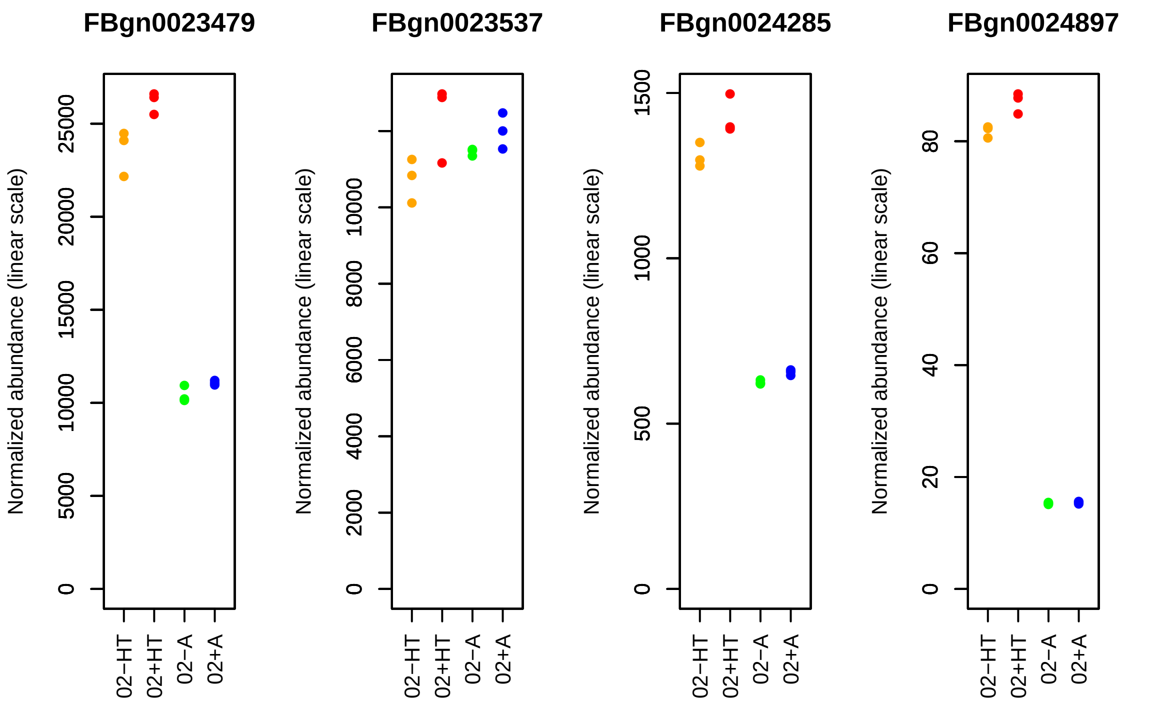

FBgn0023479
FBgn0023537
FBgn0024285
FBgn0024897
Normalized abundance (linear scale)
0
5000
10000
15000
20000
25000
2000
4000
6000
8000
500
1000
1500
20
40
60
80
02−HT
02+HT
02−A
02+A

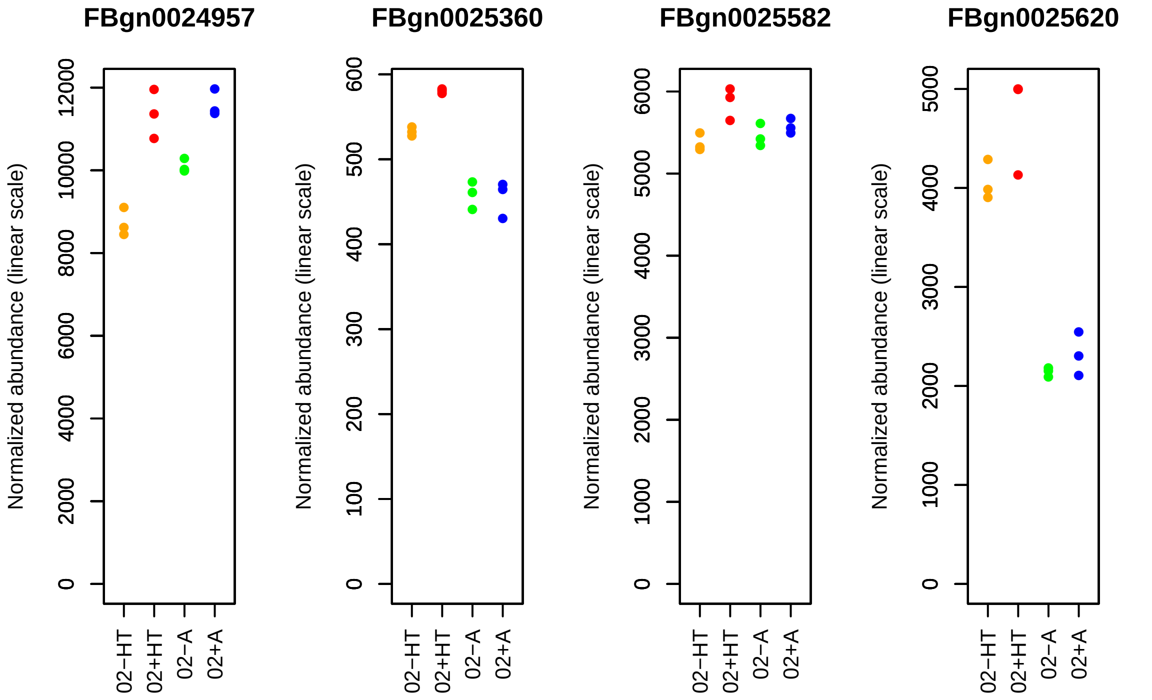
FBgn0024957
FBgn0025360
FBgn0025582
FBgn0025620
Normalized abundance (linear scale)
0
2000
4000
6000
8000
10000
12000
0
100
200
300
400
500
600
0
1000
2000
3000
4000
5000
6000
0
1000
2000
3000
4000
5000
02−HT
02+HT
02−A
02+A

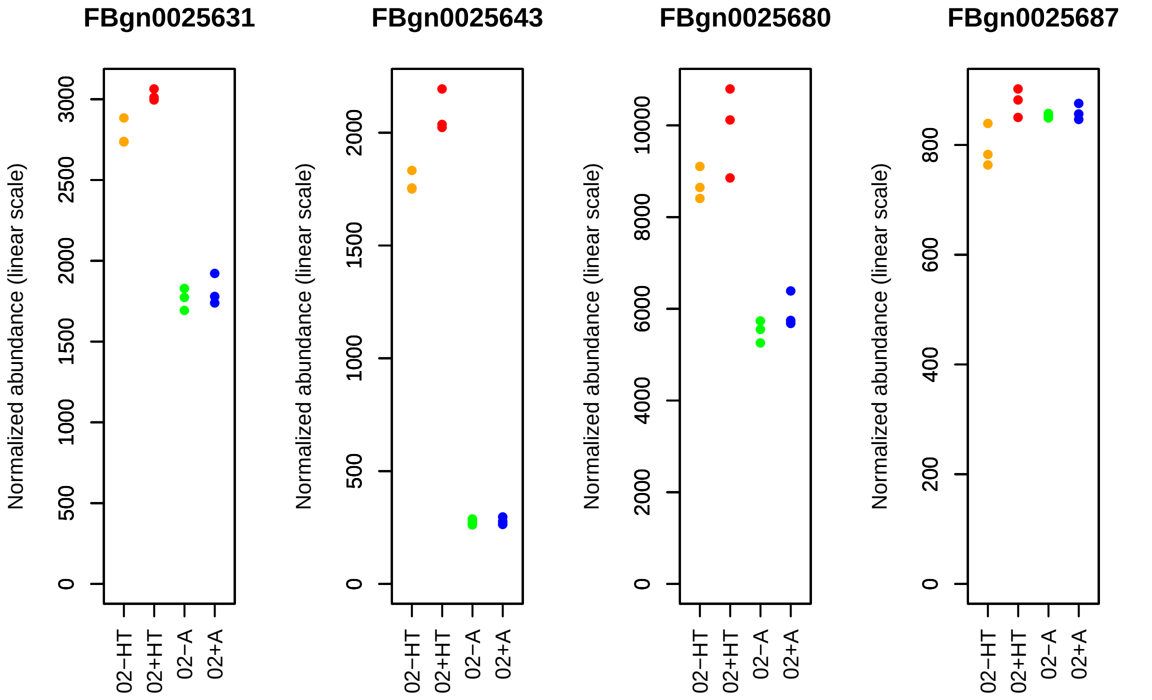

FBgn0025631
FBgn0025643
FBgn0025680
FBgn0025687
Normalized abundance (linear scale)
0
500
1000
1500
2000
2500
3000
0
500
1000
1500
2000
0
2000
4000
6000
8000
10000
0
200
400
600
800
02−HT
02+HT
02−A
02+A

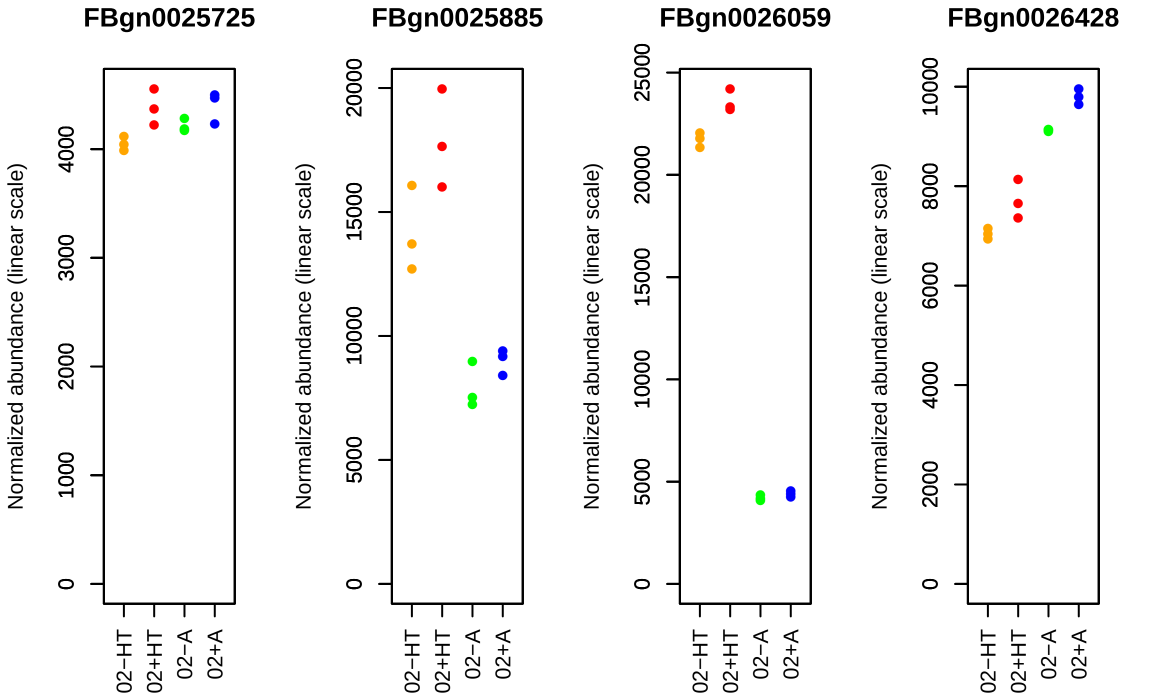

FBgn0025725
FBgn0025885
FBgn0026059
FBgn0026428
Normalized abundance (linear scale)
0
1000
2000
3000
4000
5000
10000
15000
20000
25000
6000
8000
02−HT
02+HT
02−A
02+A

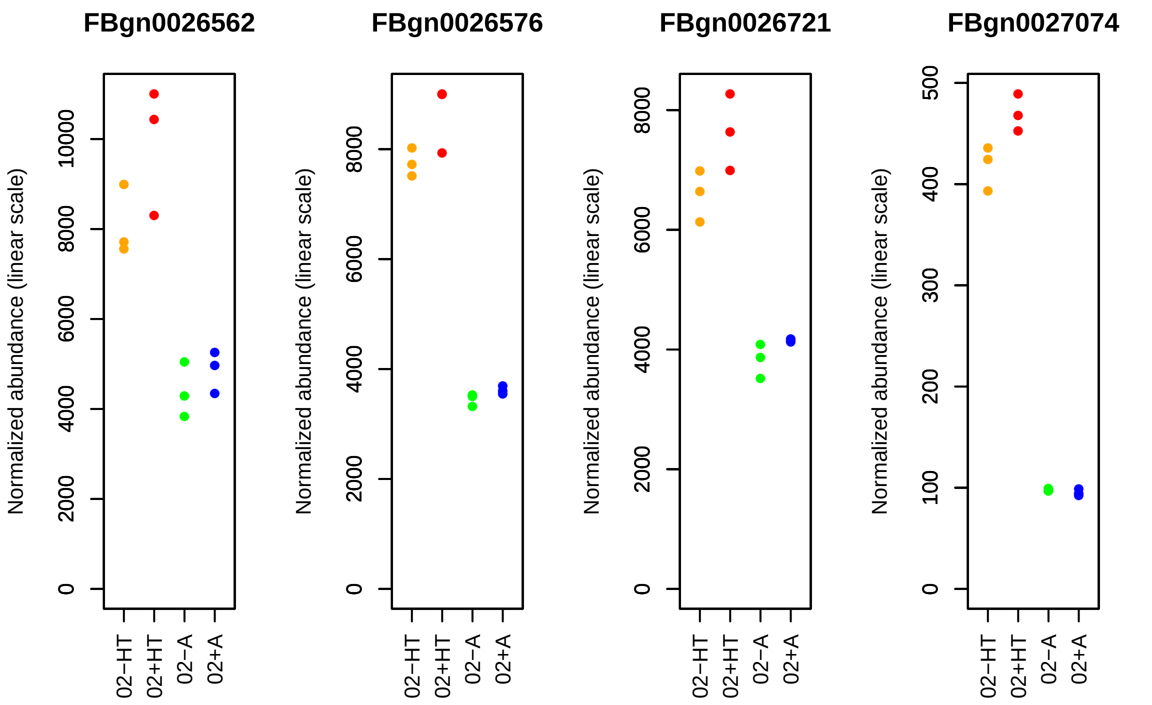
FBgn0026562
FBgn0026576
FBgn0026721
FBgn0027074
Normalized abundance (linear scale)
0
2000
4000
6000
8000
10000
100
200
300
400
500
02−HT
02+HT
02−A
02+A

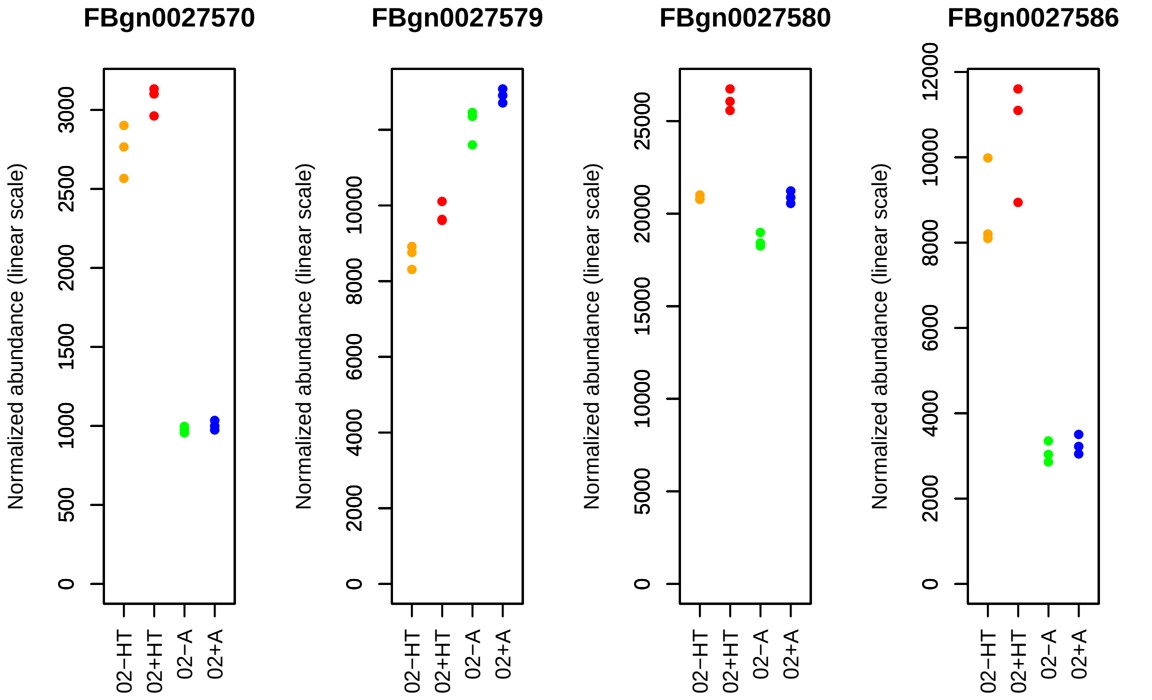

FBgn0027570
FBgn0027579
FBgn0027580
FBgn0027586
Normalized abundance (linear scale)
0
500
1000
1500
2000
2500
3000
0
2000
4000
6000
8000
10000
0
5000
10000
15000
20000
25000
0
2000
4000
6000
8000
10000
12000
02−HT
02+HT
02−A
02+A

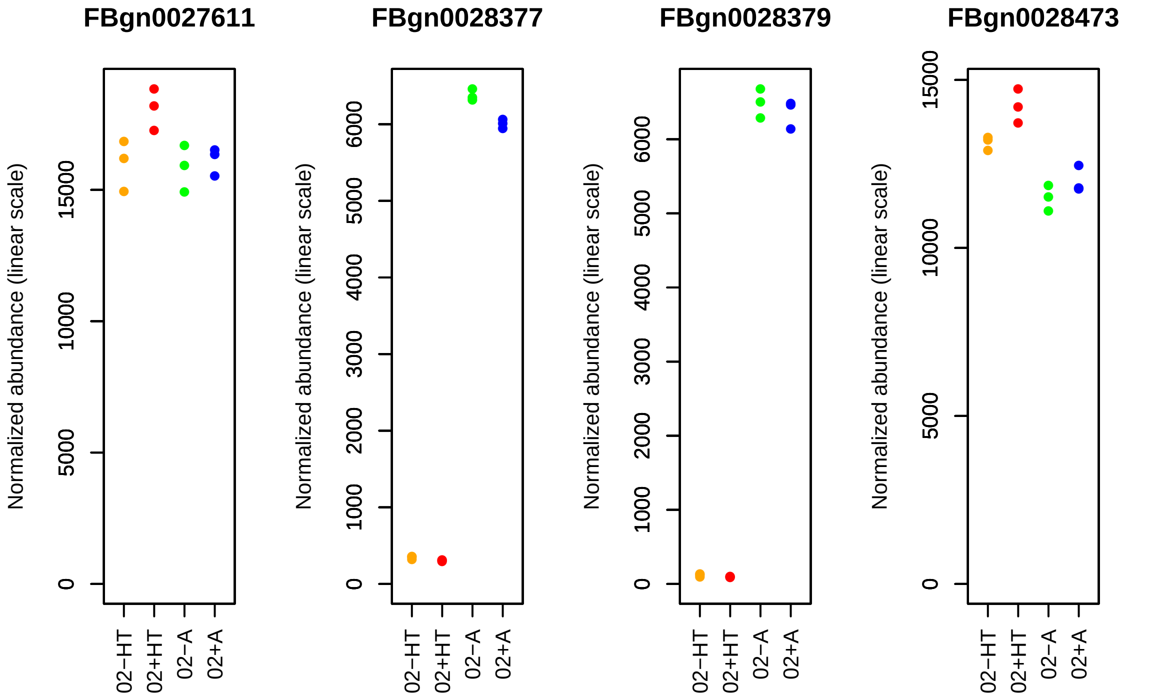

FBgn0027611
FBgn0028377
FBgn0028379
FBgn0028473
Normalized abundance (linear scale)
0
5000
10000
15000
0
1000
2000
3000
4000
5000
6000
02−HT
02+HT
02−A
02+A

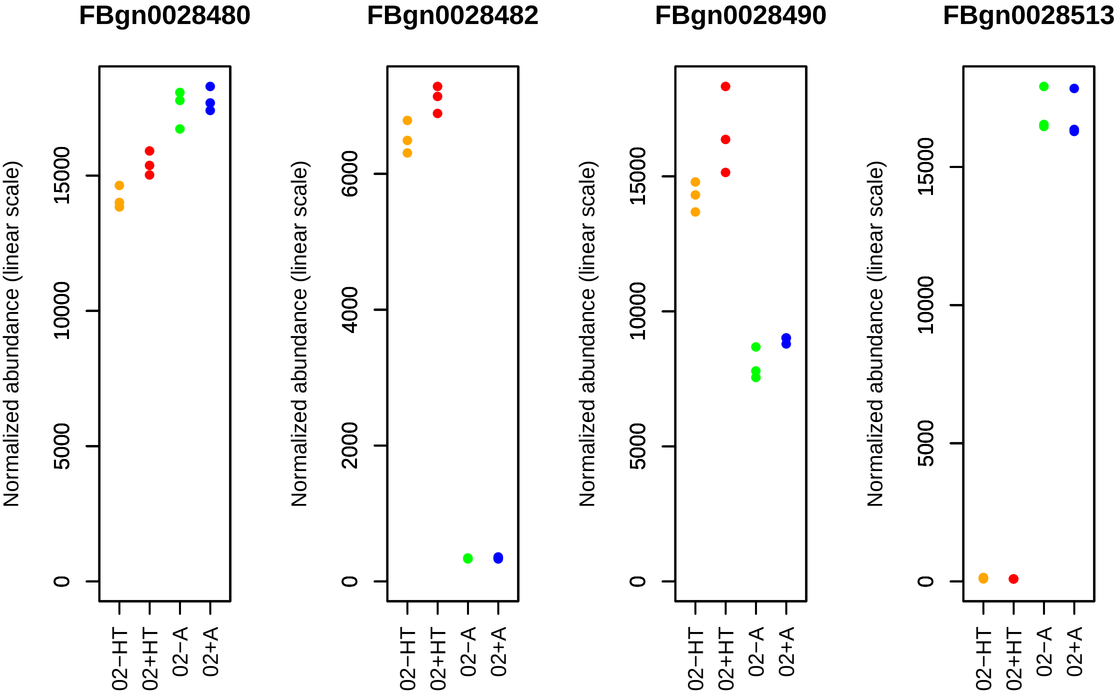
FBgn0028480
FBgn0028482
FBgn0028490
FBgn0028513
Normalized abundance (linear scale)
0
5000
10000
15000
0
2000
4000
6000
02−HT
02+HT
02−A
02+A

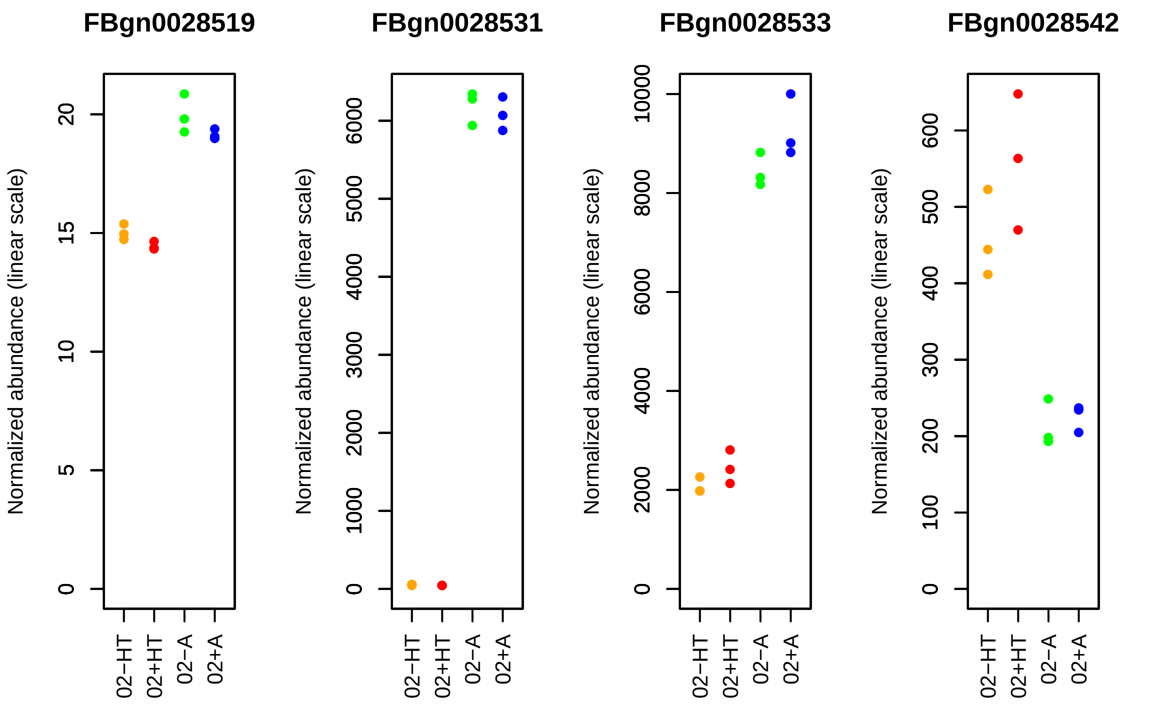

FBgn0028519
FBgn0028531
FBgn0028533
FBgn0028542
Normalized abundance (linear scale)
0
5
10
15
20
0
1000
2000
3000
4000
5000
6000
0
2000
4000
6000
8000
10000
0
100
200
300
400
500
600
02−HT
02+HT
02−A
02+A

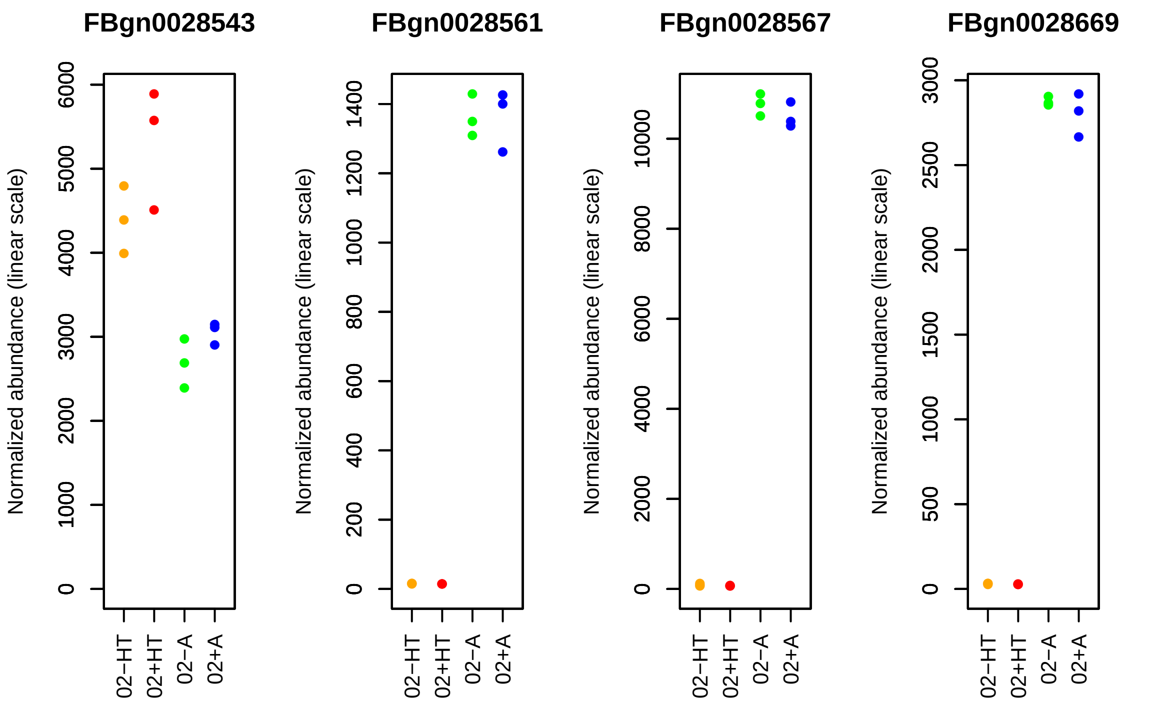
FBgn0028543
FBgn0028561
FBgn0028567
FBgn0028669
Normalized abundance (linear scale)
0
1000
2000
3000
4000
5000
6000
200
400
600
800
1200
1400
10000
8000
500
1500
2000
2500
3000
02−HT
02+HT
02−A
02+A

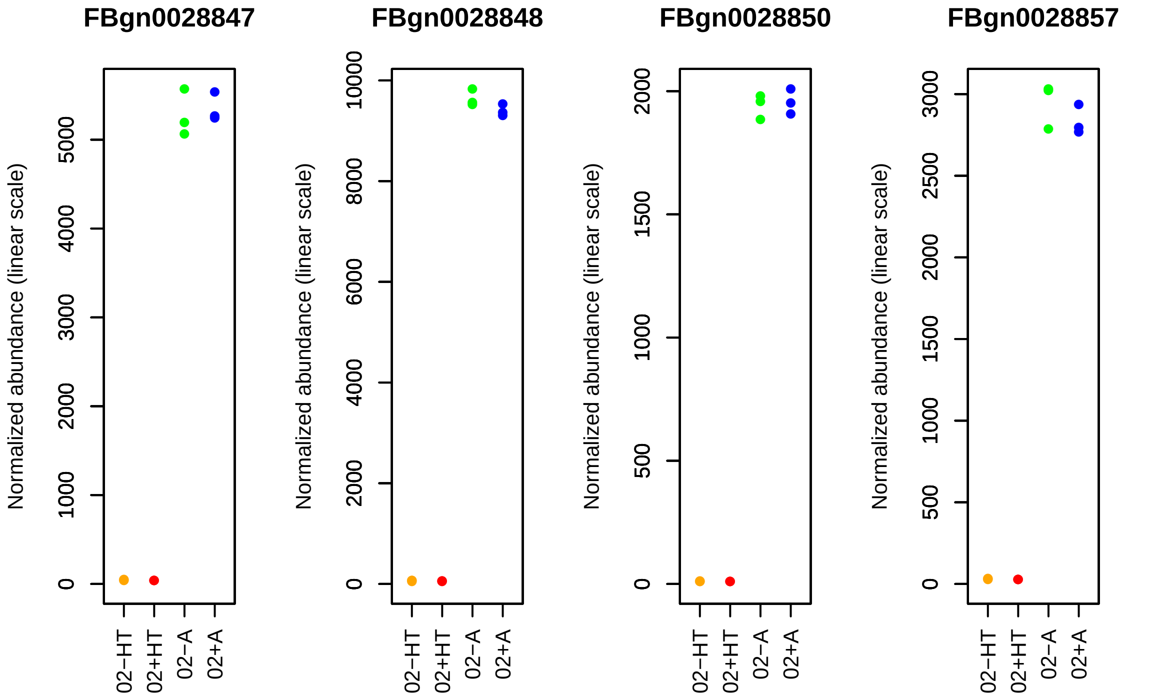

FBgn0028847
FBgn0028848
FBgn0028850
FBgn0028857
Normalized abundance (linear scale)
0
1000
2000
3000
4000
5000
0
2000
4000
6000
8000
10000
0
500
1000
1500
2000
0
500
1000
1500
2000
2500
3000
02−HT
02+HT
02−A
02+A

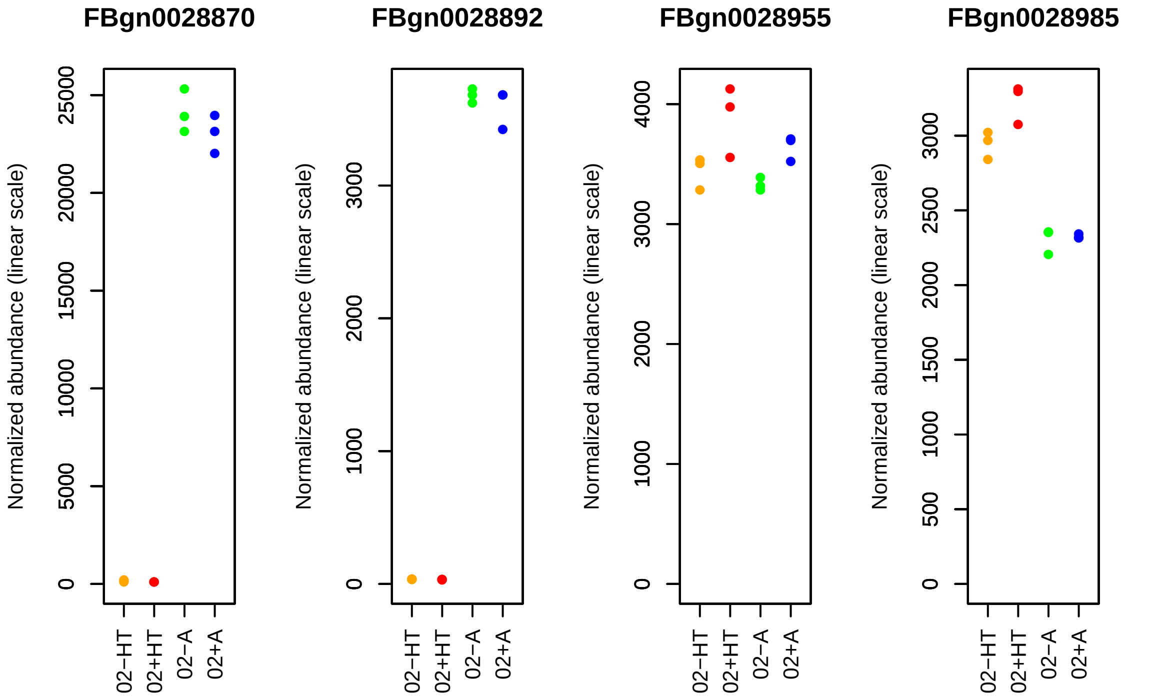

FBgn0028870
FBgn0028892
FBgn0028955
FBgn0028985
Normalized abundance (linear scale)
0
5000
10000
15000
20000
25000
1000
2000
3000
4000
500
1500
2500
02−HT
02+HT
02−A
02+A

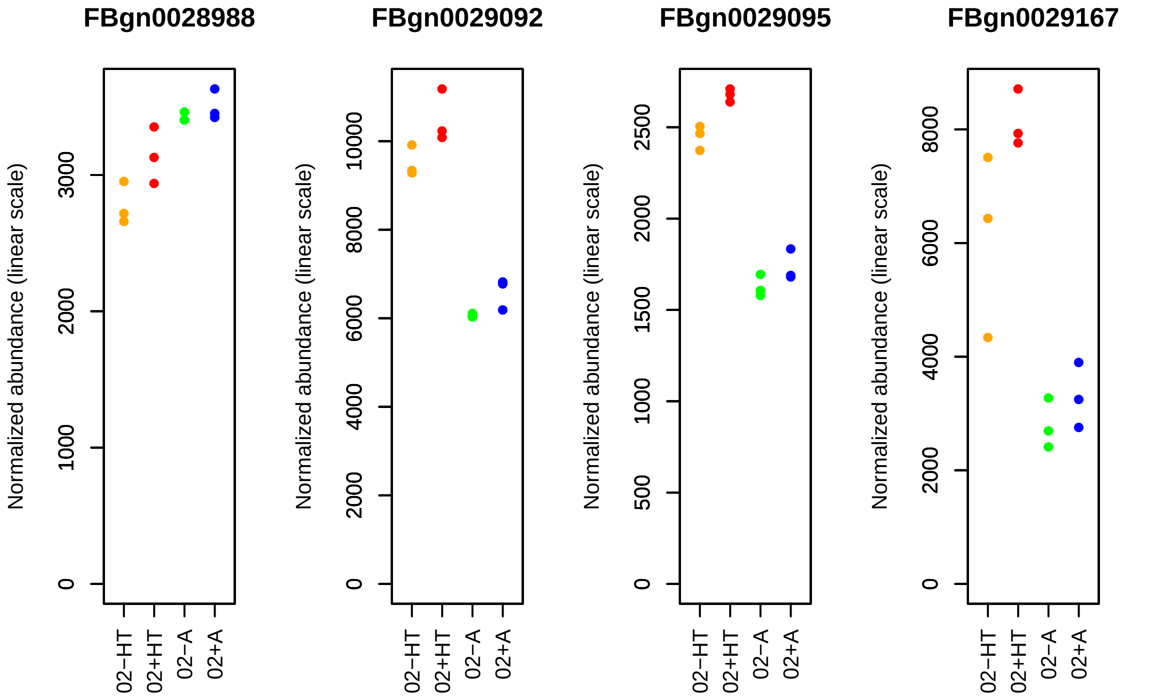

FBgn0028988
FBgn0029092
FBgn0029095
FBgn0029167
Normalized abundance (linear scale)
0
1000
2000
3000
0
2000
4000
6000
8000
10000
0
500
1000
1500
2000
2500
0
2000
4000
6000
8000
02−HT
02+HT
02−A
02+A

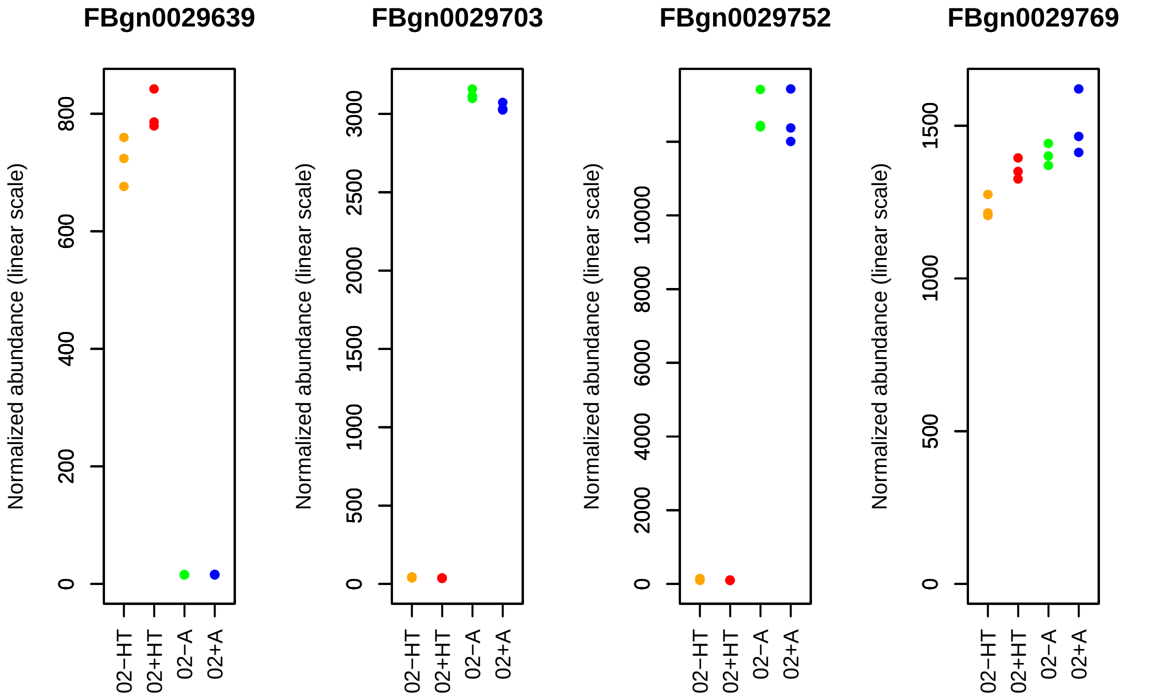
FBgn0029639
FBgn0029703
FBgn0029752
FBgn0029769
Normalized abundance (linear scale)
0
200
400
600
800
500
1000
1500
2000
2500
3000
2000
4000
6000
8000
10000
02−HT
02+HT
02−A
02+A

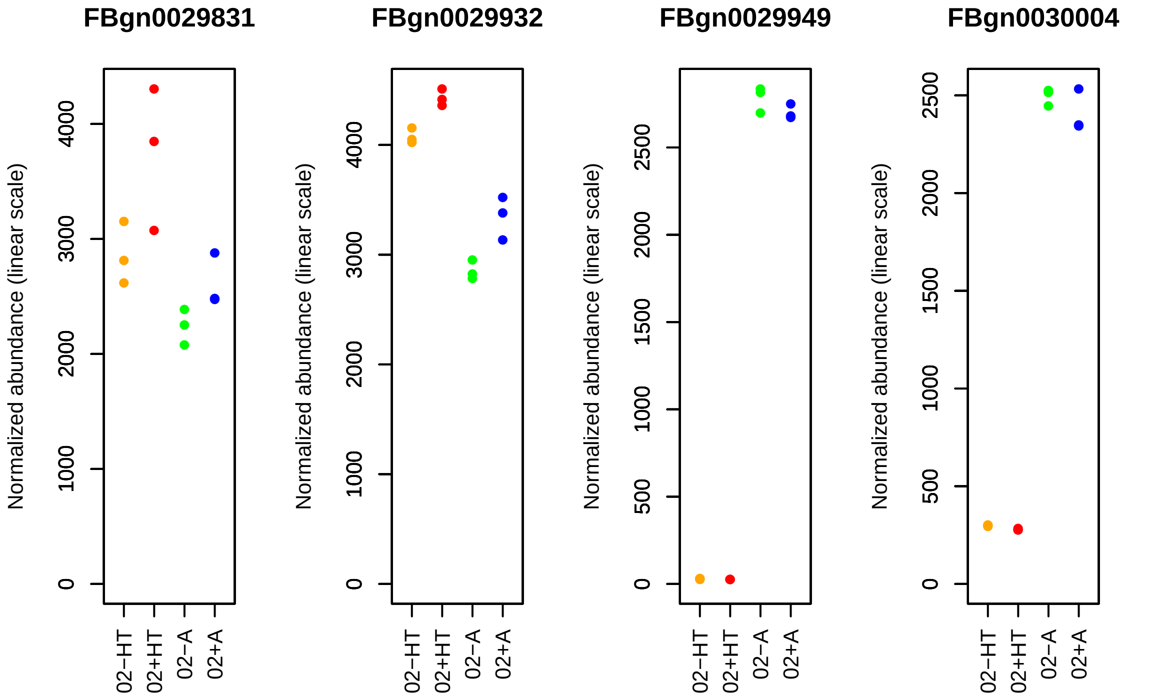
FBgn0029831
FBgn0029932
FBgn0029949
FBgn0030004
Normalized abundance (linear scale)
0
1000
2000
3000
4000
500
1500
2500
02−HT
02+HT
02−A
02+A

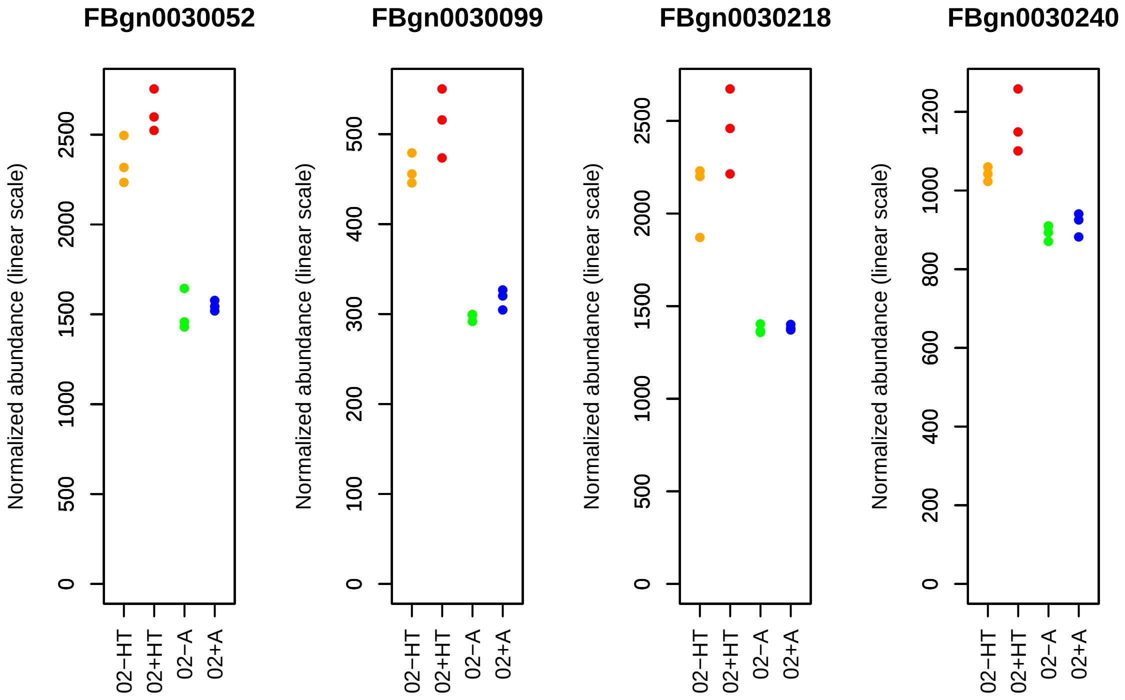

FBgn0030052
FBgn0030099
FBgn0030218
FBgn0030240
Normalized abundance (linear scale)
02−HT
02+HT
02−A
02+A

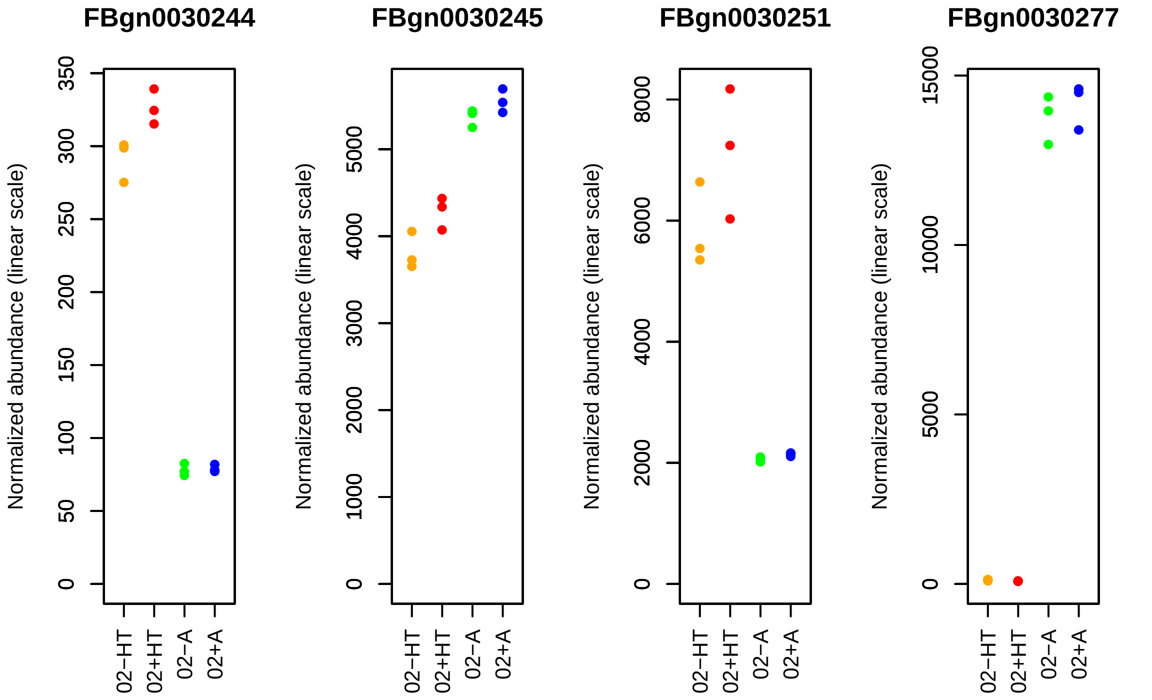
FBgn0030244
FBgn0030245
FBgn0030251
FBgn0030277
Normalized abundance (linear scale)
02−HT
02+HT
02−A
02+A

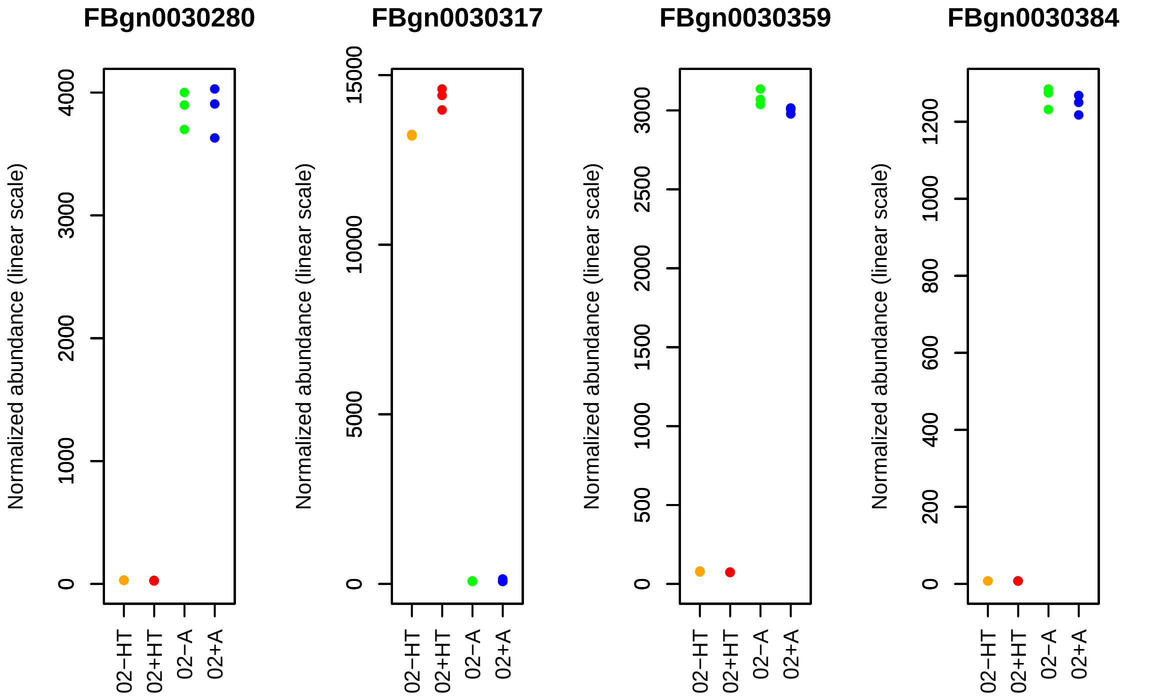
FBgn0030280
FBgn0030317
FBgn0030359
FBgn0030384
Normalized abundance (linear scale)
0
1000
2000
3000
4000
5000
10000
15000
500
1500
2000
2500
200
400
600
800
1200
02−HT
02+HT
02−A
02+A

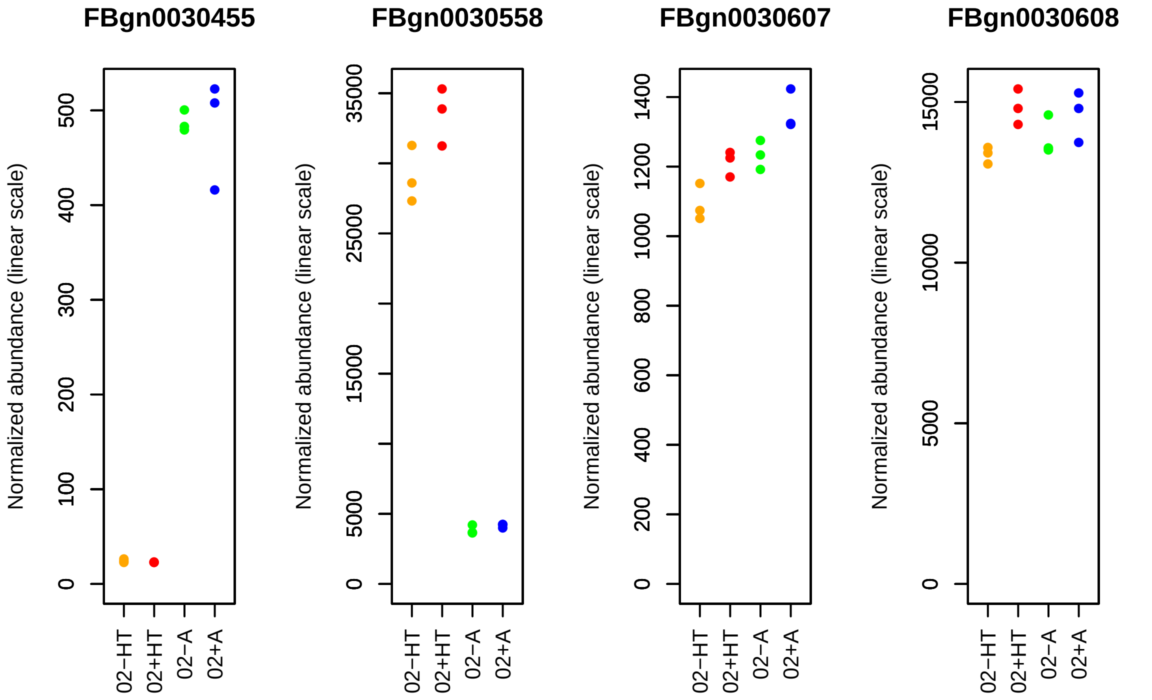
FBgn0030455
FBgn0030558
FBgn0030607
FBgn0030608
Normalized abundance (linear scale)
0
100
200
300
400
500
5000
15000
25000
35000
200
400
600
800
1000
1200
1400
10000
02−HT
02+HT
02−A
02+A

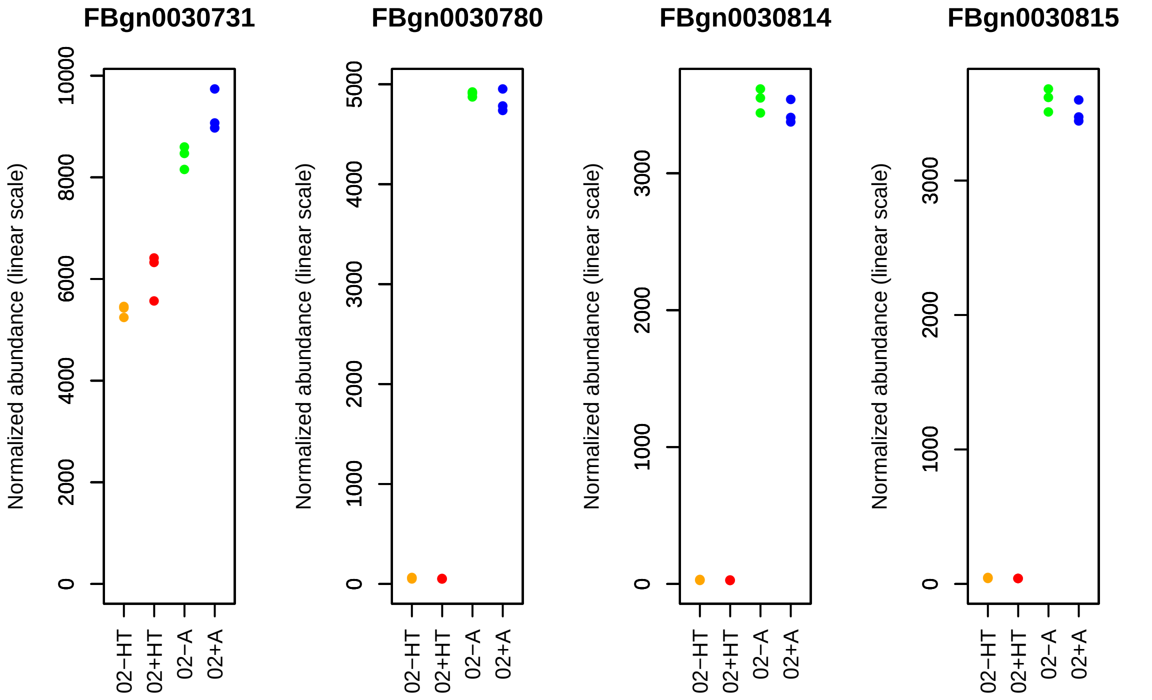

FBgn0030731
FBgn0030780
FBgn0030814
FBgn0030815
Normalized abundance (linear scale)
0
2000
4000
6000
8000
10000
1000
3000
5000
02−HT
02+HT
02−A
02+A

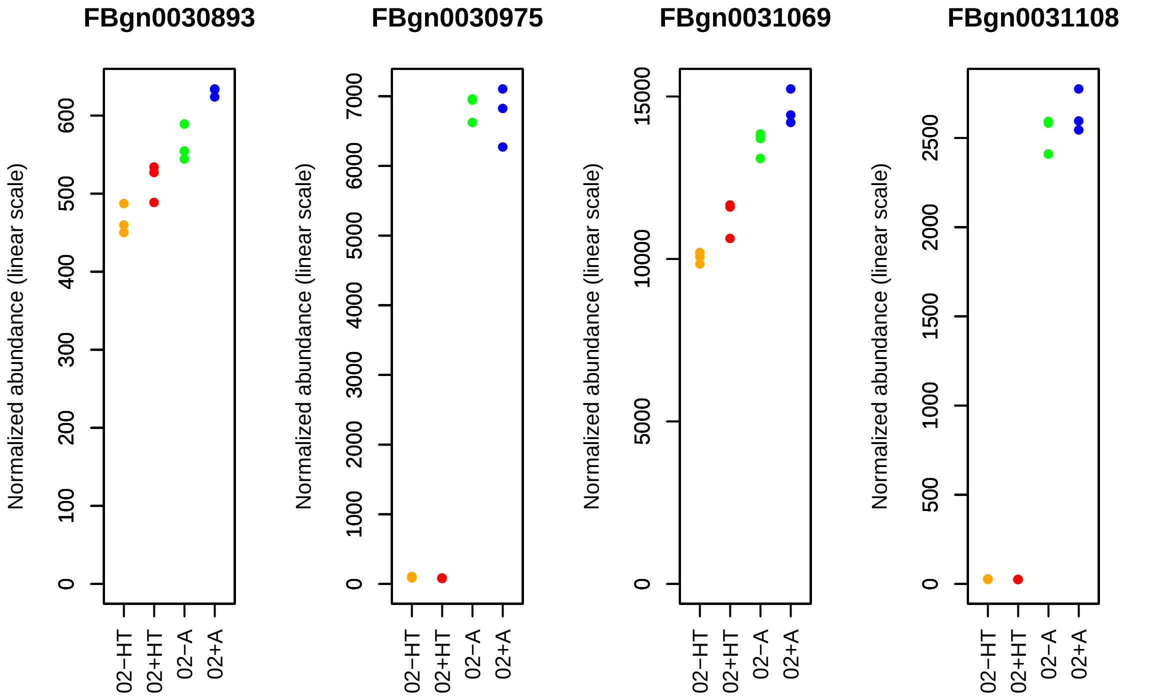

FBgn0030893
FBgn0030975
FBgn0031069
FBgn0031108
Normalized abundance (linear scale)
0
100
200
300
400
500
600
1000
2000
3000
4000
5000
6000
7000
10000
15000
1500
2500
02−HT
02+HT
02−A
02+A

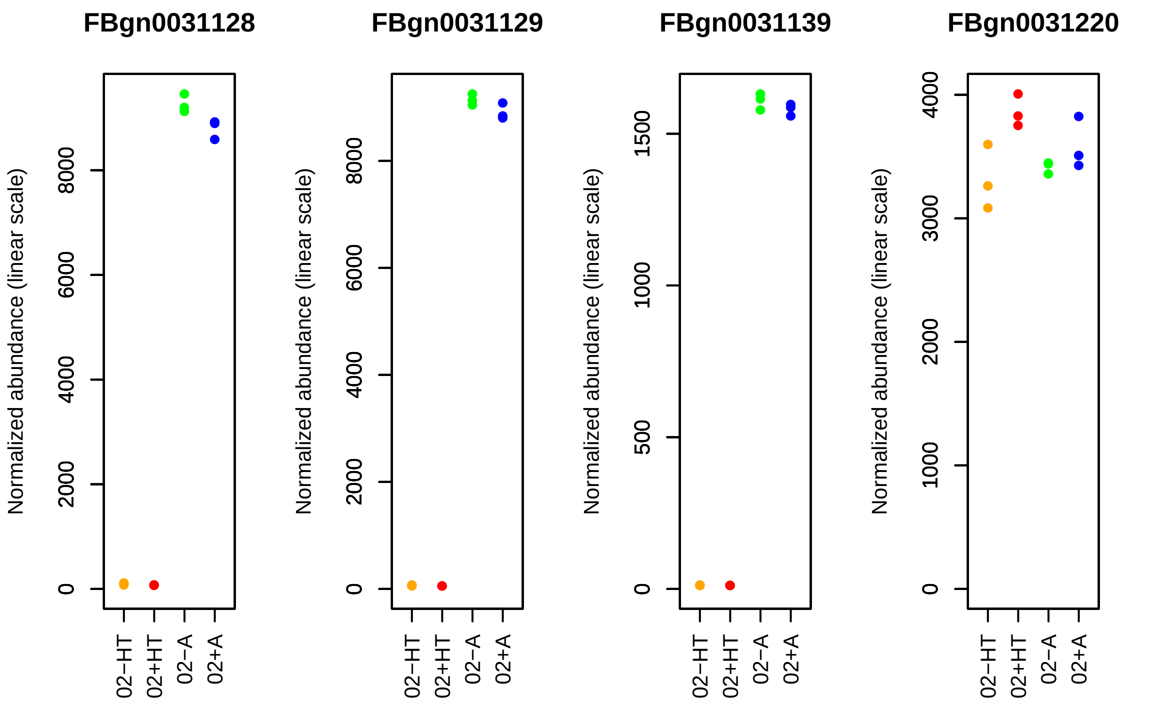
FBgn0031128
FBgn0031129
FBgn0031139
FBgn0031220
Normalized abundance (linear scale)
0
2000
4000
6000
8000
500
1000
1500
3000
02−HT
02+HT
02−A
02+A

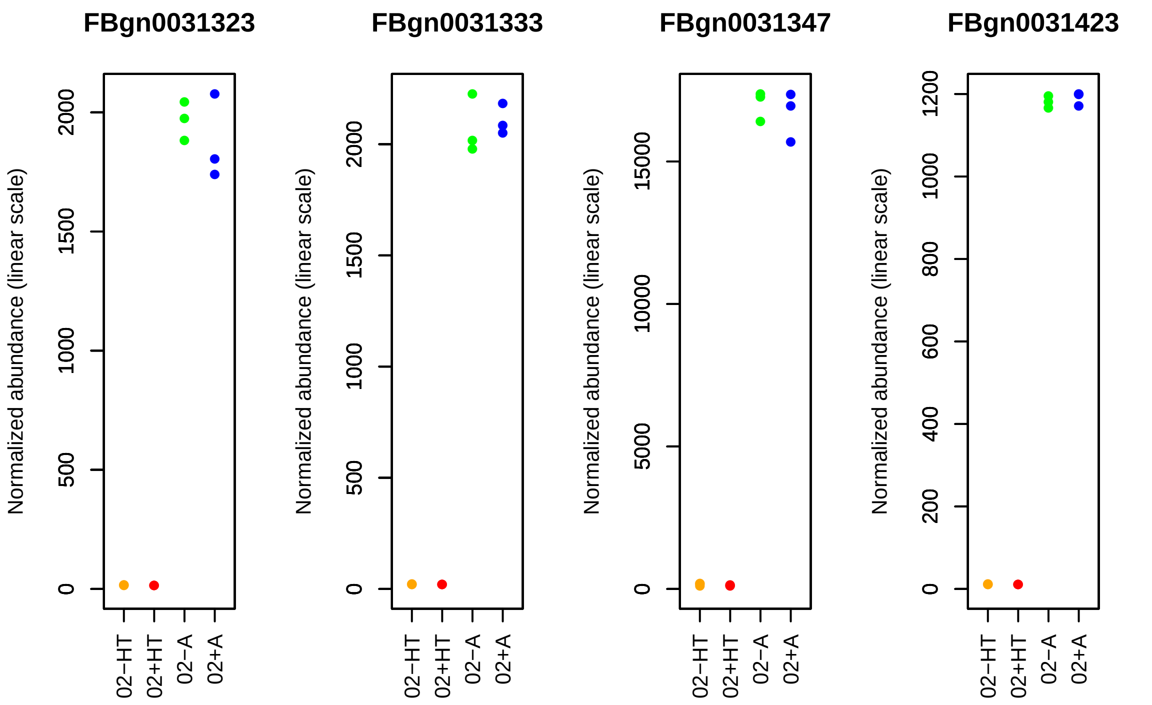

FBgn0031323
FBgn0031333
FBgn0031347
FBgn0031423
Normalized abundance (linear scale)
0
500
1000
1500
2000
5000
10000
15000
200
400
600
800
1200
02−HT
02+HT
02−A
02+A

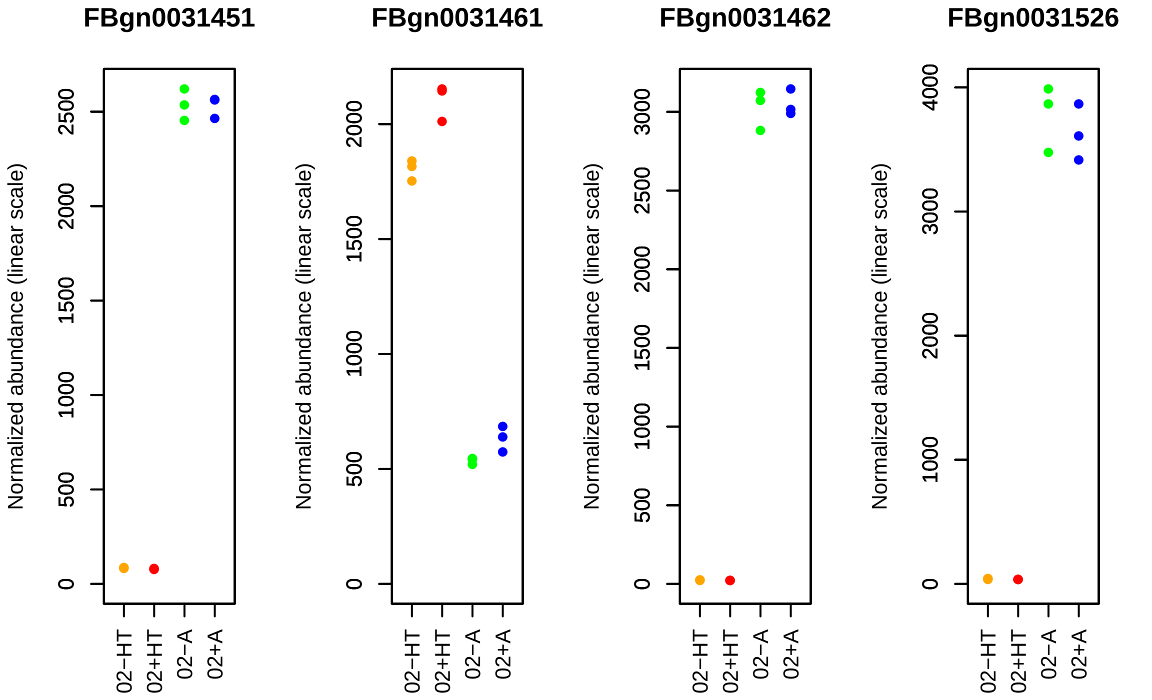
FBgn0031451
FBgn0031461
FBgn0031462
FBgn0031526
Normalized abundance (linear scale)
0
500
1000
1500
2000
2500
3000
4000
02−HT
02+HT
02−A
02+A

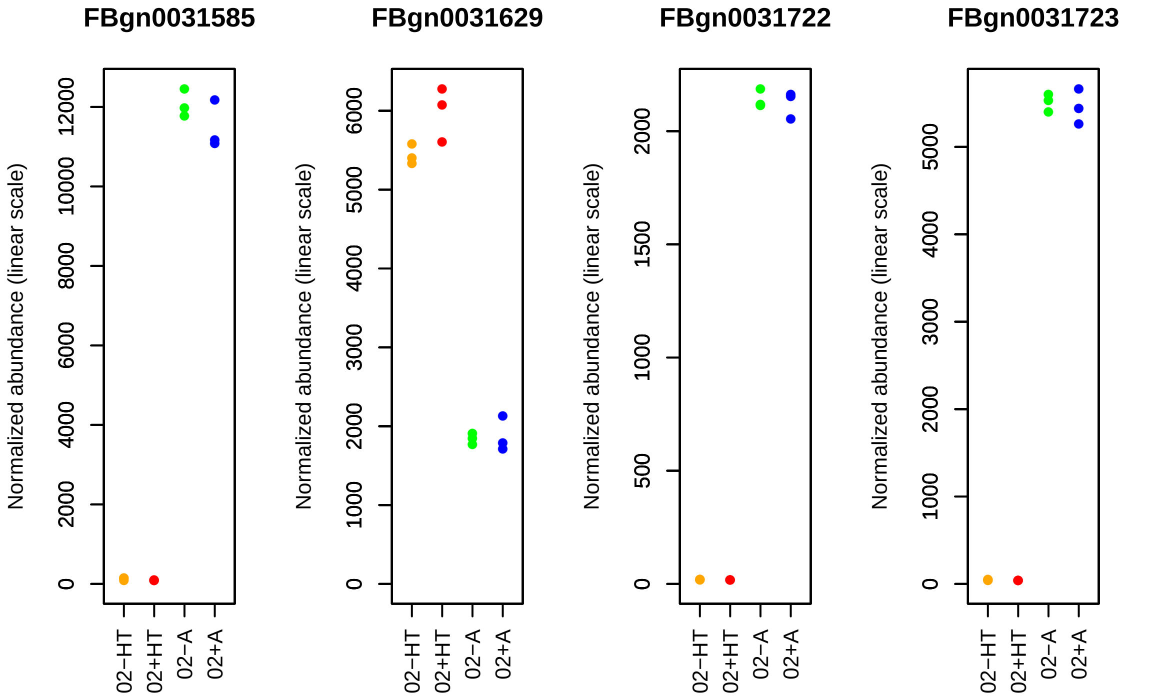

FBgn0031585
FBgn0031629
FBgn0031722
FBgn0031723
Normalized abundance (linear scale)
0
2000
4000
6000
8000
10000
12000
1000
3000
5000
500
1500
02−HT
02+HT
02−A
02+A

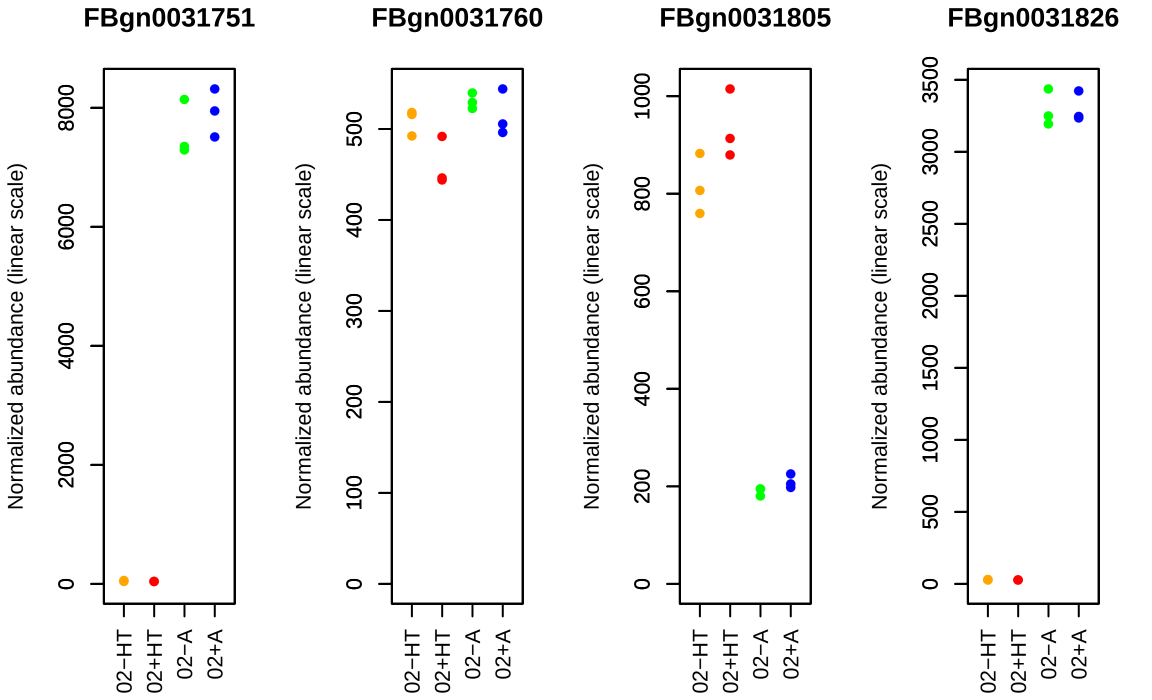

FBgn0031751
FBgn0031760
FBgn0031805
FBgn0031826
Normalized abundance (linear scale)
0
2000
4000
6000
8000
100
200
300
400
500
600
800
1000
500
1000
1500
2000
2500
3000
3500
02−HT
02+HT
02−A
02+A

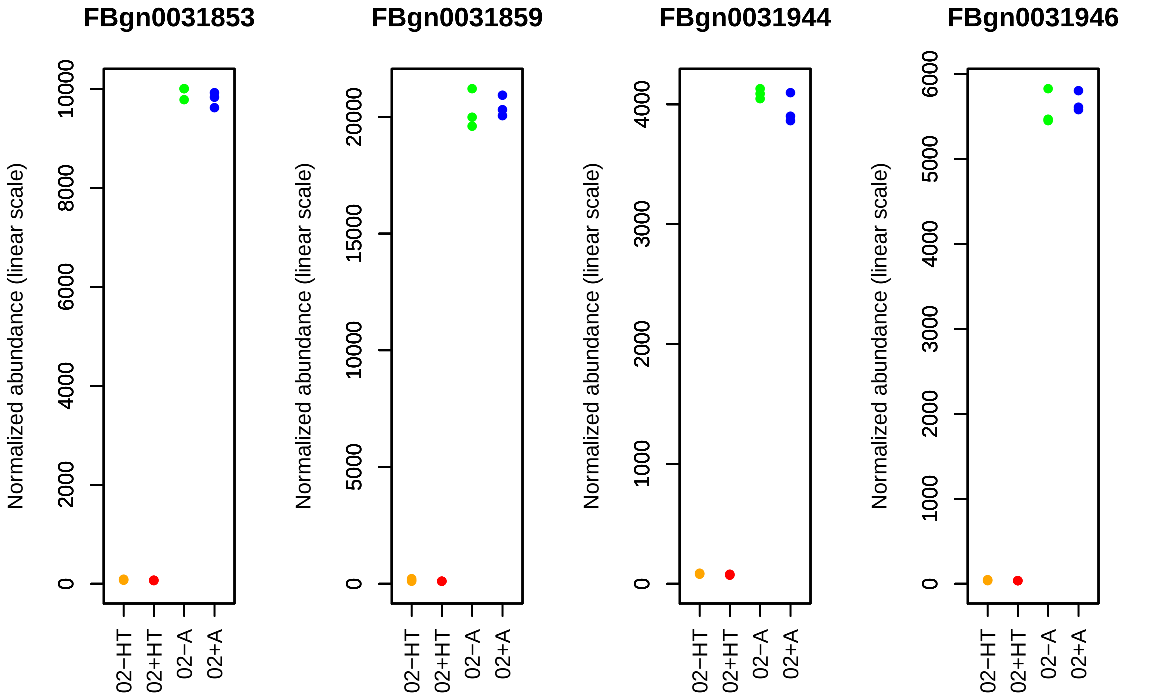
FBgn0031853
FBgn0031859
FBgn0031944
FBgn0031946
Normalized abundance (linear scale)
0
2000
4000
6000
8000
10000
0
5000
10000
15000
20000
0
1000
2000
3000
4000
0
1000
2000
3000
4000
5000
6000
02−HT
02+HT
02−A
02+A

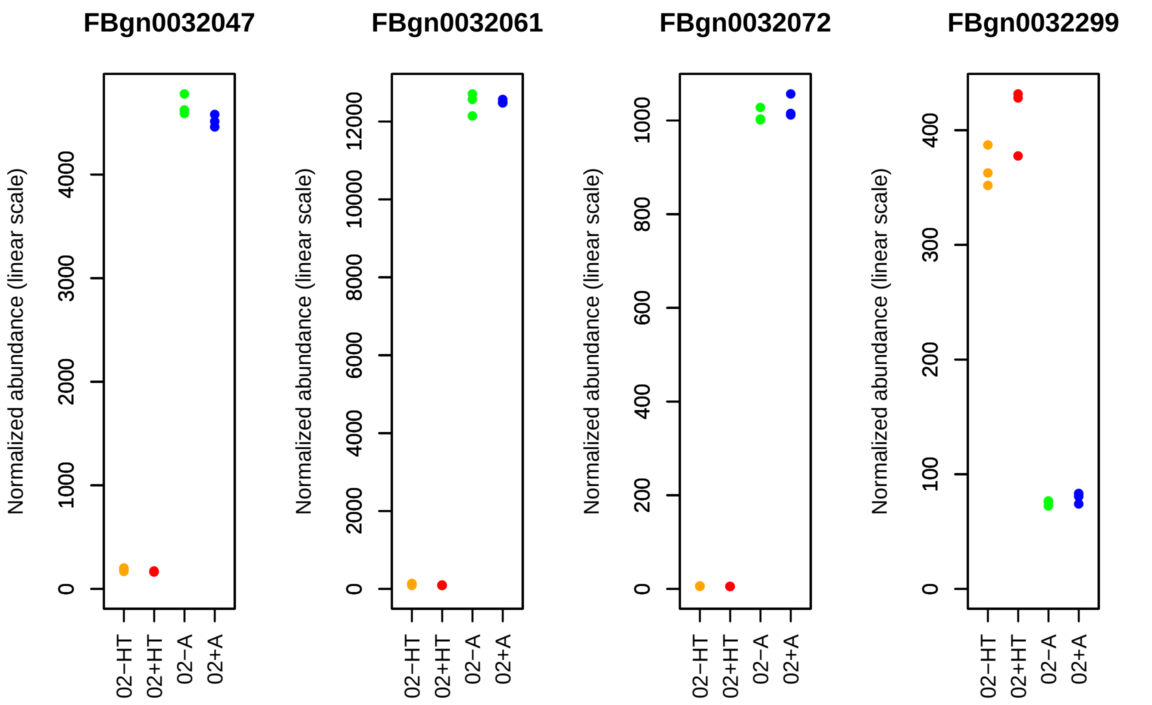
FBgn0032047
FBgn0032061
FBgn0032072
FBgn0032299
Normalized abundance (linear scale)
0
1000
2000
3000
4000
2000
4000
6000
8000
10000
12000
200
400
600
800
1000
100
200
300
400
02−HT
02+HT
02−A
02+A

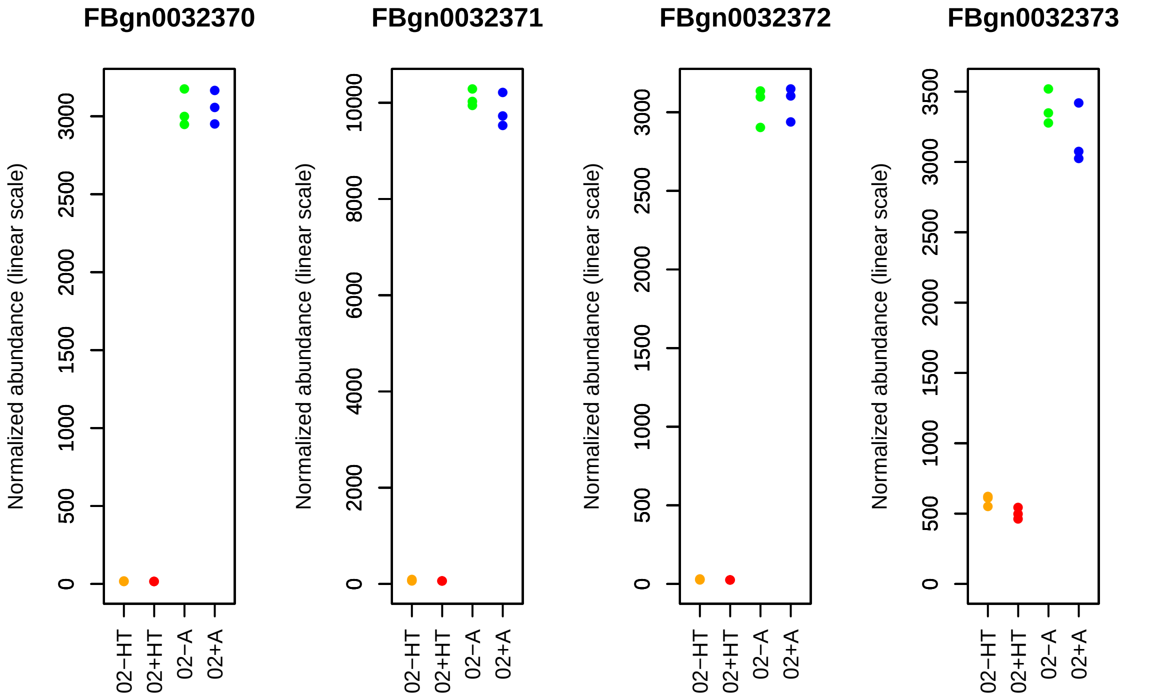

FBgn0032370
FBgn0032371
FBgn0032372
FBgn0032373
Normalized abundance (linear scale)
0
500
1000
1500
2000
2500
3000
3500
2000
4000
6000
8000
10000
02−HT
02+HT
02−A
02+A

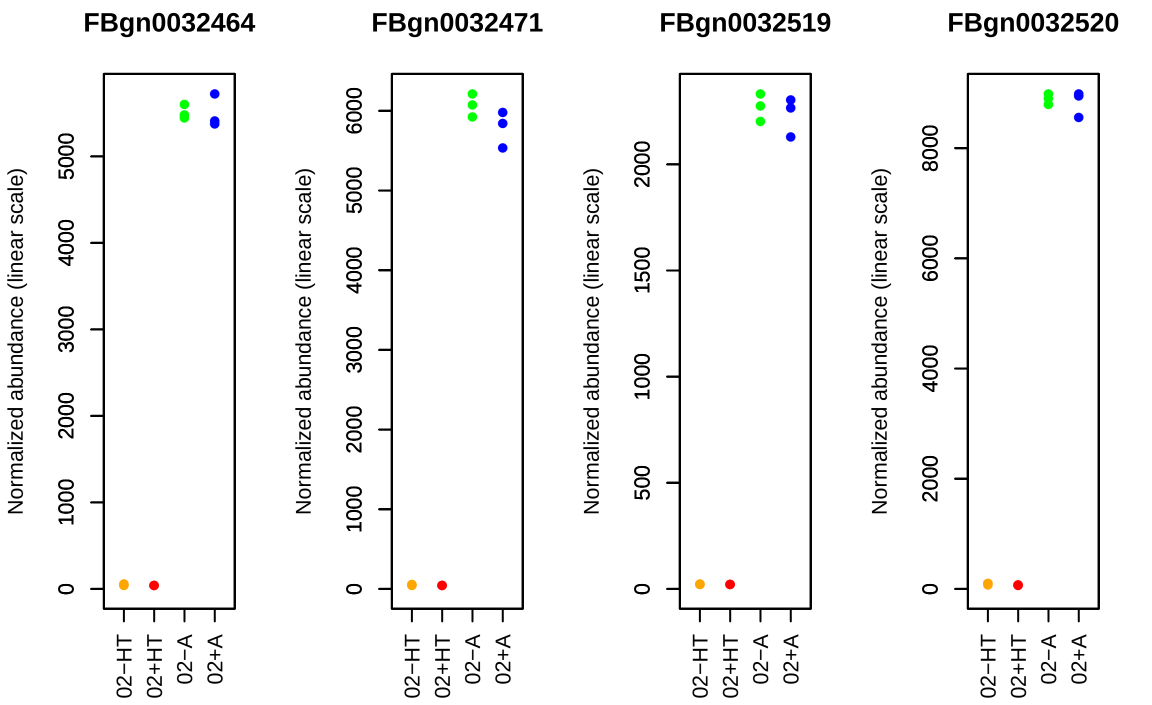

FBgn0032464
FBgn0032471
FBgn0032519
FBgn0032520
Normalized abundance (linear scale)
0
1000
2000
3000
4000
5000
6000
8000
500
1500
02−HT
02+HT
02−A
02+A

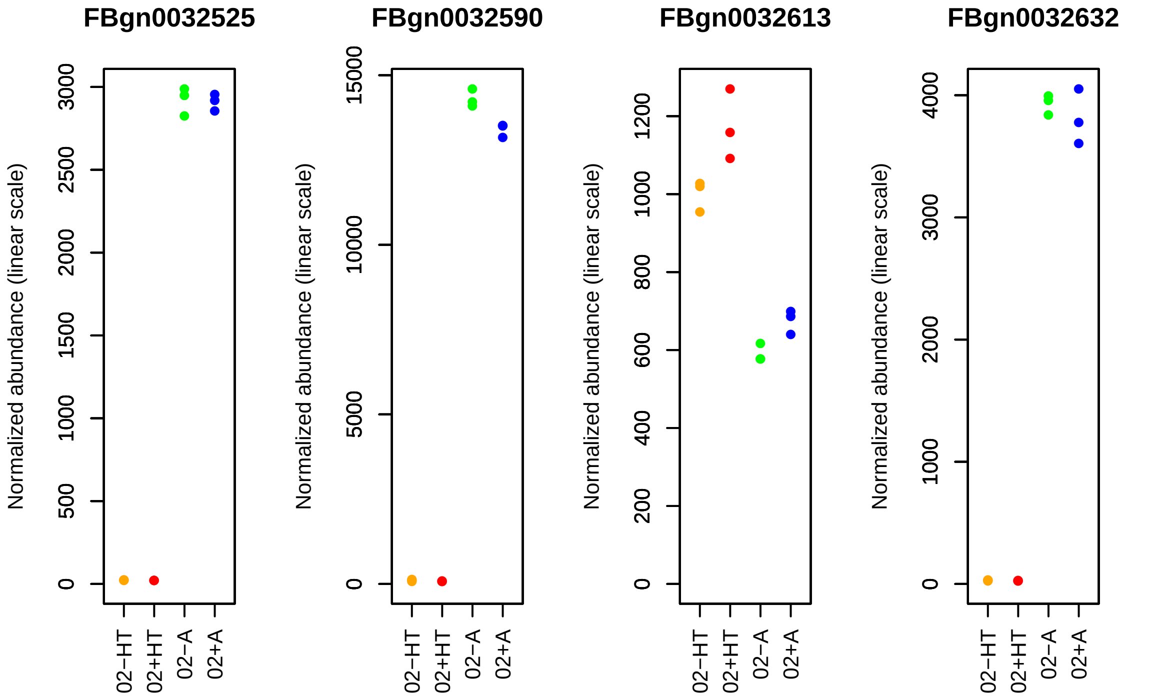

FBgn0032525
FBgn0032590
FBgn0032613
FBgn0032632
Normalized abundance (linear scale)
0
500
1000
1500
2000
2500
3000
5000
10000
15000
200
400
600
800
1200
4000
02−HT
02+HT
02−A
02+A

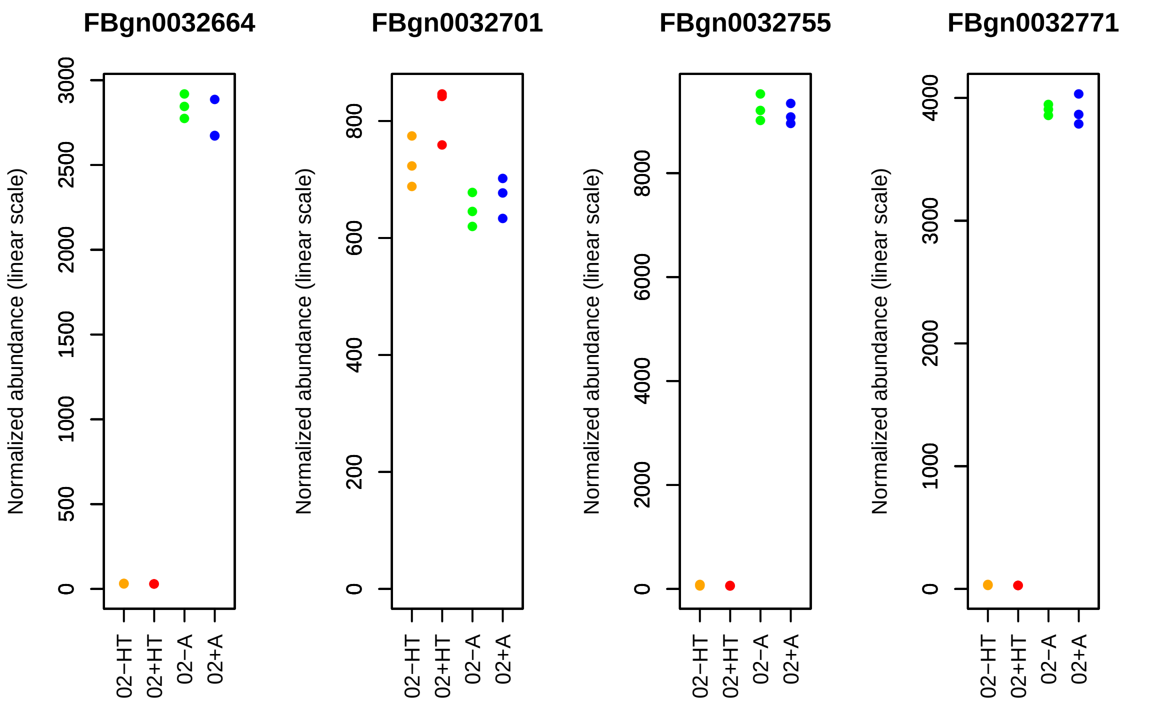
FBgn0032664
FBgn0032701
FBgn0032755
FBgn0032771
Normalized abundance (linear scale)
0
500
1000
1500
2000
2500
3000
200
400
600
800
2000
4000
6000
8000
1000
3000
4000
02−HT
02+HT
02−A
02+A

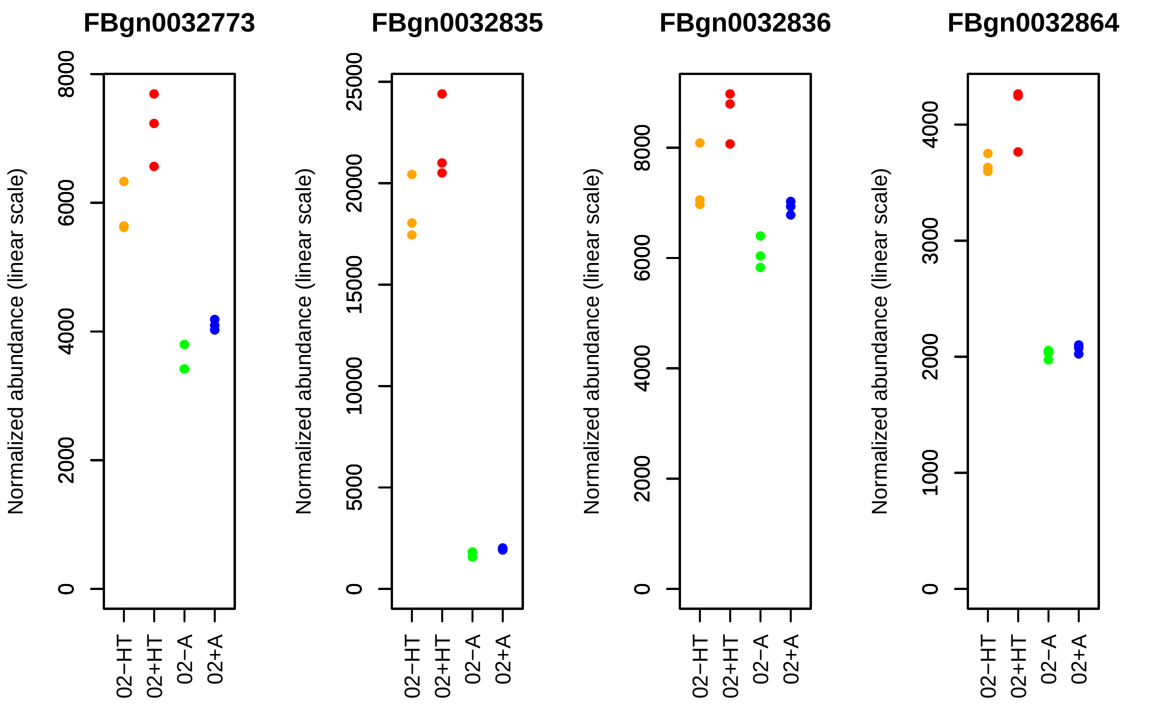

FBgn0032773
FBgn0032835
FBgn0032836
FBgn0032864
Normalized abundance (linear scale)
0
2000
4000
6000
8000
5000
10000
15000
20000
25000
1000
3000
02−HT
02+HT
02−A
02+A

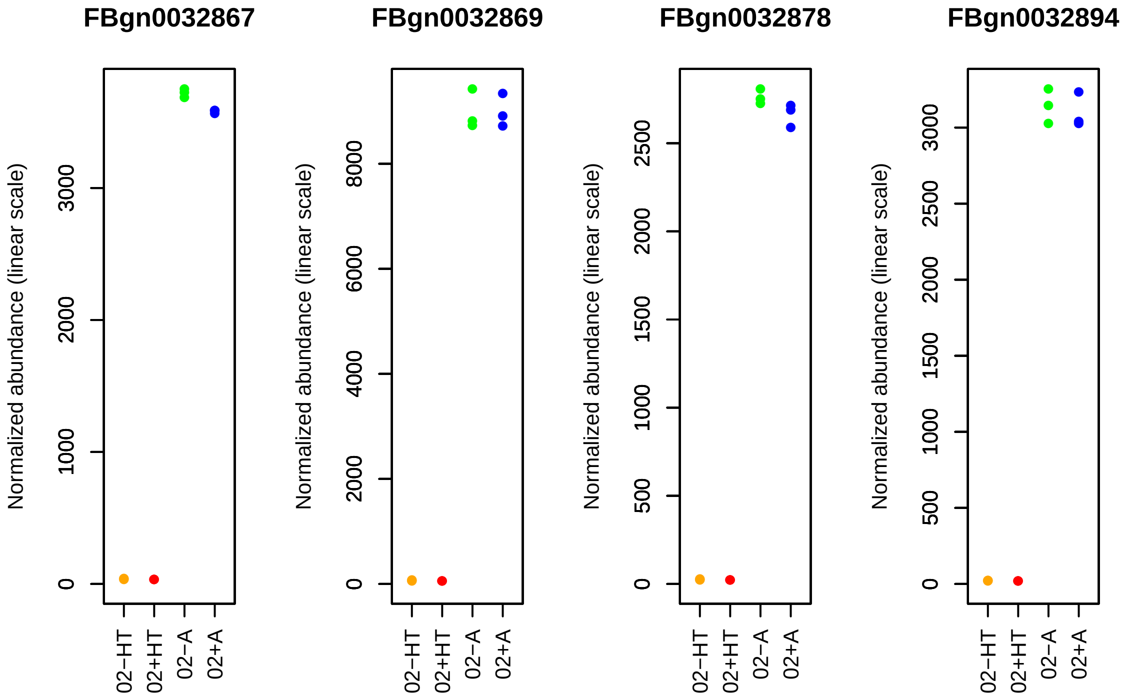

FBgn0032867
FBgn0032869
FBgn0032878
FBgn0032894
Normalized abundance (linear scale)
0
1000
2000
3000
0
2000
4000
6000
8000
0
500
1000
1500
2000
2500
0
500
1000
1500
2000
2500
3000
02−HT
02+HT
02−A
02+A

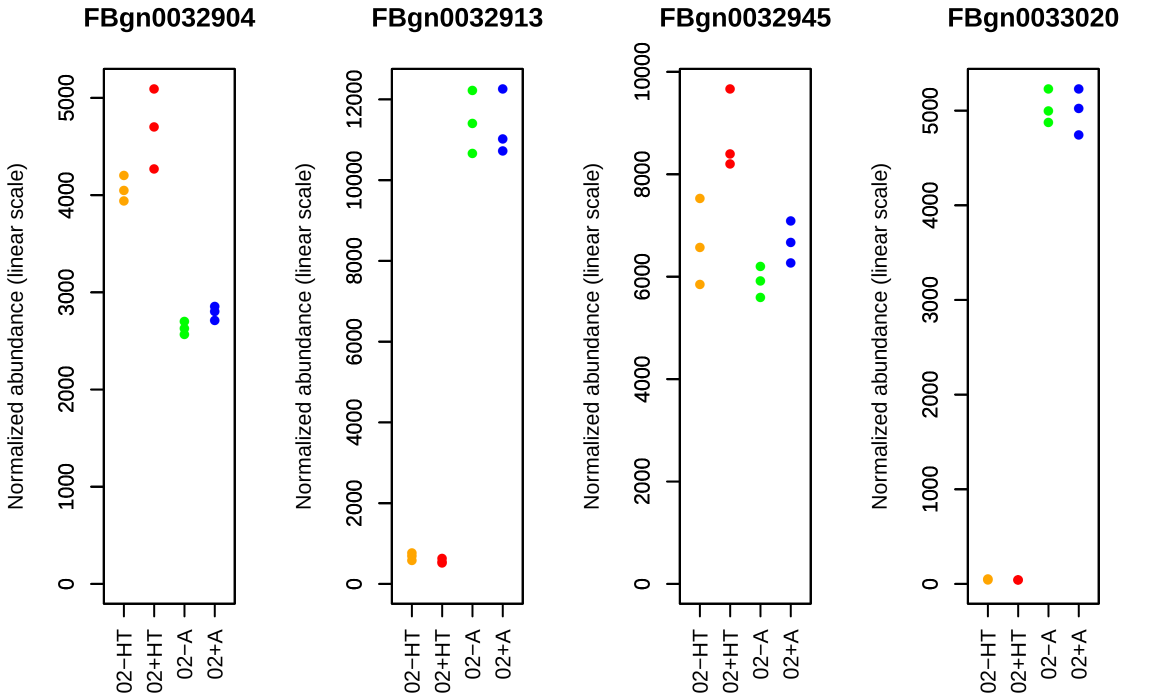

FBgn0032904
FBgn0032913
FBgn0032945
FBgn0033020
Normalized abundance (linear scale)
0
1000
2000
3000
4000
5000
6000
8000
10000
12000
02−HT
02+HT
02−A
02+A

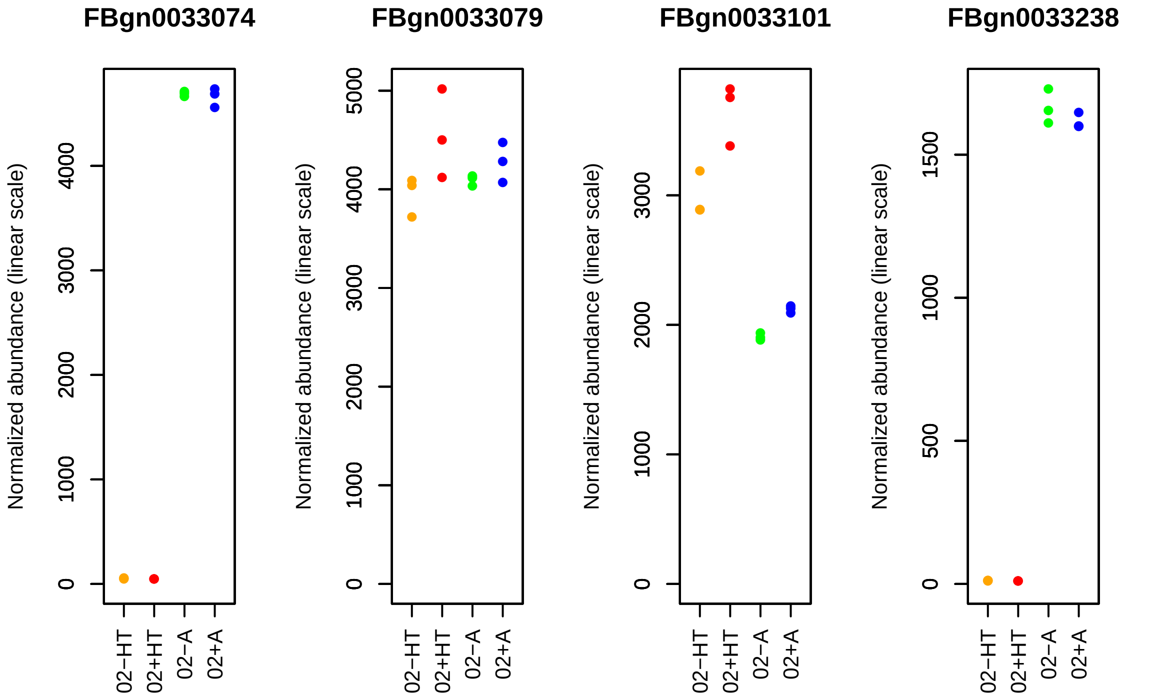
FBgn0033074
FBgn0033079
FBgn0033101
FBgn0033238
Normalized abundance (linear scale)
0
1000
2000
3000
4000
5000
500
1500
02−HT
02+HT
02−A
02+A

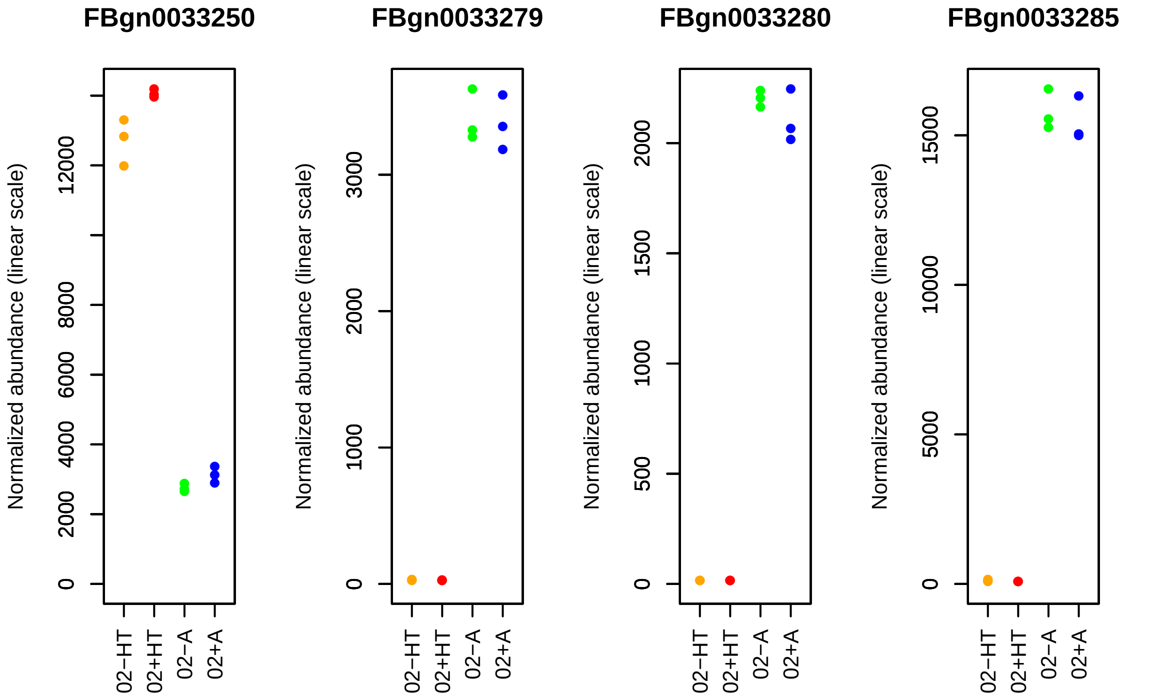

FBgn0033250
FBgn0033279
FBgn0033280
FBgn0033285
Normalized abundance (linear scale)
0
2000
4000
6000
8000
12000
1000
3000
500
1500
5000
10000
15000
02−HT
02+HT
02−A
02+A

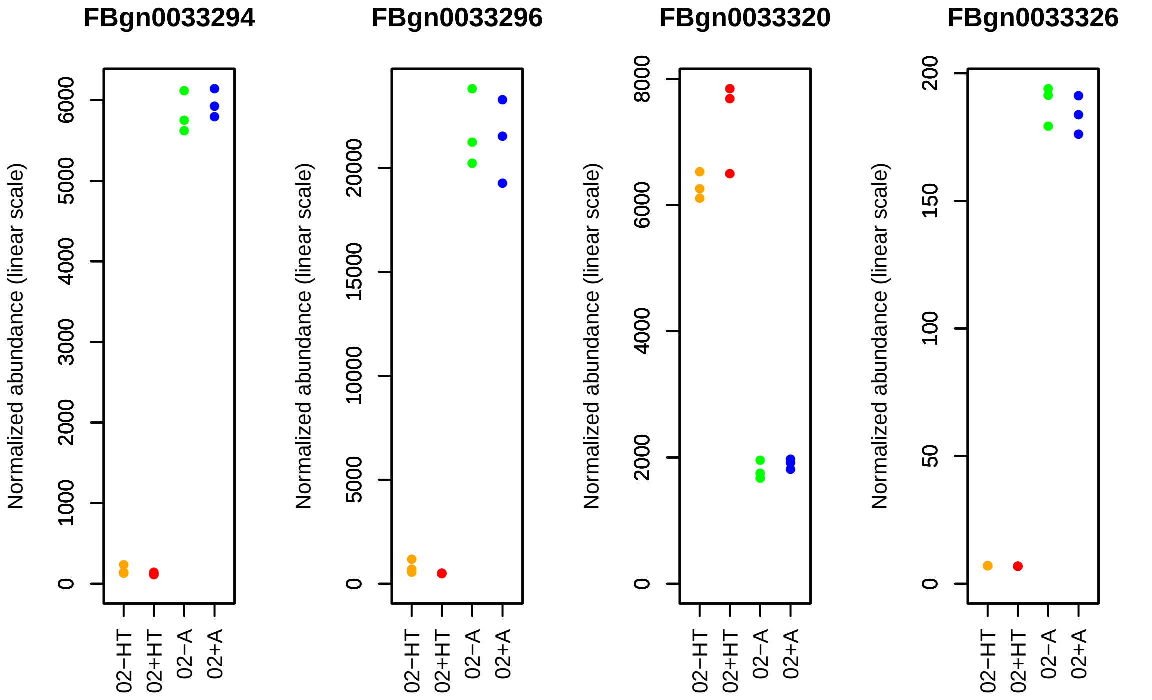

FBgn0033294
FBgn0033296
FBgn0033320
FBgn0033326
Normalized abundance (linear scale)
0
1000
2000
3000
4000
5000
6000
5000
10000
15000
20000
8000
150
100
50
200
02−HT
02+HT
02−A
02+A

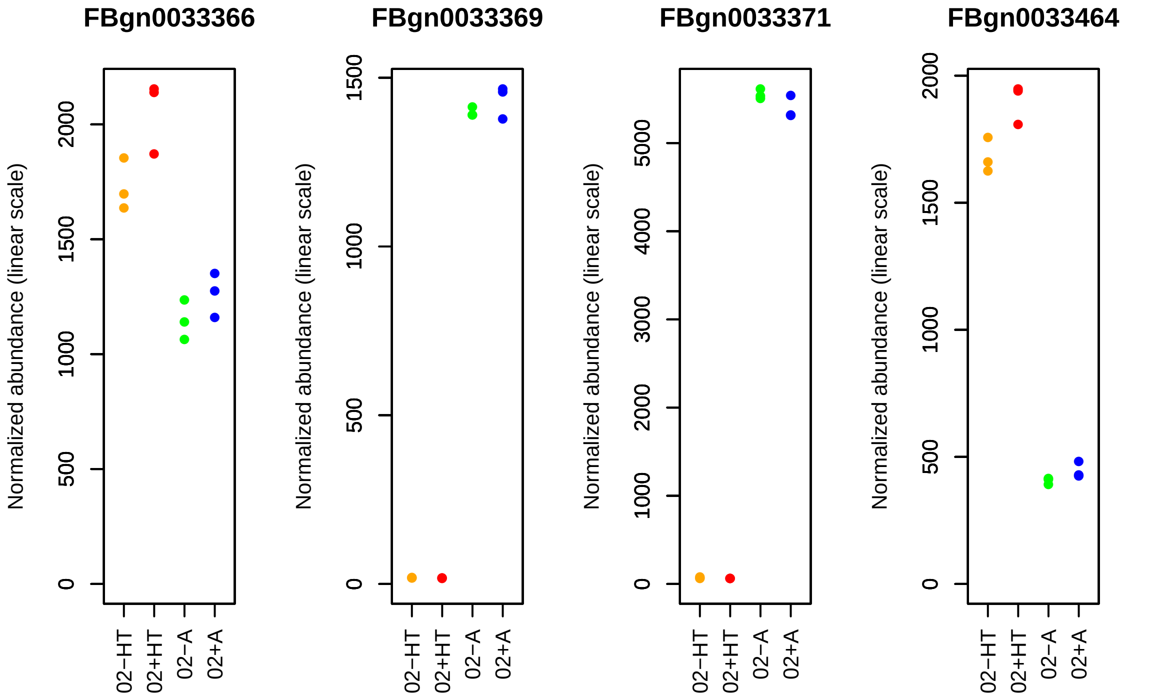

FBgn0033366
FBgn0033369
FBgn0033371
FBgn0033464
Normalized abundance (linear scale)
0
500
1000
1500
2000
5000
4000
3000
02−HT
02+HT
02−A
02+A

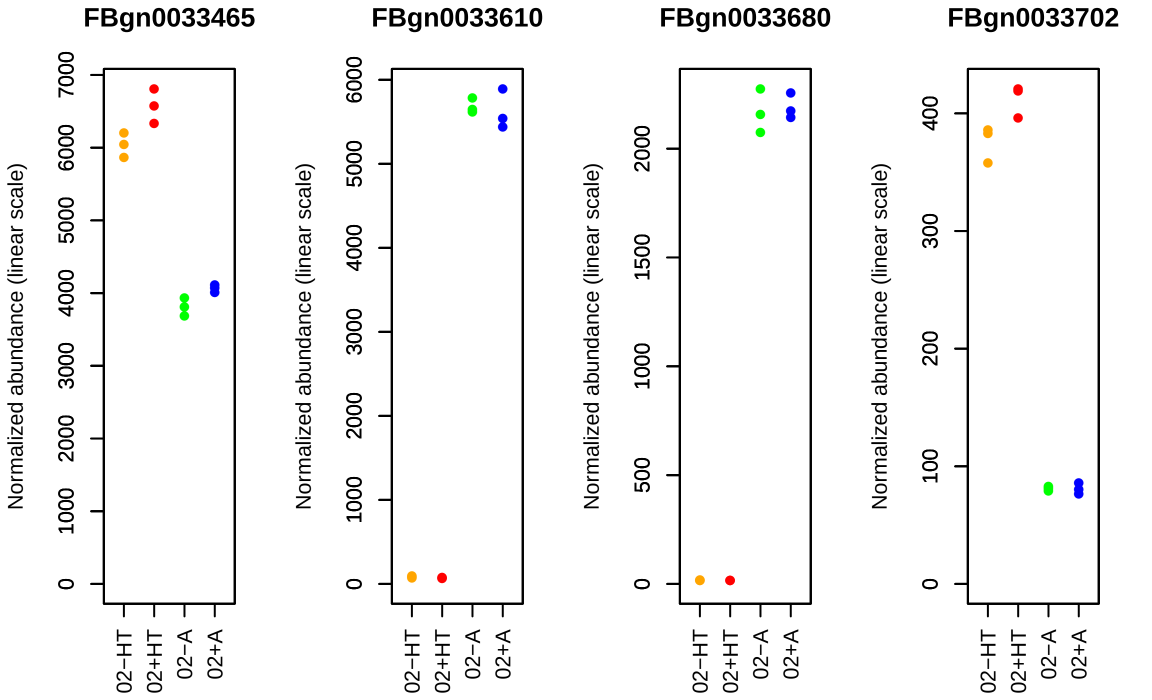
FBgn0033465
FBgn0033610
FBgn0033680
FBgn0033702
Normalized abundance (linear scale)
0
1000
2000
3000
4000
5000
6000
7000
500
1500
100
200
300
400
02−HT
02+HT
02−A
02+A

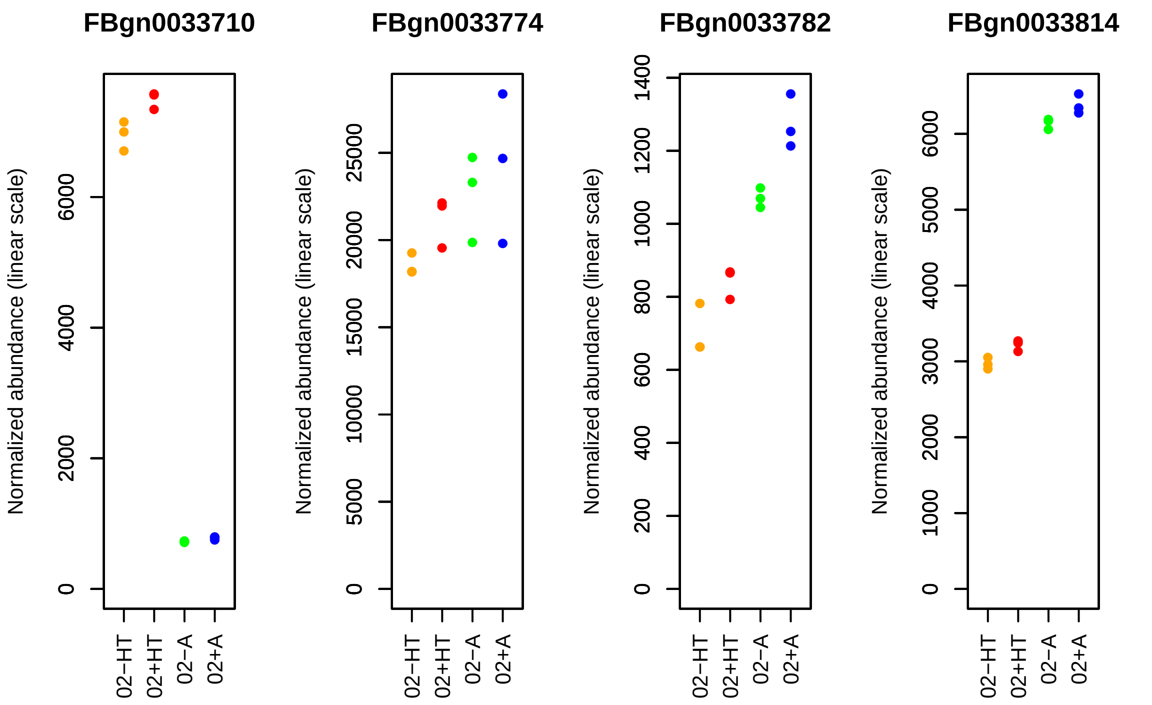

FBgn0033710
FBgn0033774
FBgn0033782
FBgn0033814
Normalized abundance (linear scale)
0
2000
4000
6000
5000
10000
15000
20000
25000
200
400
600
800
1000
1200
1400
1000
3000
5000
02−HT
02+HT
02−A
02+A

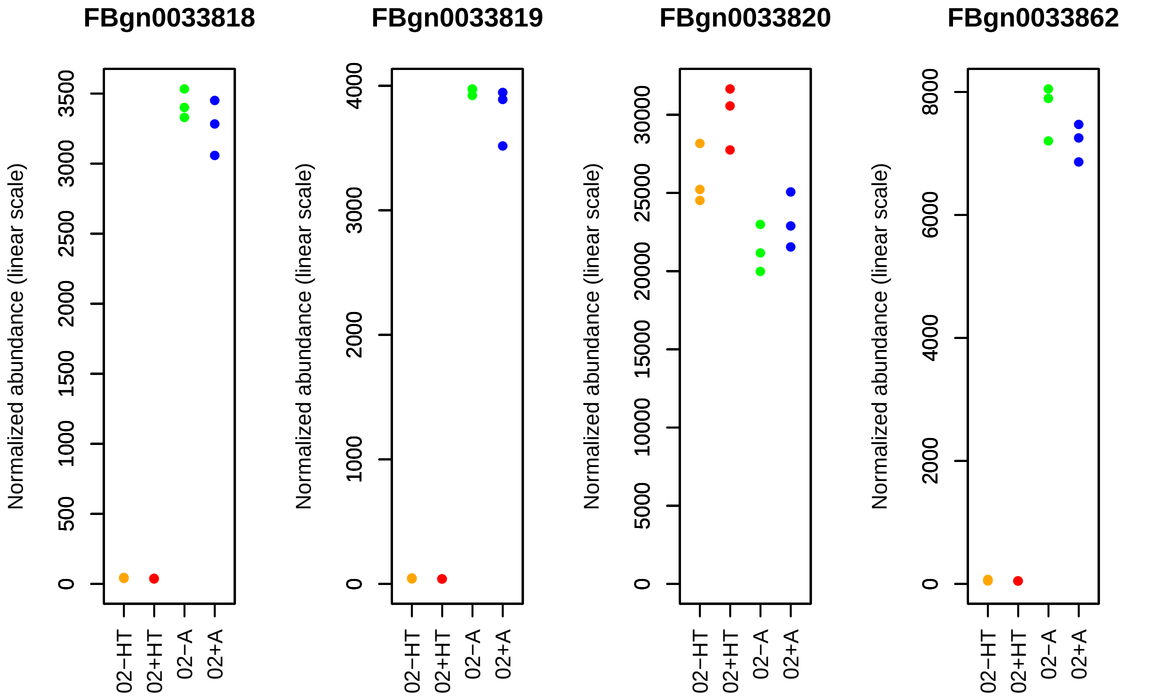
FBgn0033818
FBgn0033819
FBgn0033820
FBgn0033862
Normalized abundance (linear scale)
0
500
1000
1500
2000
2500
3000
3500
4000
6000
8000
5000
10000
15000
20000
25000
30000
02−HT
02+HT
02−A
02+A

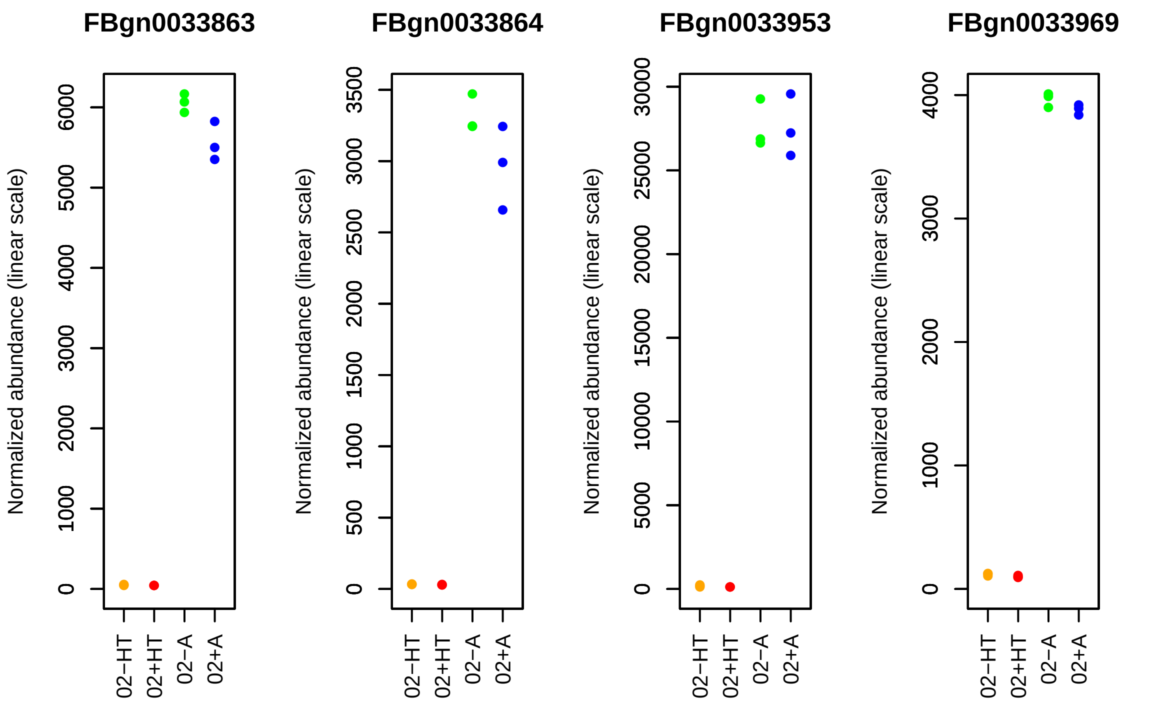
FBgn0033863
FBgn0033864
FBgn0033953
FBgn0033969
Normalized abundance (linear scale)
0
1000
2000
3000
4000
5000
6000
500
1500
2500
3500
5000
10000
15000
20000
25000
30000
02−HT
02+HT
02−A
02+A

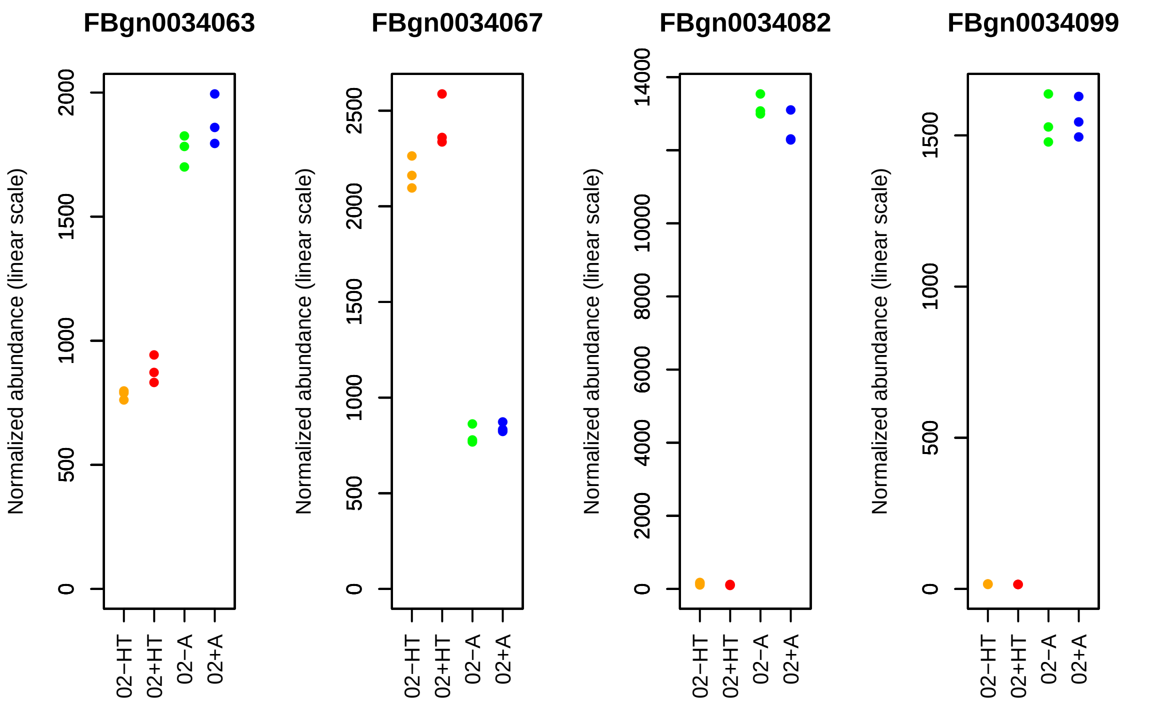

FBgn0034063
FBgn0034067
FBgn0034082
FBgn0034099
Normalized abundance (linear scale)
0
500
1000
1500
2000
2500
4000
6000
8000
10000
14000
02−HT
02+HT
02−A
02+A

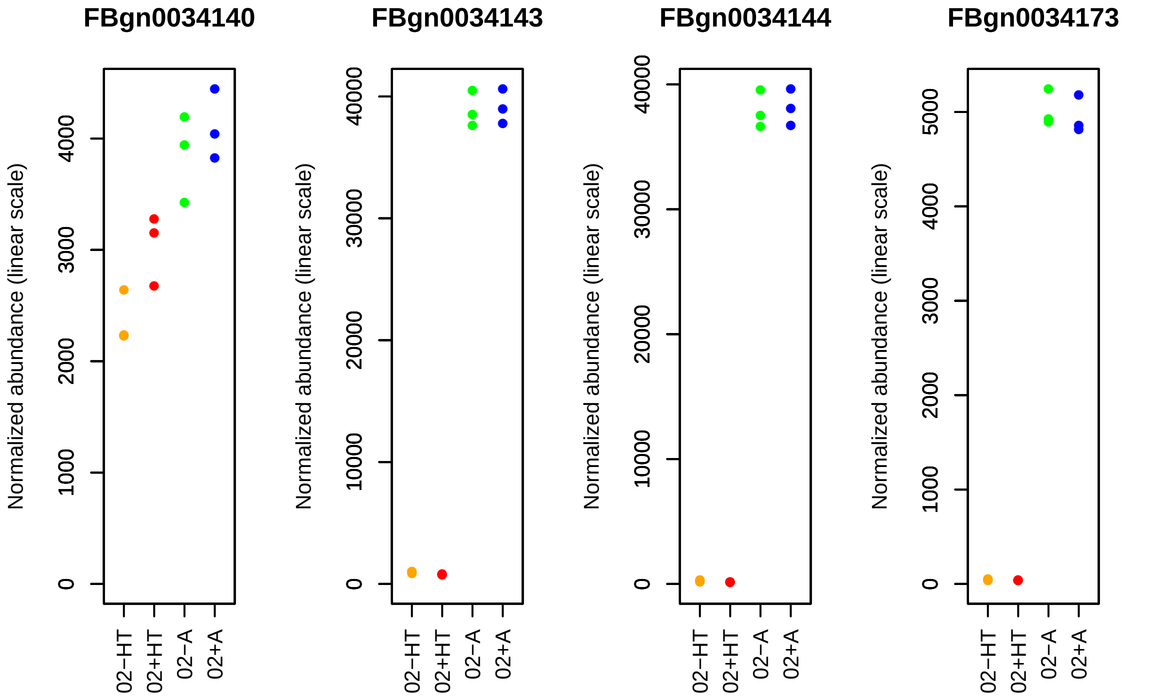

FBgn0034140
FBgn0034143
FBgn0034144
FBgn0034173
Normalized abundance (linear scale)
02−HT
02+HT
02−A
02+A

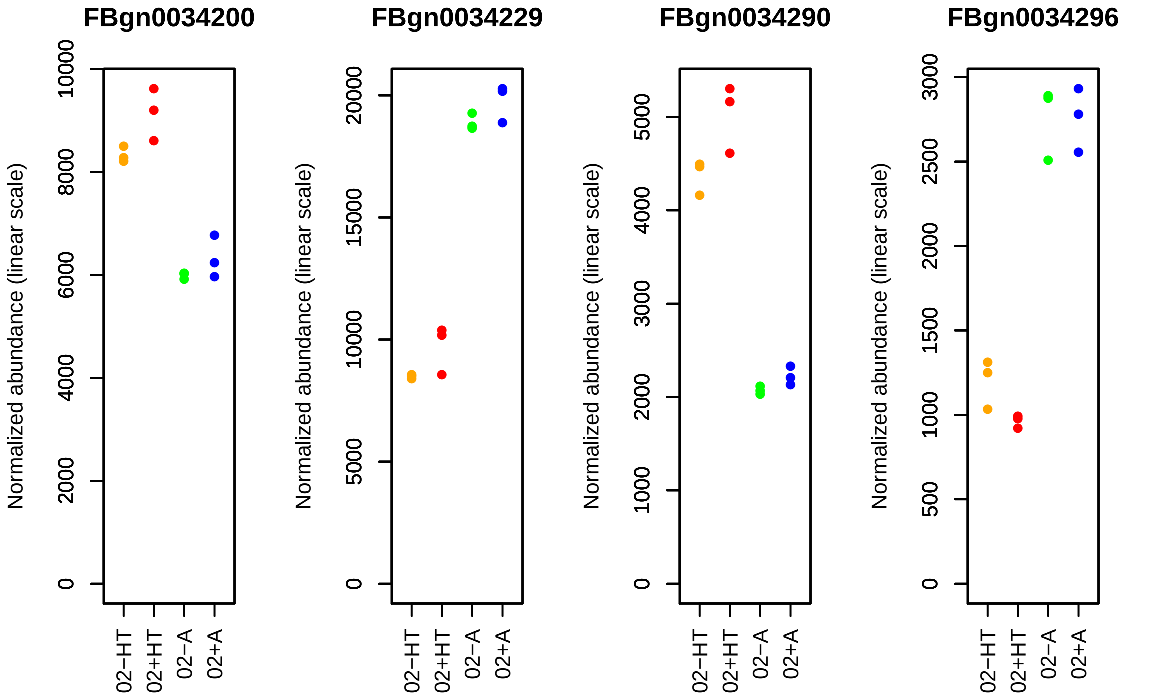

FBgn0034200
FBgn0034229
FBgn0034290
FBgn0034296
Normalized abundance (linear scale)
0
2000
4000
6000
8000
10000
5000
10000
15000
20000
1000
2000
3000
4000
5000
500
1000
1500
2000
2500
3000
02−HT
02+HT
02−A
02+A

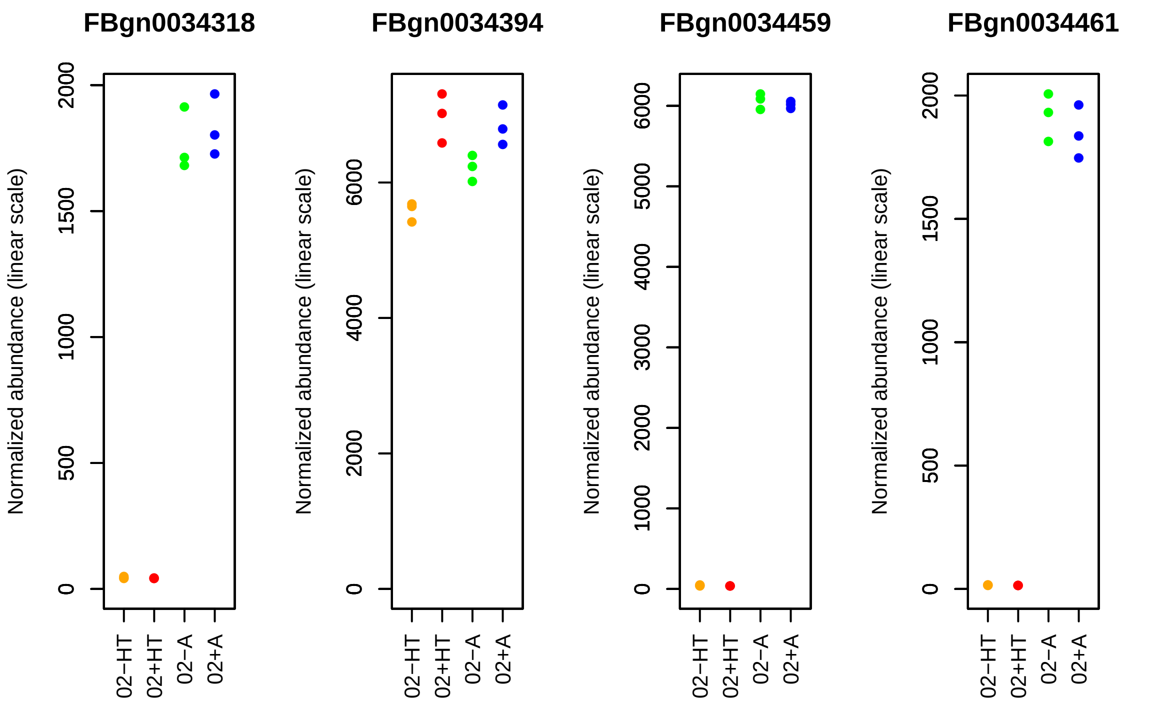

FBgn0034318
FBgn0034394
FBgn0034459
FBgn0034461
Normalized abundance (linear scale)
0
500
1000
1500
2000
2000
4000
6000
3000
5000
02−HT
02+HT
02−A
02+A

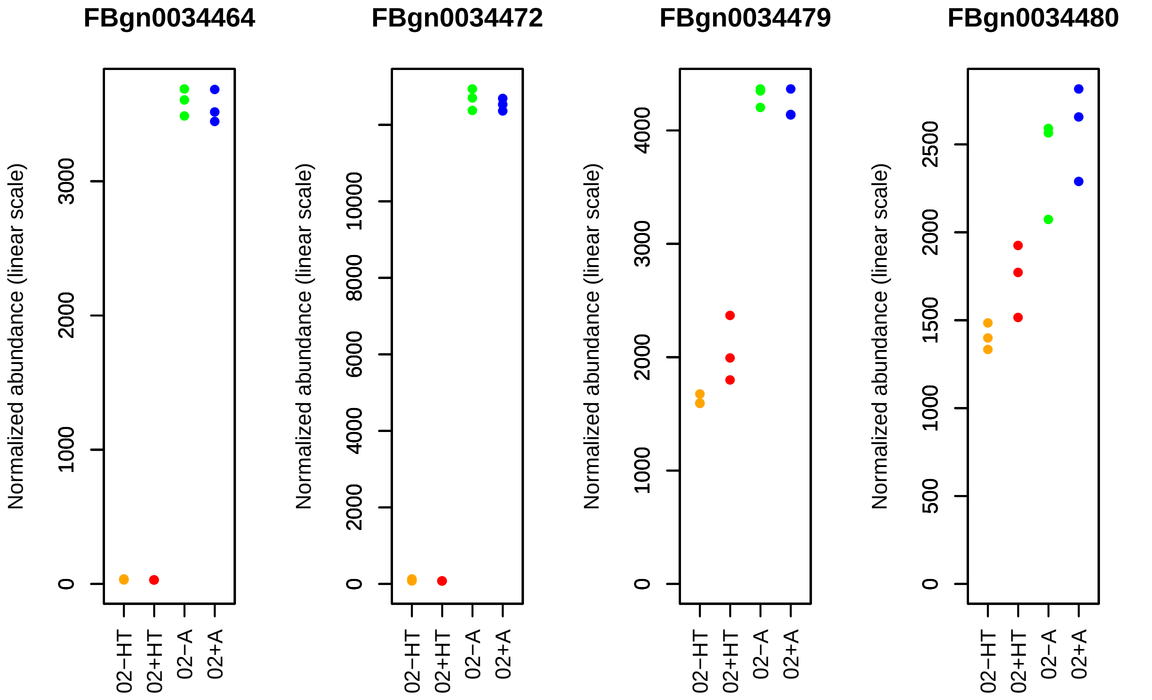
FBgn0034464
FBgn0034472
FBgn0034479
FBgn0034480
Normalized abundance (linear scale)
0
1000
2000
3000
2000
4000
6000
8000
10000
500
1500
2500
02−HT
02+HT
02−A
02+A

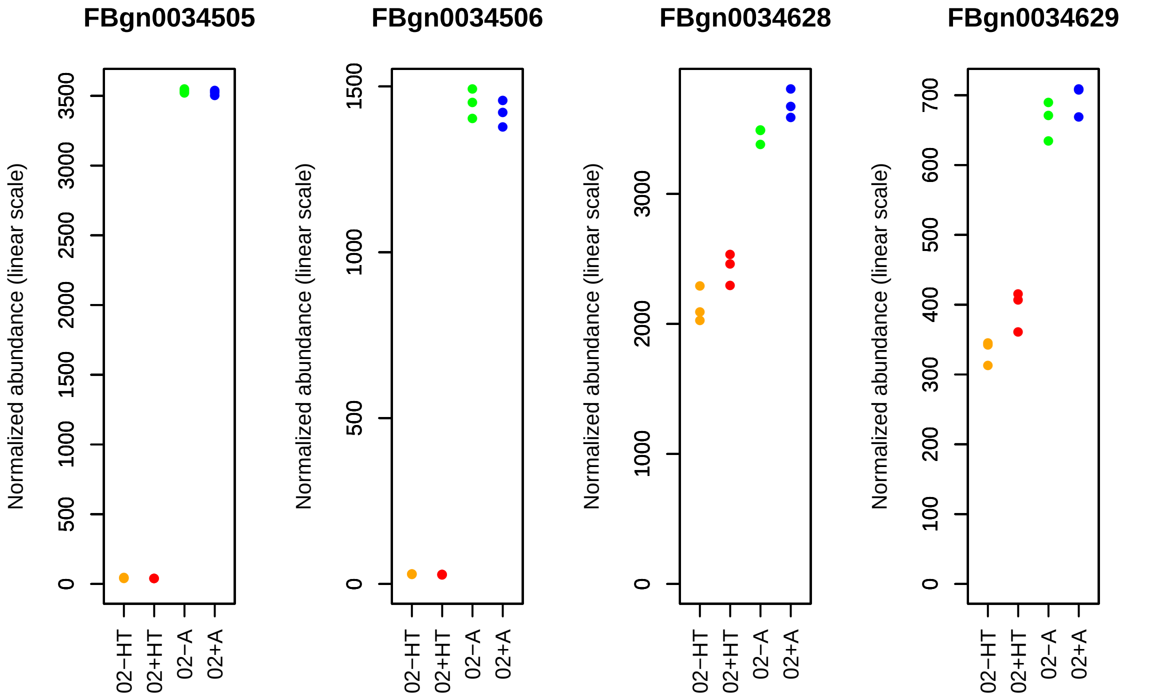

FBgn0034505
FBgn0034506
FBgn0034628
FBgn0034629
Normalized abundance (linear scale)
0
500
1000
1500
2000
2500
3000
3500
700
600
400
300
200
100
02−HT
02+HT
02−A
02+A

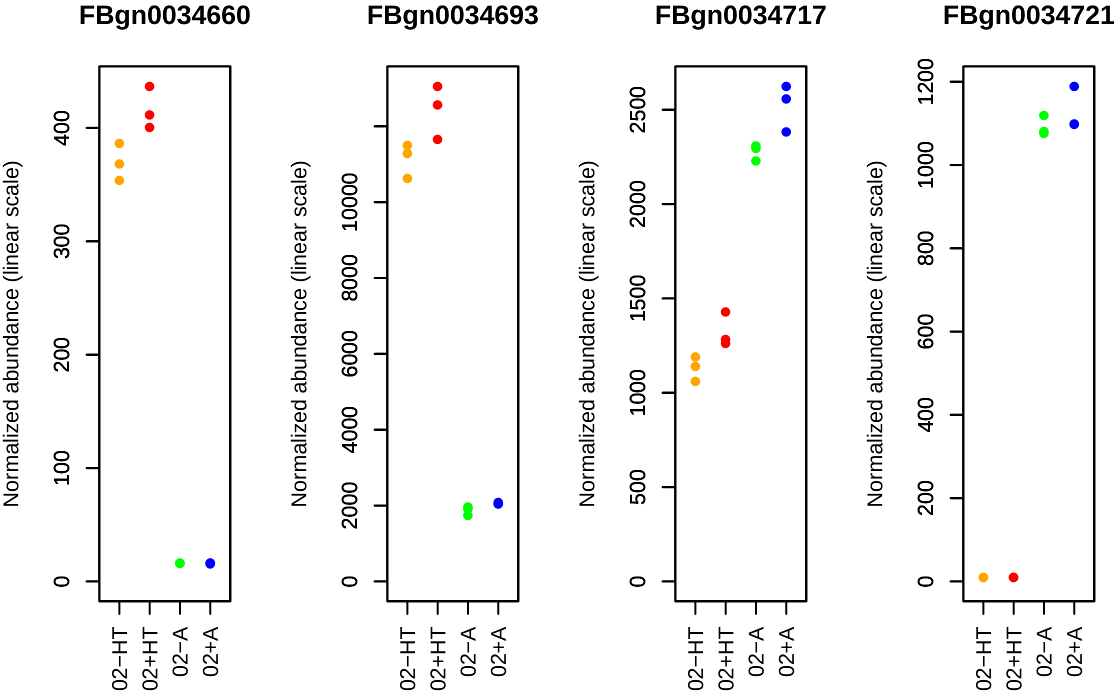
FBgn0034660
FBgn0034693
FBgn0034717
FBgn0034721
Normalized abundance (linear scale)
0
100
200
300
400
2000
4000
6000
8000
10000
500
1000
1500
2000
2500
200
400
600
800
1000
1200
02−HT
02+HT
02−A
02+A

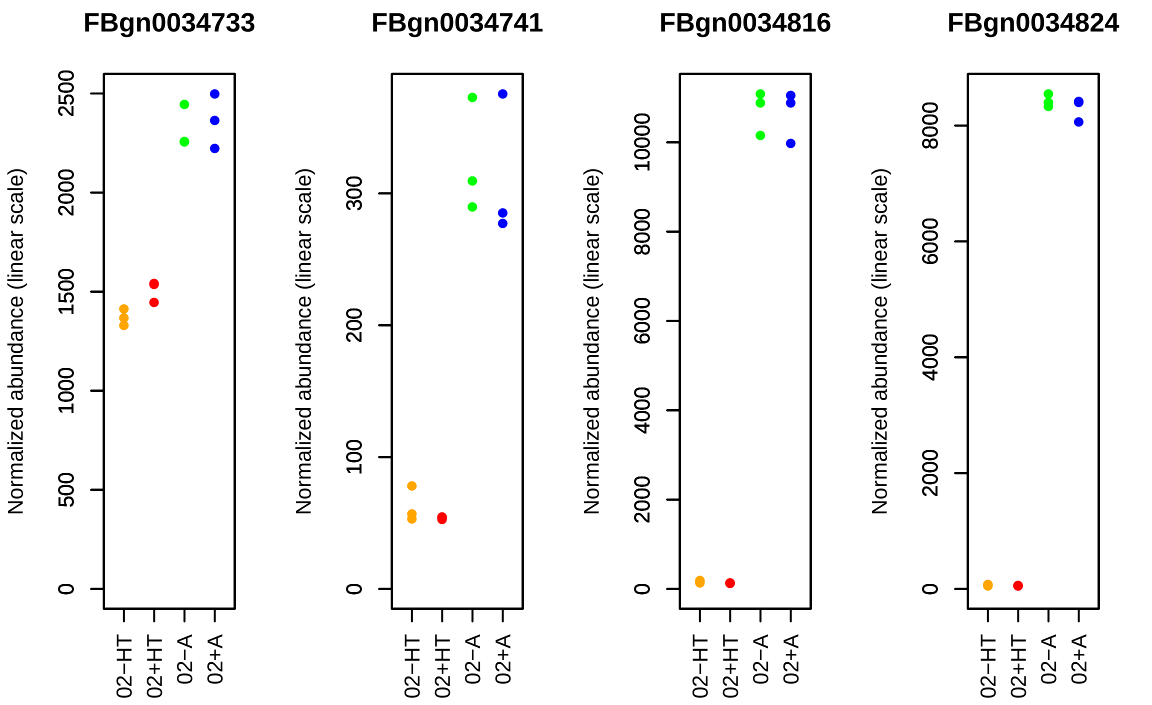

FBgn0034733
FBgn0034741
FBgn0034816
FBgn0034824
Normalized abundance (linear scale)
0
500
1000
1500
2000
2500
100
200
300
2000
4000
6000
8000
10000
02−HT
02+HT
02−A
02+A

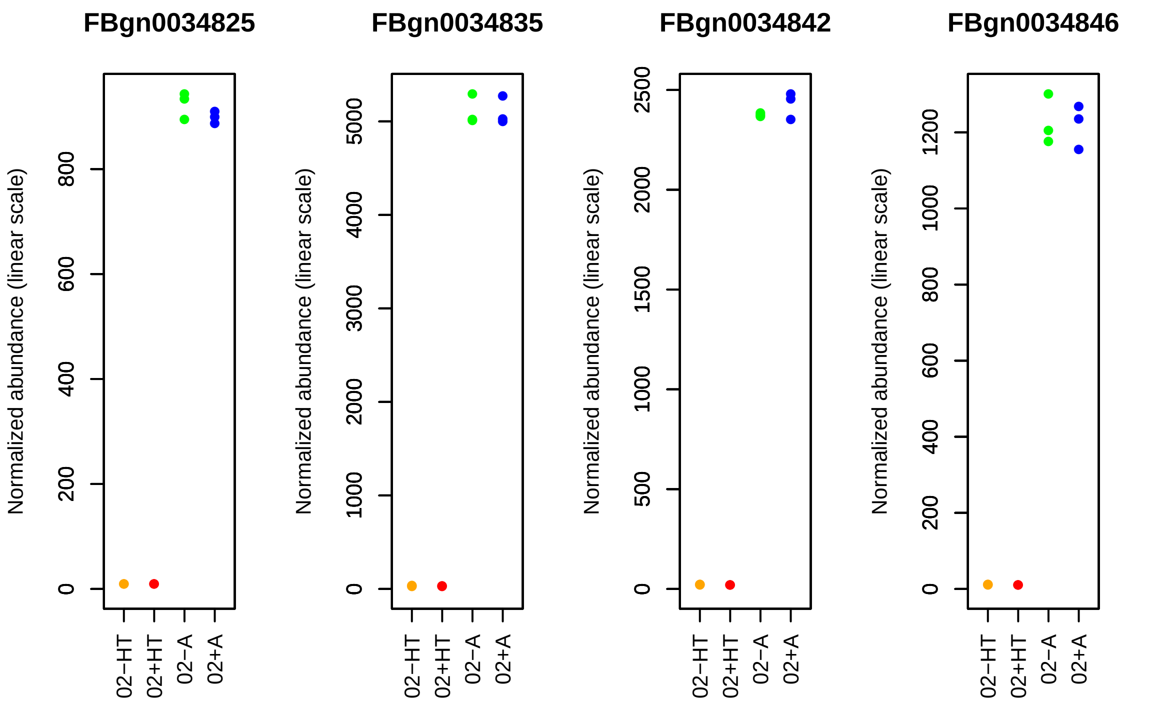
FBgn0034825
FBgn0034835
FBgn0034842
FBgn0034846
Normalized abundance (linear scale)
0
200
400
600
800
0
1000
2000
3000
4000
5000
0
500
1000
1500
2000
2500
0
200
400
600
800
1000
1200
02−HT
02+HT
02−A
02+A

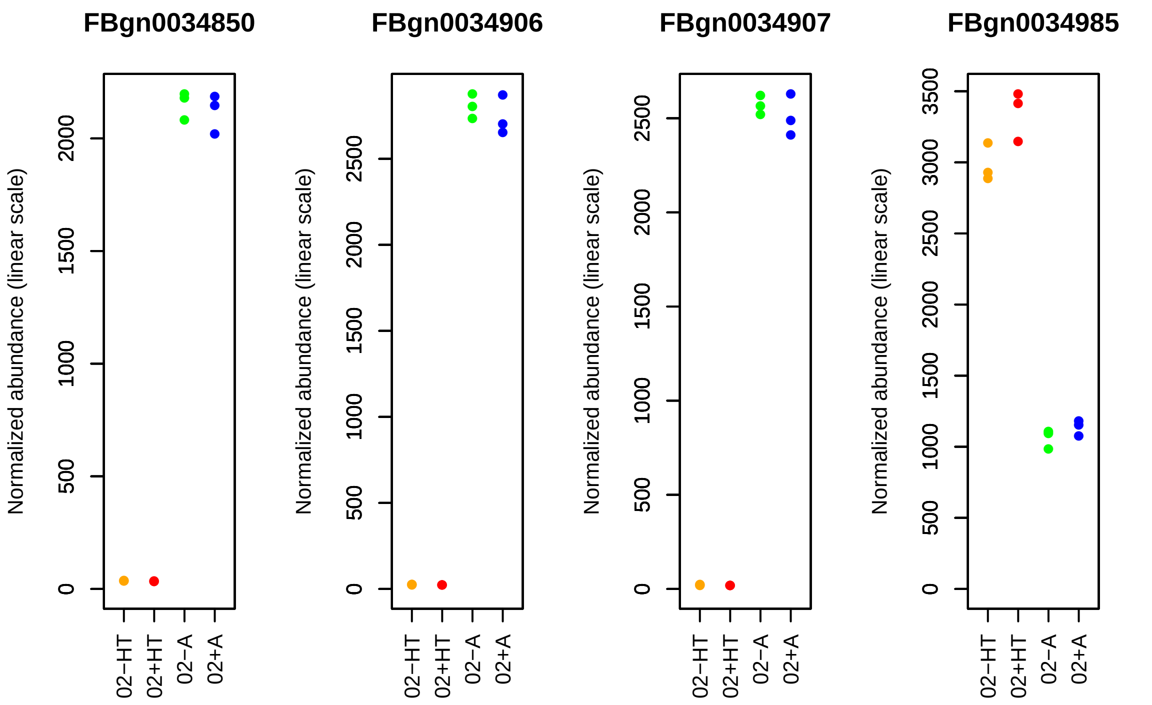

FBgn0034850
FBgn0034906
FBgn0034907
FBgn0034985
Normalized abundance (linear scale)
02−HT
02+HT
02−A
02+A

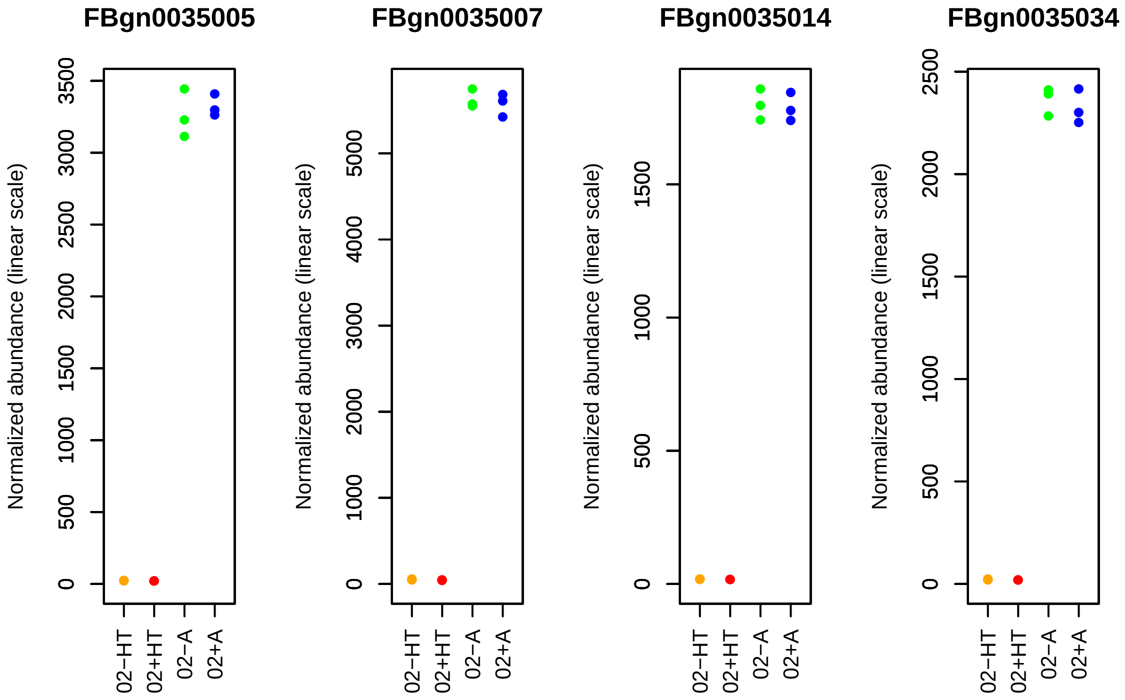
FBgn0035005
FBgn0035007
FBgn0035014
FBgn0035034
Normalized abundance (linear scale)
0
500
1000
1500
2000
2500
3000
3500
4000
5000
02−HT
02+HT
02−A
02+A

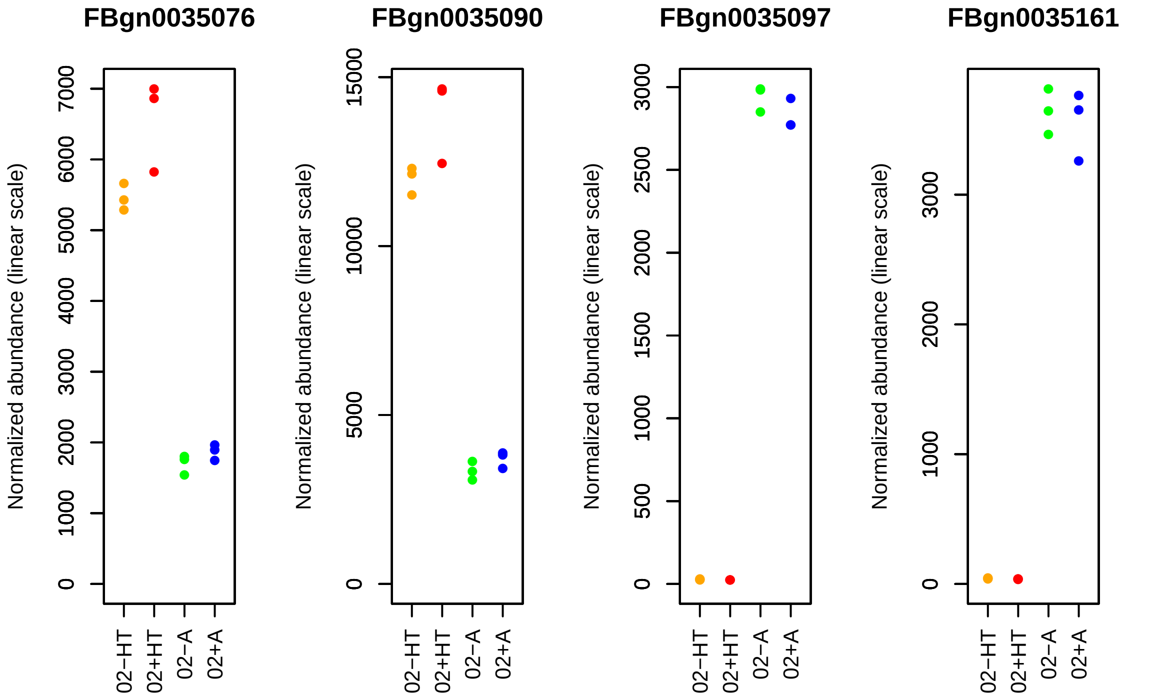
FBgn0035076
FBgn0035090
FBgn0035097
FBgn0035161
Normalized abundance (linear scale)
0
1000
2000
3000
4000
5000
6000
7000
5000
10000
15000
500
1500
2500
02−HT
02+HT
02−A
02+A

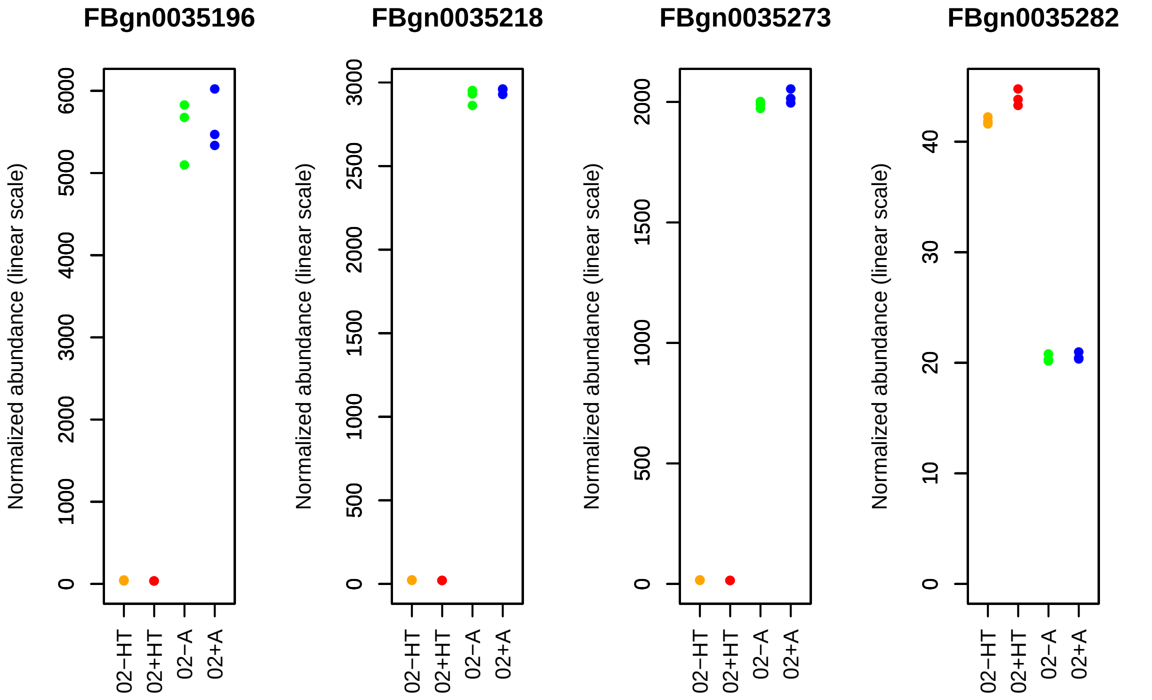
FBgn0035196
FBgn0035218
FBgn0035273
FBgn0035282
Normalized abundance (linear scale)
0
1000
2000
3000
4000
5000
6000
500
1500
2500
10
20
30
40
02−HT
02+HT
02−A
02+A

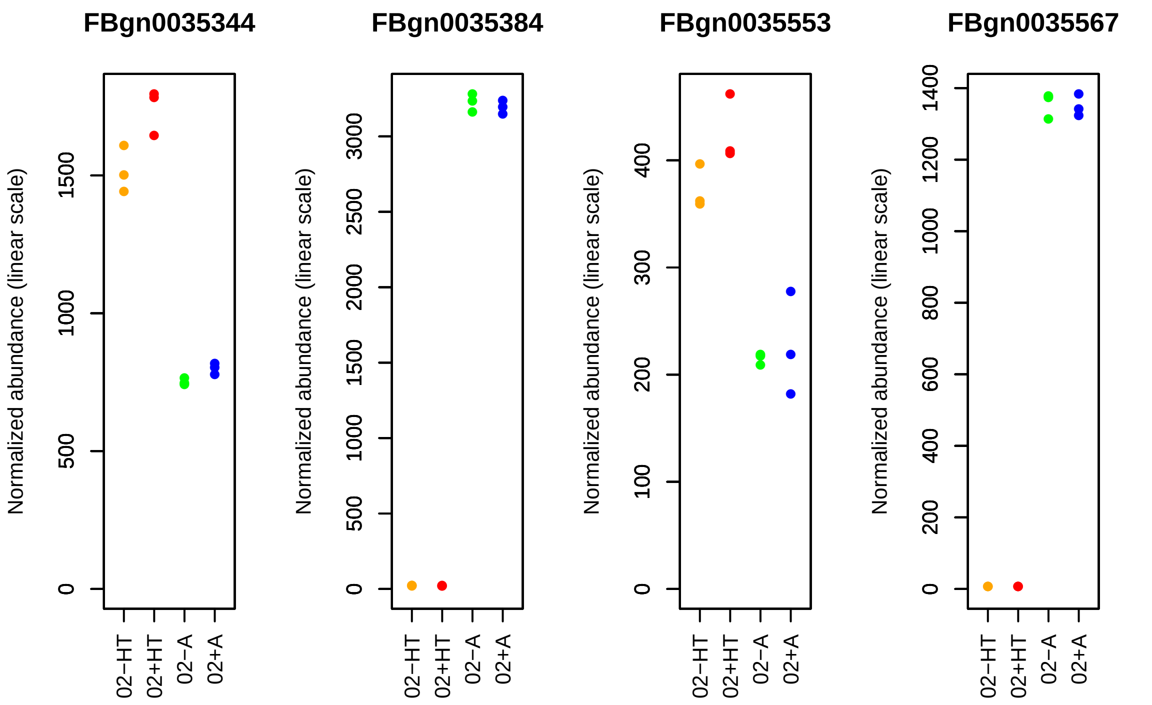

FBgn0035344
FBgn0035384
FBgn0035553
FBgn0035567
Normalized abundance (linear scale)
0
500
1000
1500
2000
2500
3000
100
200
300
400
600
800
1200
1400
02−HT
02+HT
02−A
02+A

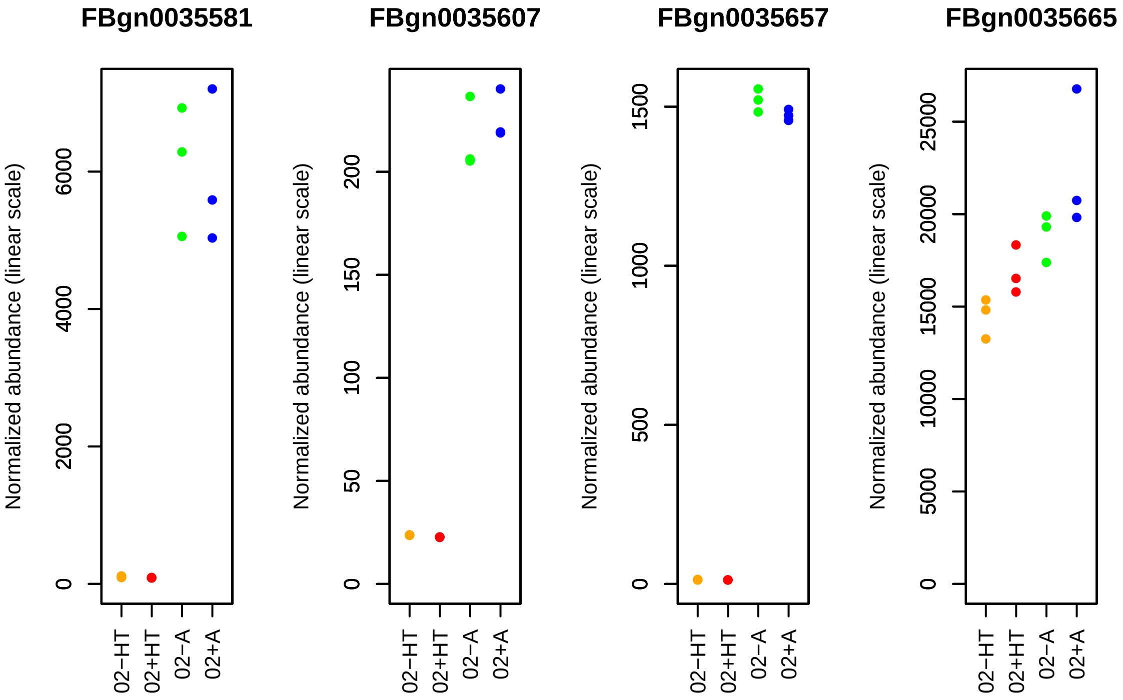

FBgn0035581
FBgn0035607
FBgn0035657
FBgn0035665
Normalized abundance (linear scale)
0
2000
4000
6000
50
100
150
200
500
1000
1500
5000
10000
15000
20000
25000
02−HT
02+HT
02−A
02+A

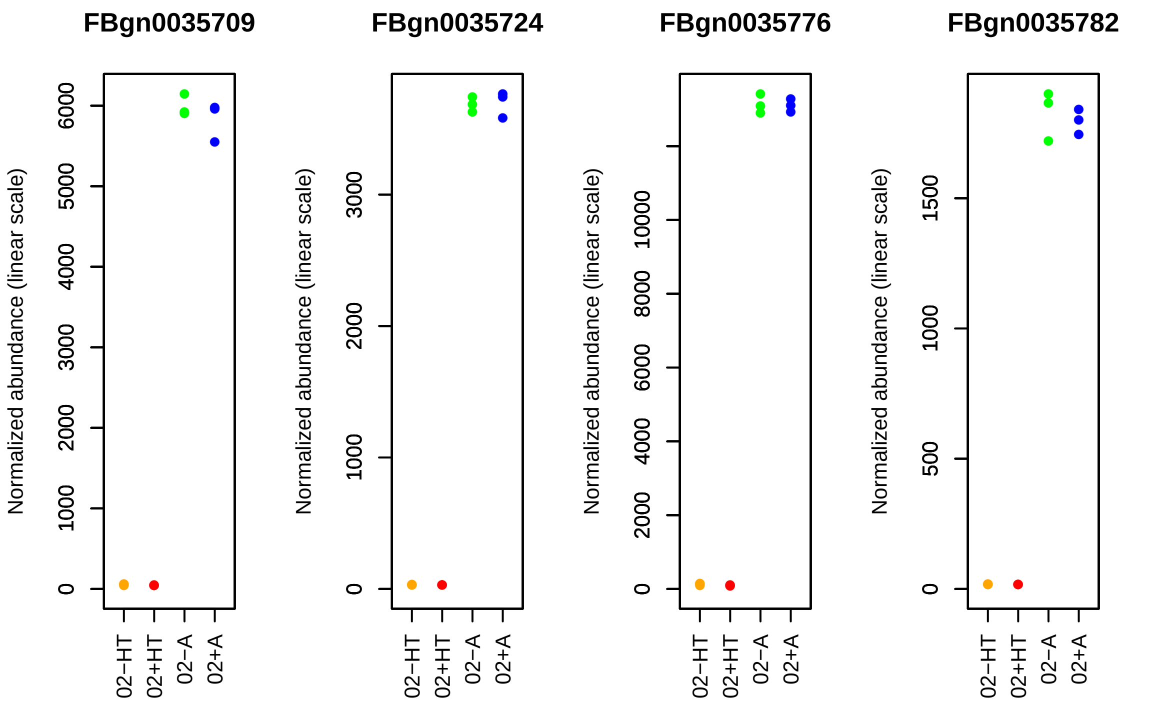

FBgn0035709
FBgn0035724
FBgn0035776
FBgn0035782
Normalized abundance (linear scale)
0
1000
2000
3000
4000
5000
6000
500
1500
8000
10000
02−HT
02+HT
02−A
02+A

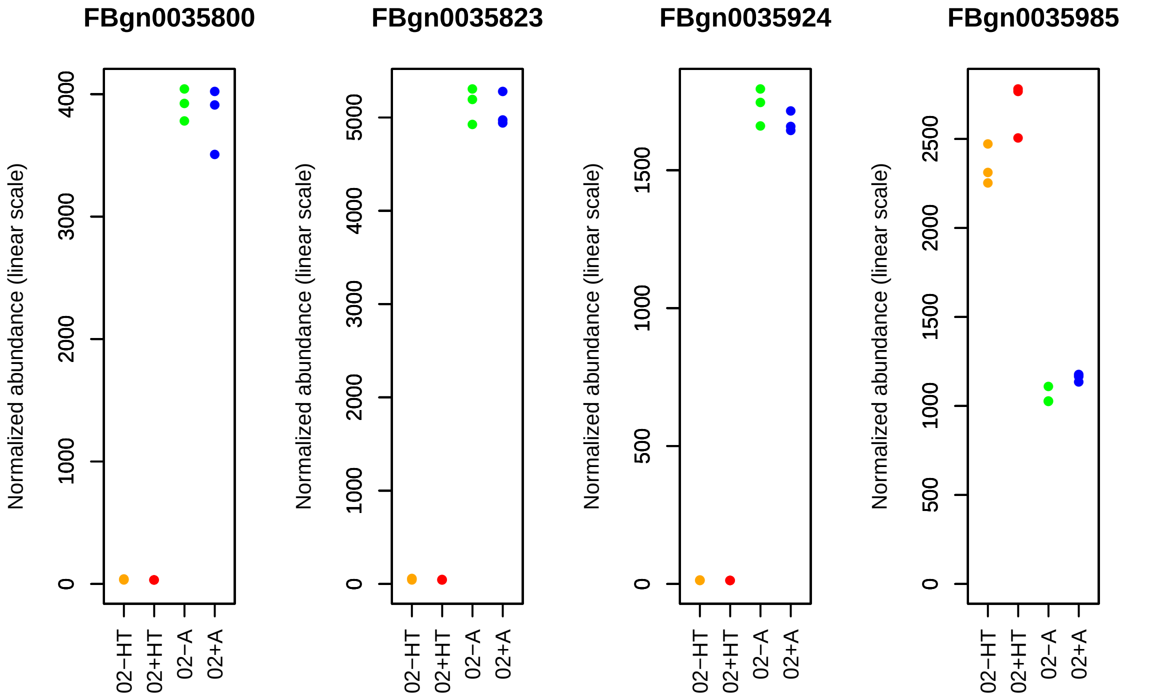

FBgn0035800
FBgn0035823
FBgn0035924
FBgn0035985
Normalized abundance (linear scale)
0
1000
2000
3000
4000
5000
500
1500
2500
02−HT
02+HT
02−A
02+A

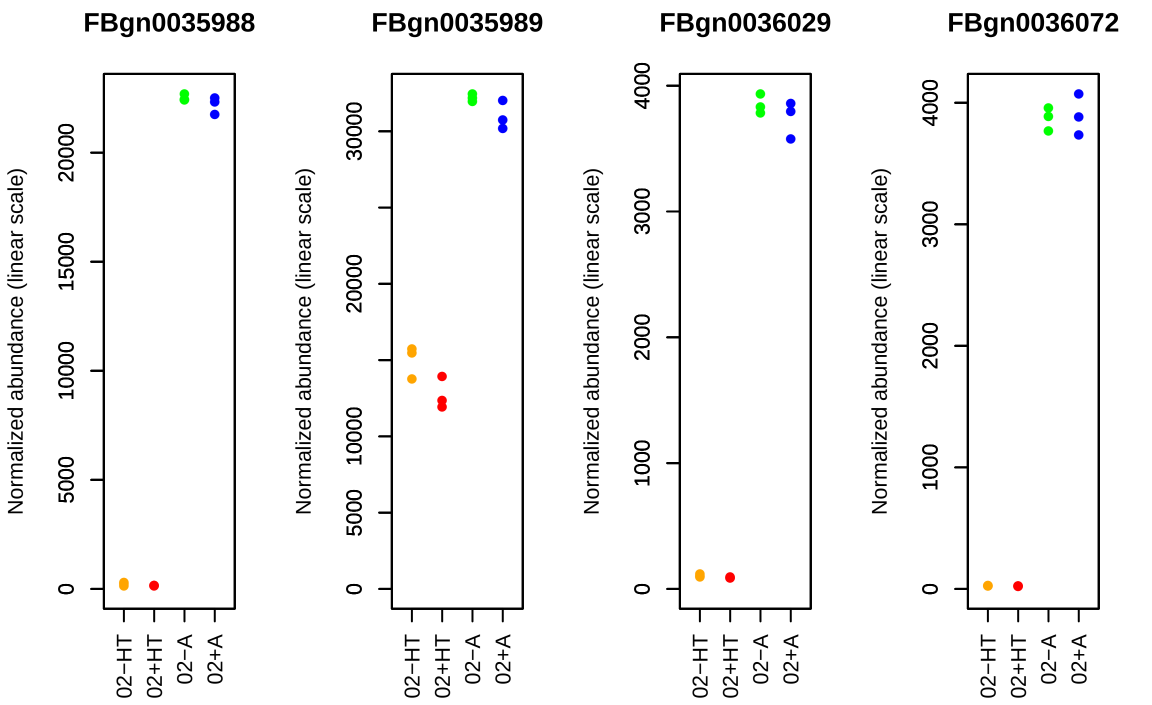

FBgn0035988
FBgn0035989
FBgn0036029
FBgn0036072
Normalized abundance (linear scale)
0
5000
10000
15000
20000
25000
30000
1000
2000
3000
4000
02−HT
02+HT
02−A
02+A

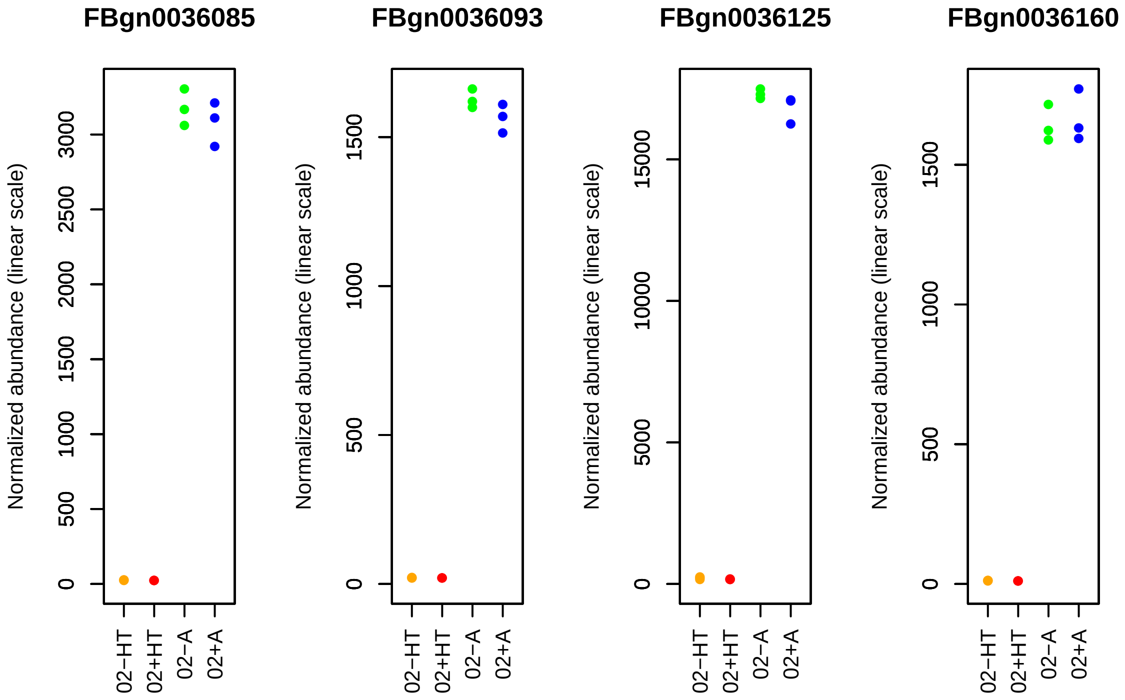
FBgn0036085
FBgn0036093
FBgn0036125
FBgn0036160
Normalized abundance (linear scale)
0
500
1000
1500
2000
2500
3000
5000
10000
15000
02−HT
02+HT
02−A
02+A

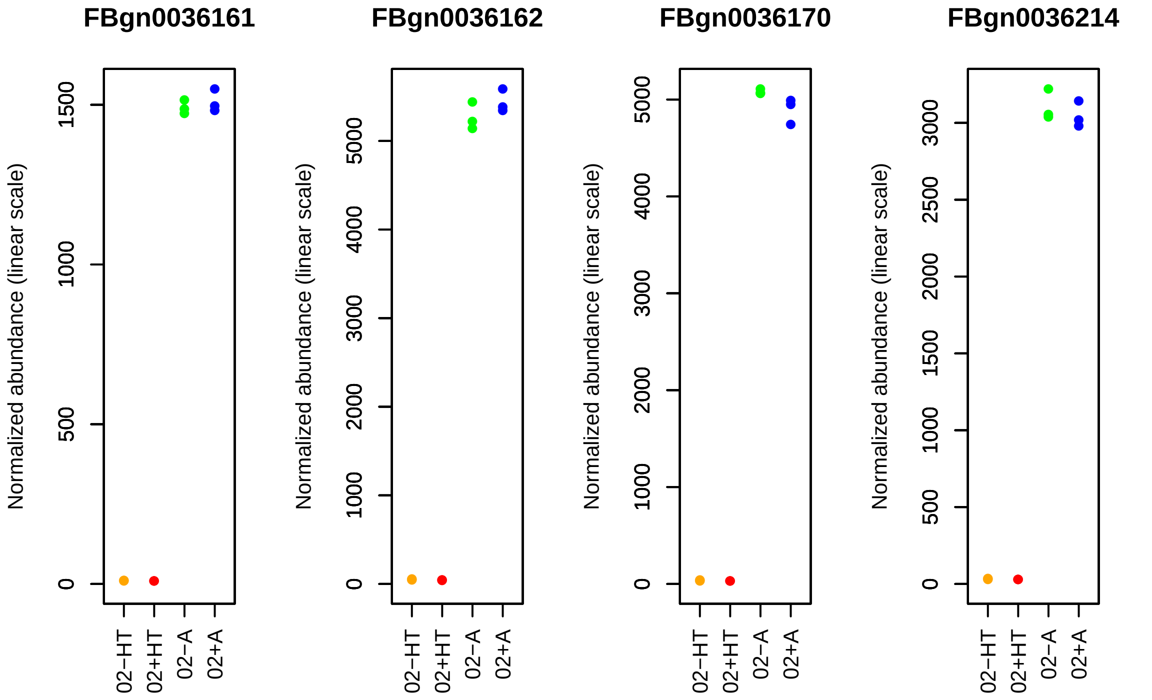
FBgn0036161
FBgn0036162
FBgn0036170
FBgn0036214
Normalized abundance (linear scale)
0
500
1000
1500
1000
2000
3000
4000
5000
2500
02−HT
02+HT
02−A
02+A

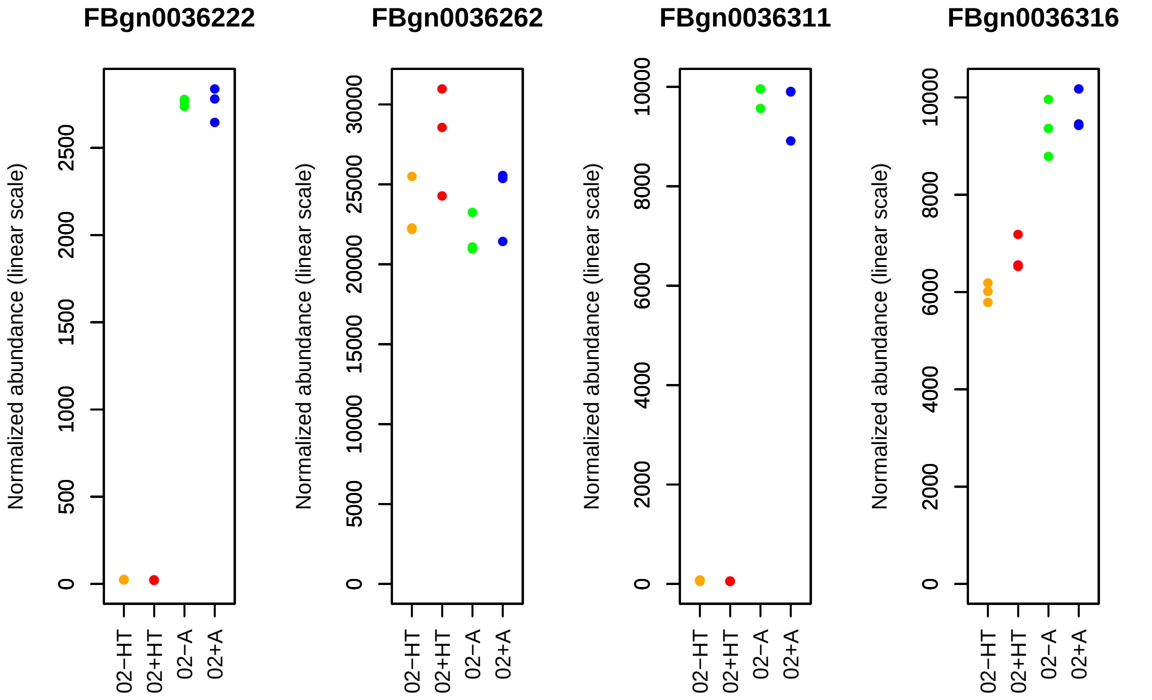

FBgn0036222
FBgn0036262
FBgn0036311
FBgn0036316
Normalized abundance (linear scale)
0
500
1000
1500
2000
2500
5000
10000
15000
20000
25000
30000
2000
4000
6000
8000
10000
02−HT
02+HT
02−A
02+A

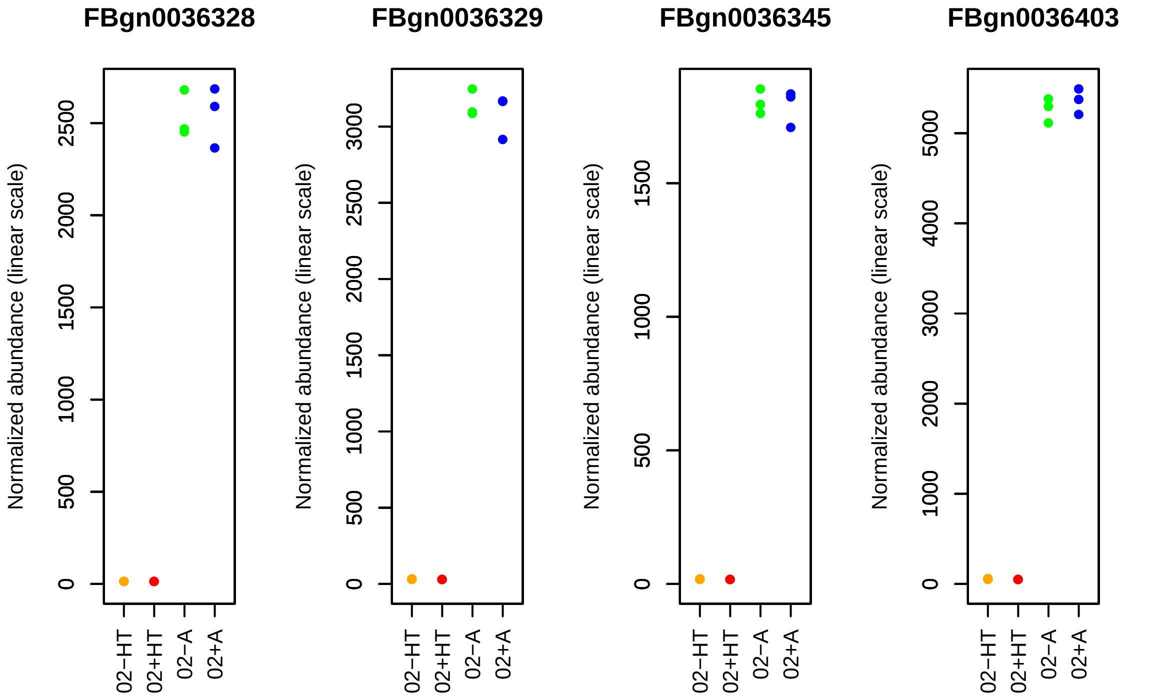

FBgn0036328
FBgn0036329
FBgn0036345
FBgn0036403
Normalized abundance (linear scale)
0
500
1000
1500
2000
2500
3000
4000
5000
02−HT
02+HT
02−A
02+A

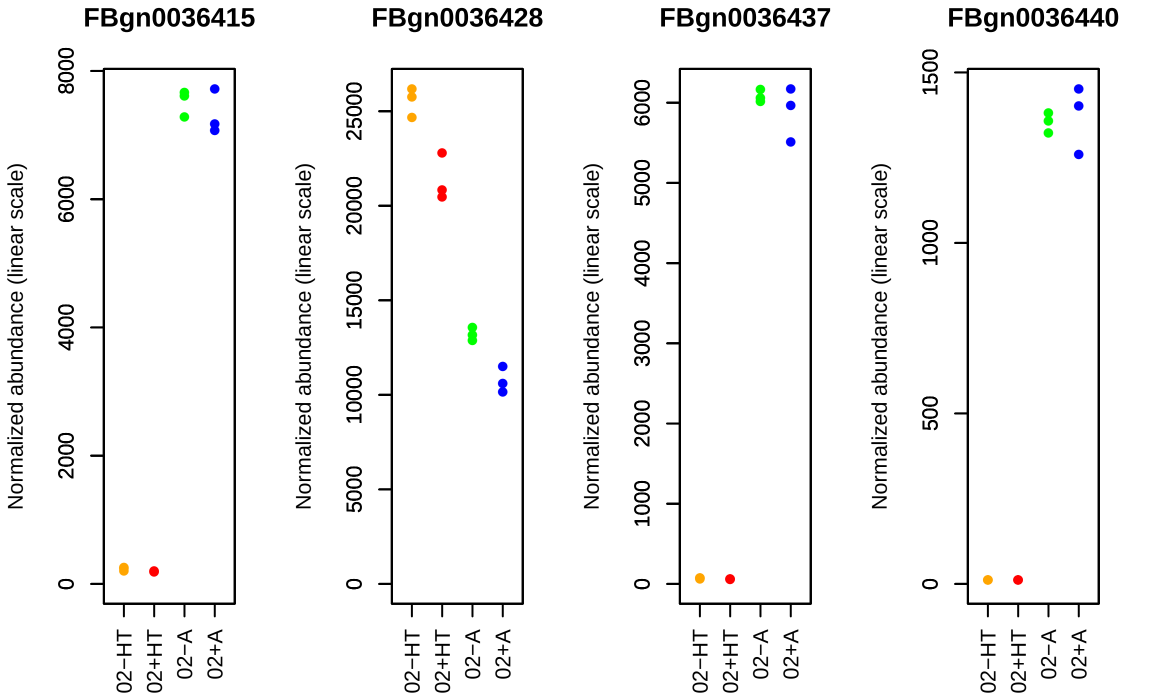
FBgn0036415
FBgn0036428
FBgn0036437
FBgn0036440
Normalized abundance (linear scale)
0
2000
4000
6000
8000
5000
10000
15000
20000
25000
1000
3000
500
1500
02−HT
02+HT
02−A
02+A

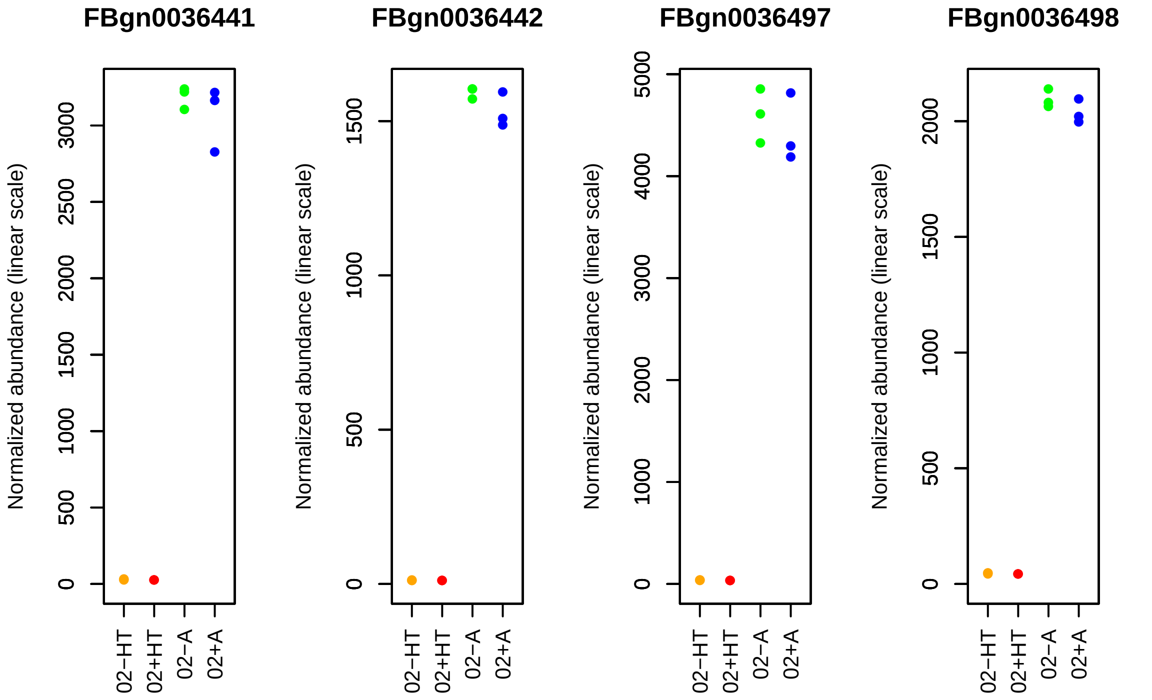

FBgn0036441
FBgn0036442
FBgn0036497
FBgn0036498
Normalized abundance (linear scale)
02−HT
02+HT
02−A
02+A

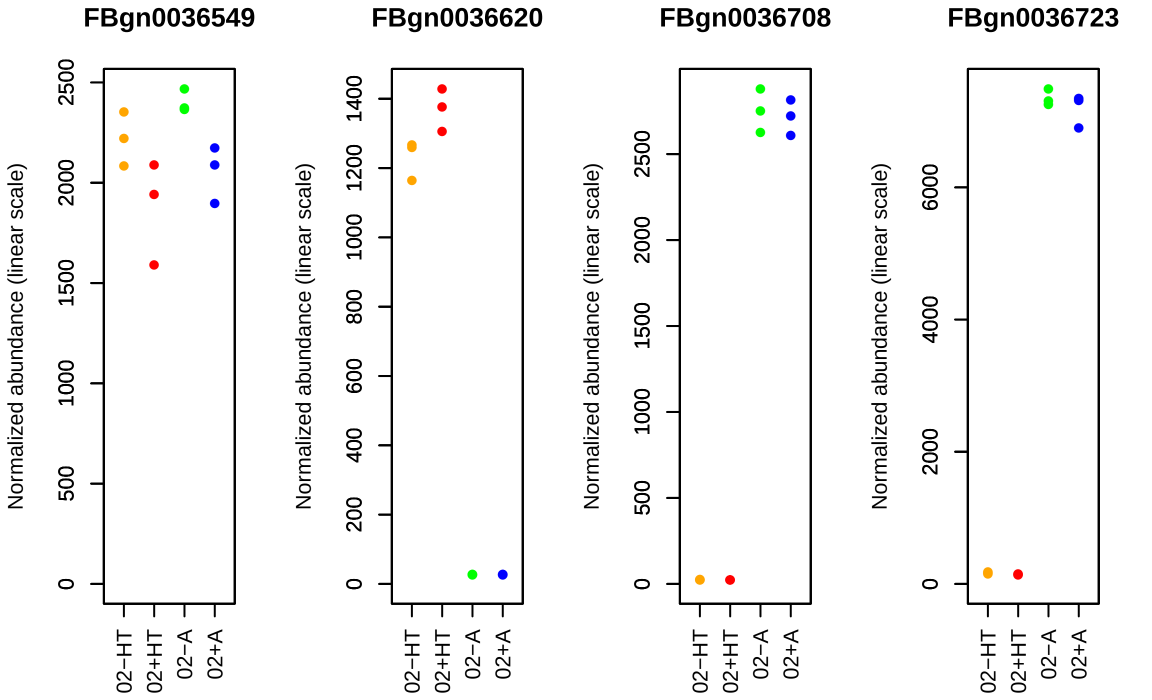

FBgn0036549
FBgn0036620
FBgn0036708
FBgn0036723
Normalized abundance (linear scale)
0
500
1000
1500
2000
2500
200
400
600
800
1000
1200
1400
2000
4000
6000
02−HT
02+HT
02−A
02+A

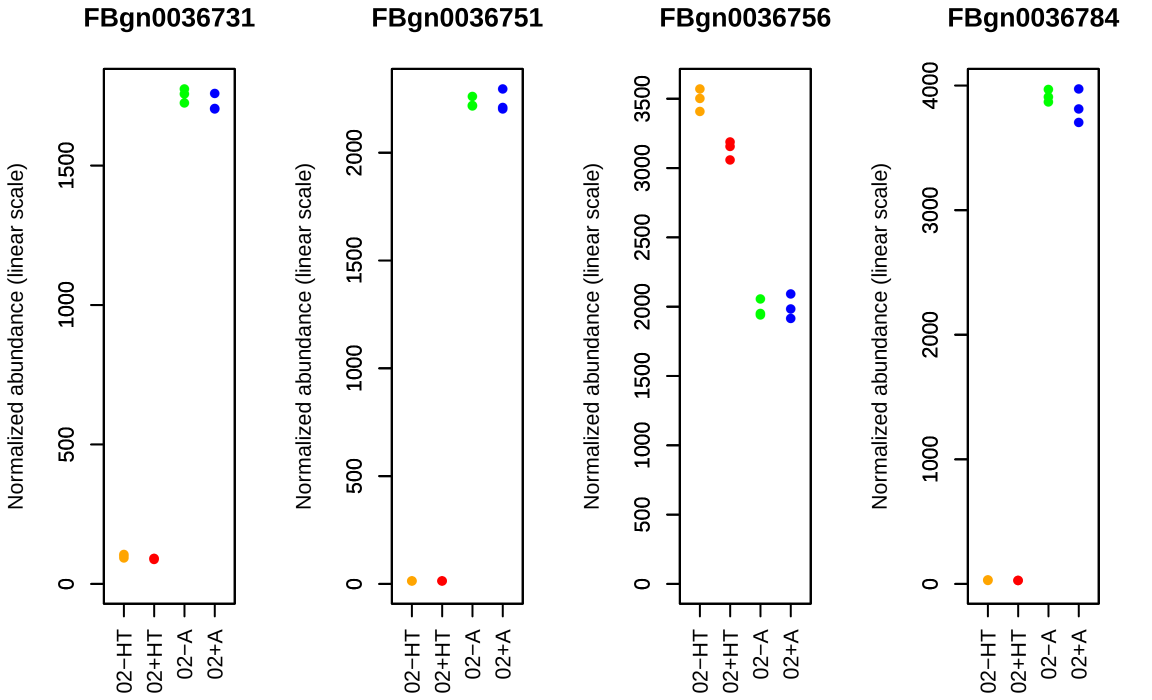

FBgn0036731
FBgn0036751
FBgn0036756
FBgn0036784
Normalized abundance (linear scale)
0
500
1000
1500
2000
2500
3000
3500
4000
02−HT
02+HT
02−A
02+A

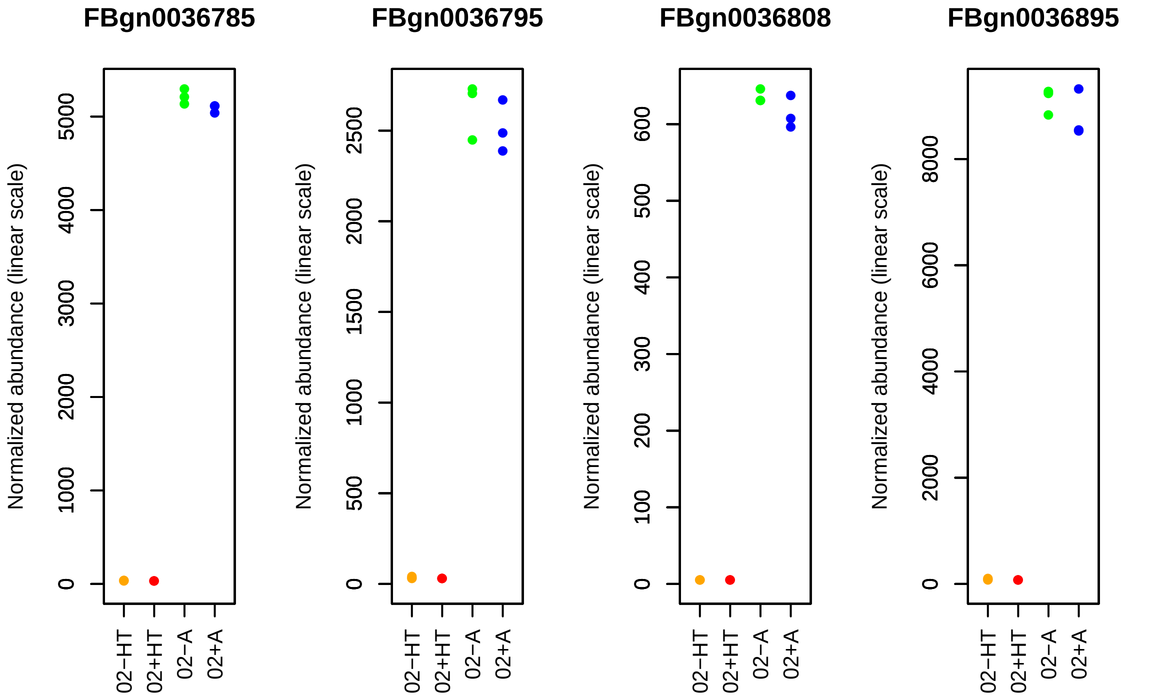

FBgn0036785
FBgn0036795
FBgn0036808
FBgn0036895
Normalized abundance (linear scale)
0
1000
2000
3000
4000
5000
500
1500
2500
100
200
300
400
600
6000
8000
02−HT
02+HT
02−A
02+A

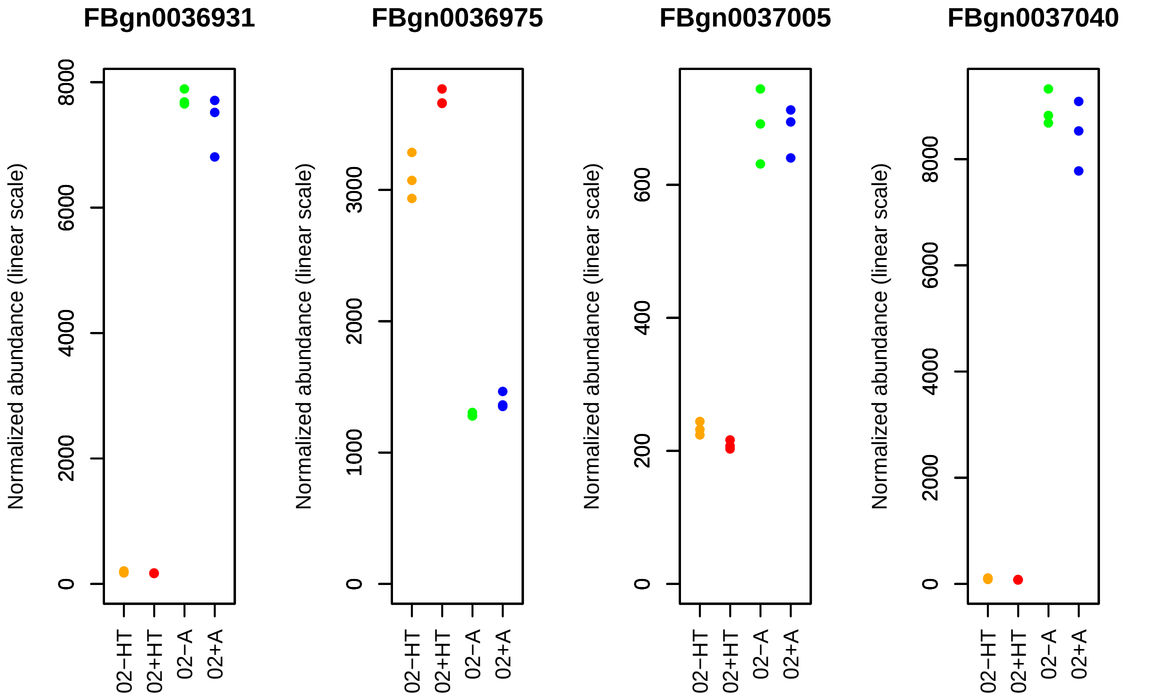
FBgn0036931
FBgn0036975
FBgn0037005
FBgn0037040
Normalized abundance (linear scale)
0
2000
4000
6000
8000
0
1000
2000
3000
0
200
400
600
02−HT
02+HT
02−A
02+A

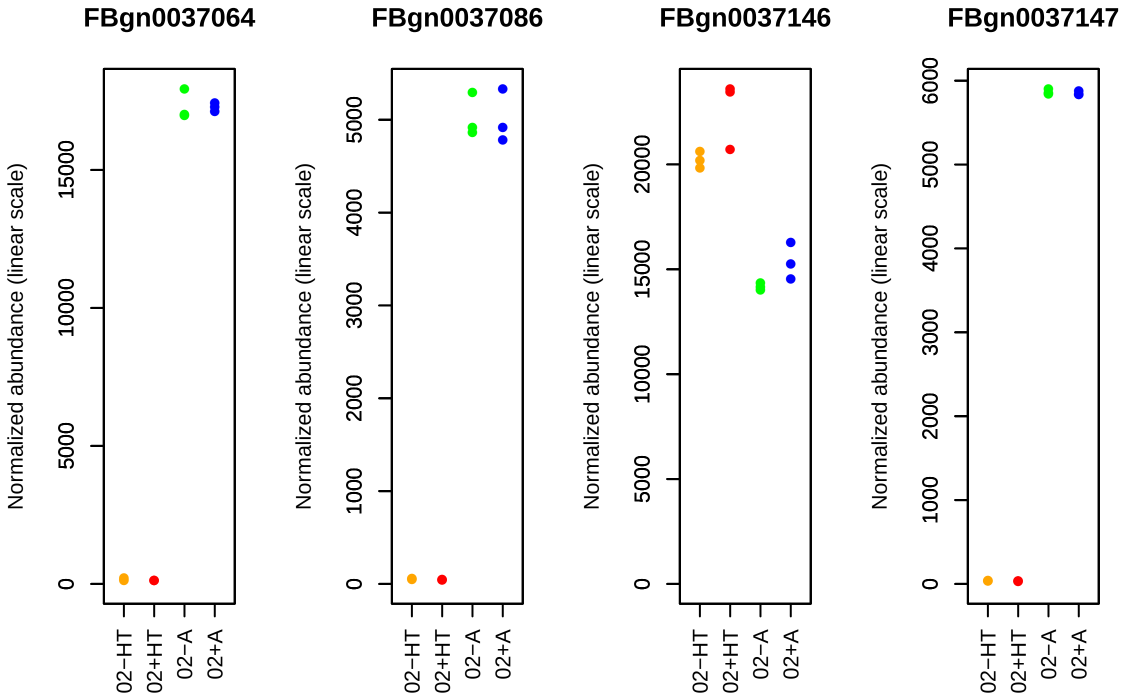

FBgn0037064
FBgn0037086
FBgn0037146
FBgn0037147
Normalized abundance (linear scale)
0
5000
10000
15000
1000
2000
3000
4000
5000
20000
6000
02−HT
02+HT
02−A
02+A

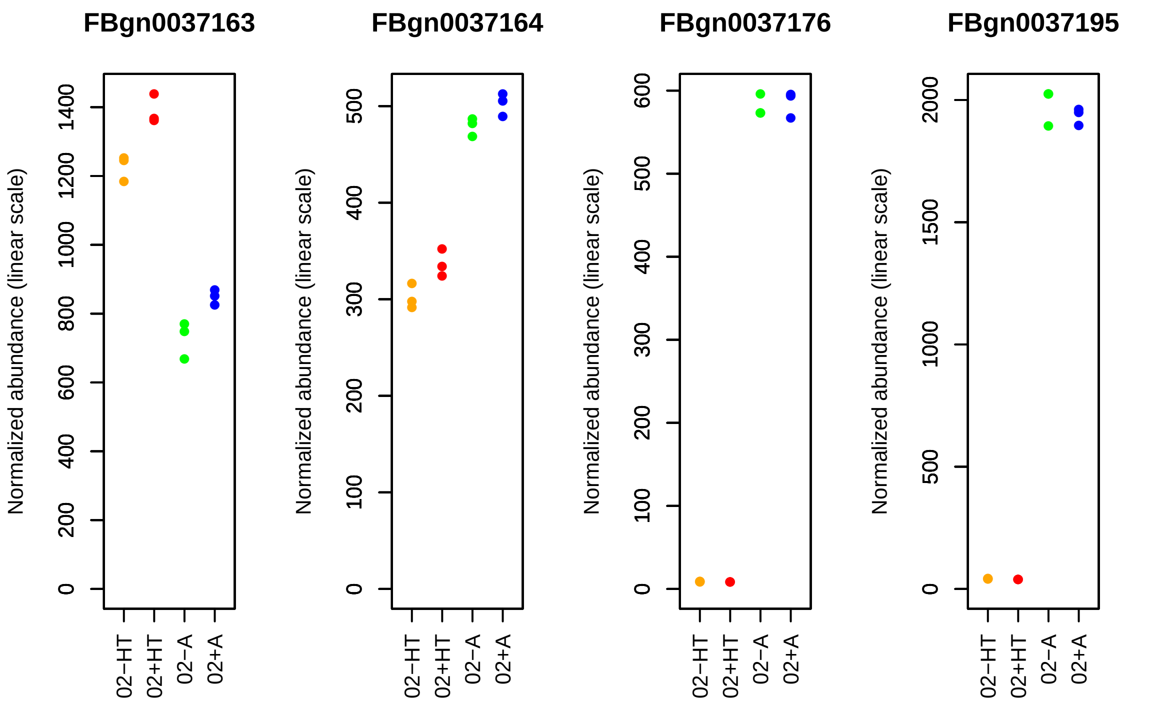

FBgn0037163
FBgn0037164
FBgn0037176
FBgn0037195
Normalized abundance (linear scale)
0
200
400
600
800
1000
1200
1400
100
300
500
1500
2000
02−HT
02+HT
02−A
02+A

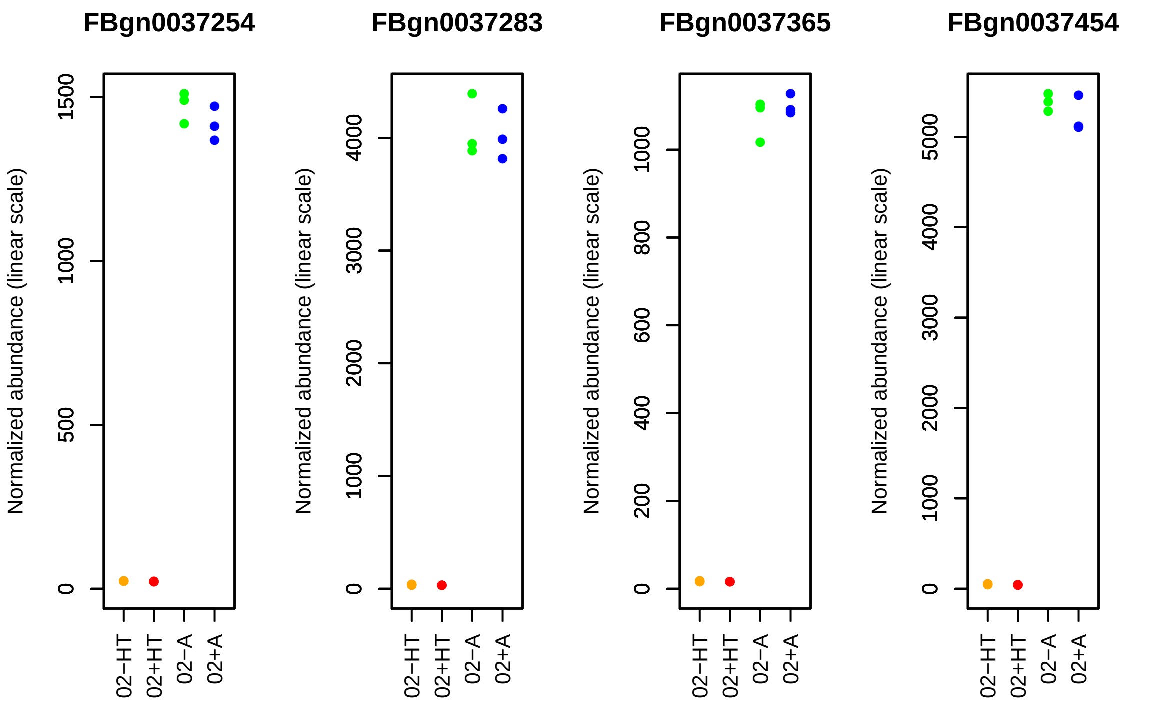
FBgn0037254
FBgn0037283
FBgn0037365
FBgn0037454
Normalized abundance (linear scale)
0
500
1000
1500
1000
2000
3000
4000
200
400
600
800
5000
02−HT
02+HT
02−A
02+A

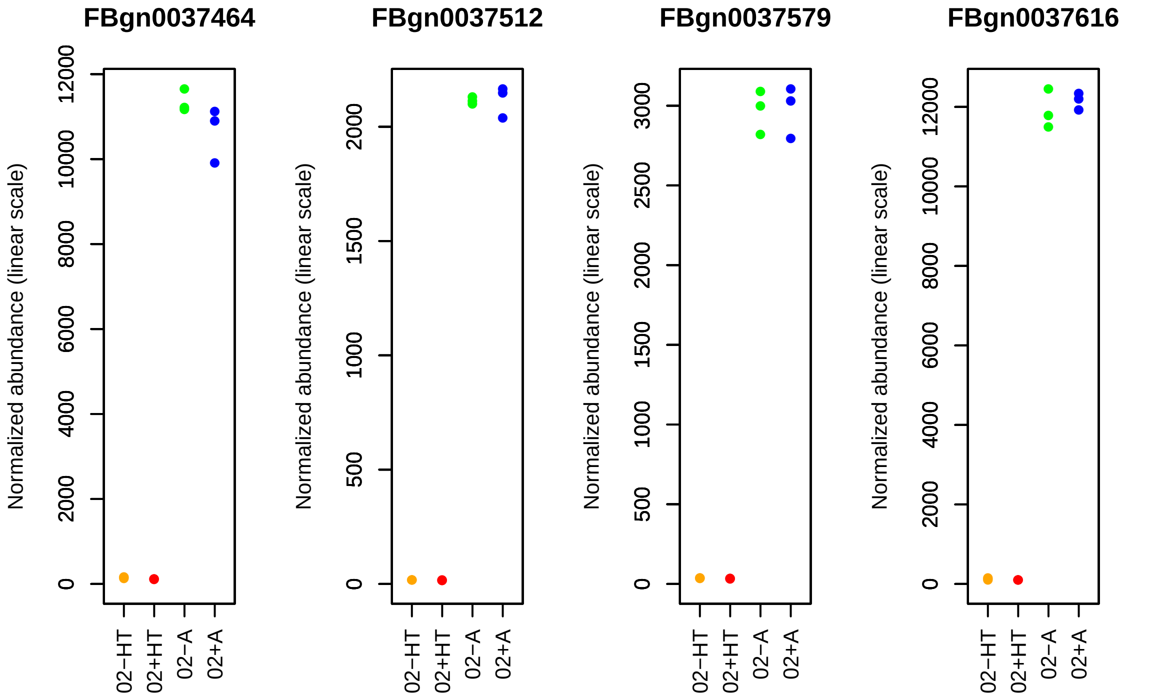
FBgn0037464
FBgn0037512
FBgn0037579
FBgn0037616
Normalized abundance (linear scale)
0
2000
4000
6000
8000
10000
12000
500
1000
1500
2000
2500
3000
02−HT
02+HT
02−A
02+A

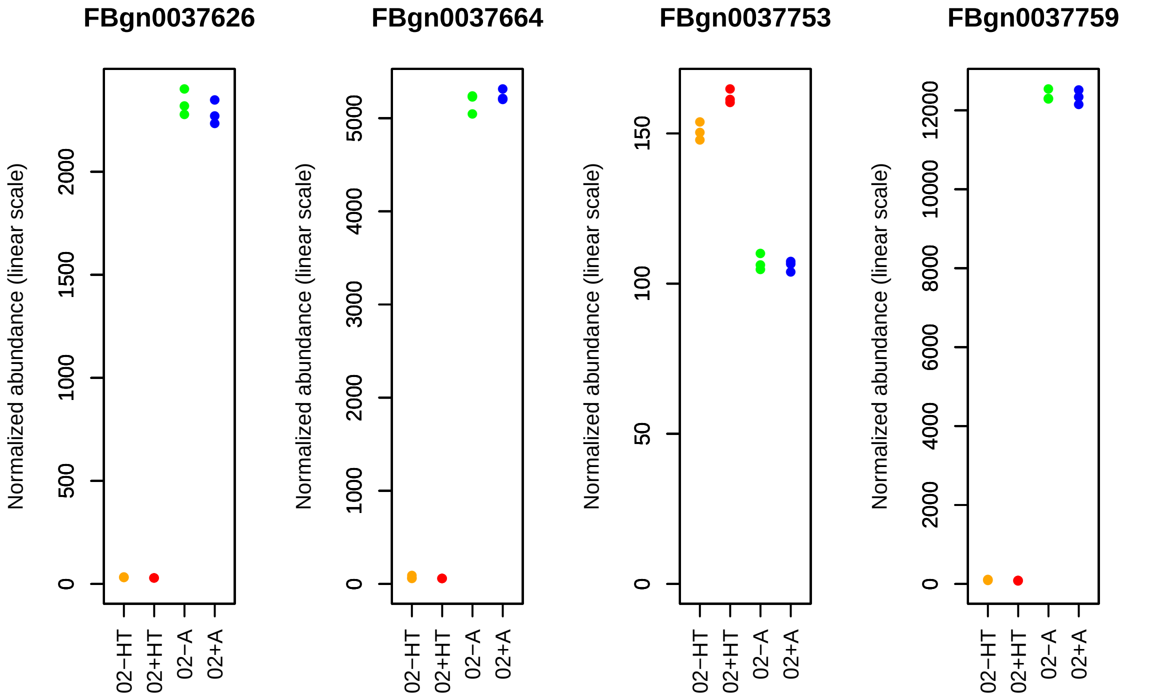
FBgn0037626
FBgn0037664
FBgn0037753
FBgn0037759
Normalized abundance (linear scale)
0
500
1000
1500
2000
0
1000
2000
3000
4000
5000
0
50
100
150
0
2000
4000
6000
8000
10000
12000
02−HT
02+HT
02−A
02+A

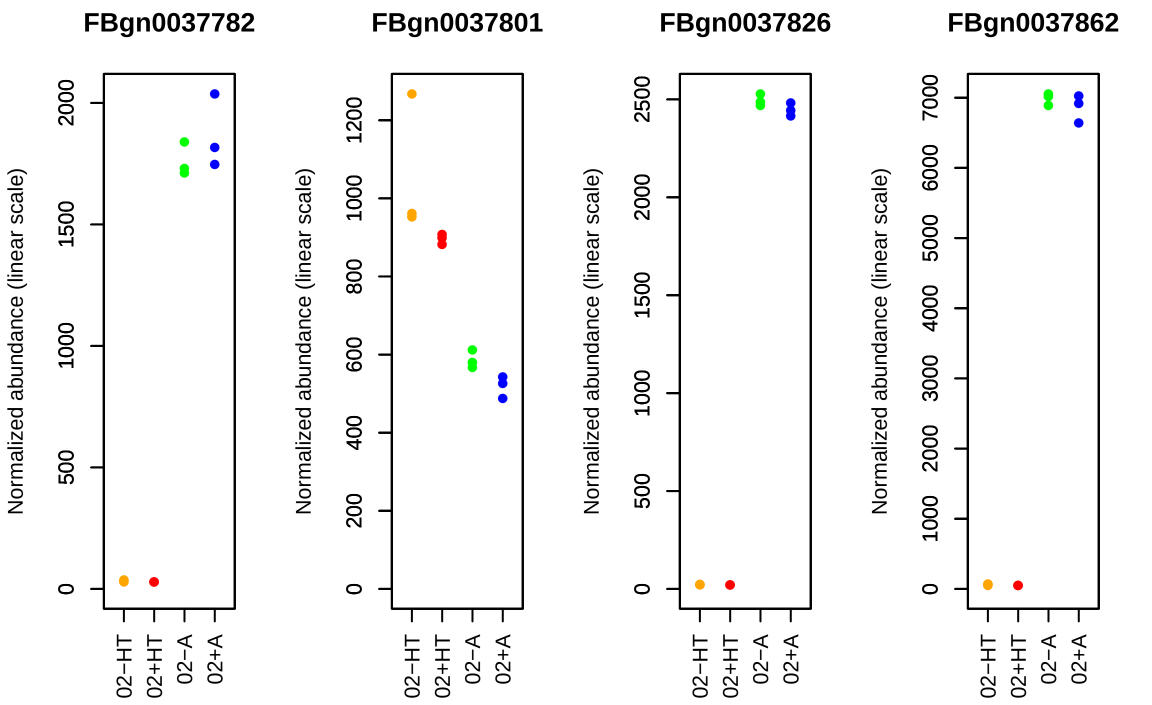

FBgn0037782
FBgn0037801
FBgn0037826
FBgn0037862
Normalized abundance (linear scale)
0
500
1000
1500
2000
0
200
400
600
800
1000
1200
0
500
1000
1500
2000
2500
0
1000
2000
3000
4000
5000
6000
7000
02−HT
02+HT
02−A
02+A

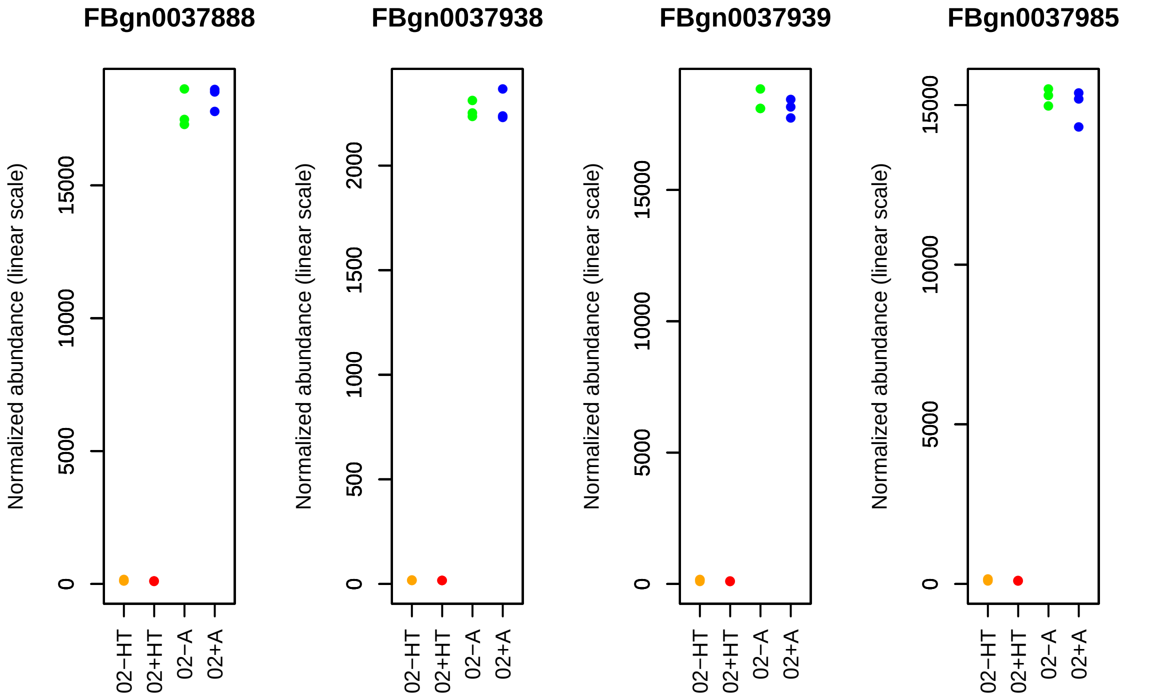
FBgn0037888
FBgn0037938
FBgn0037939
FBgn0037985
Normalized abundance (linear scale)
0
5000
10000
15000
500
1000
1500
2000
02−HT
02+HT
02−A
02+A

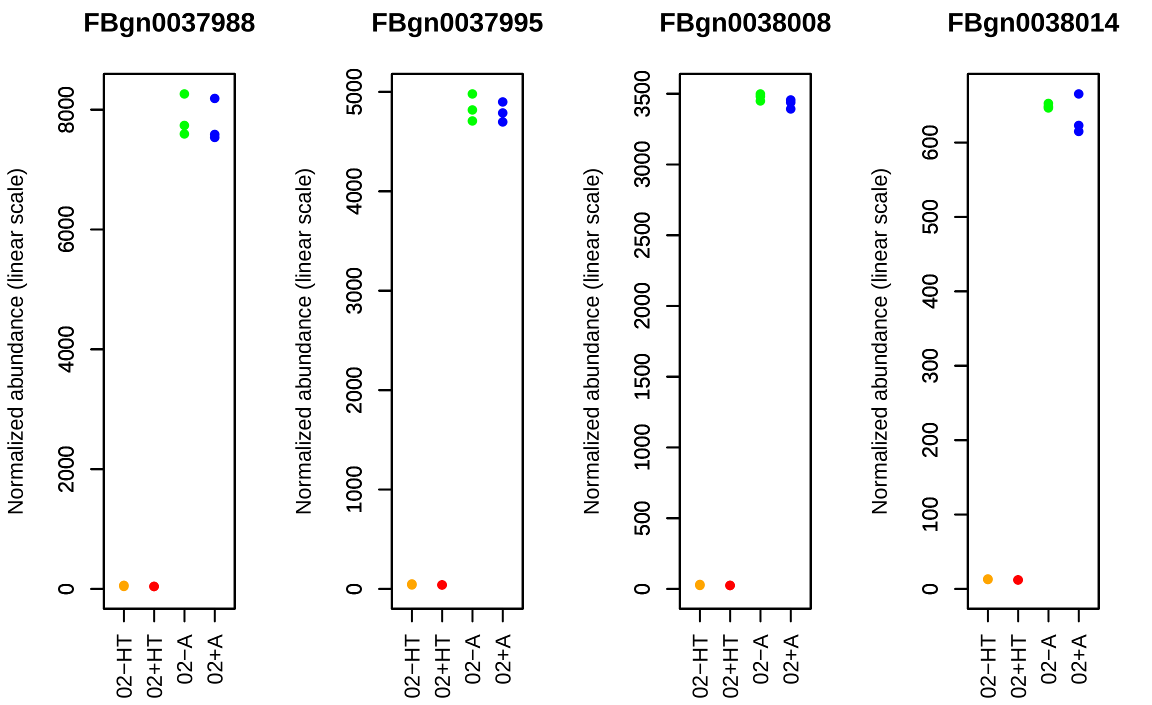

FBgn0037988
FBgn0037995
FBgn0038008
FBgn0038014
Normalized abundance (linear scale)
0
2000
4000
6000
8000
1000
3000
5000
500
1500
2500
3500
100
200
300
400
600
02−HT
02+HT
02−A
02+A

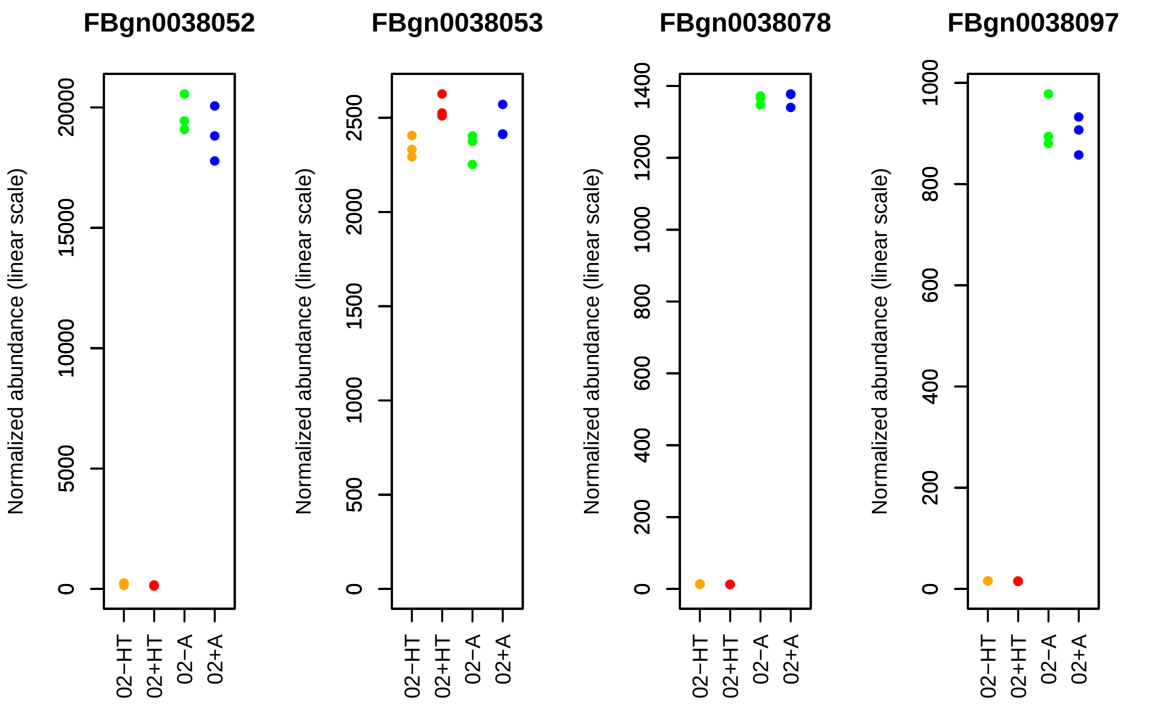

FBgn0038052
FBgn0038053
FBgn0038078
FBgn0038097
Normalized abundance (linear scale)
0
5000
10000
15000
20000
500
1000
1500
2000
2500
200
400
600
800
1000
1200
1400
02−HT
02+HT
02−A
02+A

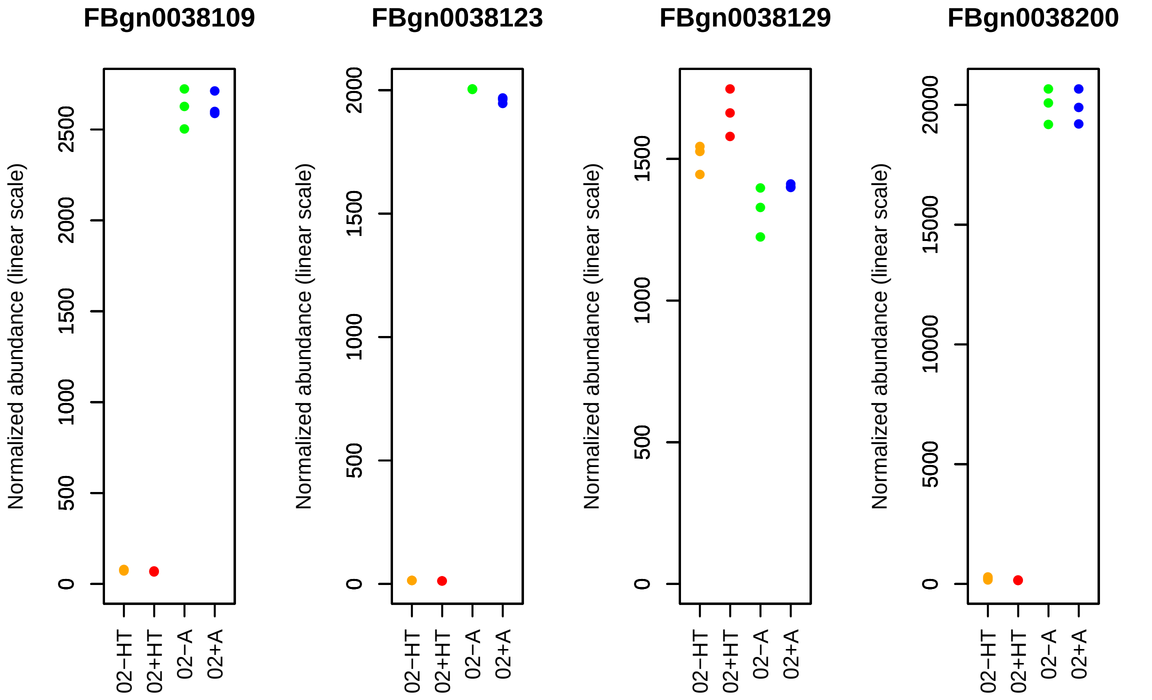

FBgn0038109
FBgn0038123
FBgn0038129
FBgn0038200
Normalized abundance (linear scale)
0
500
1000
1500
2000
2500
5000
10000
15000
20000
02−HT
02+HT
02−A
02+A

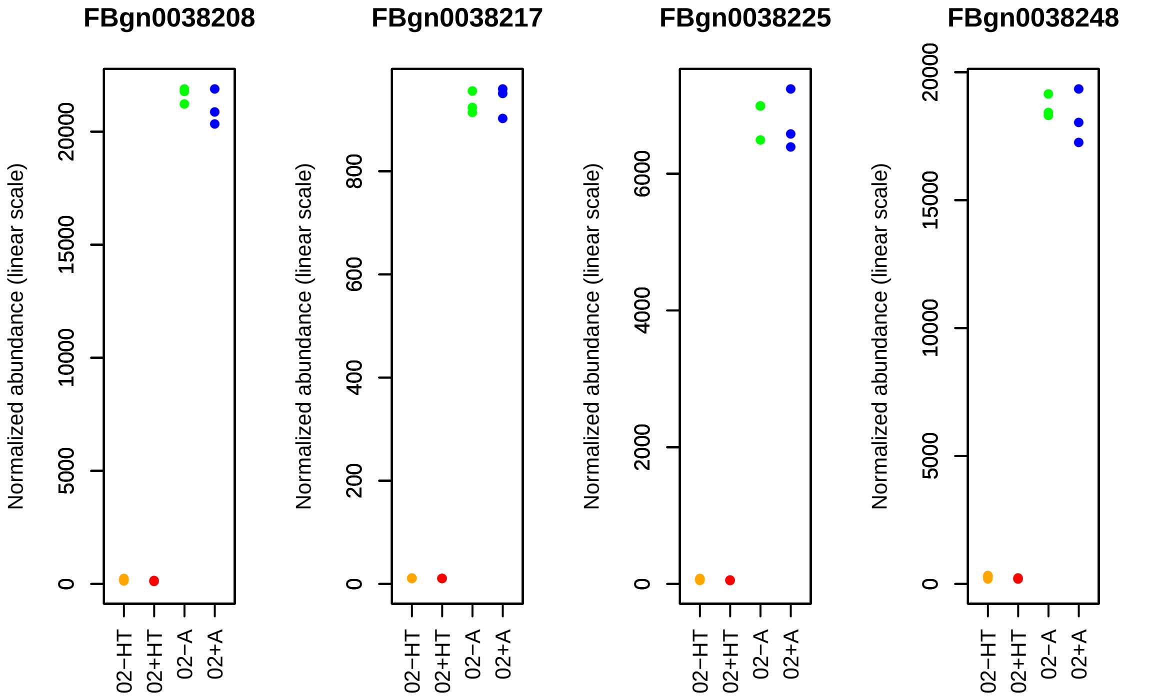
FBgn0038208
FBgn0038217
FBgn0038225
FBgn0038248
Normalized abundance (linear scale)
0
5000
10000
15000
20000
200
400
600
800
2000
4000
6000
02−HT
02+HT
02−A
02+A

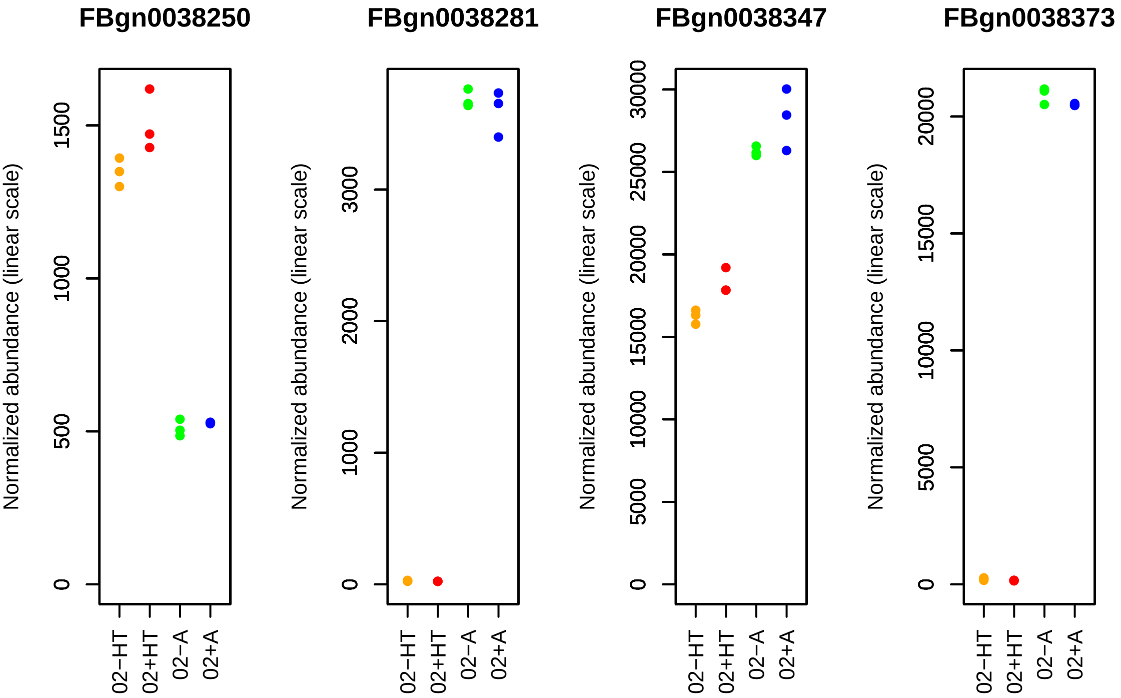

FBgn0038250
FBgn0038281
FBgn0038347
FBgn0038373
Normalized abundance (linear scale)
0
500
1000
1500
2000
3000
5000
10000
15000
20000
25000
30000
02−HT
02+HT
02−A
02+A

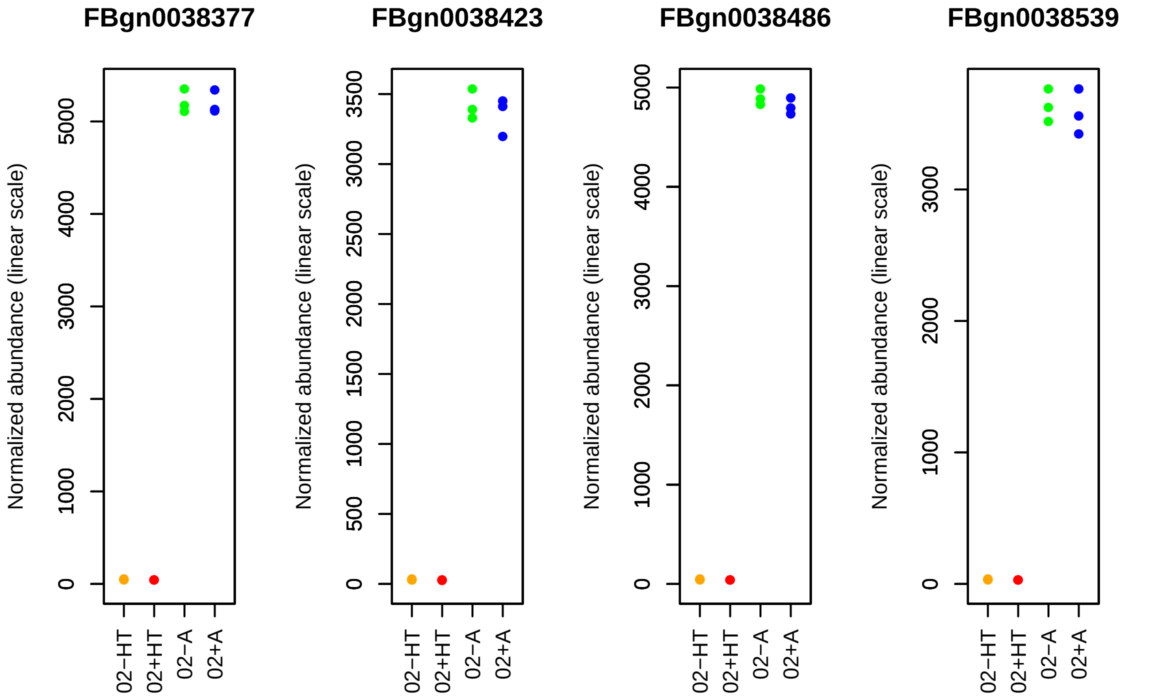
FBgn0038377
FBgn0038423
FBgn0038486
FBgn0038539
Normalized abundance (linear scale)
0
1000
2000
3000
4000
5000
500
1500
2500
3500
02−HT
02+HT
02−A
02+A

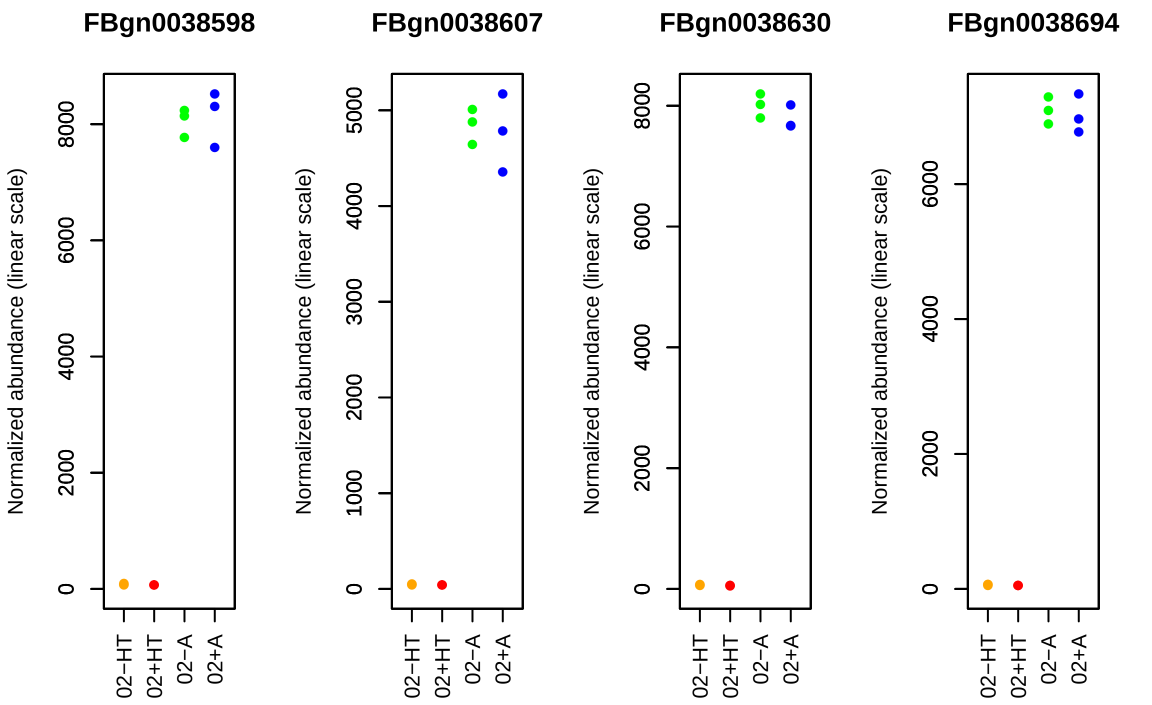
FBgn0038598
FBgn0038607
FBgn0038630
FBgn0038694
Normalized abundance (linear scale)
0
2000
4000
6000
8000
1000
3000
5000
02−HT
02+HT
02−A
02+A

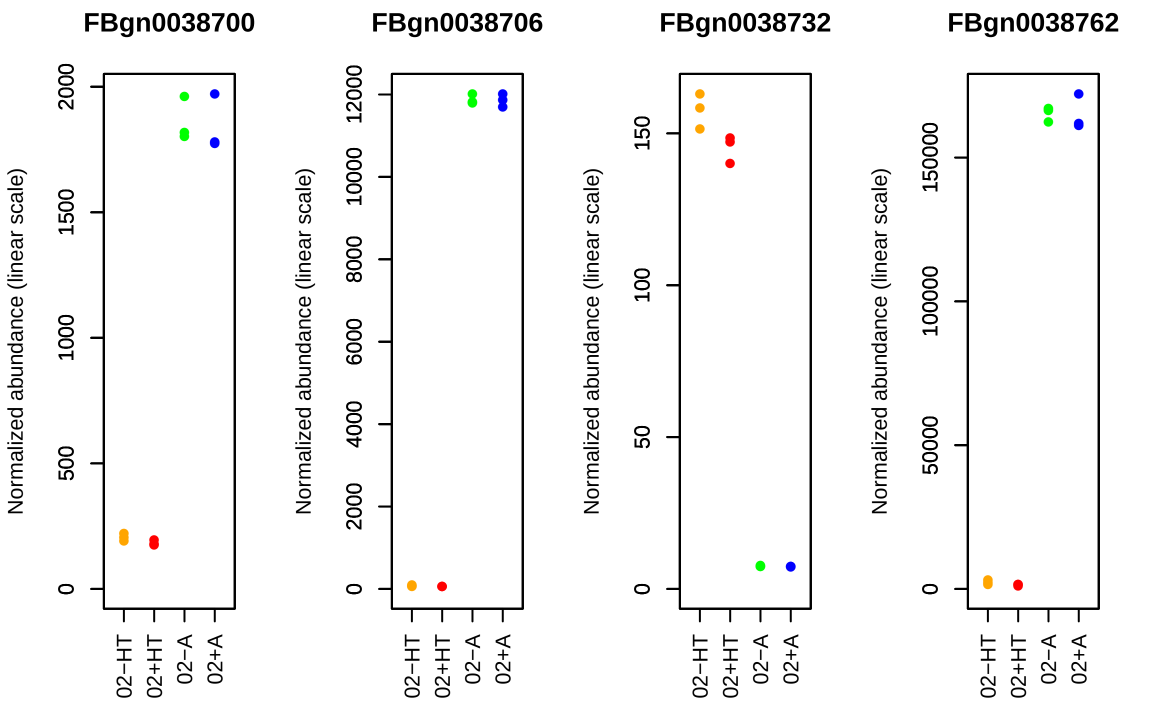
FBgn0038700
FBgn0038706
FBgn0038732
FBgn0038762
Normalized abundance (linear scale)
0
500
1000
1500
2000
0
2000
4000
6000
8000
10000
12000
0
50
100
150
0
50000
100000
150000
02−HT
02+HT
02−A
02+A

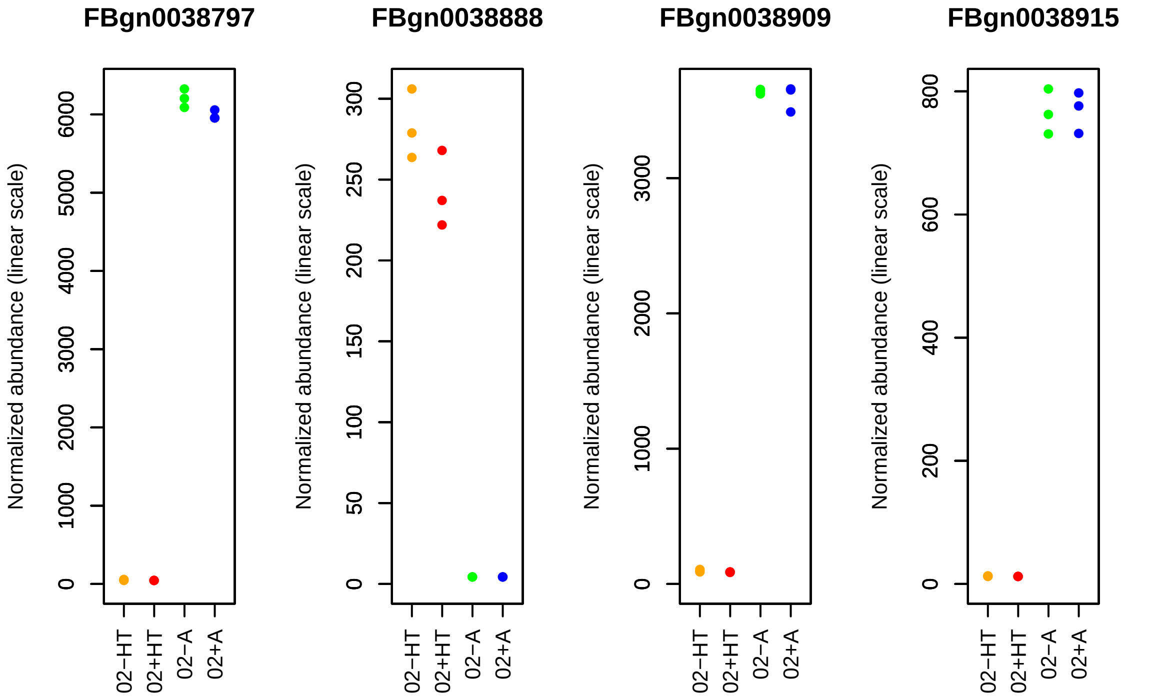
FBgn0038797
FBgn0038888
FBgn0038909
FBgn0038915
Normalized abundance (linear scale)
0
1000
2000
3000
4000
5000
6000
50
100
150
200
250
300
200
400
600
800
02−HT
02+HT
02−A
02+A

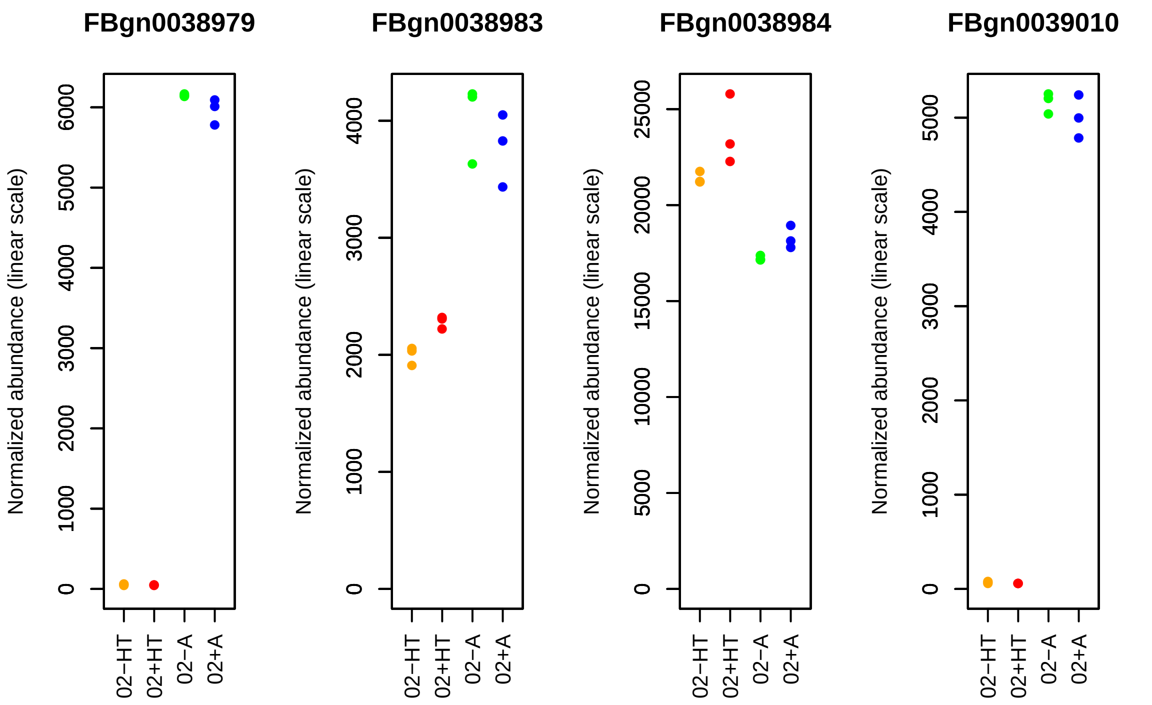
FBgn0038979
FBgn0038983
FBgn0038984
FBgn0039010
Normalized abundance (linear scale)
0
1000
2000
3000
4000
5000
6000
0
1000
2000
3000
4000
0
5000
10000
15000
20000
25000
02−HT
02+HT
02−A
02+A

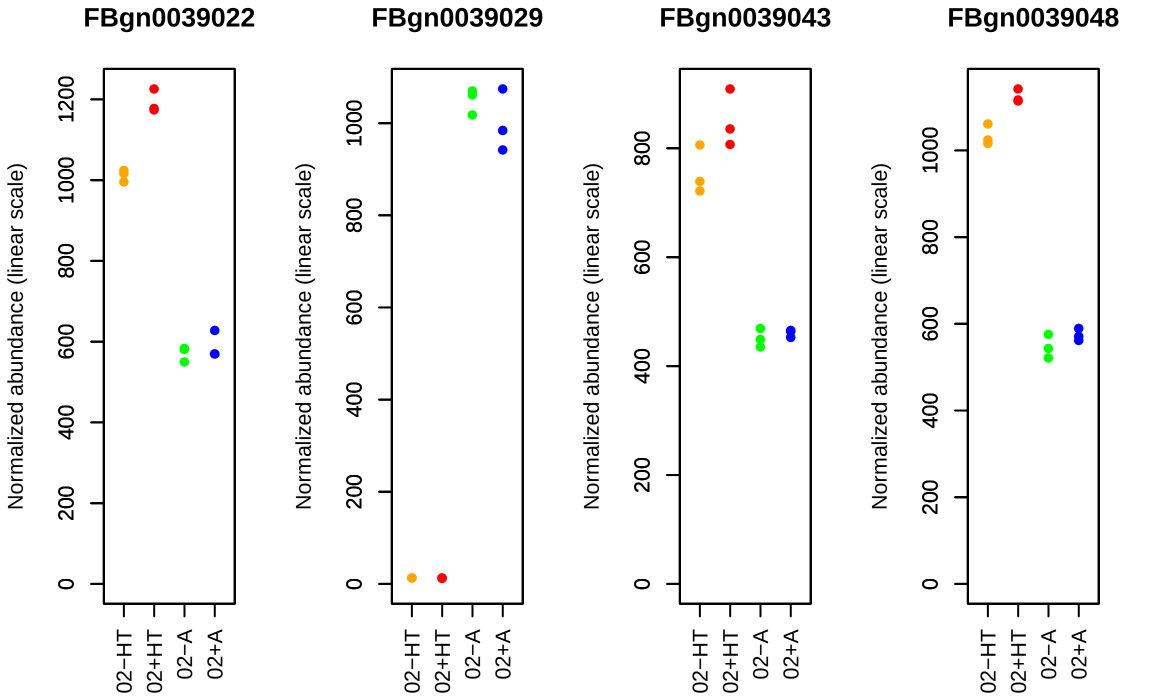

FBgn0039022
FBgn0039029
FBgn0039043
FBgn0039048
Normalized abundance (linear scale)
0
200
400
600
800
1000
1200
02−HT
02+HT
02−A
02+A

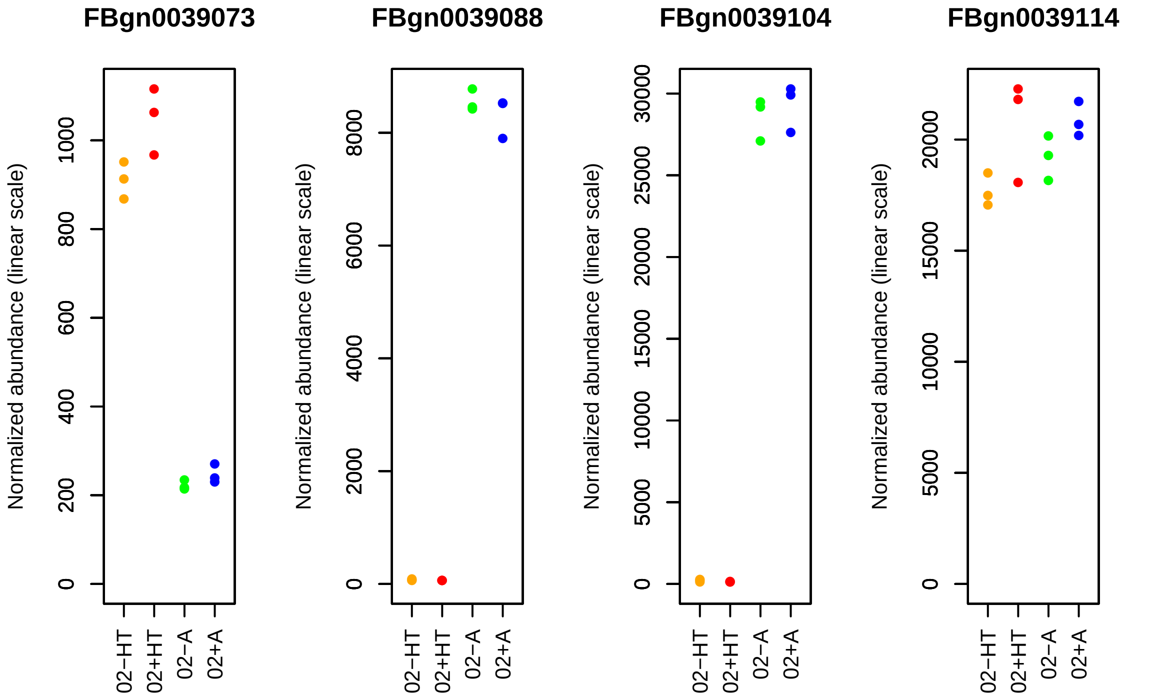
FBgn0039073
FBgn0039088
FBgn0039104
FBgn0039114
Normalized abundance (linear scale)
0
200
400
600
800
1000
2000
4000
6000
8000
5000
10000
15000
20000
25000
30000
02−HT
02+HT
02−A
02+A

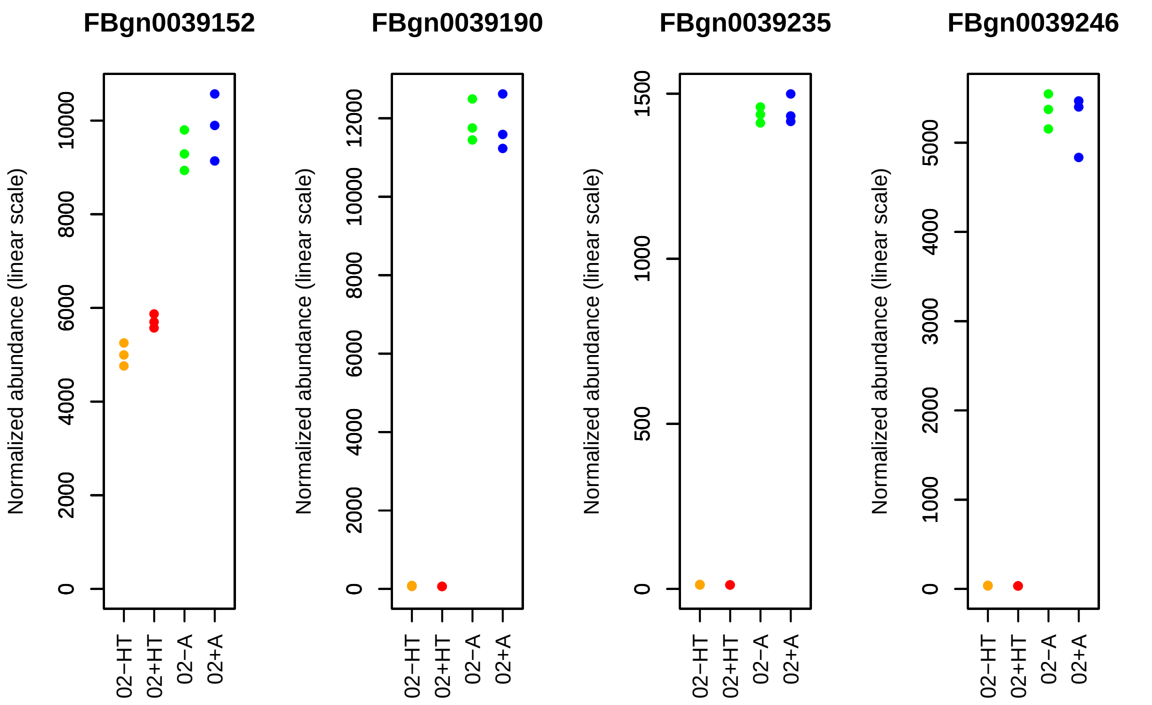

FBgn0039152
FBgn0039190
FBgn0039235
FBgn0039246
Normalized abundance (linear scale)
0
2000
4000
6000
8000
10000
12000
500
1000
1500
3000
5000
02−HT
02+HT
02−A
02+A

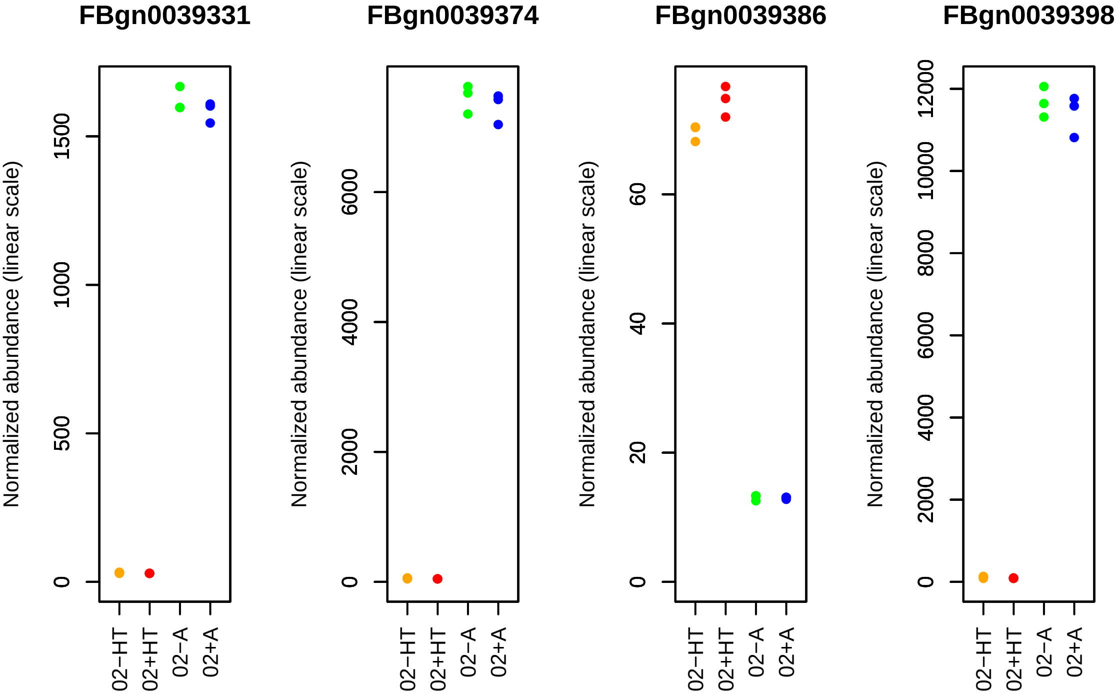
FBgn0039331
FBgn0039374
FBgn0039386
FBgn0039398
Normalized abundance (linear scale)
0
500
1000
1500
2000
4000
6000
20
40
60
8000
10000
12000
02−HT
02+HT
02−A
02+A

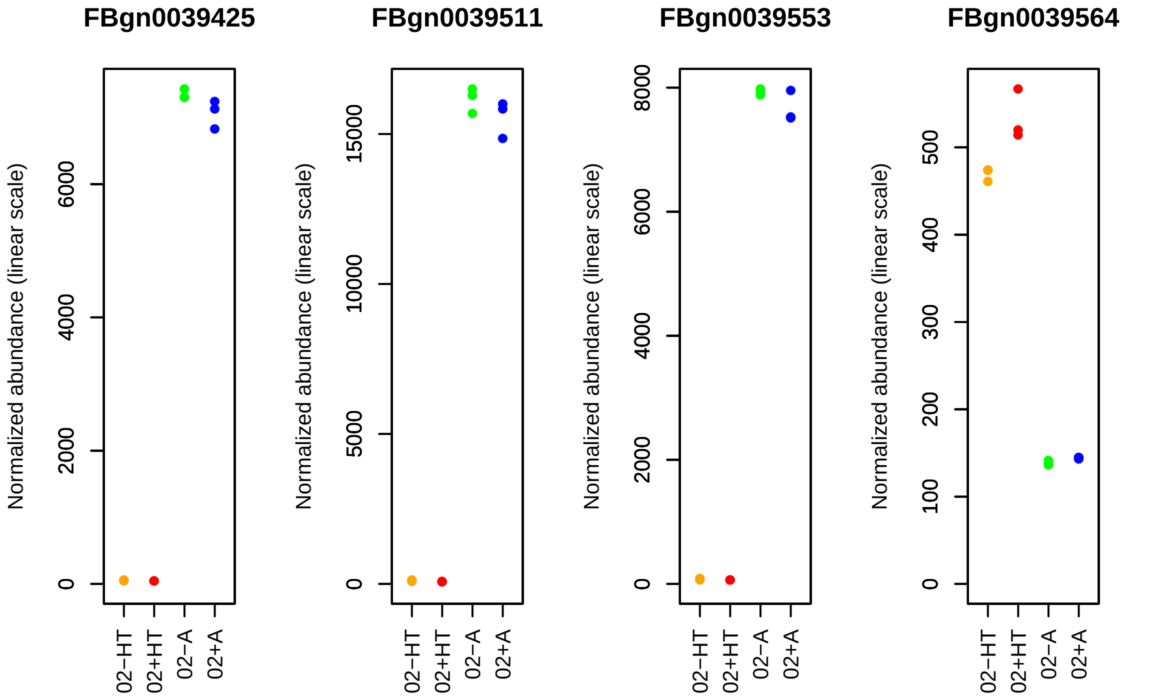

FBgn0039425
FBgn0039511
FBgn0039553
FBgn0039564
Normalized abundance (linear scale)
0
2000
4000
6000
5000
10000
15000
8000
100
200
300
400
500
02−HT
02+HT
02−A
02+A

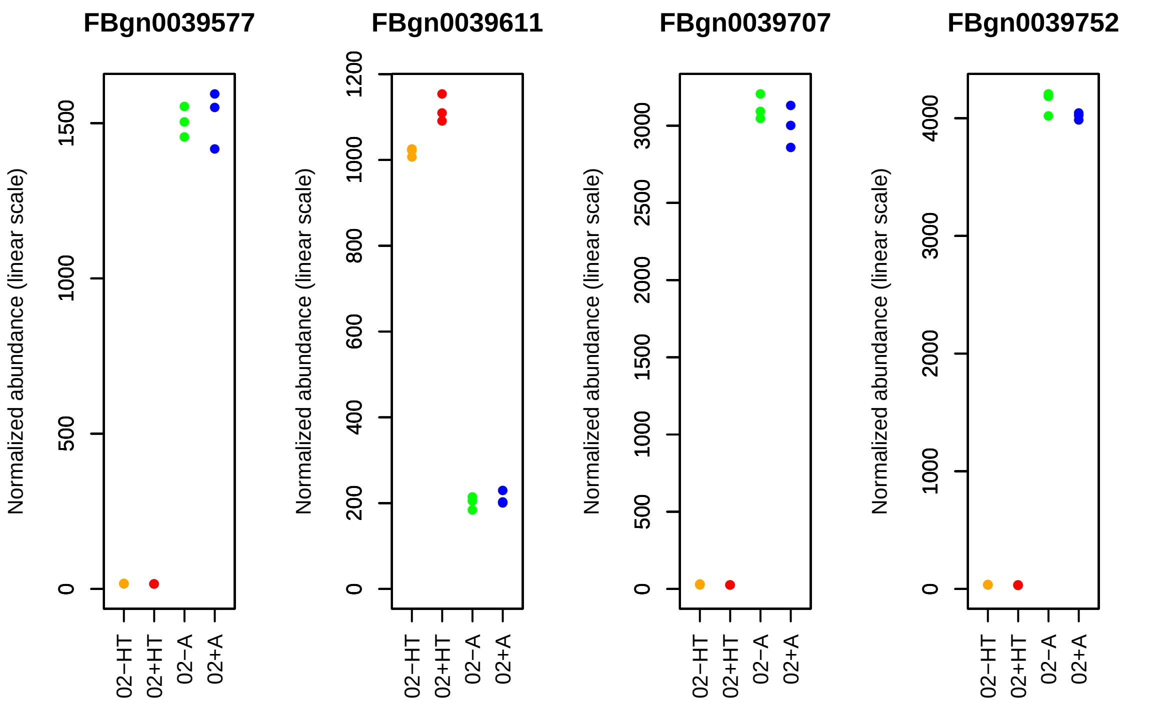

FBgn0039577
FBgn0039611
FBgn0039707
FBgn0039752
Normalized abundance (linear scale)
0
500
1000
1500
200
400
600
800
1200
2000
2500
3000
4000
02−HT
02+HT
02−A
02+A

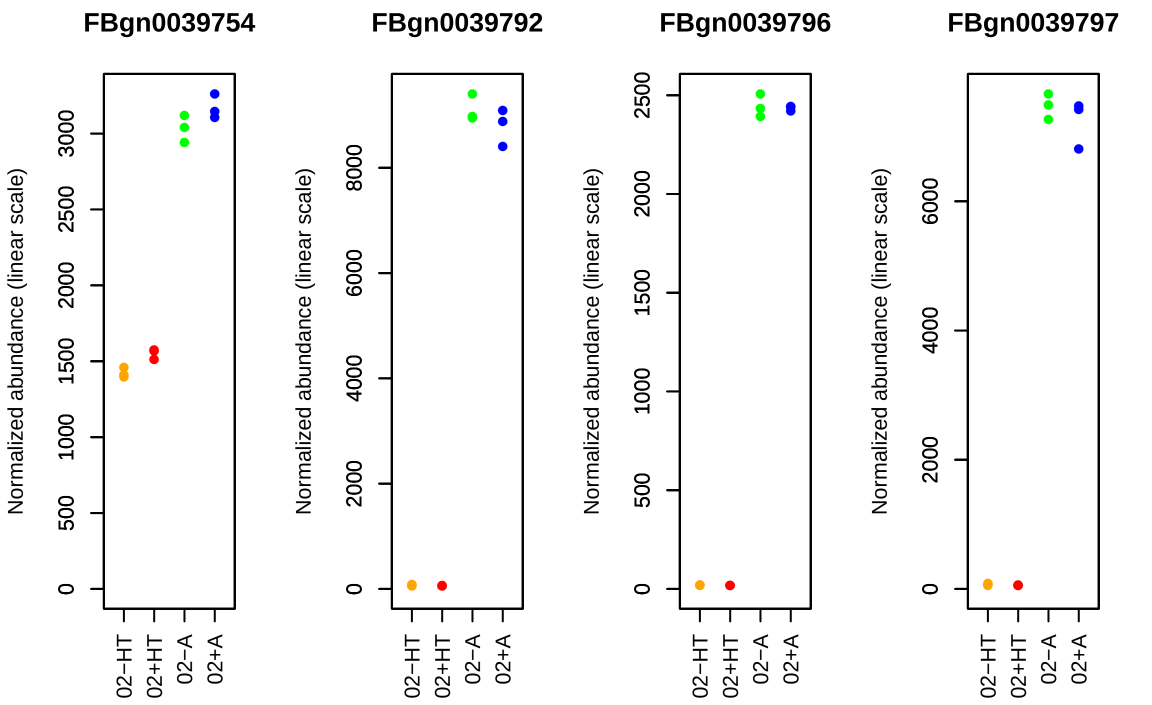
FBgn0039754
FBgn0039792
FBgn0039796
FBgn0039797
Normalized abundance (linear scale)
02−HT
02+HT
02−A
02+A

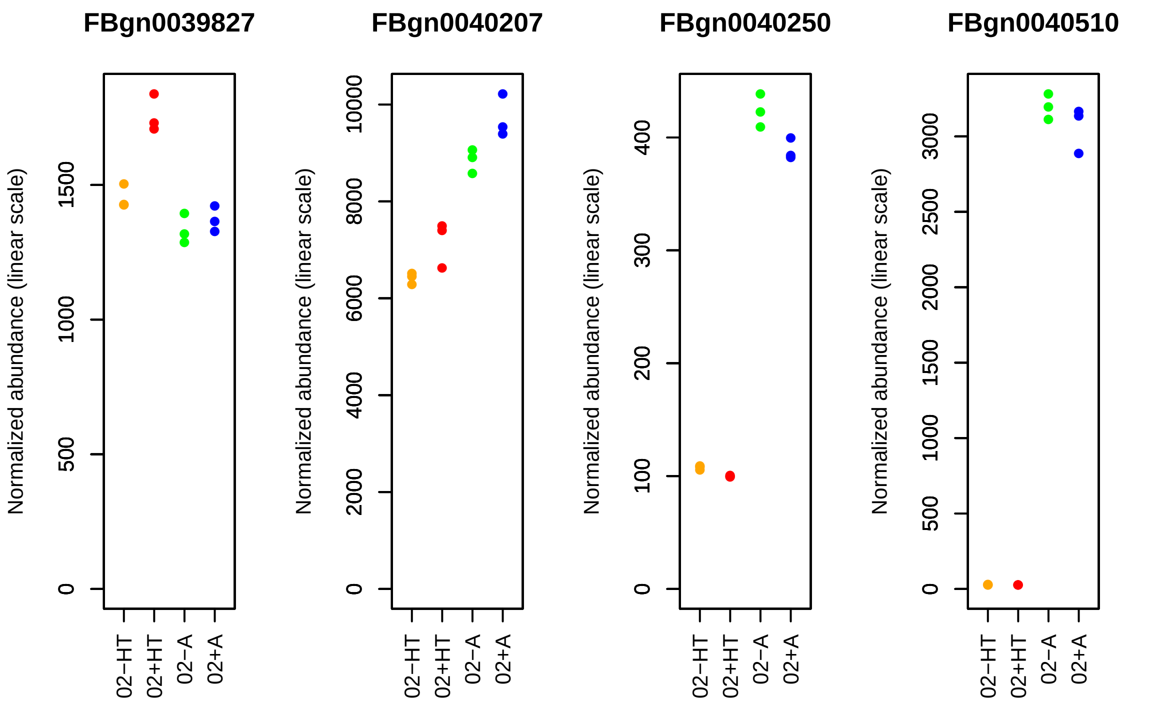
FBgn0039827
FBgn0040207
FBgn0040250
FBgn0040510
Normalized abundance (linear scale)
0
500
1000
1500
2000
4000
6000
8000
10000
100
200
300
400
2500
3000
02−HT
02+HT
02−A
02+A

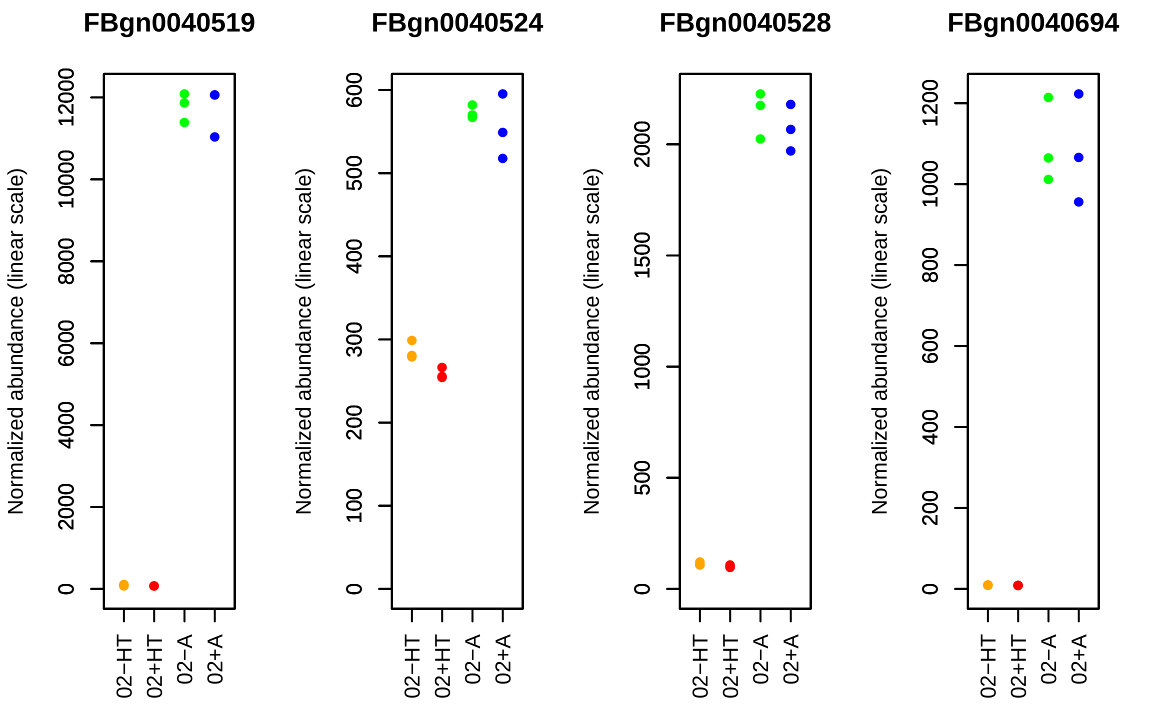
FBgn0040519
FBgn0040524
FBgn0040528
FBgn0040694
Normalized abundance (linear scale)
0
2000
4000
6000
8000
10000
12000
100
200
300
400
500
600
500
1000
1500
2000
200
400
600
800
1000
1200
02−HT
02+HT
02−A
02+A

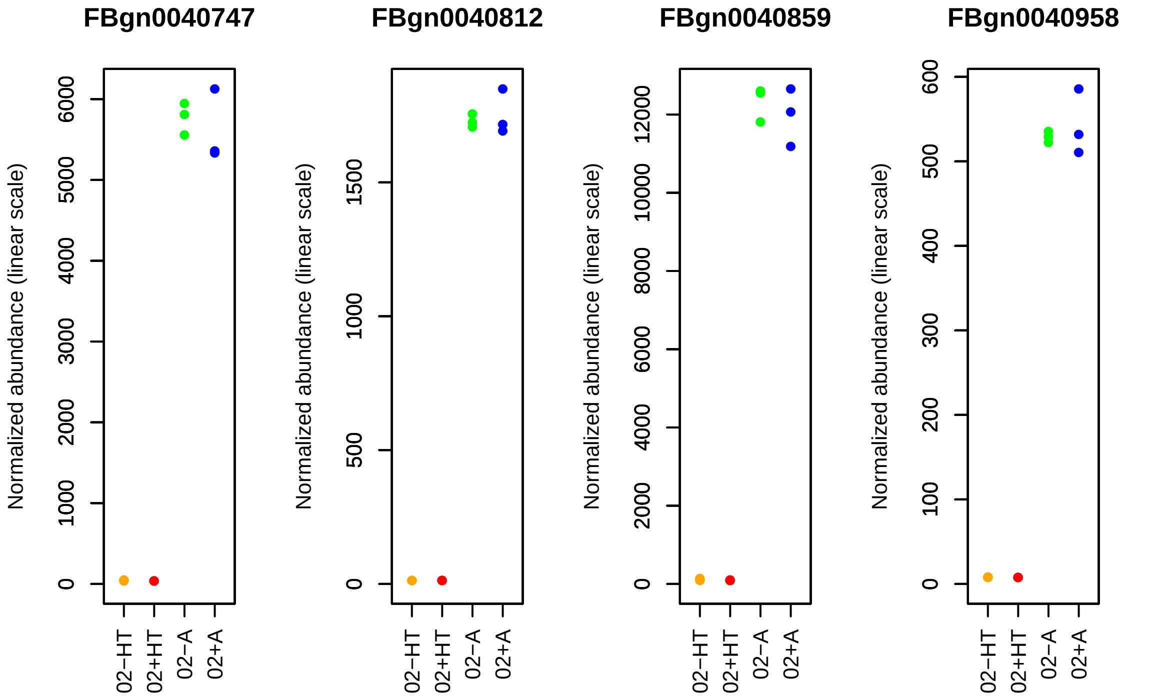

FBgn0040747
FBgn0040812
FBgn0040859
FBgn0040958
Normalized abundance (linear scale)
0
1000
2000
3000
4000
5000
6000
500
1500
8000
10000
12000
100
200
300
400
600
02−HT
02+HT
02−A
02+A

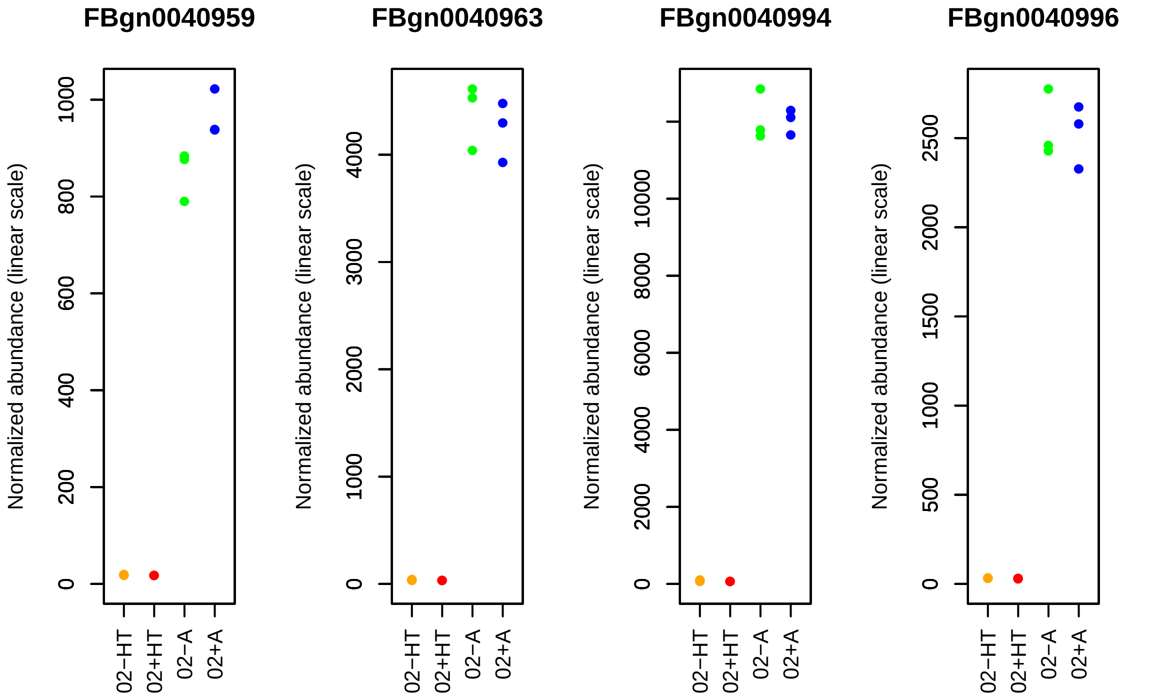

FBgn0040959
FBgn0040963
FBgn0040994
FBgn0040996
Normalized abundance (linear scale)
0
200
400
600
800
1000
1000
2000
3000
4000
2000
4000
6000
8000
10000
500
1500
2500
02−HT
02+HT
02−A
02+A

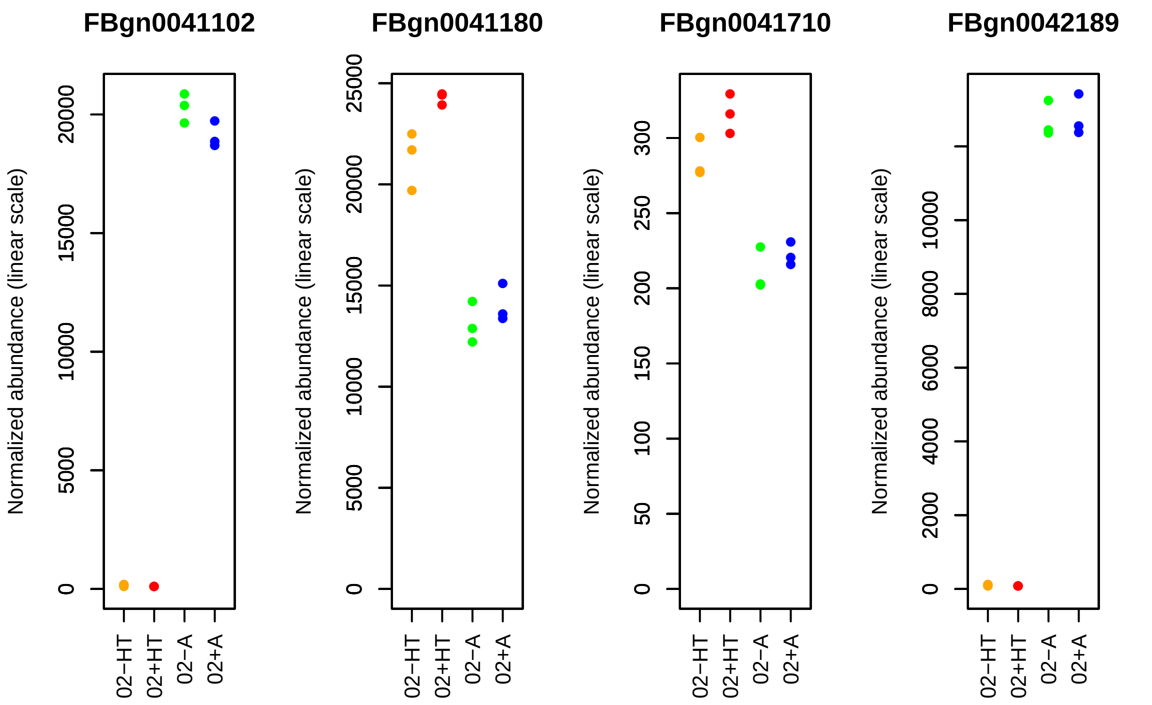

FBgn0041102
FBgn0041180
FBgn0041710
FBgn0042189
Normalized abundance (linear scale)
0
5000
10000
15000
20000
25000
50
100
150
200
250
300
2000
4000
6000
8000
10000
02−HT
02+HT
02−A
02+A

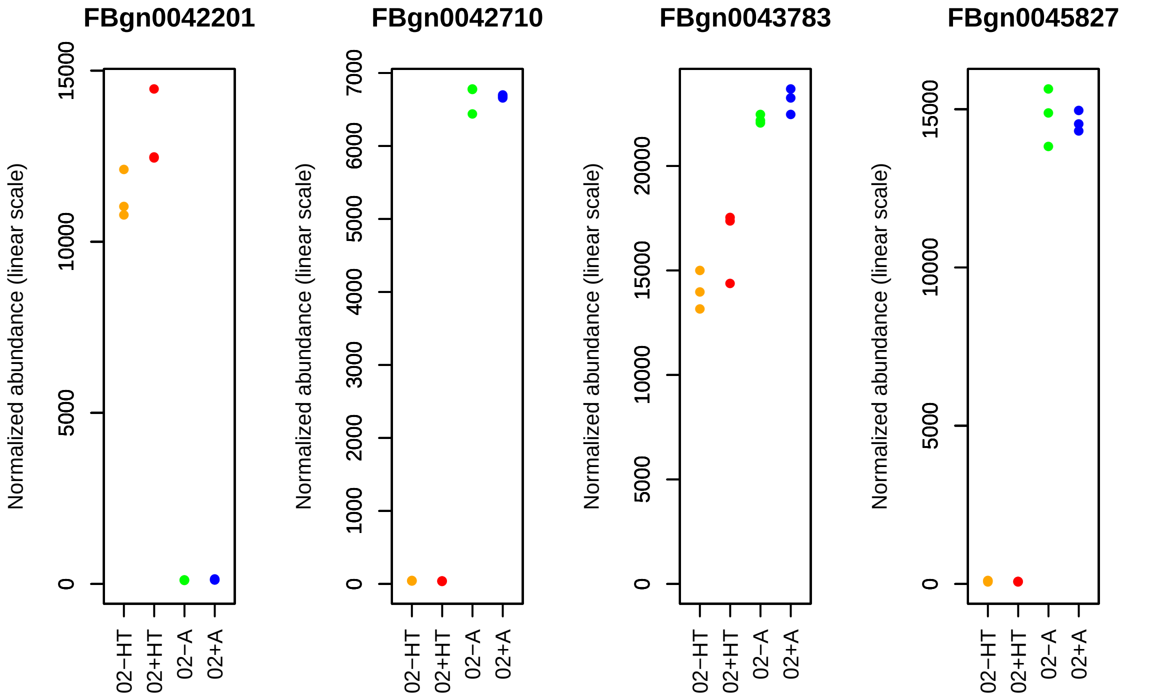

FBgn0042201
FBgn0042710
FBgn0043783
FBgn0045827
Normalized abundance (linear scale)
0
5000
10000
15000
1000
2000
3000
4000
5000
6000
7000
20000
02−HT
02+HT
02−A
02+A

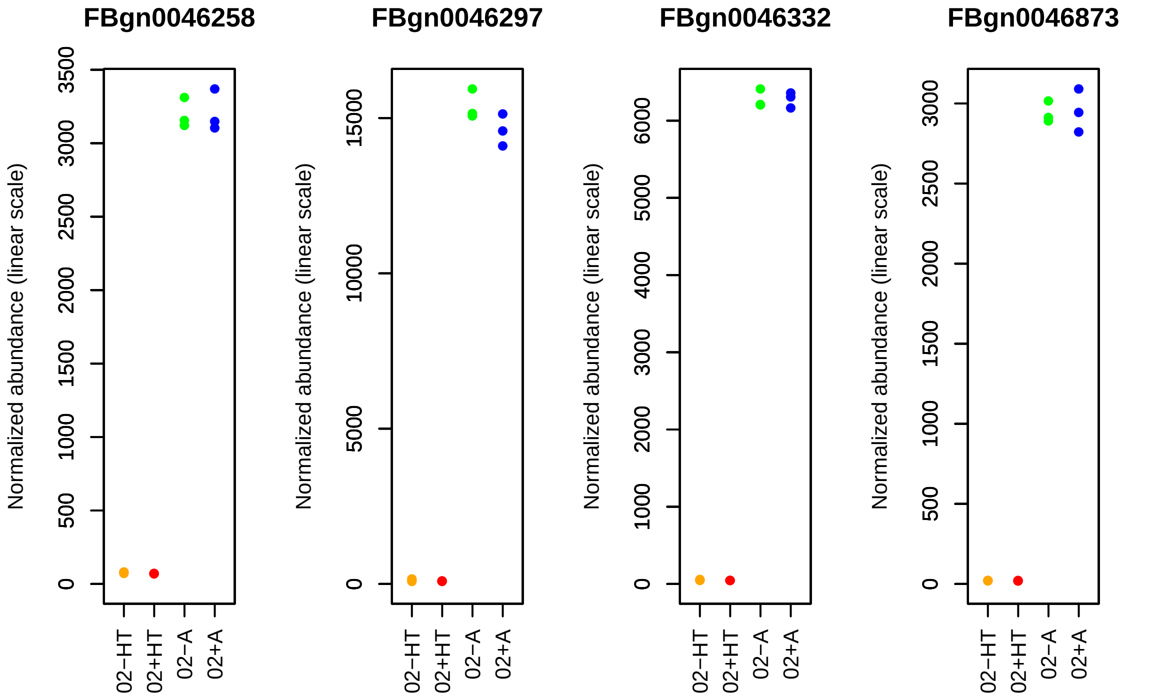
FBgn0046258
FBgn0046297
FBgn0046332
FBgn0046873
Normalized abundance (linear scale)
0
500
1000
1500
2000
2500
3000
3500
5000
10000
15000
4000
6000
02−HT
02+HT
02−A
02+A

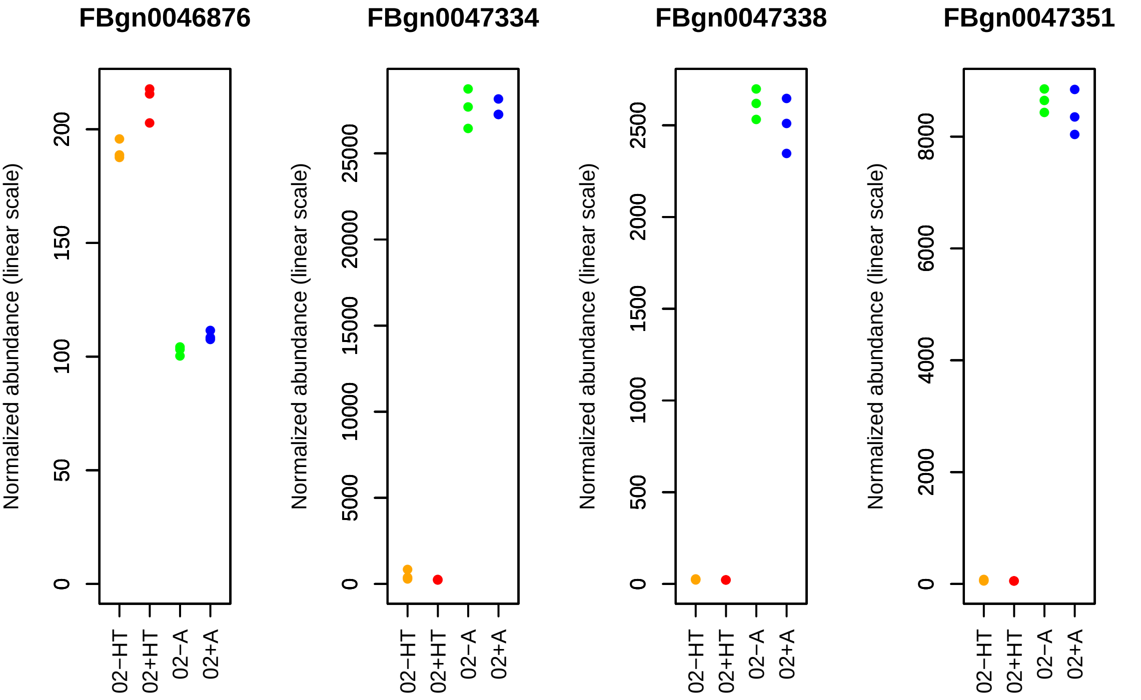
FBgn0046876
FBgn0047334
FBgn0047338
FBgn0047351
Normalized abundance (linear scale)
0
50
100
150
200
0
5000
10000
15000
20000
25000
0
500
1000
1500
2000
2500
0
2000
4000
6000
8000
02−HT
02+HT
02−A
02+A

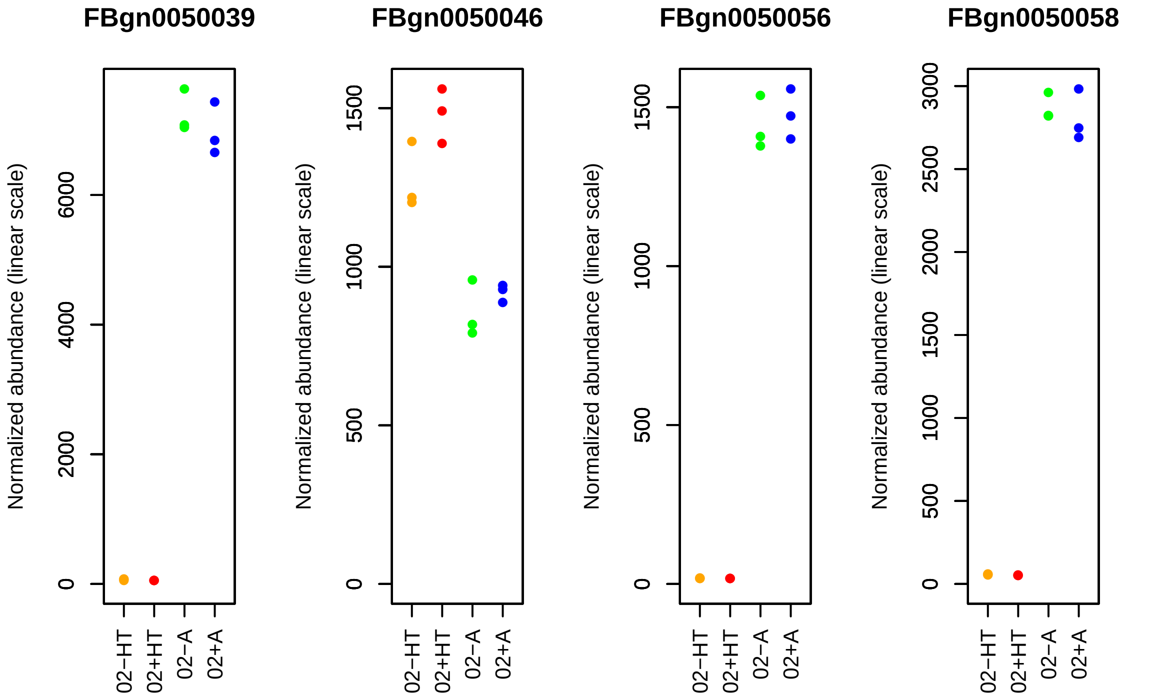
FBgn0050039
FBgn0050046
FBgn0050056
FBgn0050058
Normalized abundance (linear scale)
0
2000
4000
6000
500
1000
1500
2500
3000
02−HT
02+HT
02−A
02+A

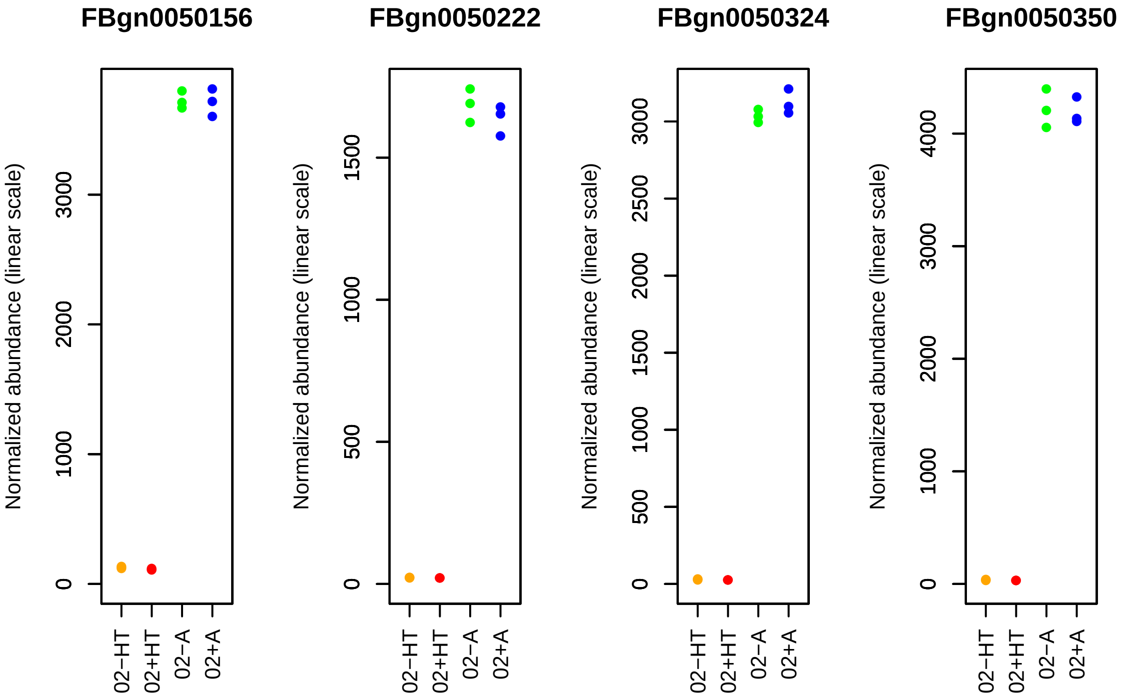

FBgn0050156
FBgn0050222
FBgn0050324
FBgn0050350
Normalized abundance (linear scale)
0
1000
2000
3000
500
1500
2500
4000
02−HT
02+HT
02−A
02+A

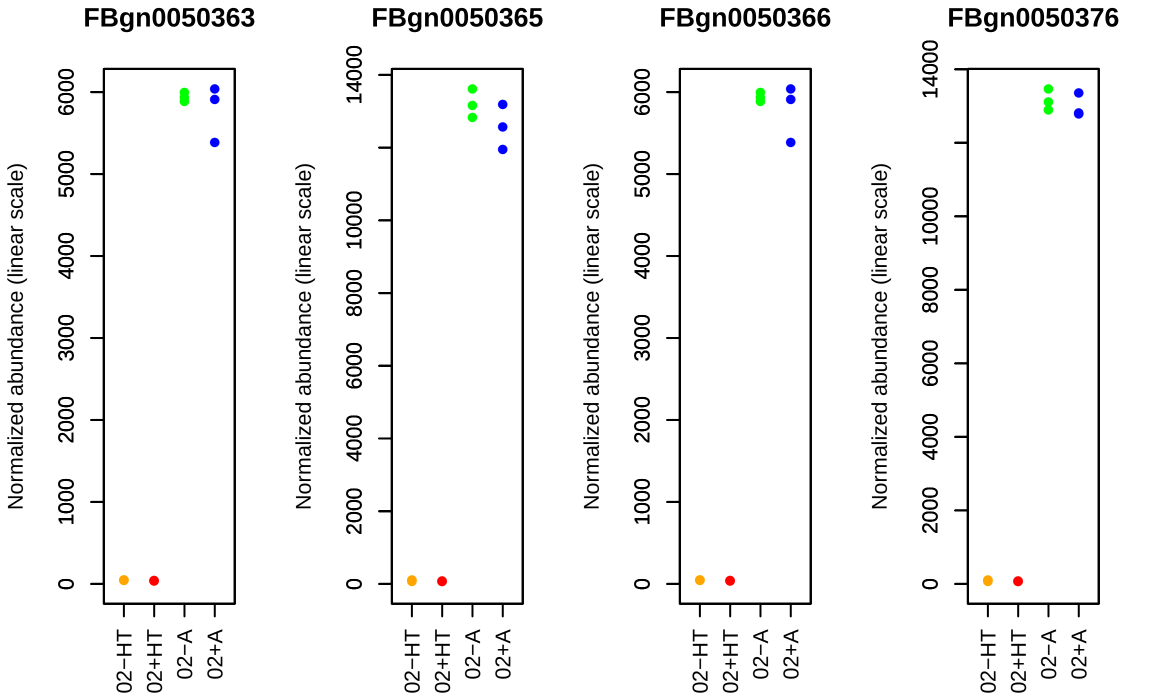
FBgn0050363
FBgn0050365
FBgn0050366
FBgn0050376
Normalized abundance (linear scale)
0
1000
2000
3000
4000
5000
6000
8000
10000
14000
02−HT
02+HT
02−A
02+A

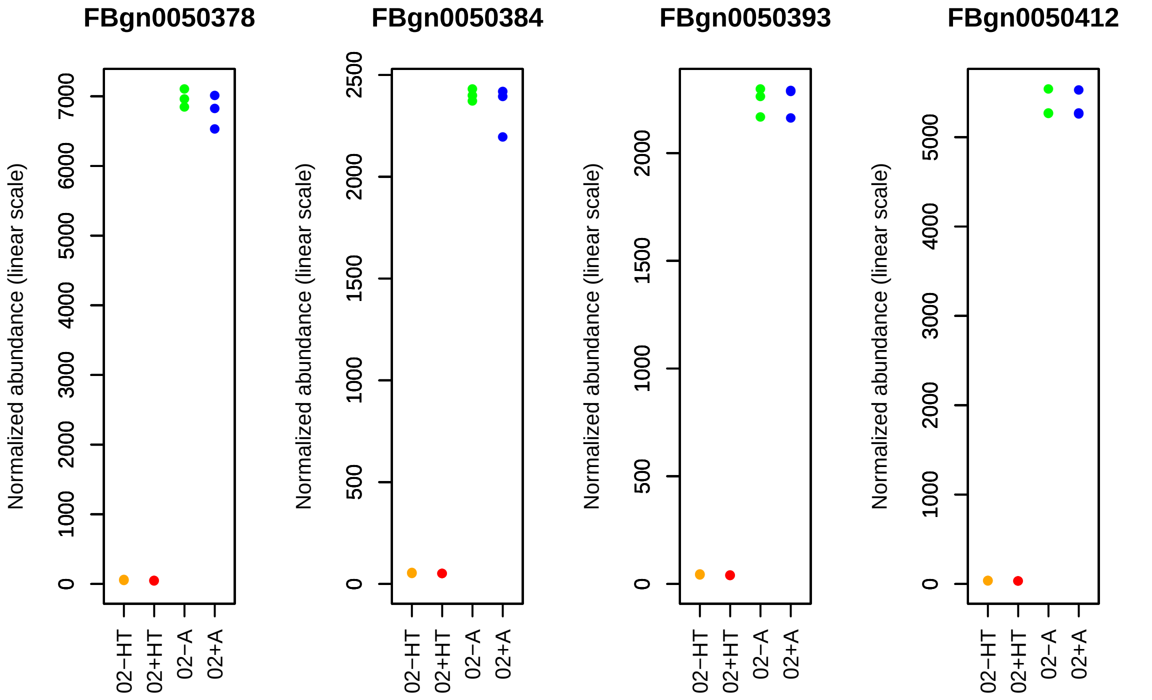

FBgn0050378
FBgn0050384
FBgn0050393
FBgn0050412
Normalized abundance (linear scale)
0
1000
2000
3000
4000
5000
6000
7000
500
1500
2500
02−HT
02+HT
02−A
02+A

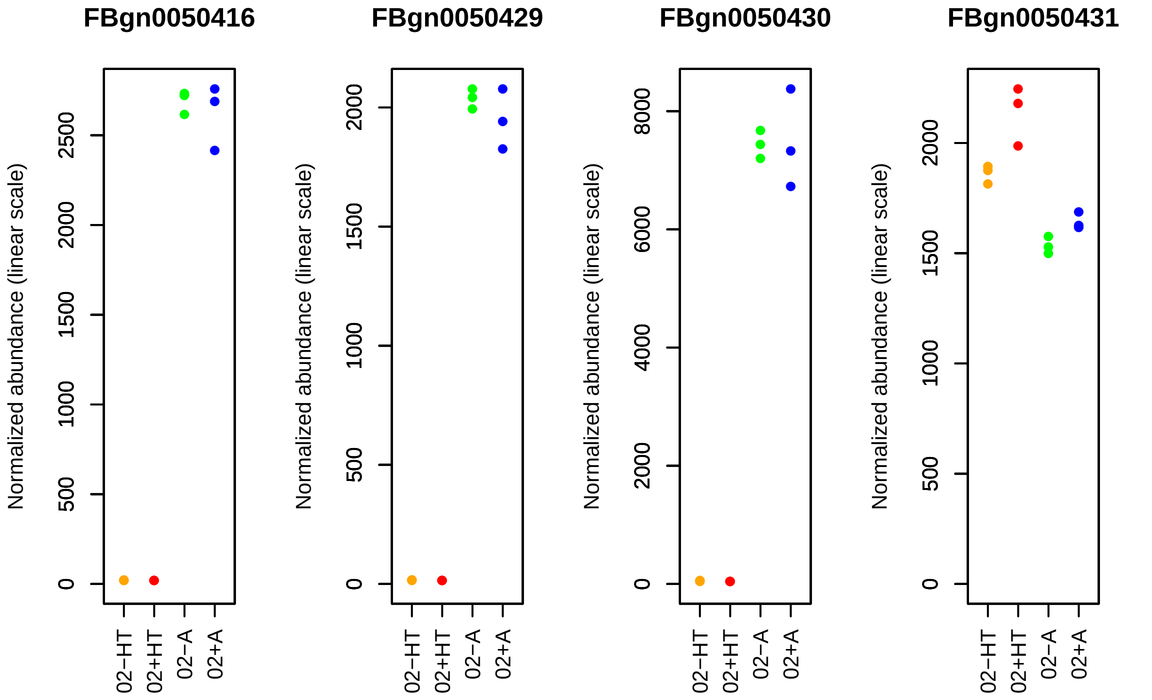
FBgn0050416
FBgn0050429
FBgn0050430
FBgn0050431
Normalized abundance (linear scale)
0
500
1000
1500
2000
2500
8000
6000
4000
02−HT
02+HT
02−A
02+A

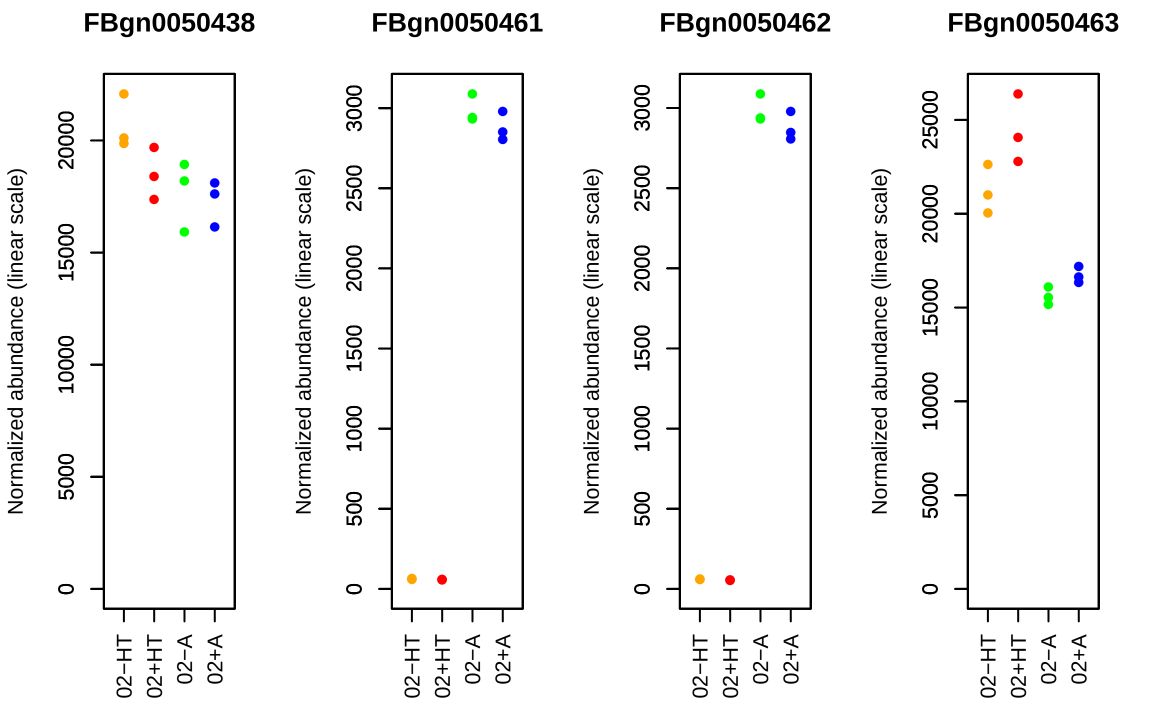
FBgn0050438
FBgn0050461
FBgn0050462
FBgn0050463
Normalized abundance (linear scale)
0
5000
10000
15000
20000
25000
500
1000
1500
2000
2500
3000
02−HT
02+HT
02−A
02+A

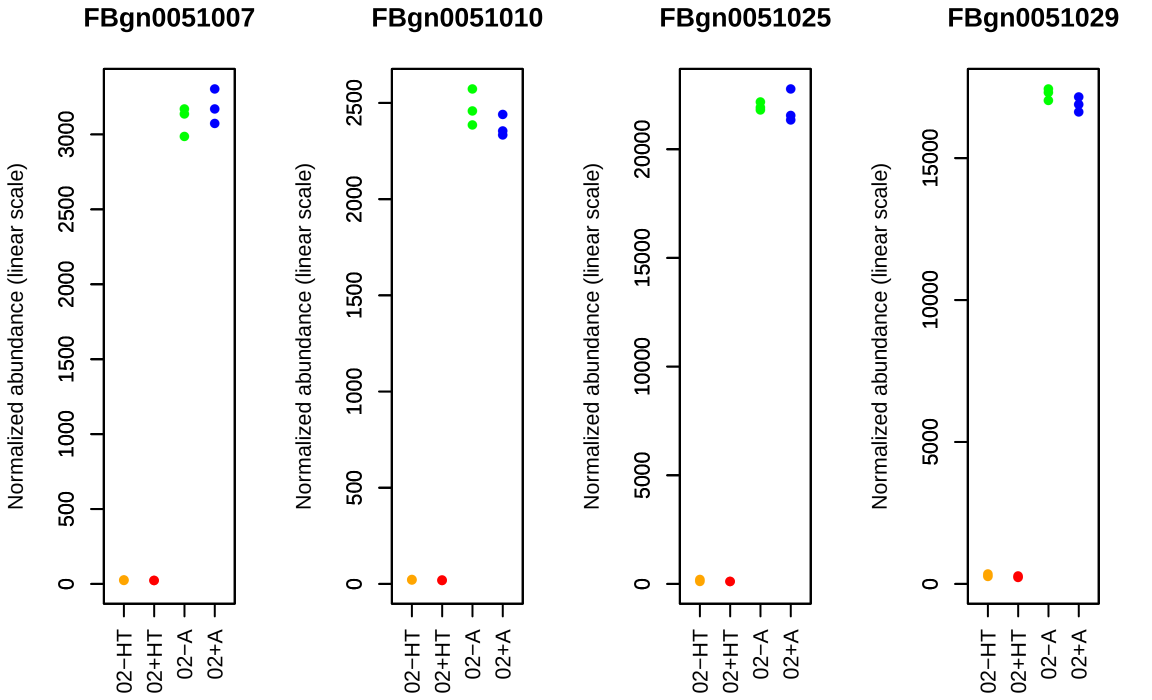
FBgn0051007
FBgn0051010
FBgn0051025
FBgn0051029
Normalized abundance (linear scale)
0
500
1000
1500
2000
2500
3000
5000
10000
15000
20000
02−HT
02+HT
02−A
02+A

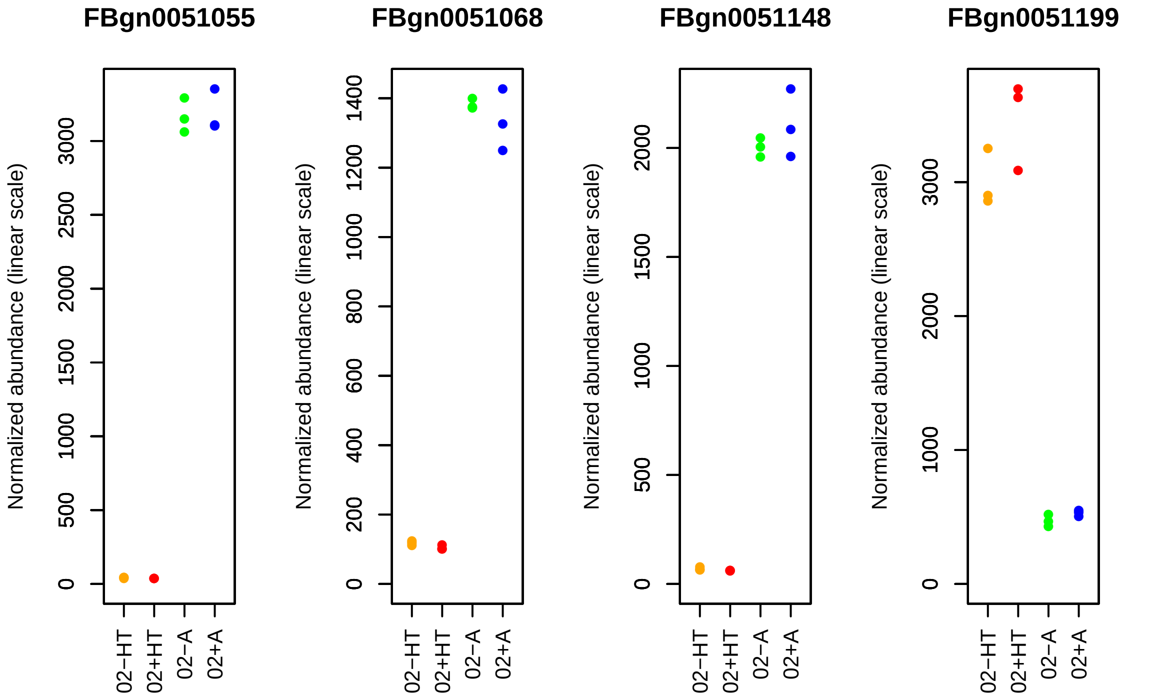

FBgn0051055
FBgn0051068
FBgn0051148
FBgn0051199
Normalized abundance (linear scale)
02−HT
02+HT
02−A
02+A

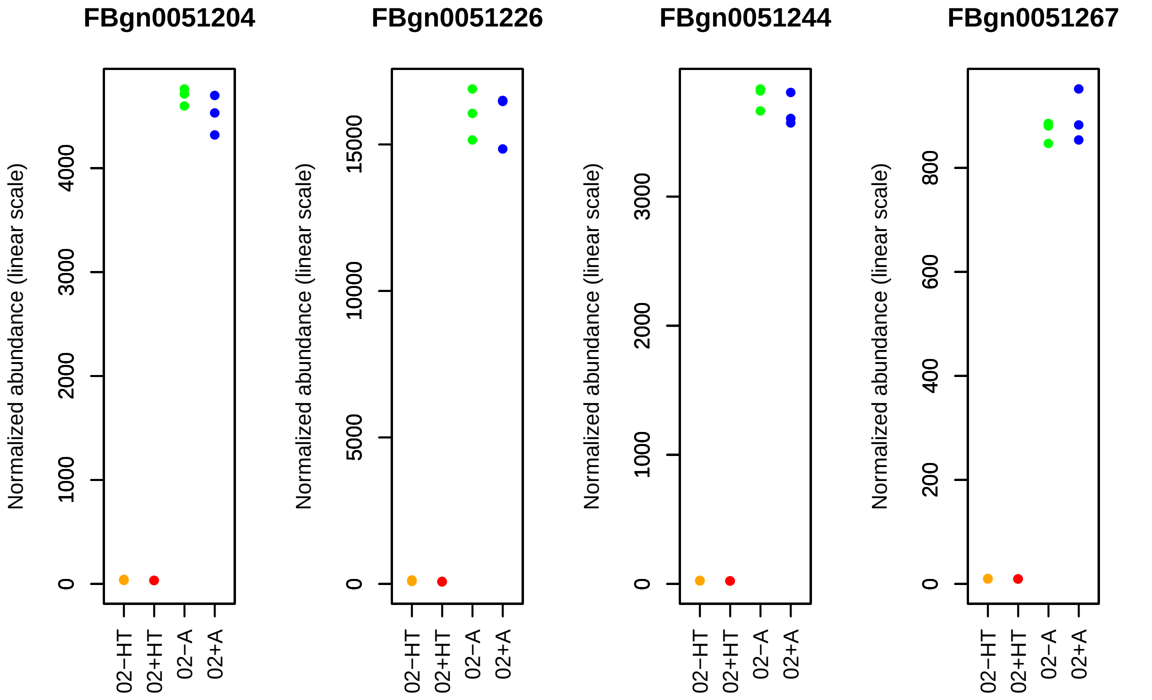
FBgn0051204
FBgn0051226
FBgn0051244
FBgn0051267
Normalized abundance (linear scale)
0
1000
2000
3000
4000
5000
10000
15000
200
400
600
800
02−HT
02+HT
02−A
02+A

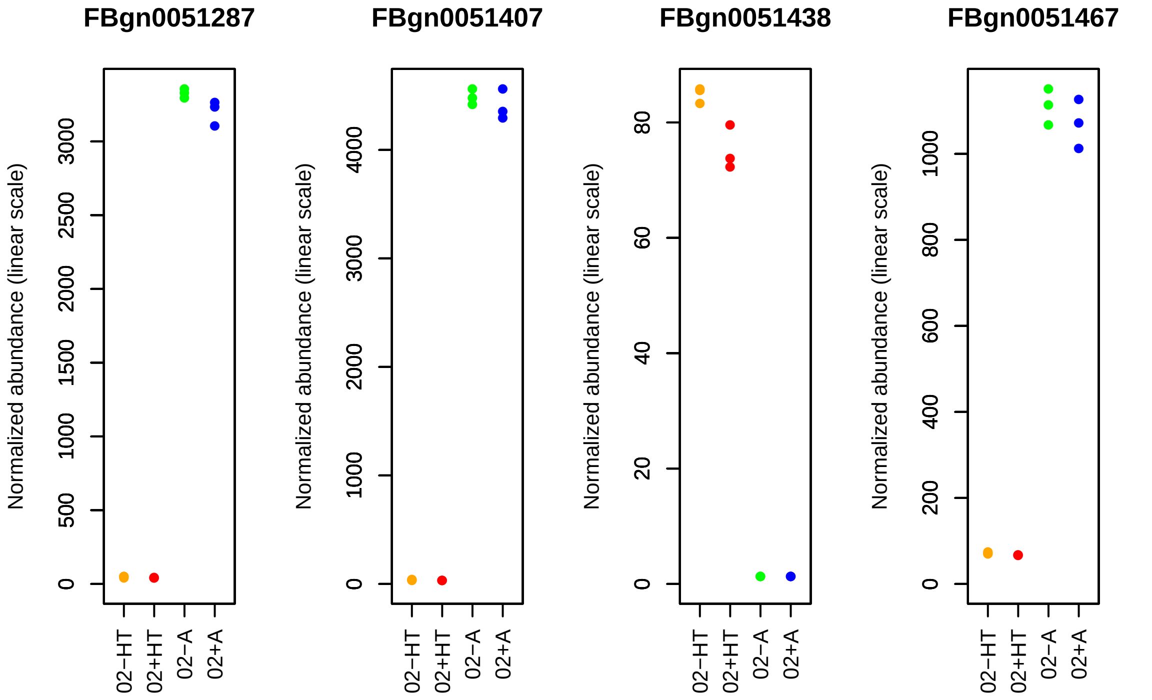

FBgn0051287
FBgn0051407
FBgn0051438
FBgn0051467
Normalized abundance (linear scale)
0
500
1000
1500
2000
2500
3000
4000
20
40
60
80
200
400
600
800
02−HT
02+HT
02−A
02+A

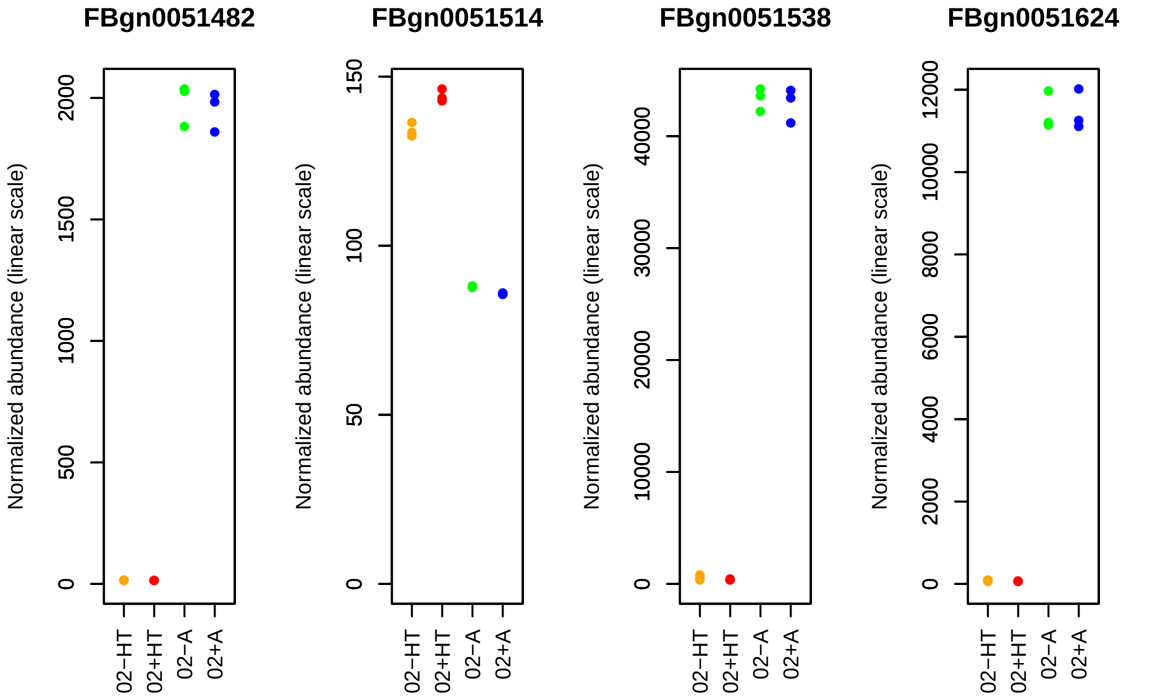

FBgn0051482
FBgn0051514
FBgn0051538
FBgn0051624
Normalized abundance (linear scale)
0
500
1000
1500
2000
50
100
150
10000
20000
30000
40000
2000
4000
6000
8000
10000
12000
02−HT
02+HT
02−A
02+A

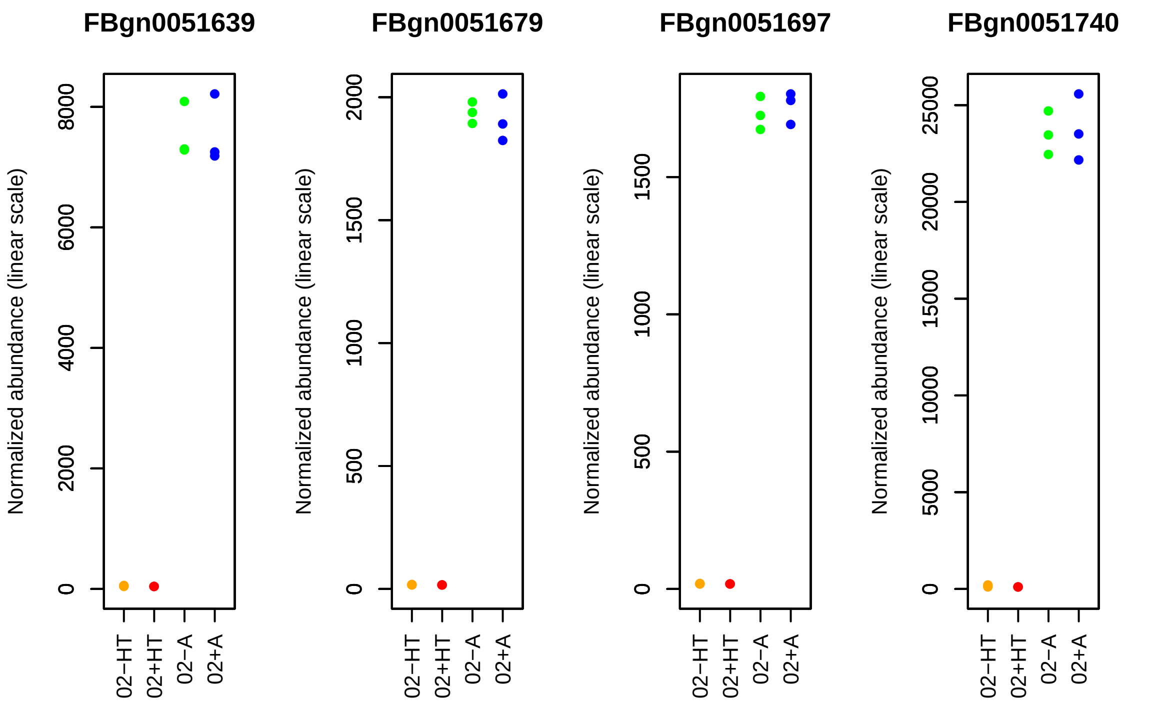
FBgn0051639
FBgn0051679
FBgn0051697
FBgn0051740
Normalized abundance (linear scale)
0
2000
4000
6000
8000
500
1000
1500
2000
5000
10000
15000
20000
25000
02−HT
02+HT
02−A
02+A

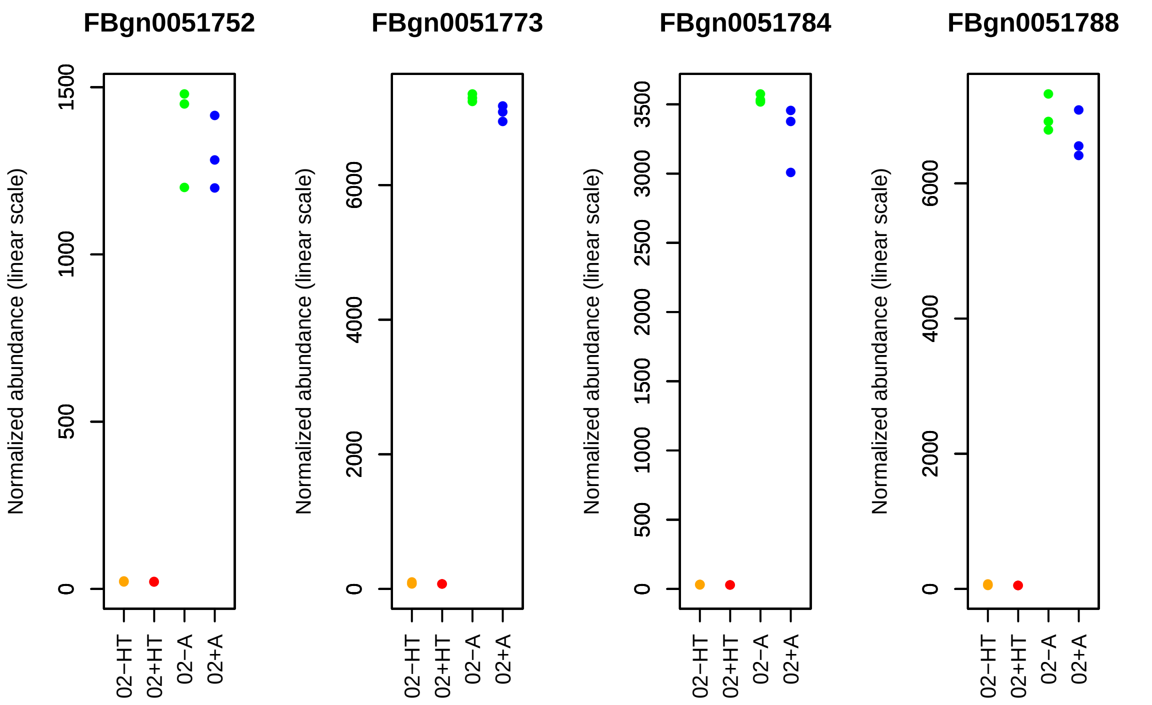

FBgn0051752
FBgn0051773
FBgn0051784
FBgn0051788
Normalized abundance (linear scale)
0
500
1000
1500
2000
2500
3000
3500
4000
6000
02−HT
02+HT
02−A
02+A

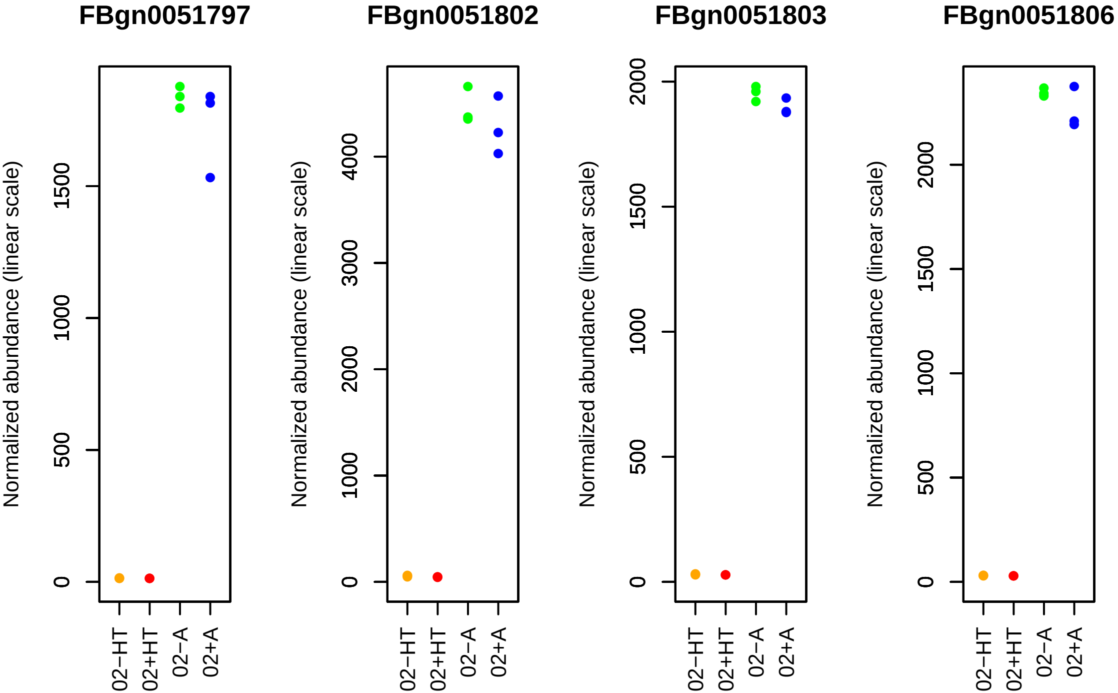

FBgn0051797
FBgn0051802
FBgn0051803
FBgn0051806
Normalized abundance (linear scale)
0
500
1000
1500
2000
3000
4000
02−HT
02+HT
02−A
02+A

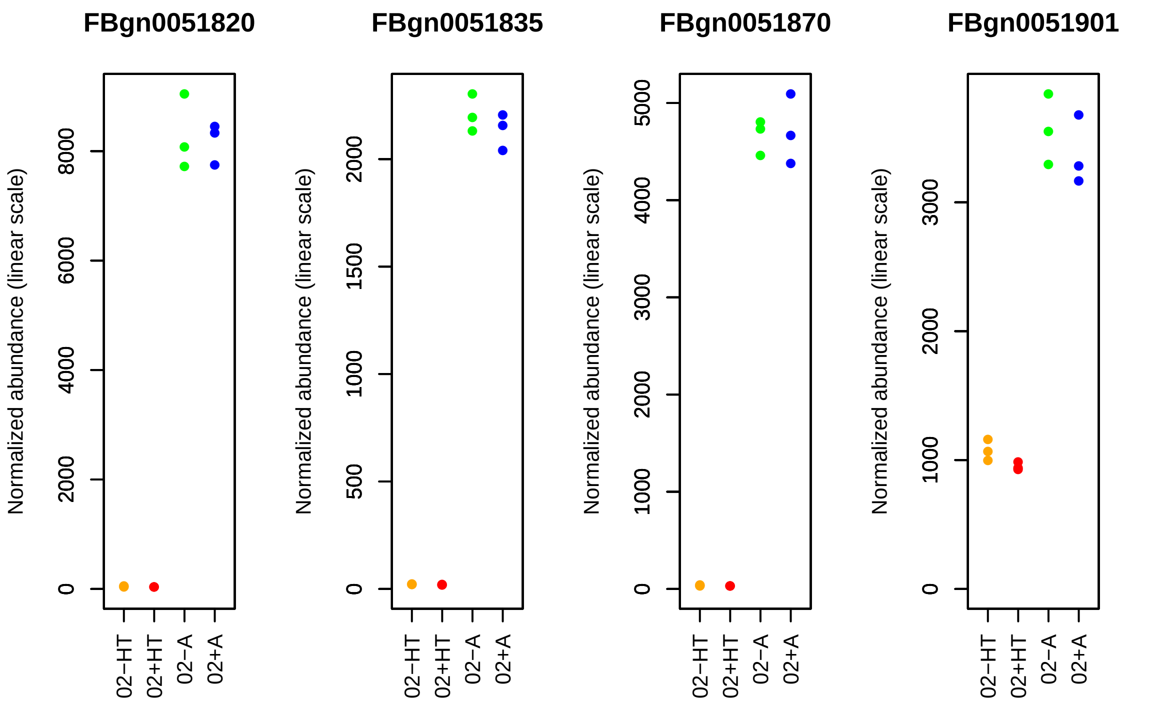
FBgn0051820
FBgn0051835
FBgn0051870
FBgn0051901
Normalized abundance (linear scale)
0
2000
4000
6000
8000
500
1000
1500
3000
5000
02−HT
02+HT
02−A
02+A

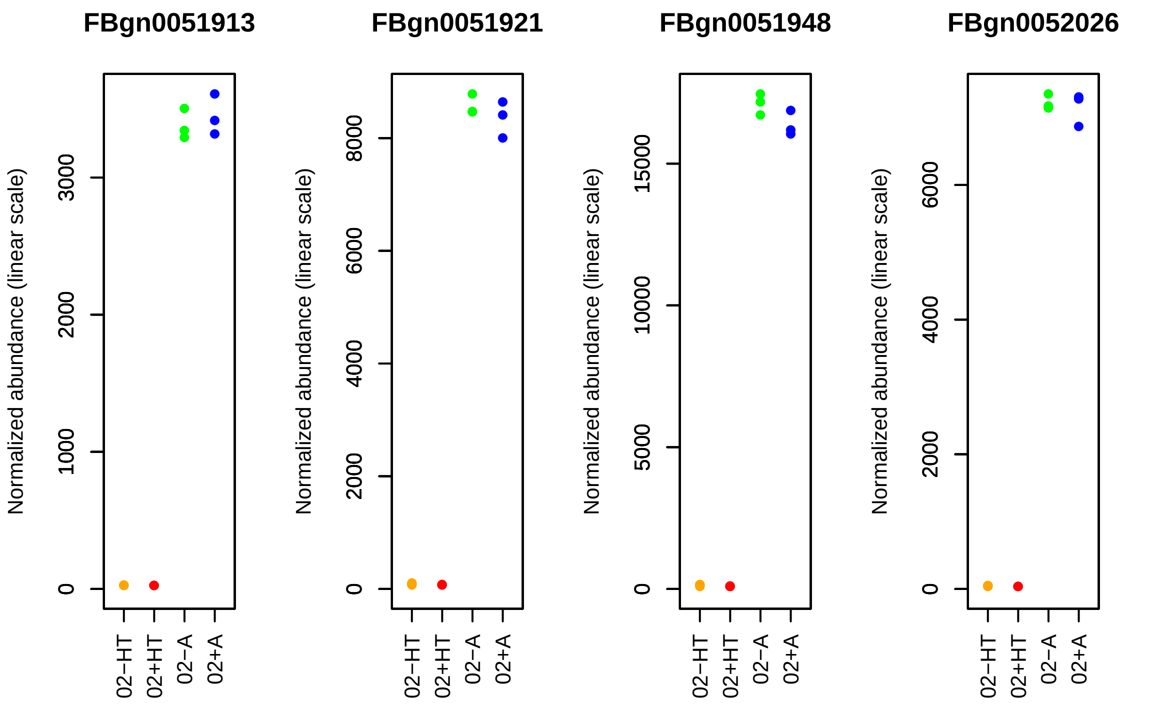

FBgn0051913
FBgn0051921
FBgn0051948
FBgn0052026
Normalized abundance (linear scale)
0
1000
2000
3000
2000
4000
6000
8000
5000
10000
15000
6000
02−HT
02+HT
02−A
02+A

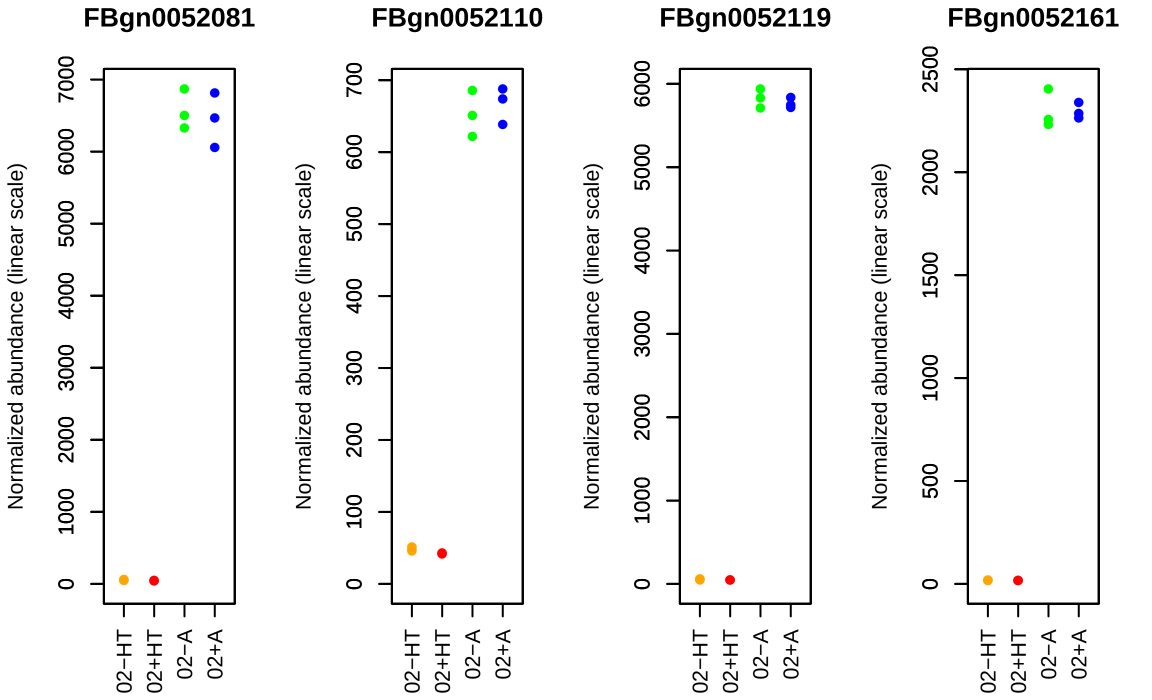
FBgn0052081
FBgn0052110
FBgn0052119
FBgn0052161
Normalized abundance (linear scale)
0
1000
2000
3000
4000
5000
6000
7000
0
100
200
300
400
500
600
700
0
500
1000
1500
2000
2500
02−HT
02+HT
02−A
02+A

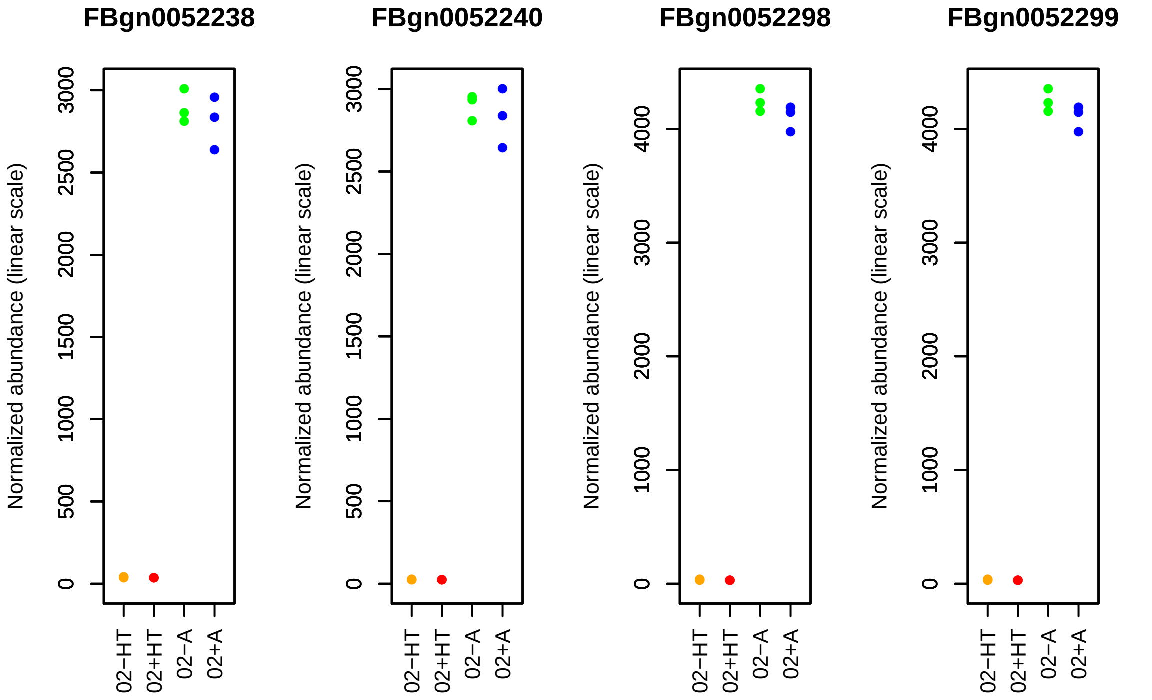
FBgn0052238
FBgn0052240
FBgn0052298
FBgn0052299
Normalized abundance (linear scale)
0
500
1000
1500
2000
2500
3000
4000
02−HT
02+HT
02−A
02+A

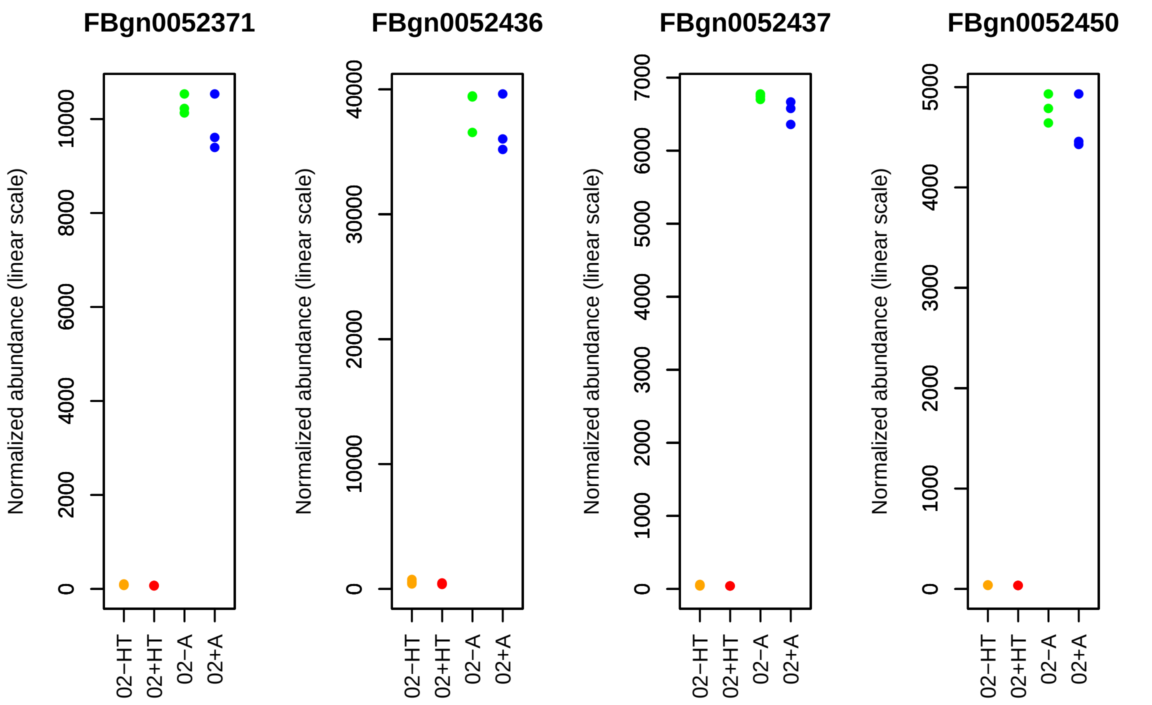
FBgn0052371
FBgn0052436
FBgn0052437
FBgn0052450
Normalized abundance (linear scale)
0
2000
4000
6000
8000
10000
10000
20000
30000
40000
1000
3000
5000
7000
02−HT
02+HT
02−A
02+A

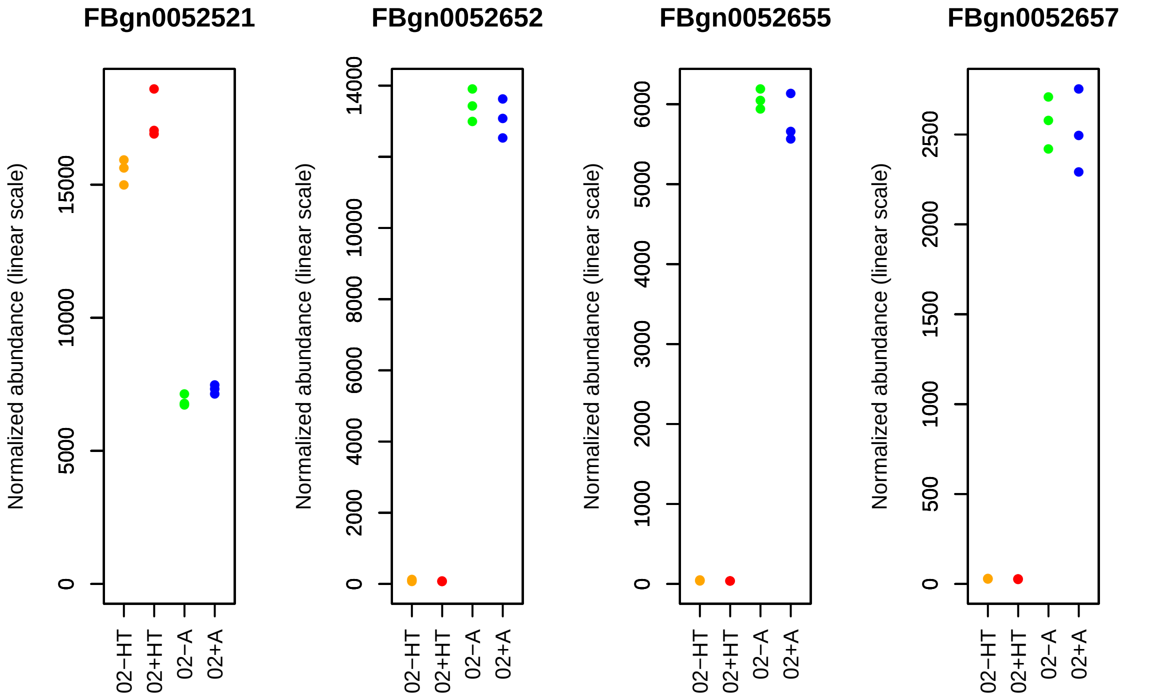

FBgn0052521
FBgn0052652
FBgn0052655
FBgn0052657
Normalized abundance (linear scale)
0
5000
10000
15000
2000
4000
6000
8000
10000
14000
1000
2000
3000
4000
5000
6000
500
1000
1500
2000
2500
02−HT
02+HT
02−A
02+A

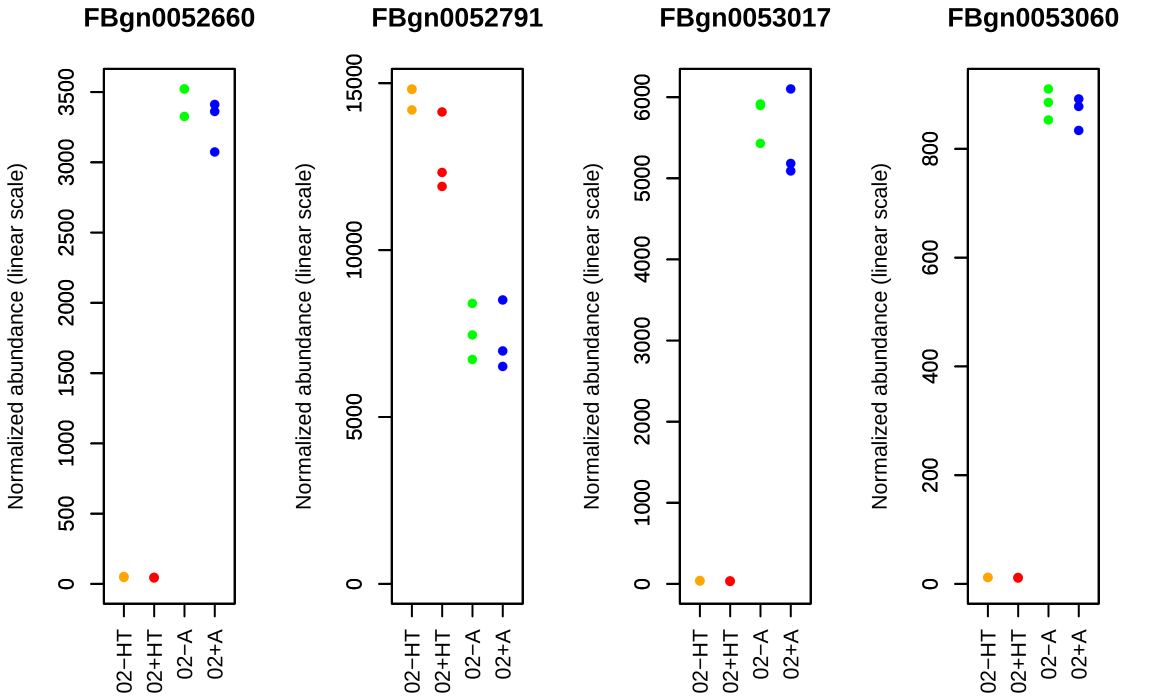

FBgn0052660
FBgn0052791
FBgn0053017
FBgn0053060
Normalized abundance (linear scale)
0
500
1000
1500
2000
2500
3000
3500
5000
10000
15000
4000
6000
200
400
600
800
02−HT
02+HT
02−A
02+A

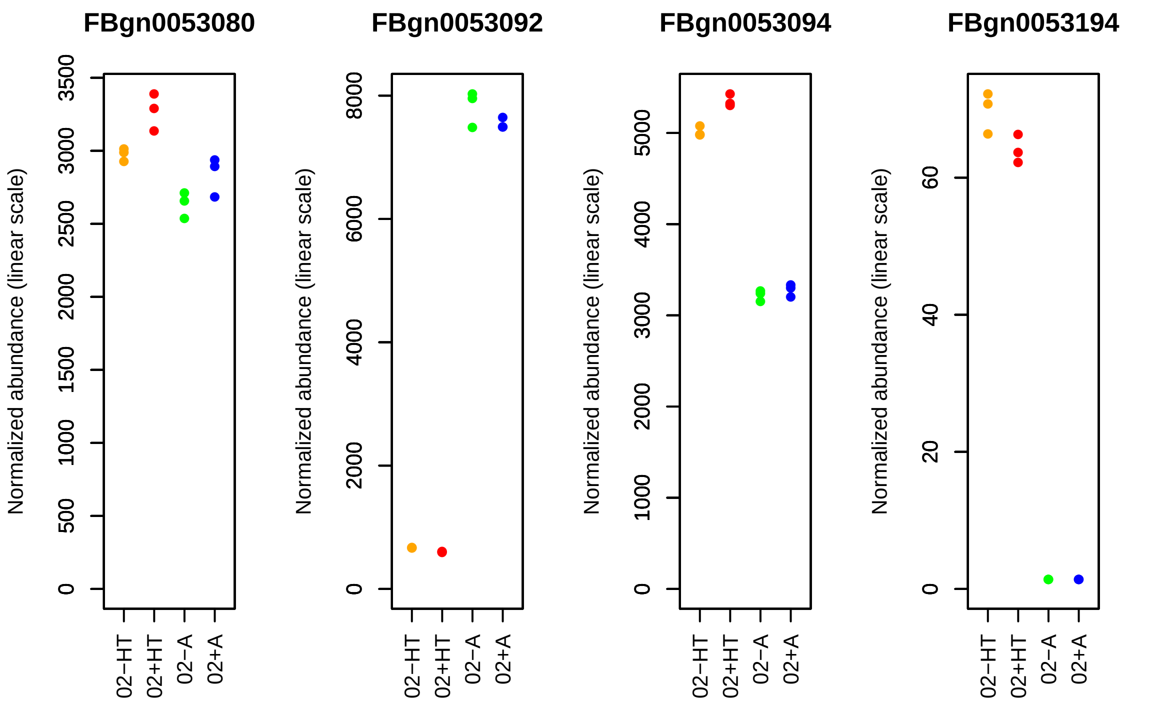

FBgn0053080
FBgn0053092
FBgn0053094
FBgn0053194
Normalized abundance (linear scale)
02−HT
02+HT
02−A
02+A

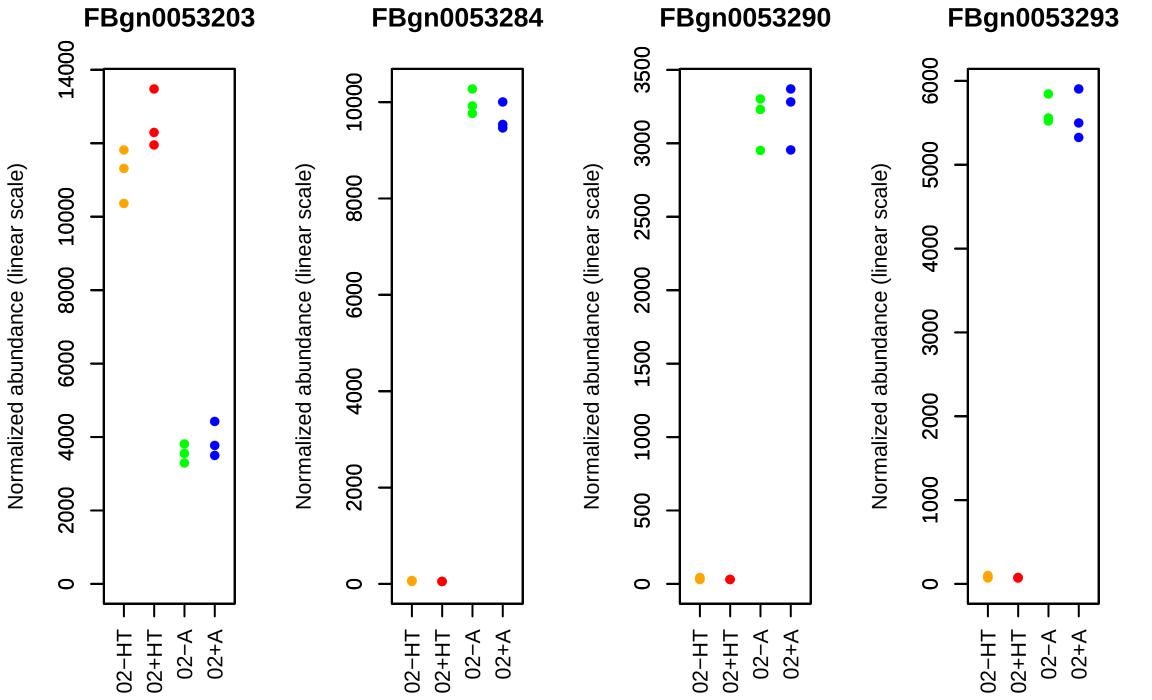
FBgn0053203
FBgn0053284
FBgn0053290
FBgn0053293
Normalized abundance (linear scale)
0
2000
4000
6000
8000
10000
14000
500
1000
1500
2000
2500
3000
3500
5000
02−HT
02+HT
02−A
02+A

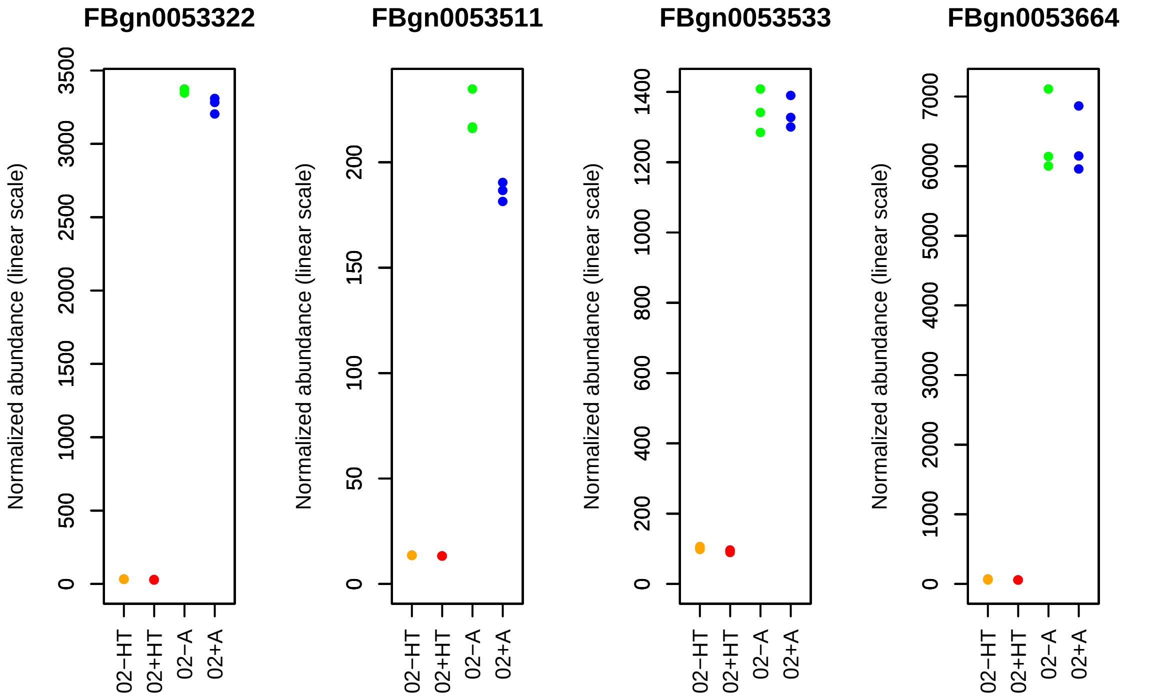
FBgn0053322
FBgn0053511
FBgn0053533
FBgn0053664
Normalized abundance (linear scale)
0
500
1000
1500
2000
2500
3000
3500
50
100
150
200
200
400
600
800
1000
1200
1400
1000
2000
3000
4000
5000
6000
7000
02−HT
02+HT
02−A
02+A

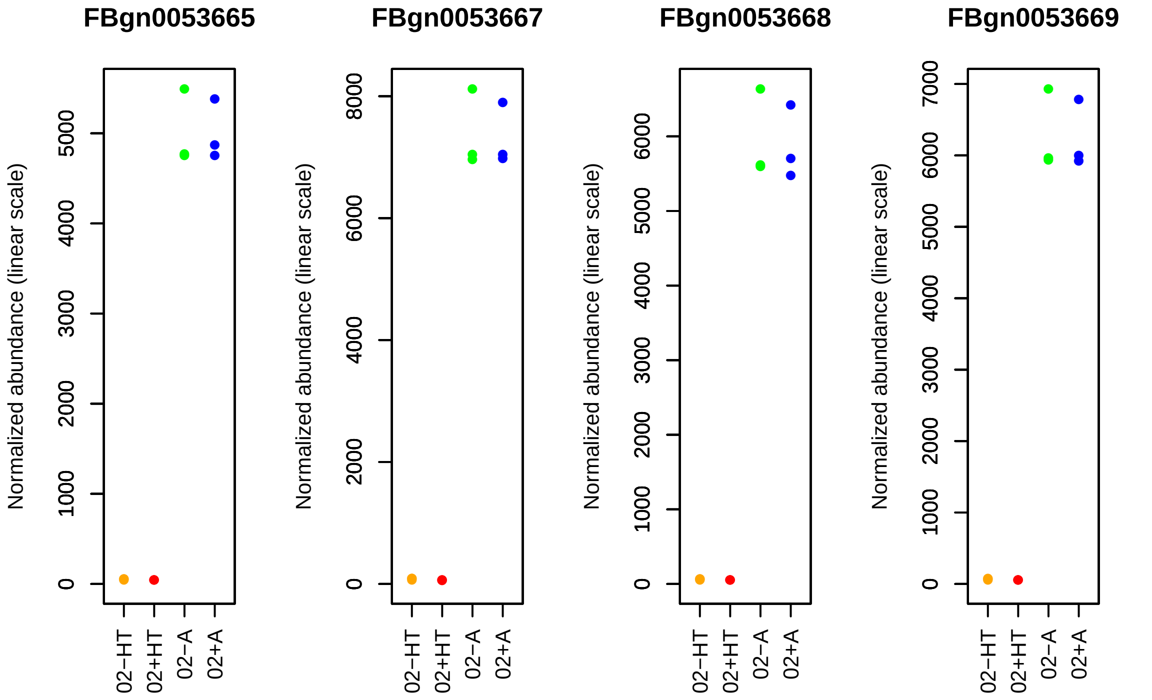

FBgn0053665
FBgn0053667
FBgn0053668
FBgn0053669
Normalized abundance (linear scale)
02−HT
02+HT
02−A
02+A

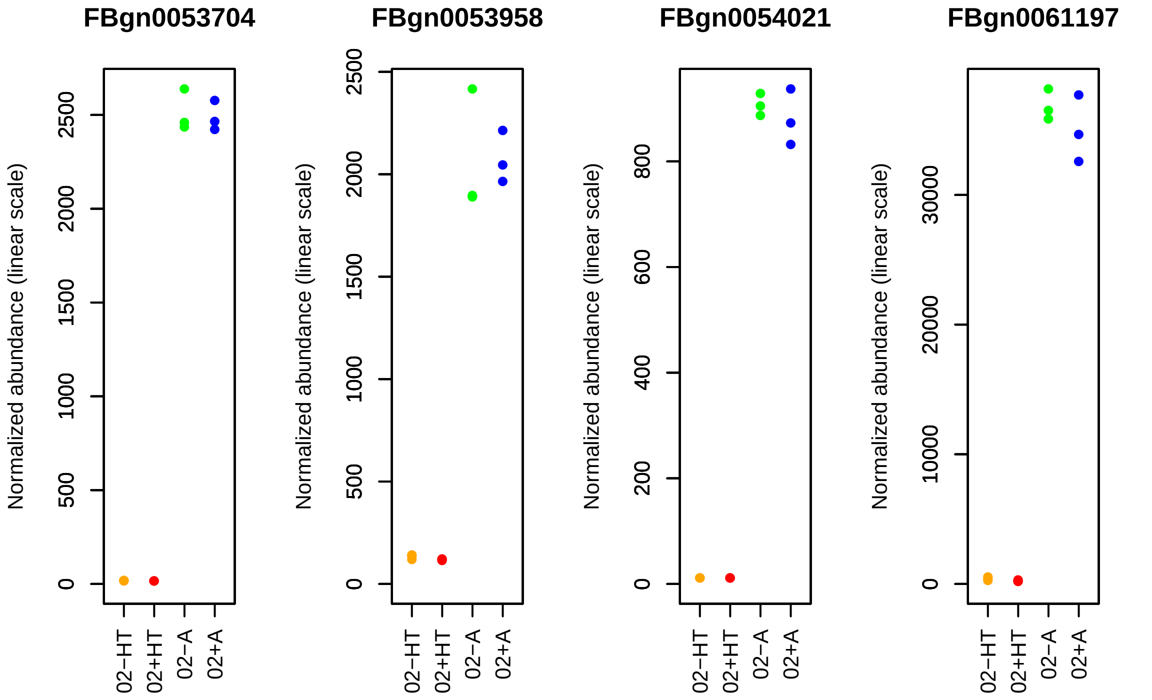

FBgn0053704
FBgn0053958
FBgn0054021
FBgn0061197
Normalized abundance (linear scale)
0
500
1000
1500
2000
2500
200
400
600
800
10000
20000
30000
02−HT
02+HT
02−A
02+A

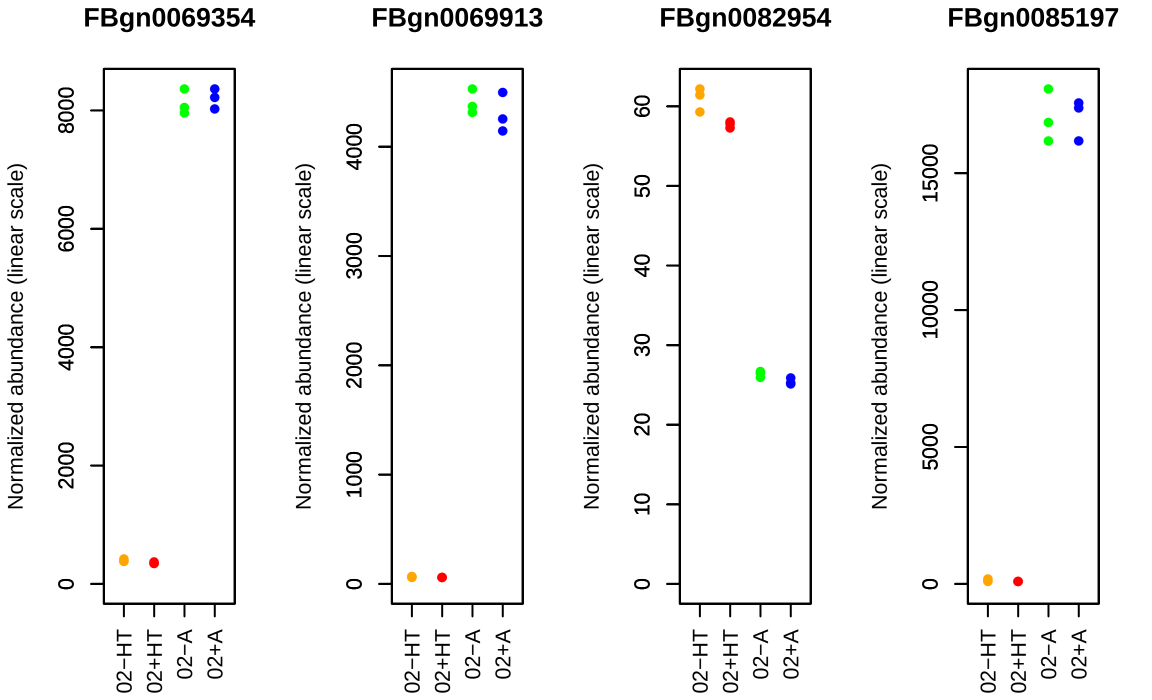

FBgn0069354
FBgn0069913
FBgn0082954
FBgn0085197
Normalized abundance (linear scale)
0
2000
4000
6000
8000
1000
3000
10
20
30
40
50
60
5000
10000
15000
02−HT
02+HT
02−A
02+A

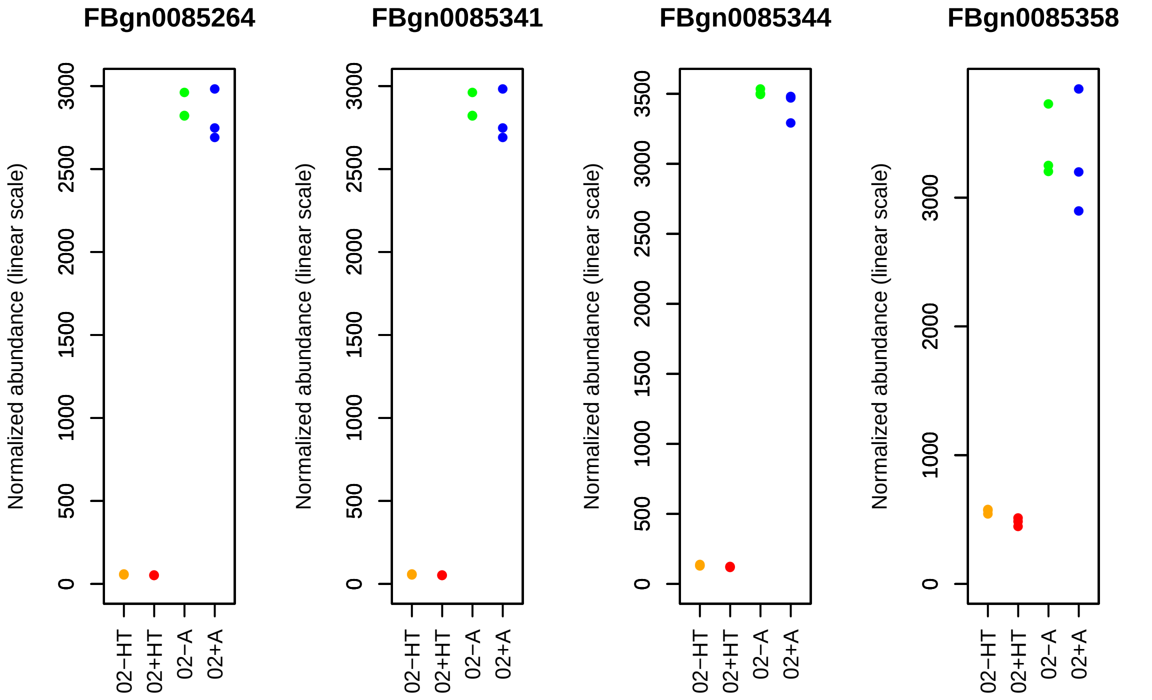
FBgn0085264
FBgn0085341
FBgn0085344
FBgn0085358
Normalized abundance (linear scale)
0
500
1000
1500
2000
2500
3000
3500
02−HT
02+HT
02−A
02+A

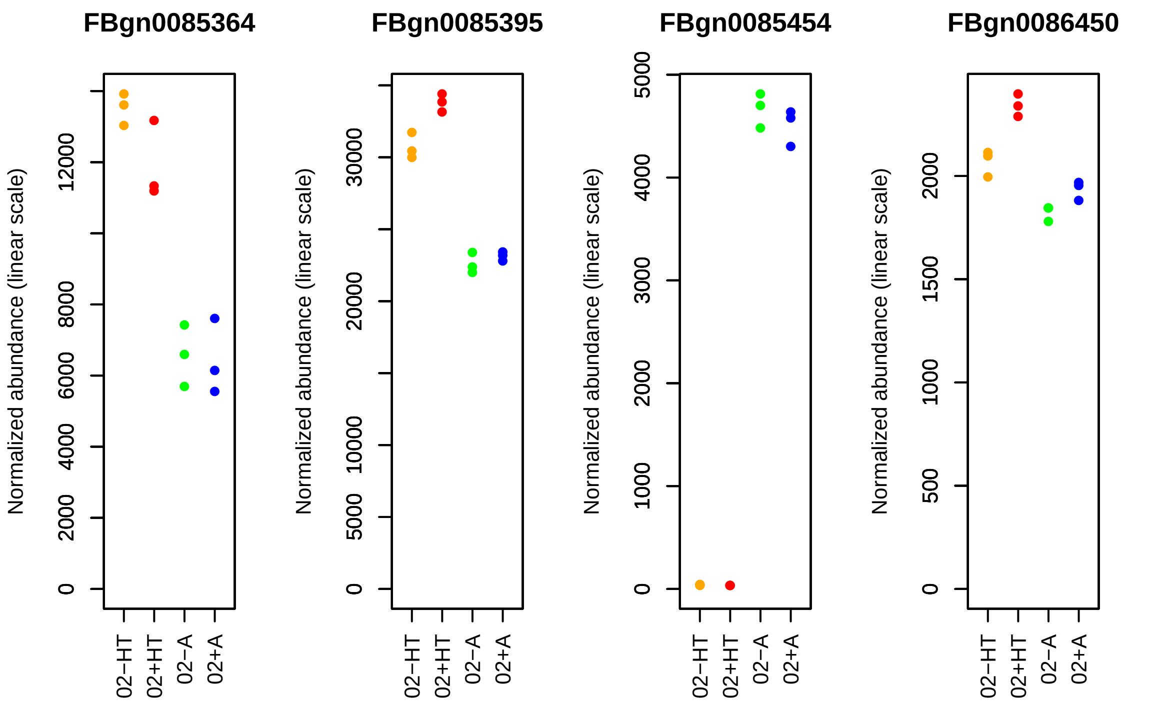

FBgn0085364
FBgn0085395
FBgn0085454
FBgn0086450
Normalized abundance (linear scale)
0
2000
4000
6000
8000
12000
0
5000
10000
20000
30000
0
1000
2000
3000
4000
5000
0
500
1000
1500
2000
02−HT
02+HT
02−A
02+A

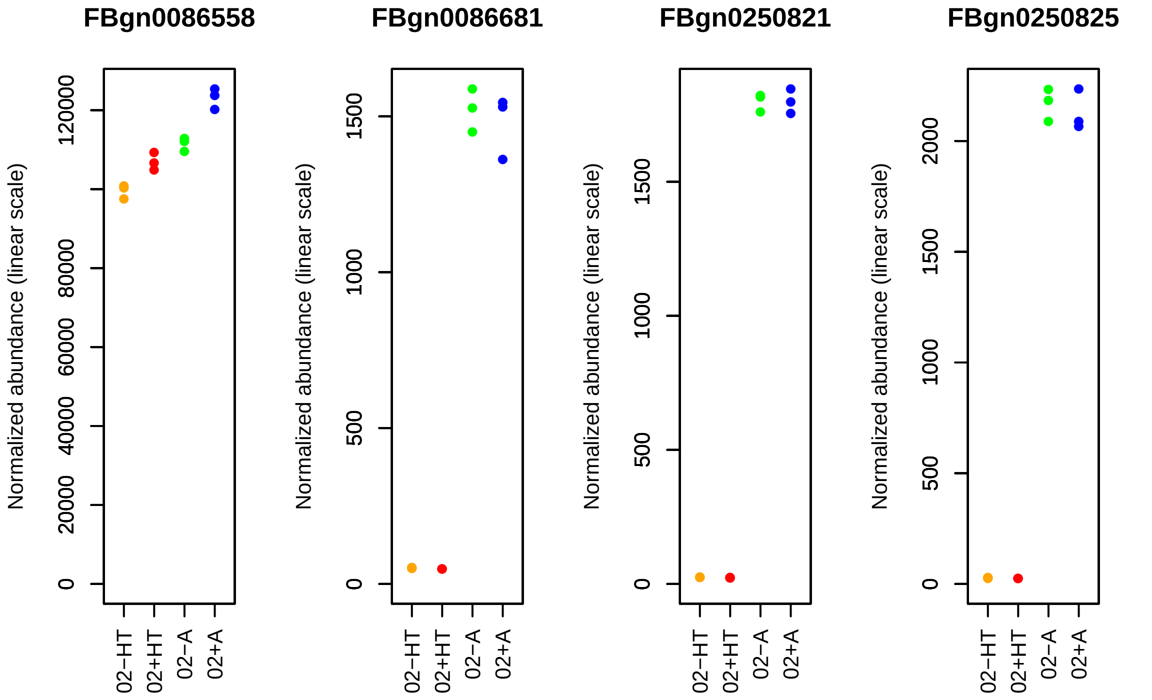

FBgn0086558
FBgn0086681
FBgn0250821
FBgn0250825
Normalized abundance (linear scale)
0
20000
40000
60000
80000
120000
500
1000
1500
2000
02−HT
02+HT
02−A
02+A

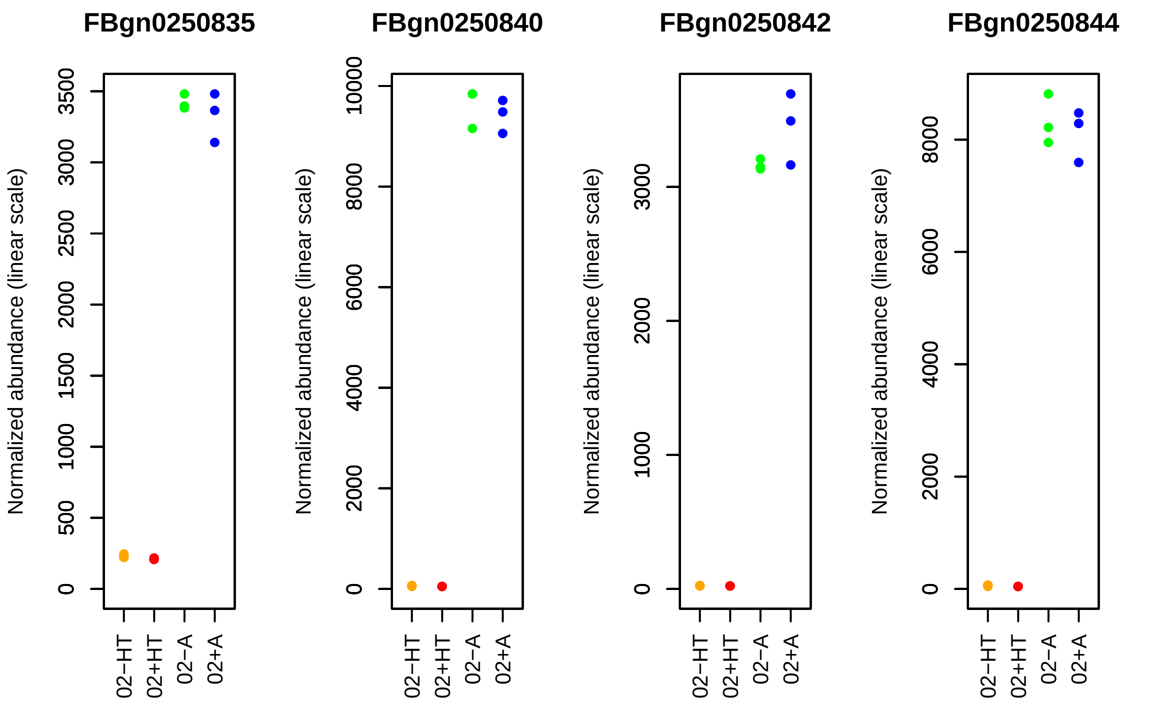
FBgn0250835
FBgn0250840
FBgn0250842
FBgn0250844
Normalized abundance (linear scale)
0
500
1000
1500
2000
2500
3000
3500
2000
4000
6000
8000
10000
02−HT
02+HT
02−A
02+A

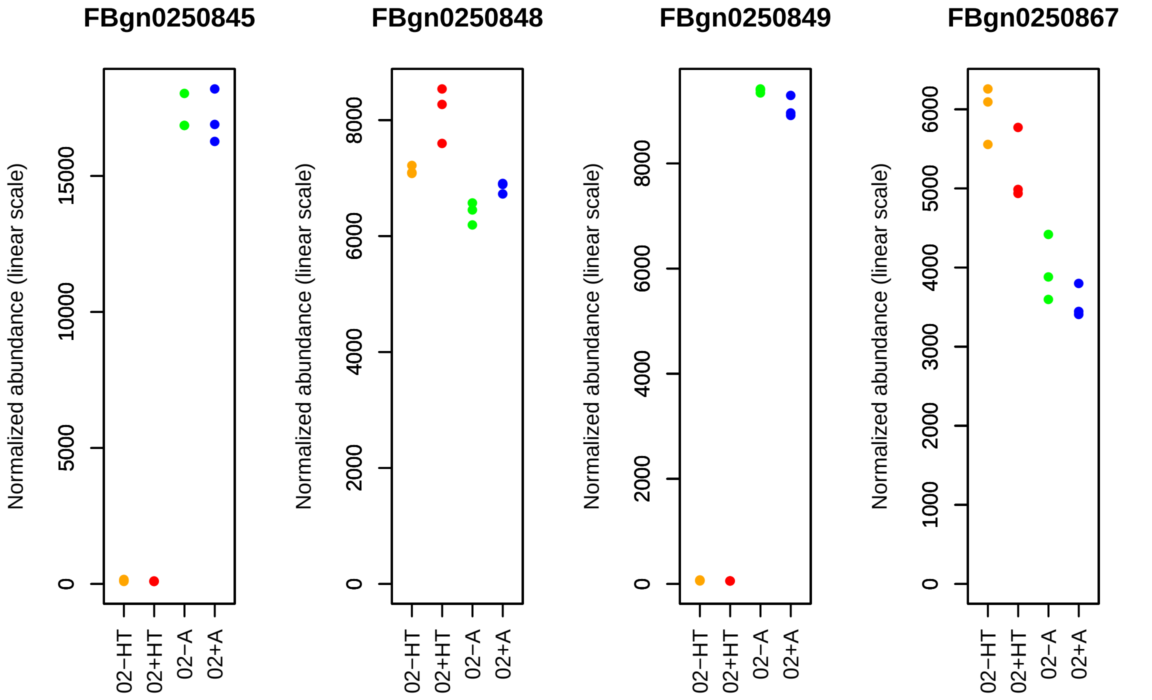

FBgn0250845
FBgn0250848
FBgn0250849
FBgn0250867
Normalized abundance (linear scale)
0
5000
10000
15000
2000
4000
6000
8000
1000
3000
5000
02−HT
02+HT
02−A
02+A

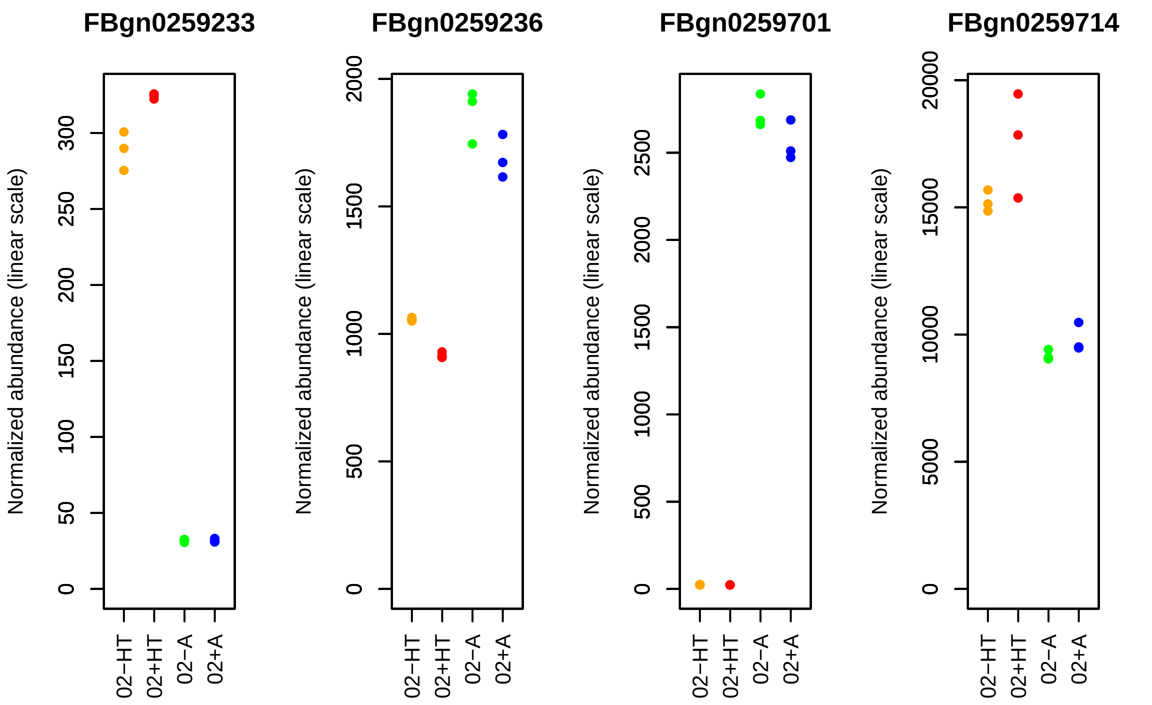
FBgn0259233
FBgn0259236
FBgn0259701
FBgn0259714
Normalized abundance (linear scale)
0
50
100
150
200
250
300
500
1000
1500
2000
2500
5000
10000
15000
20000
02−HT
02+HT
02−A
02+A

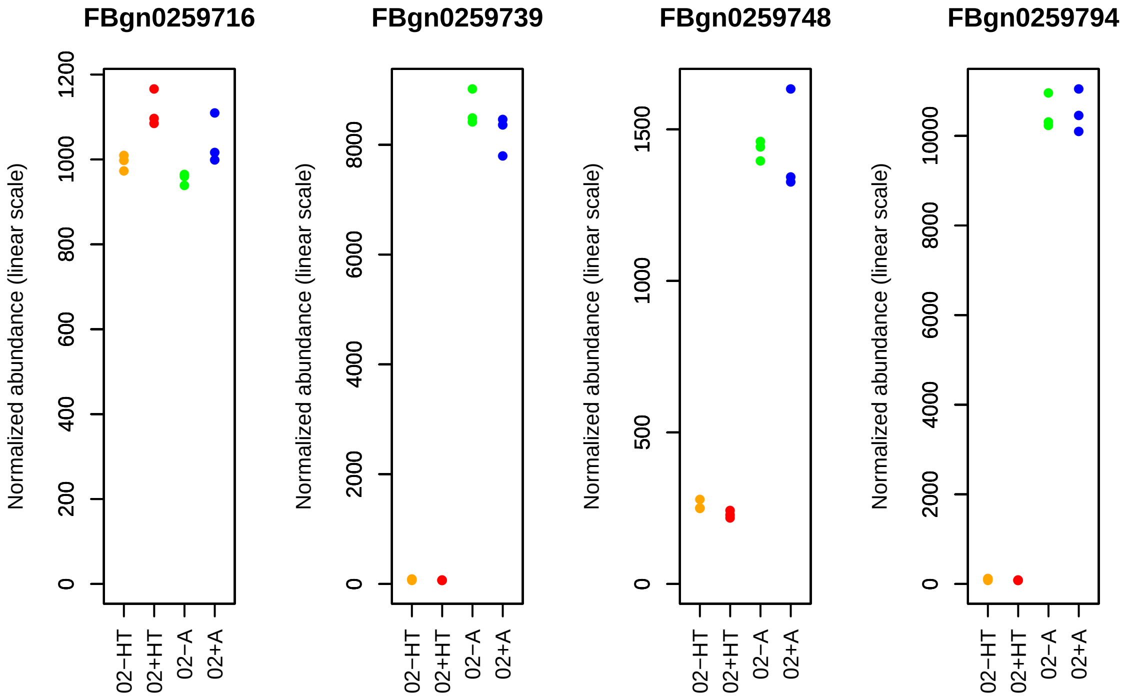

FBgn0259716
FBgn0259739
FBgn0259748
FBgn0259794
Normalized abundance (linear scale)
0
200
400
600
800
1000
1200
2000
4000
6000
8000
10000
500
1500
02−HT
02+HT
02−A
02+A

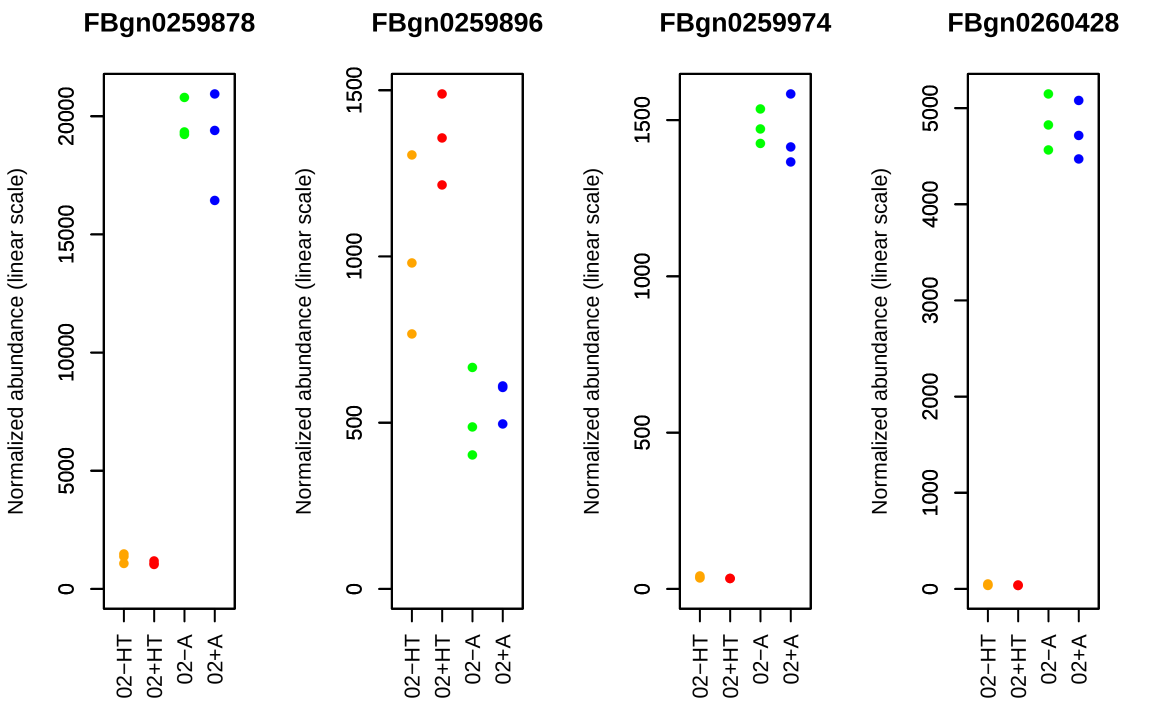

FBgn0259878
FBgn0259896
FBgn0259974
FBgn0260428
Normalized abundance (linear scale)
0
5000
10000
15000
20000
500
1000
1500
2000
3000
4000
02−HT
02+HT
02−A
02+A

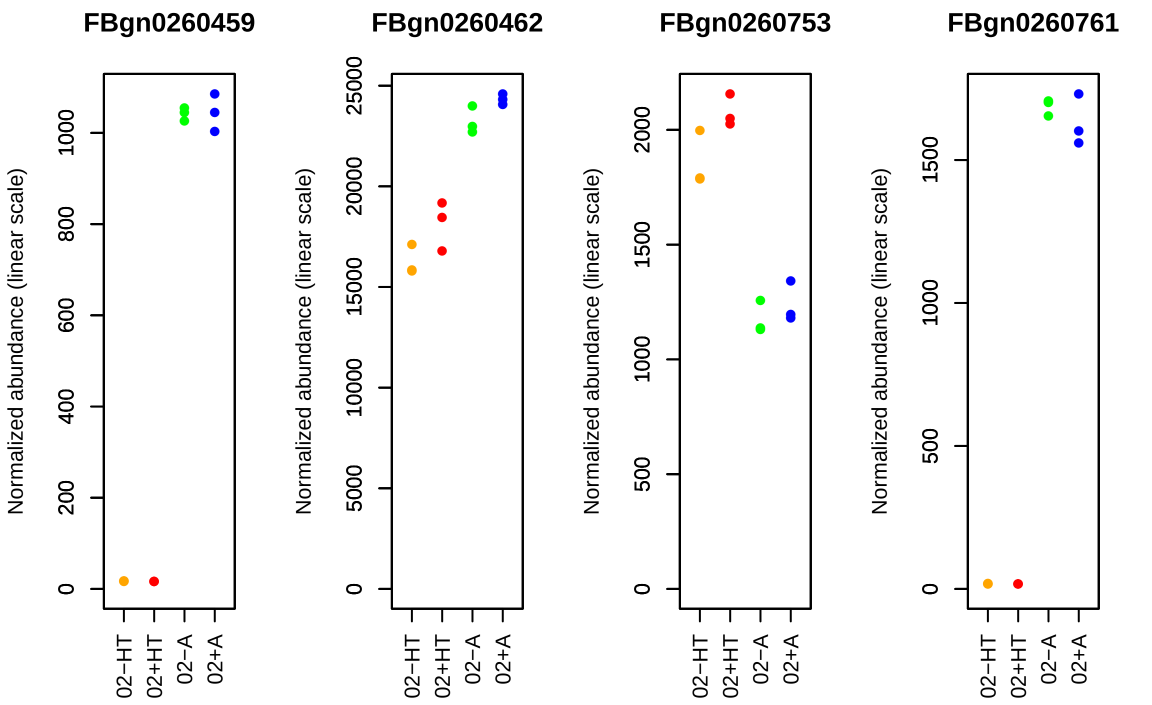
FBgn0260459
FBgn0260462
FBgn0260753
FBgn0260761
Normalized abundance (linear scale)
0
200
400
600
800
1000
0
5000
10000
15000
20000
25000
0
500
1000
1500
2000
0
500
1000
1500
02−HT
02+HT
02−A
02+A

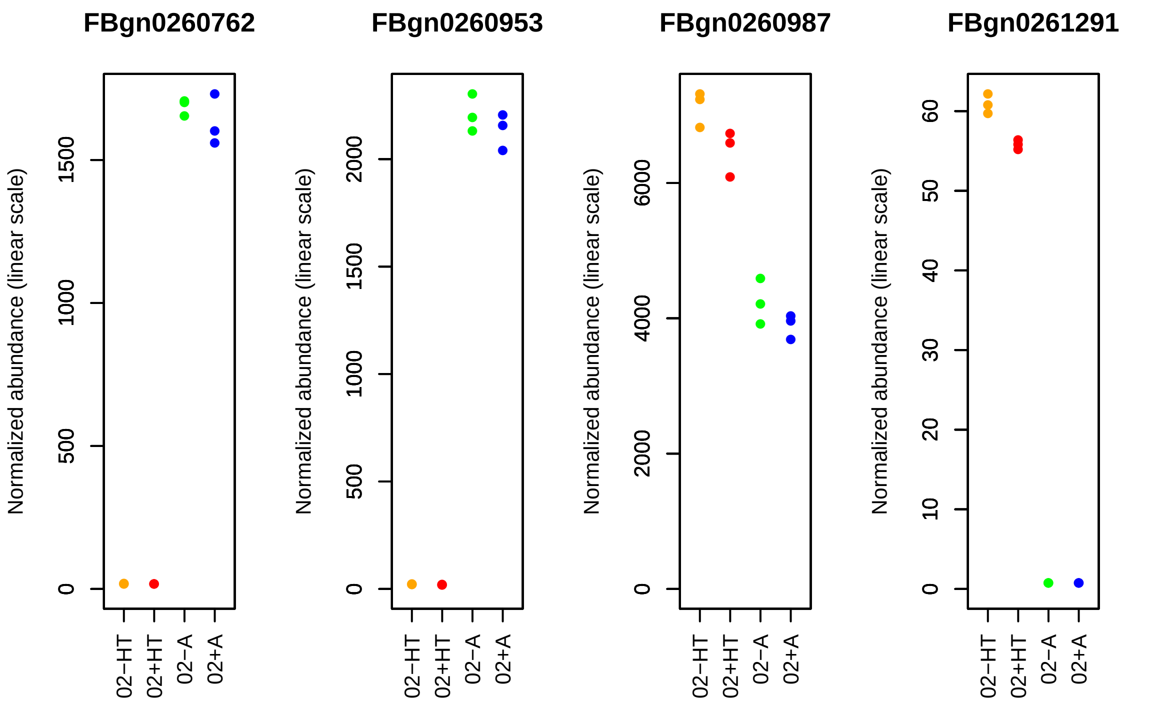

FBgn0260762
FBgn0260953
FBgn0260987
FBgn0261291
Normalized abundance (linear scale)
0
500
1000
1500
2000
4000
6000
10
20
30
40
50
60
02−HT
02+HT
02−A
02+A

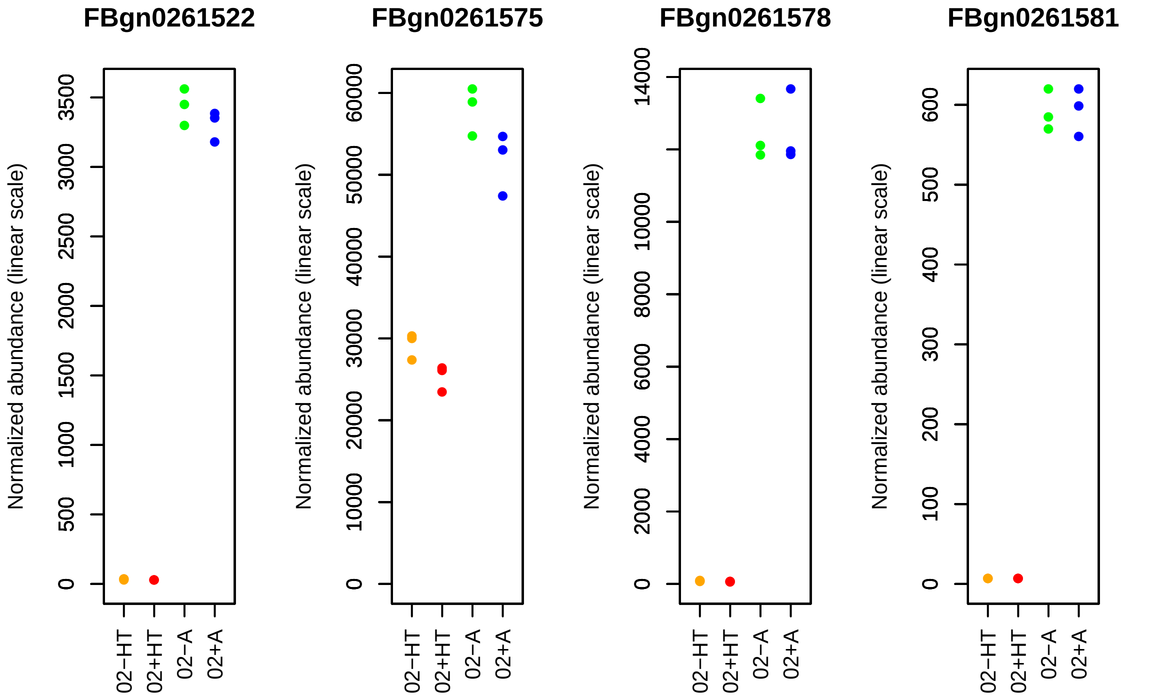

FBgn0261522
FBgn0261575
FBgn0261578
FBgn0261581
Normalized abundance (linear scale)
0
500
1000
1500
2000
2500
3000
3500
10000
20000
30000
40000
50000
60000
2000
4000
6000
8000
10000
14000
100
200
300
400
500
600
02−HT
02+HT
02−A
02+A

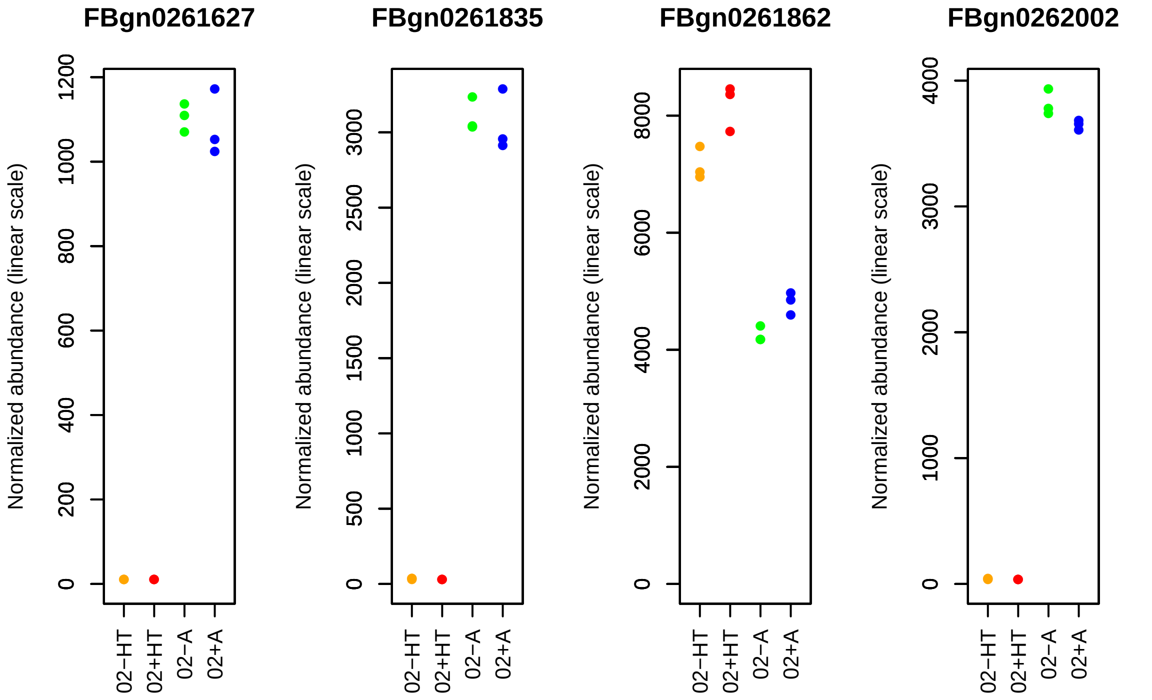

FBgn0261627
FBgn0261835
FBgn0261862
FBgn0262002
Normalized abundance (linear scale)
02−HT
02+HT
02−A
02+A

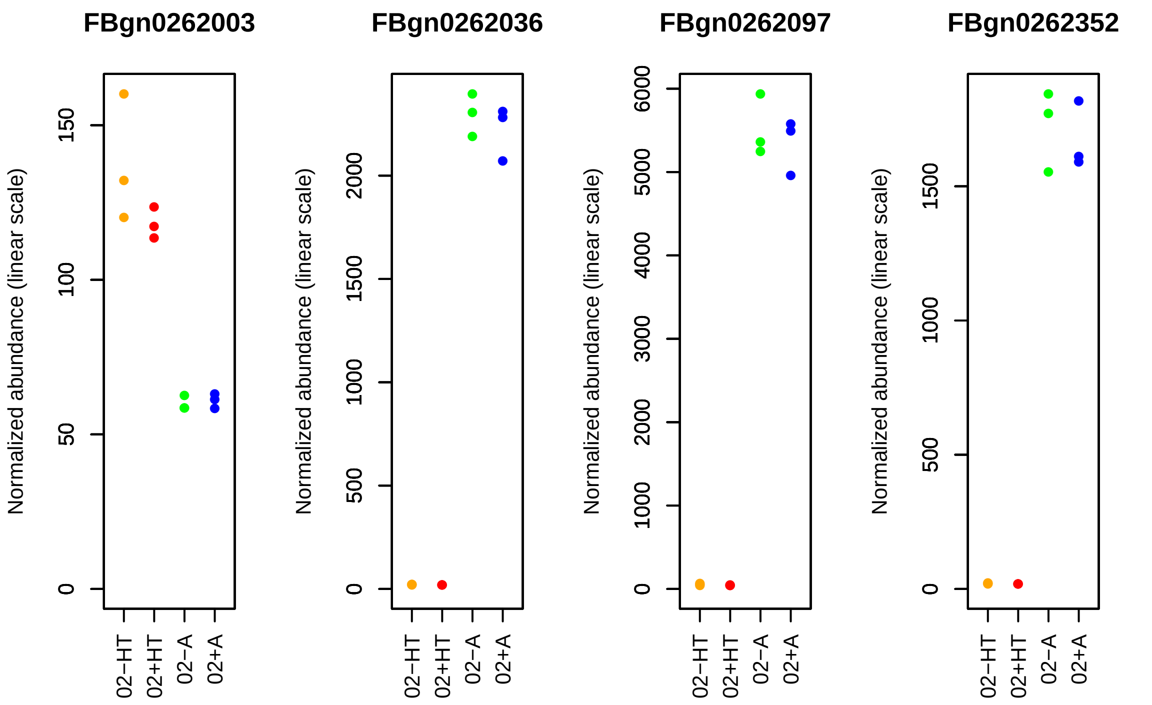
FBgn0262003
FBgn0262036
FBgn0262097
FBgn0262352
Normalized abundance (linear scale)
0
50
100
150
500
1000
1500
2000
2000
3000
4000
5000
6000
02−HT
02+HT
02−A
02+A

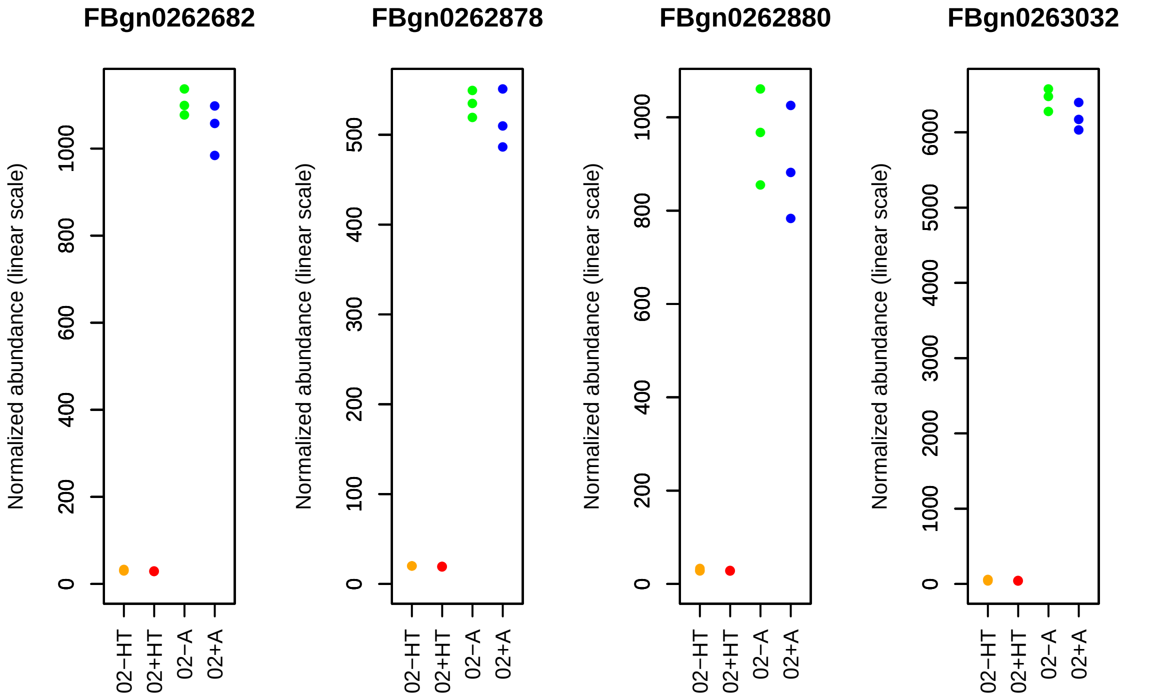
FBgn0262682
FBgn0262878
FBgn0262880
FBgn0263032
Normalized abundance (linear scale)
0
200
400
600
800
1000
0
100
200
300
400
500
0
200
400
600
800
1000
0
1000
2000
3000
4000
5000
6000
02−HT
02+HT
02−A
02+A

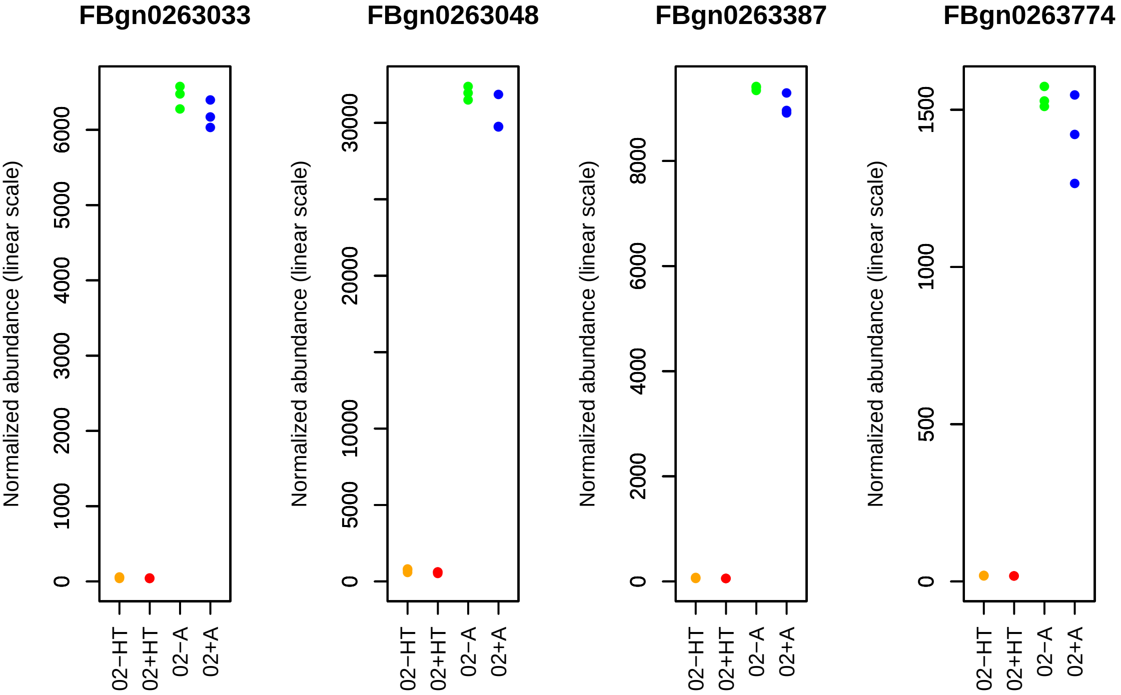
FBgn0263033
FBgn0263048
FBgn0263387
FBgn0263774
Normalized abundance (linear scale)
0
1000
2000
3000
4000
5000
6000
5000
10000
20000
30000
2000
4000
6000
8000
500
1500
02−HT
02+HT
02−A
02+A

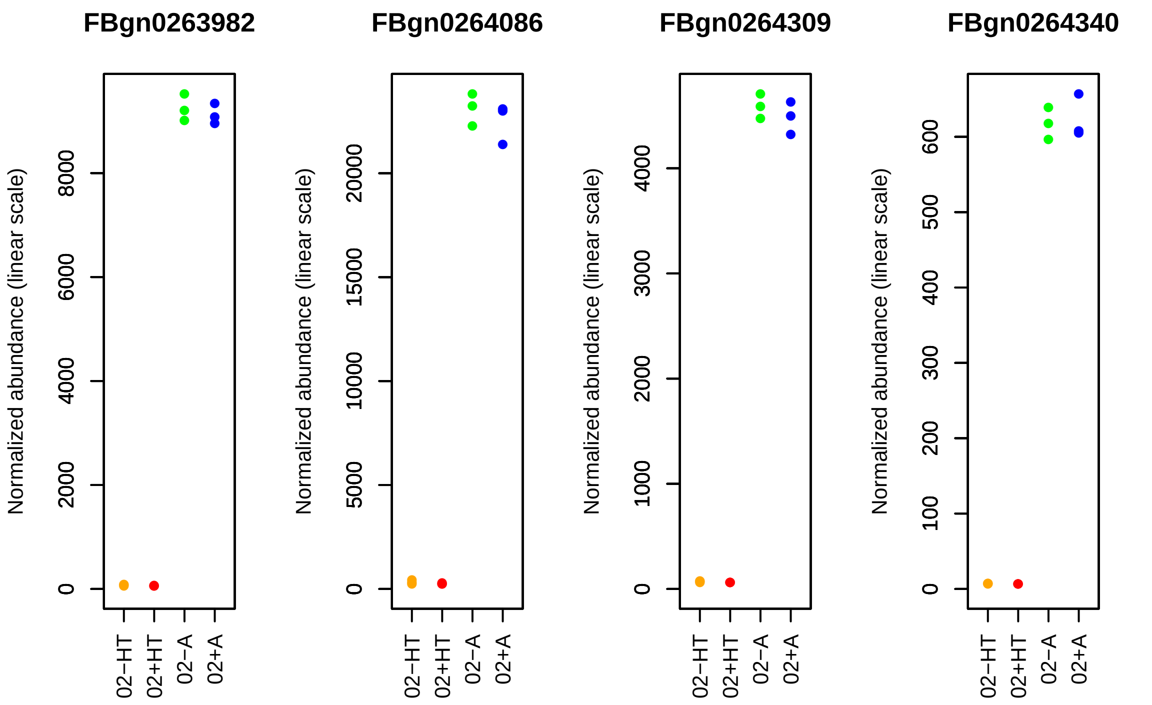

FBgn0263982
FBgn0264086
FBgn0264309
FBgn0264340
Normalized abundance (linear scale)
0
2000
4000
6000
8000
5000
10000
15000
20000
1000
3000
100
200
300
400
500
600
02−HT
02+HT
02−A
02+A

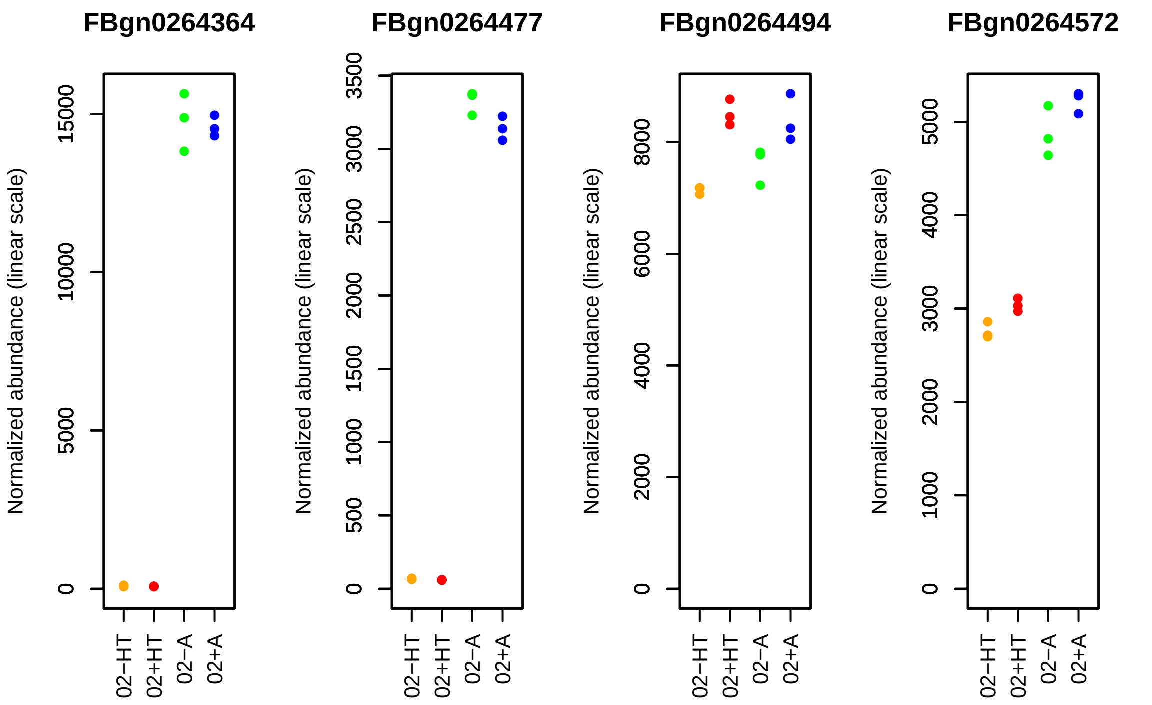
FBgn0264364
FBgn0264477
FBgn0264494
FBgn0264572
Normalized abundance (linear scale)
0
5000
10000
15000
500
1000
1500
2000
2500
3000
3500
2000
4000
6000
8000
1000
2000
3000
4000
5000
02−HT
02+HT
02−A
02+A

Fig S7C

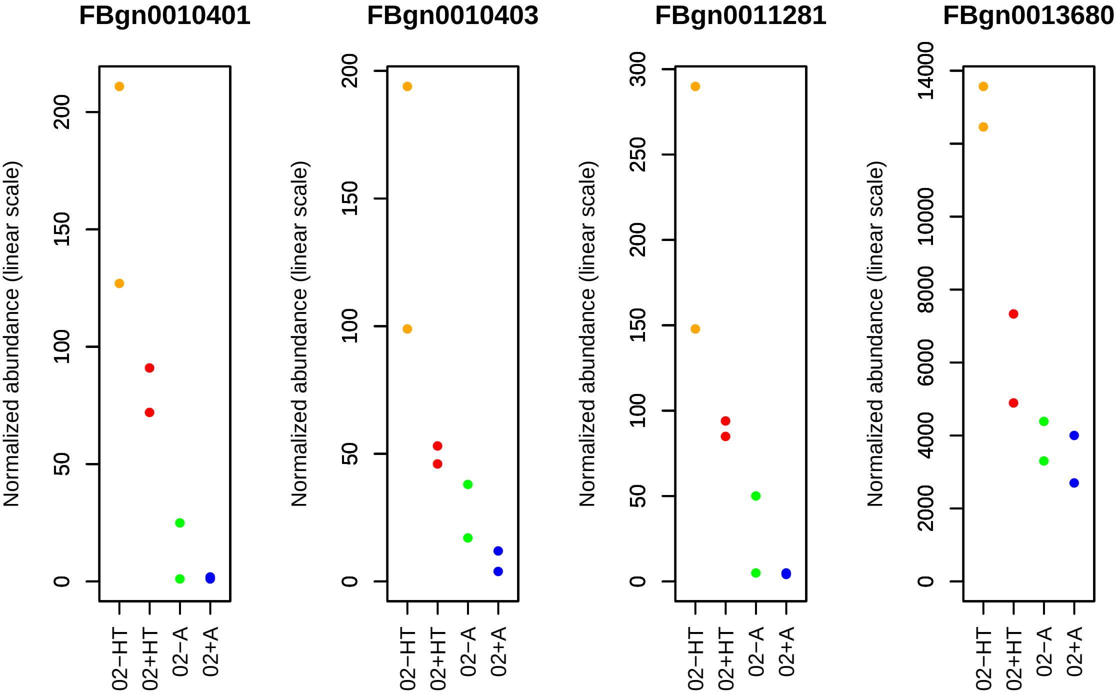

FBgn0010401
FBgn0010403
FBgn0011281
FBgn0013680
Normalized abundance (linear scale)
0
50
100
150
200
250
300
2000
4000
6000
8000
10000
14000
02−HT
02+HT
02−A
02+A

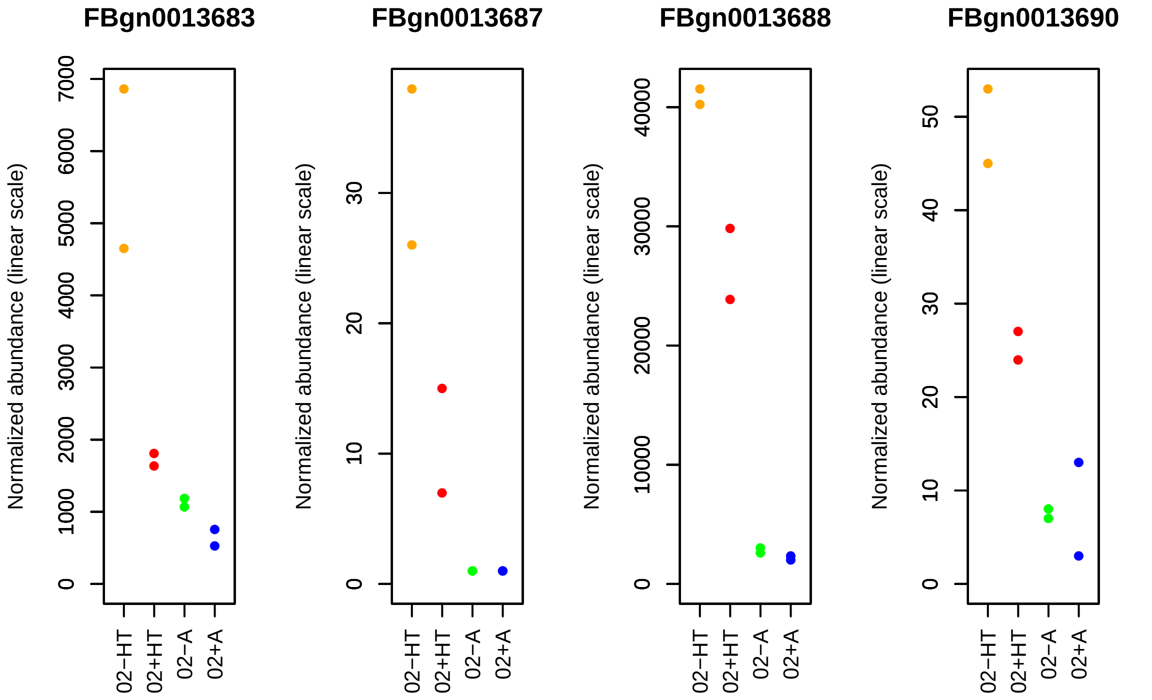

FBgn0013683
FBgn0013687
FBgn0013688
FBgn0013690
Normalized abundance (linear scale)
0
1000
2000
3000
4000
5000
6000
7000
10
20
30
10000
20000
30000
40000
40
50
02−HT
02+HT
02−A
02+A

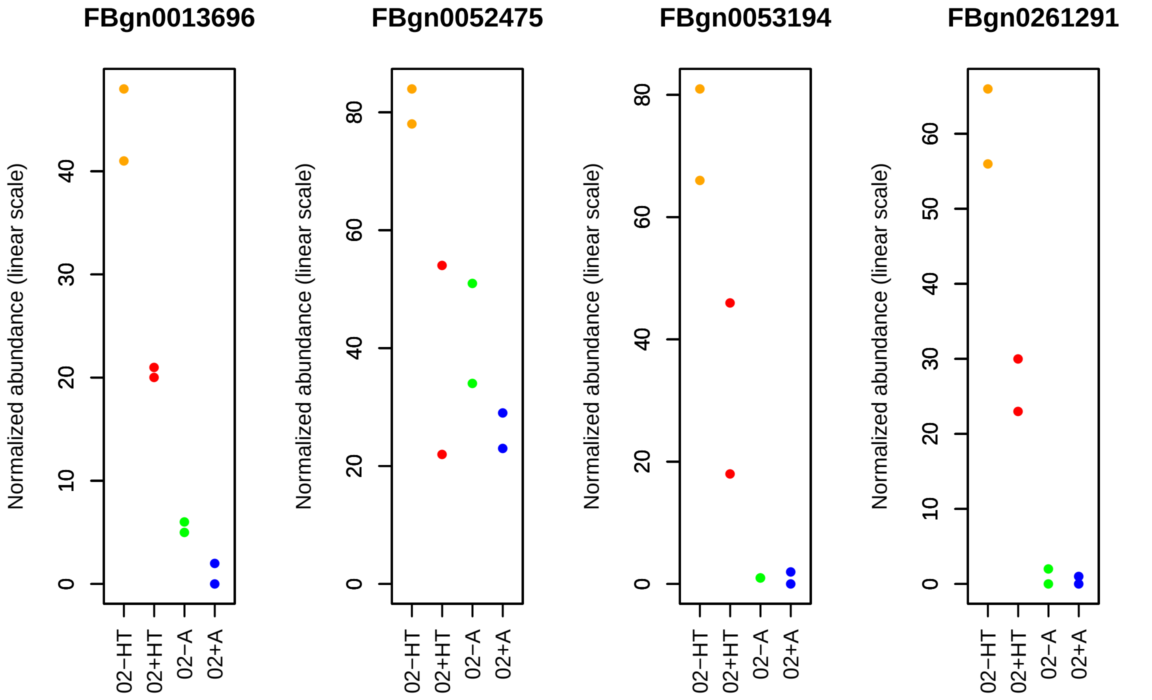

FBgn0013696
FBgn0052475
FBgn0053194
FBgn0261291
Normalized abundance (linear scale)
0
10
20
30
40
0
20
40
60
80
0
20
40
60
80
0
10
20
30
40
50
60
02−HT
02+HT
02−A
02+A

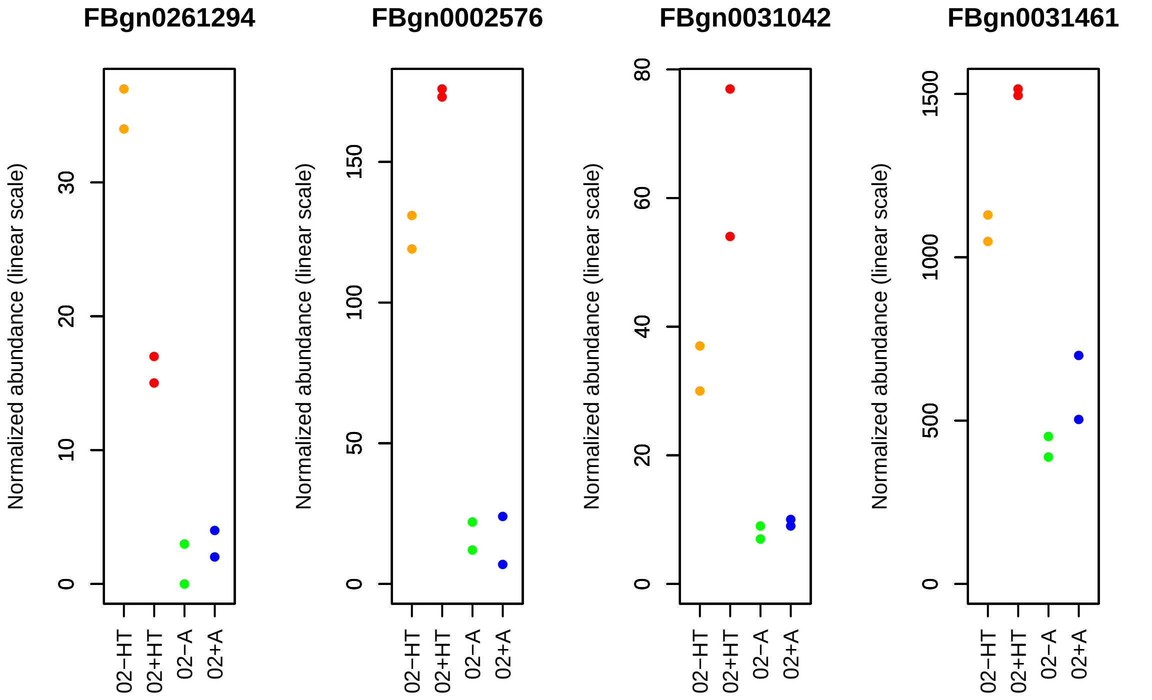
FBgn0261294
FBgn0002576
FBgn0031042
FBgn0031461
Normalized abundance (linear scale)
0
10
20
30
0
50
100
150
0
20
40
60
80
0
500
1000
1500
02−HT
02+HT
02−A
02+A

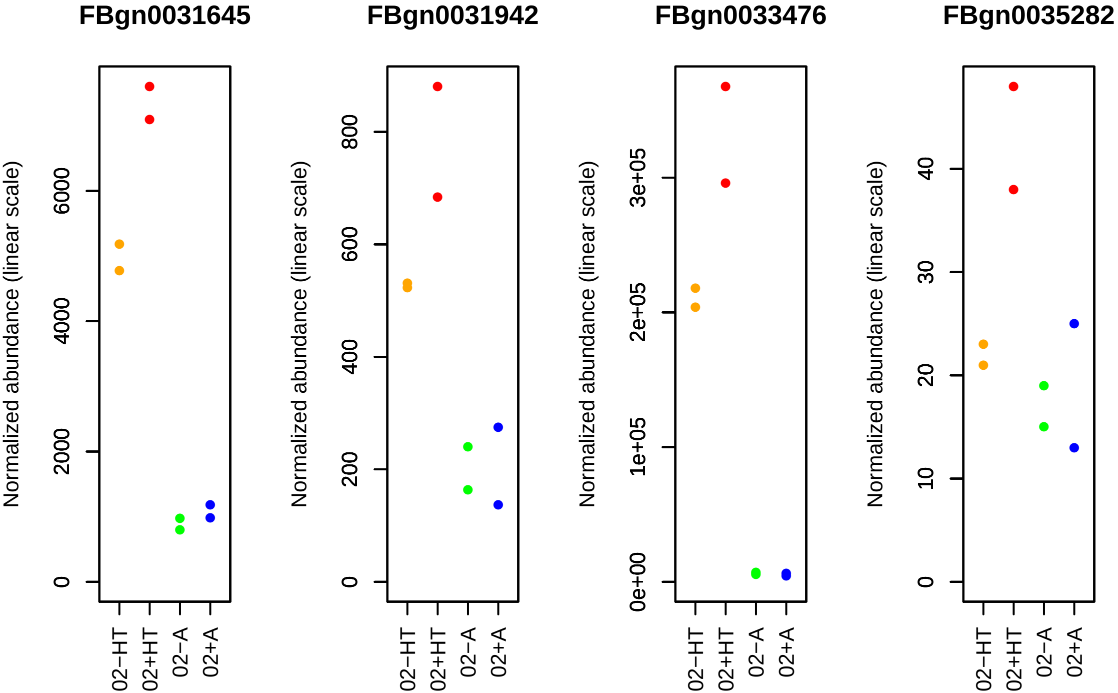
FBgn0031645
FBgn0031942
FBgn0033476
FBgn0035282
Normalized abundance (linear scale)
0
2000
4000
6000
200
400
600
800
0e+00
1e+05
2e+05
3e+05
10
20
30
40
02−HT
02+HT
02−A
02+A

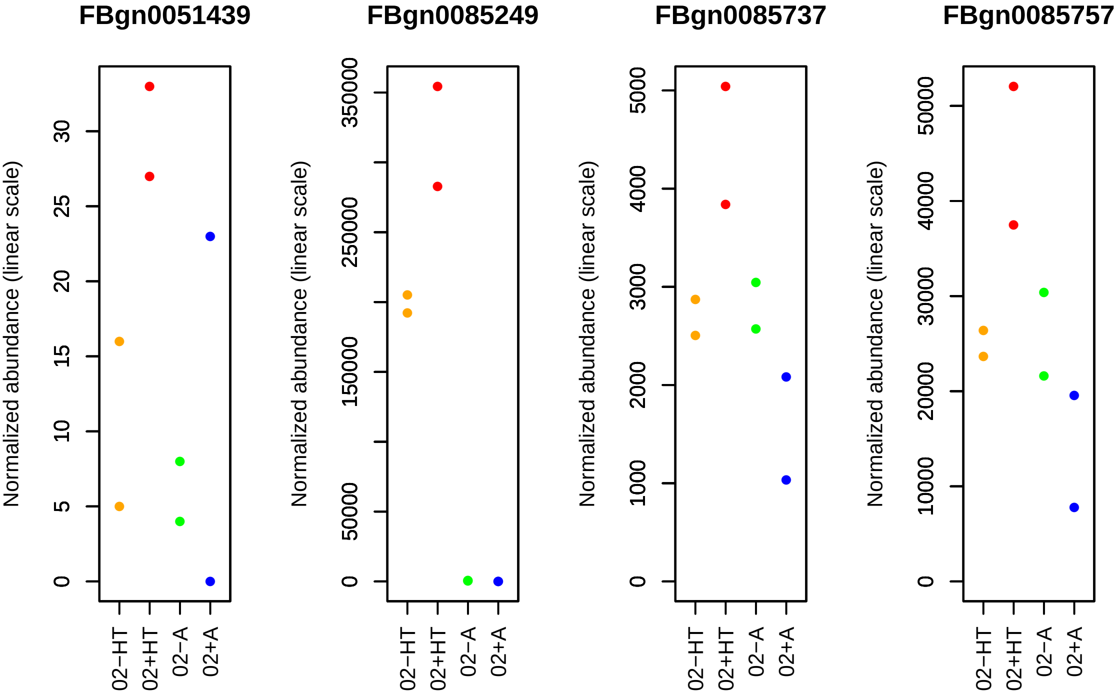
FBgn0051439
FBgn0085249
FBgn0085737
FBgn0085757
Normalized abundance (linear scale)
0
5
10
15
20
25
30
50000
150000
250000
350000
1000
2000
3000
4000
5000
10000
20000
30000
40000
50000
02−HT
02+HT
02−A
02+A

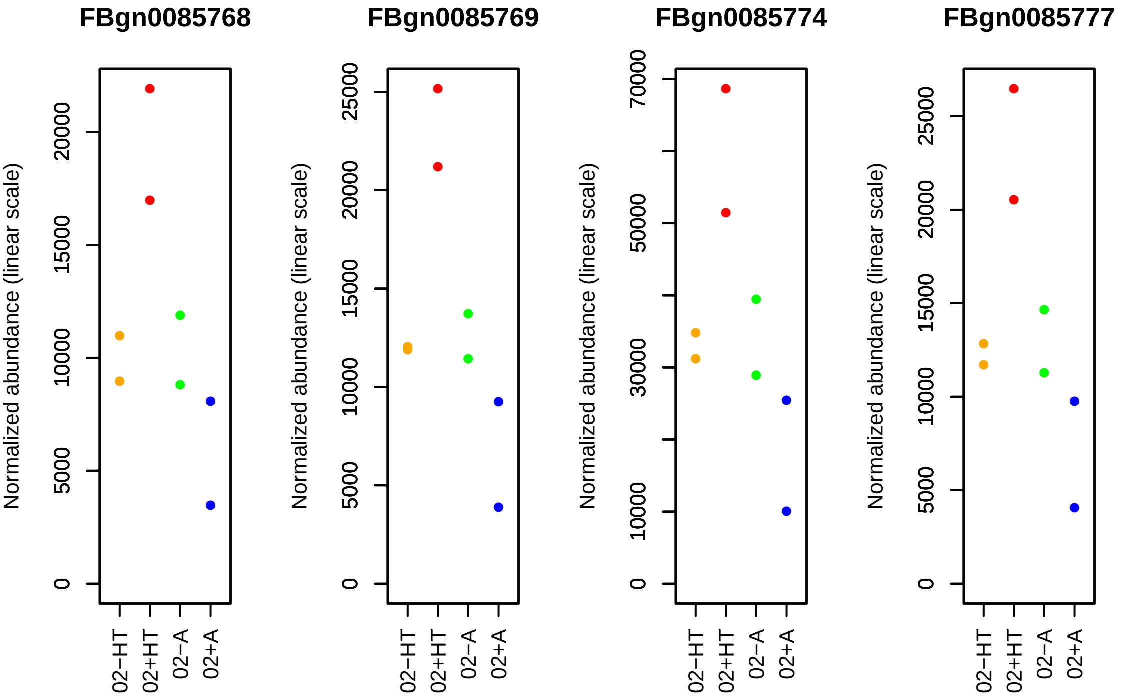
FBgn0085768
FBgn0085769
FBgn0085774
FBgn0085777
Normalized abundance (linear scale)
0
5000
10000
15000
20000
25000
30000
50000
70000
02−HT
02+HT
02−A
02+A

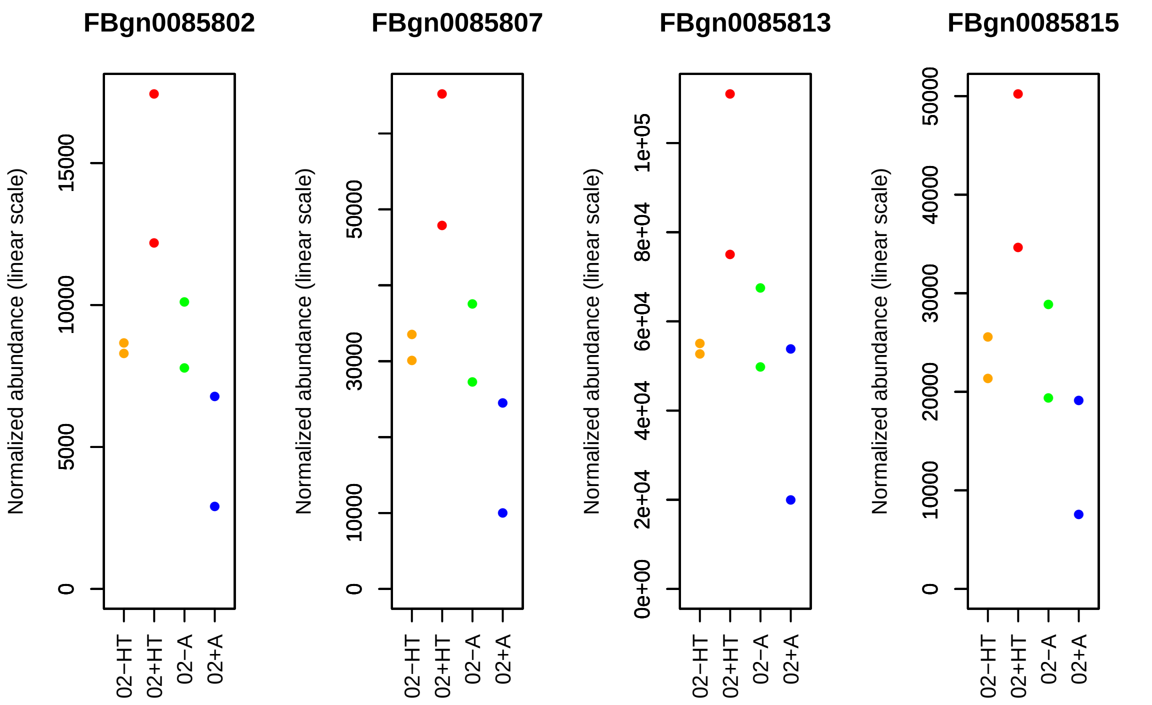

FBgn0085802
FBgn0085807
FBgn0085813
FBgn0085815
Normalized abundance (linear scale)
0
5000
10000
15000
10000
30000
50000
0e+00
2e+04
4e+04
6e+04
8e+04
1e+05
20000
40000
02−HT
02+HT
02−A
02+A

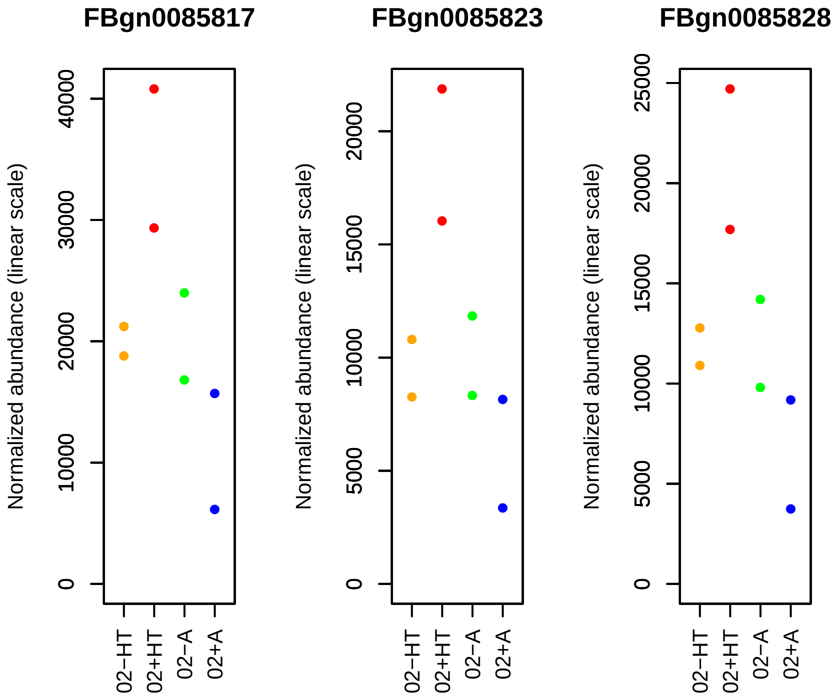
FBgn0085817
FBgn0085823
FBgn0085828
Normalized abundance (linear scale)
0
10000
20000
30000
40000
5000
15000
25000
02−HT
02+HT
02−A
02+A
