# Supplementary material for: Comparison of alternative approaches for analysing multi-level RNA-seq data
Source: PLoS One. 2017 Aug 8;12(8):e0182694. doi: 10.1371/journal.pone.0182694 (PMC5549751; doi:10.1371/journal.pone.0182694)
Supplement: S8 Fig — On the x-axis is the abundance in log2 scale, on the y-axis the coefficient of variation (CV)—the ratio between the standard deviation and the mean. For clarity, the distributions are represented as standard boxplots. The upper panels (A,B,C,D) show the CV for the original data for A samples without rivals (A), A samples with rivals (B), HT samples without rivals (C) and HT samples with rivals (D), respectively. The lower panels (E,F,G,H) give the CV for the same samples, after the subsampling normalization (without replacement). The red horizontal lines indicate 0.5 and 0,25 CV. It is clear that the subsampling normalization reduced the variance between the replicates to < 0.25 CV across most abundances (panels E-H), whereas the CV was much higher across all abundances for the raw data (panels A-D). (PDF) [file pone.0182694.s015.pdf]

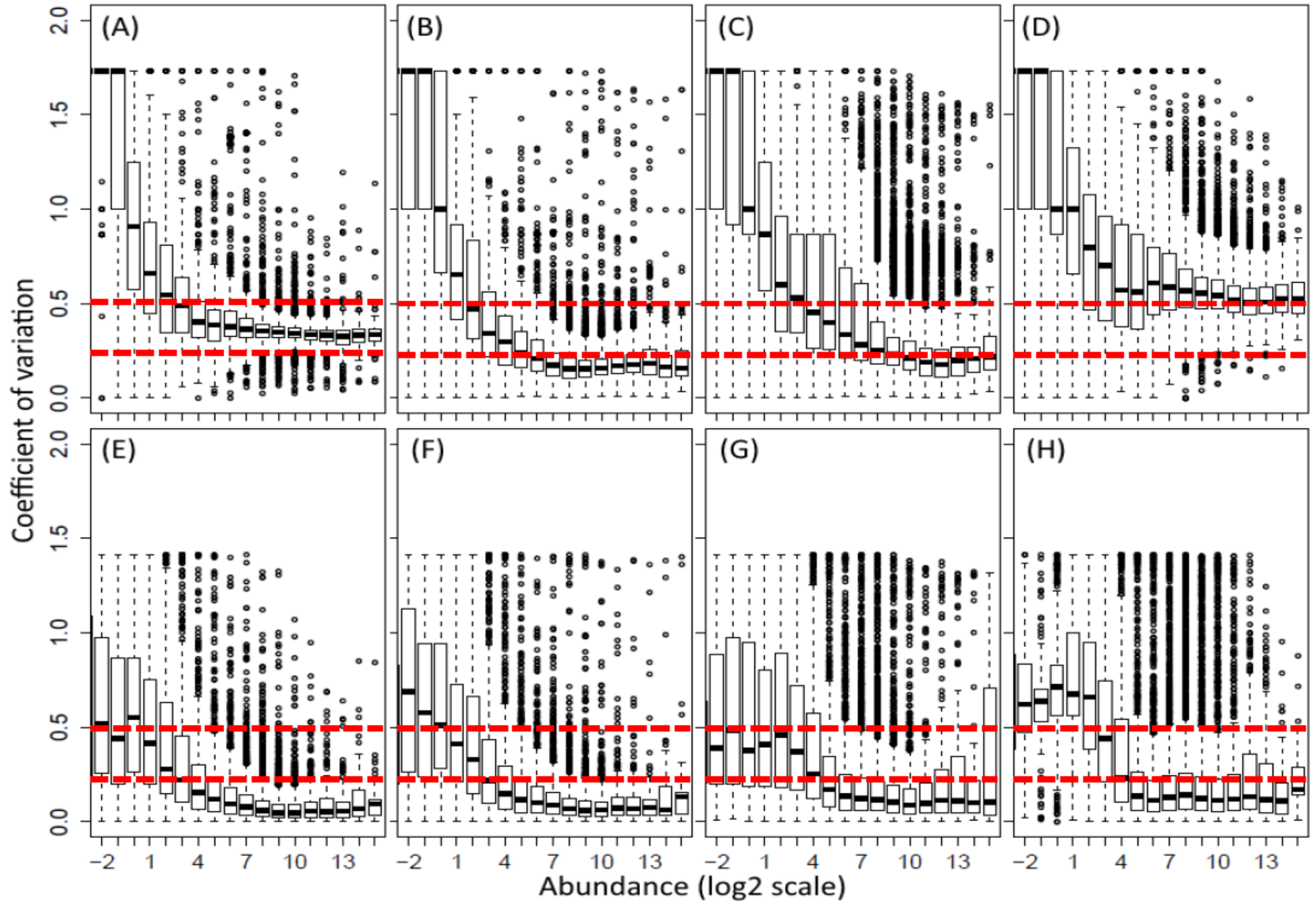

S8 Fig. Comparison of the coefficients of variation across abundance (*D. melanogaster* data). On the x-axis is the abundance in log_2 scale, on the y-axis the coefficient of variation (CV) - the ratio between the standard deviation and the mean. For clarity, the distributions are represented as standard boxplots. The upper panels (A,B,C,D) show the CV for the original data for A samples without rivals (A), A samples with rivals (B), HT samples without rivals(C) and HT samples with rivals (D), respectively. The lower panels (E,F,G,H) give the CV for the same samples, after the subsampling normalization (without replacement). The red horizontal lines indicate 0.5 and 0,25 CV. It is clear that the subsampling normalization reduced the variance between the replicates to < 0.25 CV across most abundances (panels E-H), whereas the CV was much higher across all abundances for the raw data (panels A-D).
