# Supplementary material for: Comparison of alternative approaches for analysing multi-level RNA-seq data
Source: PLoS One. 2017 Aug 8;12(8):e0182694. doi: 10.1371/journal.pone.0182694 (PMC5549751; doi:10.1371/journal.pone.0182694)
Supplement: S9 Fig — In the upper plots we show the coefficient of variation (CV), y-axis vs the average abundance, x-axis, obtained after the subsampling normalization (without replacement) for 5 sequencing pairs (each pair consisted of a Yale laboratory run compared to an Argonne run: A1,A2 = Sample 144; B1,B2 = Sample 153; C1,C2 = Sample 201; D1,D2 = Sample 209; E1,E2 = Sample 210). The F1 and F2 plots show the CV for the combined pairs for the Yale and Argonne replicates, respectively. For individual comparisons we achieved lower CVs in comparison to Zhou et al. 2014 (Nucleic Acids Res, 42:e91) analysis of these sample data. For the sets of lab replicates, Yale and Argonne, respectively, the results using subsampling without replacement are in line with the edgeR results (in red we represent the CV of these data obtained using edgeR, in blue the CV using DESeq2). Based on these distributions we concluded that the samples from the different laboratories could be rendered comparable using our subsampling approach (because it removed technical differences between the two different laboratory runs). In the lower panels, we present the MA plots, after the subsampling normalization, for the same pairs of samples. The tightness of these plots (all falling within ±0.5 OFC) supported the conclusion that the subsampling made these samples derived from sequencing in different laboratories highly comparable. (PDF) [file pone.0182694.s016.pdf]

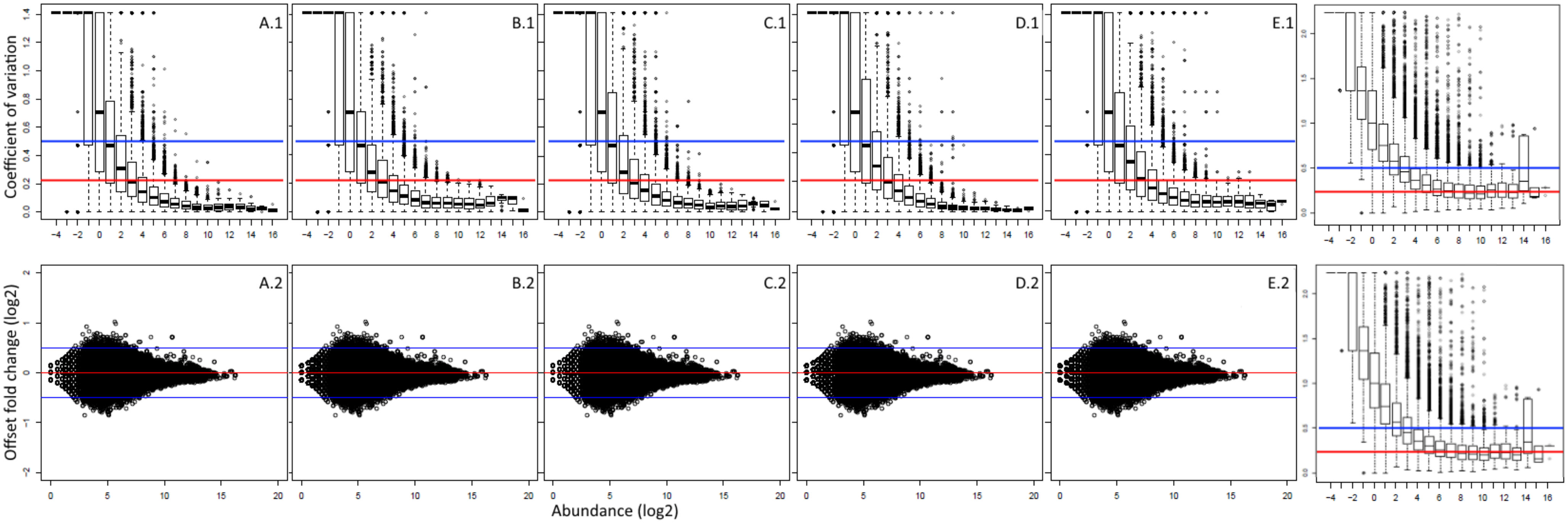

S9 Fig. **Analysis of the effect of the subsampling normalization on technical (laboratory-laboratory) variation in mRNA-seq for human mRNA-seq data (Pickrell et al. 2010, Nature, 464:768-772).** In the upper plots we show the coefficient of variation (CV), y-axis vs the average abundance, x-axis, obtained after the subsampling normalization (without replacement) for 5 sequencing pairs (each pair consisted of a Yale laboratory run compared to an Argonne run: A1,A2 = Sample 144; B1,B2 = Sample 153; C1,C2 = Sample 201; D1,D2 = Sample 209; E1,E2 = Sample 210). The F1 and F2 plots show the CV for the combined pairs for the Yale and Argonne replicates, respectively. For individual comparisons we achieved lower CVs in comparison to Zhou et al. 2014 (Nucleic Acids Res, 42:e91) analysis of these sample data. For the sets of lab replicates, Yale and Argonne, respectively, the results using subsampling without replacement are in line with the edgeR results (in red we represent the CV of these data obtained using edgeR, in blue the CV using DESeq2). Based on these distributions we concluded that the samples from the different laboratories could be rendered comparable using our subsampling approach (because it removed technical differences between the two different laboratory runs). In the lower panels, we present the MA plots, after the subsampling normalization, for the same pairs of samples. The tightness of these plots (all falling within ±0.5 OFC) supported the conclusion that the subsampling made these samples derived from sequencing in different laboratories highly comparable.
